# Cranial Nerve Anatomy Using a Modular and Multimodal Radiologic Approach

**DOI:** 10.15766/mep_2374-8265.11261

**Published:** 2022-06-10

**Authors:** Christopher M. Lack, Joseph Pena, Elizabeth X. L. Grubb, E Shen, Kevin D. Hiatt, Marc D. Benayoun, Fredrick S. Jones, Thomas G. West

**Affiliations:** 1 Assistant Professor, Department of Radiology, Wake Forest School of Medicine; 2 Second-Year Medical Student, Wake Forest School of Medicine; 3 Assistant Professor, Department of Internal Medicine, Wake Forest School of Medicine; 4 Instructor, Department of Radiology, Wake Forest School of Medicine

**Keywords:** MRI, CT, Neurology, Neuroscience, Radiology, Surgery - Neurosurgery

## Abstract

**Introduction:**

Medical students often struggle with learning cranial nerve anatomy. Typically, cranial nerve anatomy is taught using didactic lectures and textbook illustrations, often leaving students frustrated.

**Methods:**

We developed a multimodal radiologic approach to teaching cranial nerve anatomy. First, 150 students were presented with carefully curated preclass material from which to prepare. Next, they received a didactic lecture that was recorded for them to revisit on their own time. Last, students worked in groups in a lab setting with expert radiologists to identify the cranial nerves and related anatomy and learn about some basic pathophysiology. We used a pretest and posttest to examine the effectiveness of our teaching methods and a survey to measure students’ satisfaction.

**Results:**

Student knowledge of cranial nerve structure was significantly improved after our module, with quiz scores increasing from 4.6 to 6.8 out of 9.0 (*p* < .001). In addition, students reported feeling more confident in their knowledge of the material and offered high satisfaction scores.

**Discussion:**

The breadth of knowledge covered during the preclinical training years continues to expand despite stable or even contracted durations of training, requiring knowledge to be delivered in an ever more efficient manner. Ultimately, the multimodal pedagogy used by our resource leads to students who are more confident and engaged in their learning, resulting in increased knowledge.

## Educational Objectives

By the end of this activity, learners will be able to:

1. Identify the major cranial nerve nuclei based on anatomic landmarks on cross-sectional imaging.
2. Name anatomic relationships of cisternal portions of cranial nerves and nearby structures.
3. Identify and name the foramina and corresponding cranial nerves based on their exit from the skull base on cross-sectional imaging.
4. Identify the innervation and action of the extraocular muscles.
5. Describe the anatomic relationships of the cranial nerves and vasculature in the cavernous sinus.

## Introduction

Cranial nerve anatomy is a complex topic that is intimidating and difficult to master for most medical students. Each of the 12 cranial nerves originates in different nuclei in the brain; exits along the undersurface of the cerebrum, brain stem, or upper cervical spinal cord; courses through the cerebrospinal fluid to reach the skull base; exits through various foramina; and finally travels to its respective targets to provide motor and sensory input. Due to this complex design, it is no wonder that the 12 nerves can generate so much angst for the learner and likely contribute to why head and neck anatomy is so difficult.^1^

Traditionally, cranial nerve anatomy is taught through a combination of gross anatomy, didactic lectures, and textbooks that discuss the complex pathway of the nerves, usually with the aid of graphic illustrations. This approach relies heavily on passive learning, which can be an obstacle to mastering the material. In addition, limiting students to traditional resources such as these does not provide realistic visualization of the course of the nerves in their normal state in a clinical setting. An ideal situation would allow students to study the cranial nerves in vivo; however, that is not feasible at this level of training. In fact, very few physicians, unless practicing skull base surgery, will visualize the cranial nerves in vivo using a live patient. Instead, in clinical practice, imaging plays a central role in the screening, diagnosis, and treatment of diseases of the cranial nerves.^2^ For example, neuroradiologists image and examine the cranial nerves during standard clinical practice.^3^ Readily available high-resolution imaging of the cranial nerves could serve as a tool to teach their normal anatomy by providing a visual framework for student physicians in conjunction with gross anatomy labs.^4^ This approach could potentially reinforce and expand on the anatomy knowledge acquired through more traditional methods of teaching.^5^

Despite the heavy use of radiology in clinical practice, most imaging education in medical school occurs during clinical rotations, primarily during the fourth year and in the setting of electives.^6^ In the preclinical years, radiology education is often incorporated into existing courses such as anatomy. Major challenges of implementing radiology in preclinical teaching include time limits in the curriculum and a lack of both resources and radiology faculty participation.^7^ Therefore, resources developed to teach radiology in the preclinical years should be designed with those challenges in mind by using a multimodal approach that can efficiently teach medical students the necessary skills to excel in this topic. Previously, some imaging resources have been developed for the teaching of the abdominal and pelvic, thoracic, and limb anatomy.^8–11^ Self-guided modules such as these can positively impact students’ overall anatomy knowledge and provide evidence that clinical imaging is a viable adjunct in enhancing anatomy education.^12^ However, these self-guided, tutorial-based approaches do not necessarily integrate with team-based learning and instruction or allow active recall of material.

The success of team-based learning modules in incorporating radiology, anatomy, and clinical decision making in preclinical education has been demonstrated in prior studies focusing on pancreatic cancer, vascular anatomy, and head and neck pathology.^13–15^ To our knowledge, this method has never been used for teaching cranial nerve anatomy. The few cranial nerve anatomy teaching modules that exist utilize videos of gross dissection and 2D schematic diagrams, relying primarily on one teaching modality.^16–18^ In contrast, our resource allows for a multimodal approach to teaching cranial nerve anatomy involving a combination of self-guided learning, a faculty-guided video, a faculty lecture, and a team-based faculty-guided lab. This approach allows for collaboration and real-time feedback through interaction with radiology faculty. Our resource builds on the strengths of this approach and adds to the literature with a focus on cranial nerve anatomy. The hybrid lecture format can be easily incorporated into the preclinical neuroscience block and adapted in a variety of settings. At our institution, medical students initially learn about the cranial nerves through more traditional and didactic methods in the gross anatomy course during their first year. Additionally, students gain a basic understanding of CT and MRI. Our module specifically serves as a concise review at a time when students are starting to integrate this knowledge with clinical application. However, some institutions may elect to use this teaching module as an introduction to cranial nerves prior to gross dissection.

Using high-resolution MRI studies of the cranial nerves, the learner actively interacts with the imaging, focusing on identifying anatomic relationships. Many of these images give the student the ability to manipulate the view in real time. In addition to MRI, CT images have been incorporated into the lesson to supplement visualization of anatomic structures relevant to understanding the path of the cranial nerves. This educational experience is intended for students who have already learned the basic concepts of anatomy and physiology of the central nervous system, as well as the basic radiology concepts related to radiography, CT, and MRI. Using pre- and posttests and survey questions, we evaluated the efficacy of our teaching methods to measure the impact.

## Methods

We developed this educational material as part of our institution's preclinical curriculum, to be taught during the second semester of the first year of medical school in the medical neuroscience course. The sessions were developed and taught by neuroradiology faculty. The medical students had already completed their anatomy course, which included 16 hours of an interactive radiology-anatomy lab. The students already had experience scrolling through CT and MRI images of normal anonymized patients using iNtuitionViewer (version 4.4.11.369; TeraRecon, Inc.), a radiology viewing software.

### Lesson Design

We developed this module as a supplement to material already taught during the medical neuroscience block. Our resource was unique in that it focused specifically on neuroanatomy using radiological imaging. The educational material was separated into prelearning material, a cranial nerve didactic lecture, and an interactive neuroanatomy lab.

#### Cranial nerve prelearning

We developed two self-study resources for the students in preparation for the didactic lecture and the cranial nerve lab. These prelearning resources were assigned to the students the week prior to didactic lecture, to be completed on their own time. The first resource was a normal axial MRI sequence in which we labeled the visible cranial nerve segments (predominantly the cisternal segments) and relevant adjacent anatomic structures, such as the skull base foramina through which the nerves travel. This (Appendix A) was made available to the students and was formatted to allow them to scroll through images using either a mouse or the keyboard. The second resource was a 20-minute video on cranial nerve anatomy narrated by a radiology faculty member (Appendix B).

#### Cranial nerve lecture

The 50-minute in-person cranial nerve lecture was a PowerPoint presentation (Appendix C) delivered to the students a few days prior to the neuroanatomy lab. The lecture itself was led by one of the neuroradiology faculty. The lecture covered normal anatomy of cranial nerves I-XII, using imaging (CT, MRI) and pathologic cases to highlight the anatomy and the importance of understanding the anatomy. During this lecture, basic concepts of the anatomic divisions of the cranial nerves, including nuclei, central course, cisternal course, skull base exit sites, and peripheral courses, were introduced to the students. These skills were then built upon during the neuroanatomy lab section of the lesson. The students could attend this lecture in person, watch it live remotely, or watch it at a later time as all lectures were recorded. Attendance was not mandatory.

#### Neuroanatomy lab

The 110-minute in-person neuroanatomy lab was created as a PowerPoint presentation and included lab instructions and six clinical cases with imaging (Appendix D). It was divided into three sections—brain, upper cranial nerves, and lower cranial nerves—and was delivered to the students over two sessions, with the class split in half to accommodate the classroom size. The material was displayed on a screen using a projector. Students were encouraged to work collaboratively during the lab, with breakout groups consisting of three to four students. Normal scans including CT head, MRI brain, and cranial nerves (Appendices E–H) containing high-resolution T2 weighted imaging (fast imaging employing steady-state acquisition) were uploaded to the course page to be accessible for students to download and view using the iNtuitionViewer. Note that images in Appendices E–H here have been converted to scrollable PowerPoint presentation to avoid dependence on third-party software and increase widespread usability. At the beginning of each section, students were given a list of anatomical structures (e.g., Sylvian fissure, midbrain, Meckel's cave, vertebral artery) to identify on MRI and CT imaging. The students worked in groups of three or four to complete the task. During this time, five radiology faculty members circulated around the room to assist the students in identifying the structures. After completion of each section, we presented clinical cases with images. After reviewing the images, the students worked in their small groups to answer questions (e.g., identify the lesion, cranial nerves involved, and anticipated deficits). Attending the lab was mandatory for all students.

### Pretest and Posttest Design

The pretest and posttest (Appendix I) included nine multiple-choice topic-based questions. The questions were designed around the educational objectives we set forth. The pre- and posttests included the same questions and answer choices, but the correct answer was not revealed to any of the questions. The tests were administered using Poll Everywhere (Poll Everywhere, Inc.), an online polling application that students could access from their phone or computer. This platform was chosen because the number of active participants could be tracked in real time and could be easily incorporated in a lecture setting. Participation was voluntary and each student who participated was deidentified and assigned a numerical ID to link the pre- and posttest answers. The pretest was given at the start of the cranial nerve lecture, and the posttest was given at the end of the in-person neuroanatomy lab, which occurred 4 days after the initial didactic cranial nerve lecture. Following the content-related questions, students were asked to rate their confidence in their answers on a 5-point scale (1 = *not confident,* 5 = *very confident*). The test could only be taken in real time, and students who watched the lecture asynchronously were not able to participate. To conduct within-subjects statistical analysis, one additional question was added to the posttest to identify students had who answered the pretest. Students who did not consent to using their responses could still answer the questions during the live interactions. Those who watched the lecture asynchronously could see the questions but could not use the live polling software.

At the completion of the educational block, an anonymous survey (Appendix J) was sent out to all students who had participated in the neuroanatomy lab asking them to rate their confidence level in gained knowledge and satisfaction with the learning session on Likert-type scales using REDCap.^19,20^ A total of 46 students responded to the survey. Students were incentivized to provide feedback with a random drawing for three $10 gift cards.

In preparing this publication, we have included a guide to the appendices to help future users determine where the resource can be incorporated into their curricula (Appendix K).

### Participant Recruitment

The evaluation of students was approved by the Internal Review Board at the Wake Forest School of Medicine. A total of 150 first-year medical students were recruited prior to the beginning of the initial cranial nerve lecture by using email to advertise the sessions. A consent form was included in the email to the students. Prior to answering the pretest and posttest questions, the students gave their consent to participate. Recruitment resulted in the consent of 45 first-year medical students. At the end of the sessions, we were able to electronically match 32 first-year medical students who gave their consent and completed both pretest and posttest questions.

### Statistical Analysis

Individual student performance on the pretest and posttest was compared using deidentified numerical IDs to pair responses. Only data from participants who had completed both a pretest and a posttest were included in the final statistical analysis. A paired-samples *t* test was used to compare pre- and posttests as a whole and to evaluate confidence level. Performance on individual questions was compared using the McNemar test. A *p* value less than .05 was considered statistically significant.

## Results

A total of 32 students attended both the lecture and lab and completed the pre- and posttests.

A paired-samples *t* test was conducted to compare pretest and posttest scores for the tests taken as a whole. There was a significant difference in pretest (*M* = 4.6, *SD* = 1.5) and posttest scores (*M* = 6.8, *SD* = 1.5), *t*(31) = −7.228, *p* < .001, 95% CI: −2.845 to −1.593.

The percentage correct for each question in the pre- and posttests and the *p* value for the McNemar test are shown in Table 1.

**Table 1.**
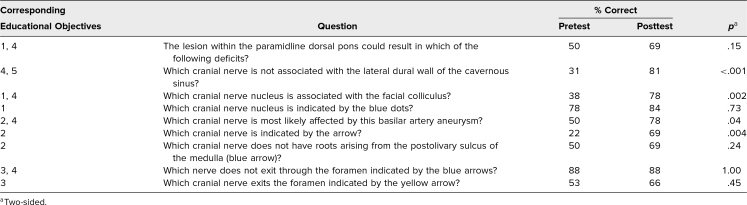
Students’ Performance on Test Questions (*N* = 32)

### Confidence

Students were asked how confident they were in their answers to the test questions on a 5-point scale (1 = *not confident,* 5 = *very confident*). The Figure shows the students’ pretest and posttest confidence-rating percentages. A paired-samples *t* test showed that posttest confidence level (*M* = 3.7, *SD* = 0.9) was significantly higher than pretest confidence level (*M* = 2.7, *SD* = 0.9), *t*(31) = −7.517, *p* < .001, 95% CI: −1.477 to −0.846.

**Figure.**
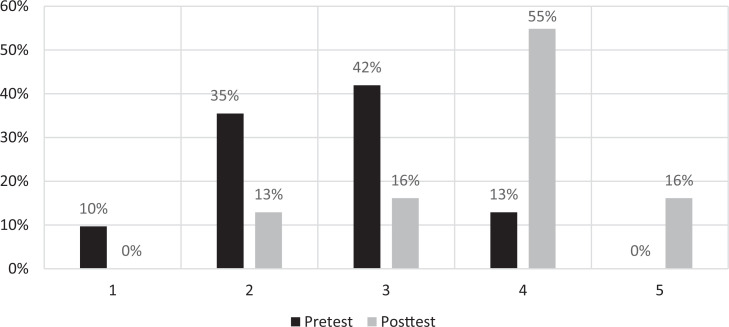
Students’ pre- and posttest confidence-rating percentages (*n* = 31). Confidence was rated on a 5-point Likert scale (1 = *not confident,* 5 = *very confident*).

### Satisfaction

Students were asked about their perception of the quality of the learning activity and their confidence in having learned the material. The percentage of *agree*/*strongly agree* or *excellent*/*good* responses is listed in Table 2.

**Table 2.**
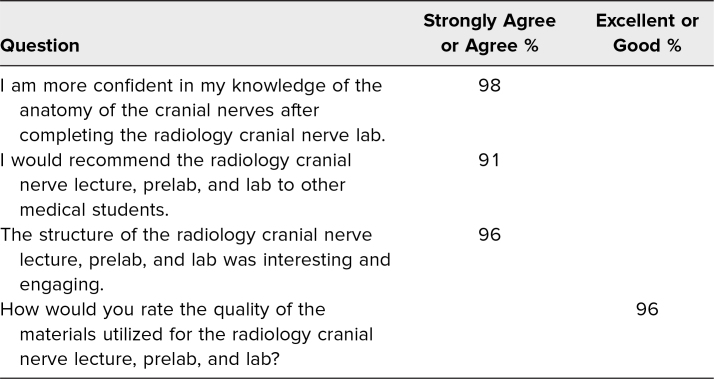
Students’ Agreement and Rating Scores for Survey Questions After Completion of the Course (*n* = 46)

## Discussion

We successfully implemented a radiologic approach for teaching medical students the complex anatomy of the cranial nerves that increased their knowledge base and confidence in an innovative and engaging manner. It was highly rated by the participants and significantly increased their knowledge of cranial nerve anatomy. This was accomplished by utilizing a didactic lecture that set the framework for the curriculum, a self-guided review of labeled radiologic slides and a video walk-through of the cranial nerves, and finally a live laboratory where cranial nerve imaging was reviewed with expert assistance from neuroradiology faculty. The material created will be used yearly to teach medical students and can easily be adapted to suit other learners, such as radiology residents, nonradiology residents, and fellows. Cranial nerves are universally taught in medical school, and the work presented here could benefit others exploring new ways to deliver the material.

Over the last 5 years, there has been an increase in radiology representation in the preclinical curriculum at our institution. Led by the radiology department, the addition of eight radiology labs to the anatomy curriculum has been an extremely rewarding experience. It has allowed increased face-to-face contact with the students and faculty. It has also increased educational experience for residents and fellows in the radiology department, who often assist during the labs. The biggest benefit has been the early introduction of radiology as a specialty, showcasing its use in diagnosis and treatment and its utility as a tool for learning the pertinent anatomy of the body. We are continually evaluating and editing the material to better serve students as they progress through the medical school experience. To appropriately deliver this material, it is vitally important that educators be familiar with the curriculum students have received prior to this exercise. This requires great organizational skills and frequent communication between educators to make sure the material presented is not too repetitive and not too advanced, since lectures and labs in medical education are taught by a wide range of professionals.

The creation of the teaching materials, lab, and video walk-through of the cranial nerves was time intensive. The editing phase of the video was particularly time consuming, but having a script and storyboard detailing the content covered can save significant time during editing and decrease the need for rerecording and adding material. The prerecorded video content allowed the students to efficiently prepare for the laboratory sessions at their convenience, enabling a greater breadth of material to be covered. It is our experience that many students routinely watch prerecorded material at an accelerated rate of two to three times the recorded speed, often watching multiple times. Delivering the lab session required significant participation from neuroradiology faculty, and participation was high due to the high interest in teaching among our faculty and the great support from our department. We do not believe five faculty members are needed for each lab session with 75 students. Similar success could probably be achieved with a lower expert-to-learner ratio.

Evaluation of the effectiveness of the course was done by comparing the pretest and posttest results. All questions for which the pretest score was ≤50% showed statistically significant improvement on the posttest. For most of the questions for which the pretest percentage correct was >50%, an increase in percentage correct was seen, but it was not statistically significant. It is possible that analysis was underpowered to find a significant change at these levels and that a higher level of student participation may have shown a significant difference. Also, the high scores on two pretest questions (78% and 88%) suggest prior exposure to some of the learning material, which could have blunted the performance response.

There were some limitations to our assessments and educational efforts. First, attendance at the initial didactic cranial nerve lecture was not mandatory, with students having the option of viewing the recorded lecture asynchronously. Giving the initial pretest at the lecture reduced the power of our statistical analysis as only 32 students out of a class of 150 were able to successfully complete the pretest and posttest. Requiring real-time student participation would significantly improve analysis of our material but would reduce the scheduling flexibility for students. Second, there may have been a bias in performance for those who attended the pretest lecture, though we have no reason to assume that students who did not attend to pretest lecture would have shown dissimilar knowledge gain or learning satisfaction results compared to the group analyzed.

In the future, we would like to remove the brain section from the lab and expand the cranial nerve component. Our evaluation indicated students had prior knowledge of cranial nerve anatomy, which presents an opportunity to go into greater depth. If there is time in the lecture schedule, we would like to develop a similar project for teaching the anatomy of the brain using radiology. Our presentation has already piqued interest in many faculty and trainees alike to take this approach in various directions, whether it be practical application in neuroradiology, creation of further educational modules, expansion of content material, or the basis for further specialized development for neurosurgical and ear, nose, and throat training applications. One extension would be adding virtual reality content, which would have the synergistic benefit of improving visualization of anatomy and facilitating distance learning. Another would be to utilize 3D printing developed from the radiology image data set for teaching cranial nerve anatomy.^21^ To improve the application of this learning material, a module could be created for use with cadaveric anatomy labs.

This multimodal radiologic approach to cranial nerve anatomy provides several benefits to the students. Anatomy requires many lower-order processes in Bloom's taxonomy, including remembering and understanding.^22^ Prelearning material focuses on these processes by allowing students to become familiar with concepts before class on their own time. Recorded didactic lectures reinforce the prelearning material and give in-person students a chance to ask questions, while allowing attendance to be optional allows students to review material multiple times at their own pace. The lab portion of our module addresses higher-order processes, including application, evaluation, and analysis. The active team-based learning setting allows students to put their knowledge to the test by collaborating with classmates. Ultimately, this multimodal approach leads to students who are more confident and more engaged in their learning.

## Appendices


Self-guided Anatomy Review.pptxCranial Nerve Video.mp4Cranial Nerve Lecture.pptxNeuroanatomy Lab.pptxNormal MRI and CT Scans - CT Bone Axials.pptxNormal MRI and CT Scans - T1 Sagittal.pptxNormal MRI and CT Scans - T2 Axial.pptxNormal MRI and CT Scans - T2 SPACE Axial.pptxPre- and Posttest.pptxSatisfaction Survey.docxAppendix Guide.docx
*All appendices are peer reviewed as integral parts of the Original Publication.*
