# Supplementary material for: Cranial Nerve Anatomy Using a Modular and Multimodal Radiologic Approach
Source: MedEdPORTAL. 2022 Jun 10;18:11261. doi: 10.15766/mep_2374-8265.11261 (PMC9184306; doi:10.15766/mep_2374-8265.11261)

## Slide 1
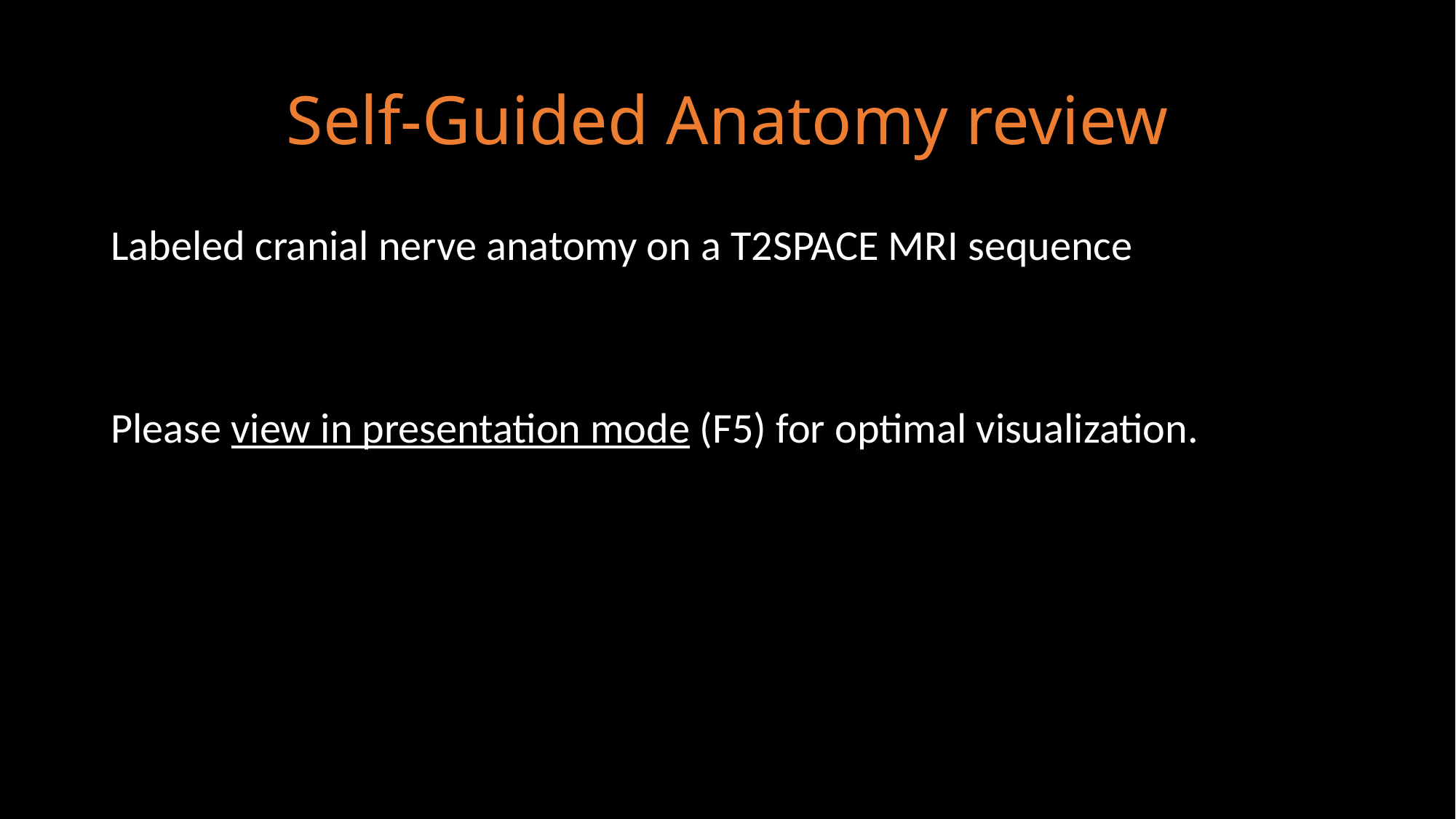

# Self-Guided Anatomy review
Labeled cranial nerve anatomy on a T2SPACE MRI sequence
Please view in presentation mode (F5) for optimal visualization.

## Slide 2
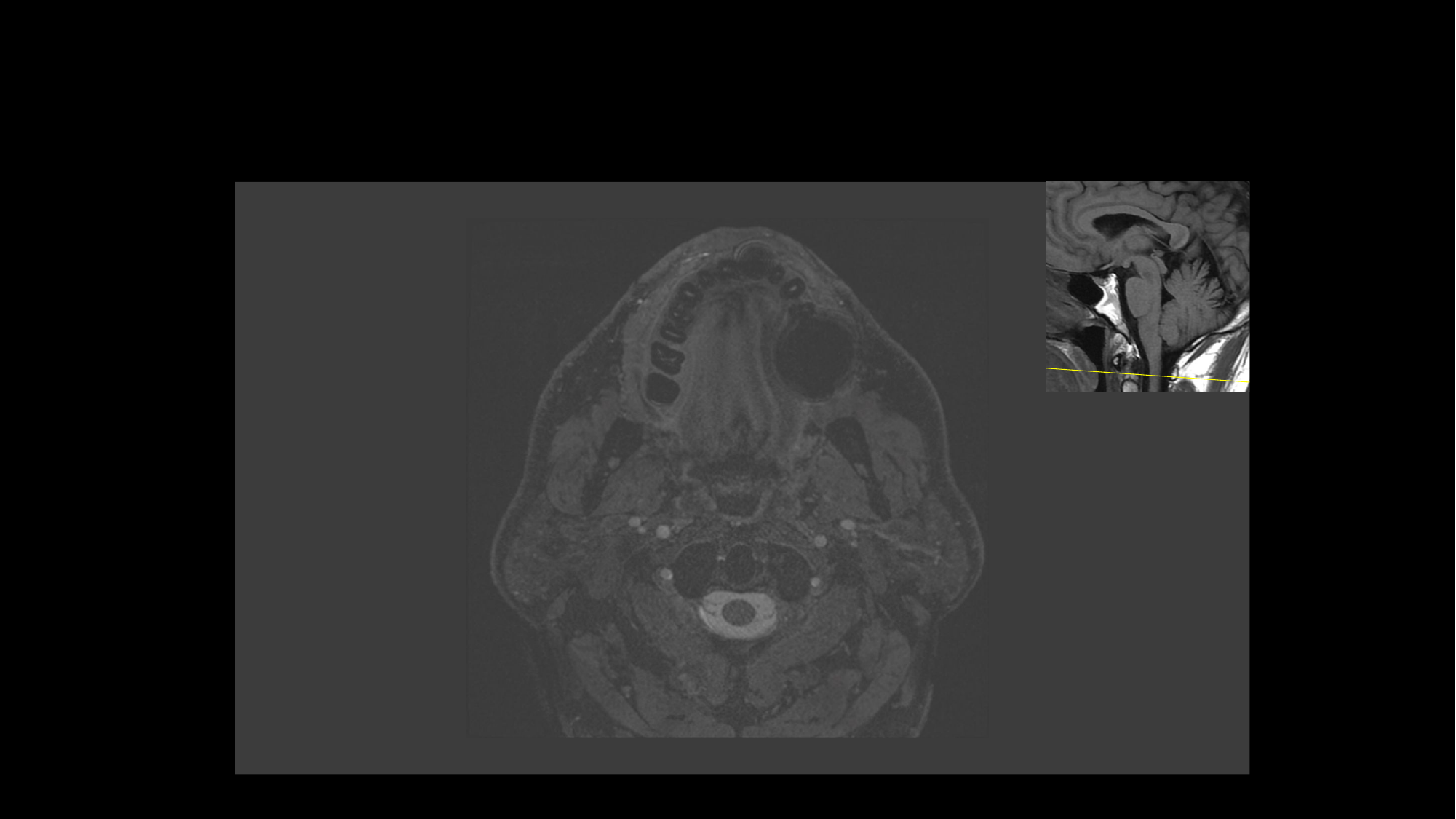

## Slide 3
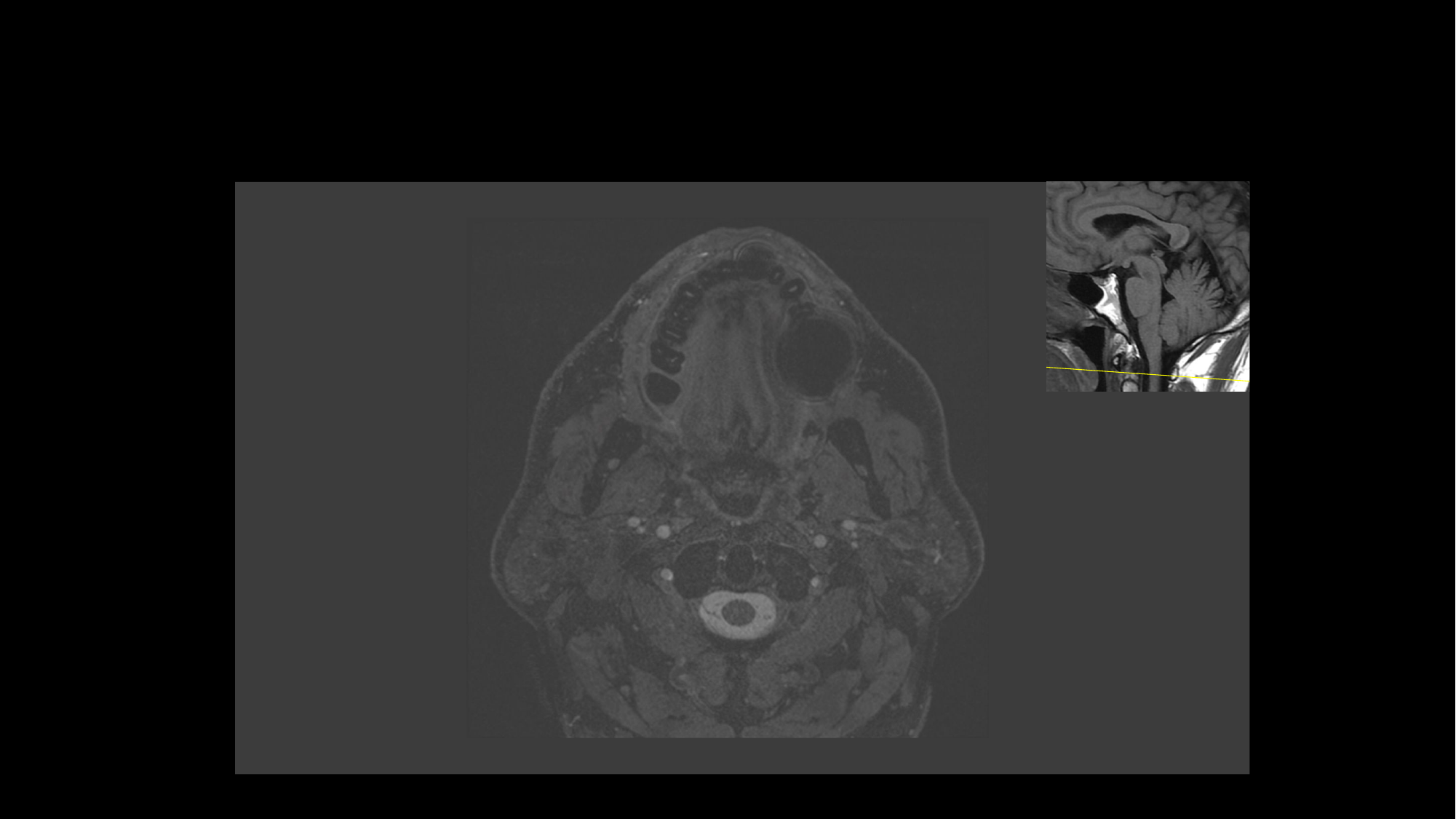

## Slide 4
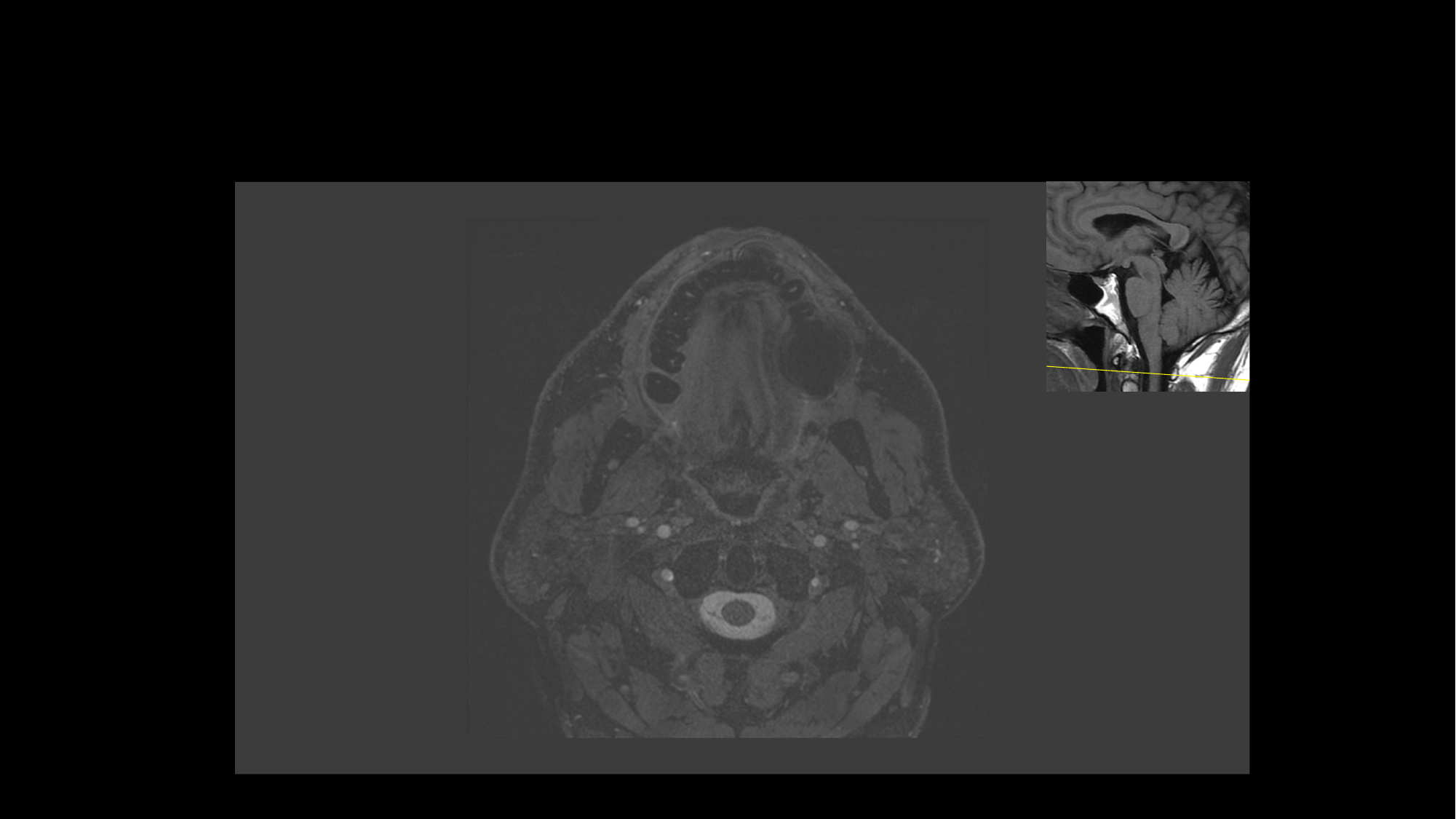

## Slide 5
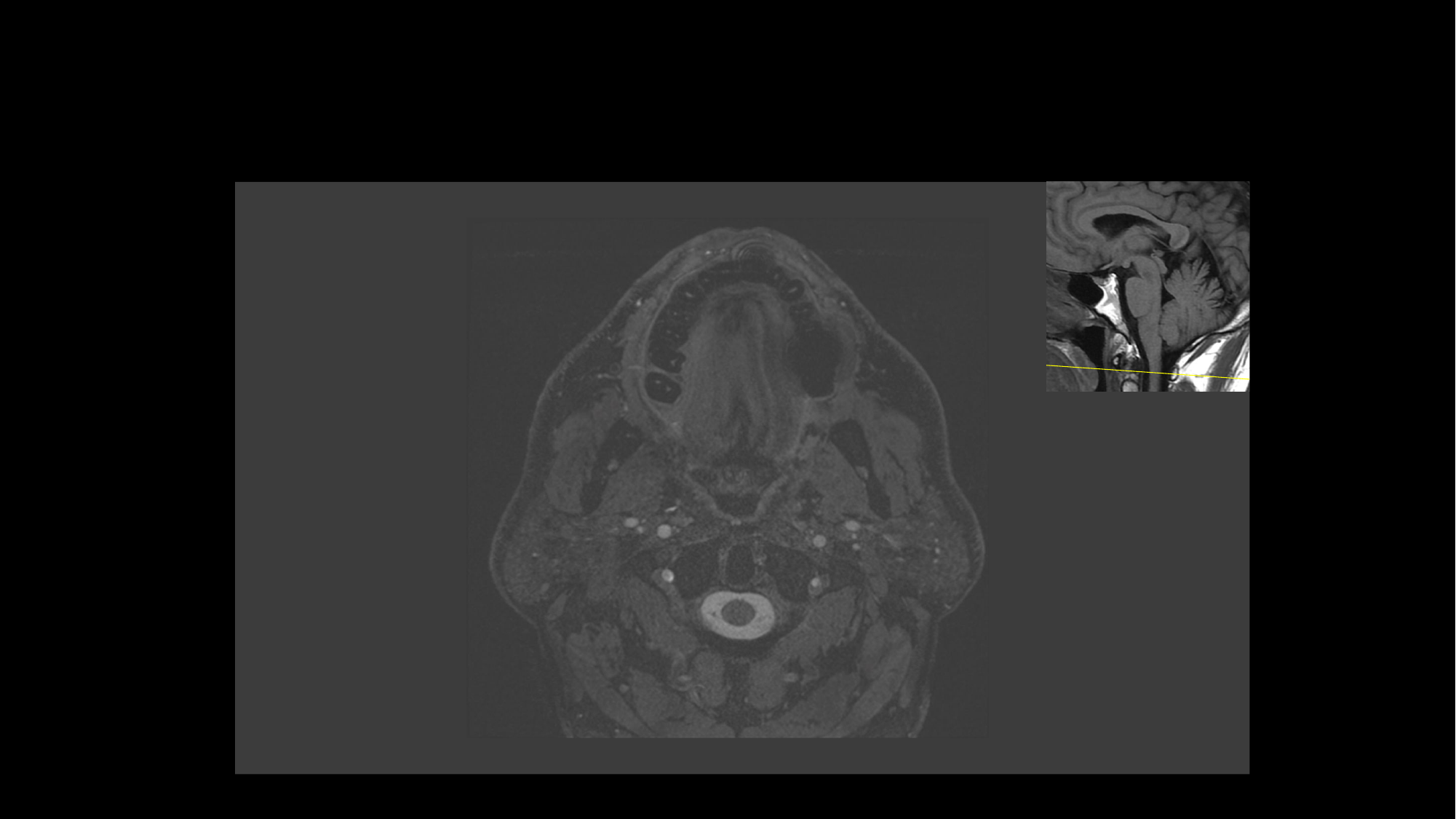

## Slide 6
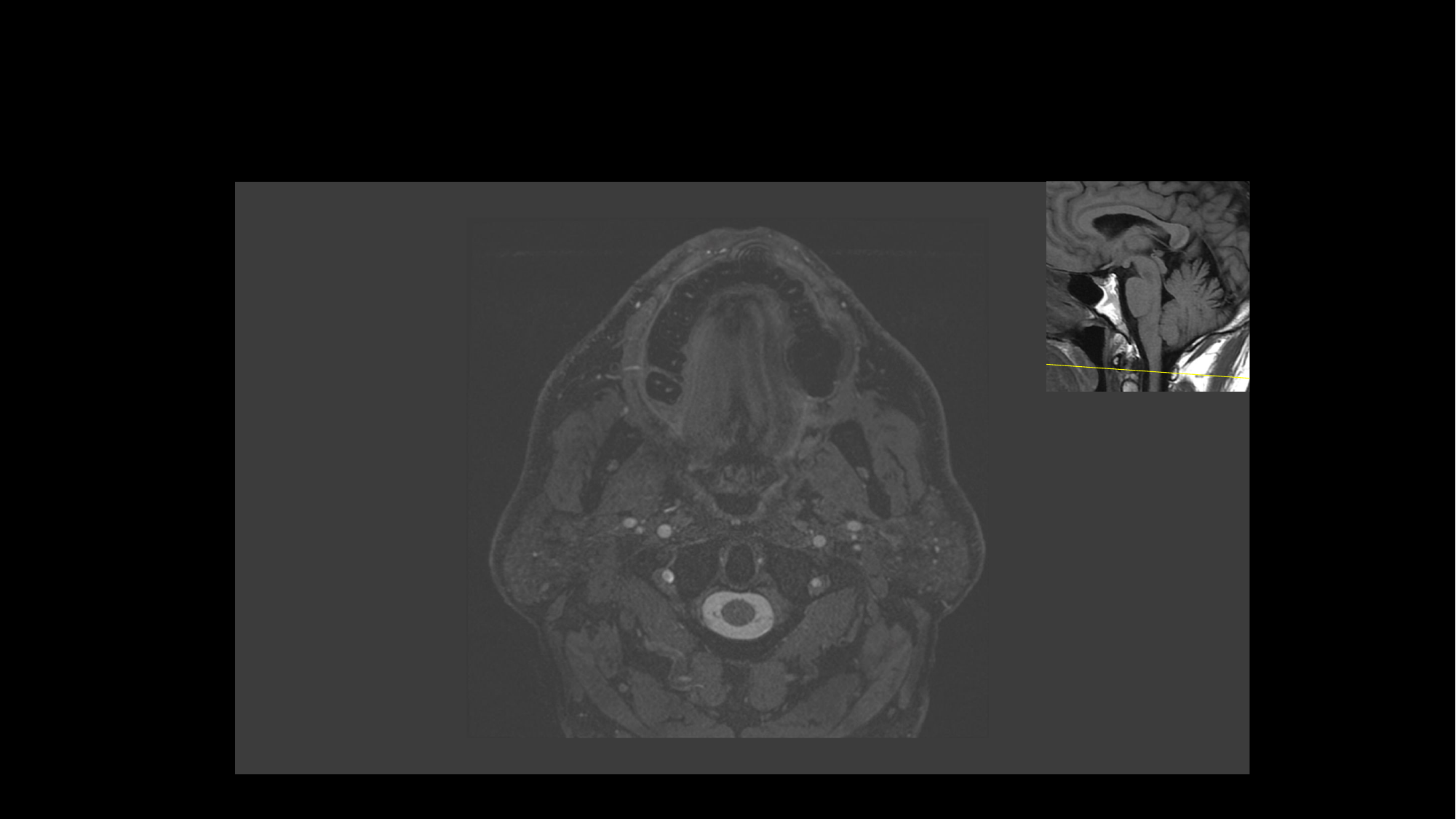

## Slide 7
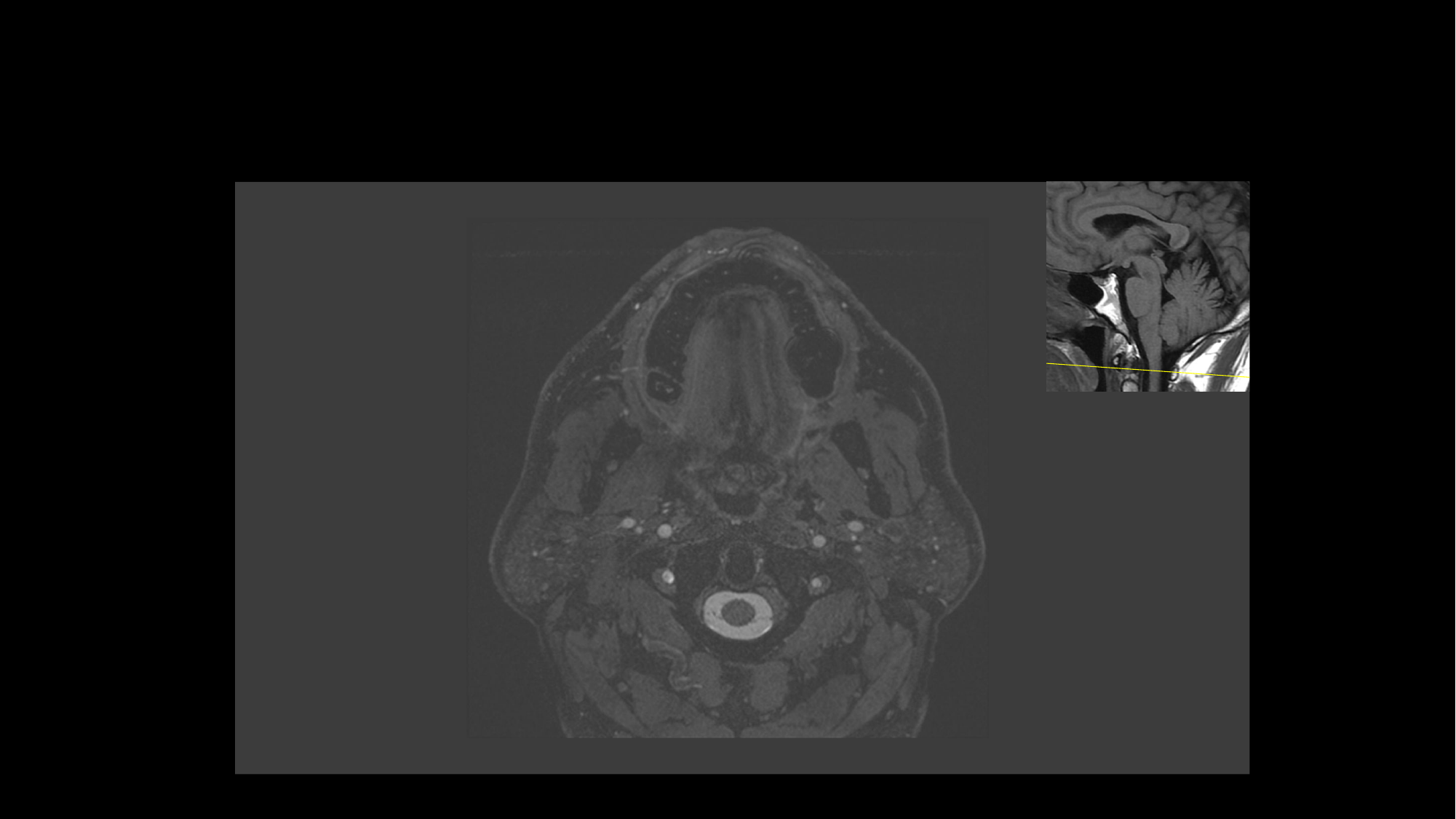

## Slide 8
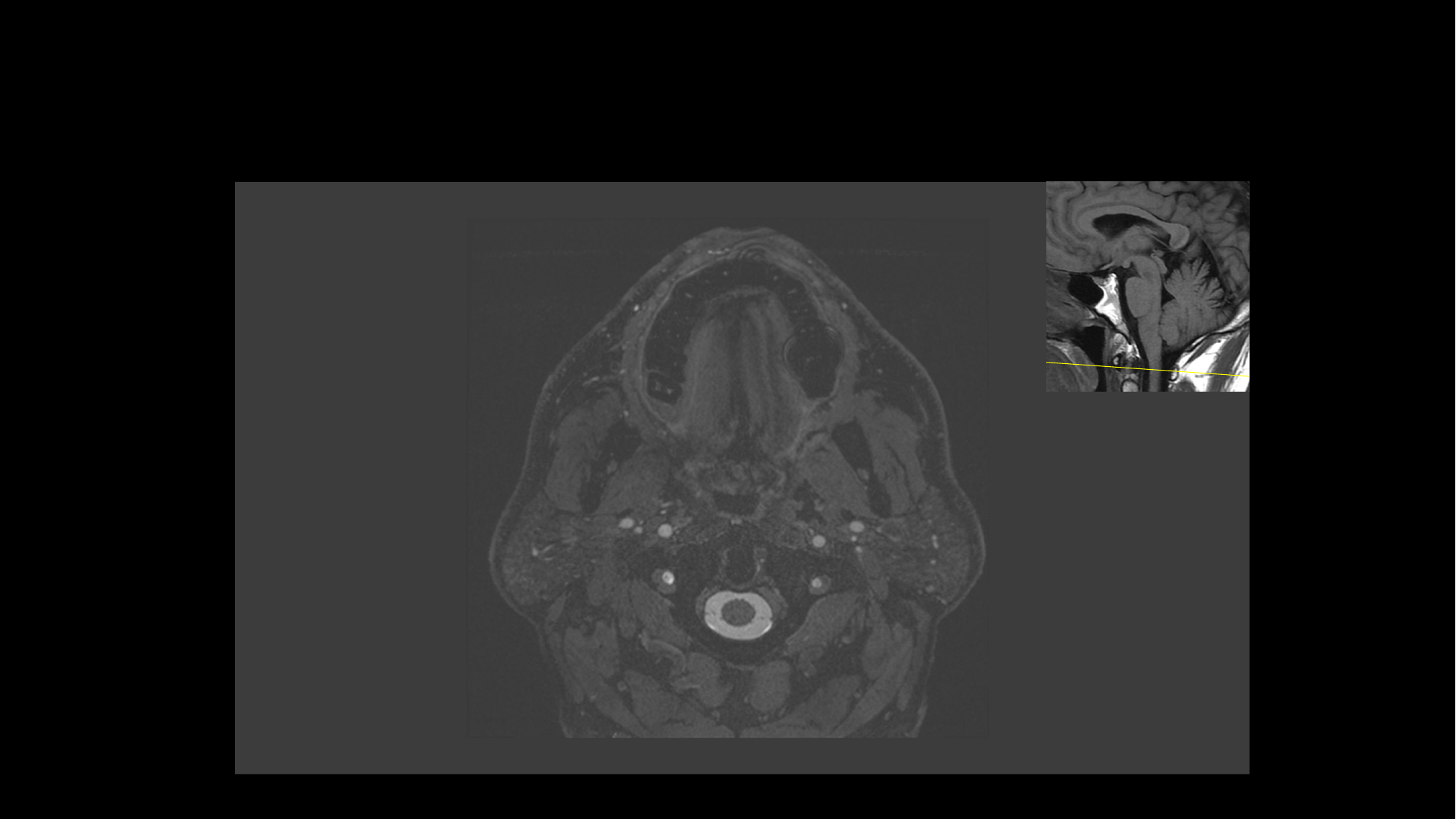

## Slide 9
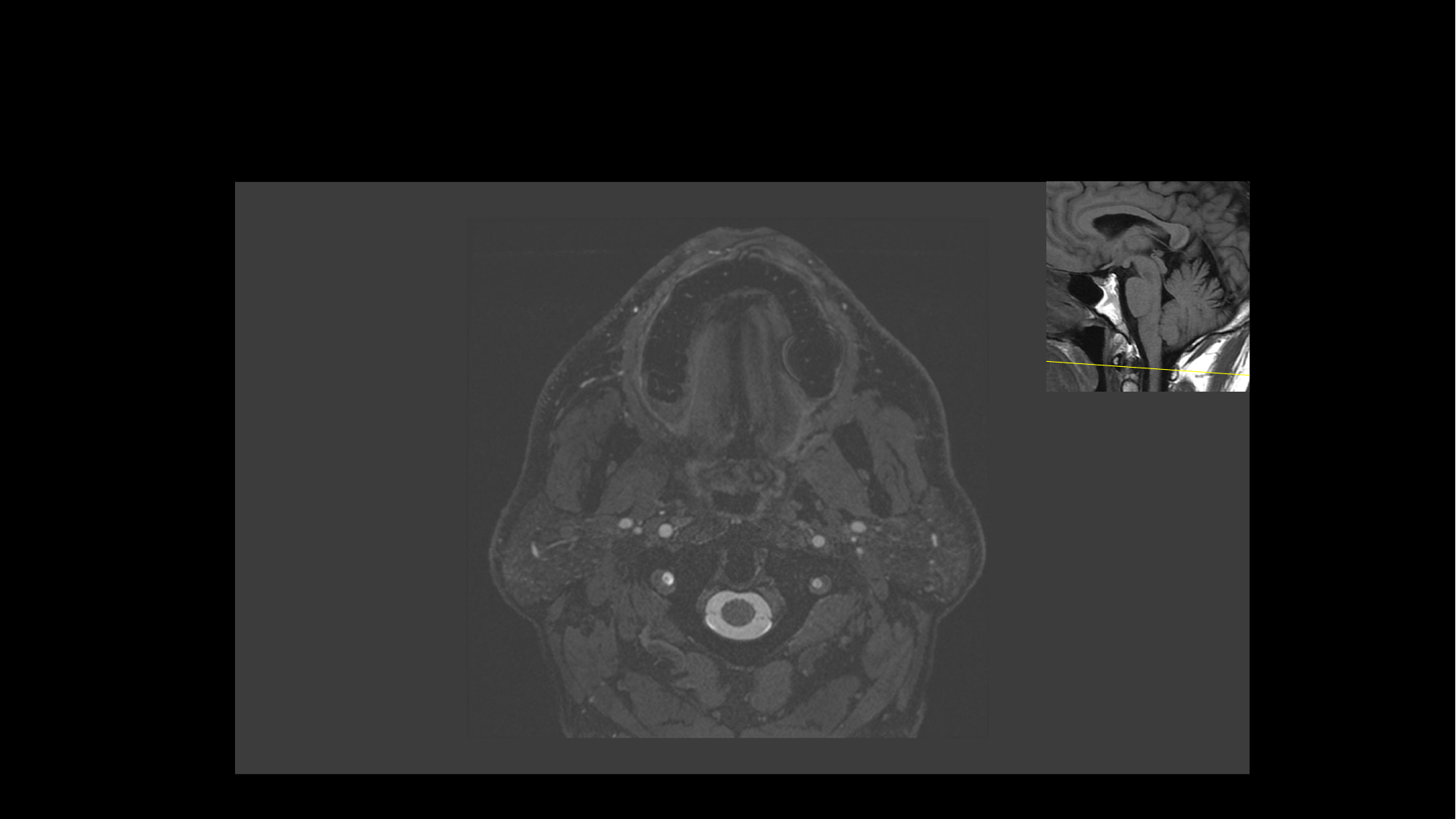

## Slide 10
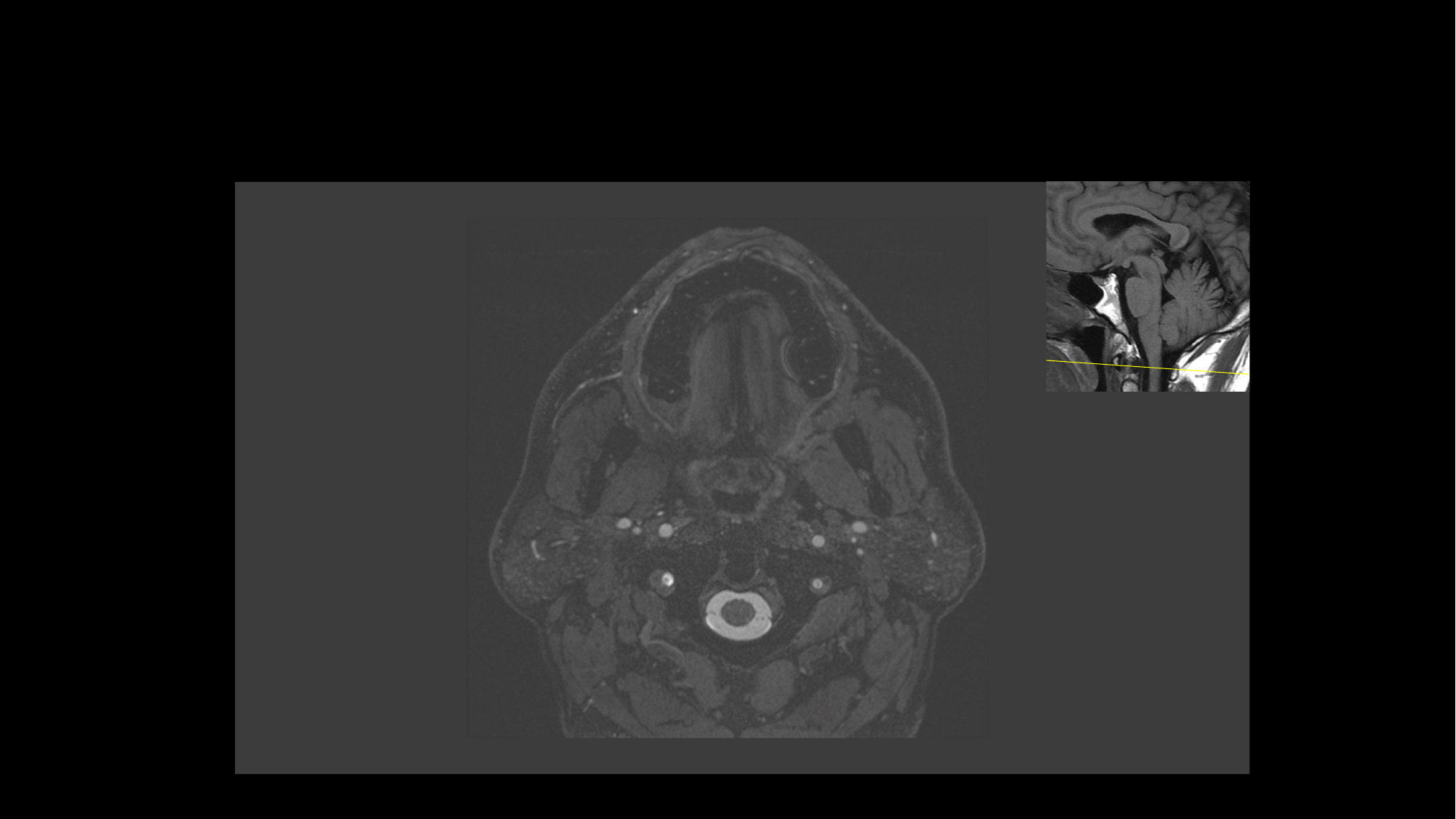

## Slide 11
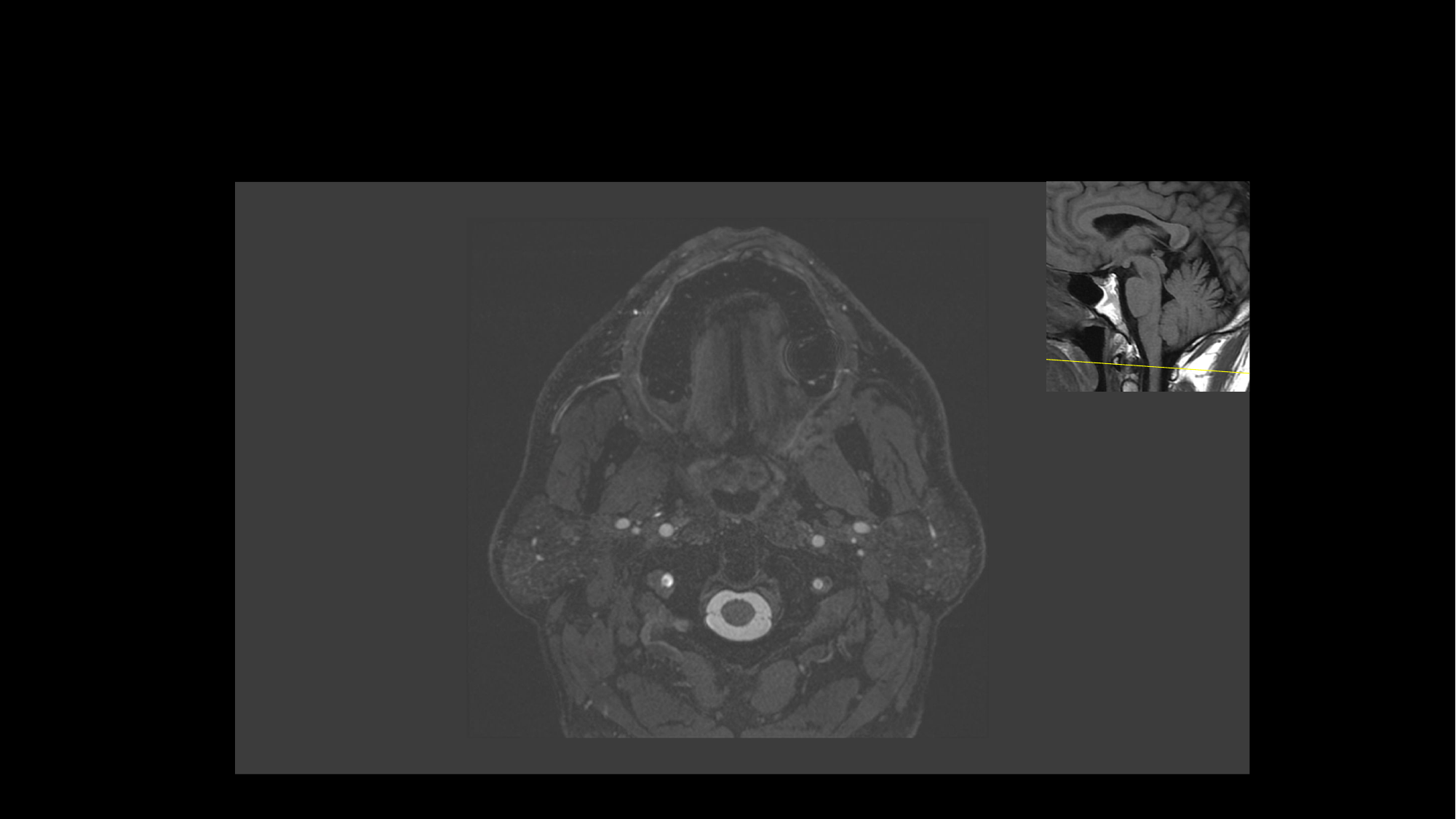

## Slide 12
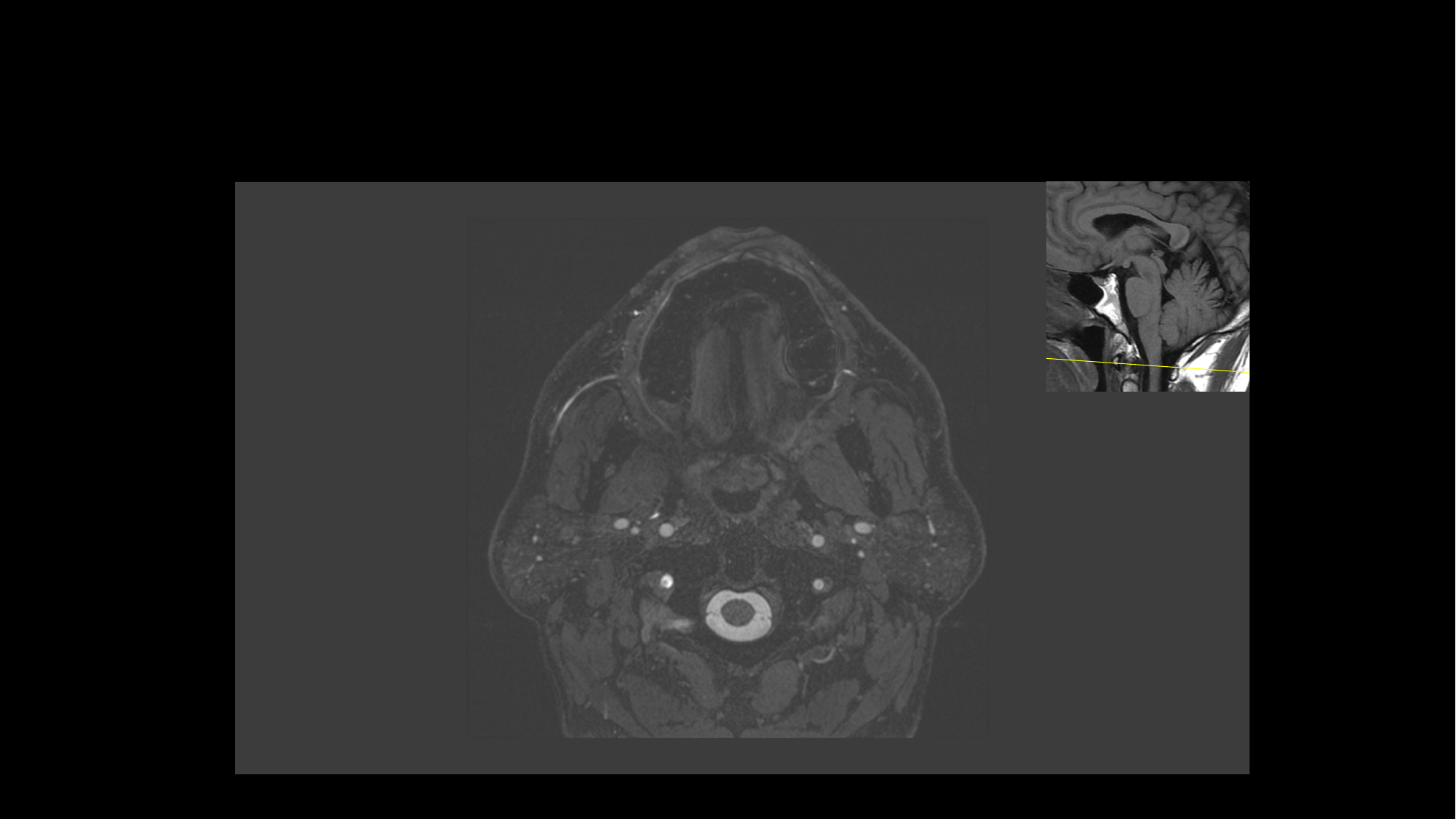

## Slide 13
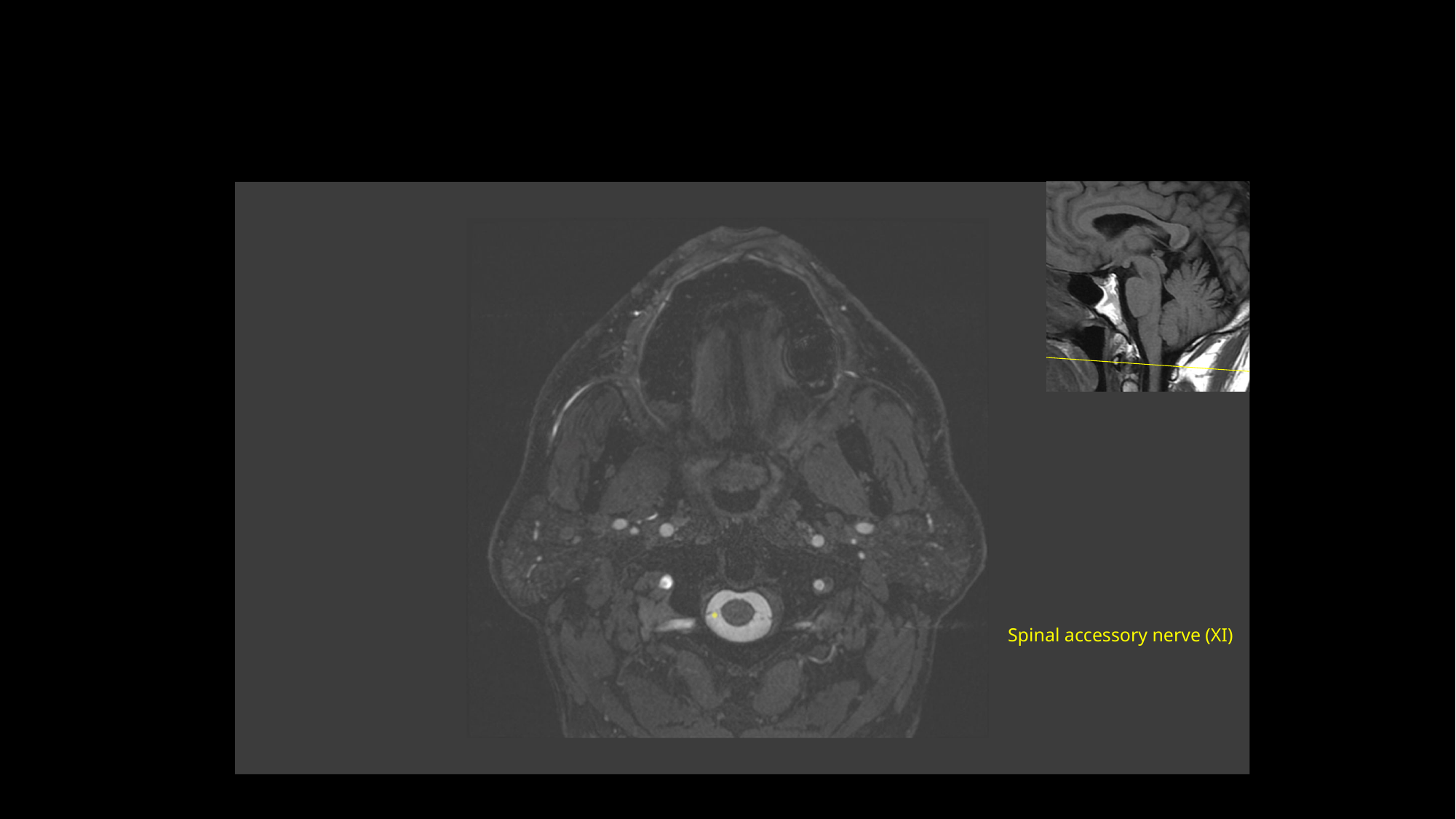

Spinal accessory nerve (XI)

## Slide 14
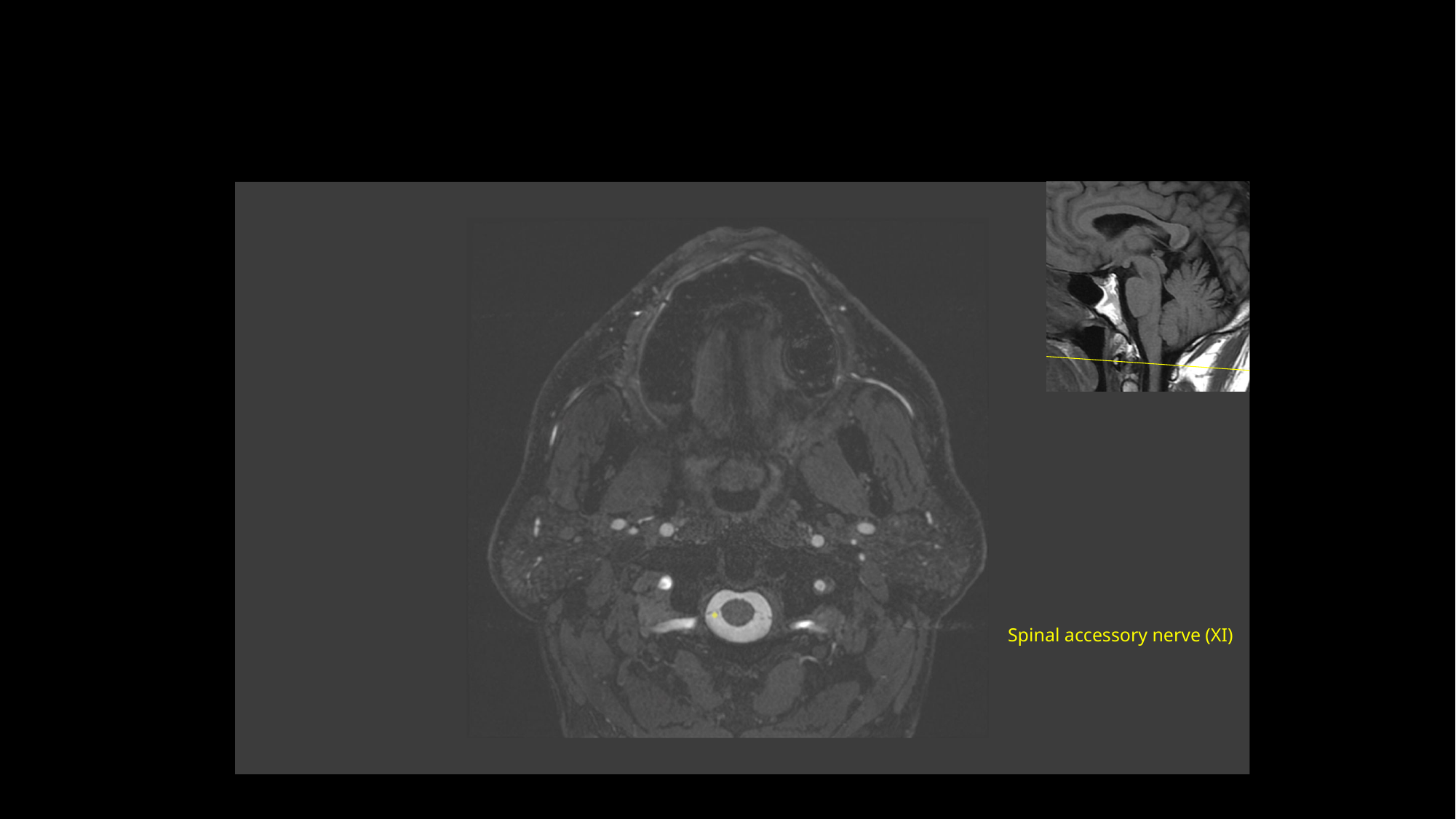

Spinal accessory nerve (XI)

## Slide 15
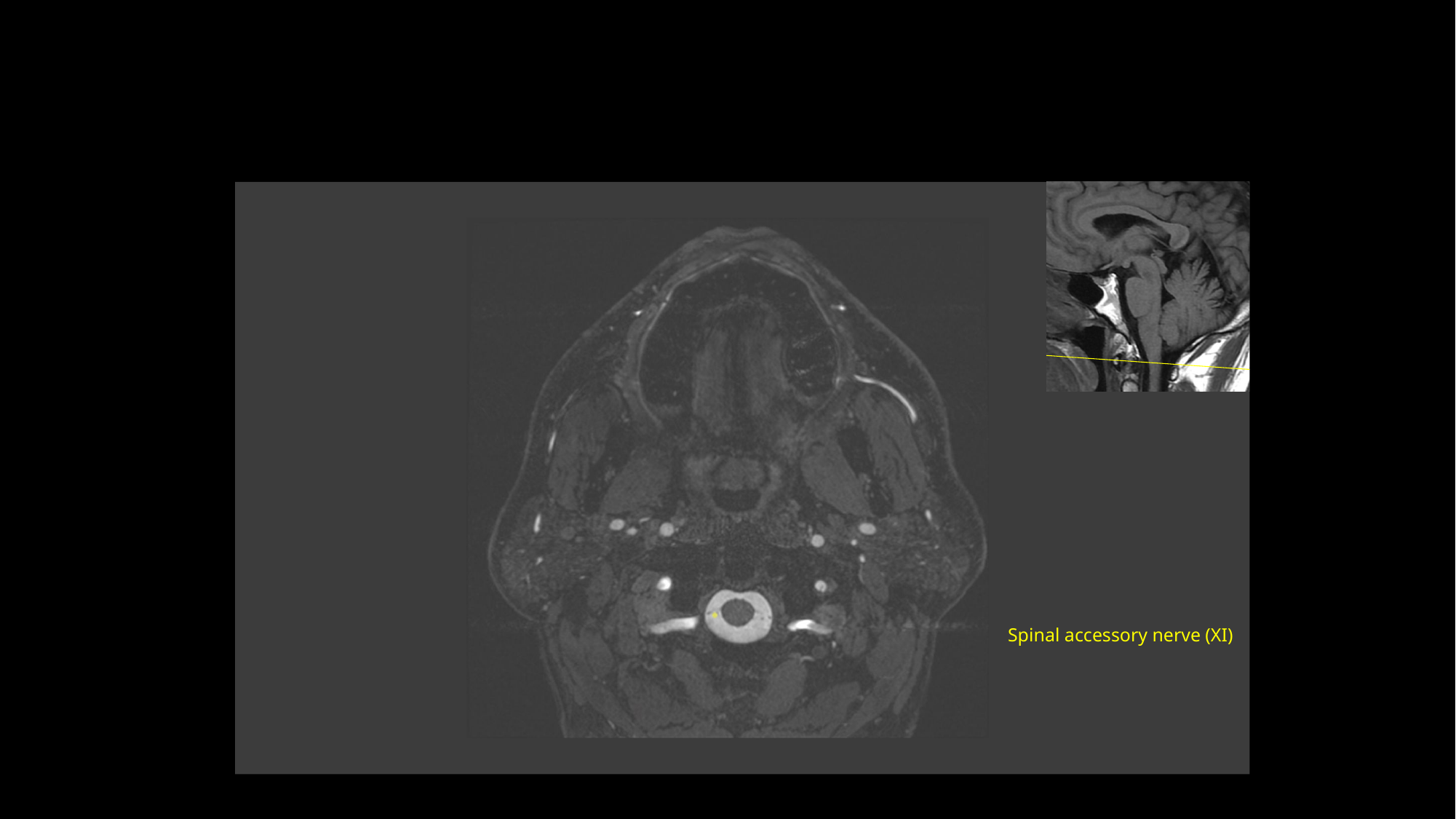

Spinal accessory nerve (XI)

## Slide 16
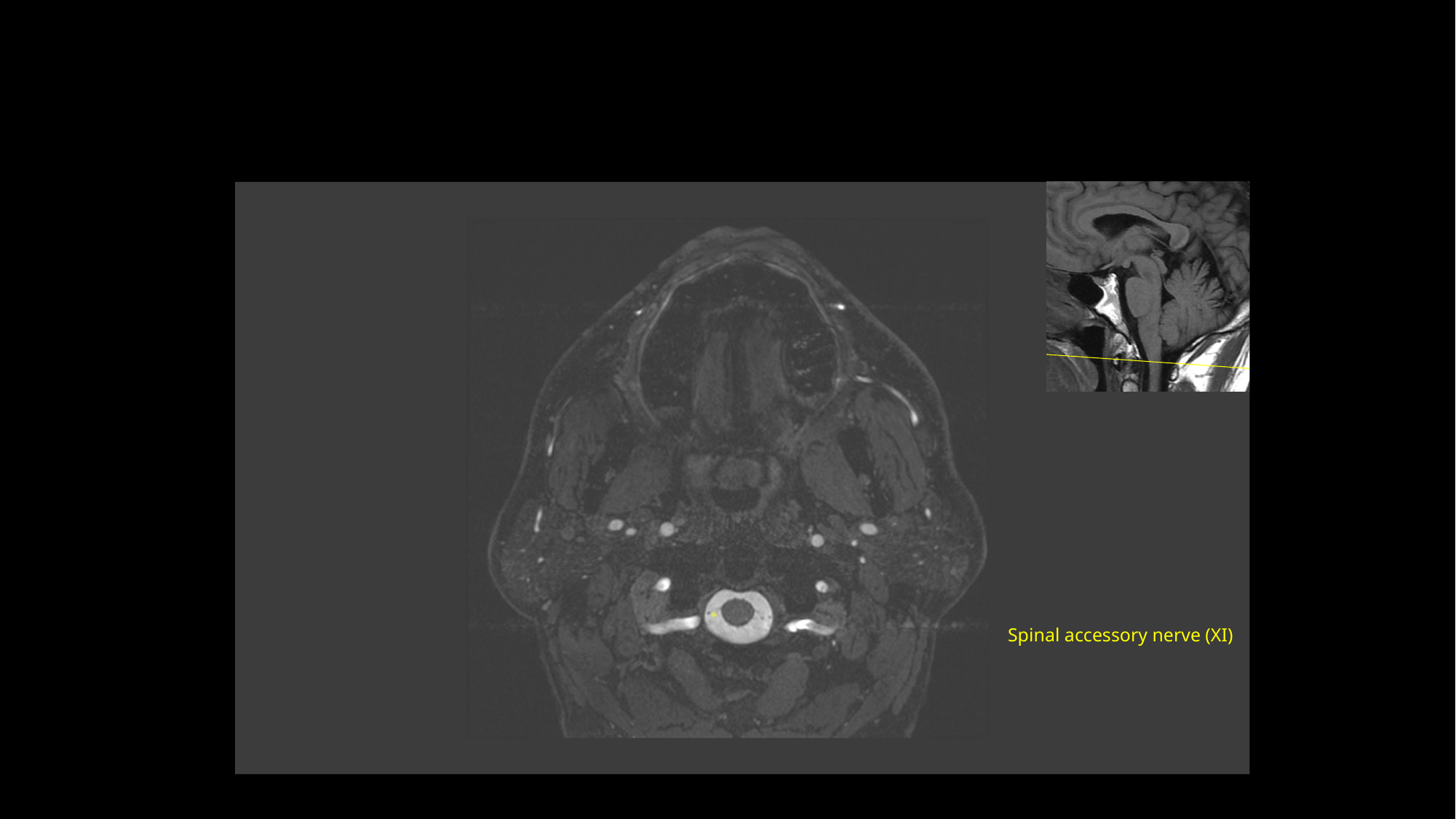

Spinal accessory nerve (XI)

## Slide 17
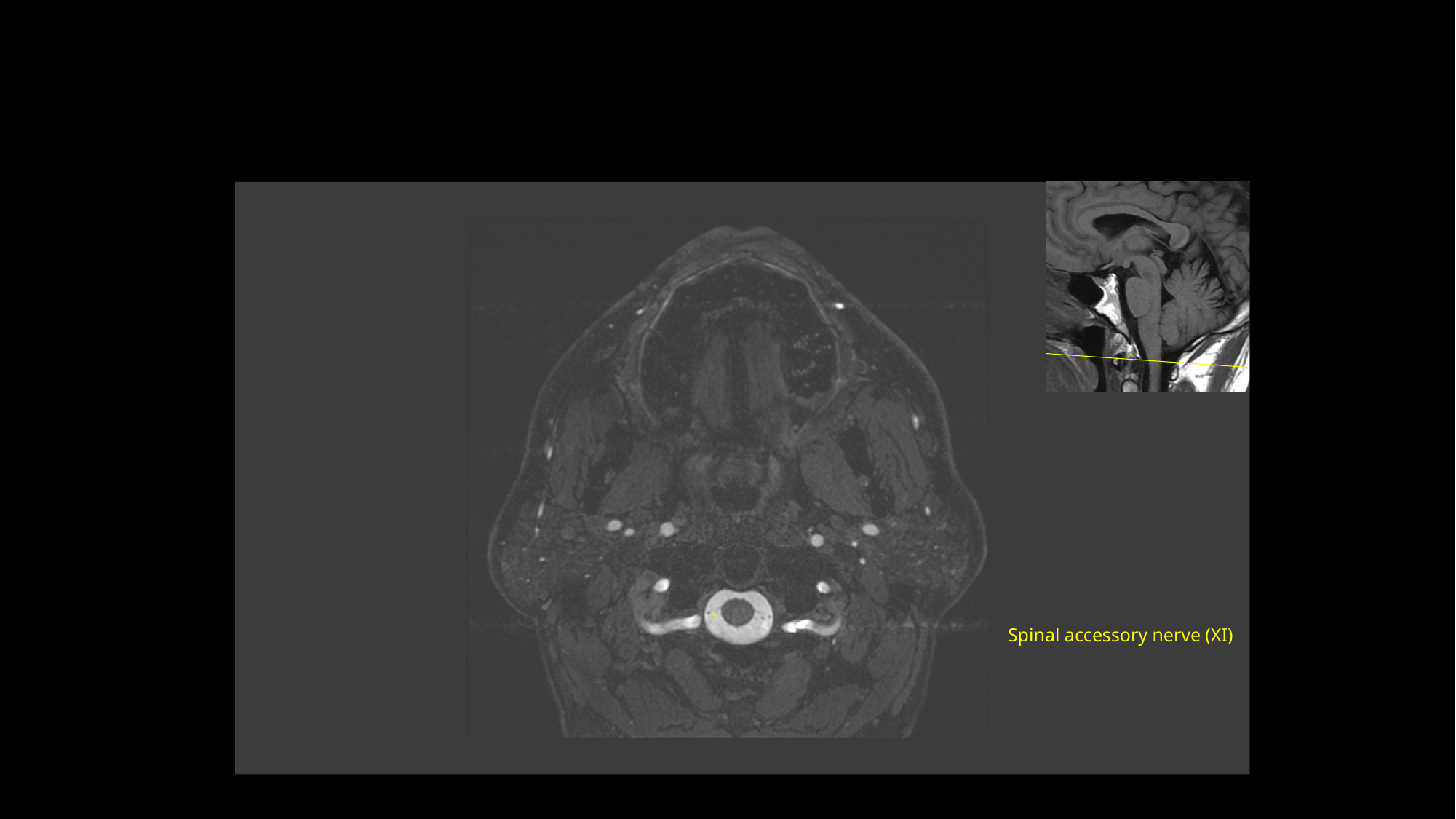

Spinal accessory nerve (XI)

## Slide 18
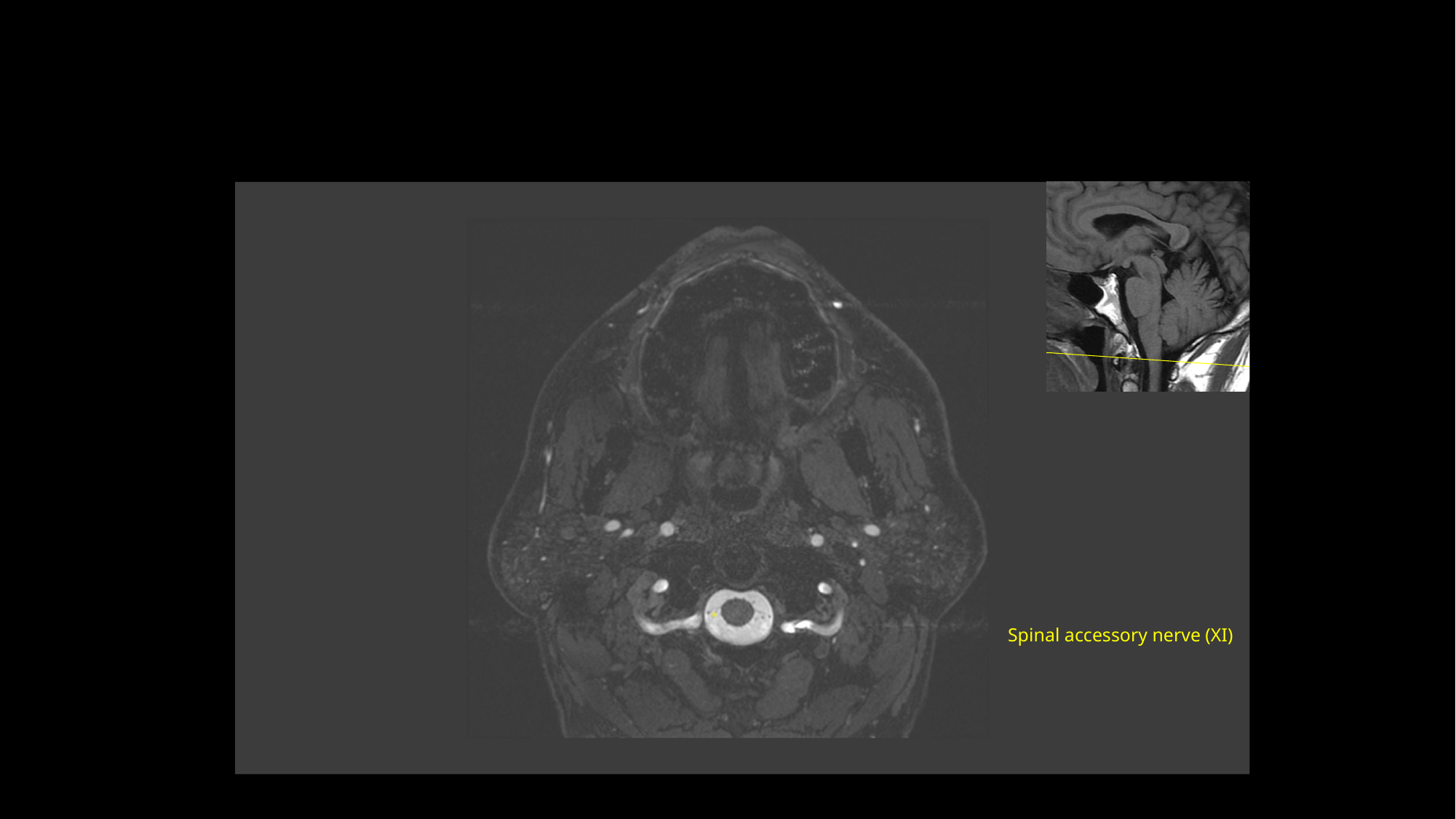

Spinal accessory nerve (XI)

## Slide 19
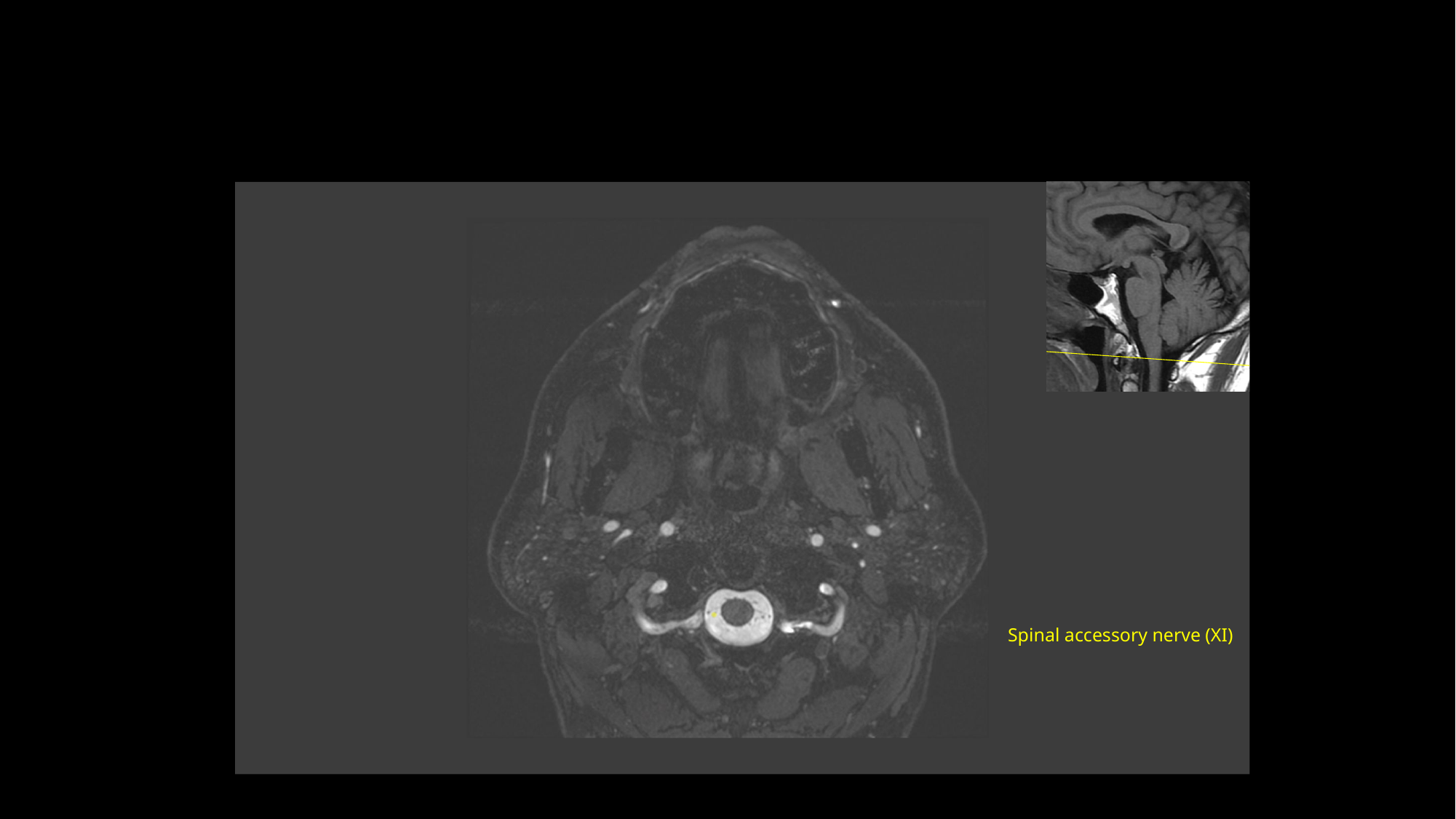

Spinal accessory nerve (XI)

## Slide 20
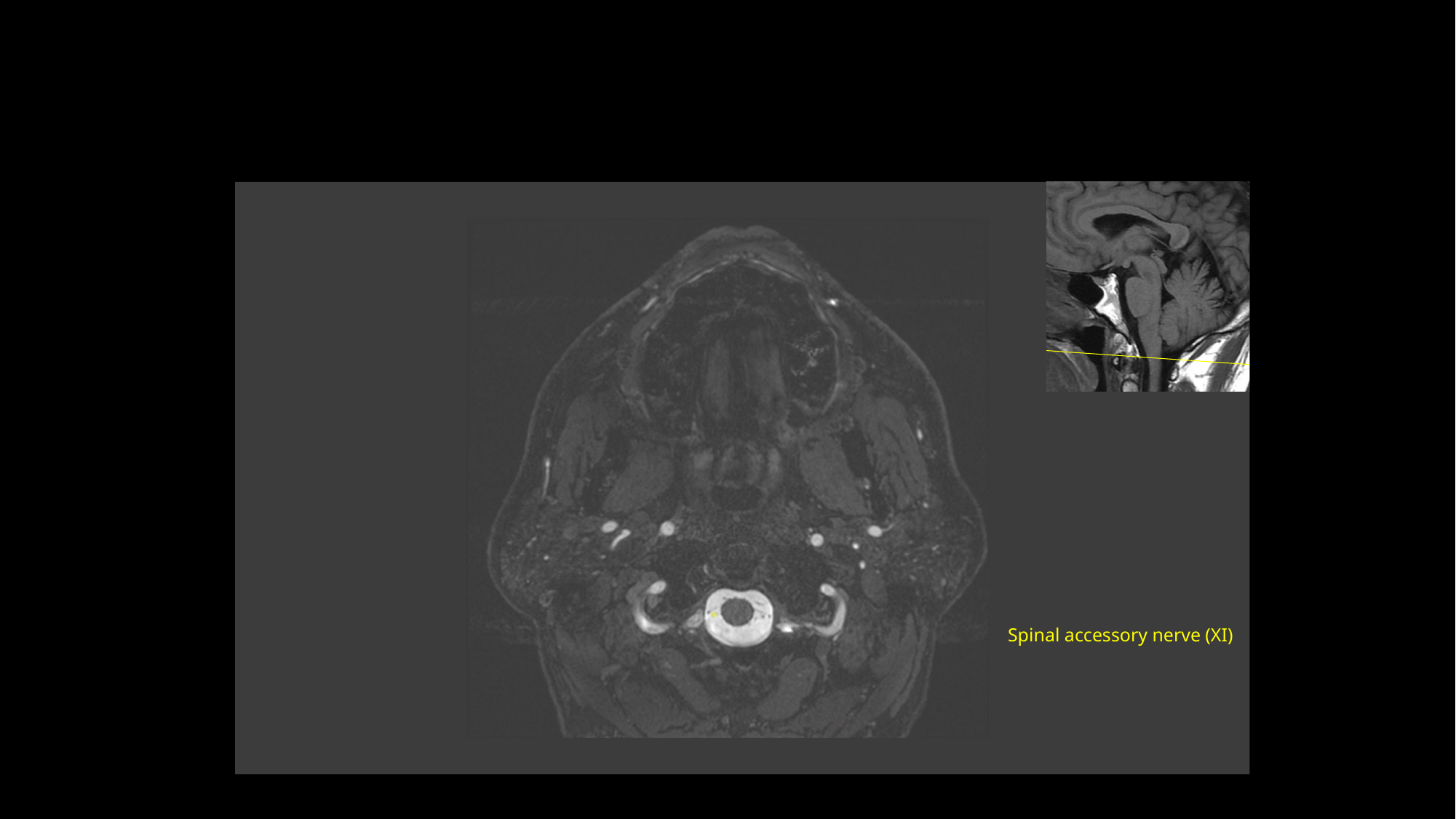

Spinal accessory nerve (XI)

## Slide 21
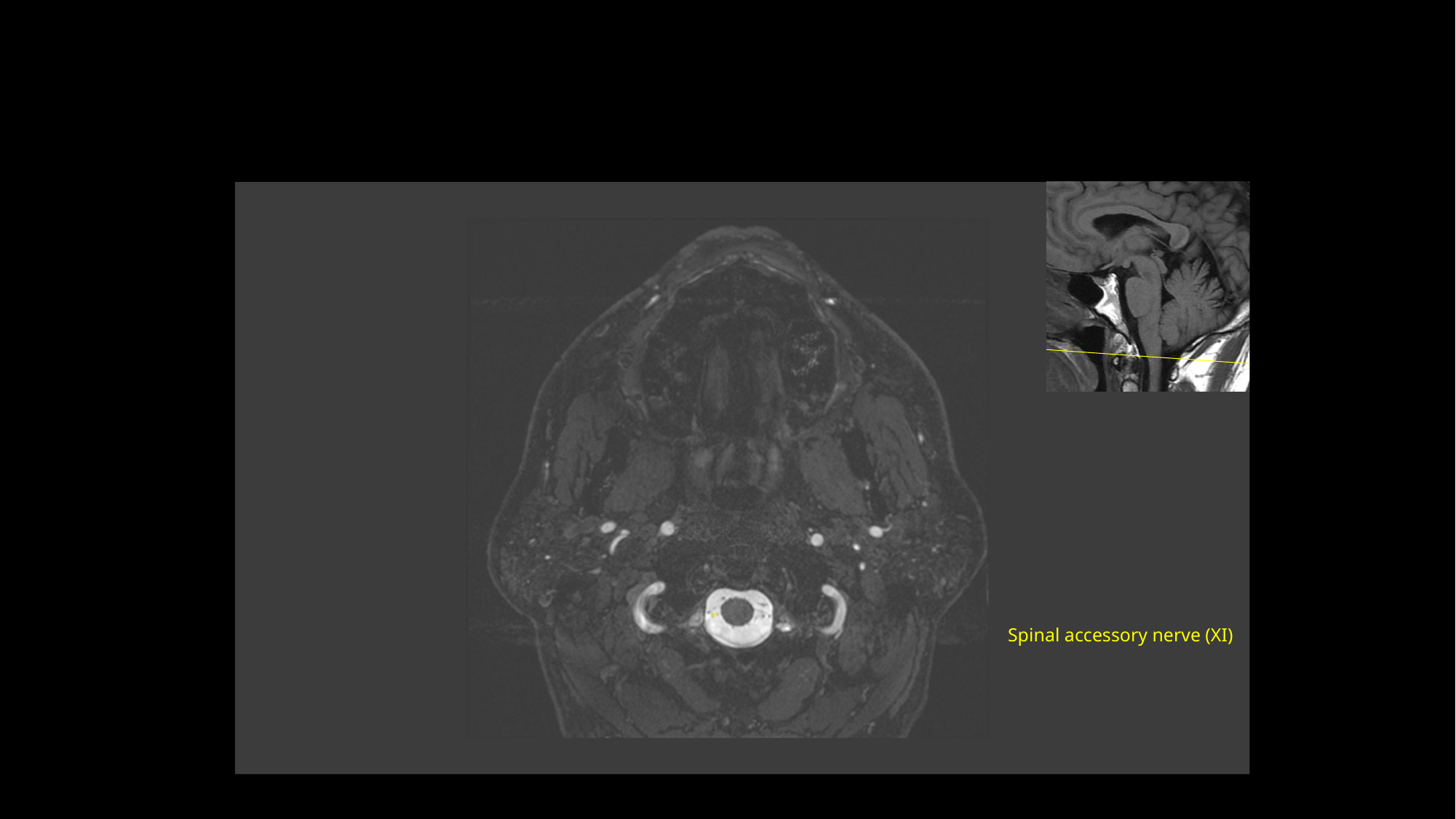

Spinal accessory nerve (XI)

## Slide 22
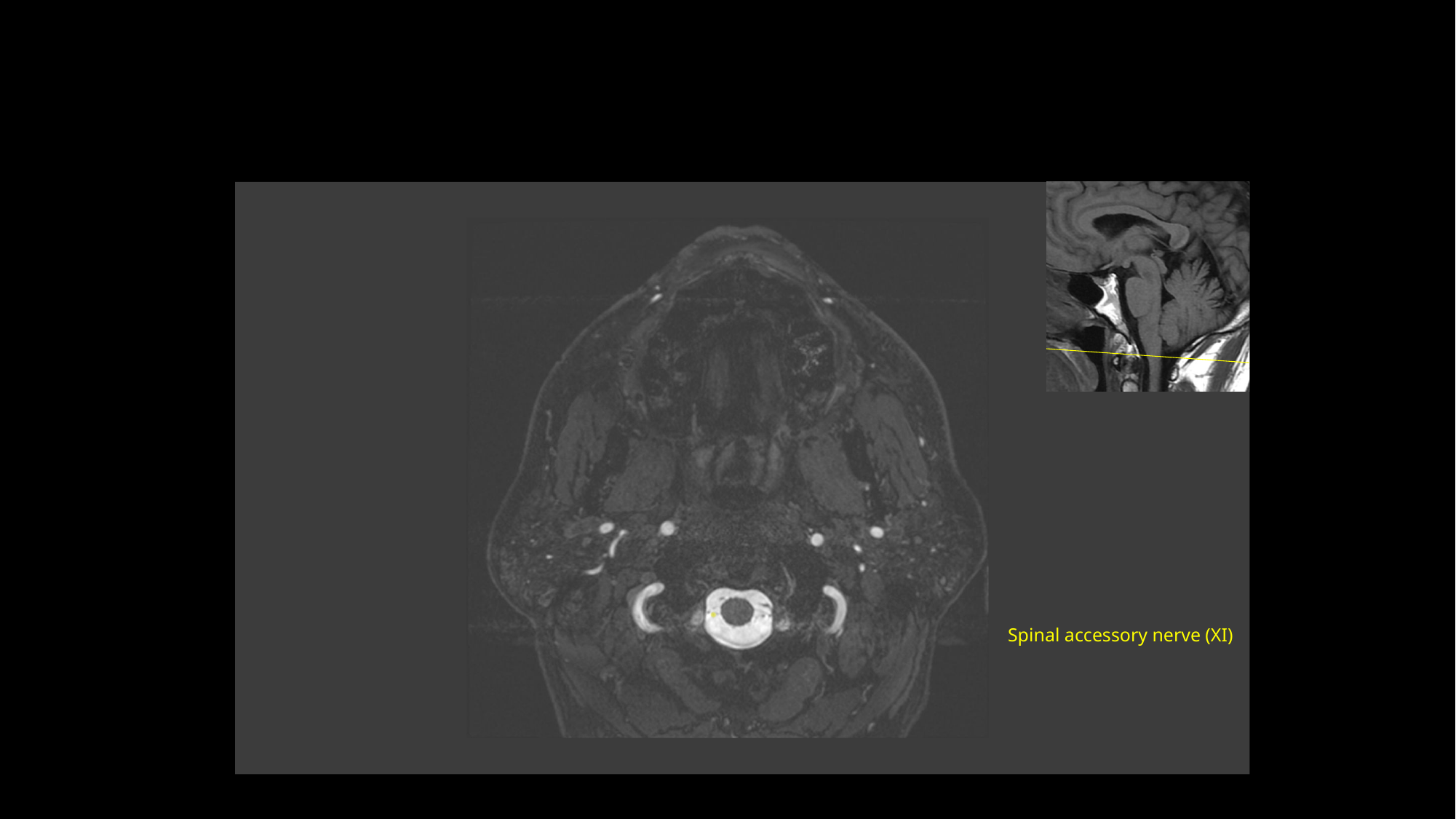

Spinal accessory nerve (XI)

## Slide 23
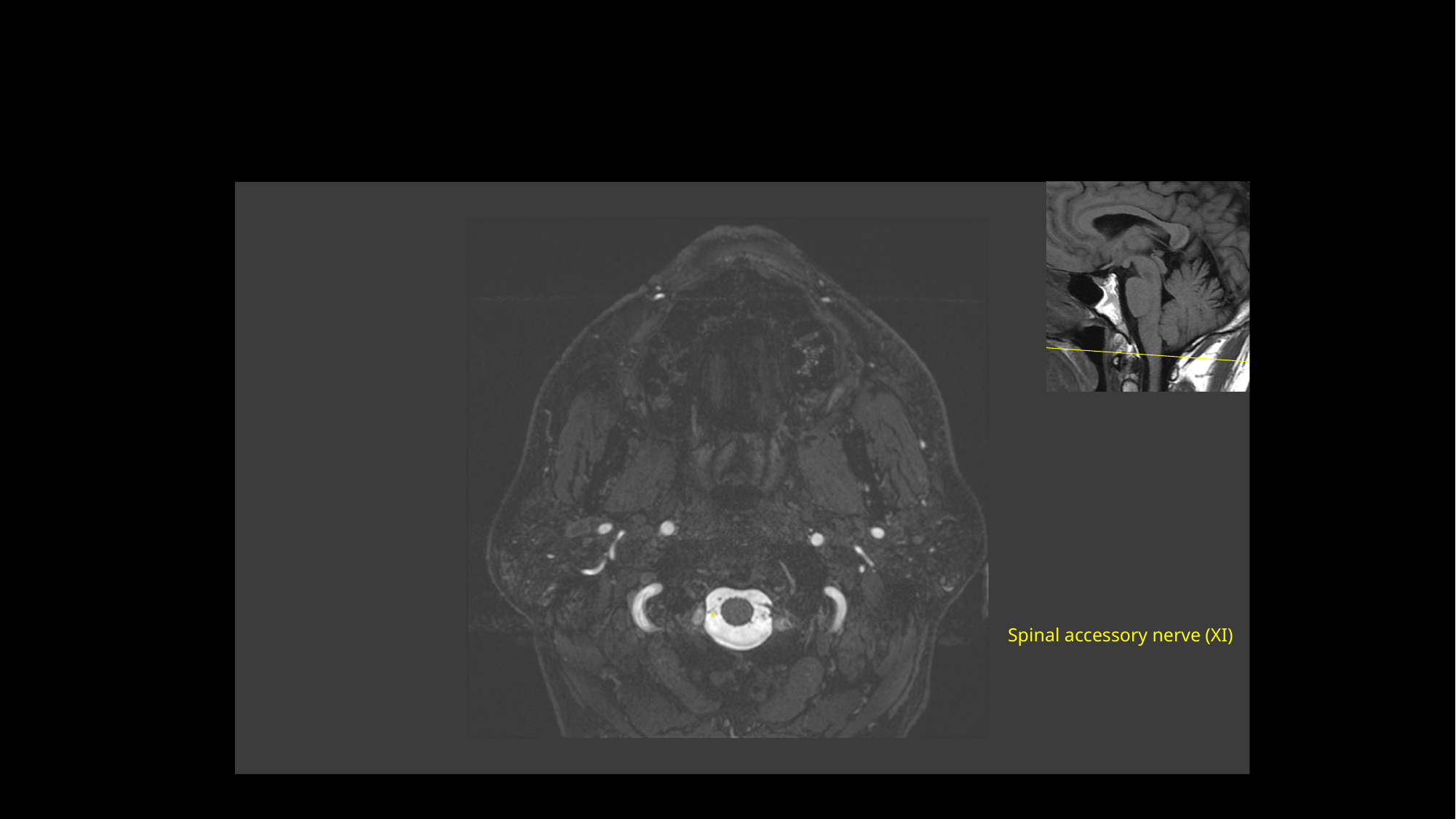

Spinal accessory nerve (XI)

## Slide 24
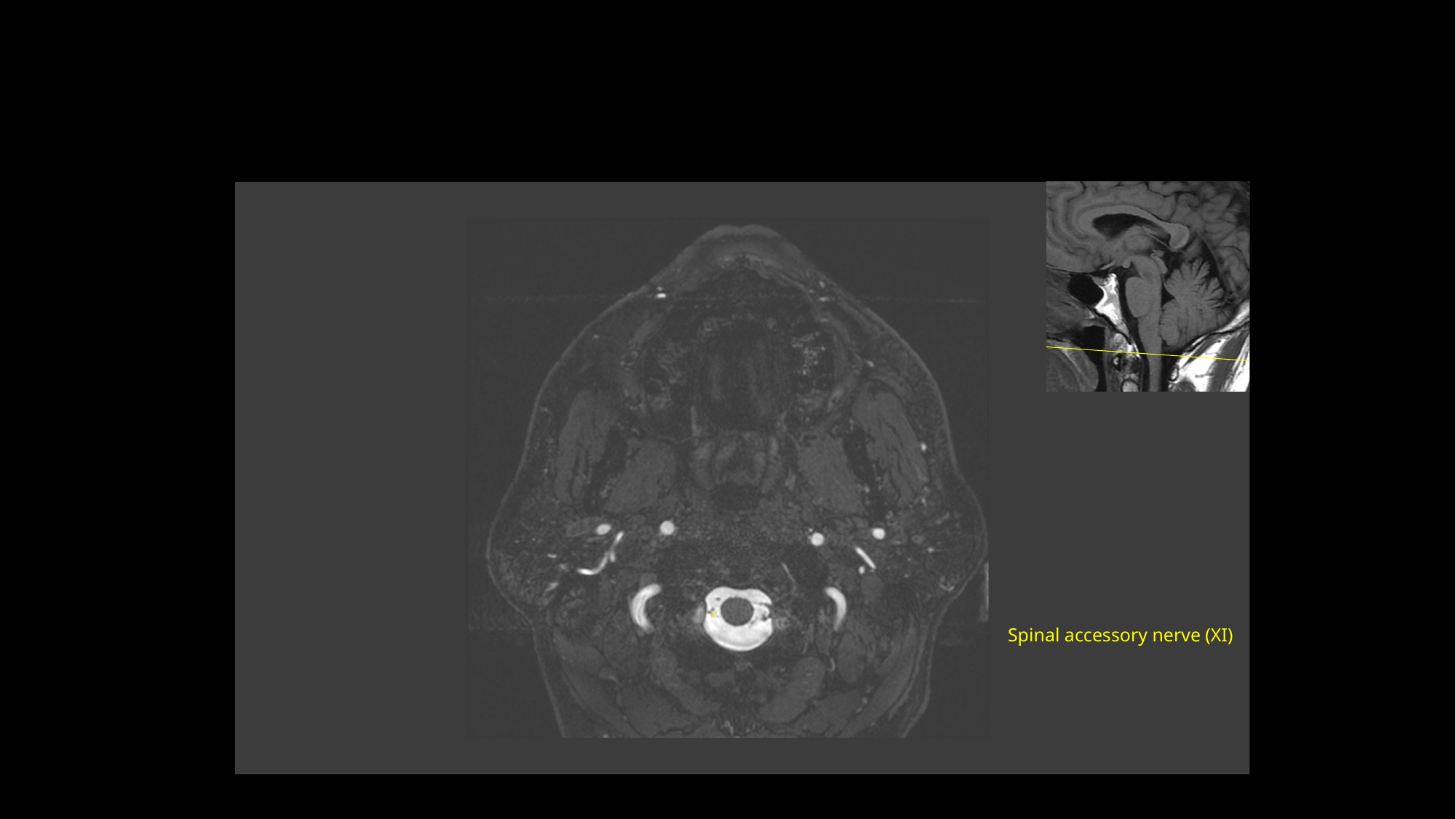

Spinal accessory nerve (XI)

## Slide 25
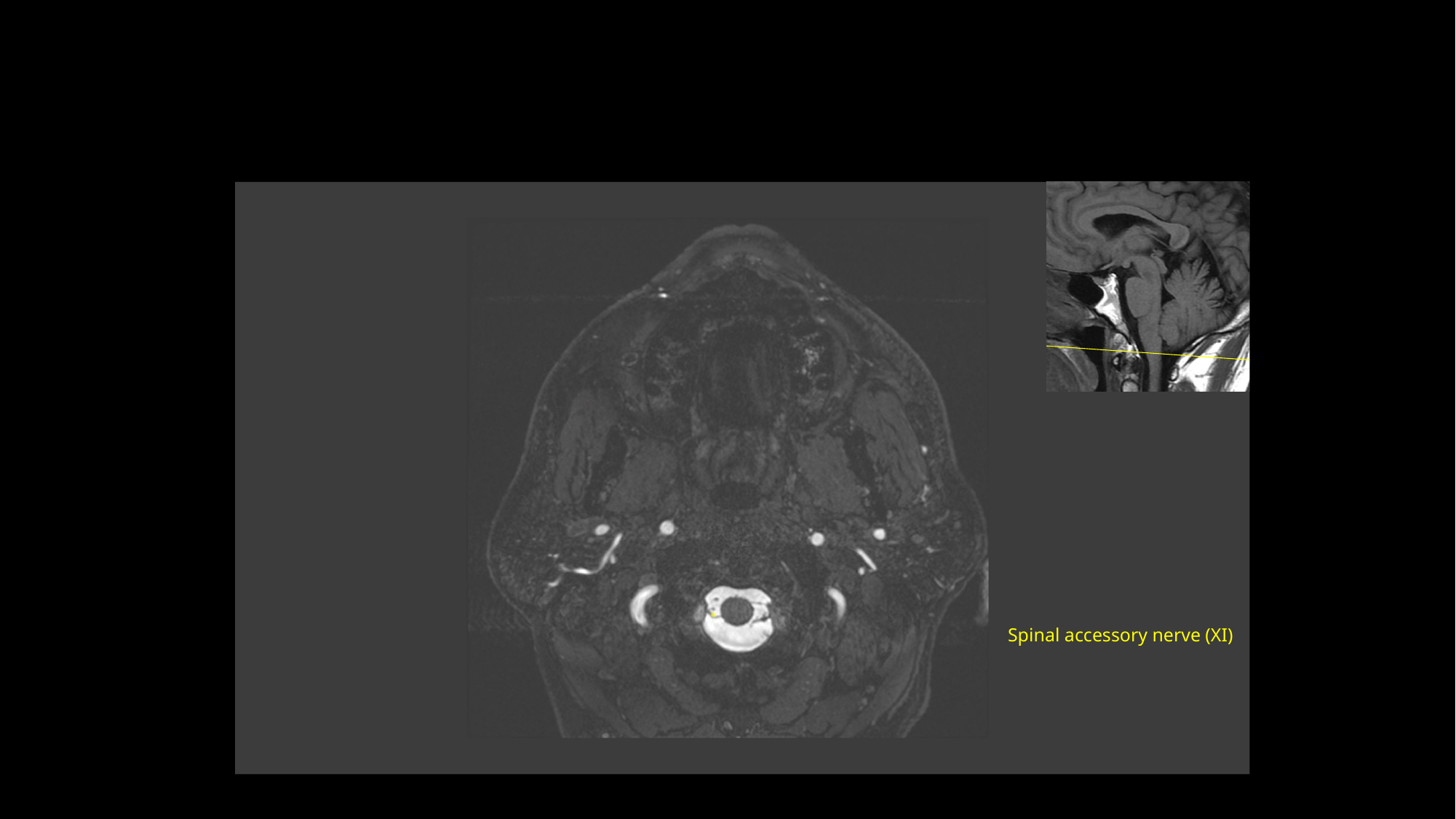

Spinal accessory nerve (XI)

## Slide 26
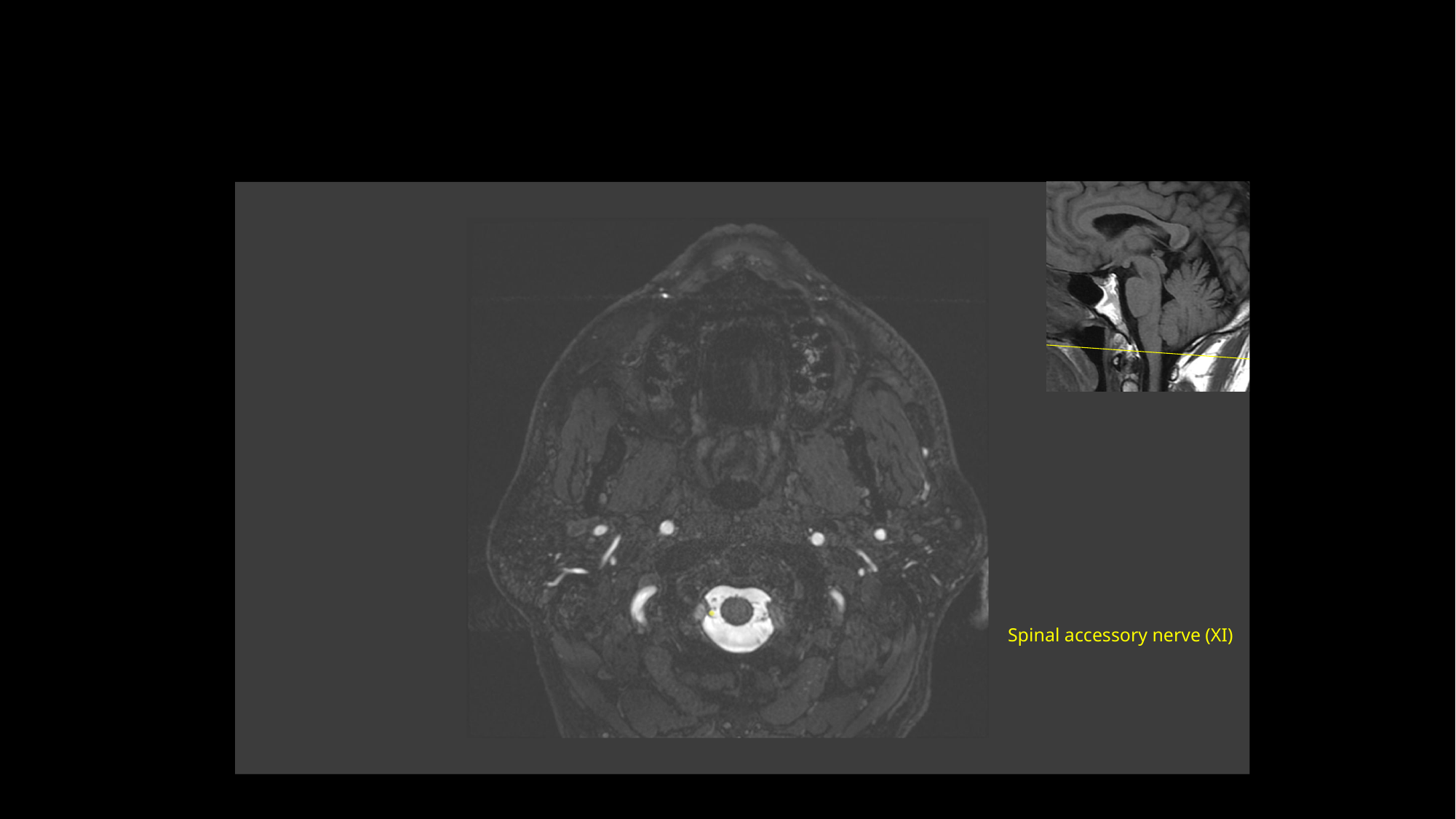

Spinal accessory nerve (XI)

## Slide 27
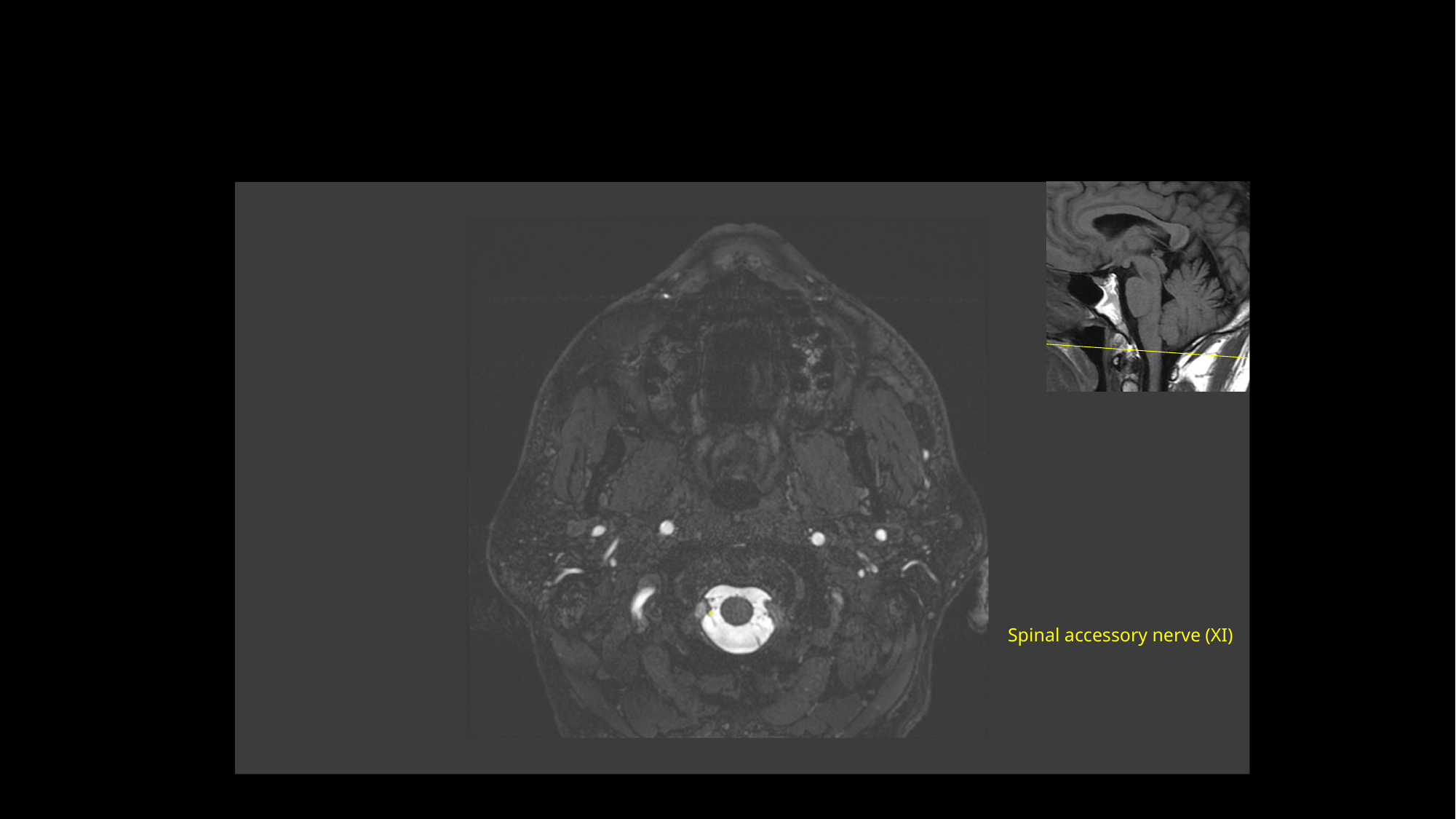

Spinal accessory nerve (XI)

## Slide 28
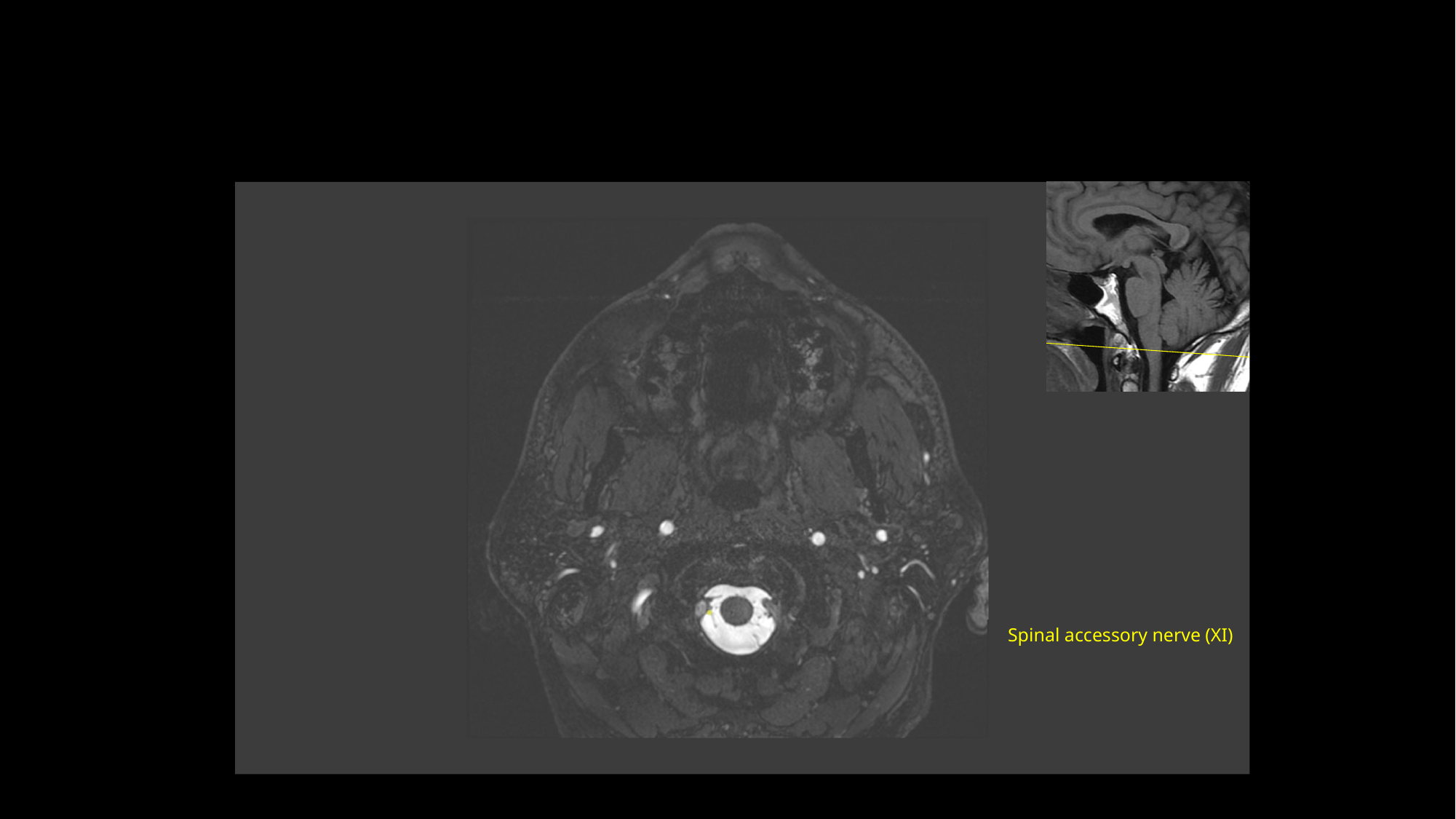

Spinal accessory nerve (XI)

## Slide 29
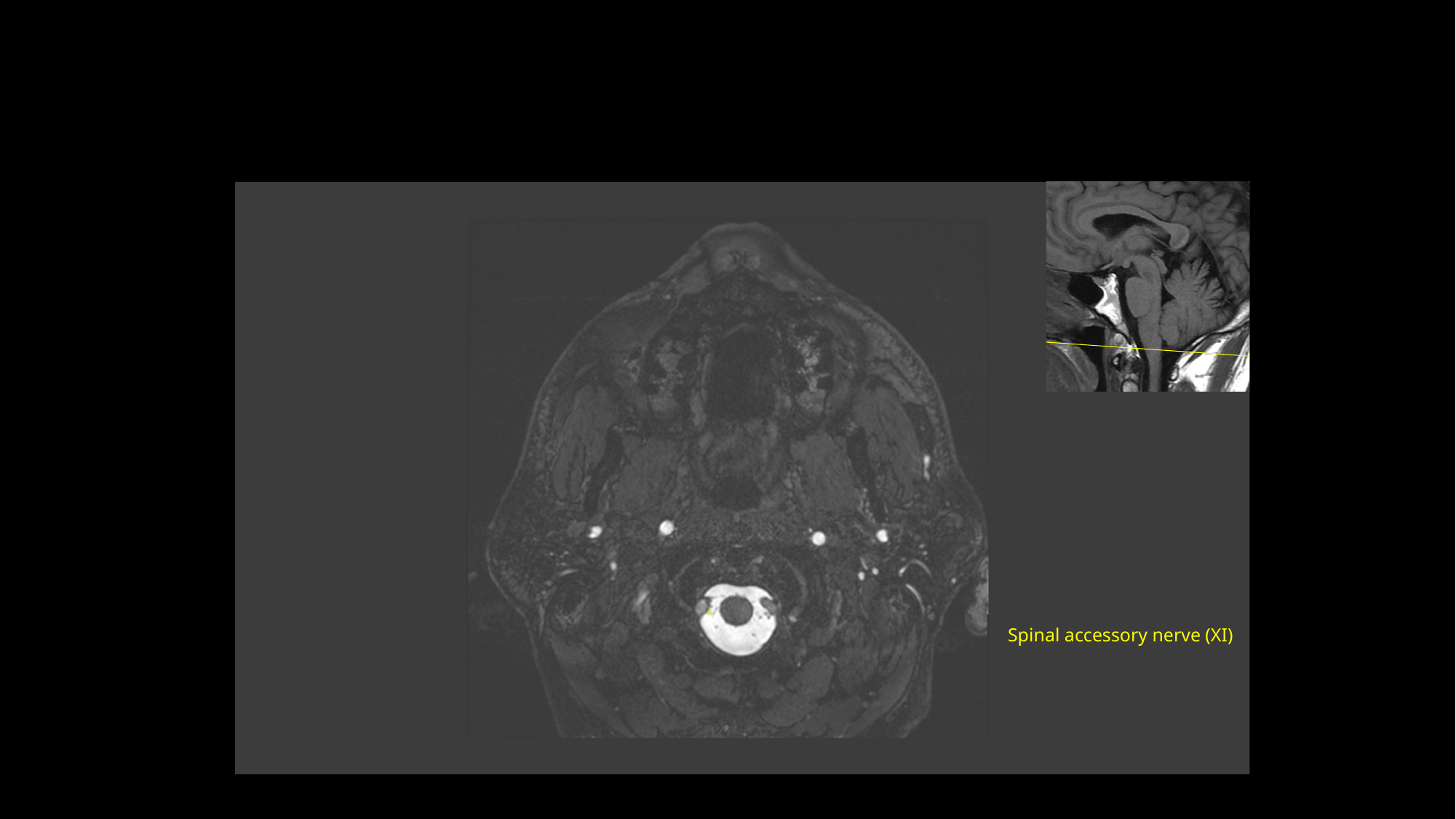

Spinal accessory nerve (XI)

## Slide 30
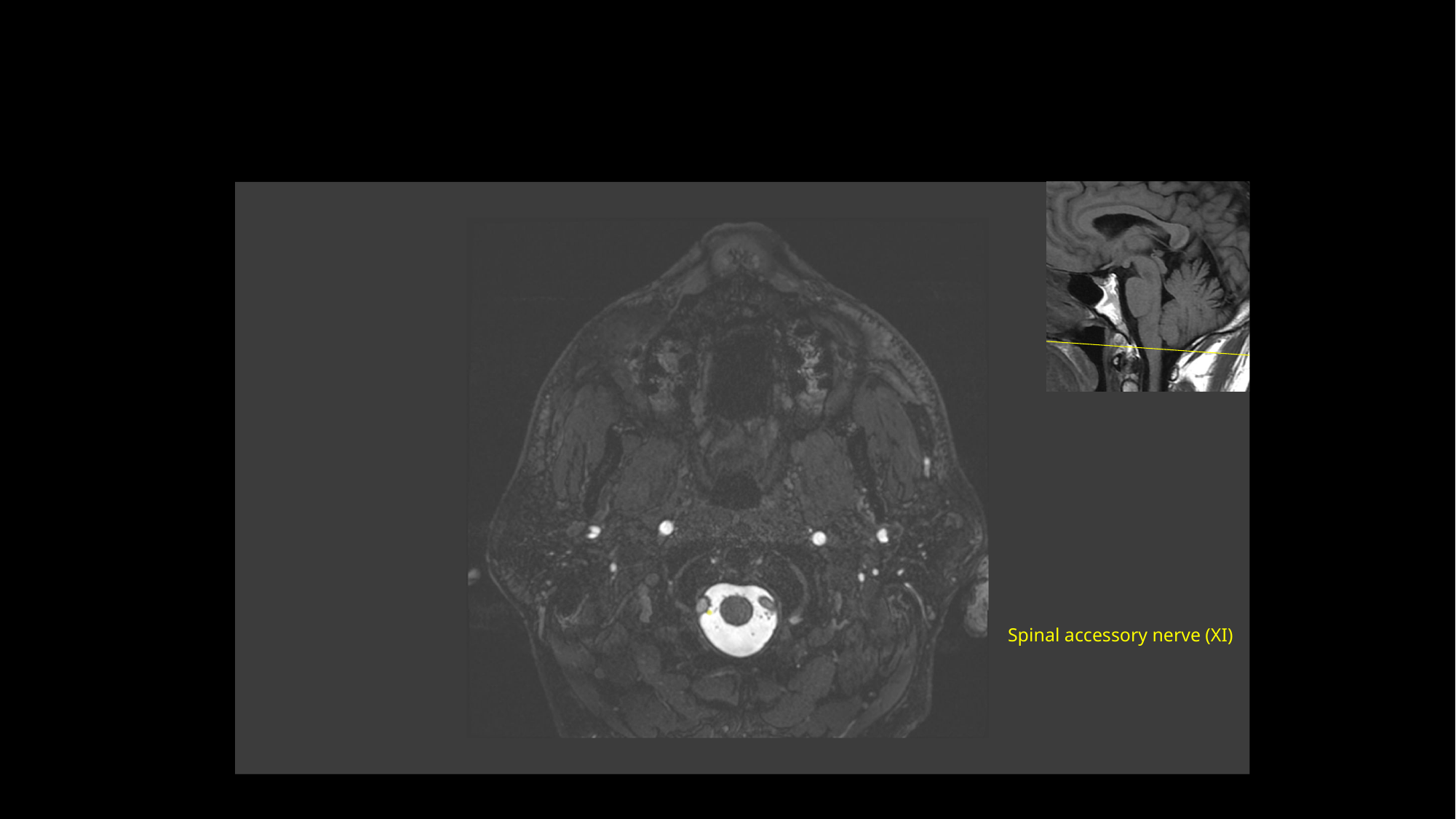

Spinal accessory nerve (XI)

## Slide 31
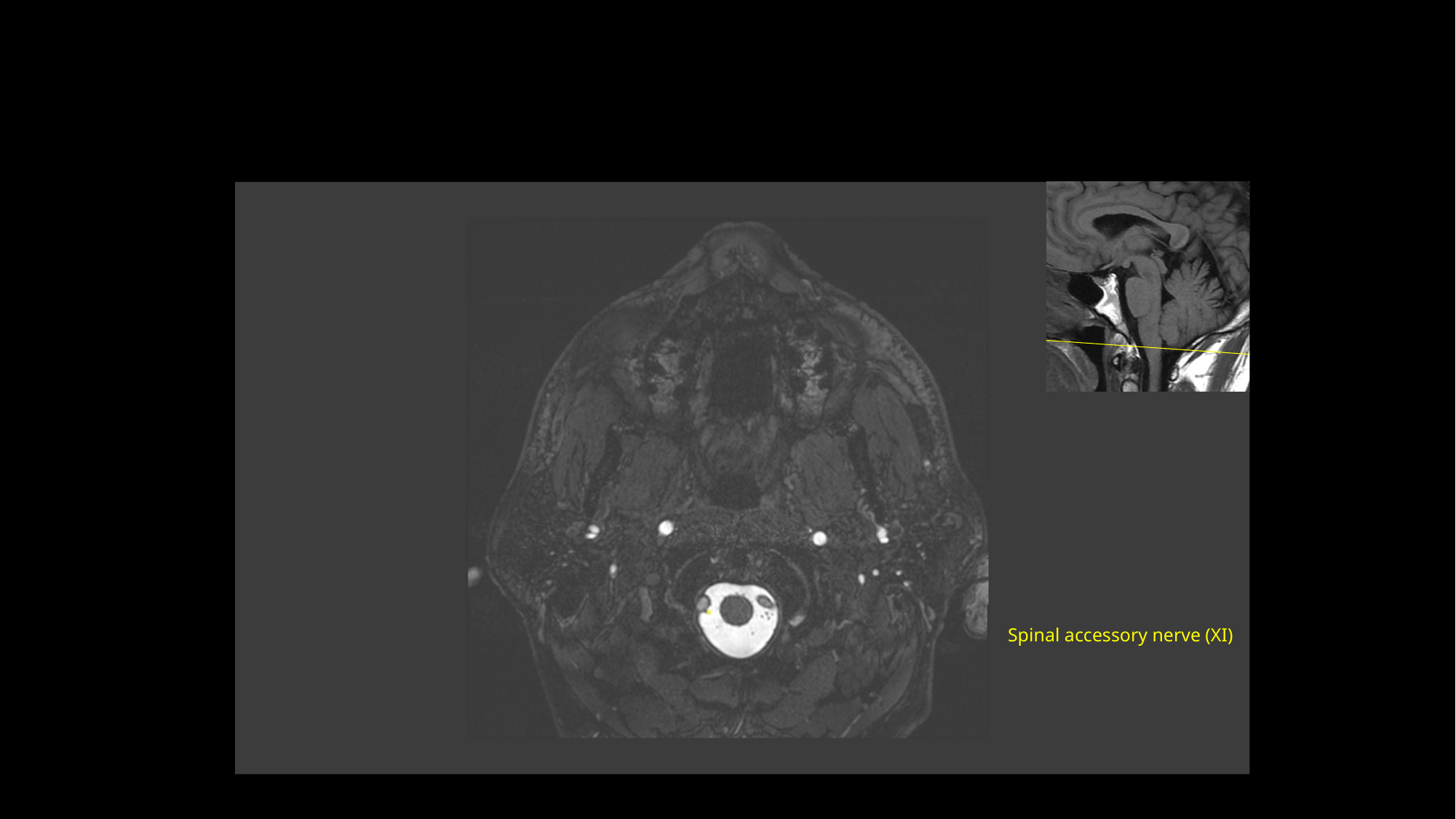

Spinal accessory nerve (XI)

## Slide 32
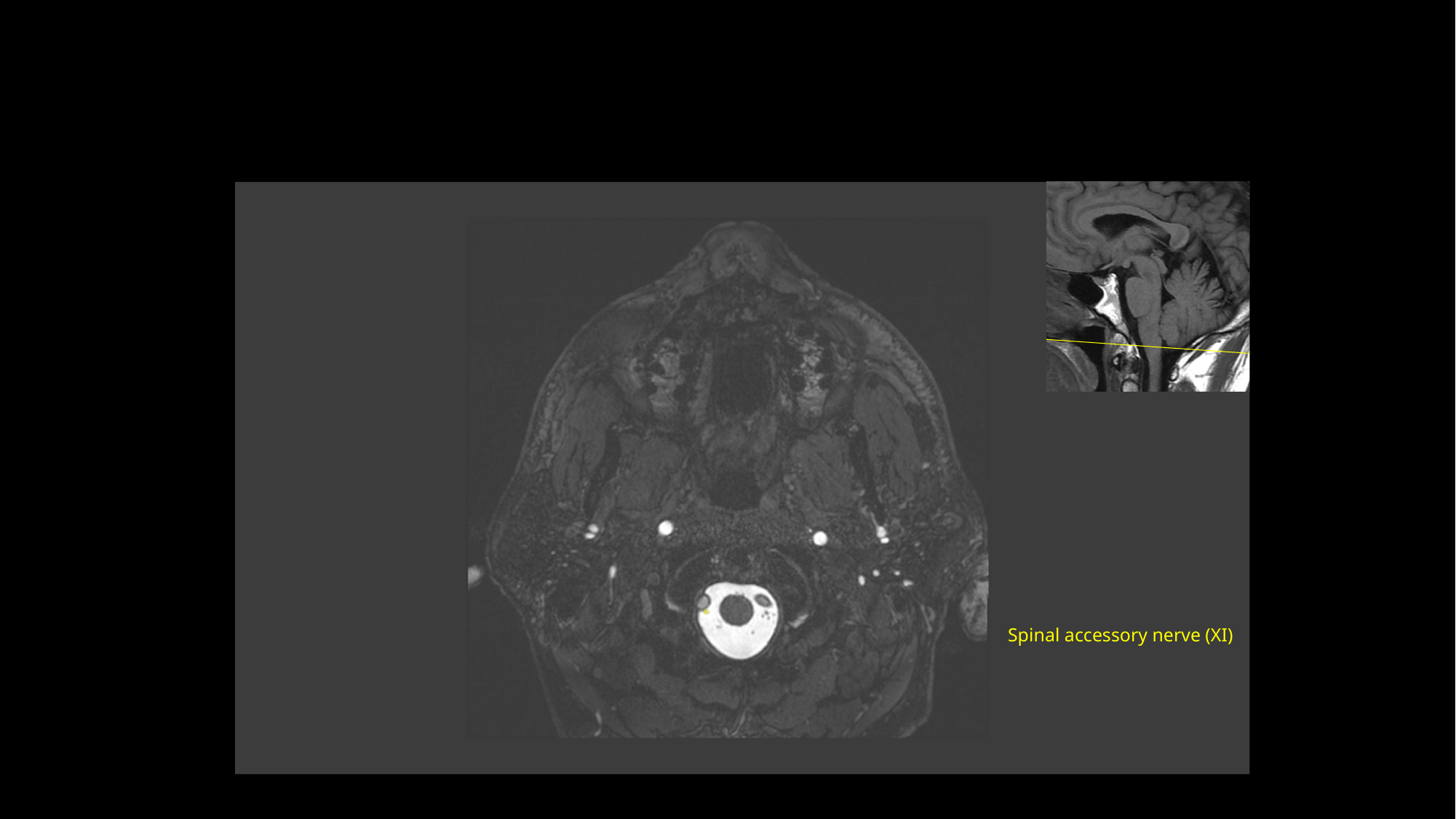

Spinal accessory nerve (XI)

## Slide 33
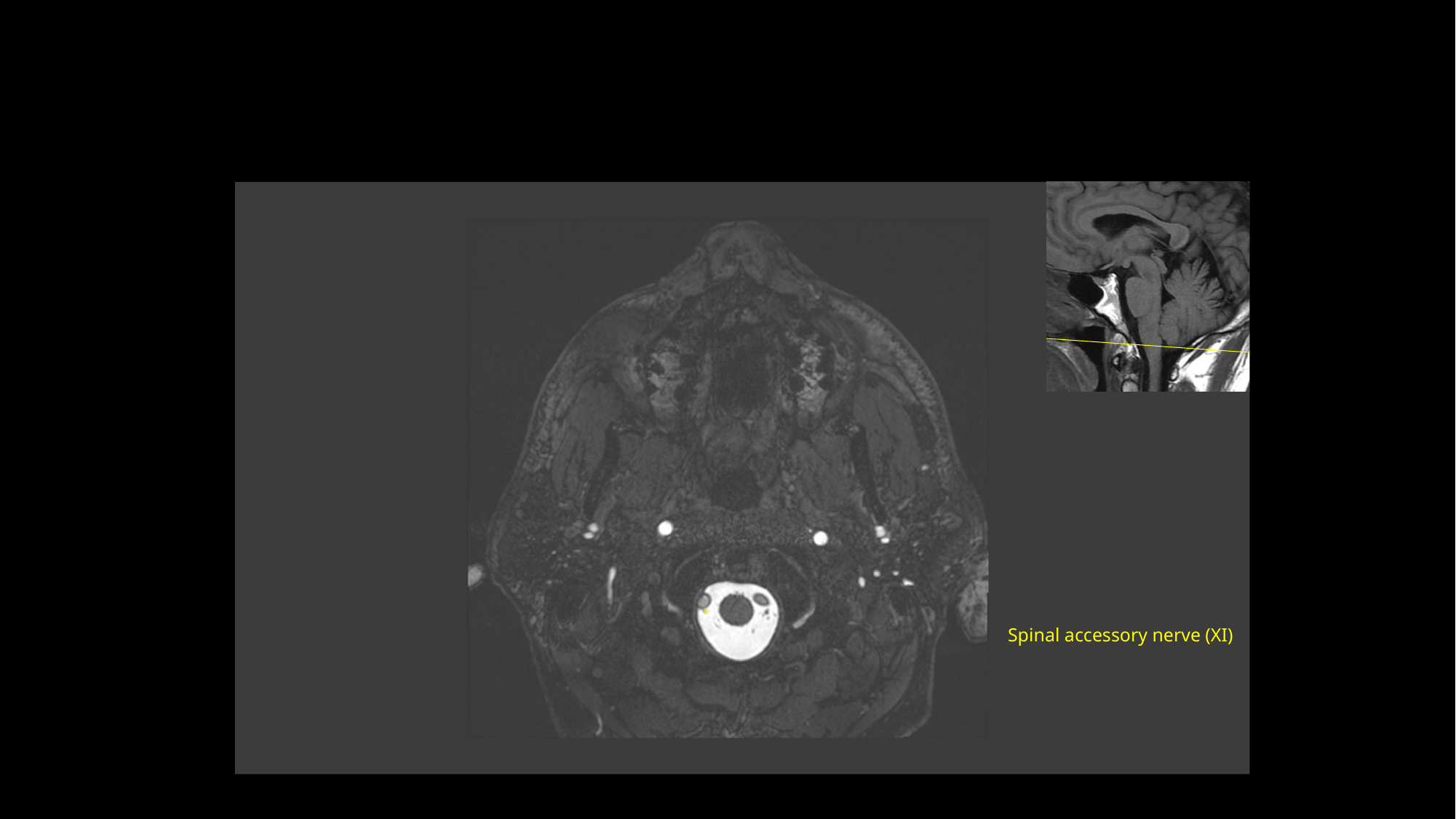

Spinal accessory nerve (XI)

## Slide 34
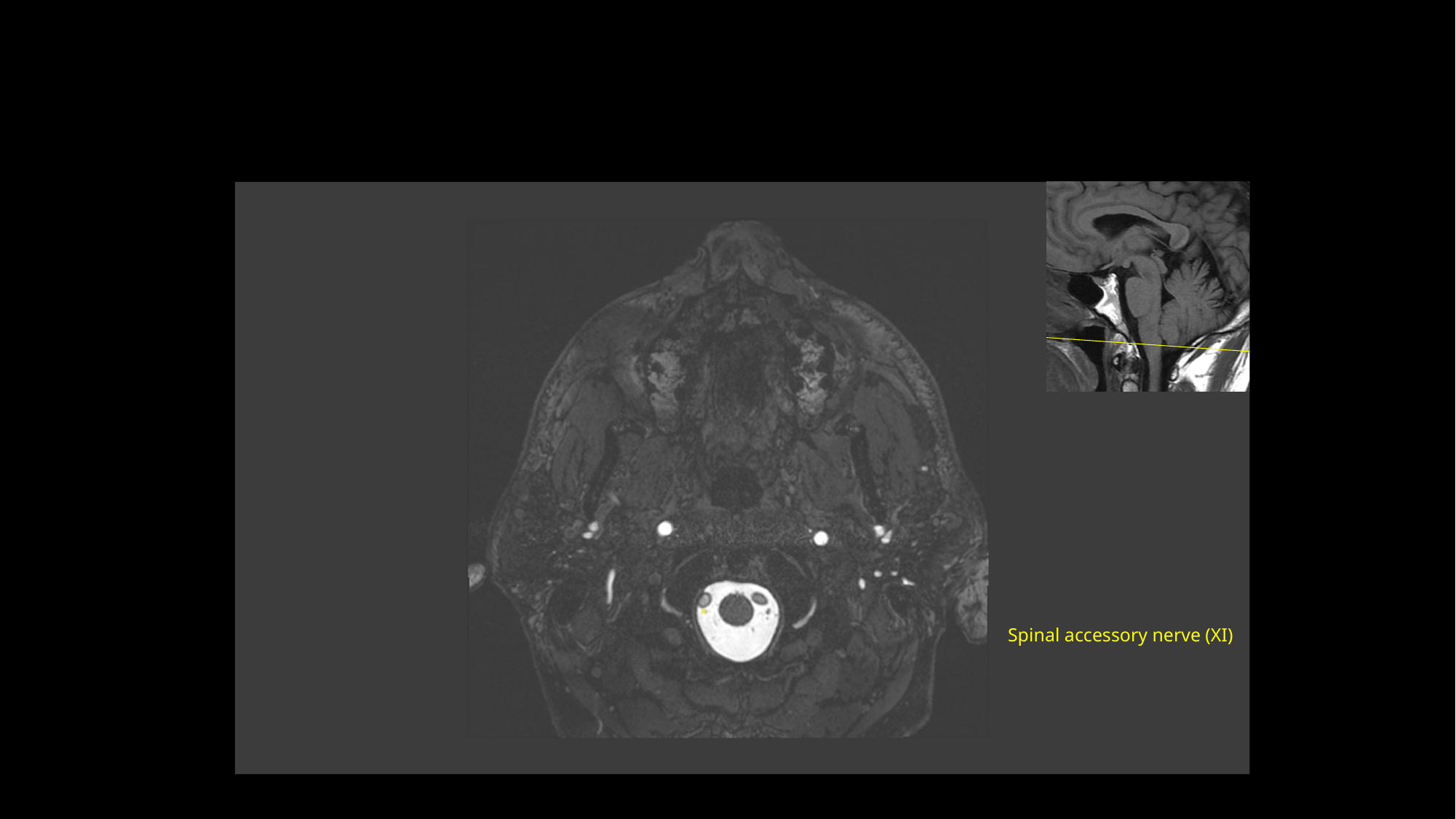

Spinal accessory nerve (XI)

## Slide 35
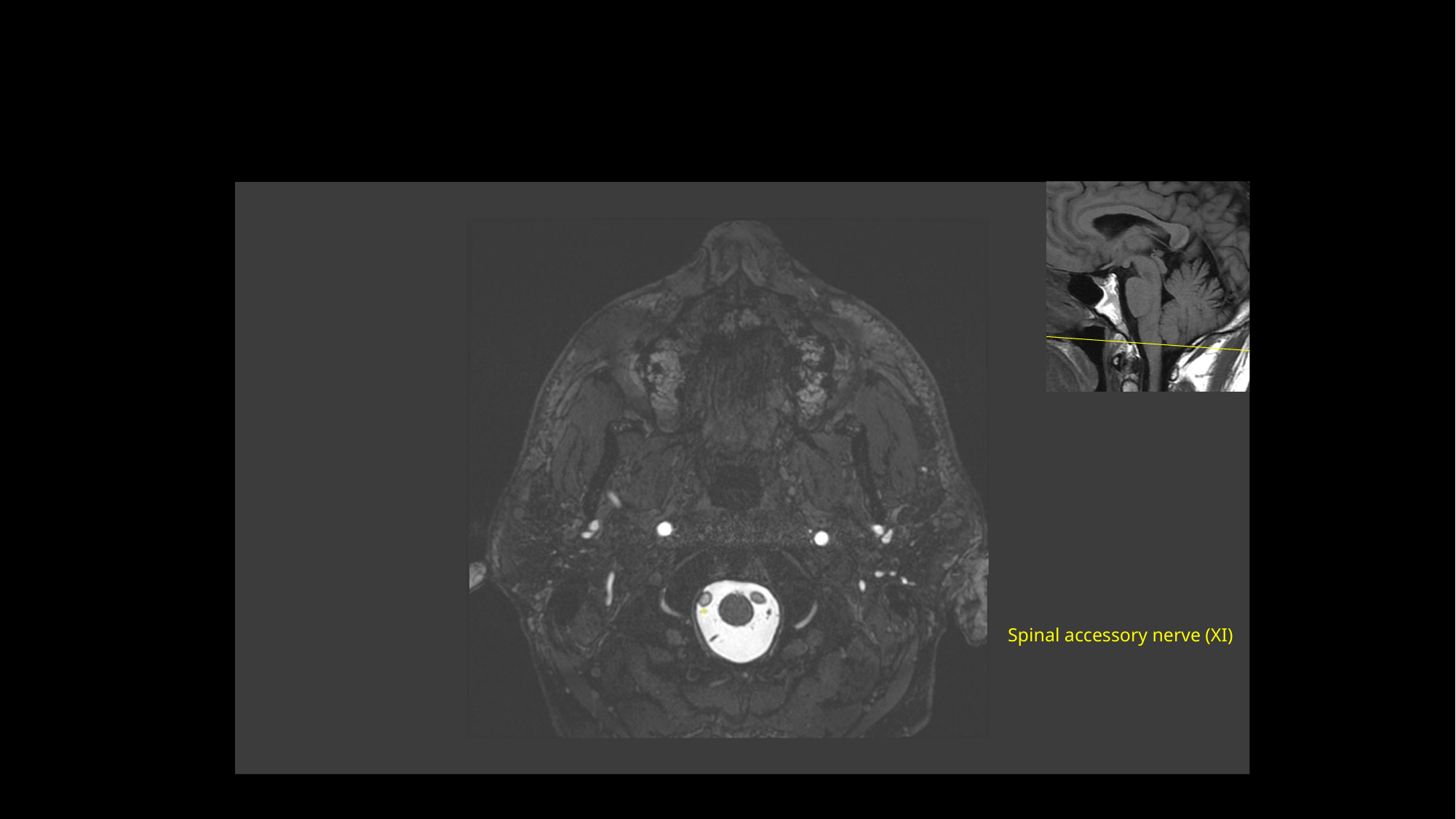

Spinal accessory nerve (XI)

## Slide 36
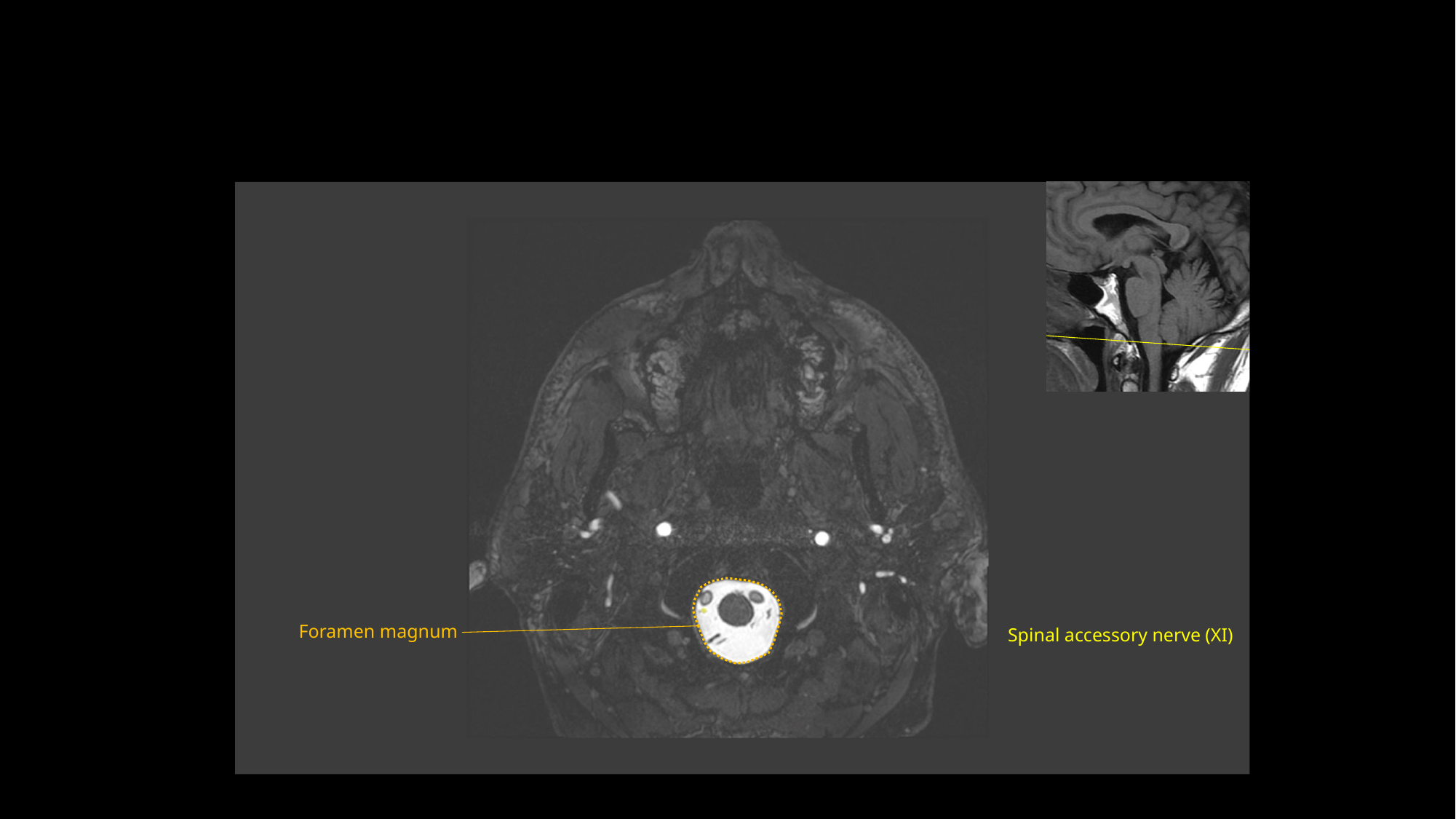

Foramen magnum
Spinal accessory nerve (XI)

## Slide 37
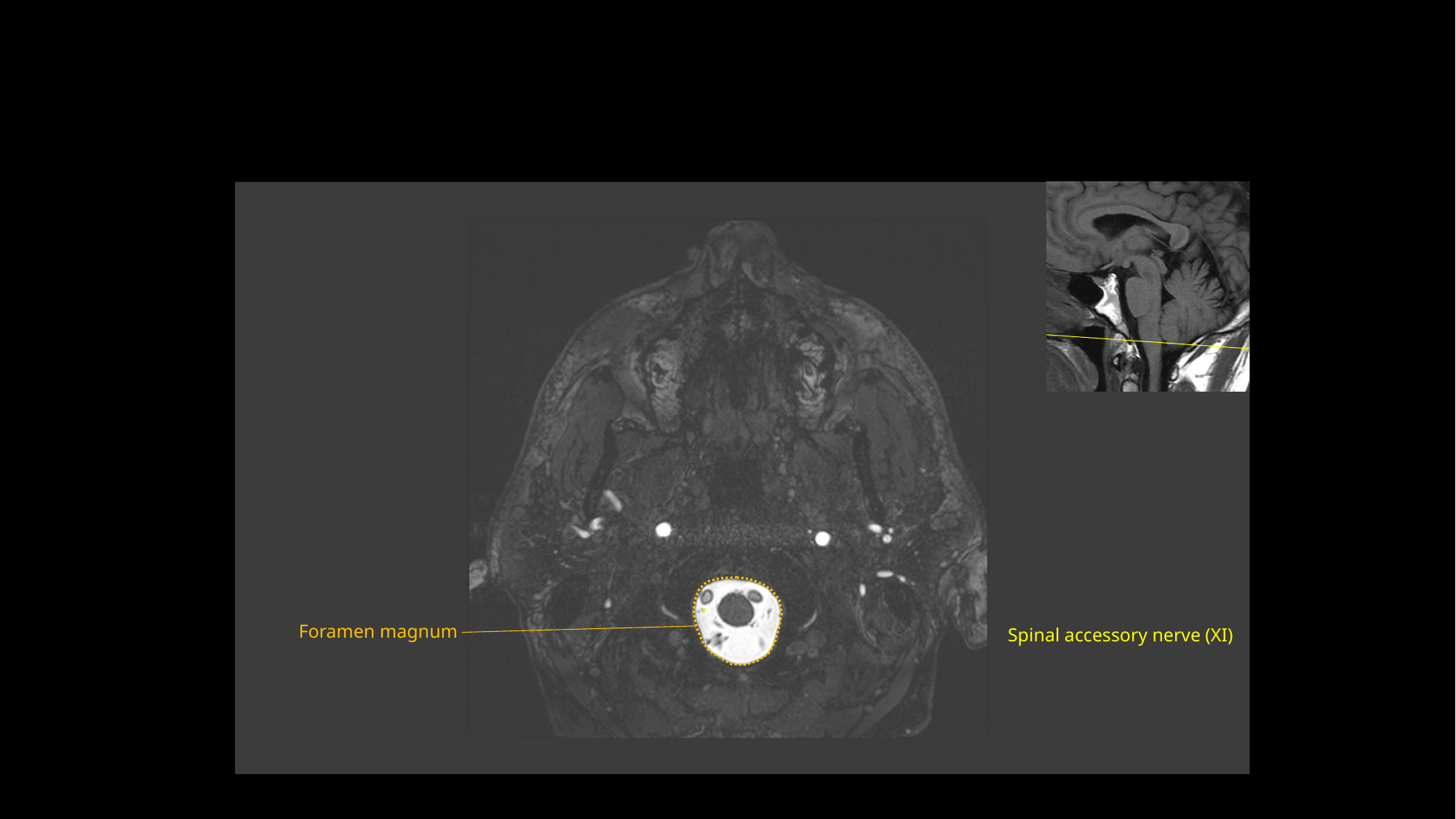

Foramen magnum
Spinal accessory nerve (XI)

## Slide 38
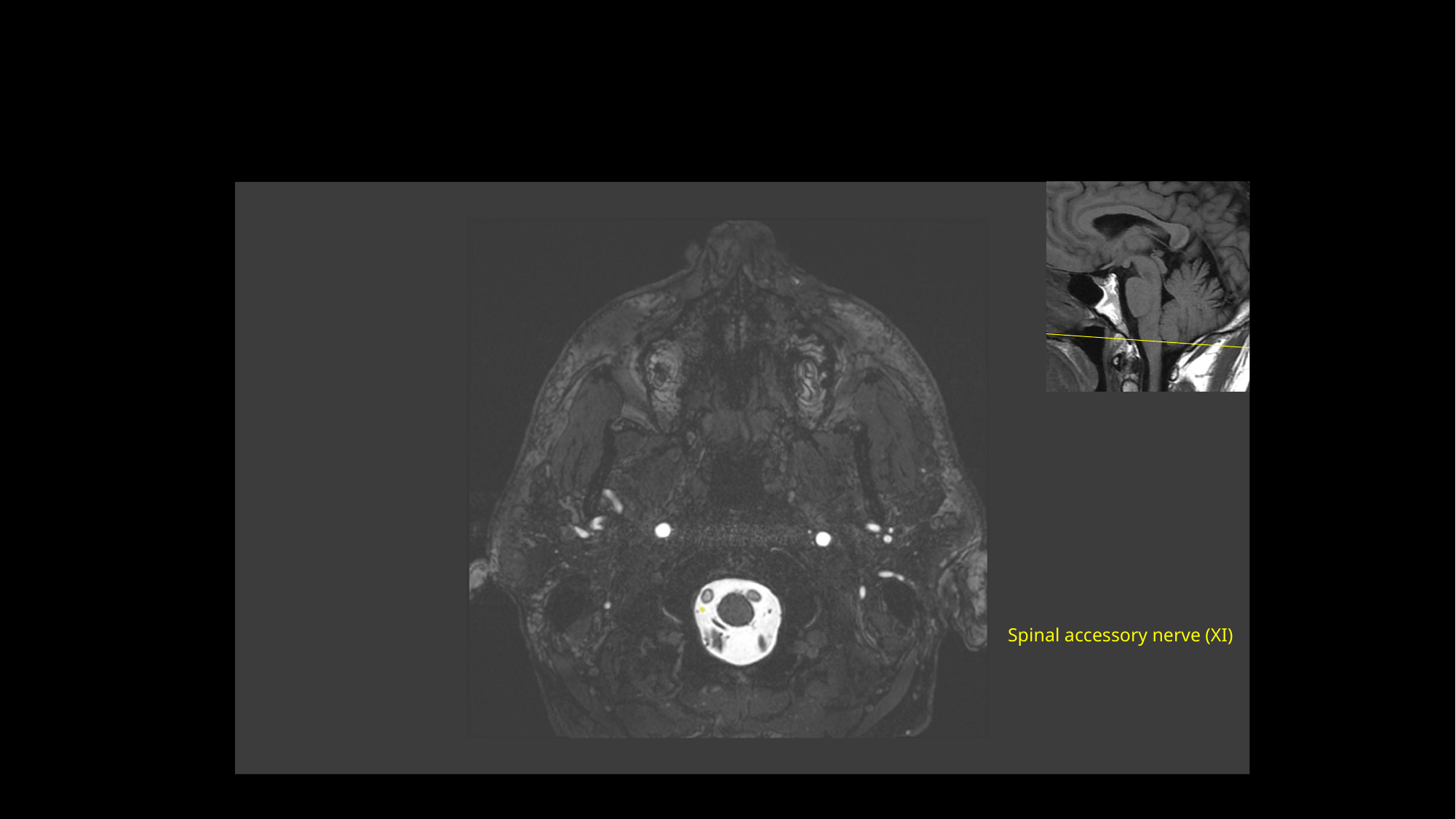

Spinal accessory nerve (XI)

## Slide 39
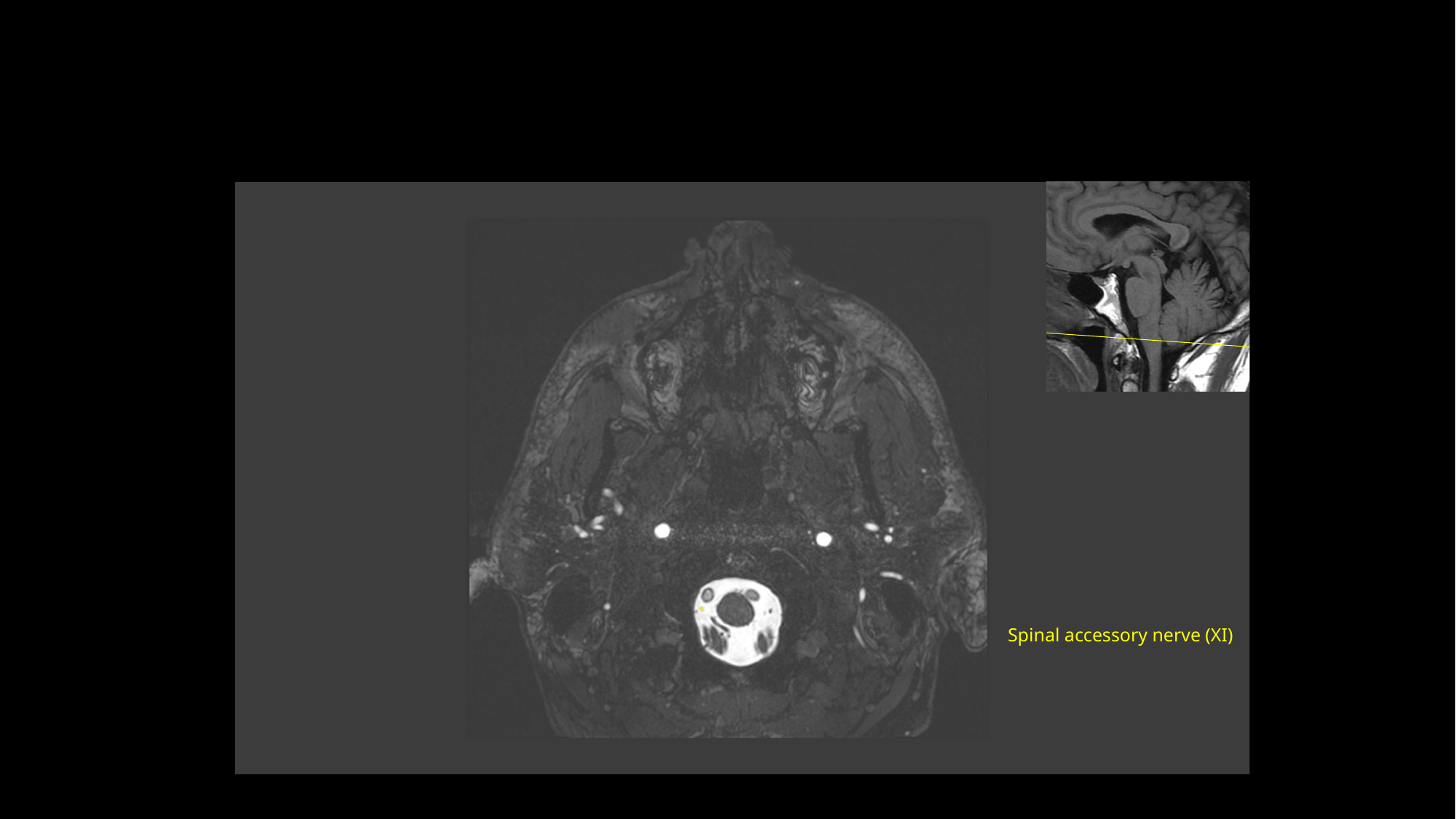

Spinal accessory nerve (XI)

## Slide 40
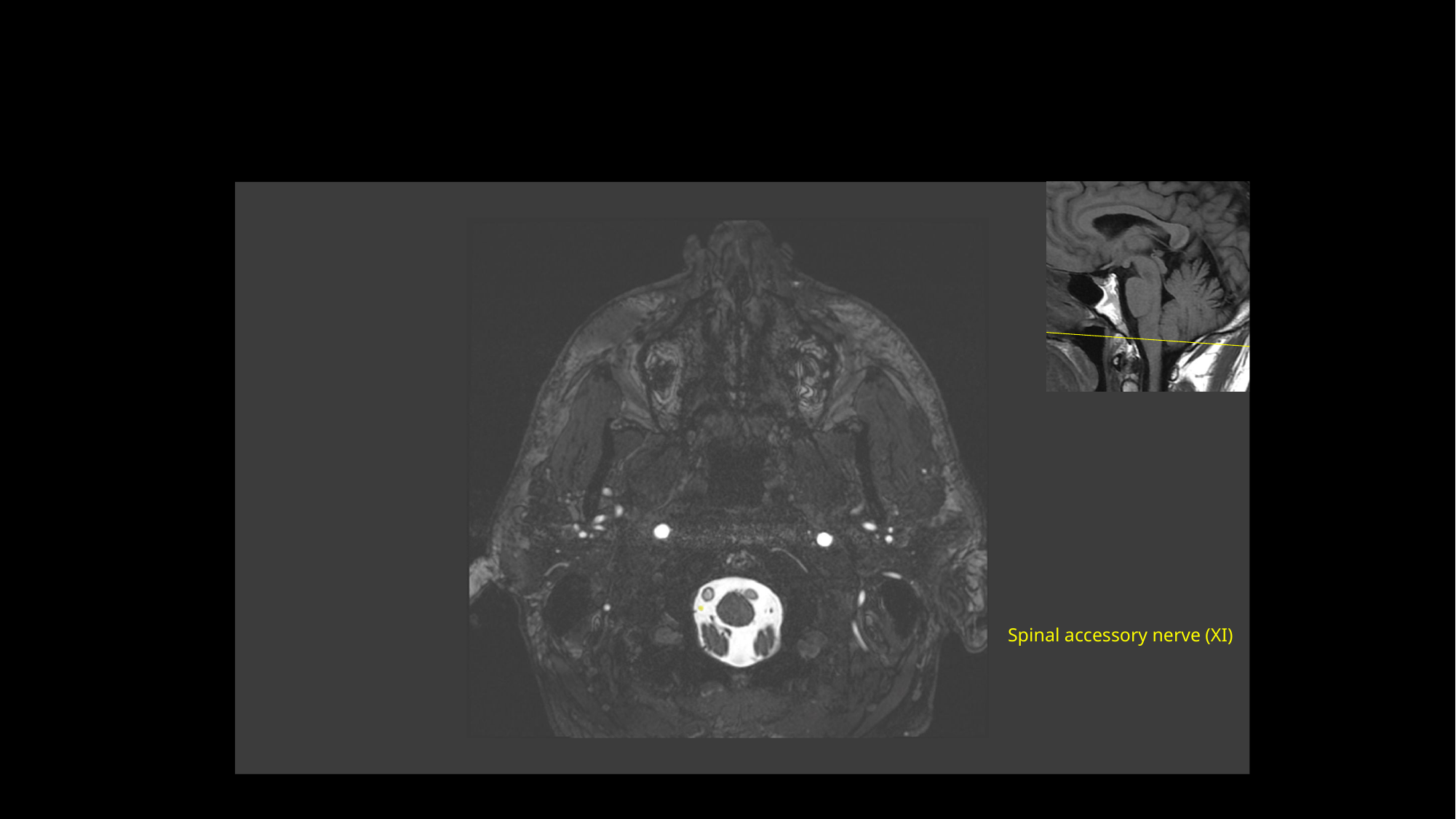

Spinal accessory nerve (XI)

## Slide 41
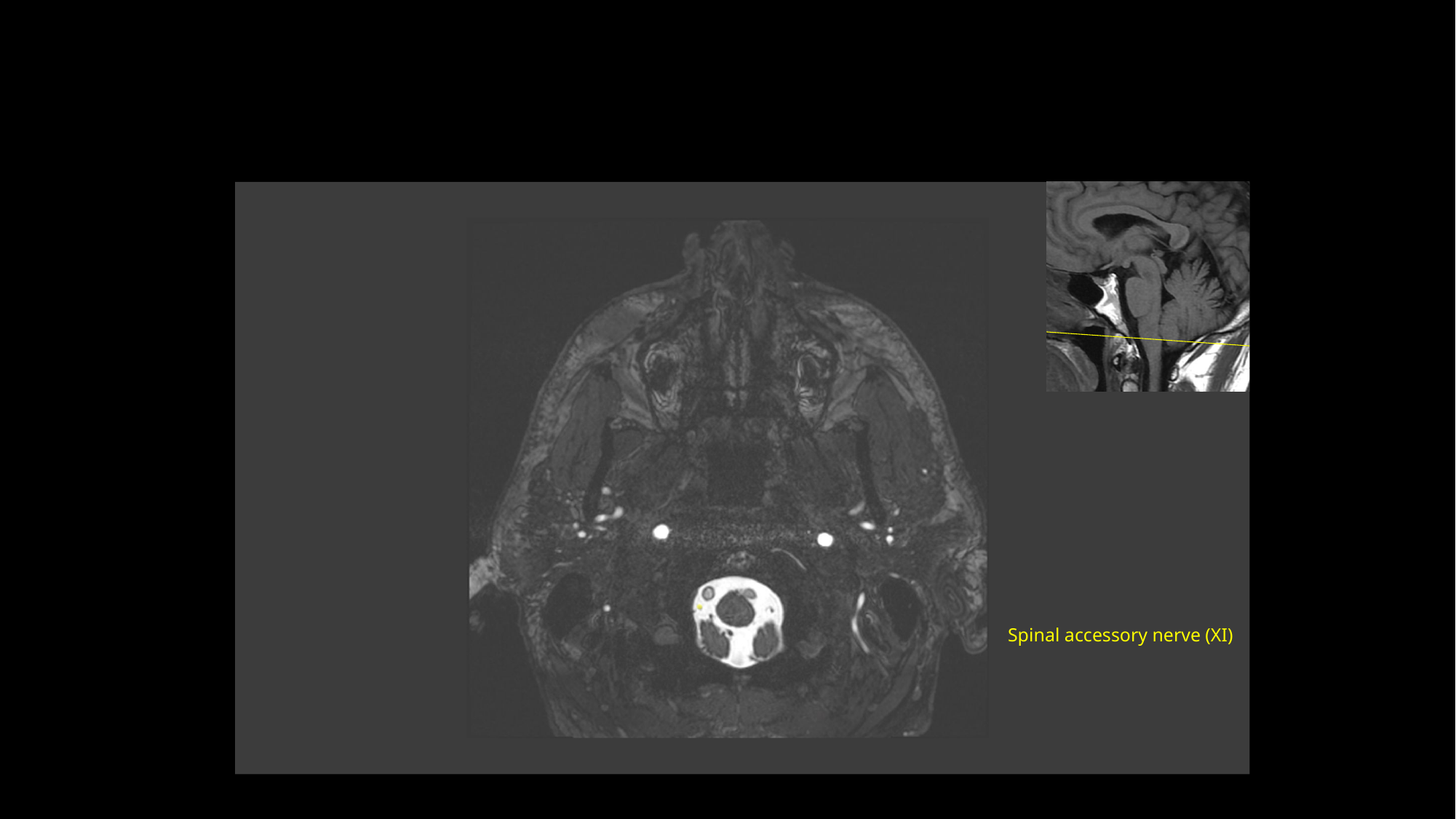

Spinal accessory nerve (XI)

## Slide 42
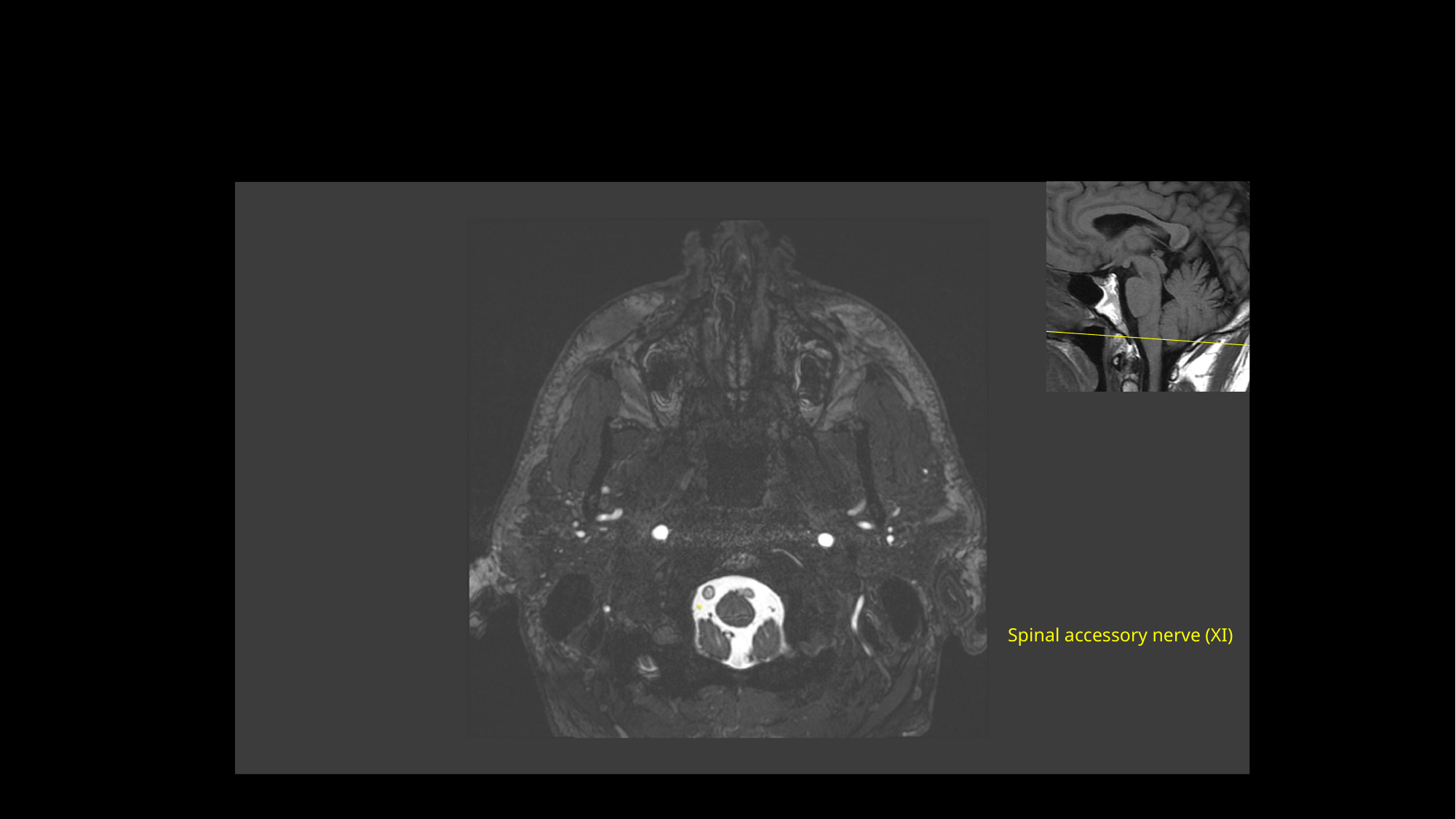

Spinal accessory nerve (XI)

## Slide 43
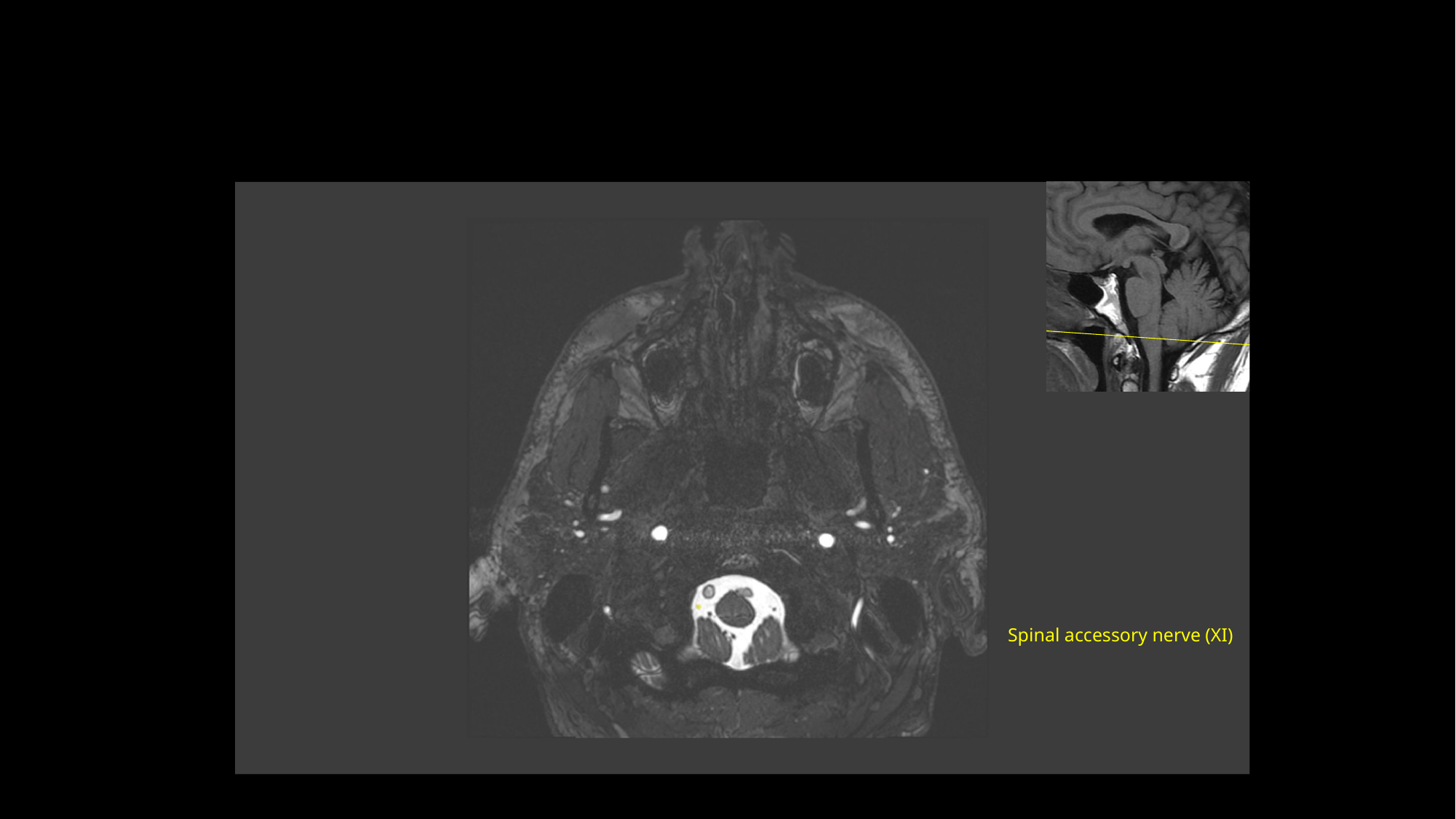

Spinal accessory nerve (XI)

## Slide 44
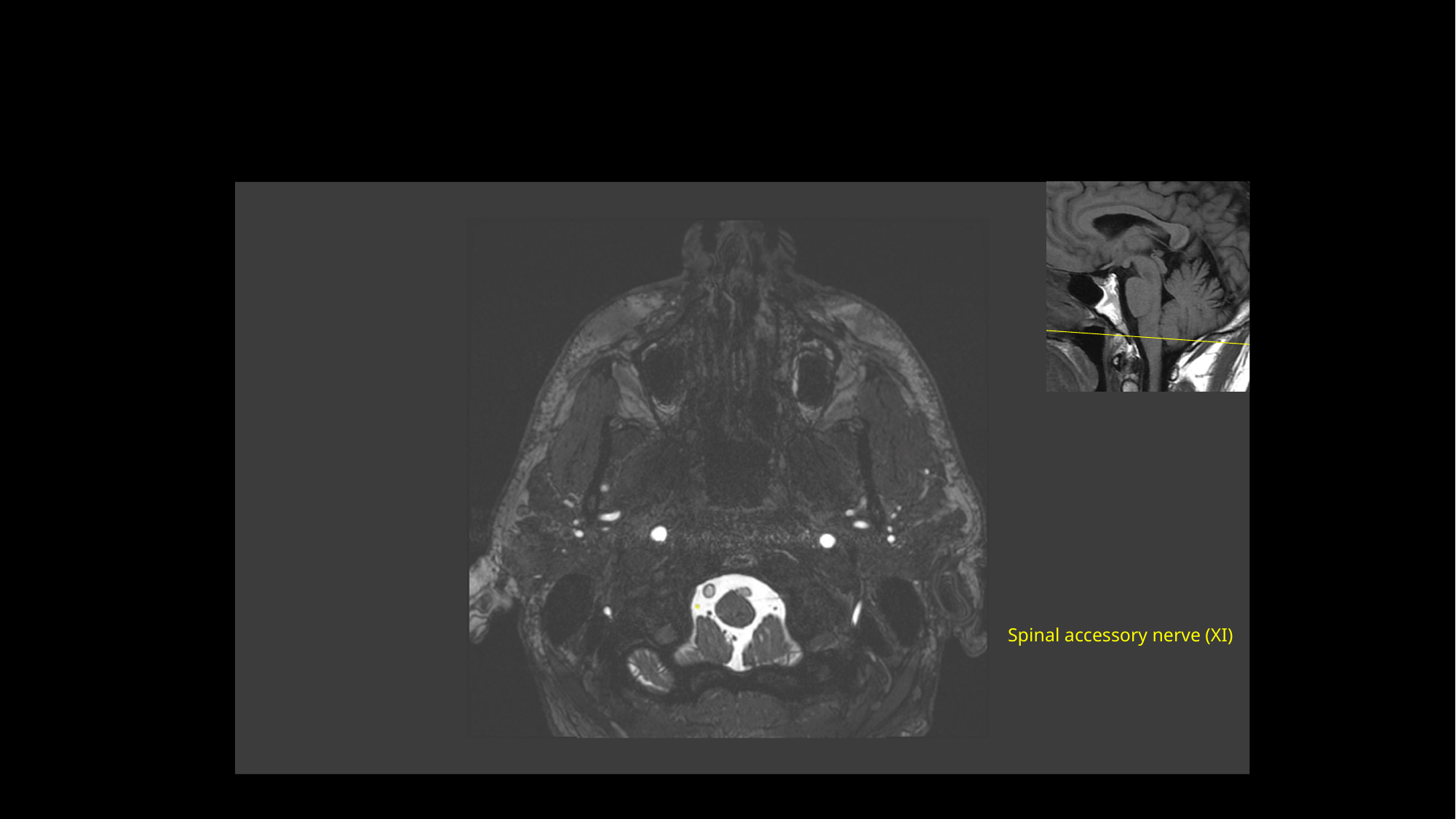

Spinal accessory nerve (XI)

## Slide 45
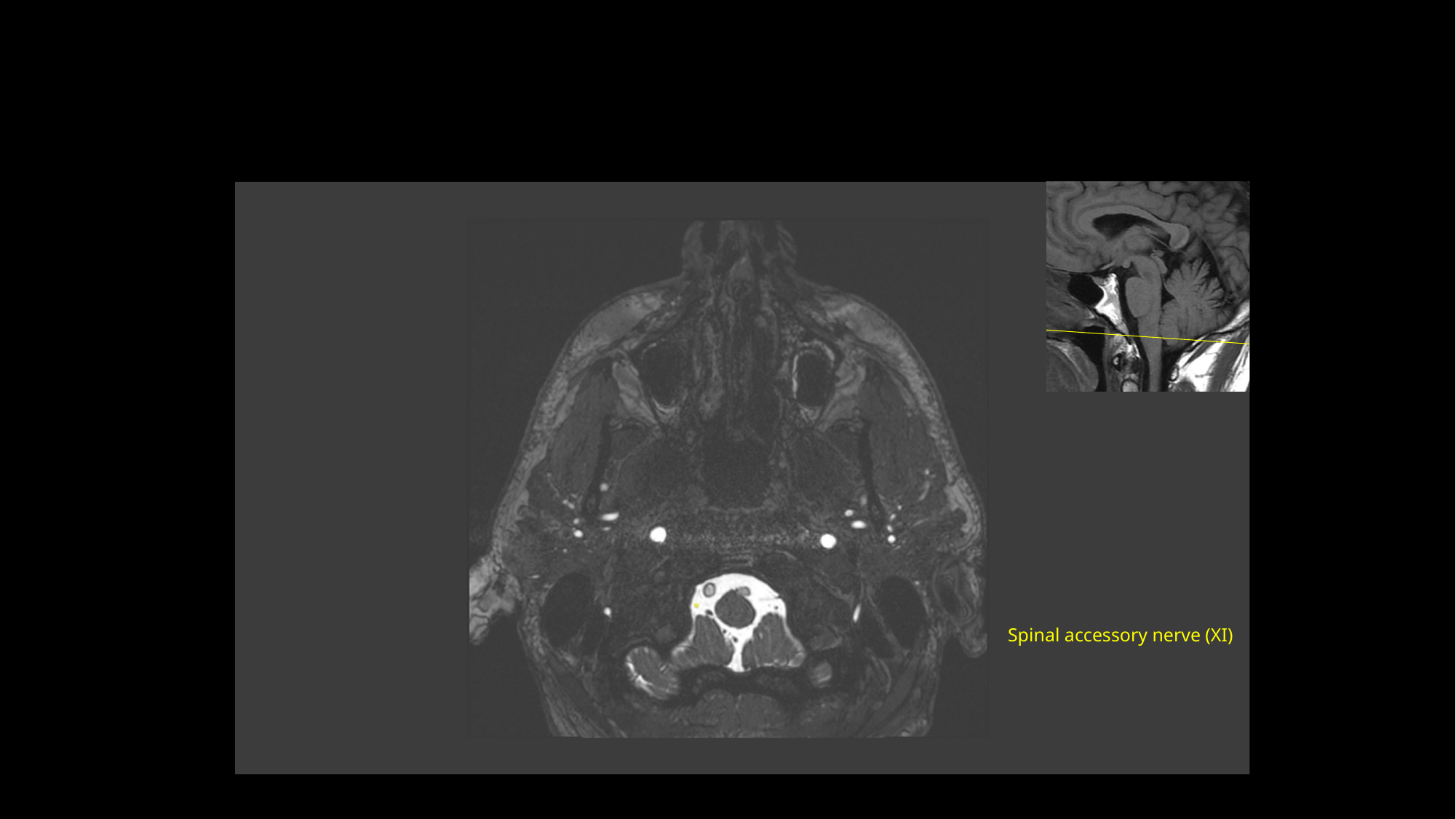

Spinal accessory nerve (XI)

## Slide 46
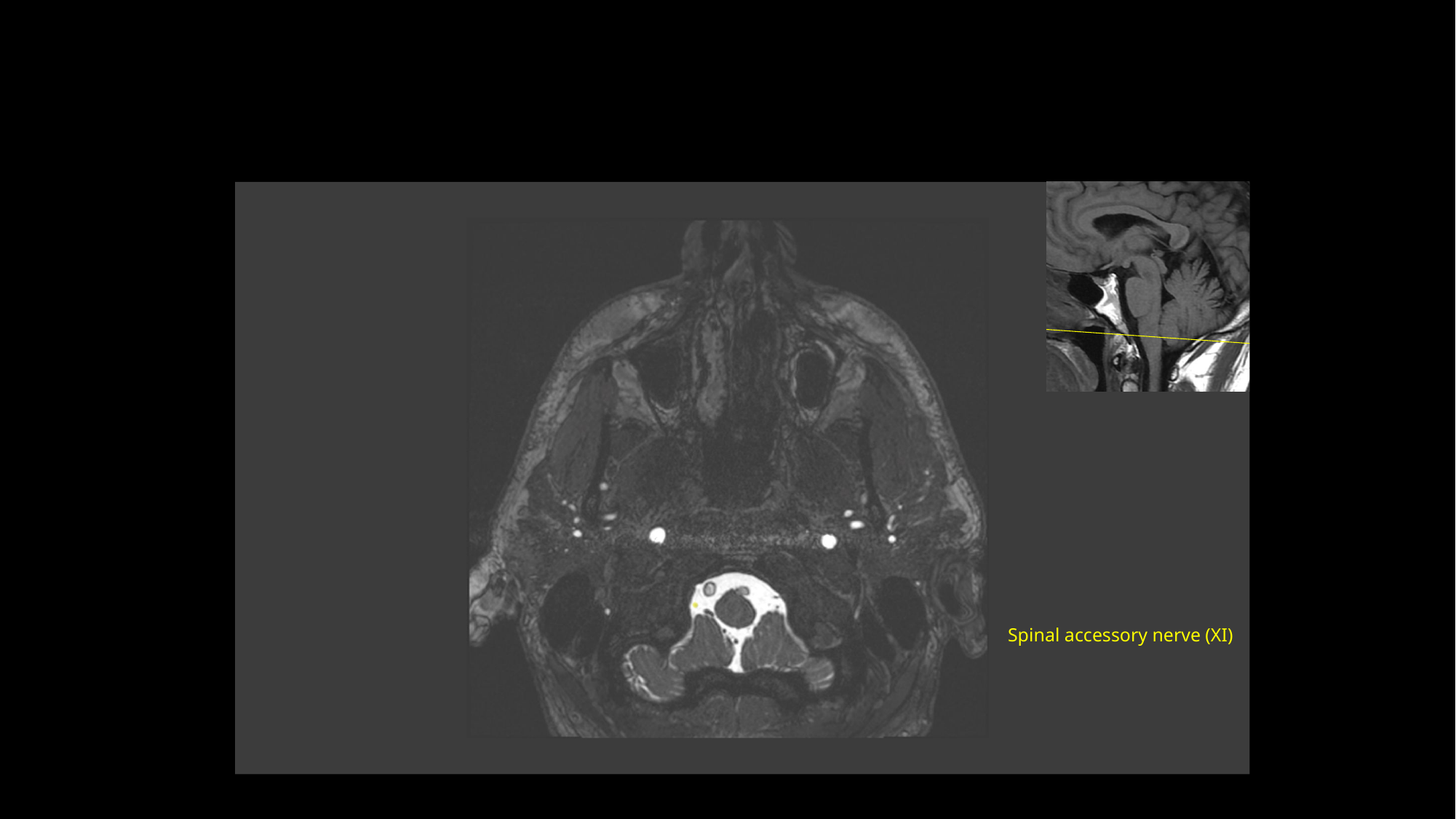

Spinal accessory nerve (XI)

## Slide 47
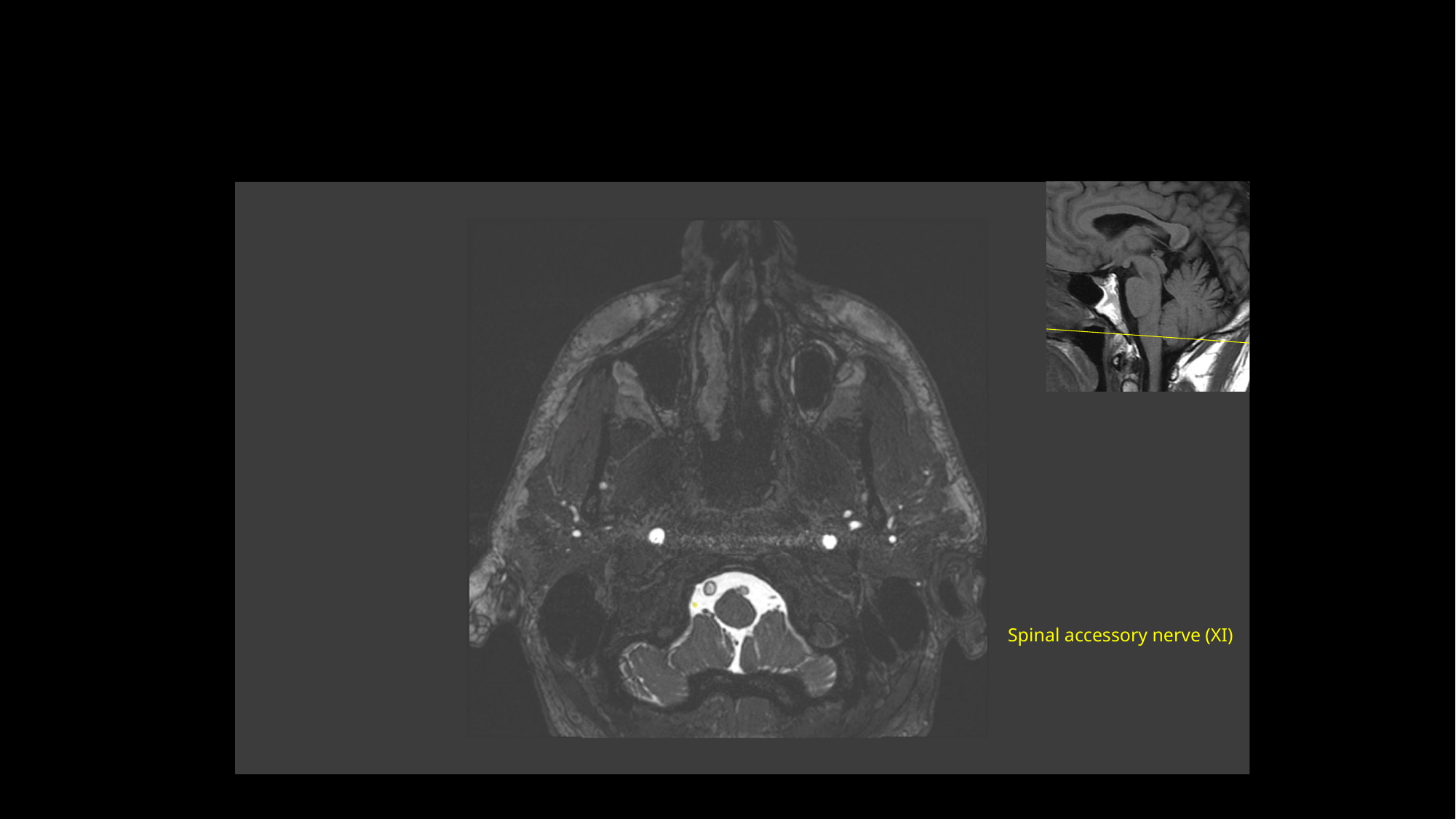

Spinal accessory nerve (XI)

## Slide 48
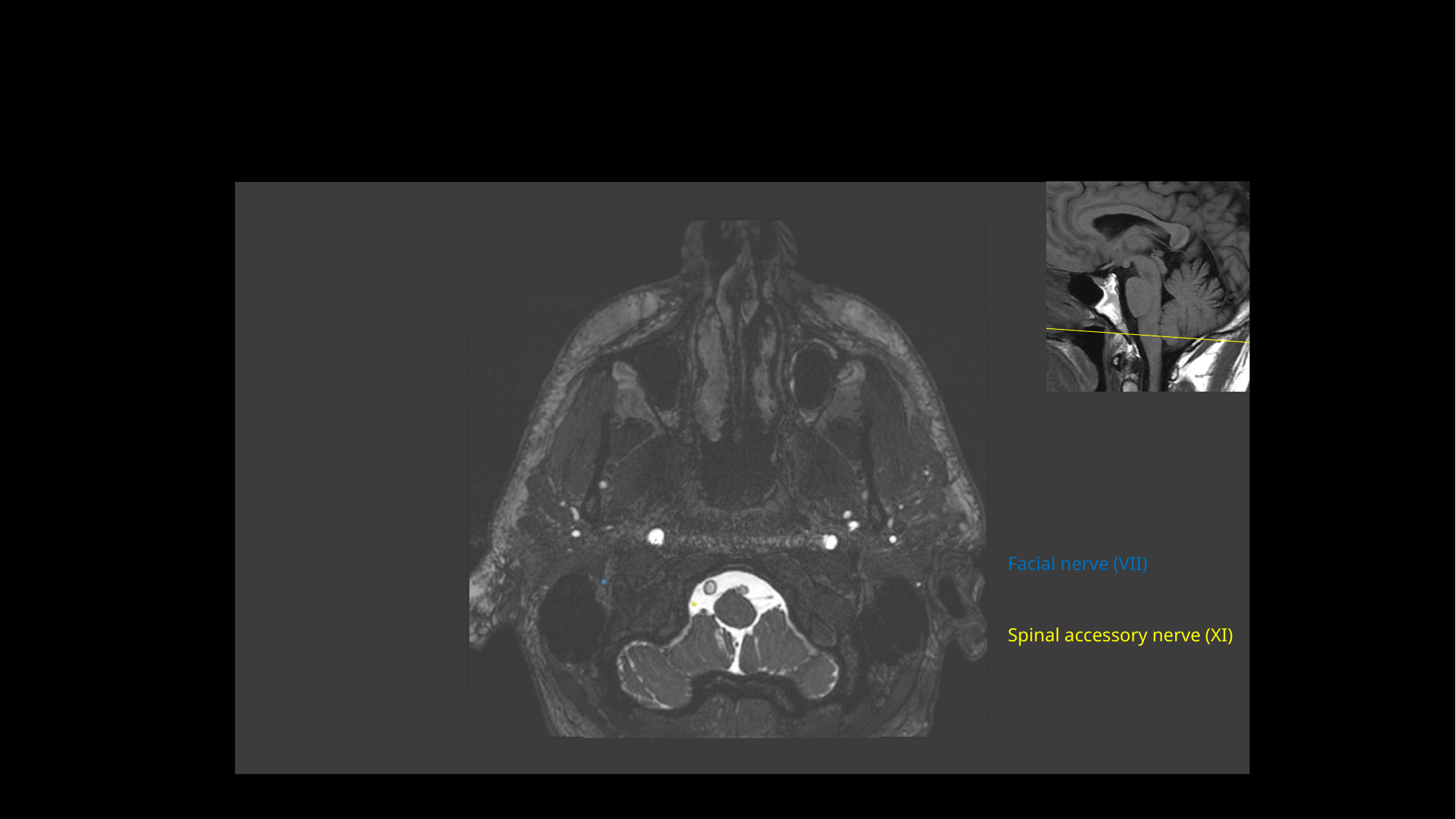

Facial nerve (VII)
Spinal accessory nerve (XI)

## Slide 49
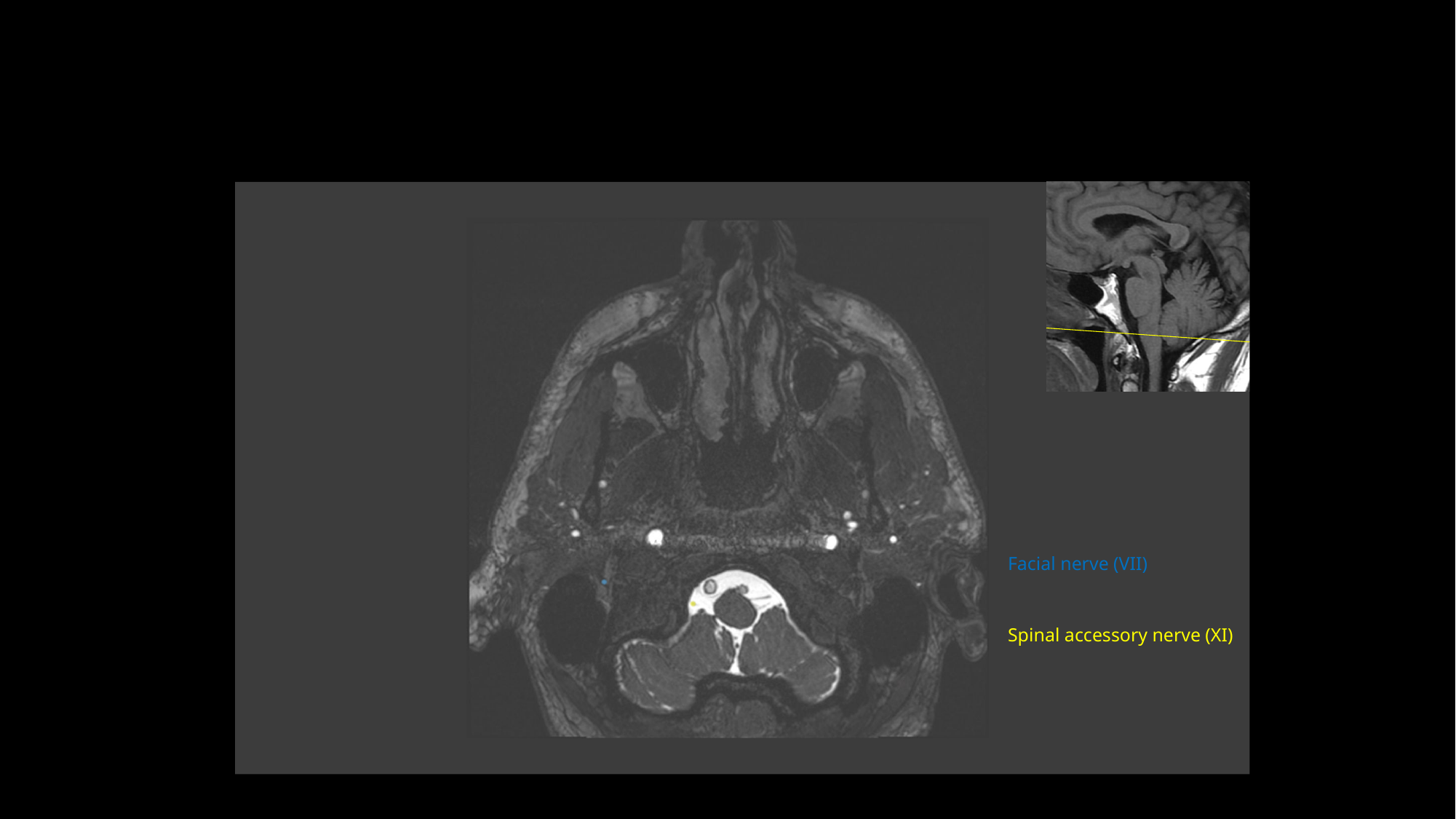

Facial nerve (VII)
Spinal accessory nerve (XI)

## Slide 50
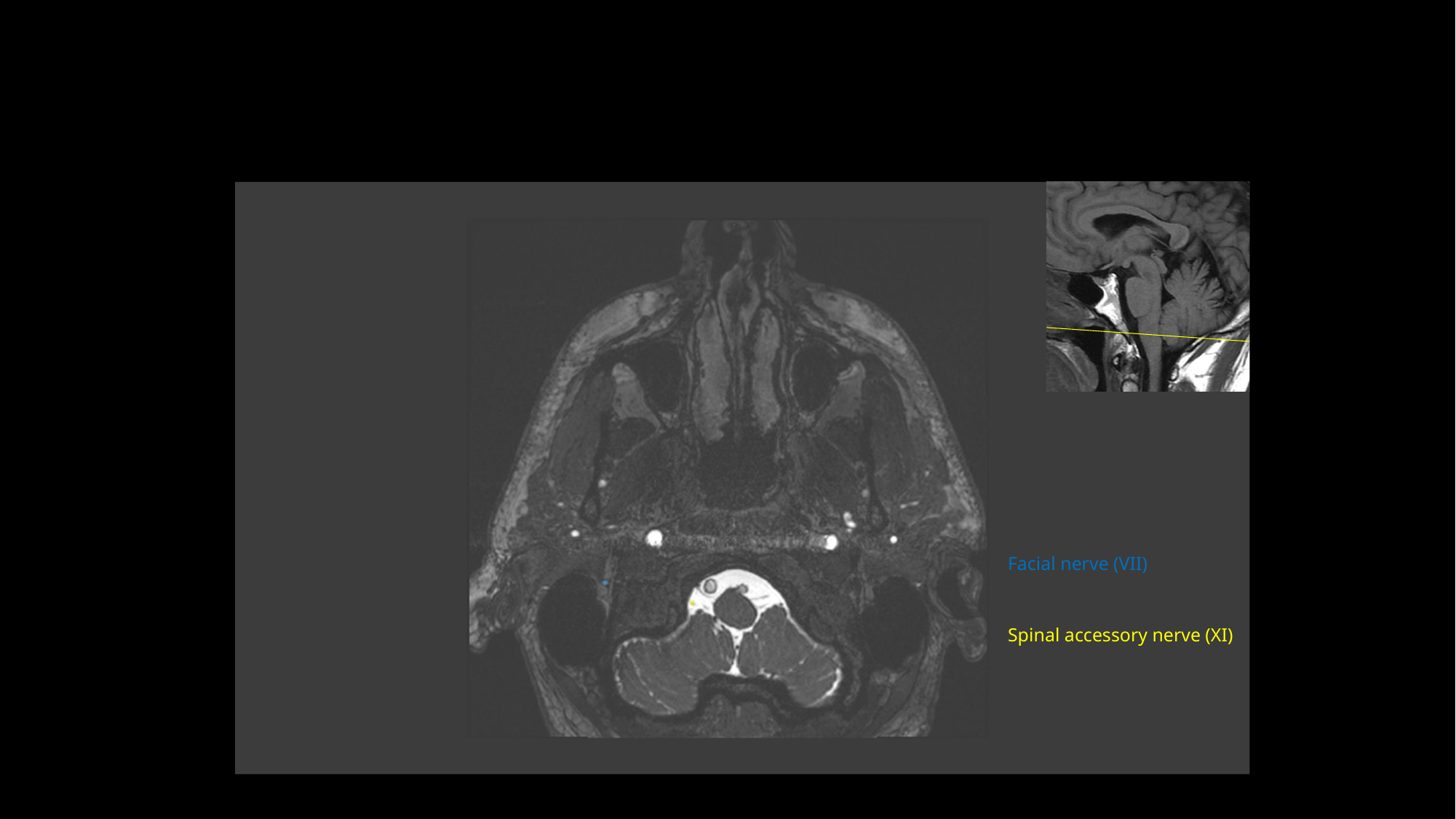

Facial nerve (VII)
Spinal accessory nerve (XI)

## Slide 51
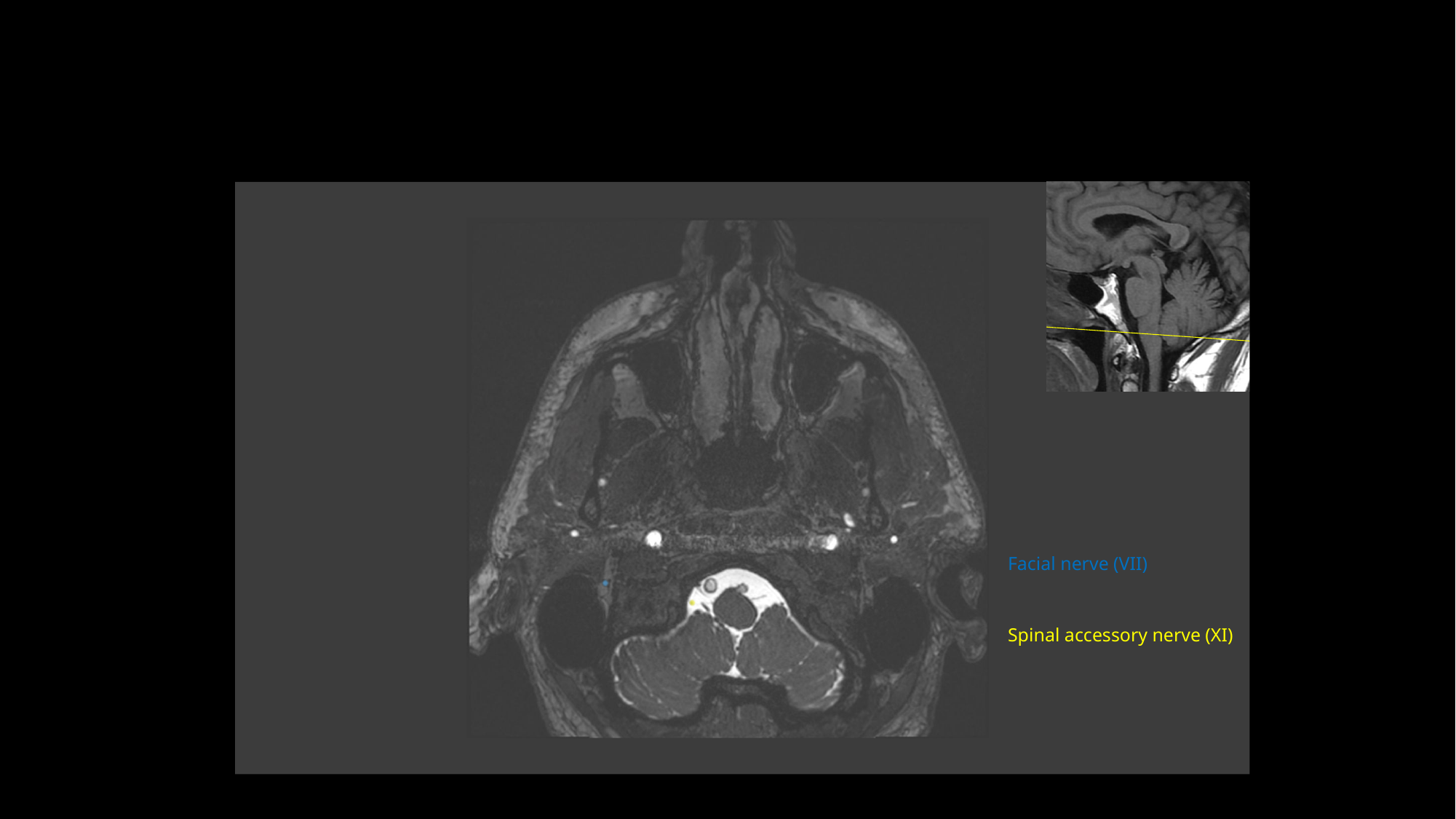

Facial nerve (VII)
Spinal accessory nerve (XI)

## Slide 52
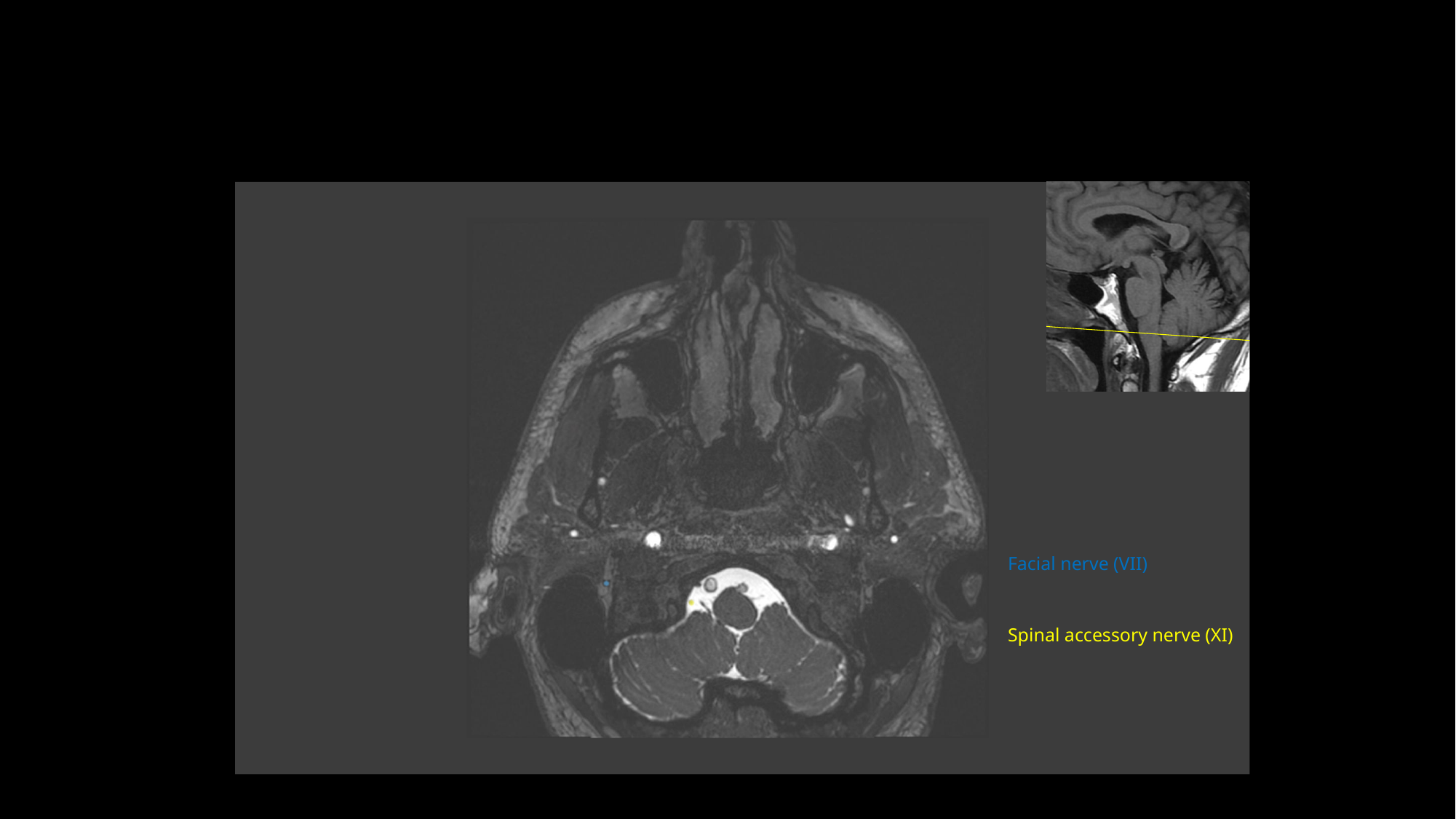

Facial nerve (VII)
Spinal accessory nerve (XI)

## Slide 53
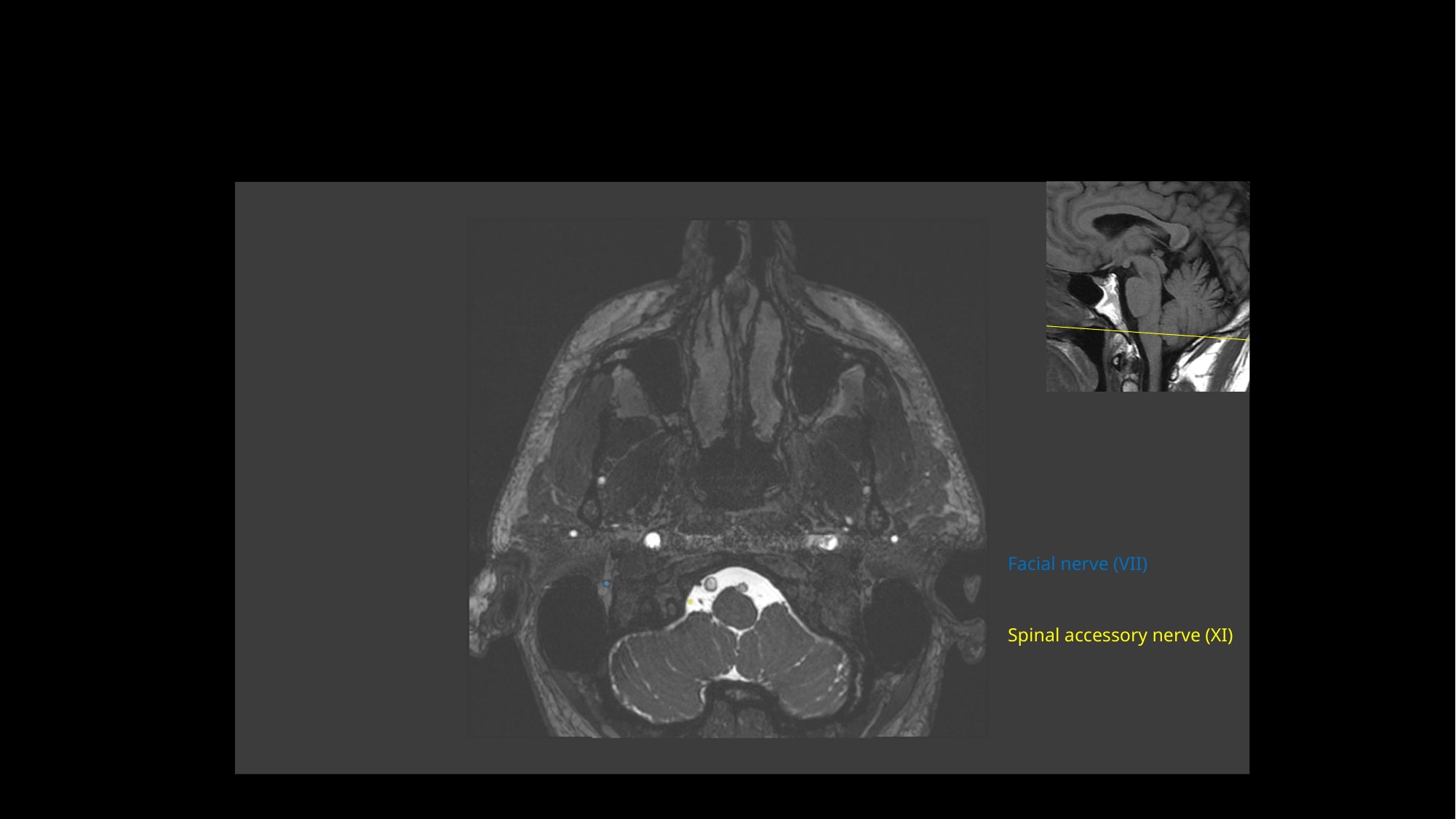

Facial nerve (VII)
Spinal accessory nerve (XI)

## Slide 54
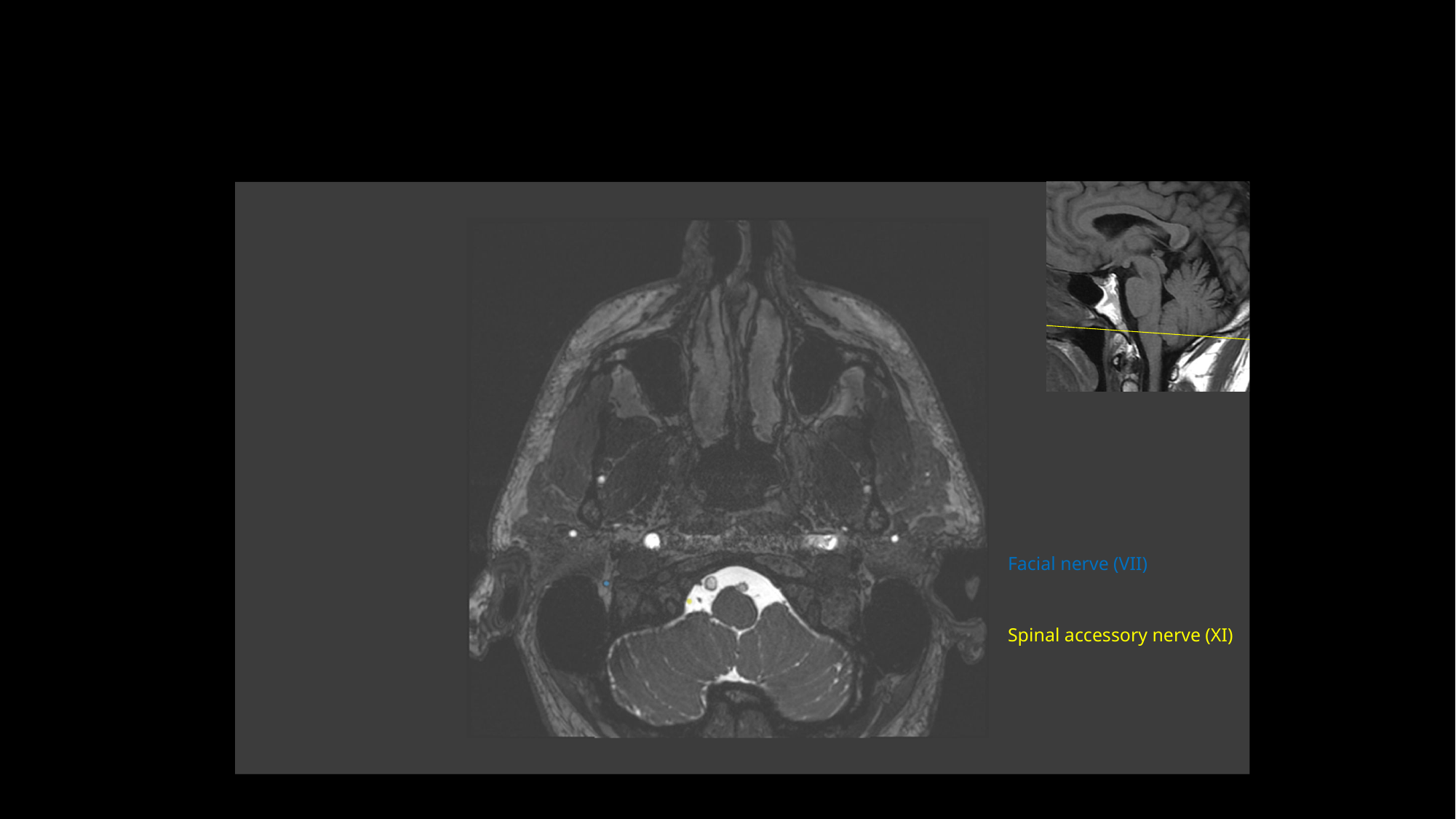

Facial nerve (VII)
Spinal accessory nerve (XI)

## Slide 55
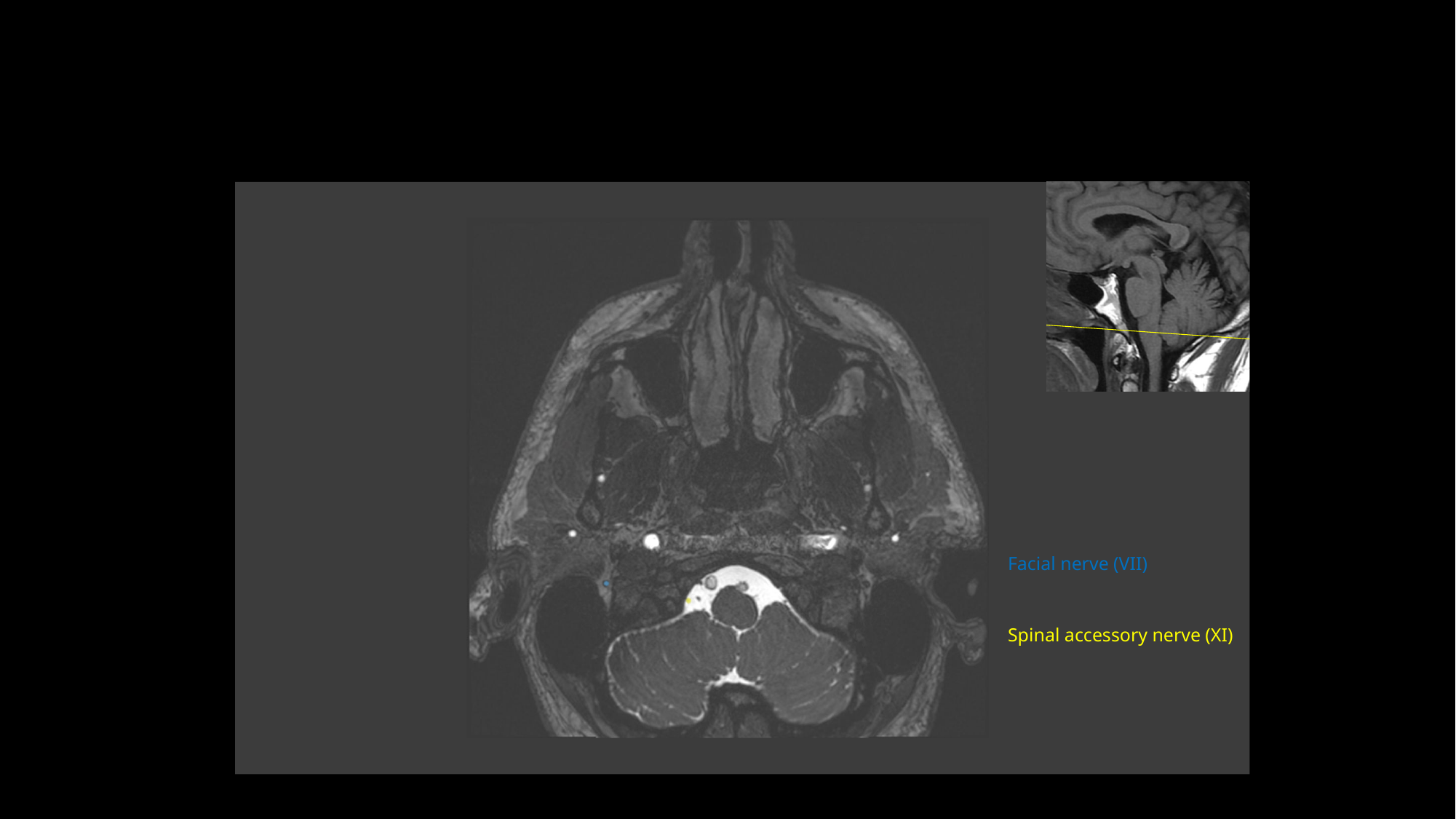

Facial nerve (VII)
Spinal accessory nerve (XI)

## Slide 56
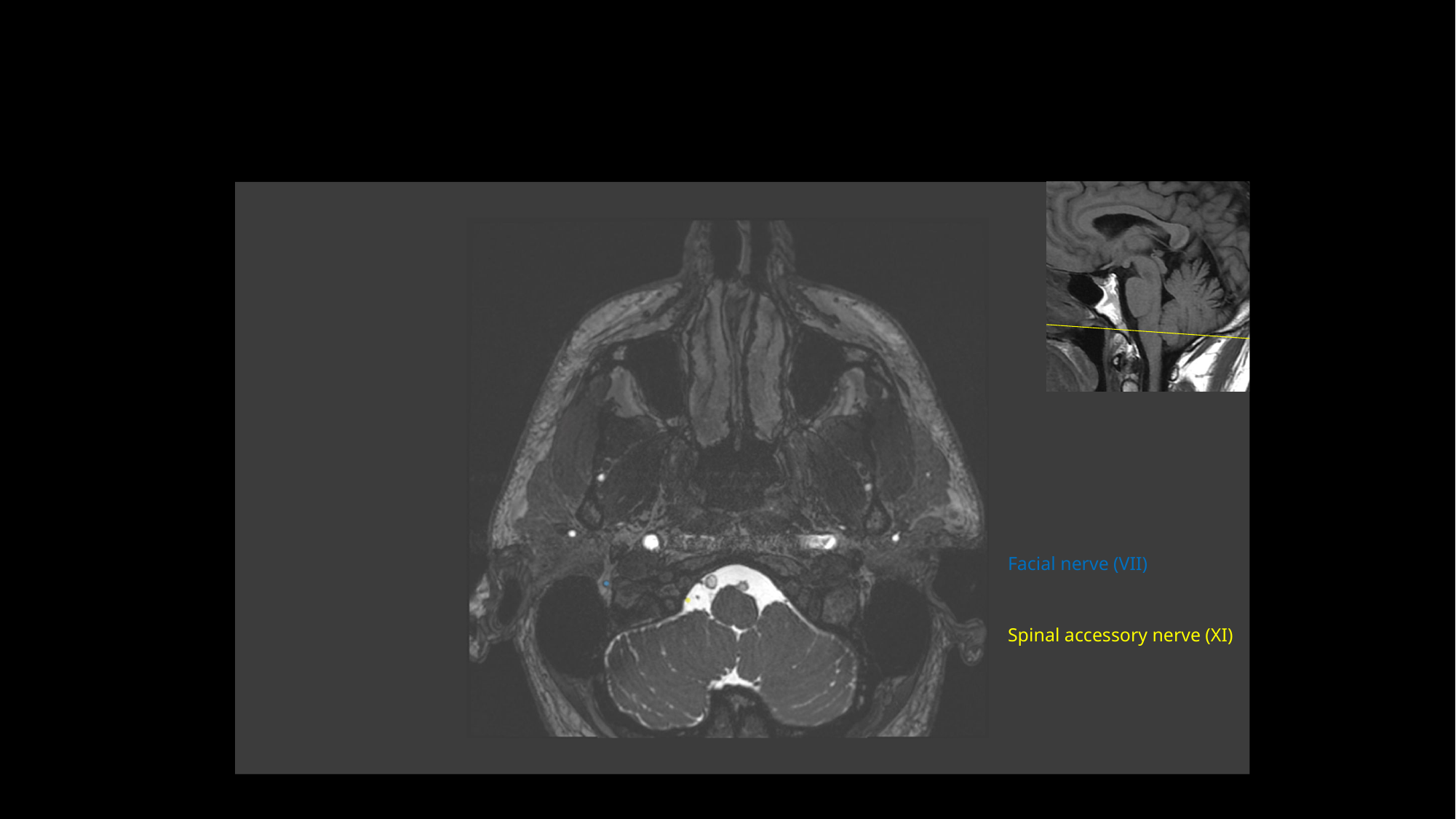

Facial nerve (VII)
Spinal accessory nerve (XI)

## Slide 57
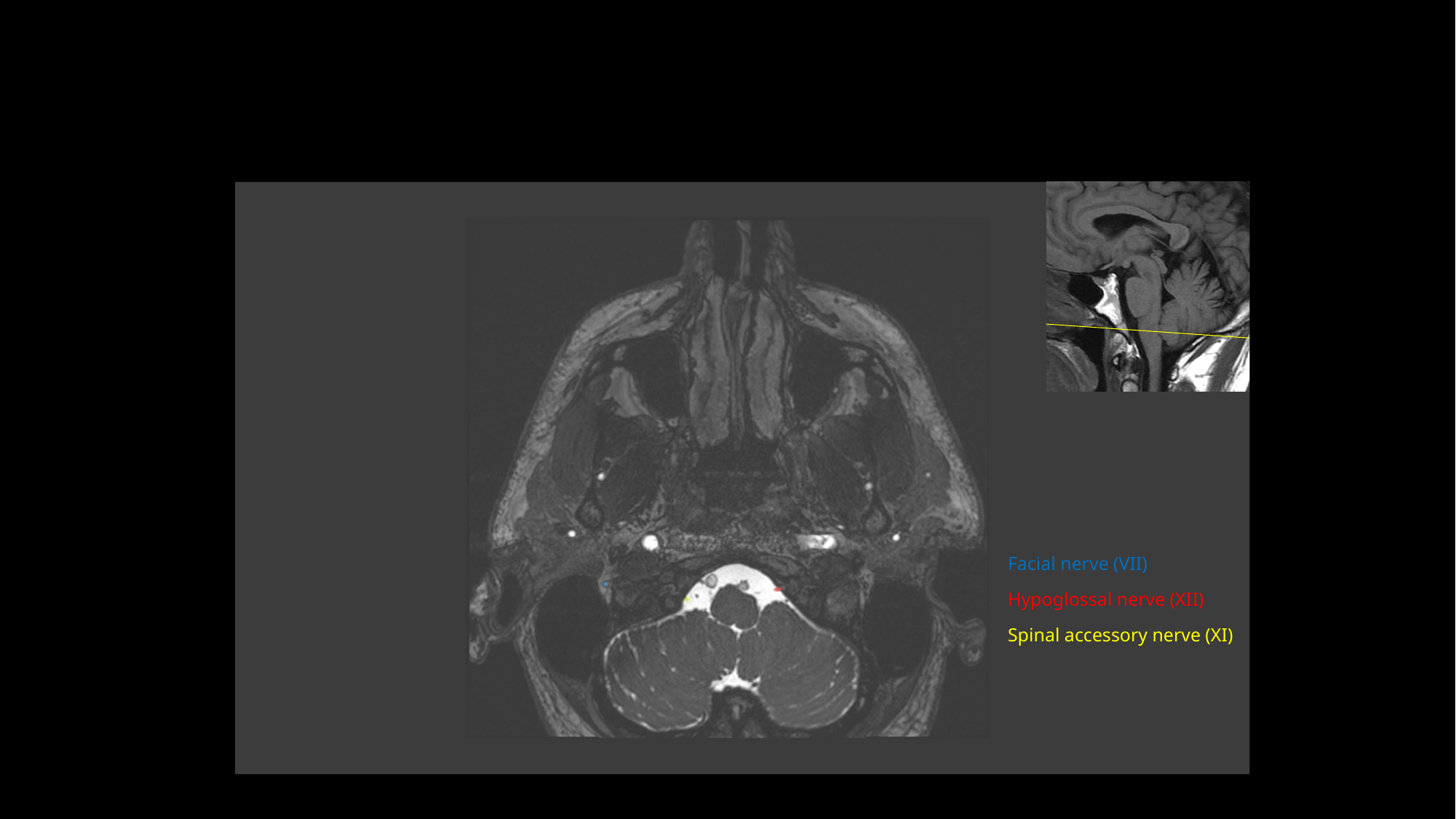

Facial nerve (VII)
Hypoglossal nerve (XII)
Spinal accessory nerve (XI)

## Slide 58
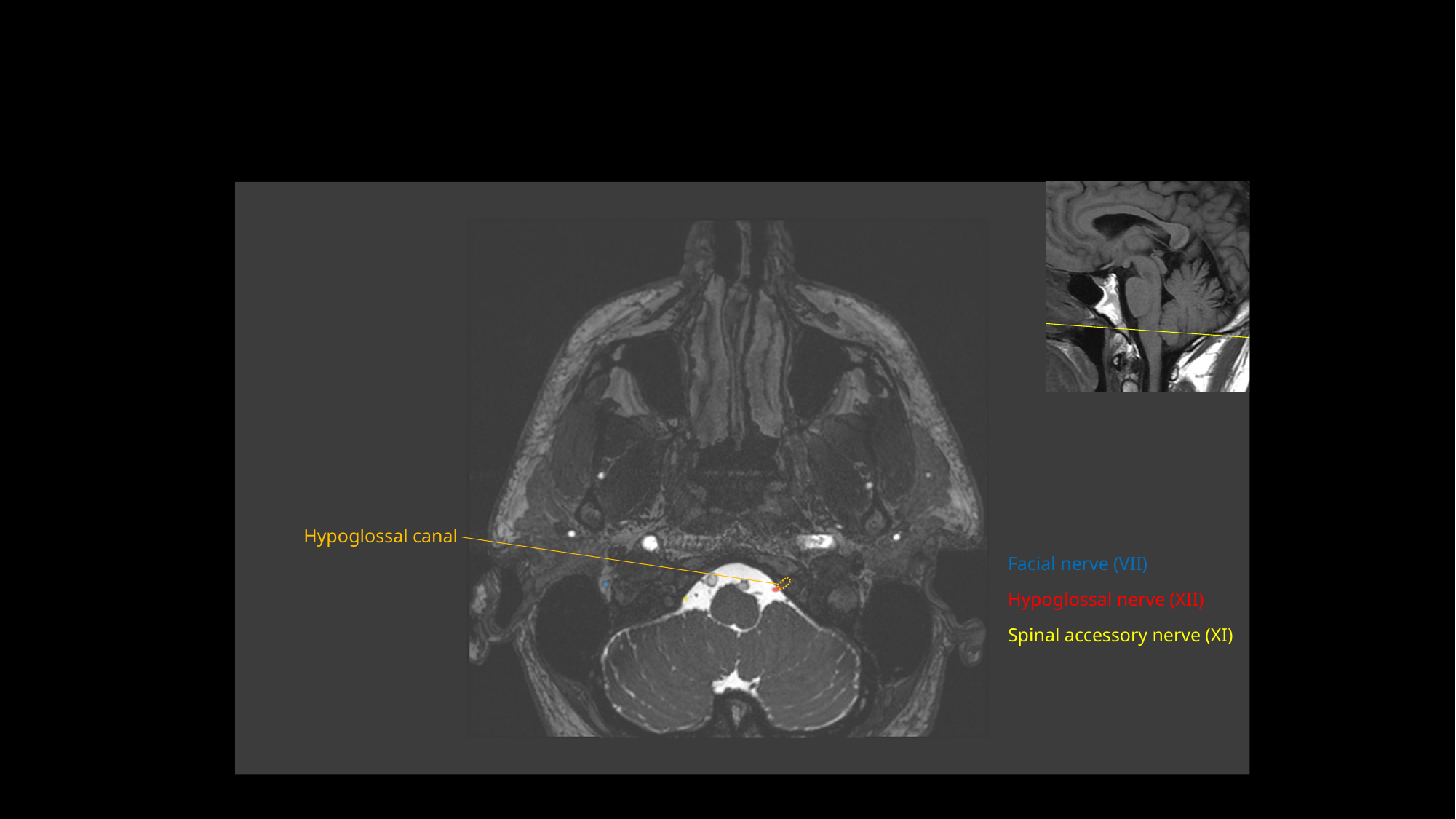

Hypoglossal canal
Facial nerve (VII)
Hypoglossal nerve (XII)
Spinal accessory nerve (XI)

## Slide 59
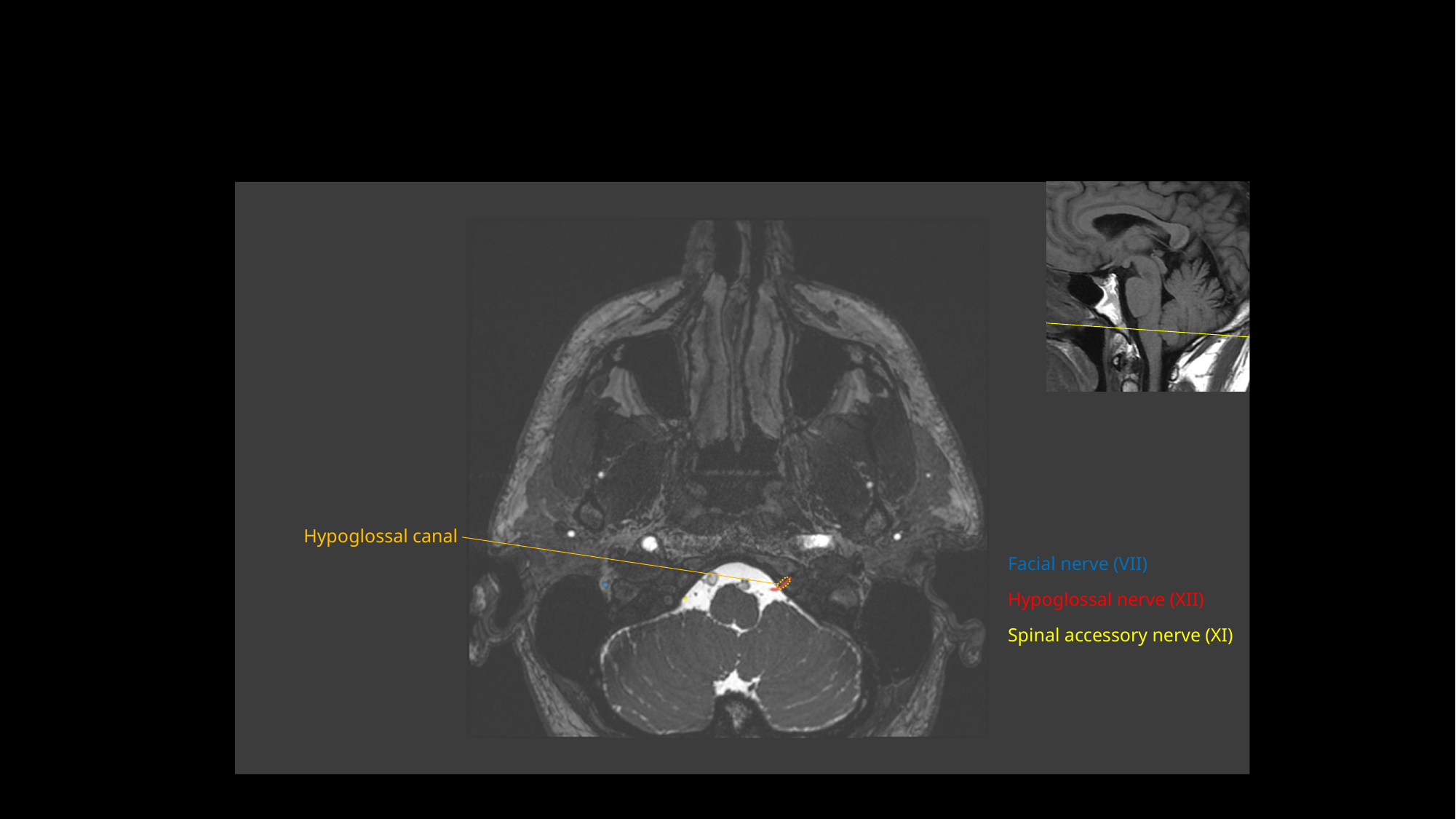

Hypoglossal canal
Facial nerve (VII)
Hypoglossal nerve (XII)
Spinal accessory nerve (XI)

## Slide 60
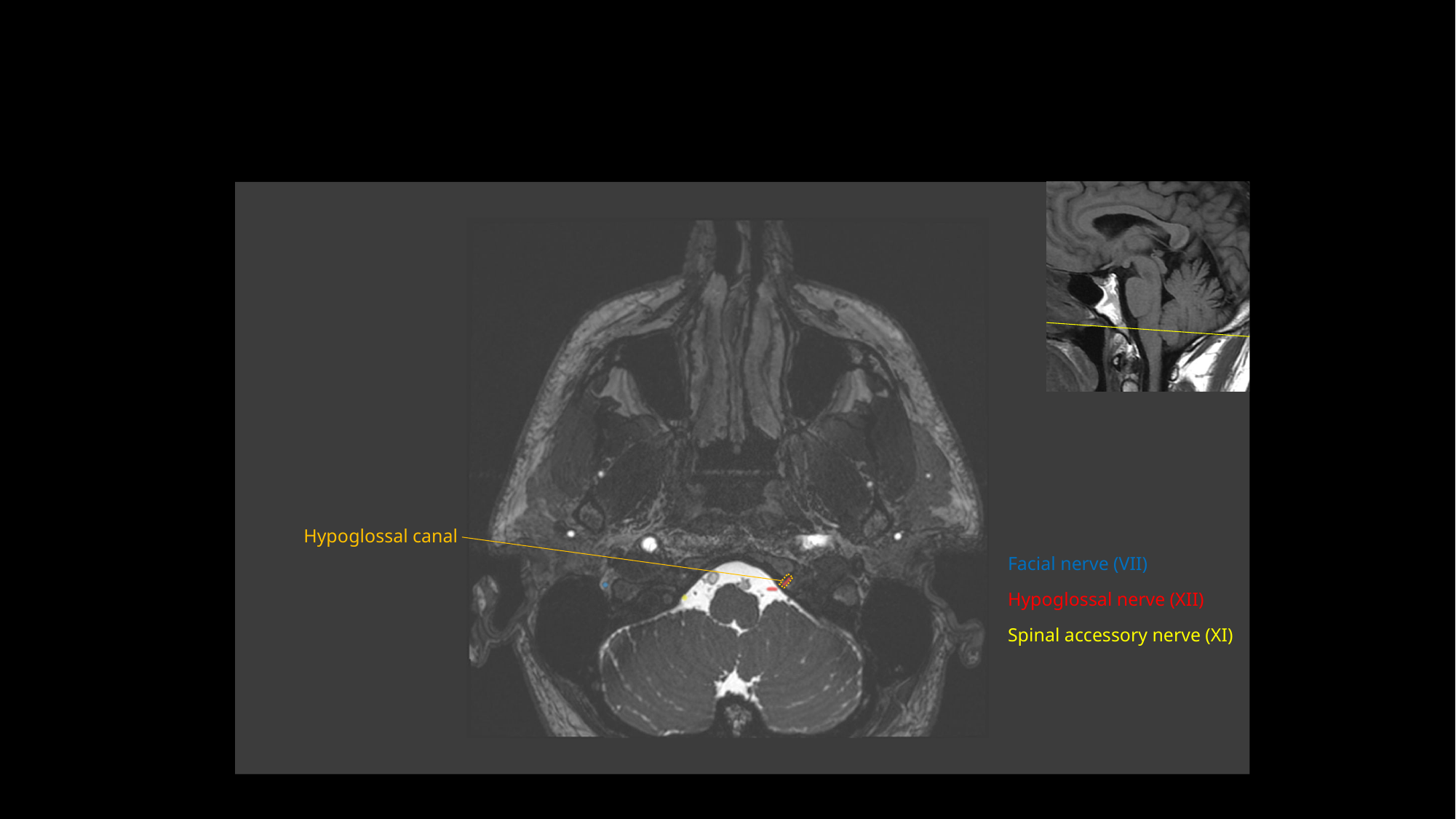

Hypoglossal canal
Facial nerve (VII)
Hypoglossal nerve (XII)
Spinal accessory nerve (XI)

## Slide 61
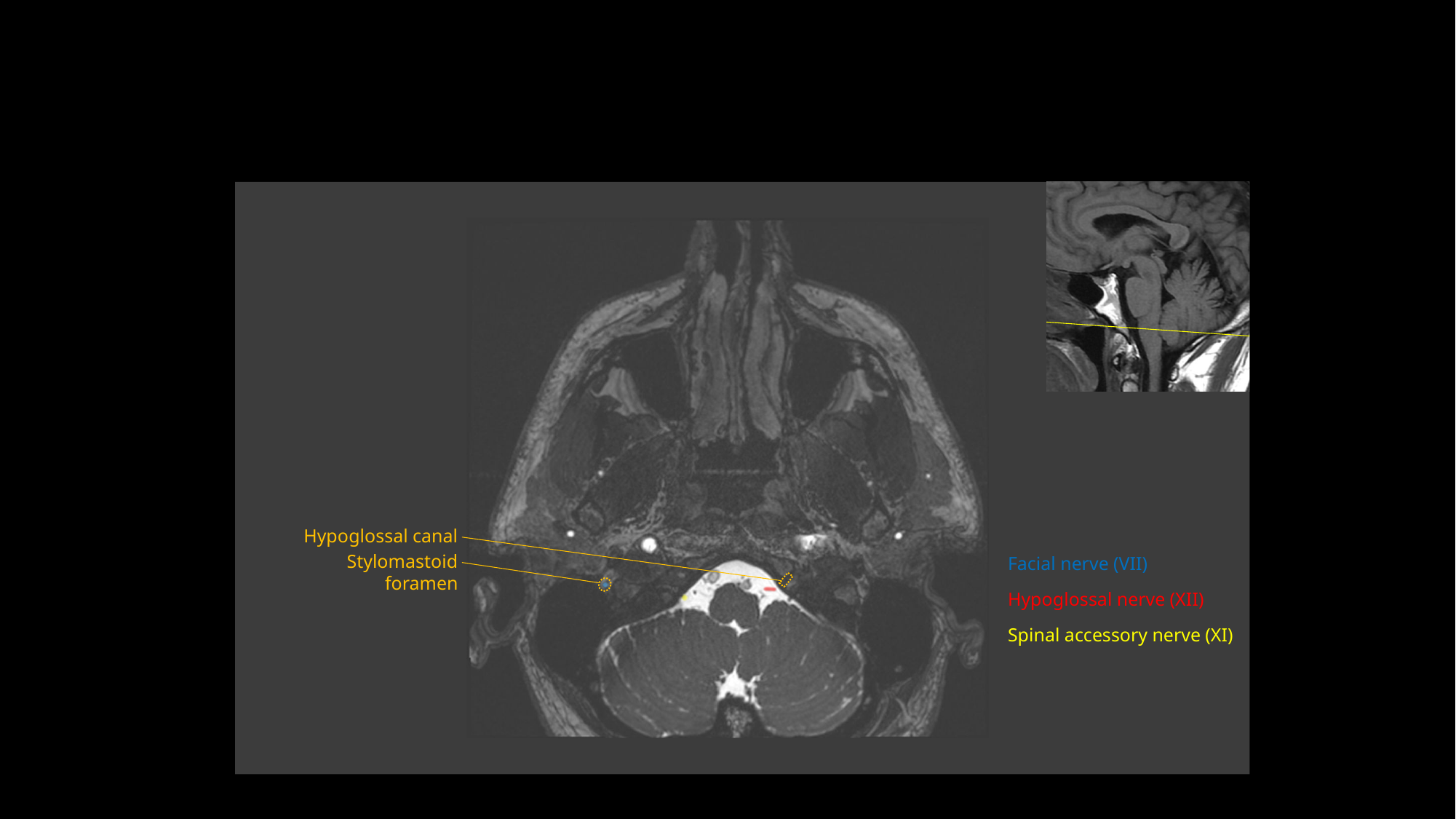

Hypoglossal canal
Stylomastoid foramen
Facial nerve (VII)
Hypoglossal nerve (XII)
Spinal accessory nerve (XI)

## Slide 62
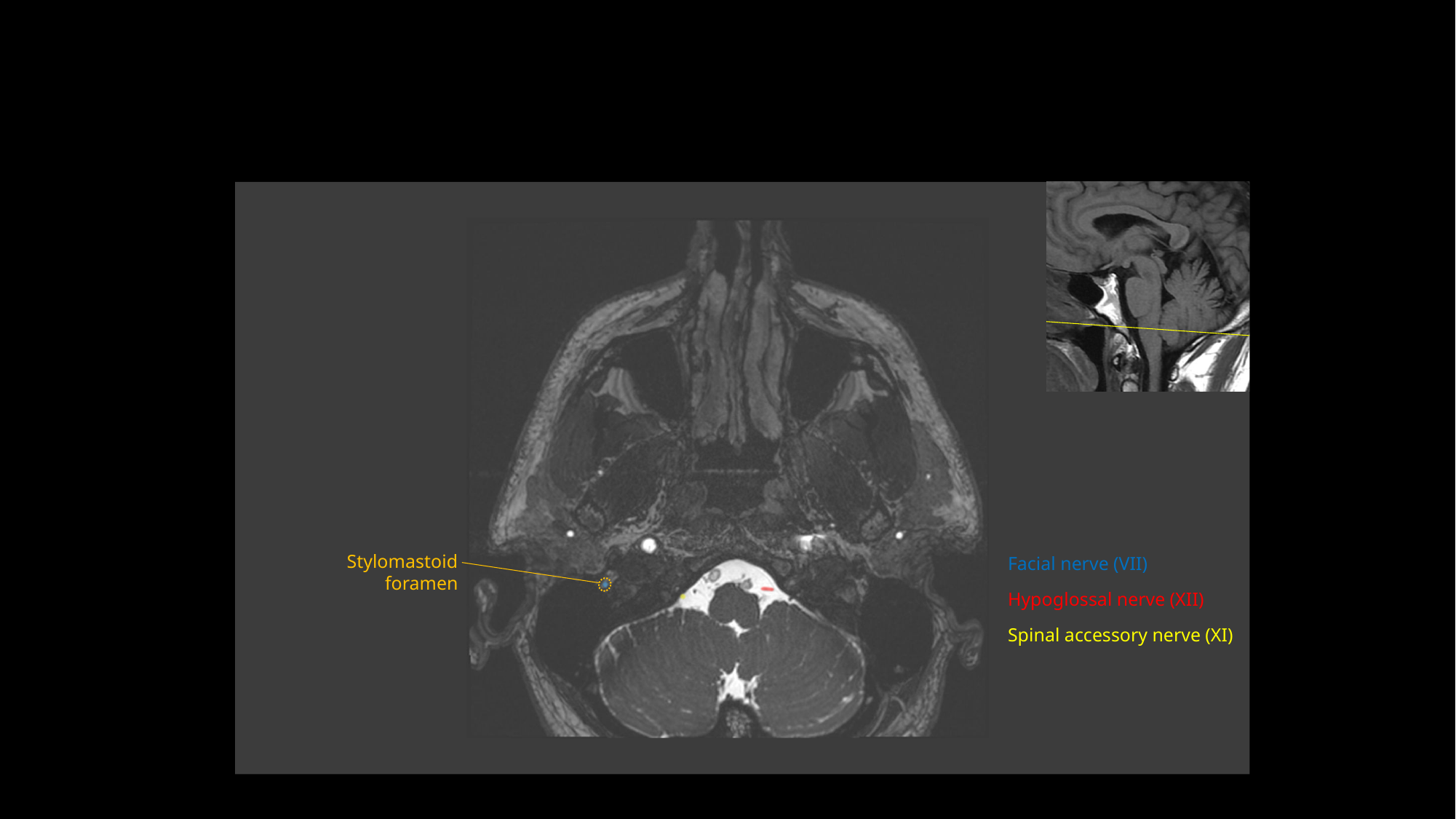

Stylomastoid foramen
Facial nerve (VII)
Hypoglossal nerve (XII)
Spinal accessory nerve (XI)

## Slide 63
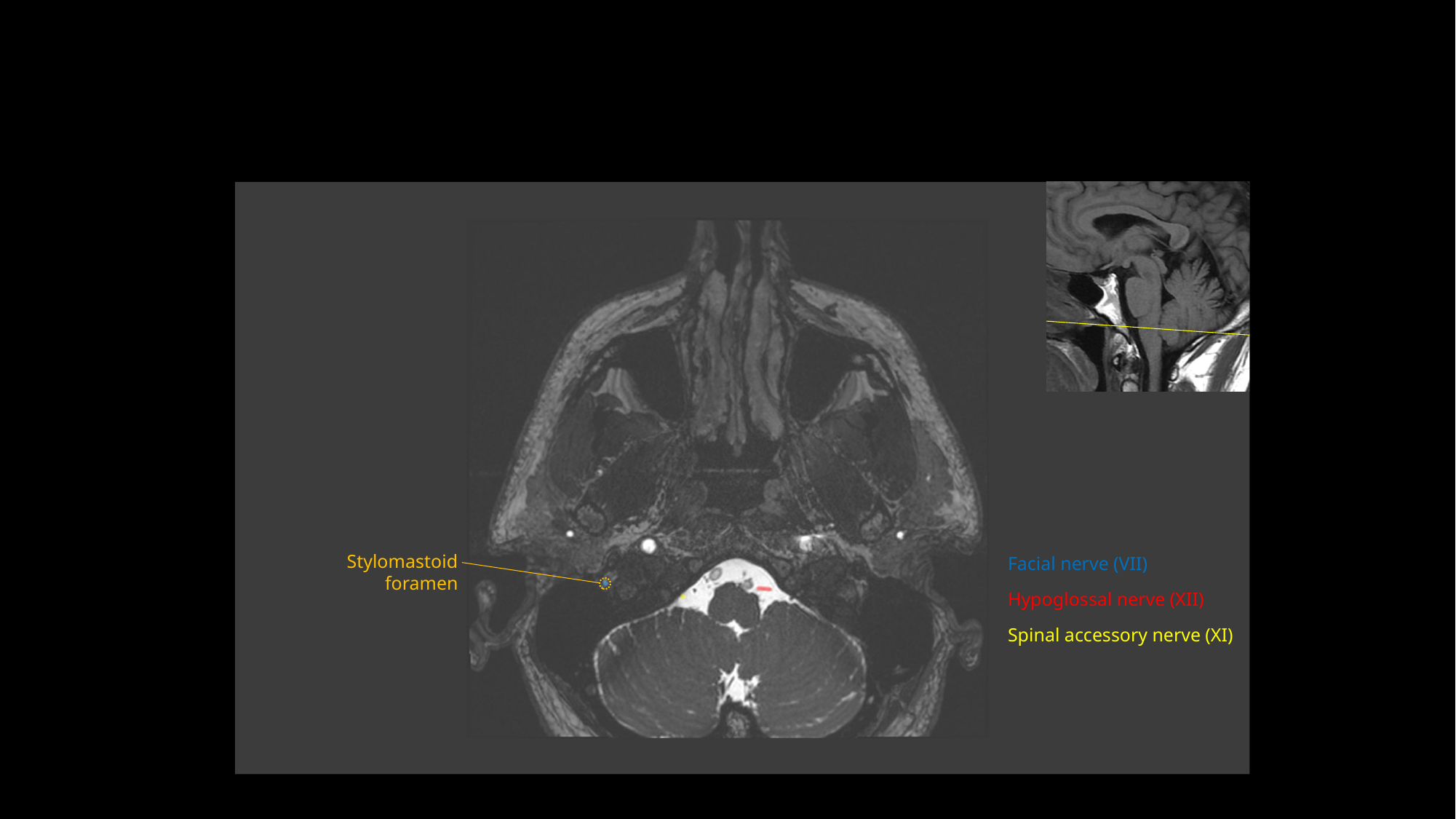

Stylomastoid foramen
Facial nerve (VII)
Hypoglossal nerve (XII)
Spinal accessory nerve (XI)

## Slide 64
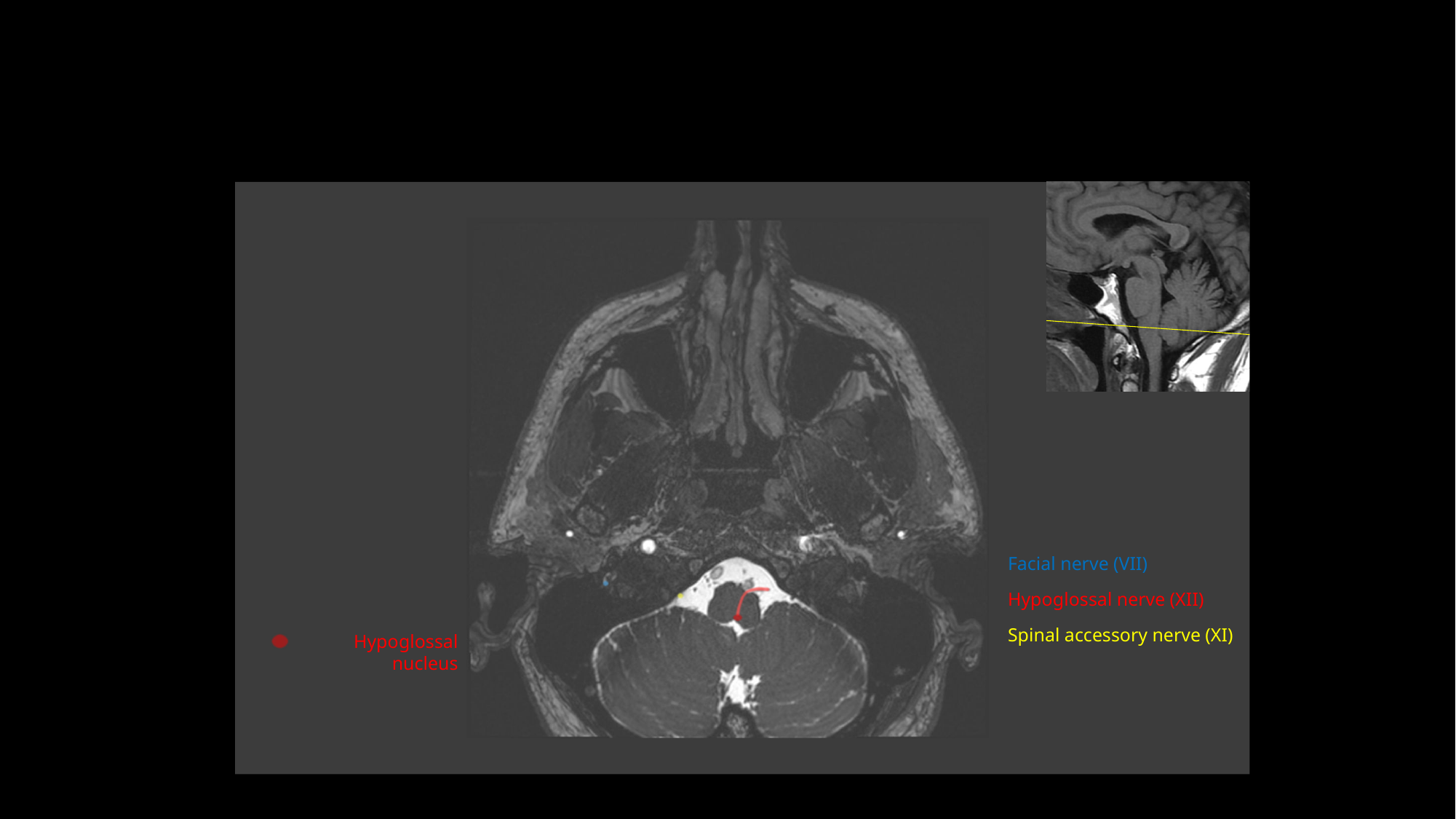

Facial nerve (VII)
Hypoglossal nerve (XII)
Spinal accessory nerve (XI)
Hypoglossal nucleus

## Slide 65
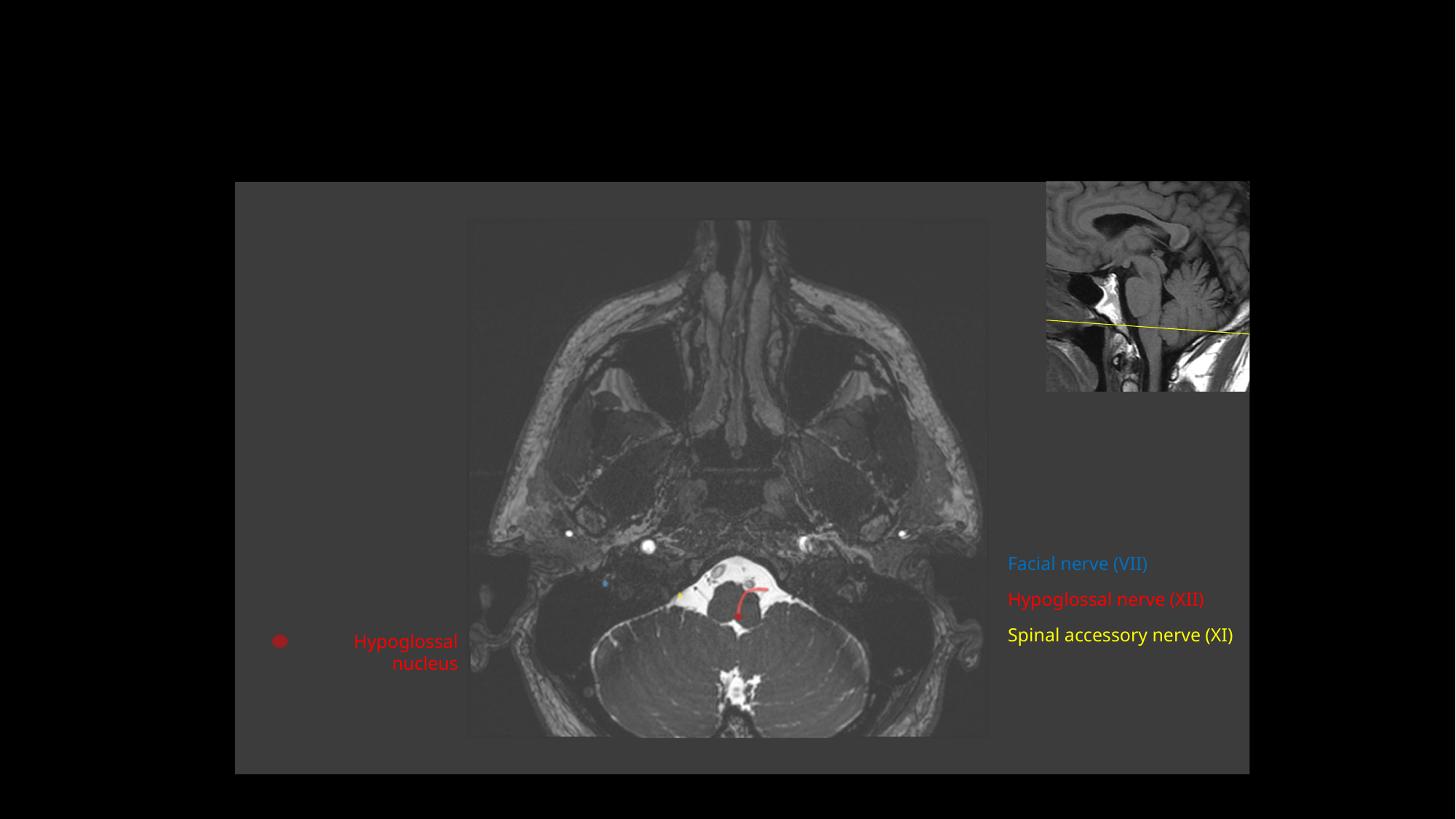

Facial nerve (VII)
Hypoglossal nerve (XII)
Spinal accessory nerve (XI)
Hypoglossal nucleus

## Slide 66
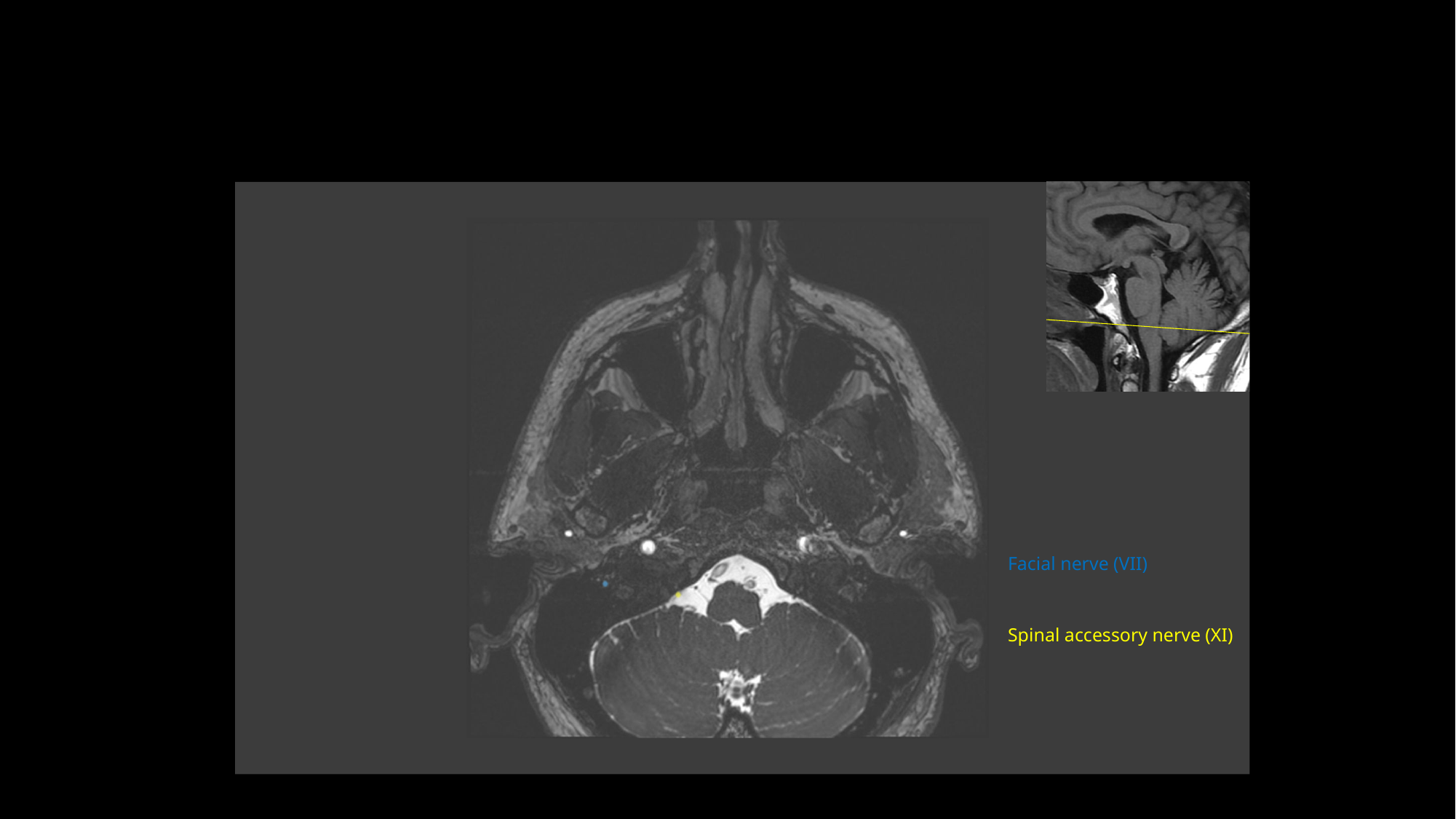

Facial nerve (VII)
Spinal accessory nerve (XI)

## Slide 67
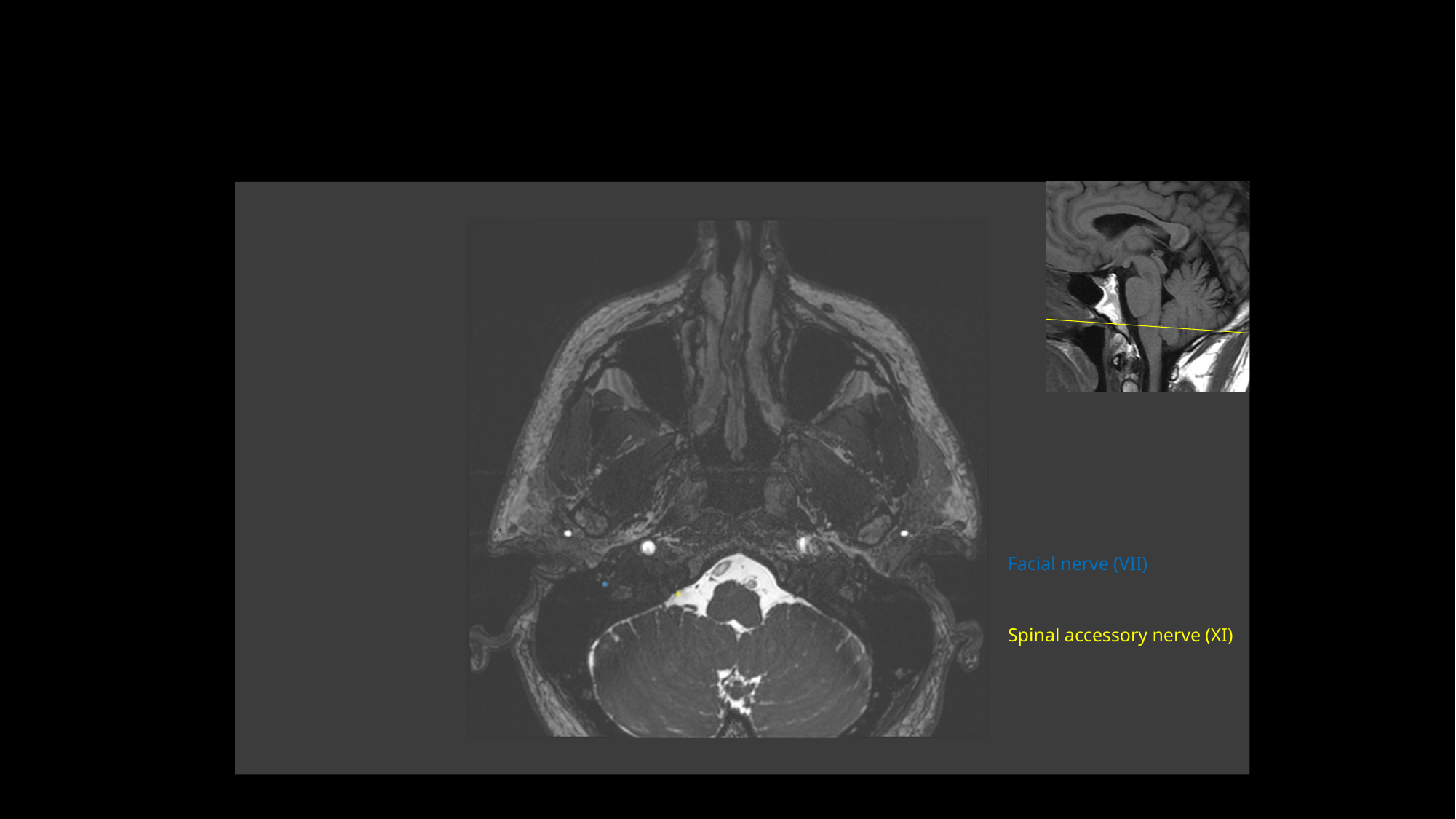

Facial nerve (VII)
Spinal accessory nerve (XI)

## Slide 68
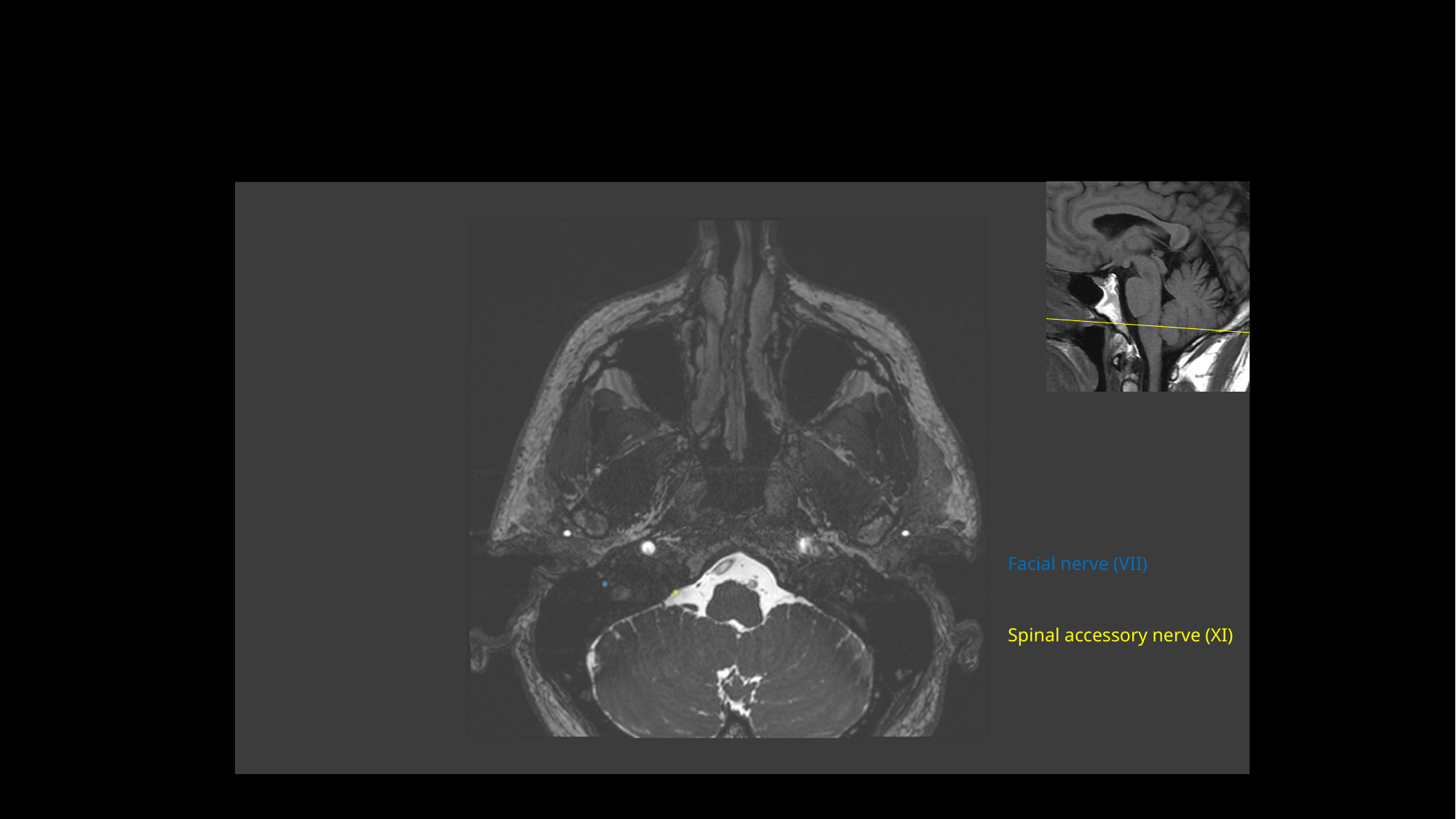

Facial nerve (VII)
Spinal accessory nerve (XI)

## Slide 69
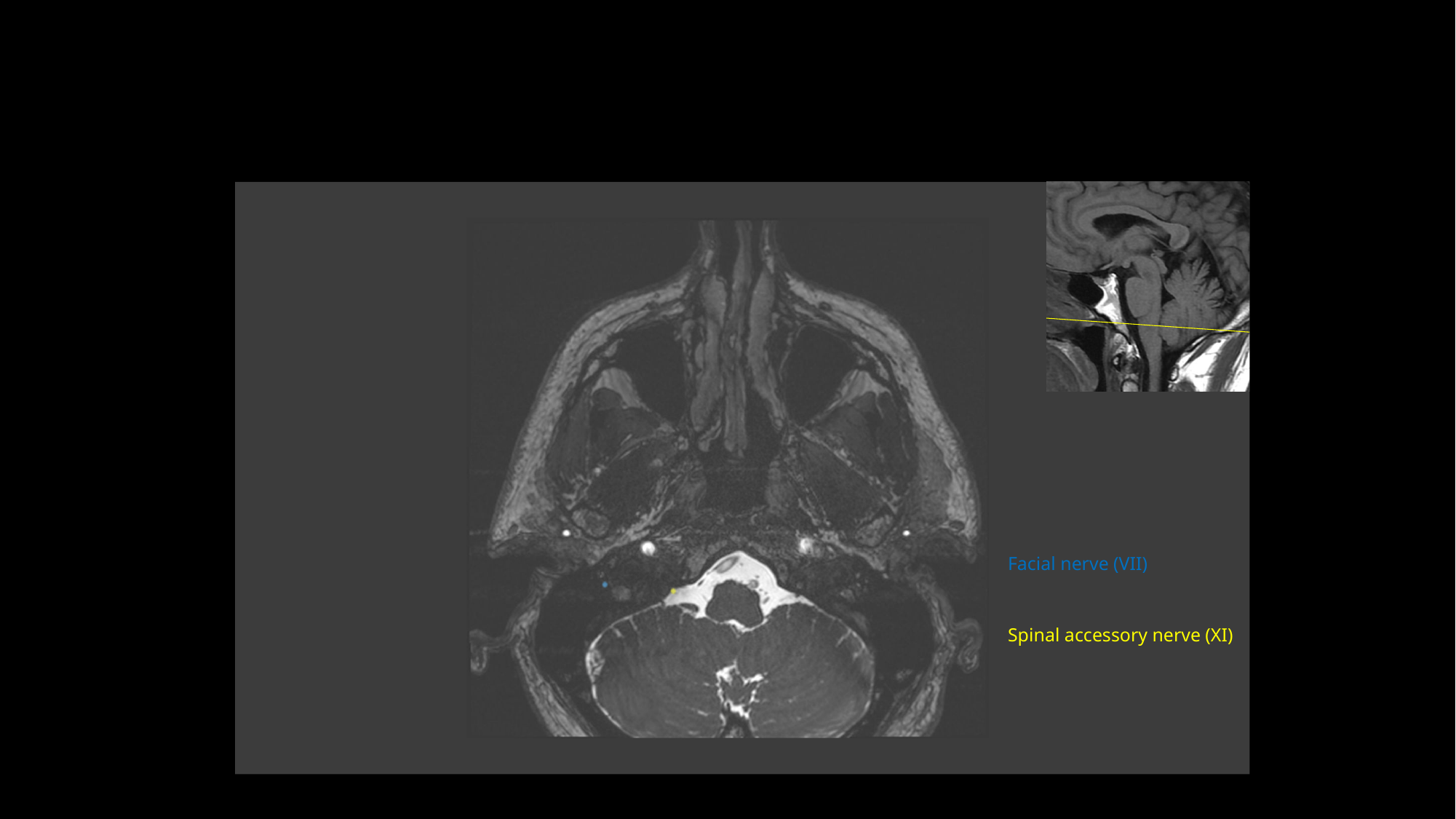

Facial nerve (VII)
Spinal accessory nerve (XI)

## Slide 70
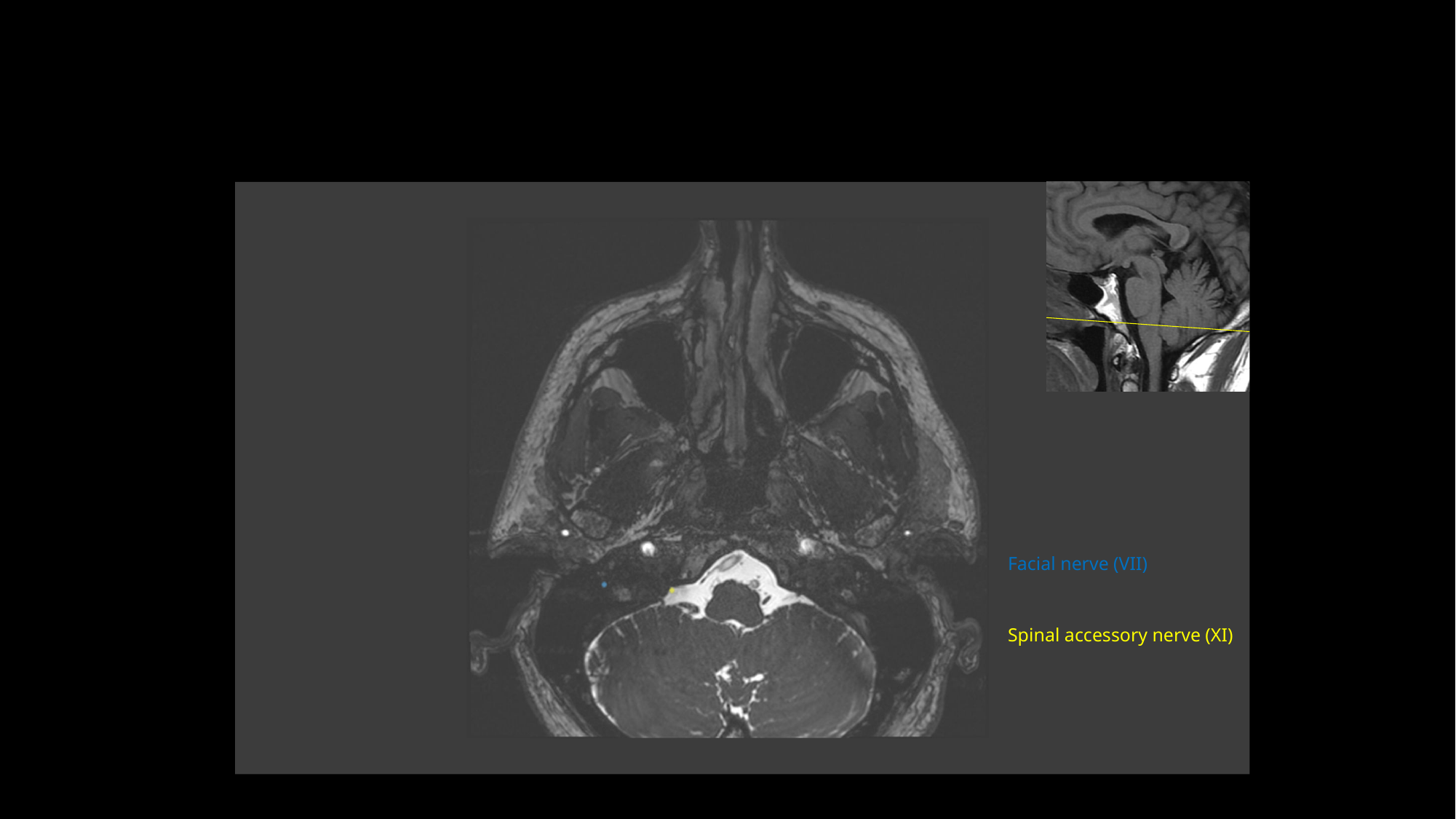

Facial nerve (VII)
Spinal accessory nerve (XI)

## Slide 71
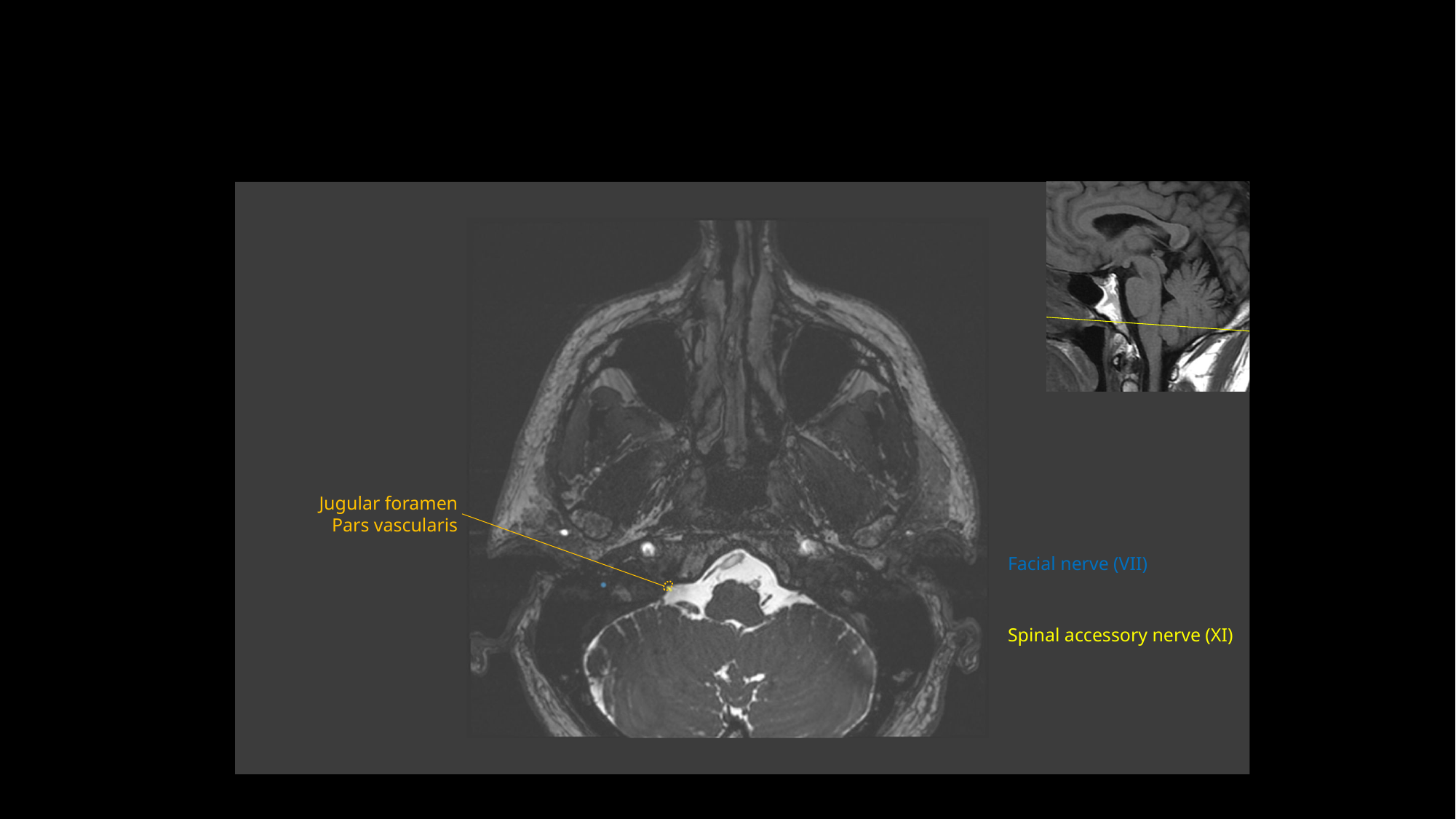

Jugular foramen
Pars vascularis
Facial nerve (VII)
Spinal accessory nerve (XI)

## Slide 72
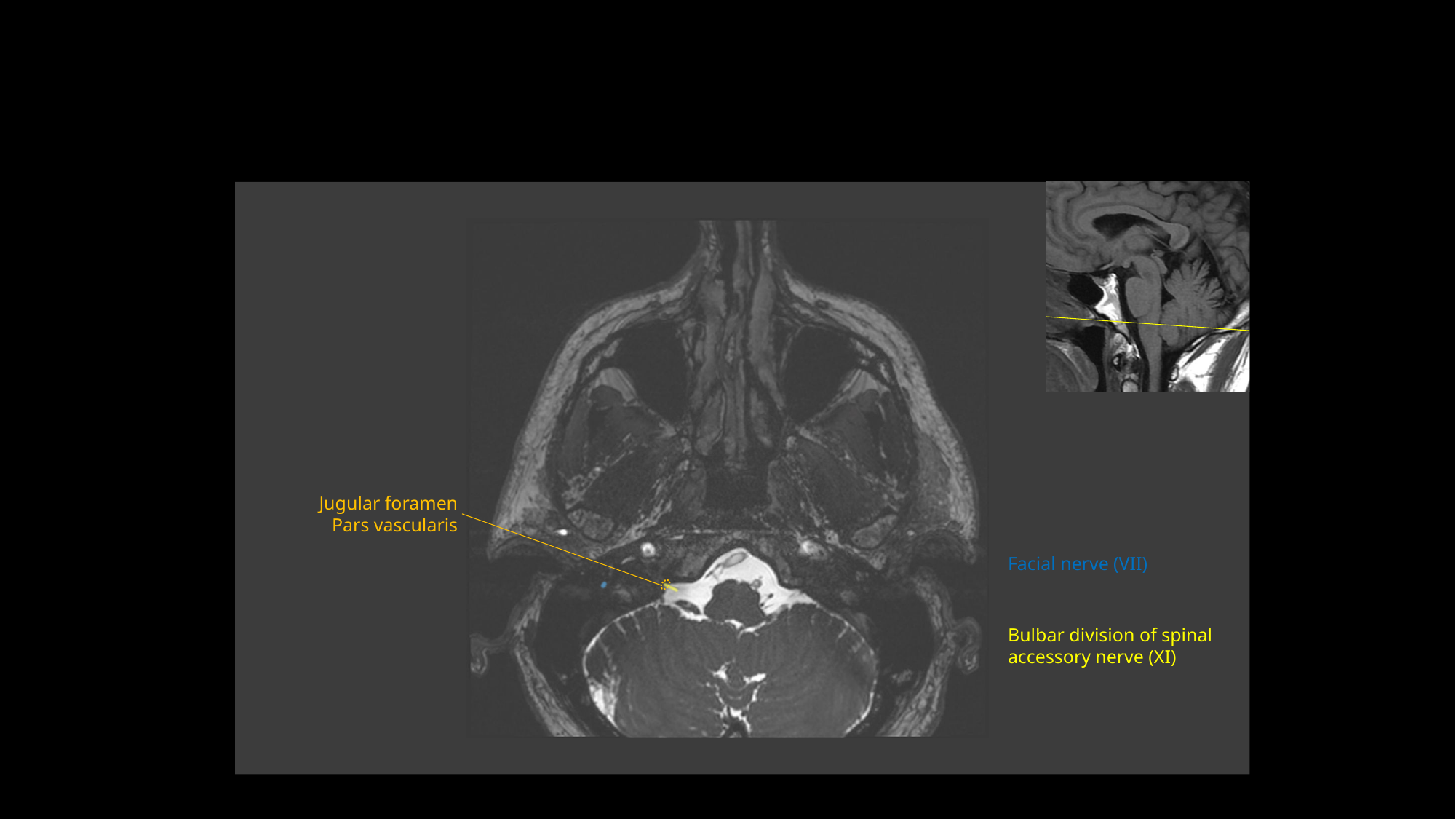

Jugular foramen
Pars vascularis
Facial nerve (VII)
Bulbar division of spinal accessory nerve (XI)

## Slide 73
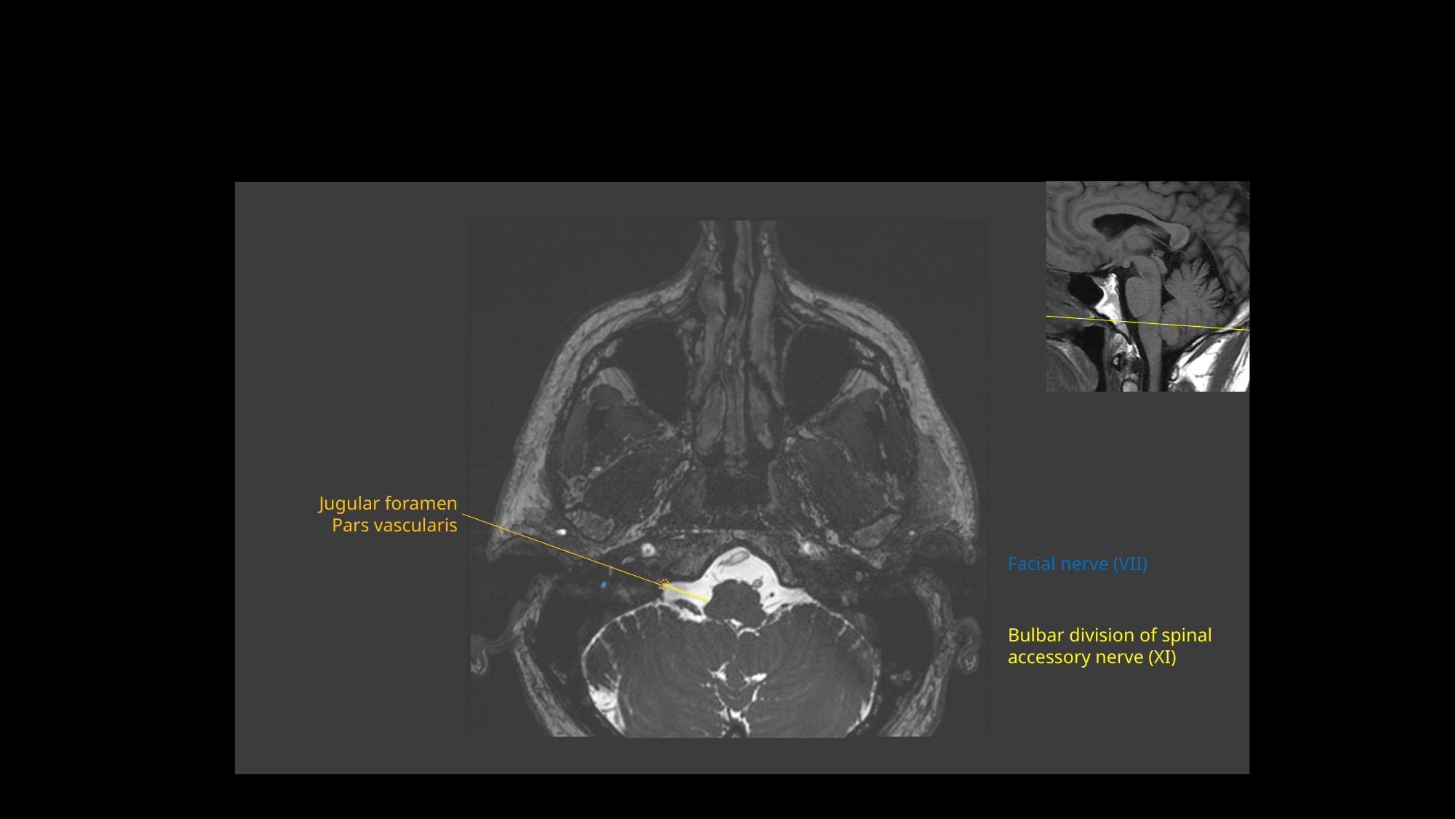

Jugular foramen
Pars vascularis
Facial nerve (VII)
Bulbar division of spinal accessory nerve (XI)

## Slide 74
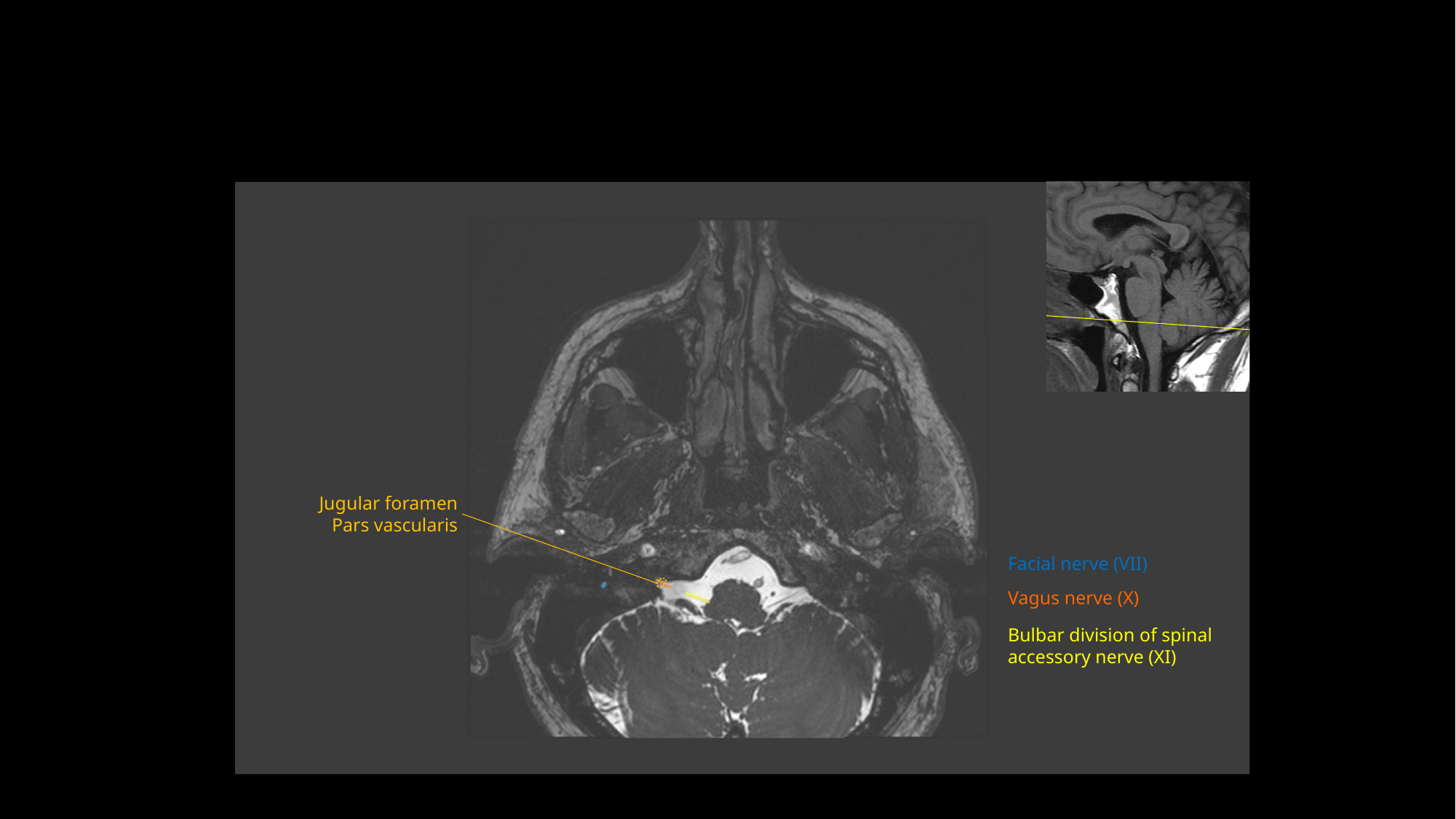

Jugular foramen
Pars vascularis
Facial nerve (VII)
Vagus nerve (X)
Bulbar division of spinal accessory nerve (XI)

## Slide 75
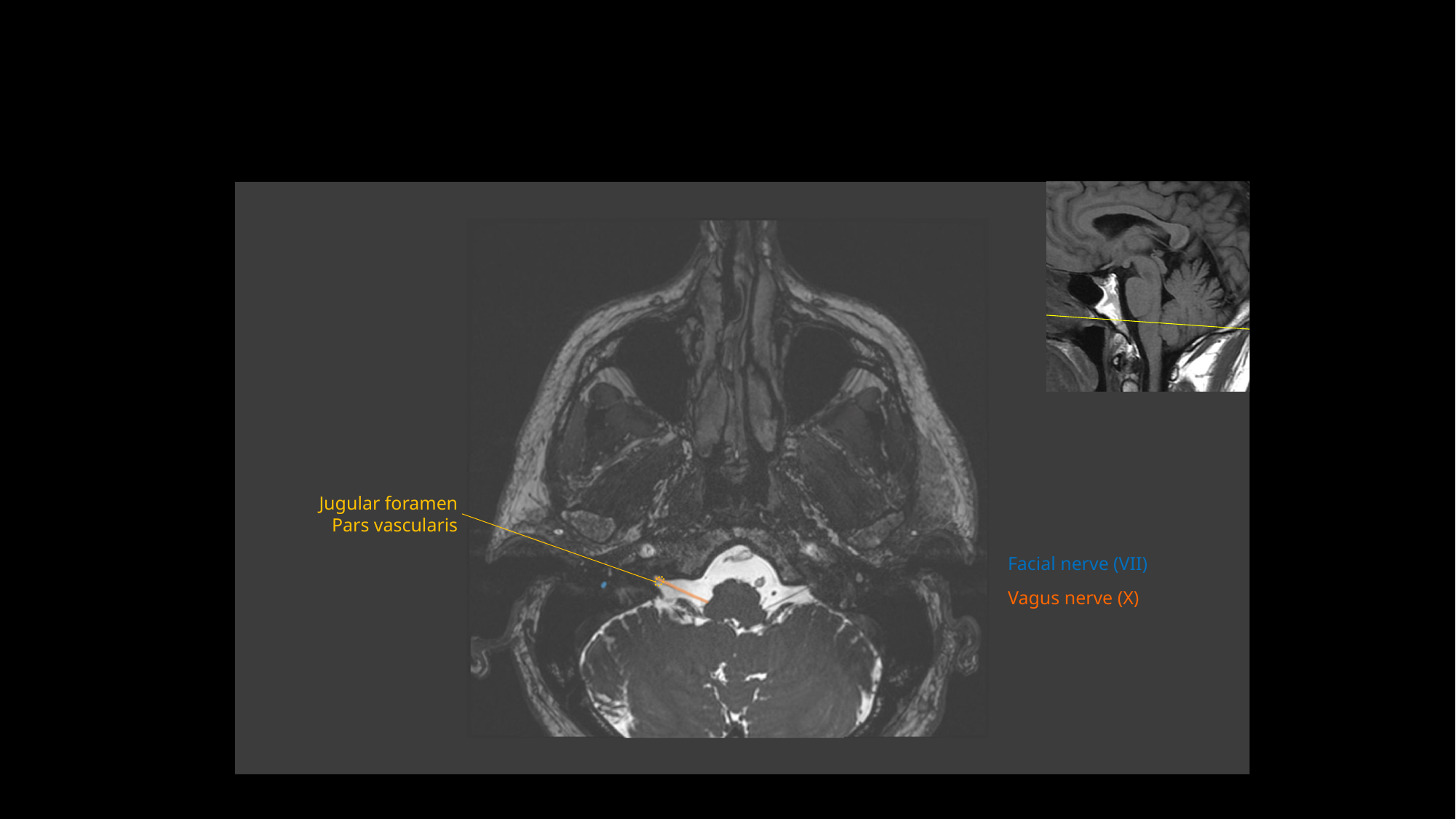

Jugular foramen
Pars vascularis
Facial nerve (VII)
Vagus nerve (X)

## Slide 76
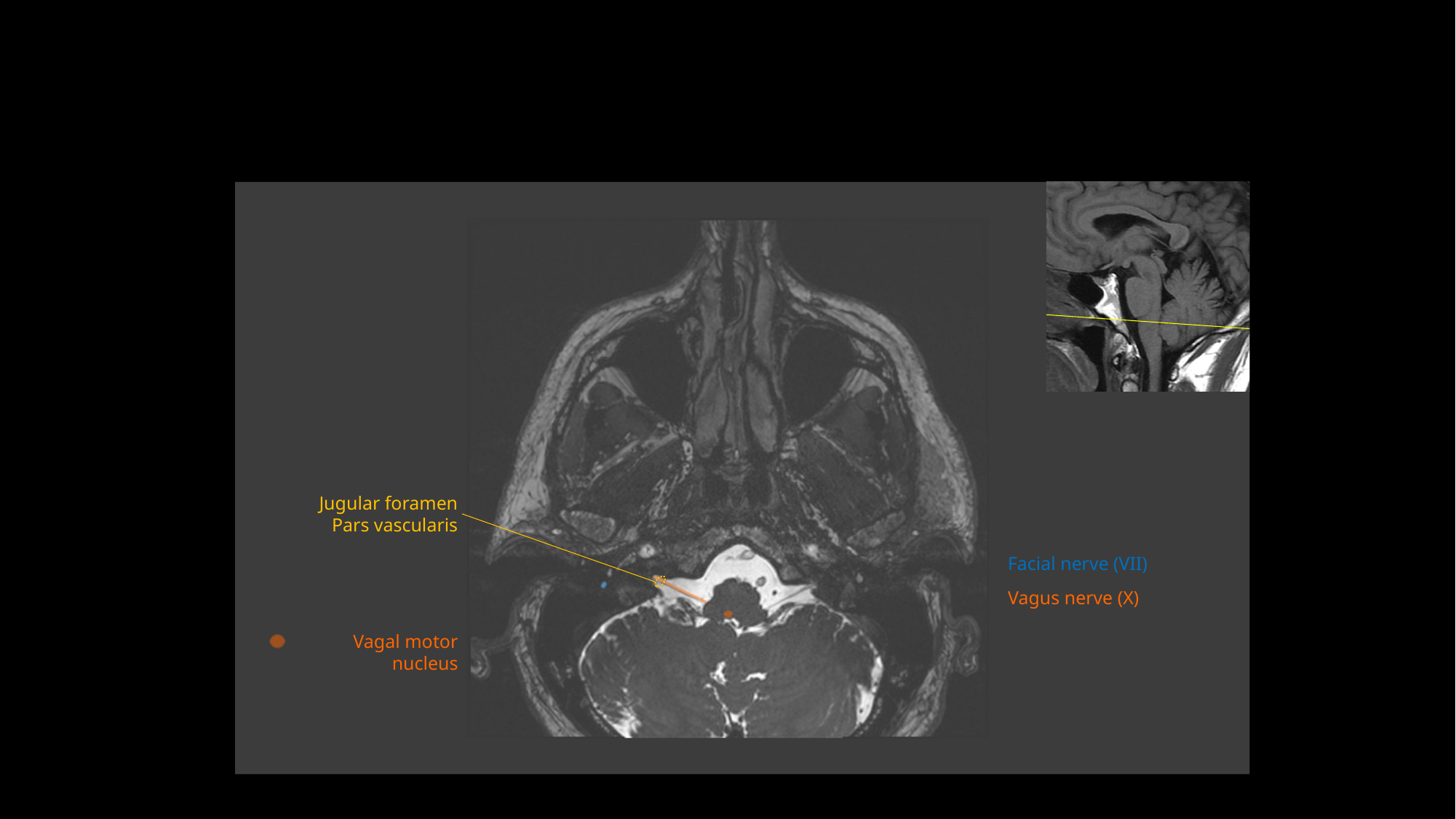

Jugular foramen
Pars vascularis
Facial nerve (VII)
Vagus nerve (X)
Vagal motor nucleus

## Slide 77
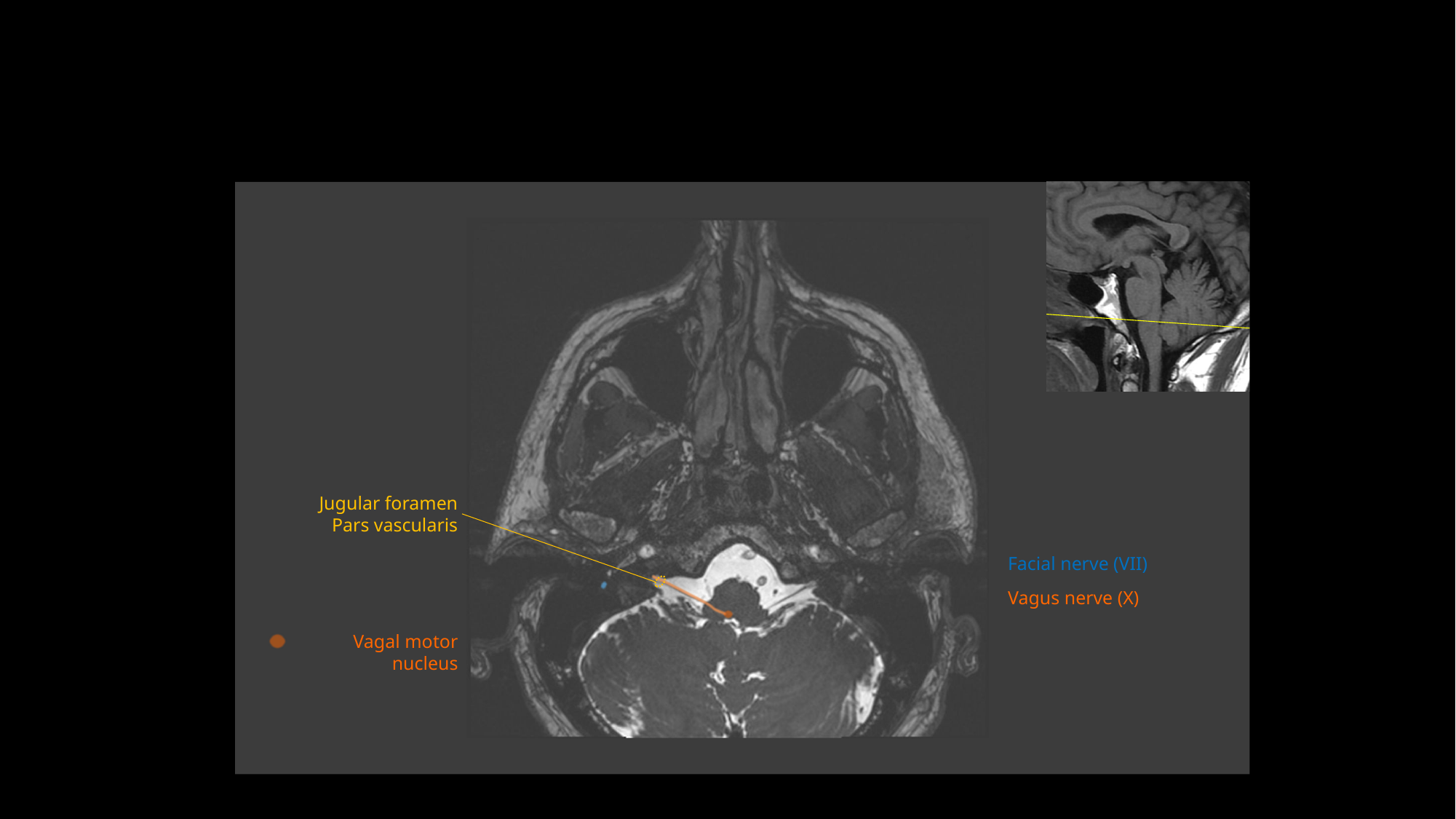

Jugular foramen
Pars vascularis
Facial nerve (VII)
Vagus nerve (X)
Vagal motor nucleus

## Slide 78
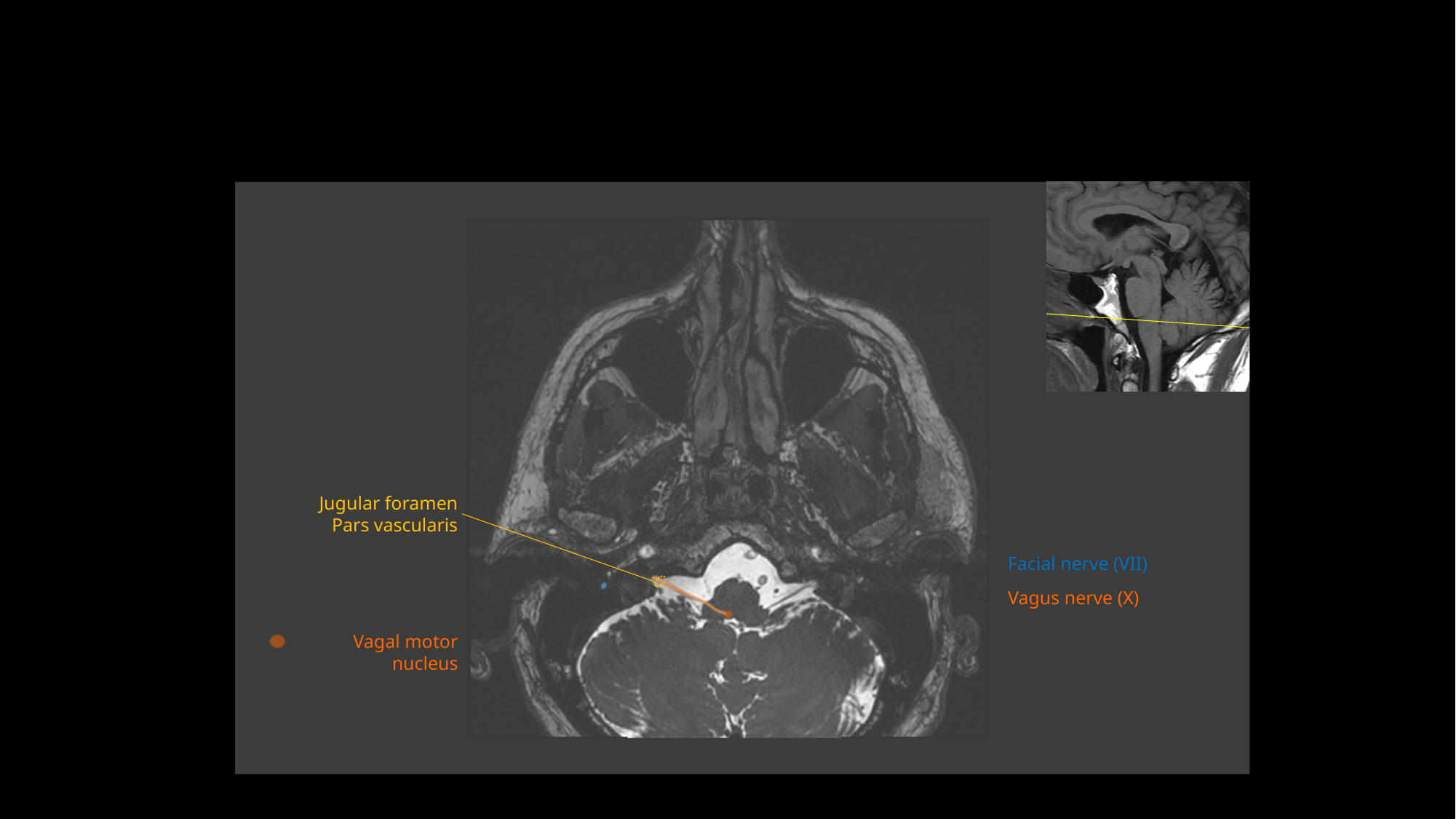

Jugular foramen
Pars vascularis
Facial nerve (VII)
Vagus nerve (X)
Vagal motor nucleus

## Slide 79
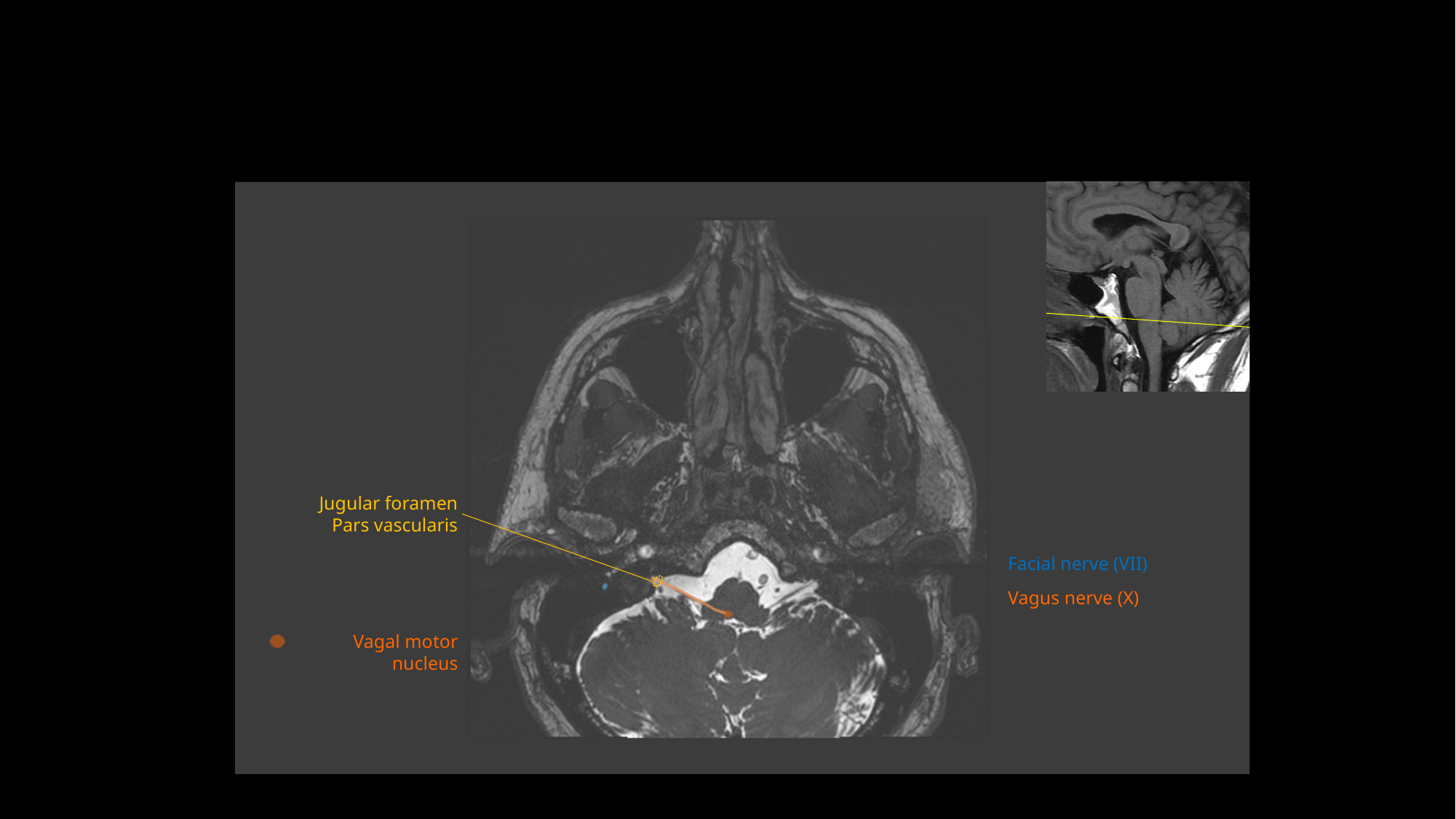

Jugular foramen
Pars vascularis
Facial nerve (VII)
Vagus nerve (X)
Vagal motor nucleus

## Slide 80
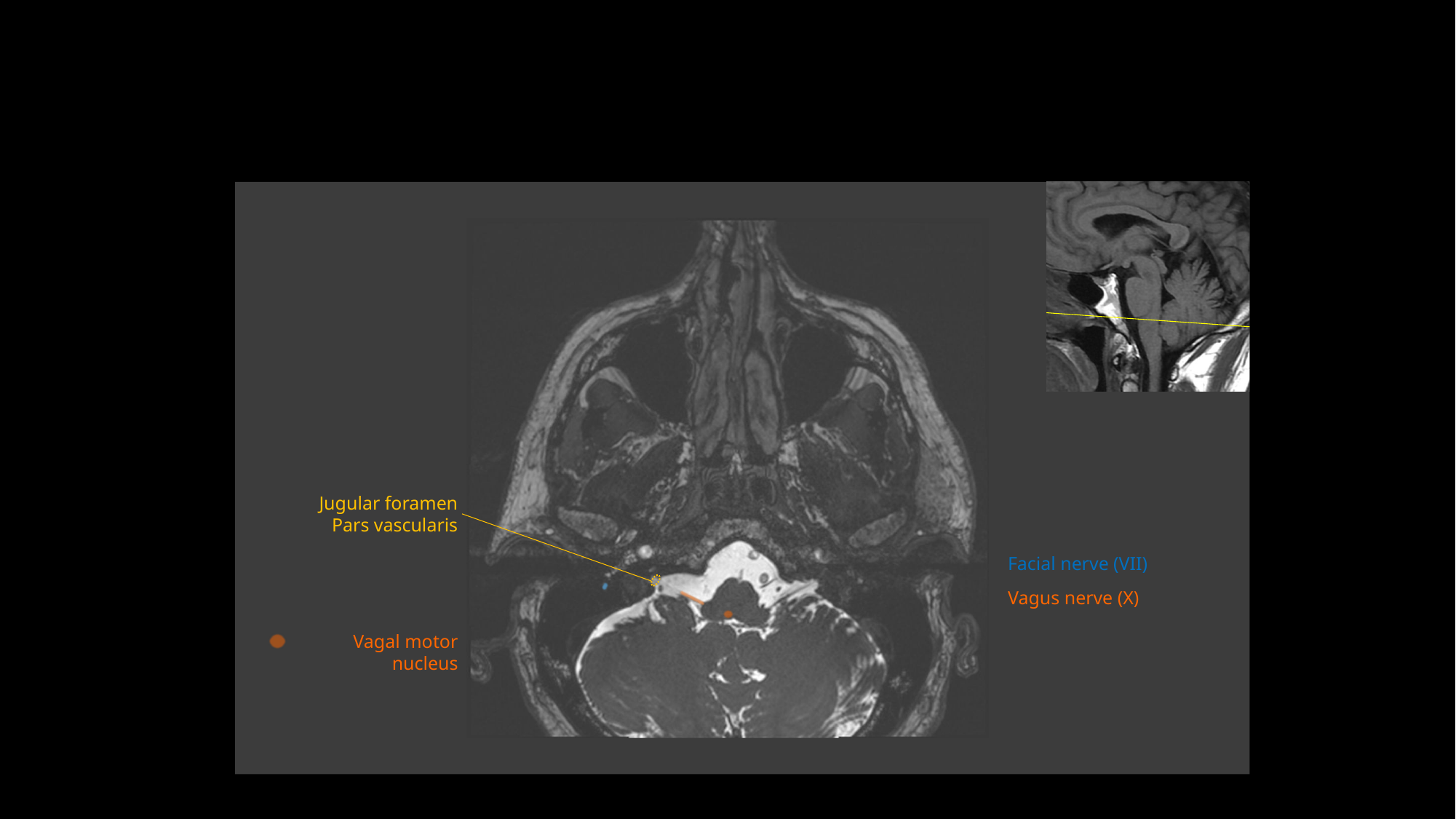

Jugular foramen
Pars vascularis
Facial nerve (VII)
Vagus nerve (X)
Vagal motor nucleus

## Slide 81
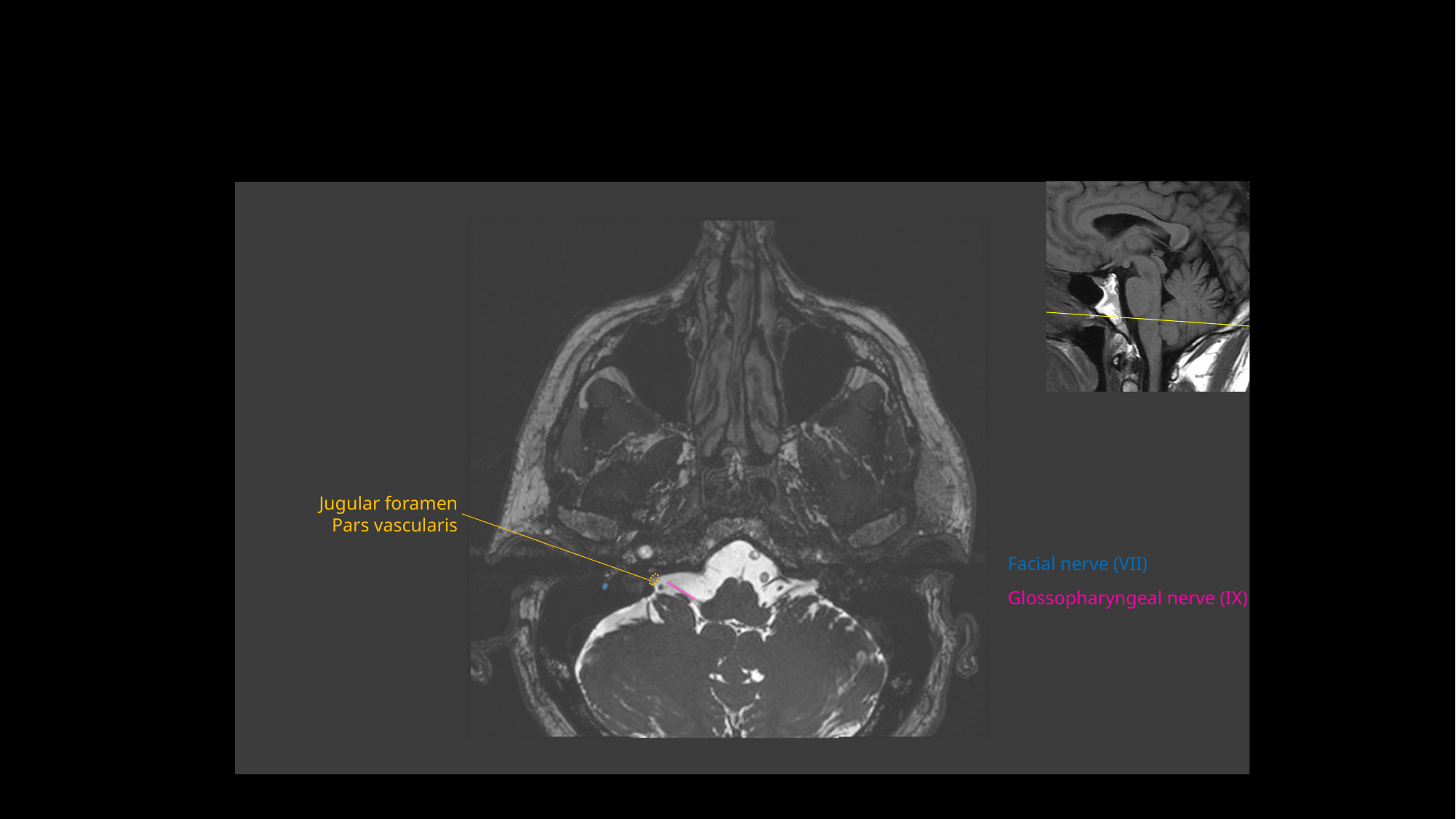

Jugular foramen
Pars vascularis
Facial nerve (VII)
Glossopharyngeal nerve (IX)

## Slide 82
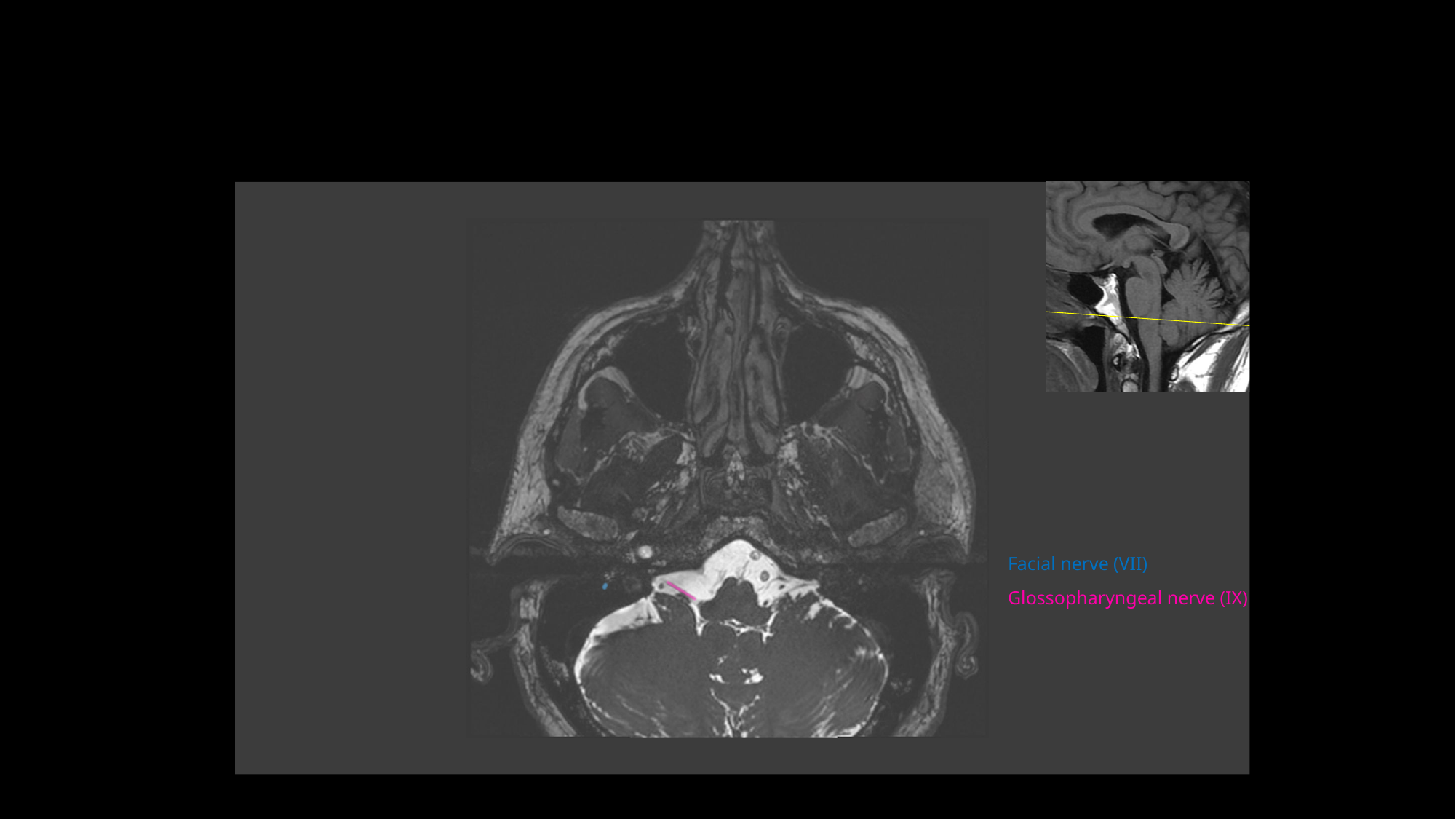

Facial nerve (VII)
Glossopharyngeal nerve (IX)

## Slide 83
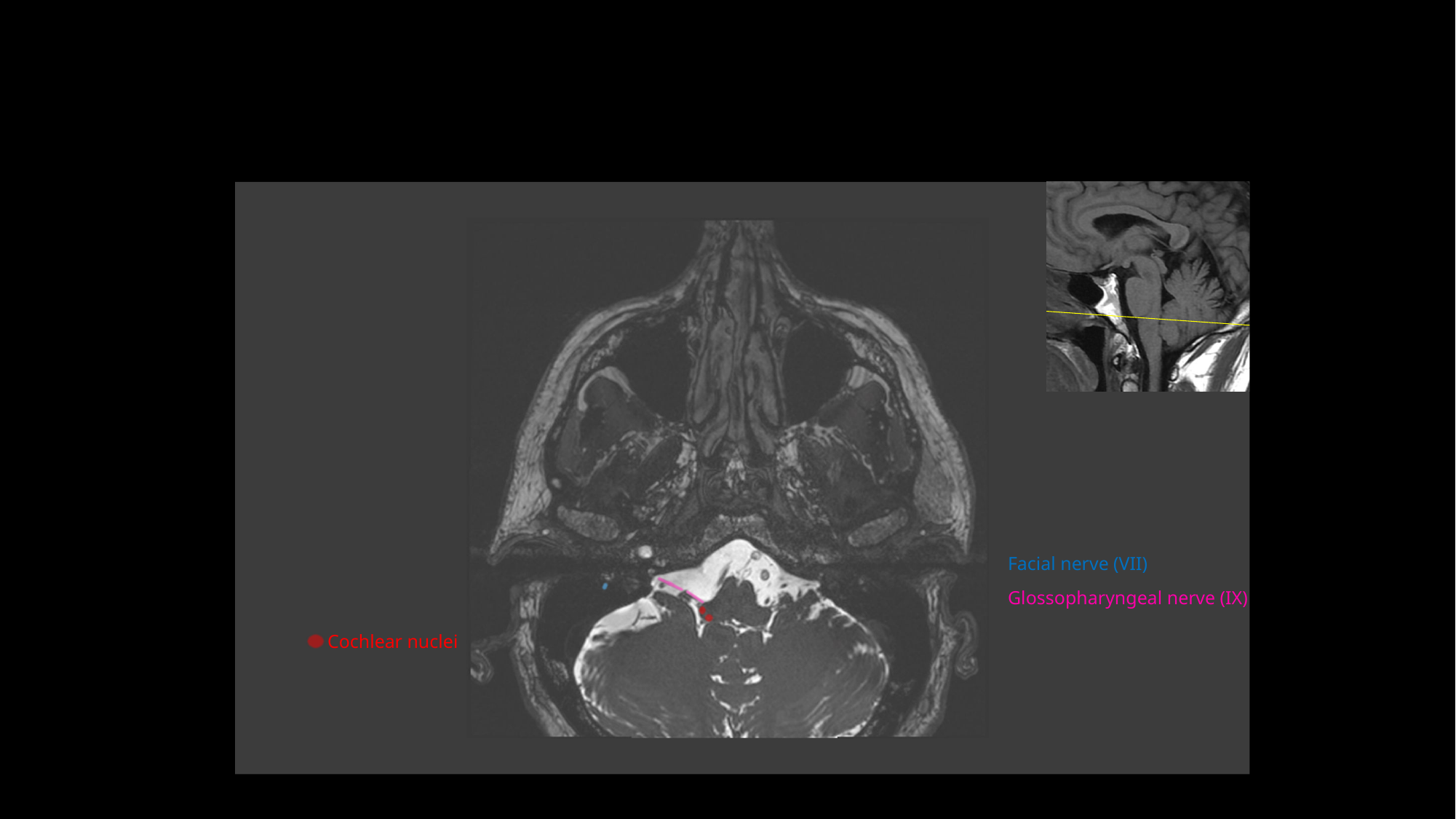

Facial nerve (VII)
Glossopharyngeal nerve (IX)
Cochlear nuclei

## Slide 84
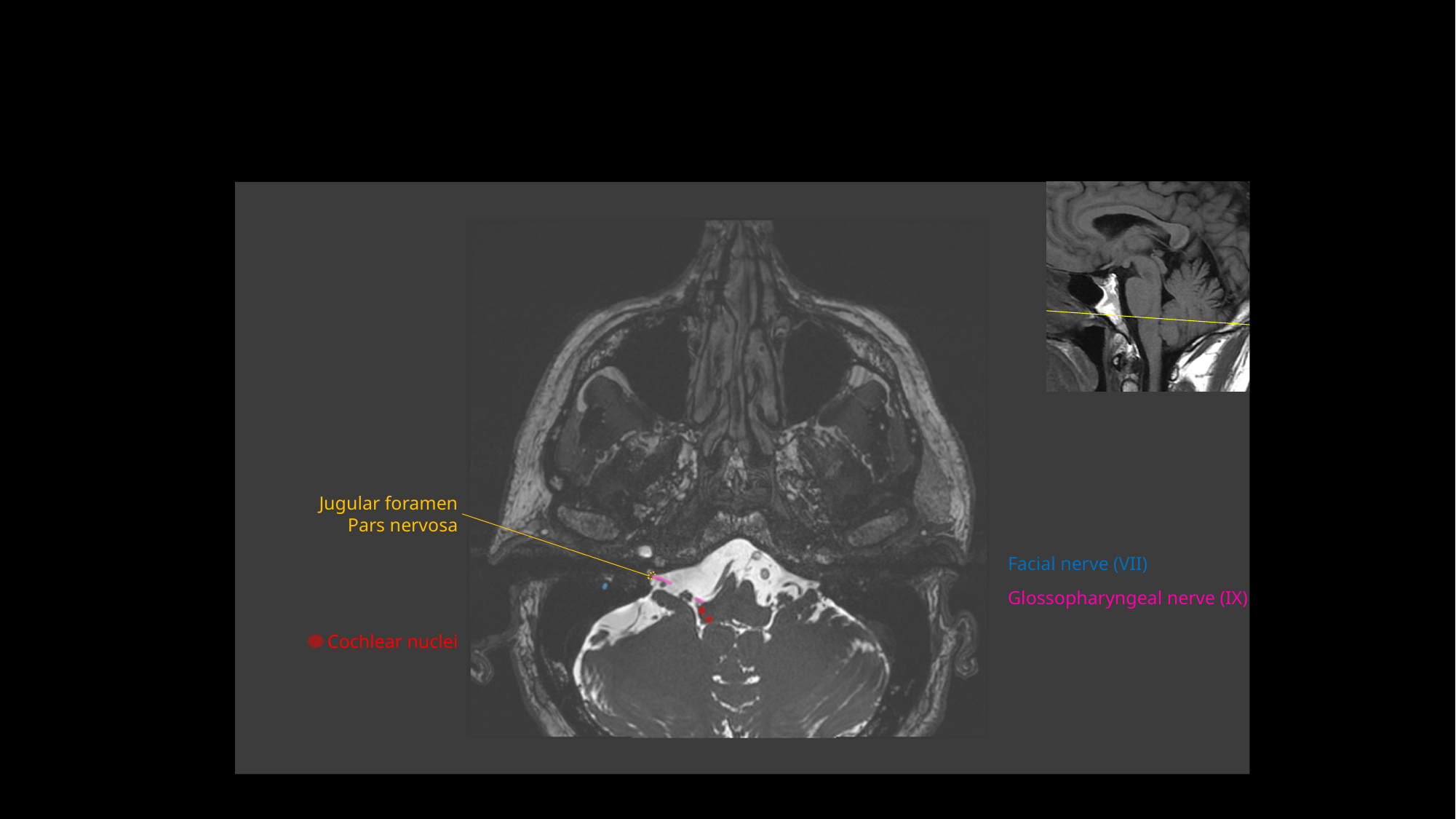

Jugular foramen
Pars nervosa
Facial nerve (VII)
Glossopharyngeal nerve (IX)
Cochlear nuclei

## Slide 85
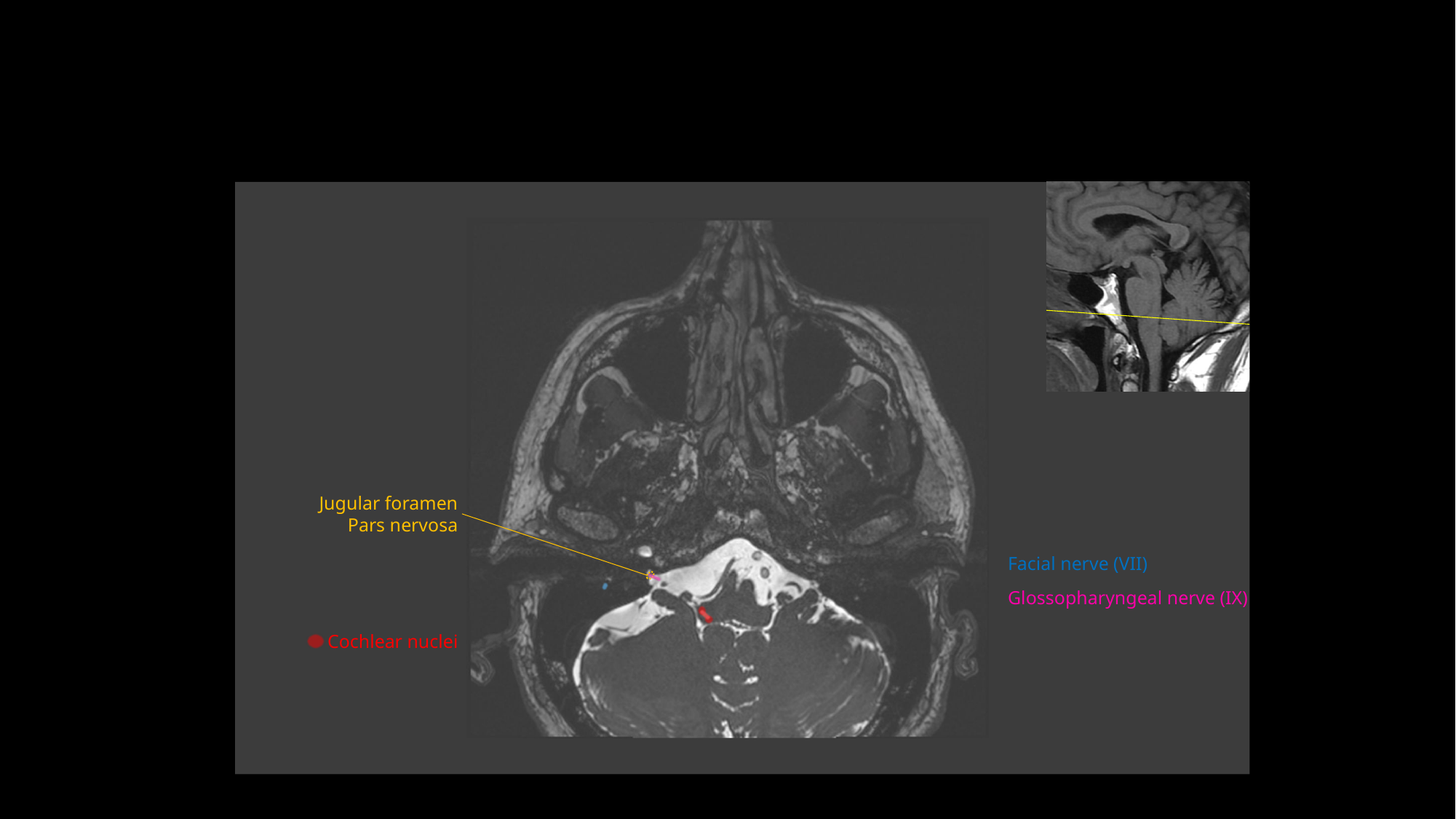

Jugular foramen
Pars nervosa
Facial nerve (VII)
Glossopharyngeal nerve (IX)
Cochlear nuclei

## Slide 86
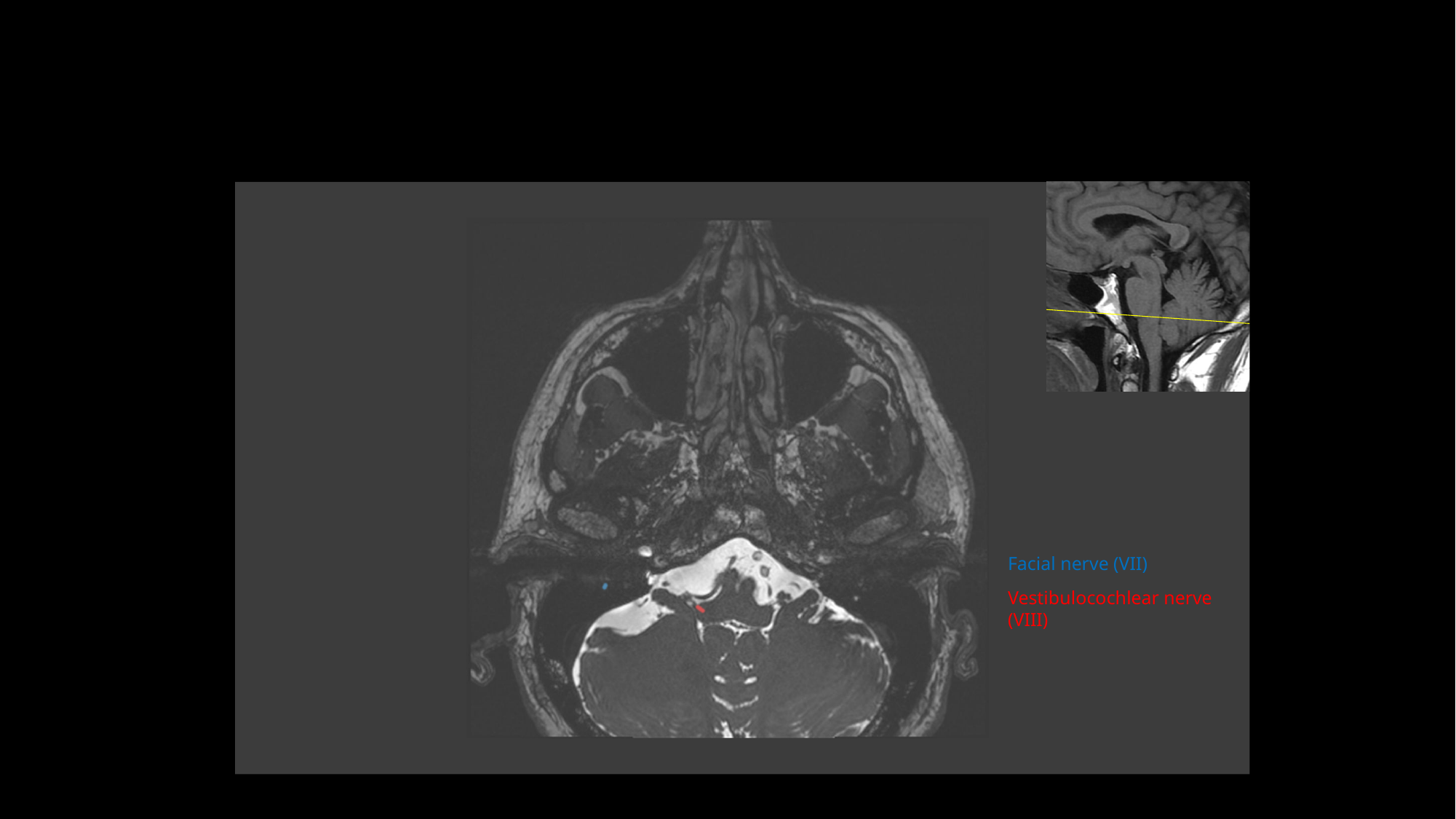

Facial nerve (VII)
Vestibulocochlear nerve (VIII)

## Slide 87
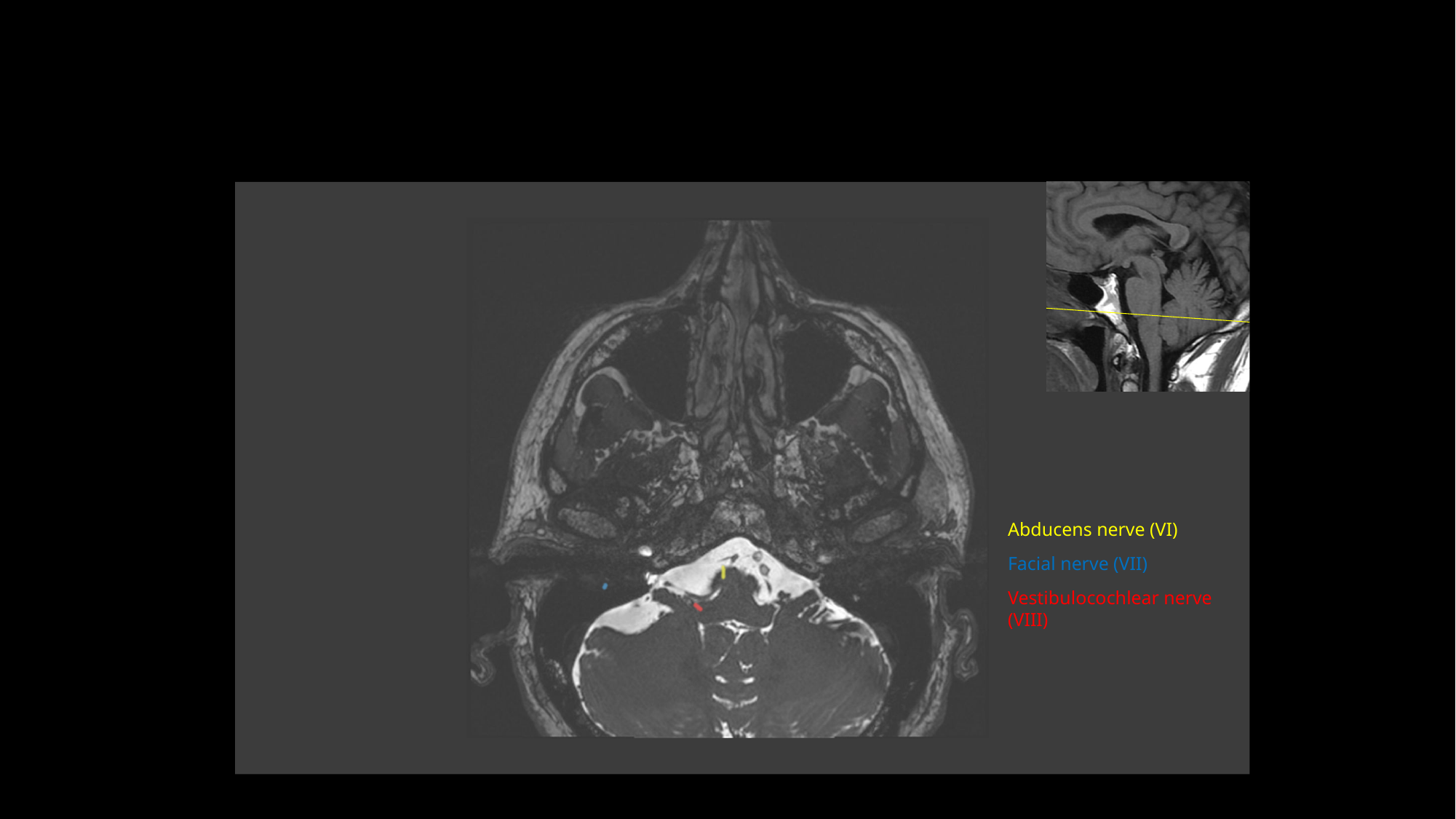

Abducens nerve (VI)
Facial nerve (VII)
Vestibulocochlear nerve (VIII)

## Slide 88
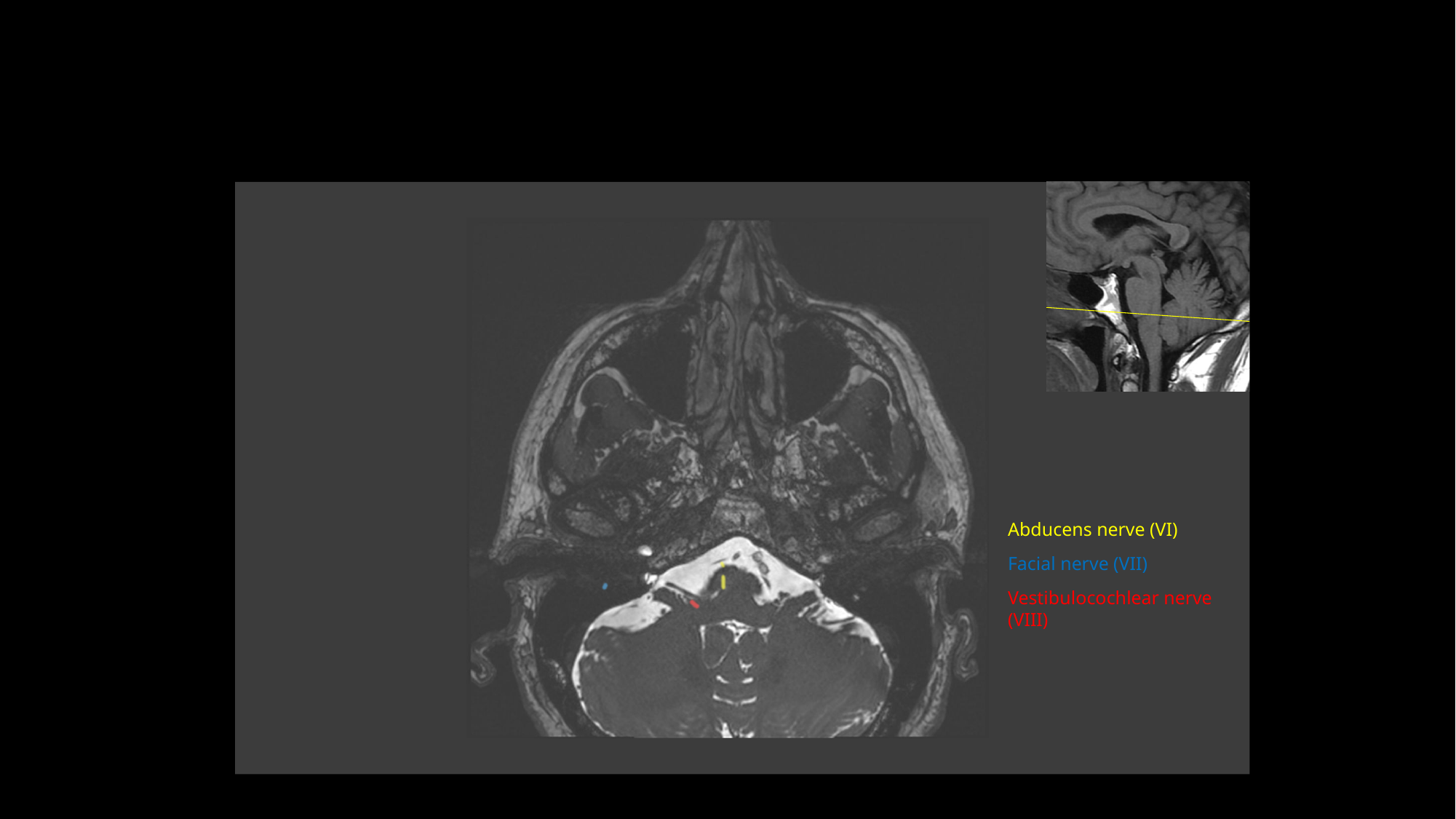

Abducens nerve (VI)
Facial nerve (VII)
Vestibulocochlear nerve (VIII)

## Slide 89
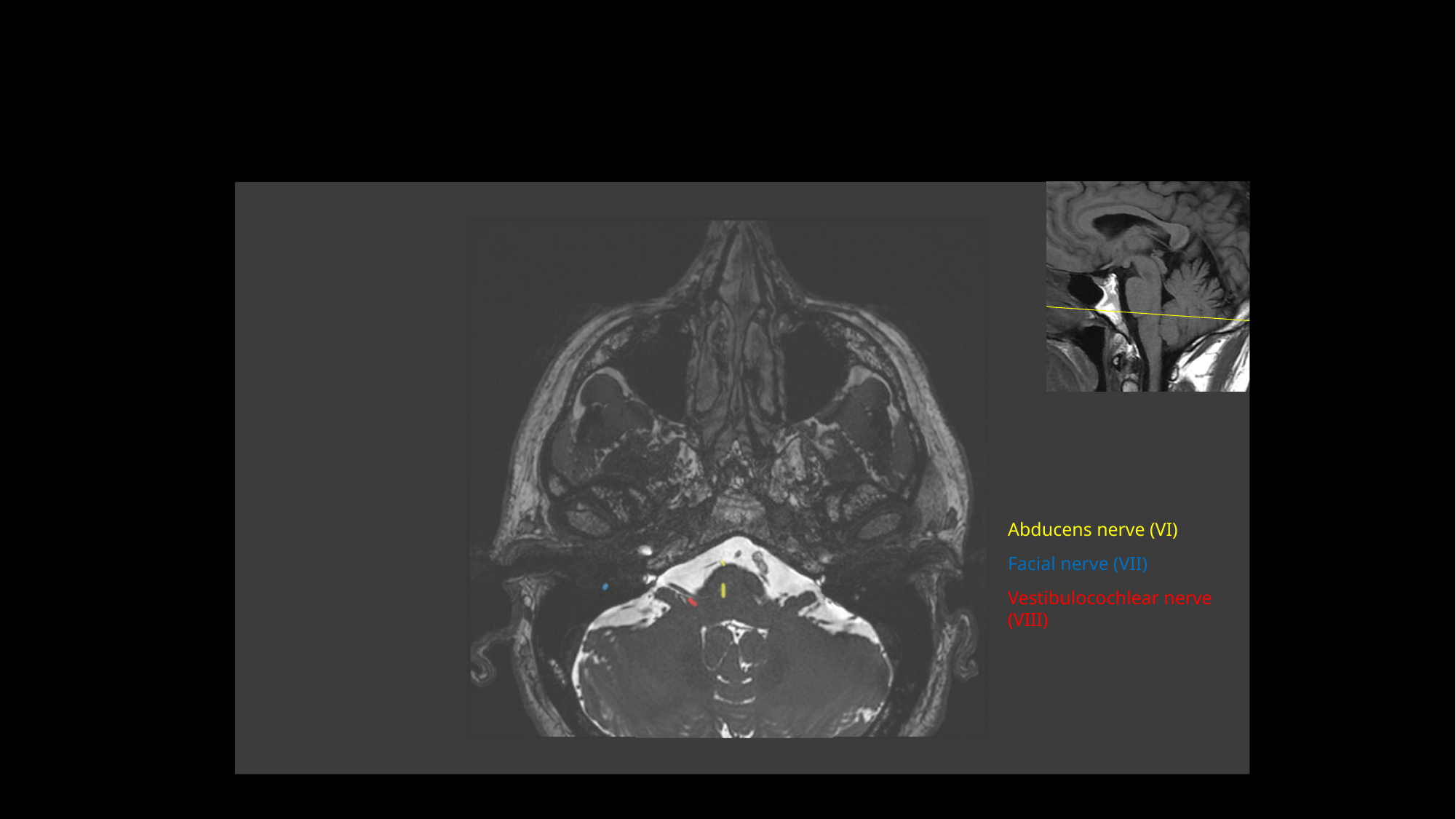

Abducens nerve (VI)
Facial nerve (VII)
Vestibulocochlear nerve (VIII)

## Slide 90
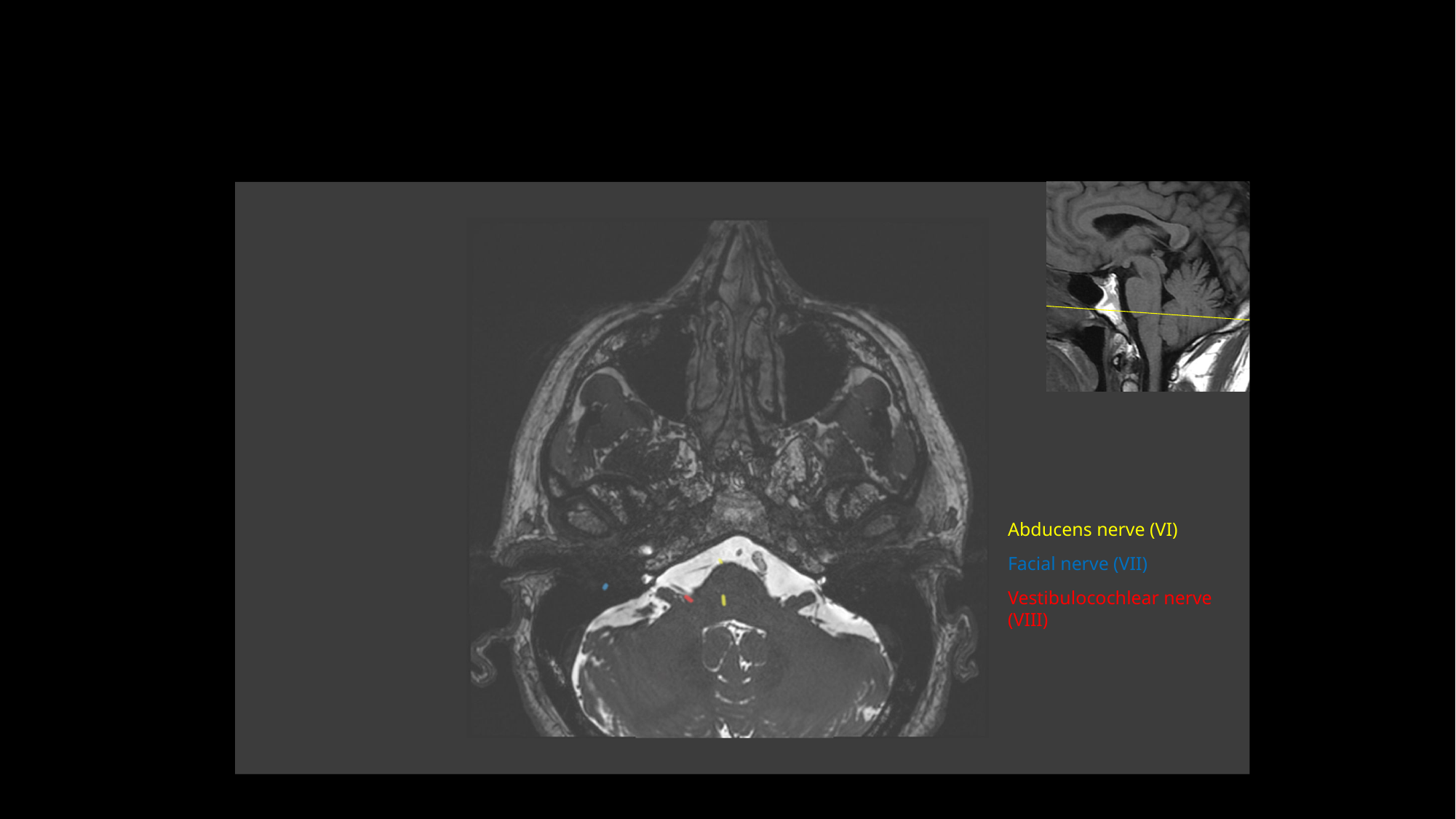

Abducens nerve (VI)
Facial nerve (VII)
Vestibulocochlear nerve (VIII)

## Slide 91
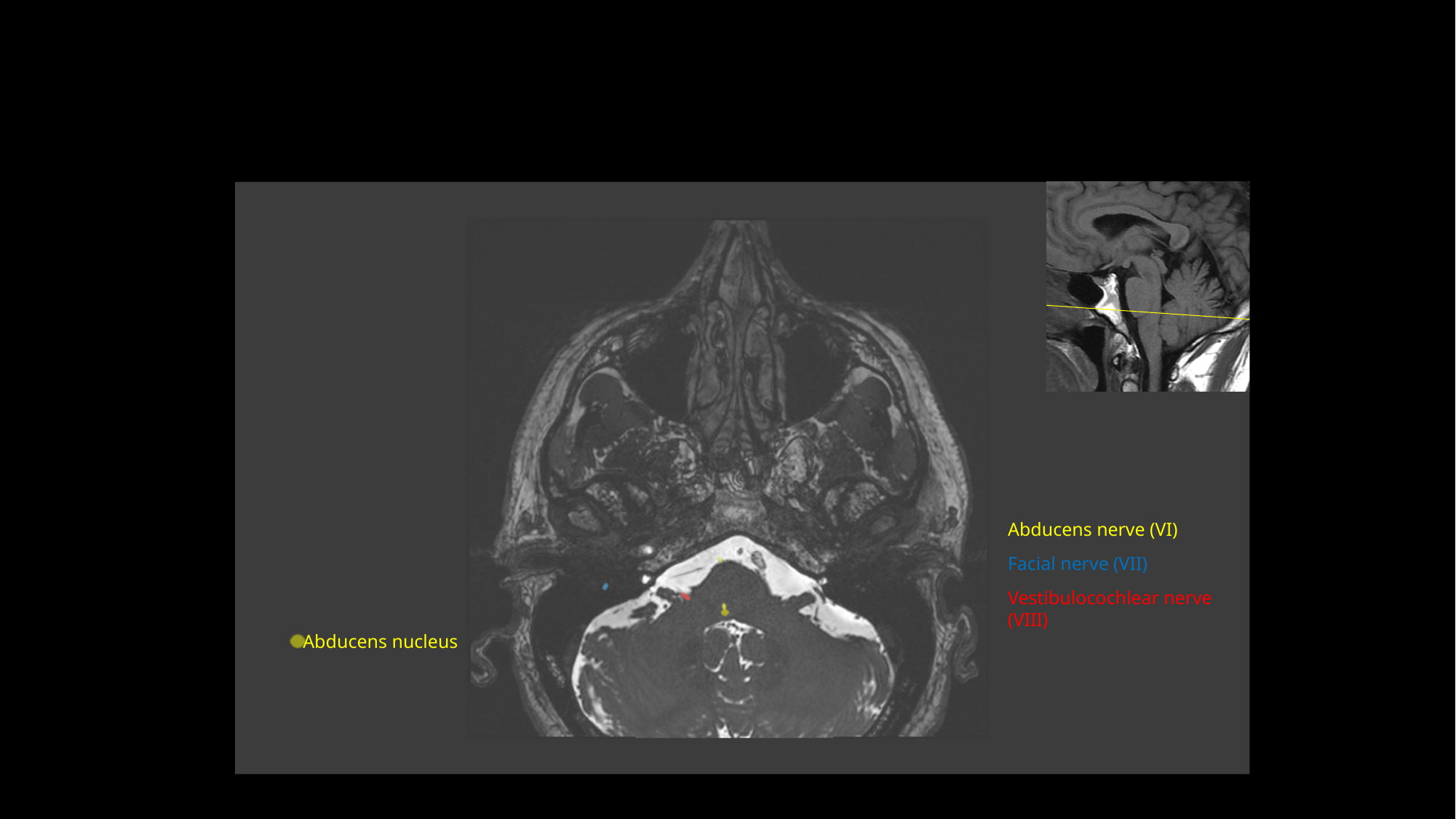

Abducens nerve (VI)
Facial nerve (VII)
Vestibulocochlear nerve (VIII)
Abducens nucleus

## Slide 92
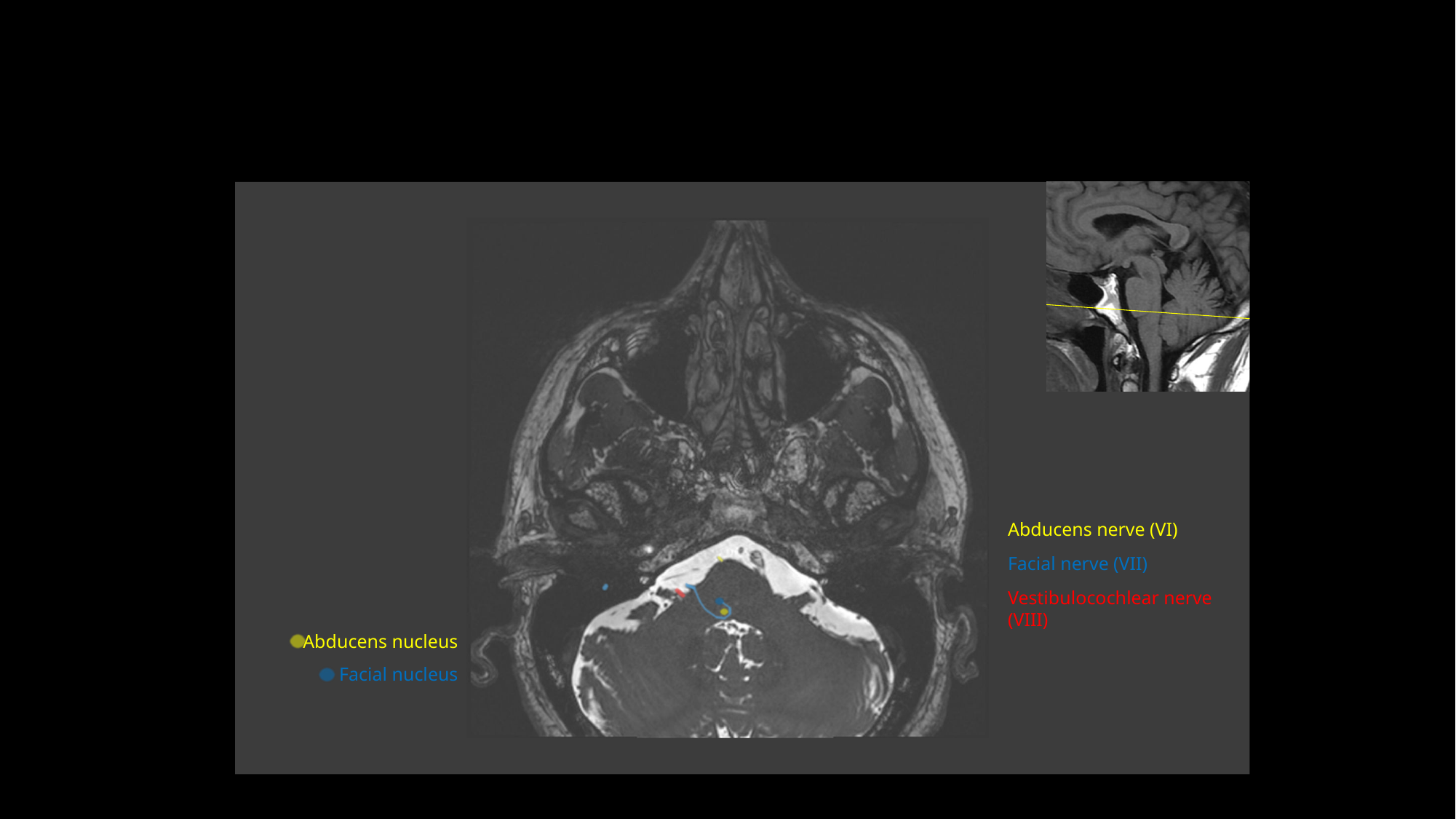

Abducens nerve (VI)
Facial nerve (VII)
Vestibulocochlear nerve (VIII)
Abducens nucleus
Facial nucleus

## Slide 93
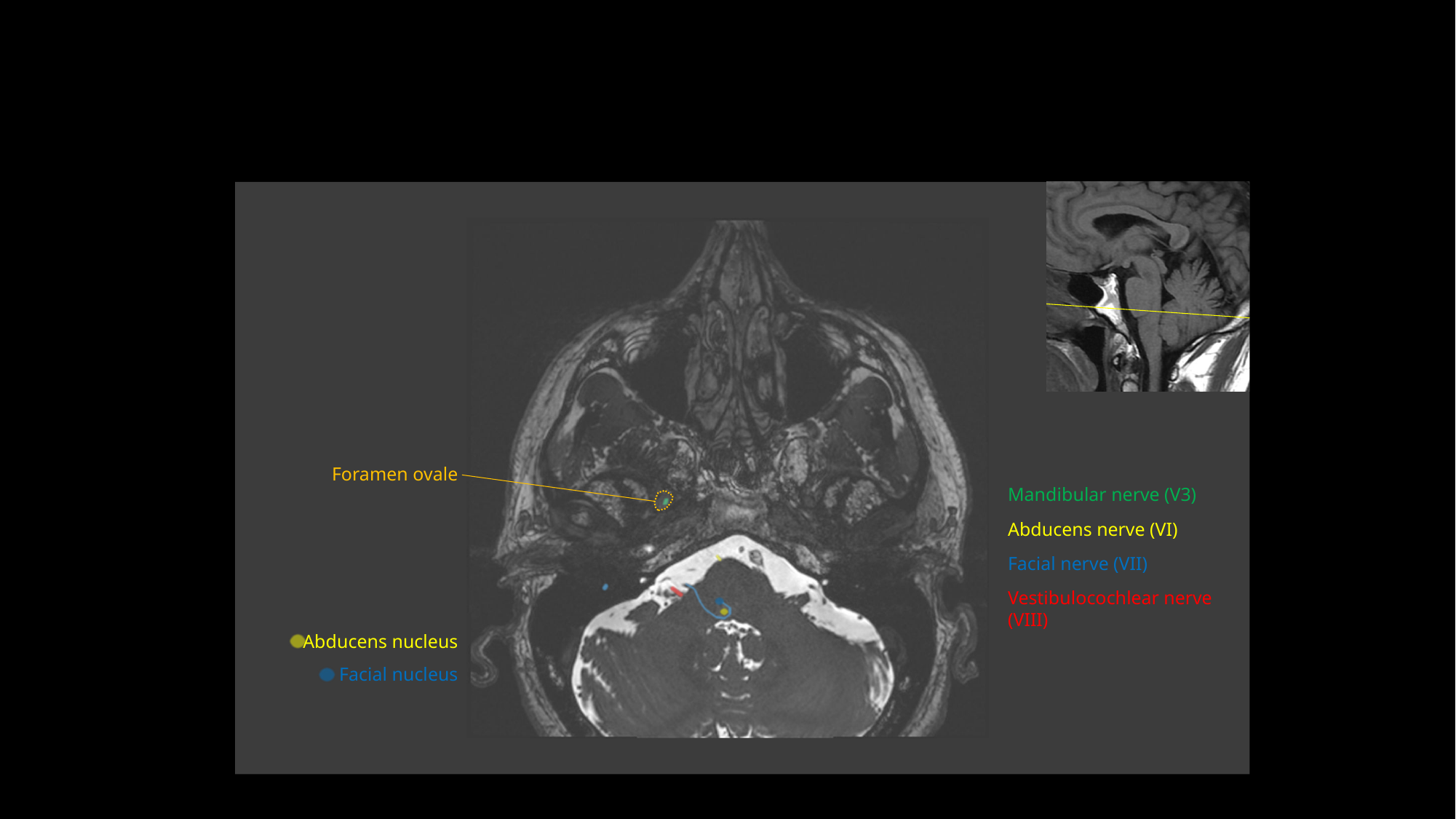

Foramen ovale
Mandibular nerve (V3)
Abducens nerve (VI)
Facial nerve (VII)
Vestibulocochlear nerve (VIII)
Abducens nucleus
Facial nucleus

## Slide 94
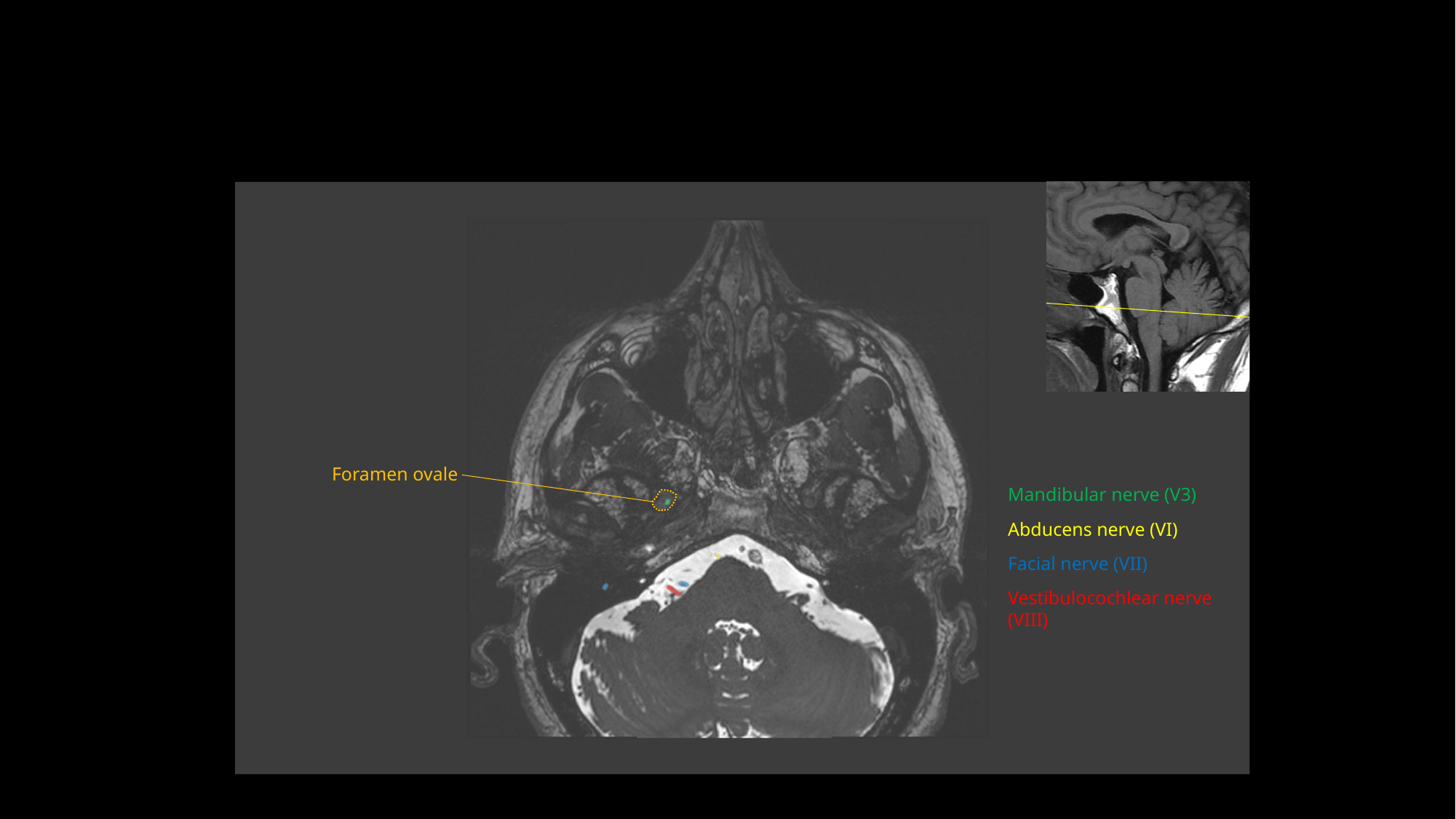

Foramen ovale
Mandibular nerve (V3)
Abducens nerve (VI)
Facial nerve (VII)
Vestibulocochlear nerve (VIII)

## Slide 95
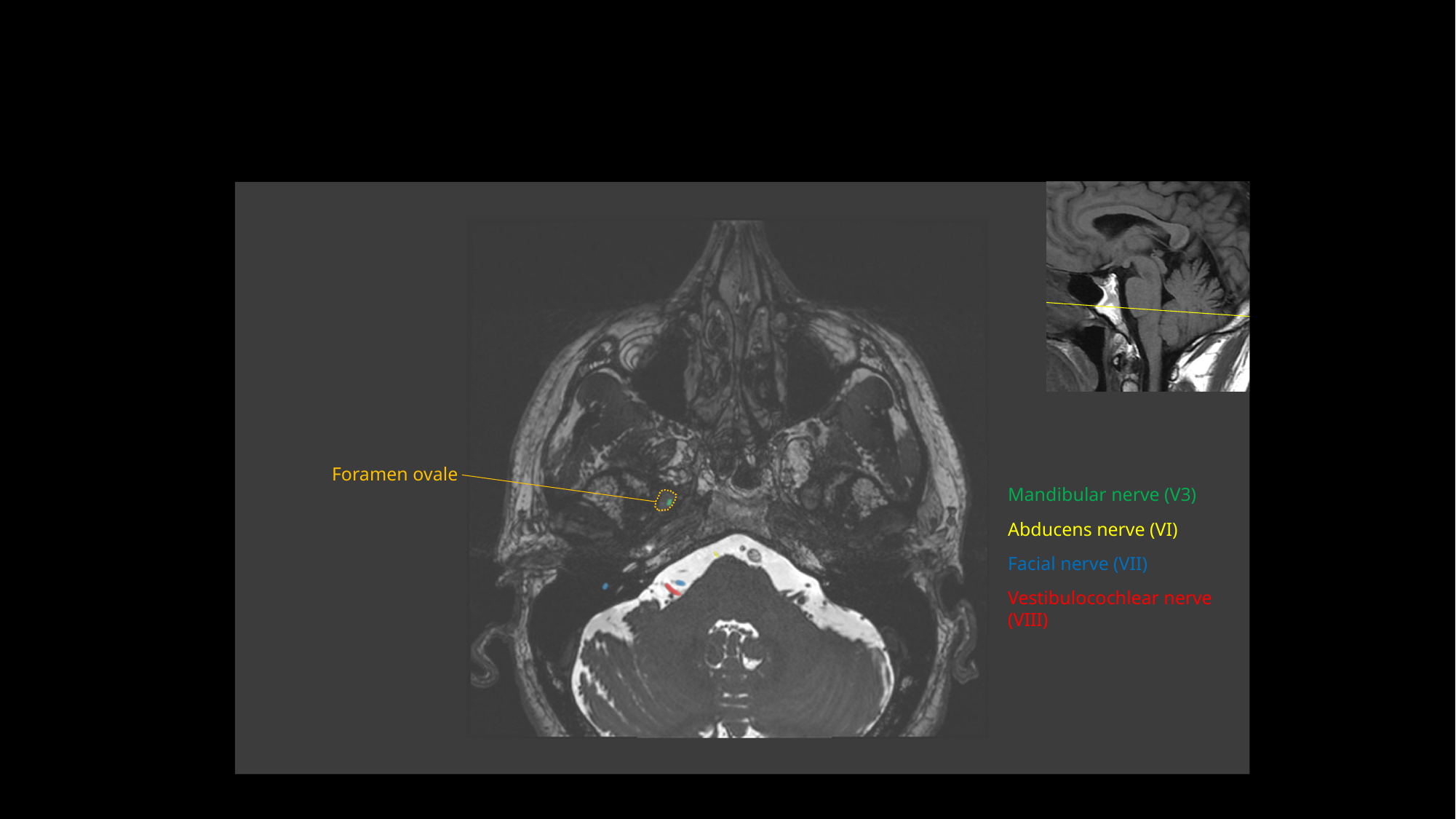

Foramen ovale
Mandibular nerve (V3)
Abducens nerve (VI)
Facial nerve (VII)
Vestibulocochlear nerve (VIII)

## Slide 96
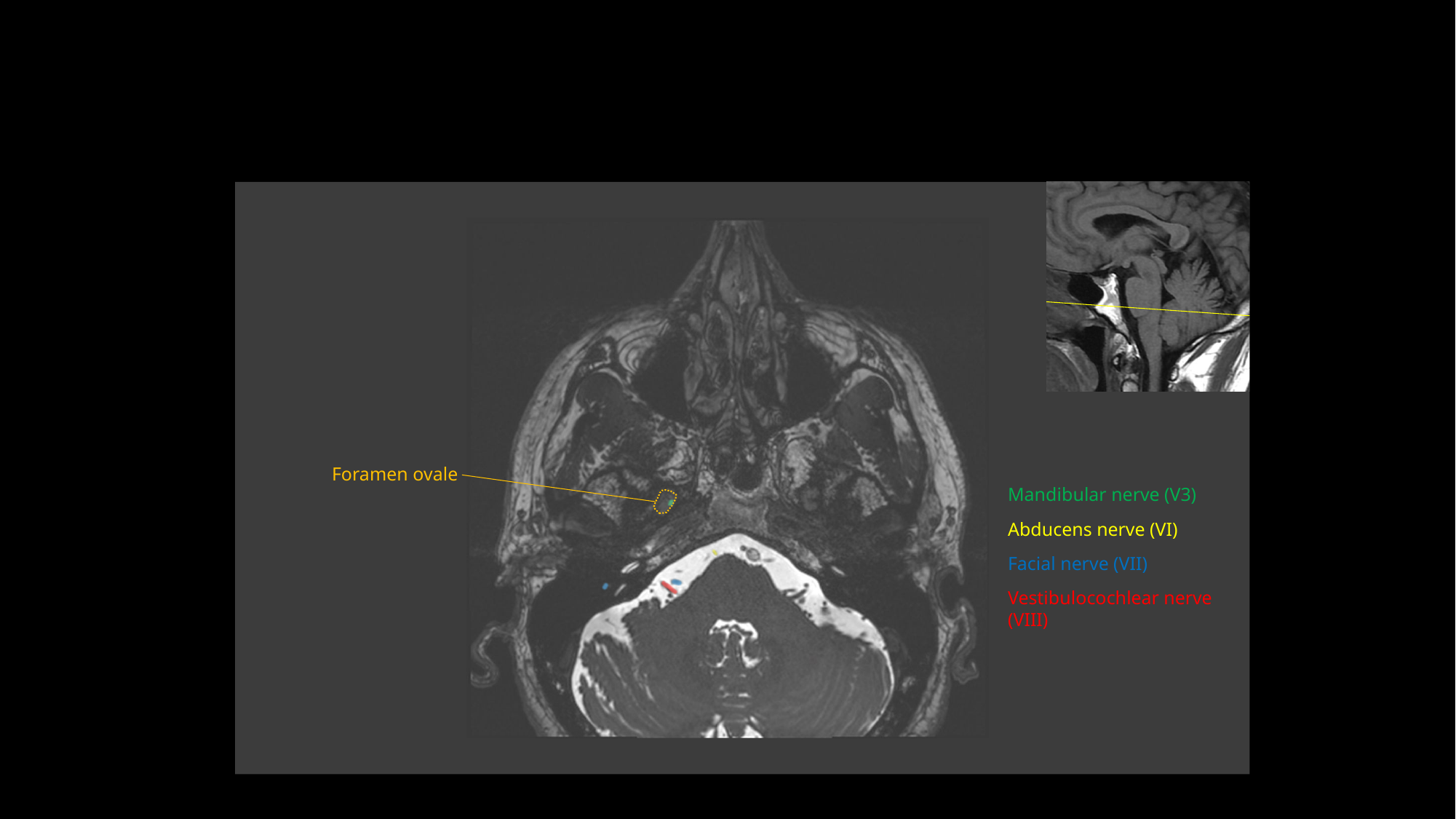

Foramen ovale
Mandibular nerve (V3)
Abducens nerve (VI)
Facial nerve (VII)
Vestibulocochlear nerve (VIII)

## Slide 97
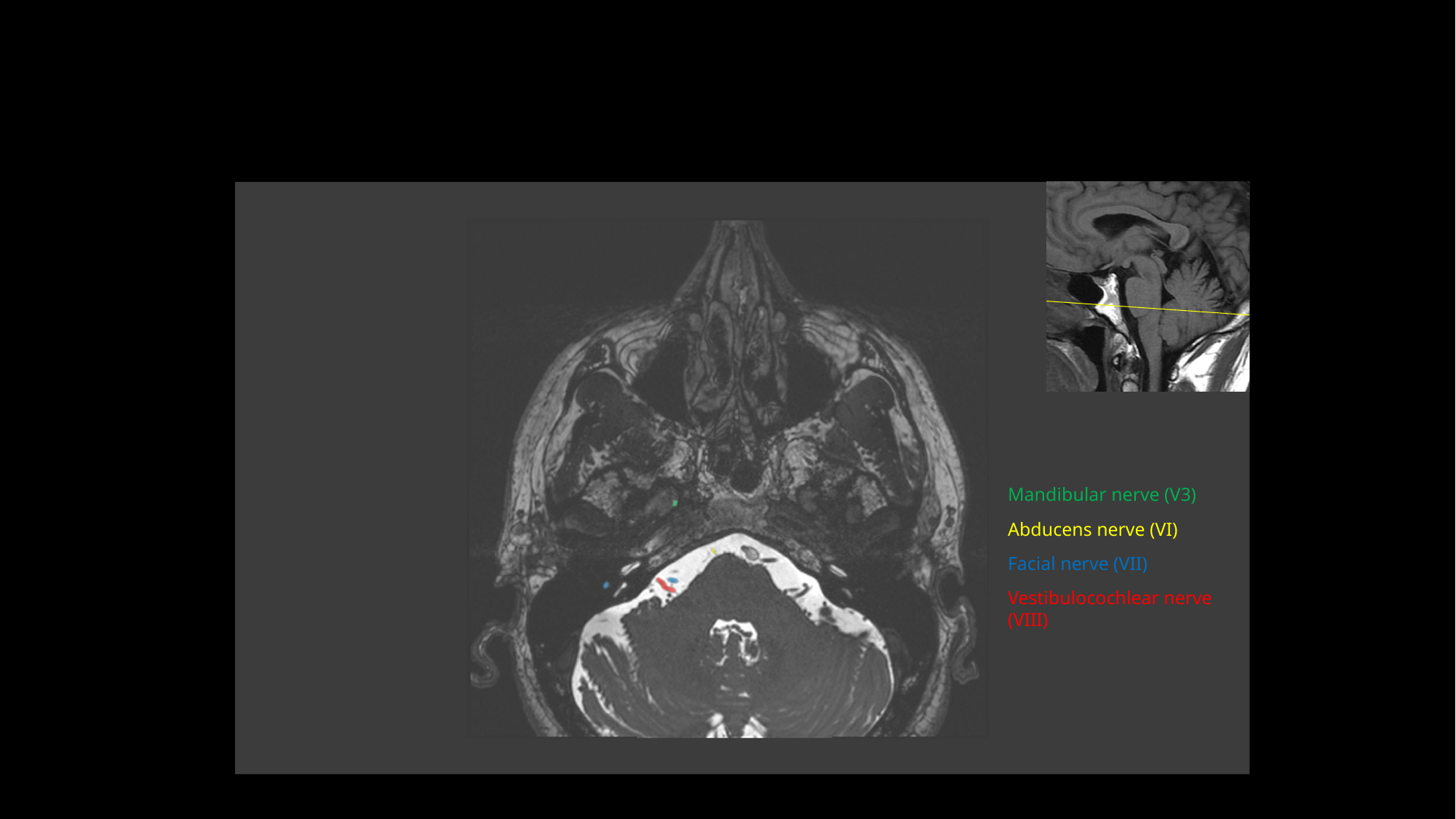

Mandibular nerve (V3)
Abducens nerve (VI)
Facial nerve (VII)
Vestibulocochlear nerve (VIII)

## Slide 98
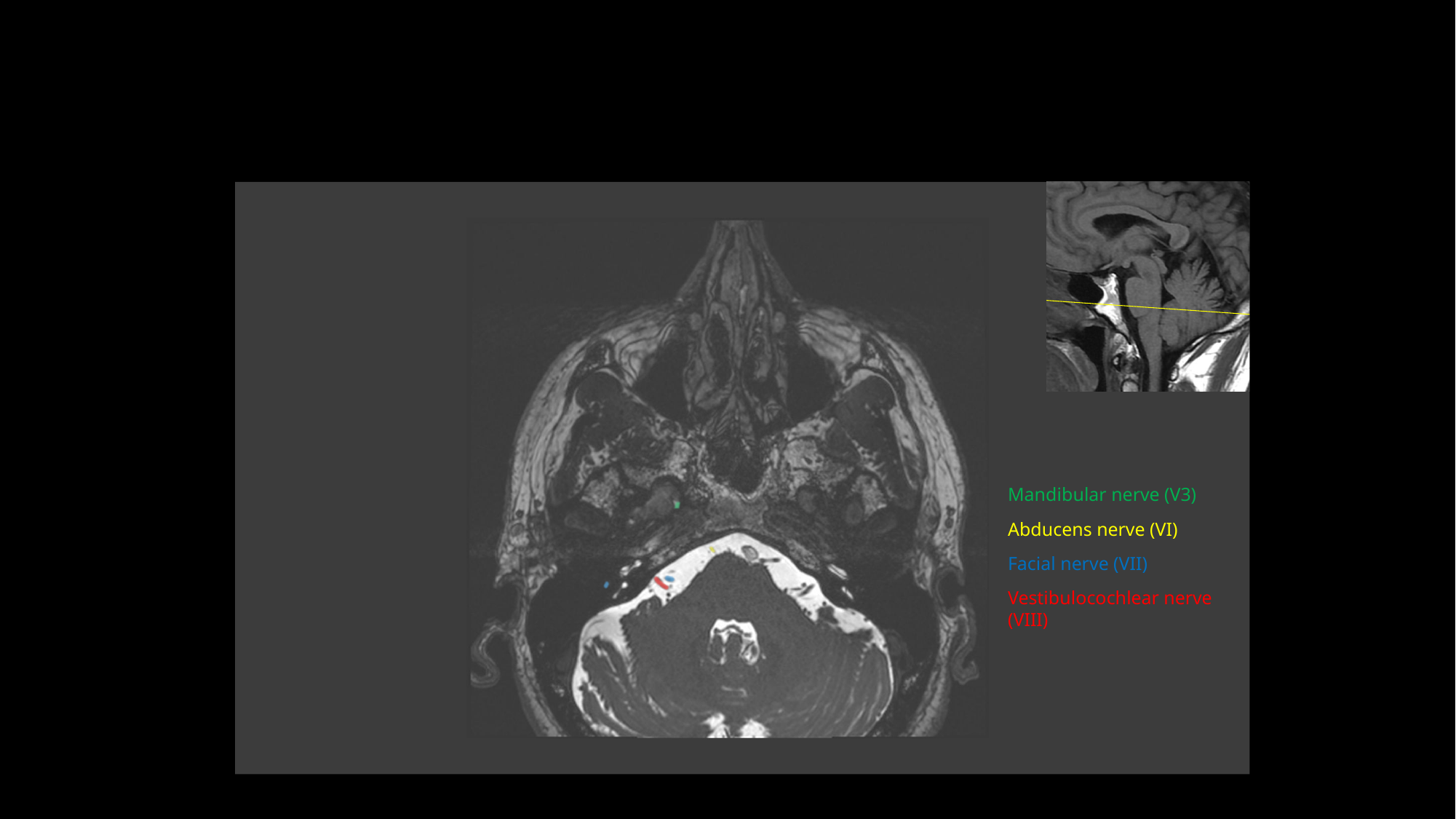

Mandibular nerve (V3)
Abducens nerve (VI)
Facial nerve (VII)
Vestibulocochlear nerve (VIII)

## Slide 99
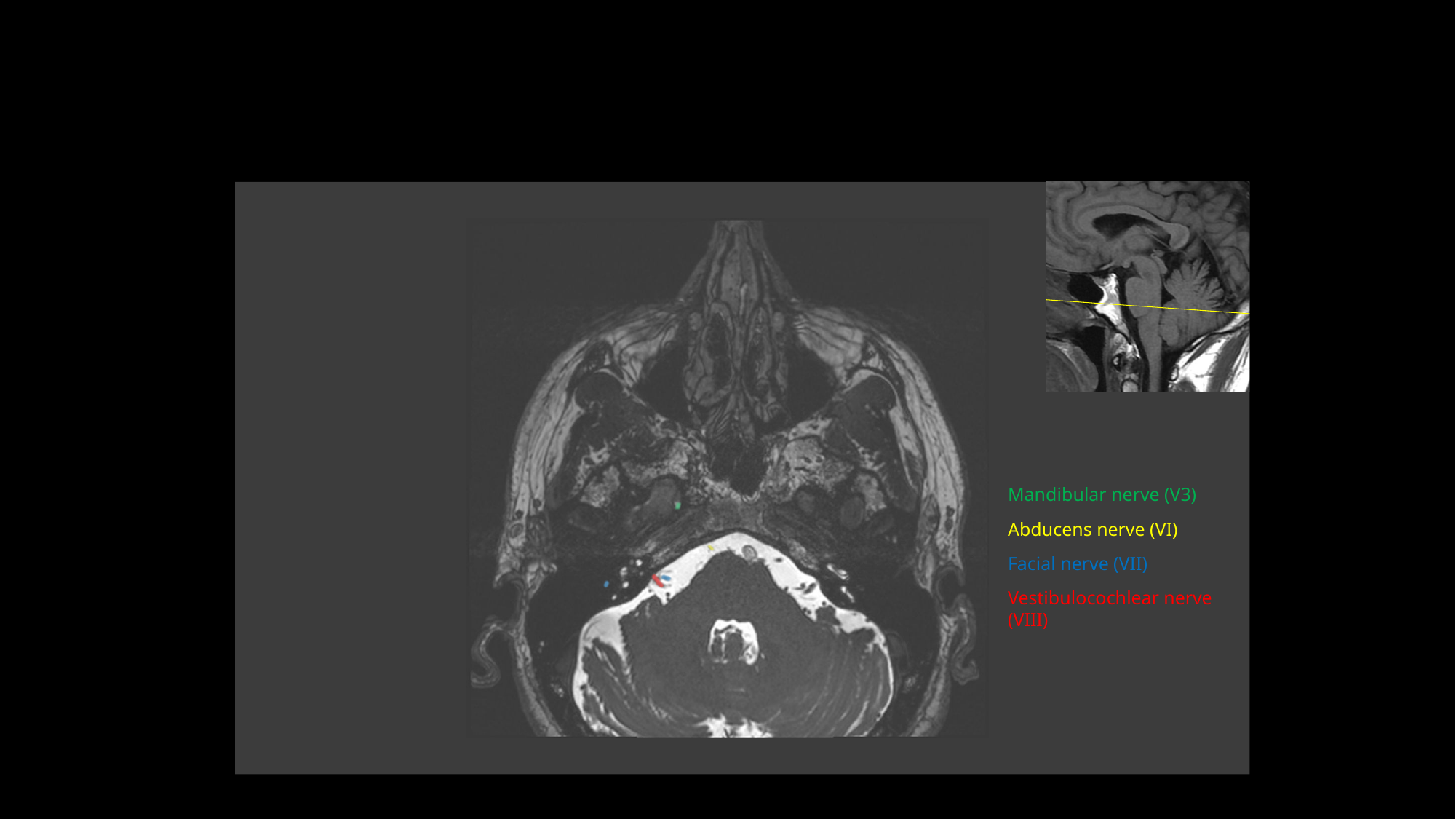

Mandibular nerve (V3)
Abducens nerve (VI)
Facial nerve (VII)
Vestibulocochlear nerve (VIII)

## Slide 100
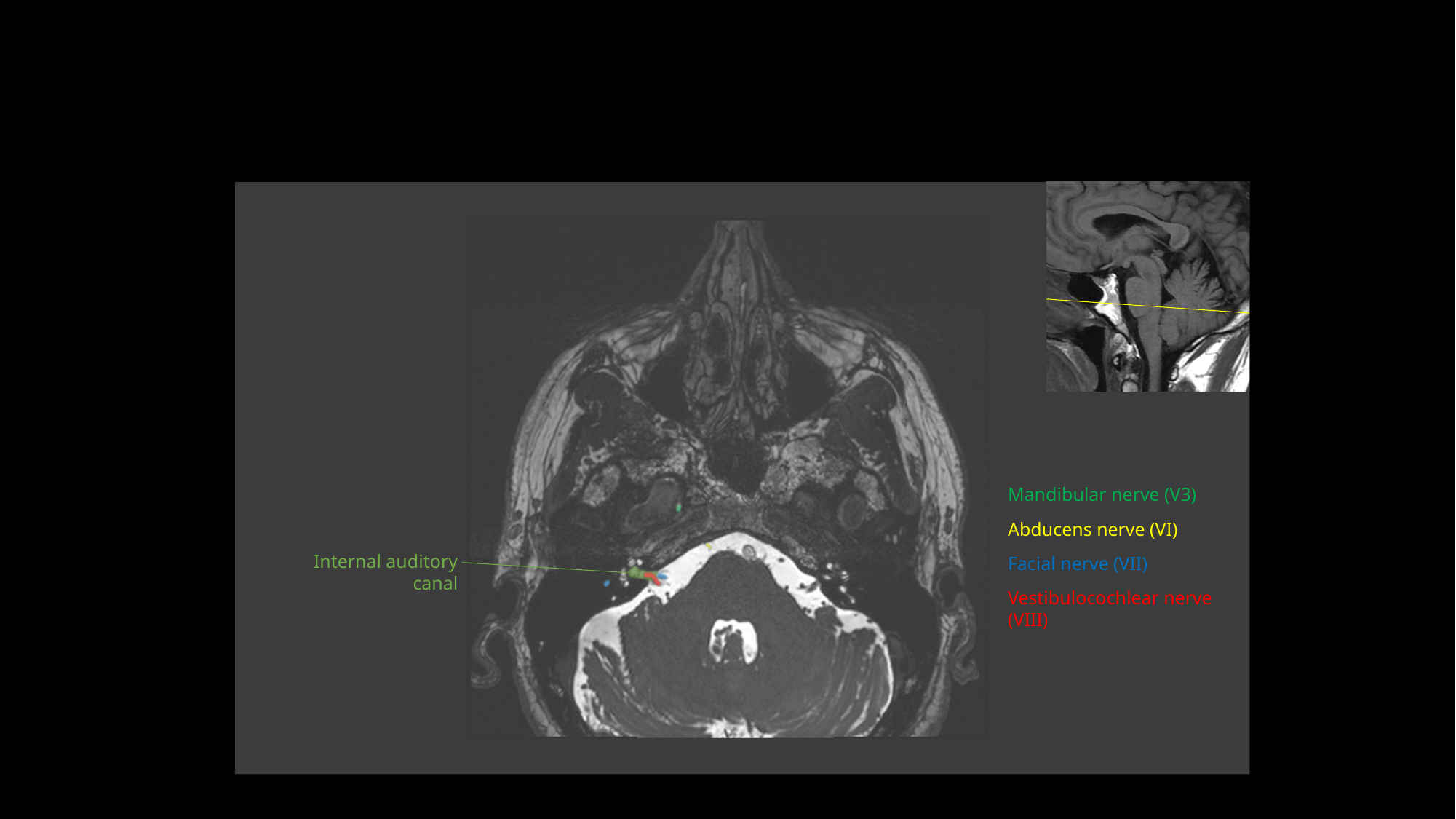

Mandibular nerve (V3)
Abducens nerve (VI)
Internal auditory canal
Facial nerve (VII)
Vestibulocochlear nerve (VIII)

## Slide 101
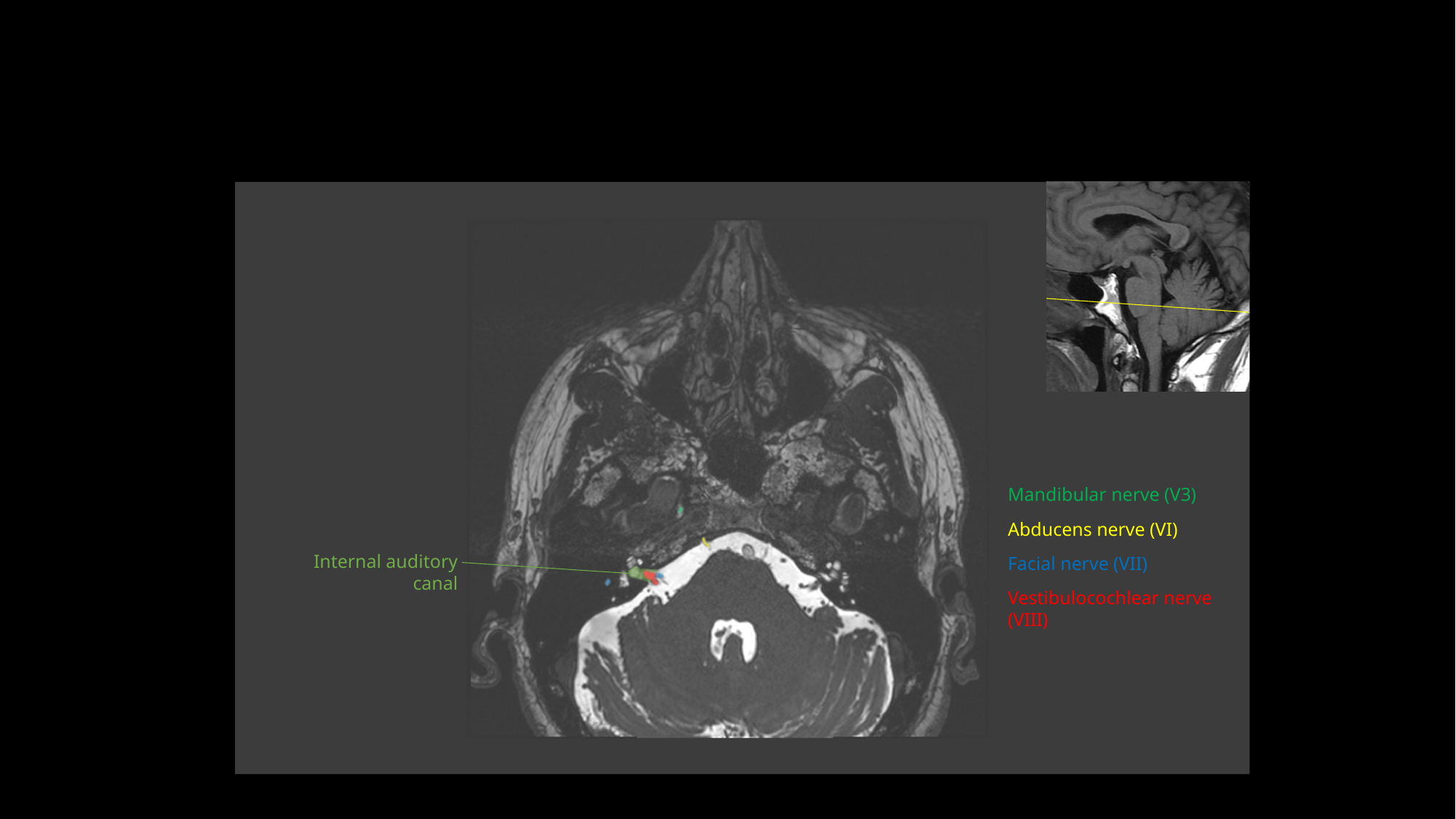

Mandibular nerve (V3)
Abducens nerve (VI)
Internal auditory canal
Facial nerve (VII)
Vestibulocochlear nerve (VIII)

## Slide 102
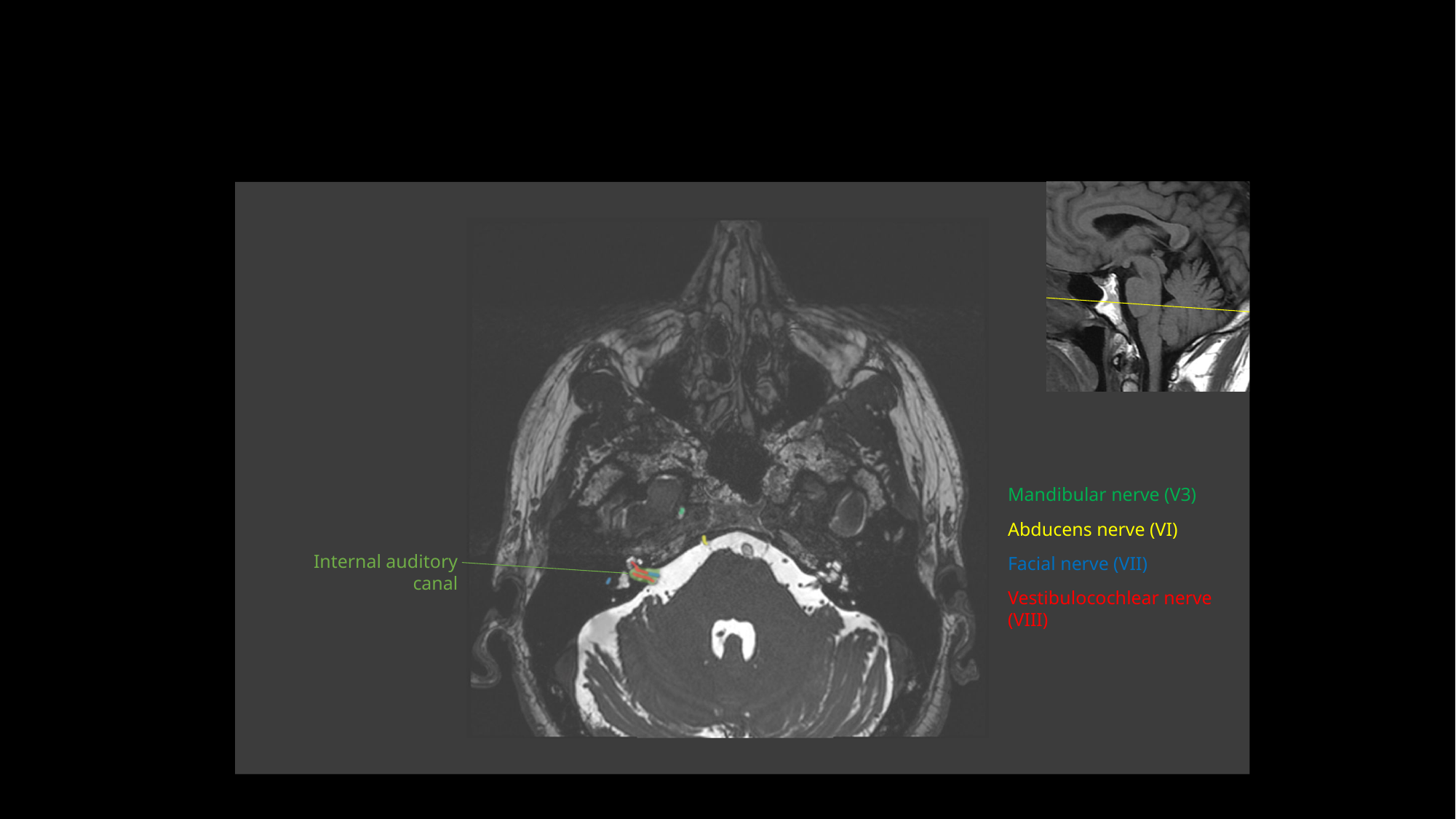

Mandibular nerve (V3)
Abducens nerve (VI)
Internal auditory canal
Facial nerve (VII)
Vestibulocochlear nerve (VIII)

## Slide 103
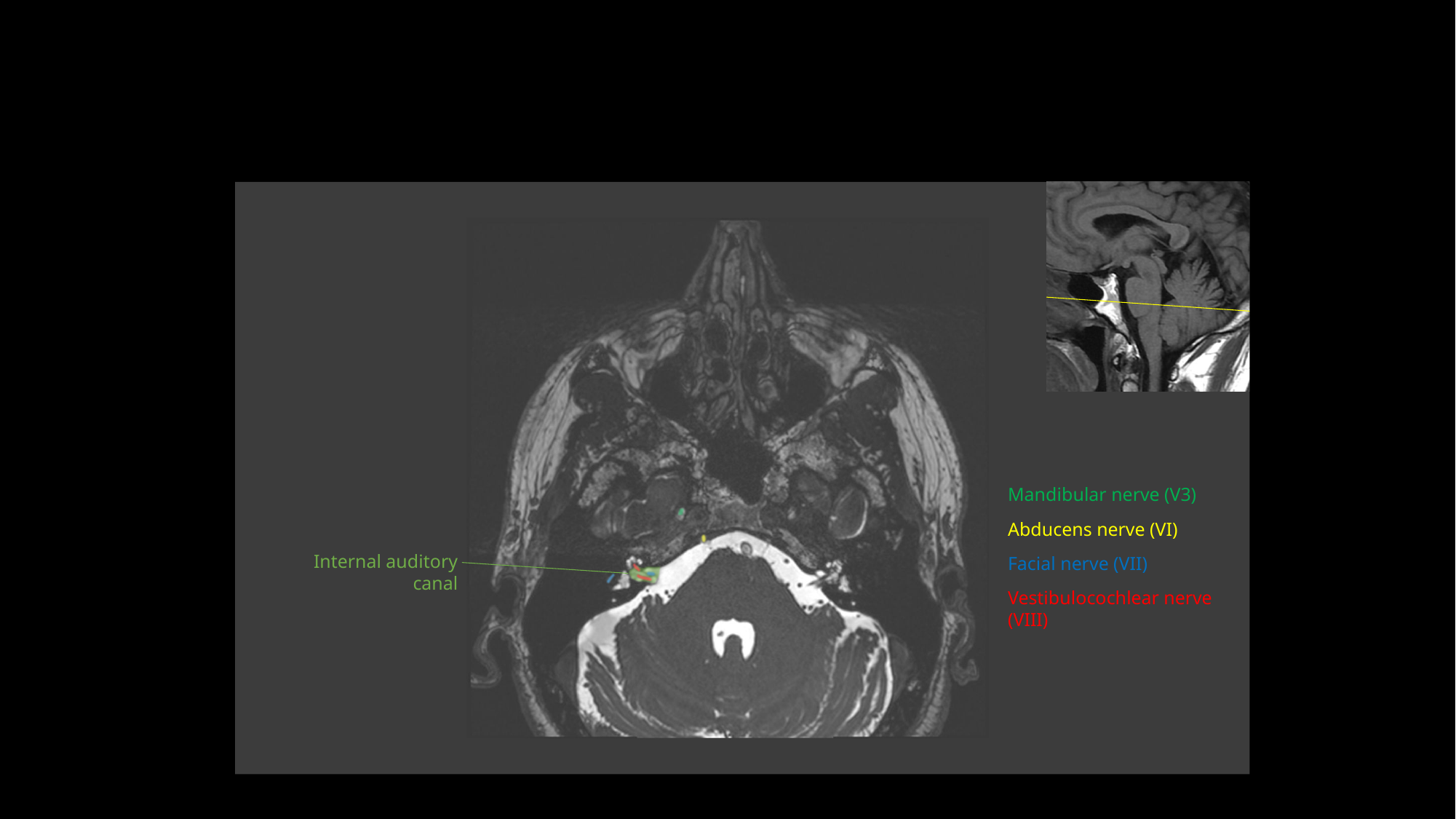

Mandibular nerve (V3)
Abducens nerve (VI)
Internal auditory canal
Facial nerve (VII)
Vestibulocochlear nerve (VIII)

## Slide 104
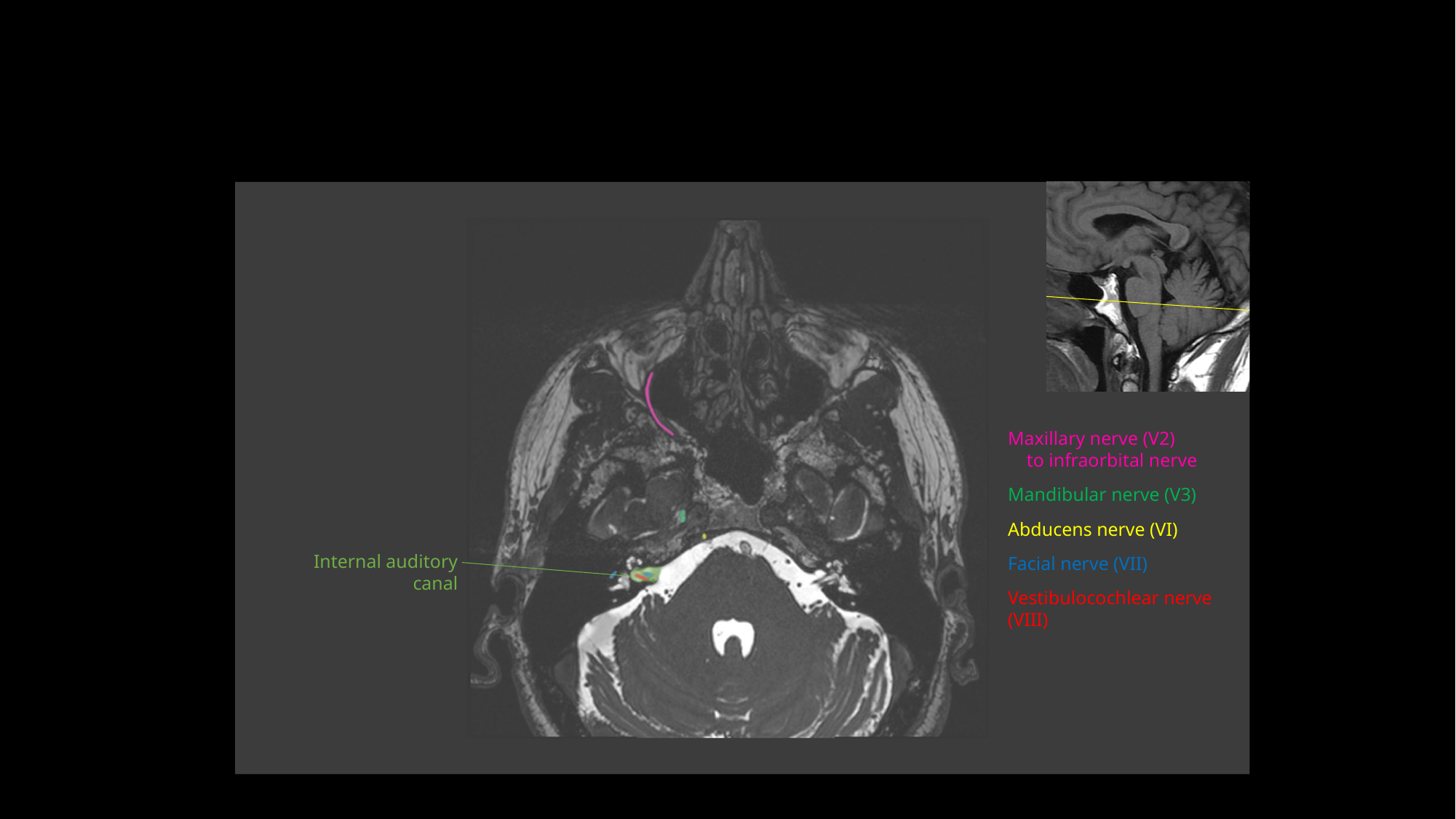

Maxillary nerve (V2)
 to infraorbital nerve
Mandibular nerve (V3)
Abducens nerve (VI)
Internal auditory canal
Facial nerve (VII)
Vestibulocochlear nerve (VIII)

## Slide 105
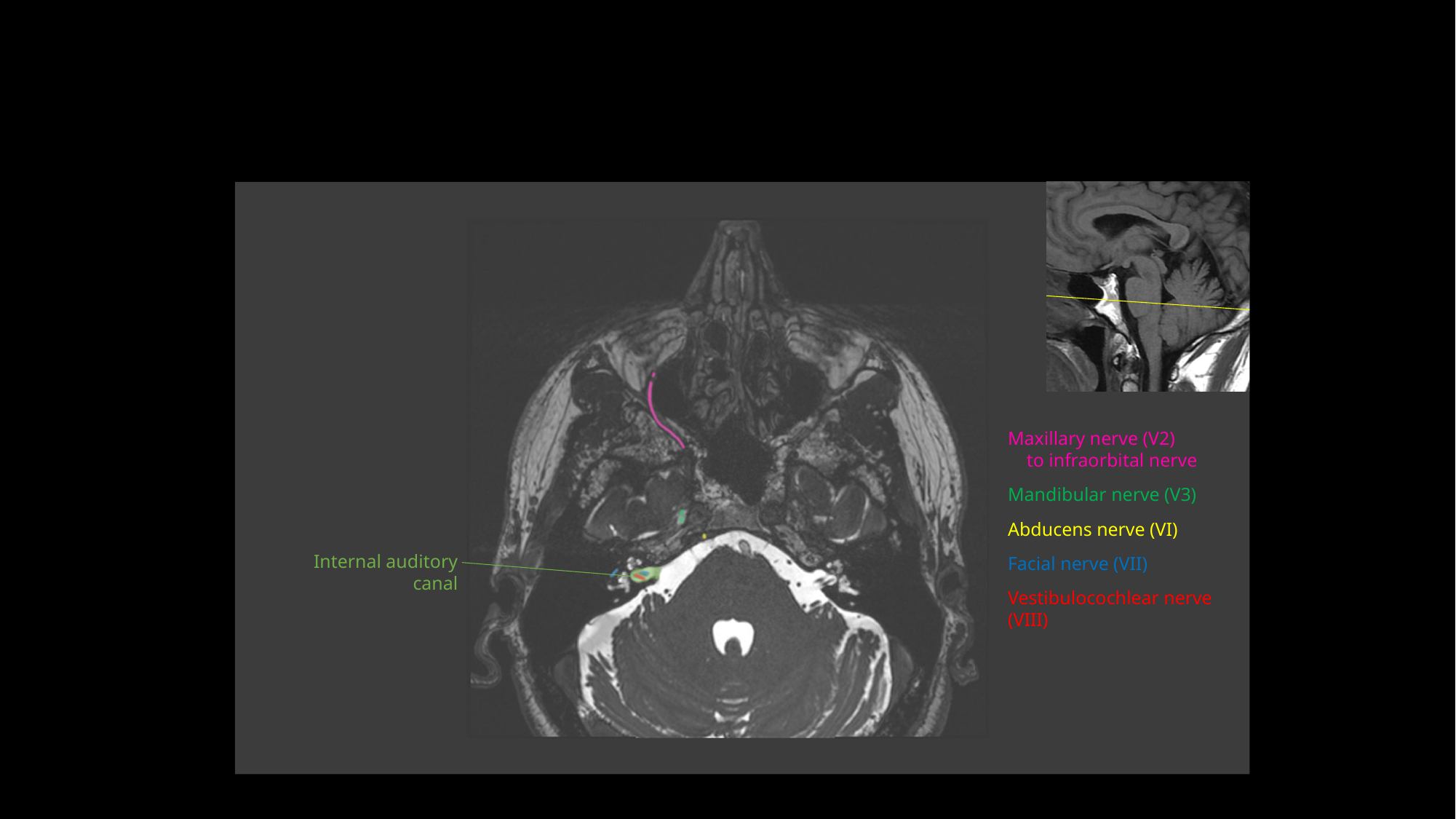

Maxillary nerve (V2)
 to infraorbital nerve
Mandibular nerve (V3)
Abducens nerve (VI)
Internal auditory canal
Facial nerve (VII)
Vestibulocochlear nerve (VIII)

## Slide 106
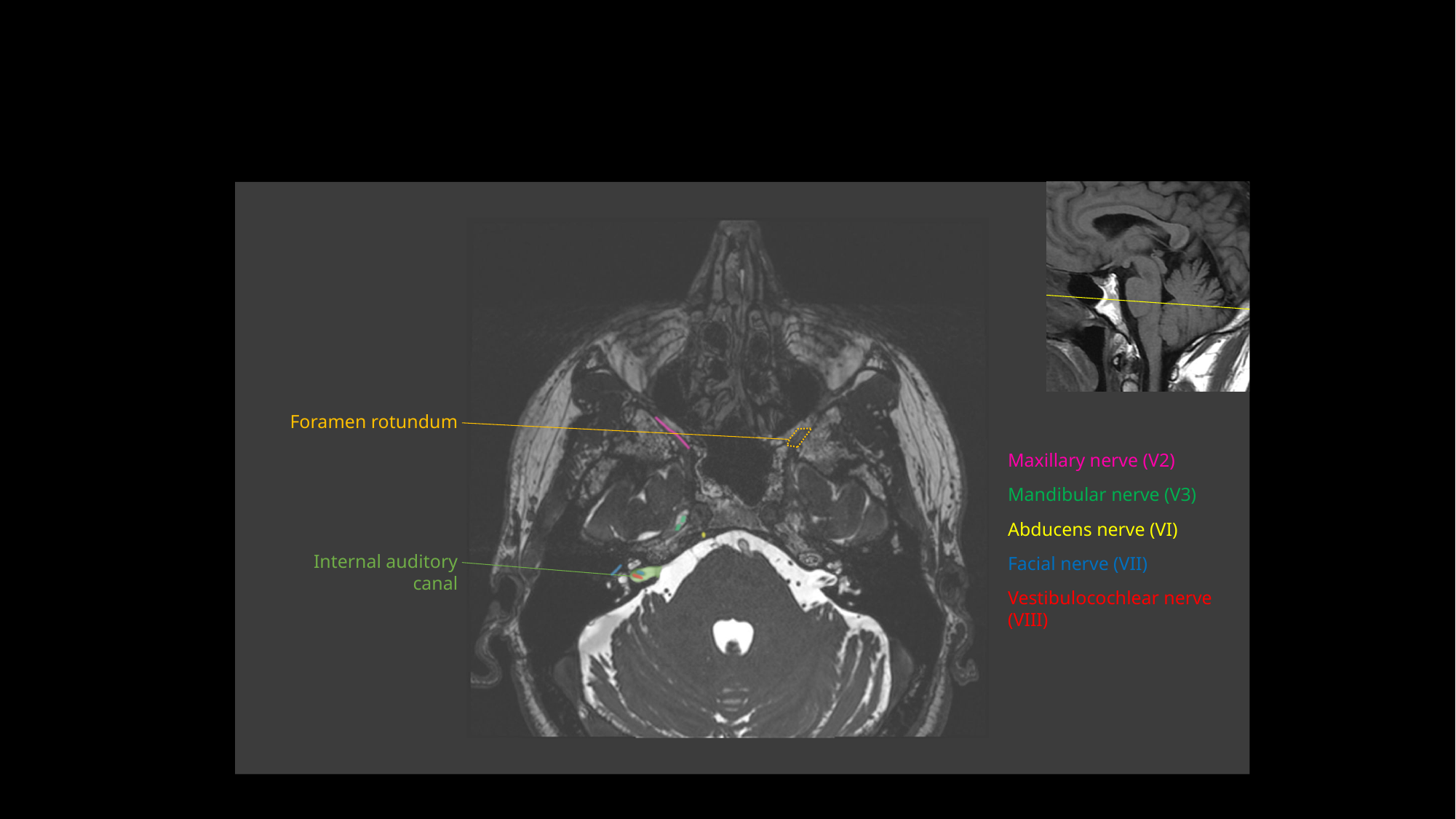

Foramen rotundum
Maxillary nerve (V2)
Mandibular nerve (V3)
Abducens nerve (VI)
Internal auditory canal
Facial nerve (VII)
Vestibulocochlear nerve (VIII)

## Slide 107
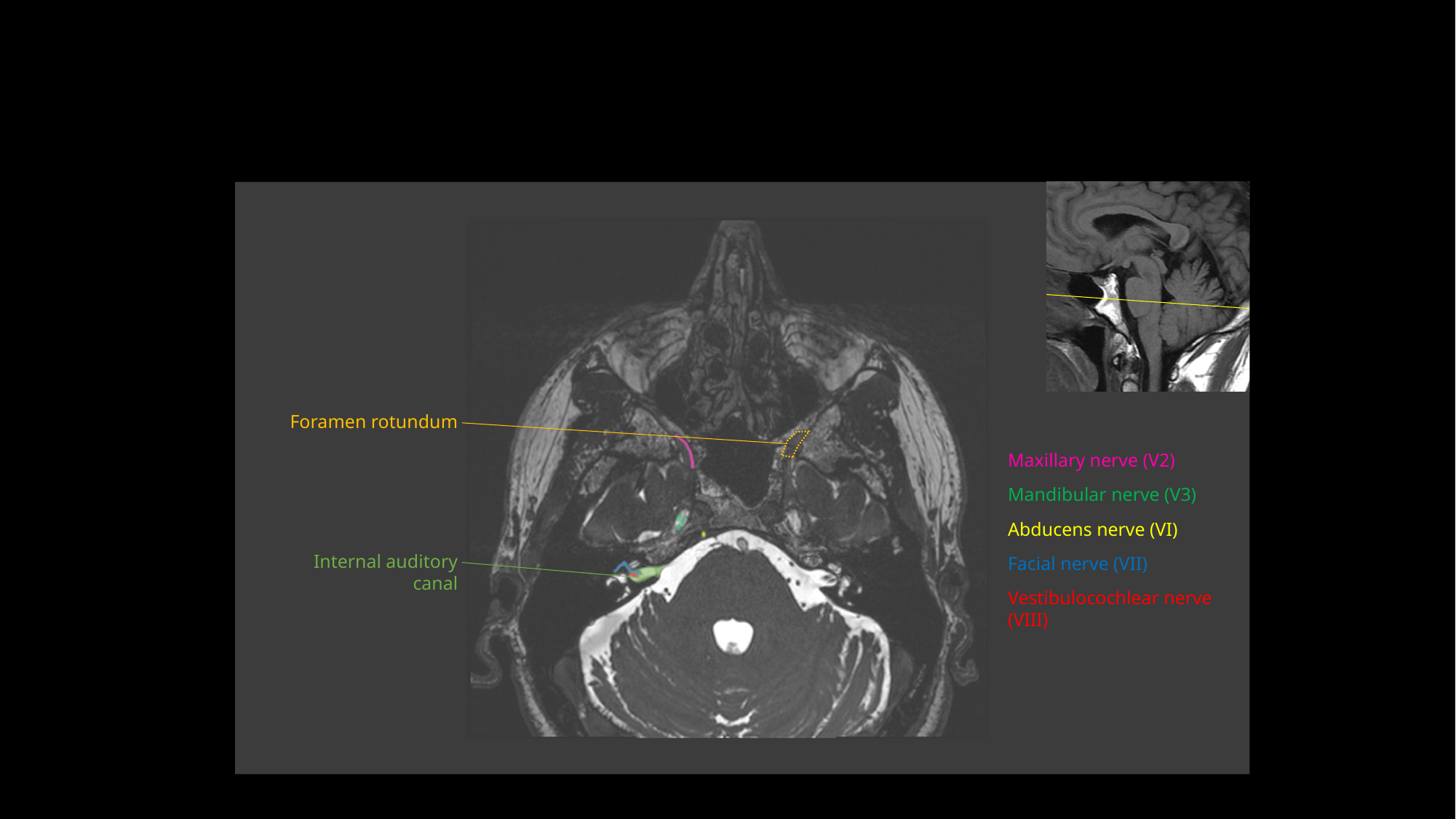

Foramen rotundum
Maxillary nerve (V2)
Mandibular nerve (V3)
Abducens nerve (VI)
Internal auditory canal
Facial nerve (VII)
Vestibulocochlear nerve (VIII)

## Slide 108
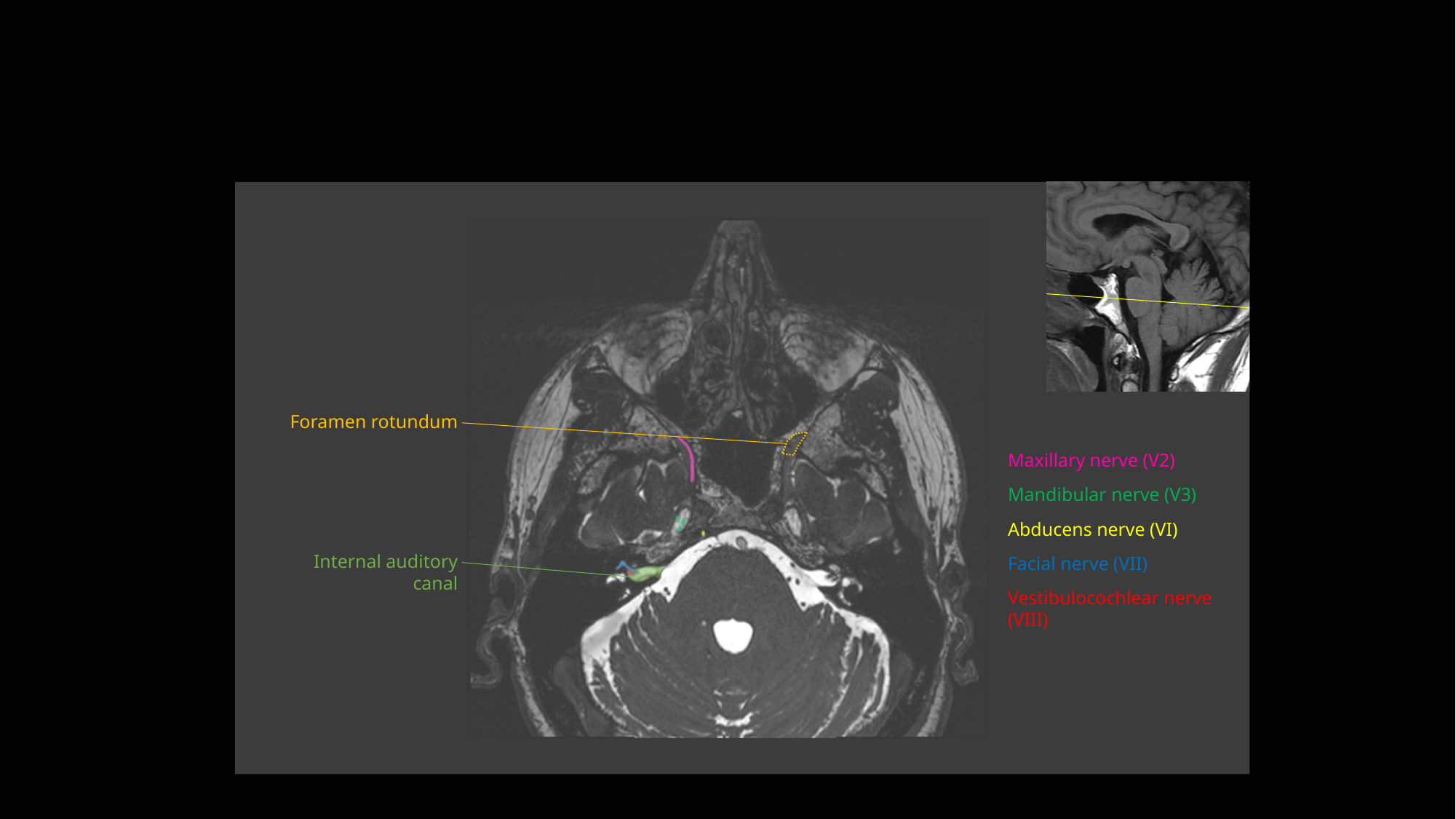

Foramen rotundum
Maxillary nerve (V2)
Mandibular nerve (V3)
Abducens nerve (VI)
Internal auditory canal
Facial nerve (VII)
Vestibulocochlear nerve (VIII)

## Slide 109
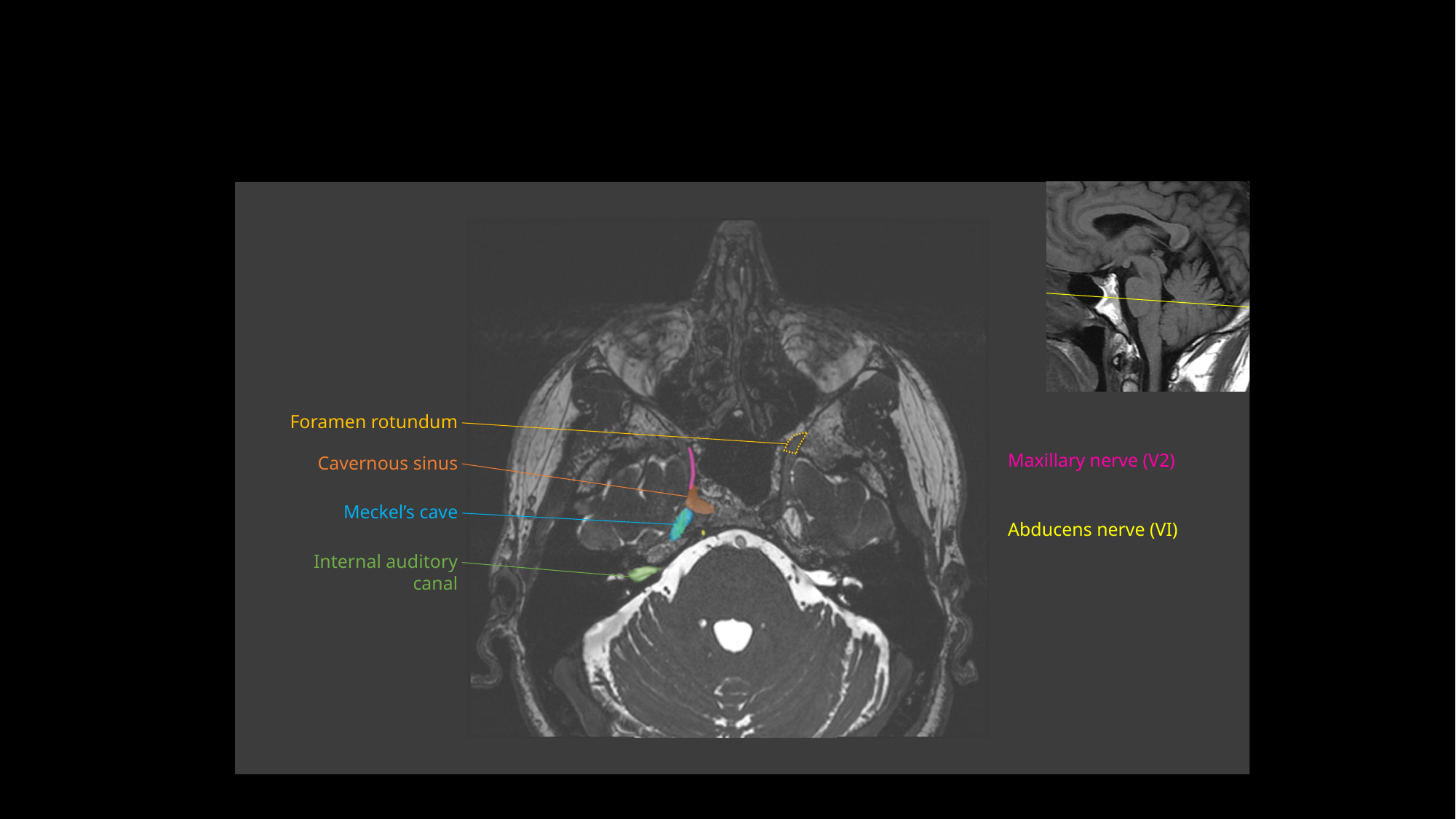

Foramen rotundum
Maxillary nerve (V2)
Cavernous sinus
Meckel’s cave
Abducens nerve (VI)
Internal auditory canal

## Slide 110
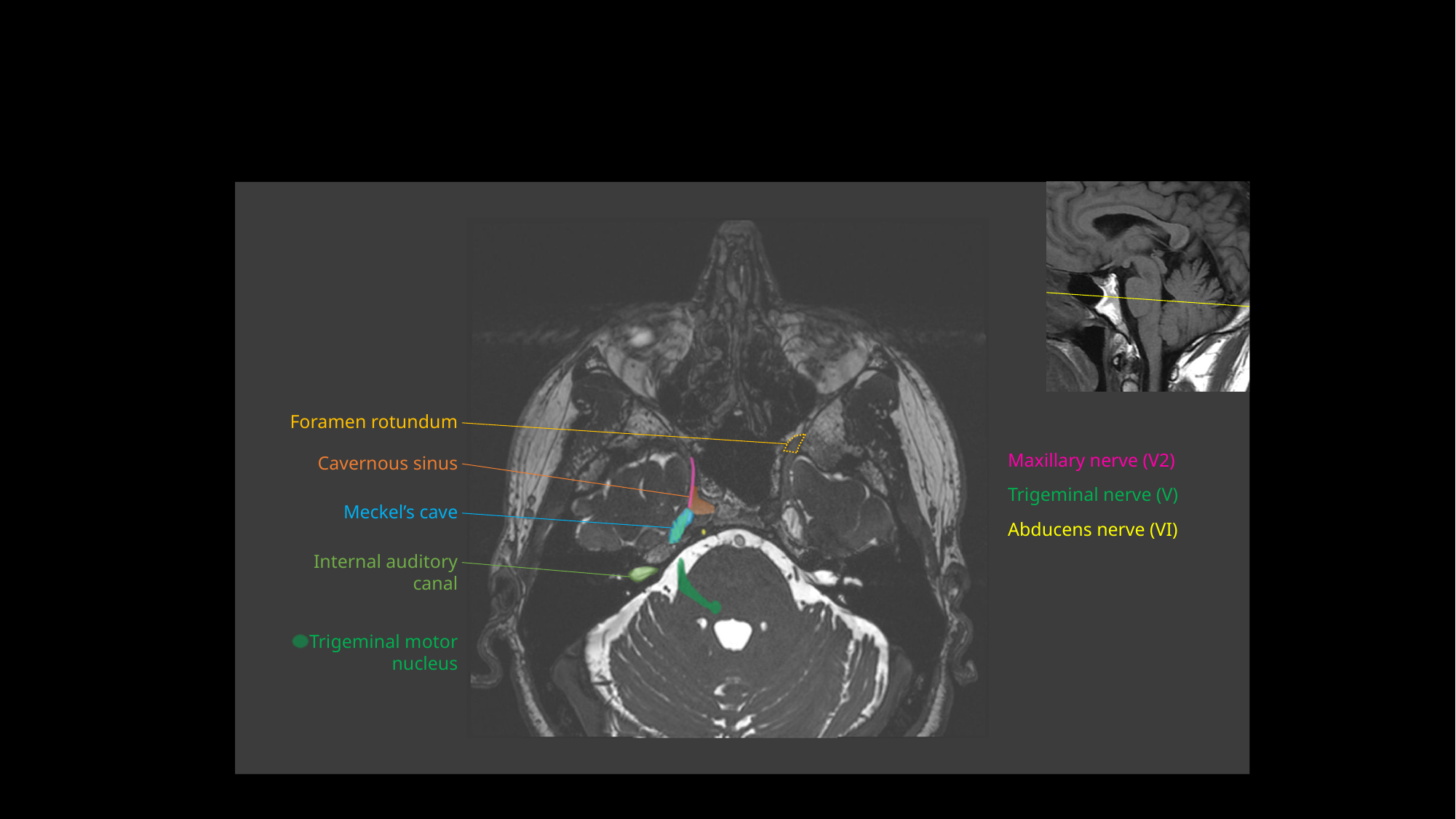

Foramen rotundum
Maxillary nerve (V2)
Cavernous sinus
Trigeminal nerve (V)
Meckel’s cave
Abducens nerve (VI)
Internal auditory canal
Trigeminal motor nucleus

## Slide 111
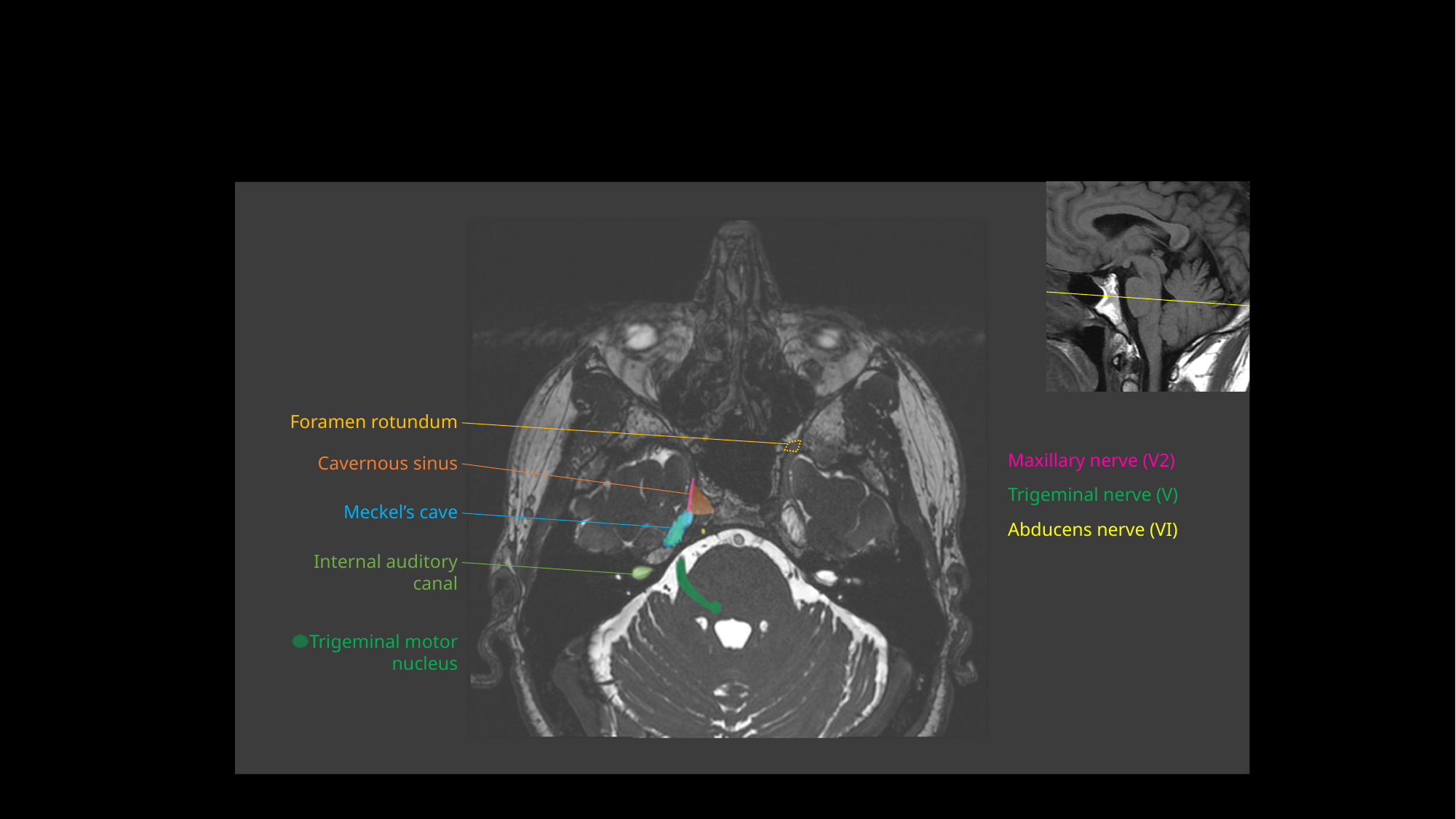

Foramen rotundum
Maxillary nerve (V2)
Cavernous sinus
Trigeminal nerve (V)
Meckel’s cave
Abducens nerve (VI)
Internal auditory canal
Trigeminal motor nucleus

## Slide 112
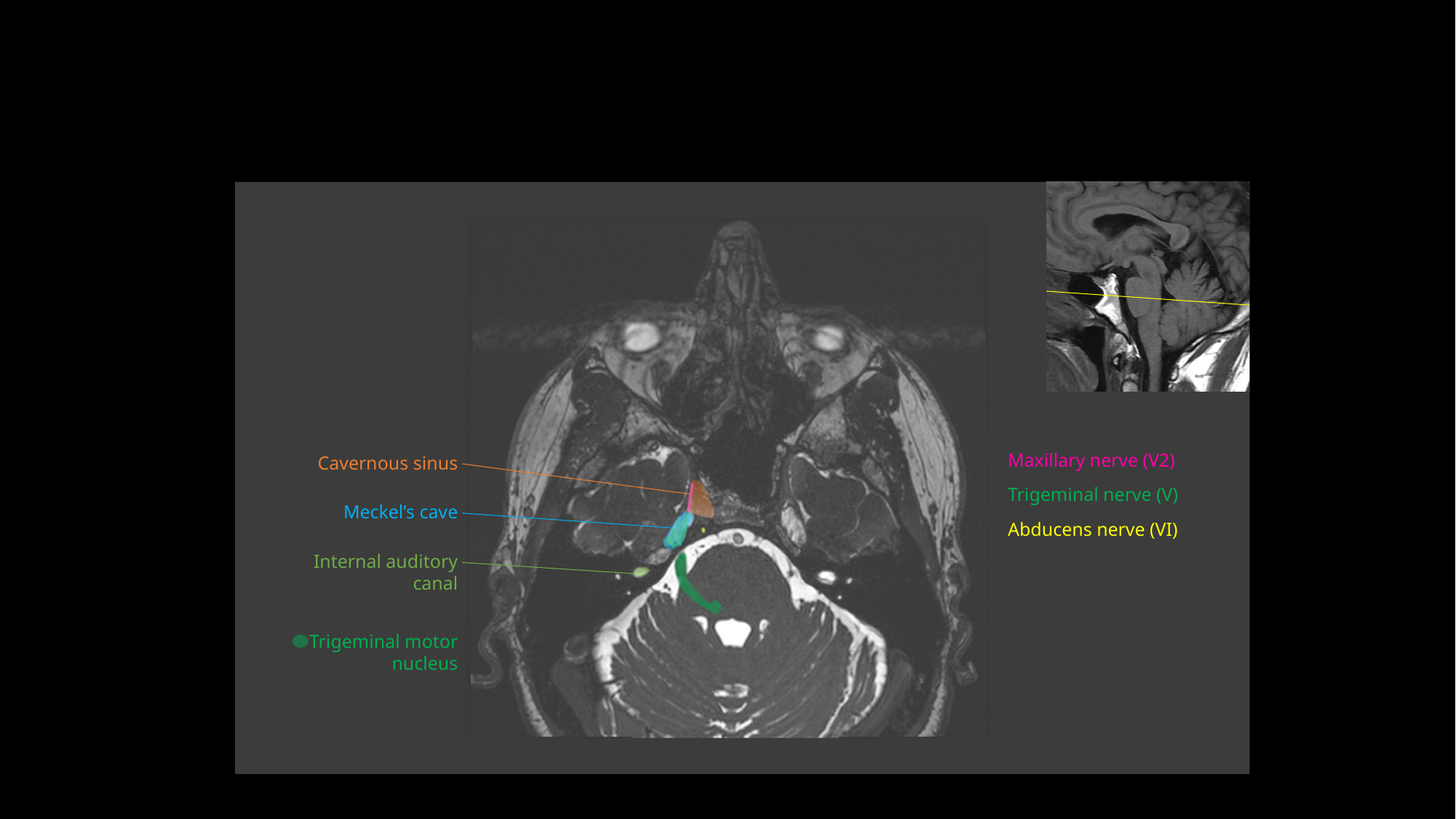

Maxillary nerve (V2)
Cavernous sinus
Trigeminal nerve (V)
Meckel’s cave
Abducens nerve (VI)
Internal auditory canal
Trigeminal motor nucleus

## Slide 113
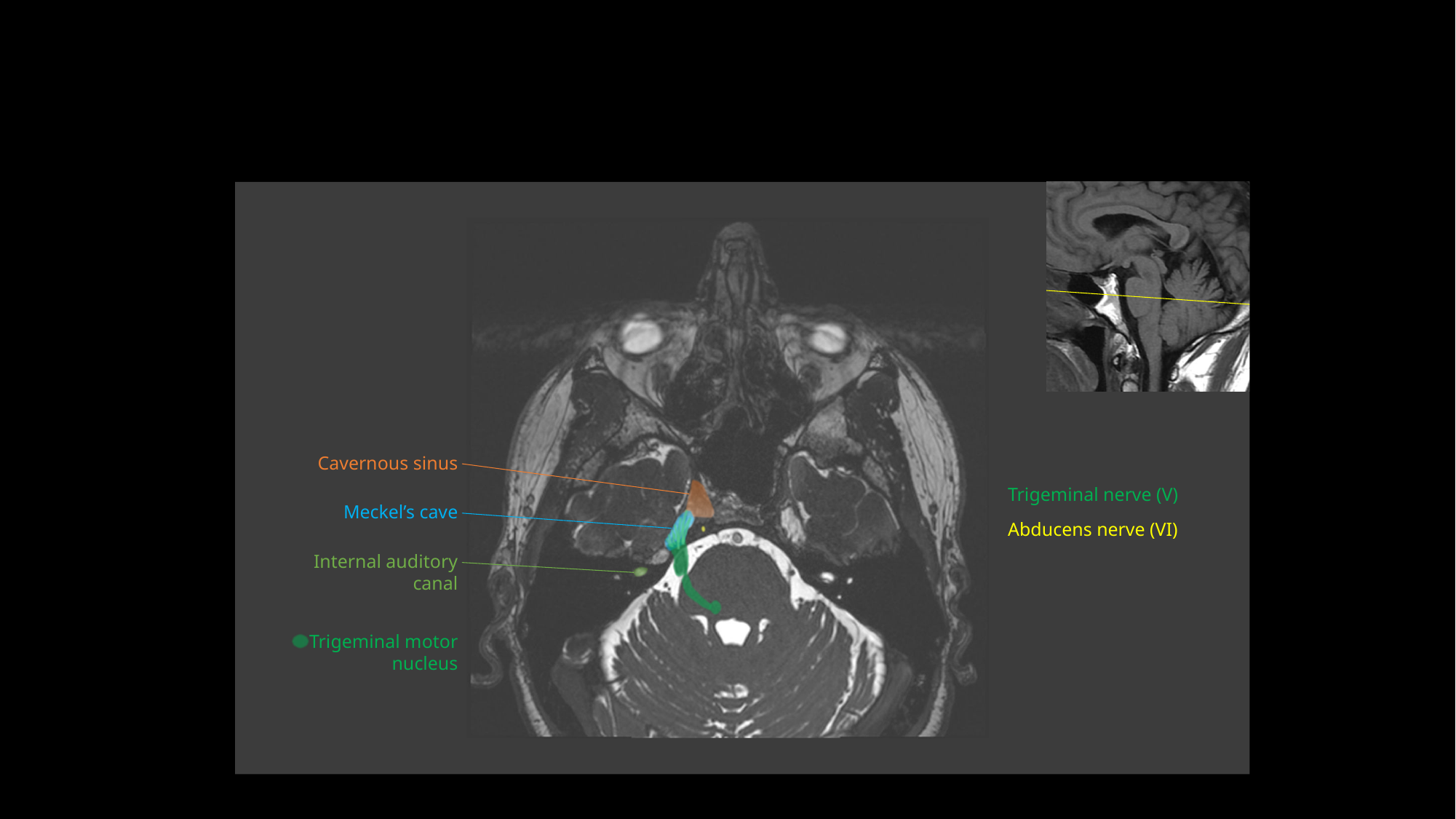

Cavernous sinus
Trigeminal nerve (V)
Meckel’s cave
Abducens nerve (VI)
Internal auditory canal
Trigeminal motor nucleus

## Slide 114
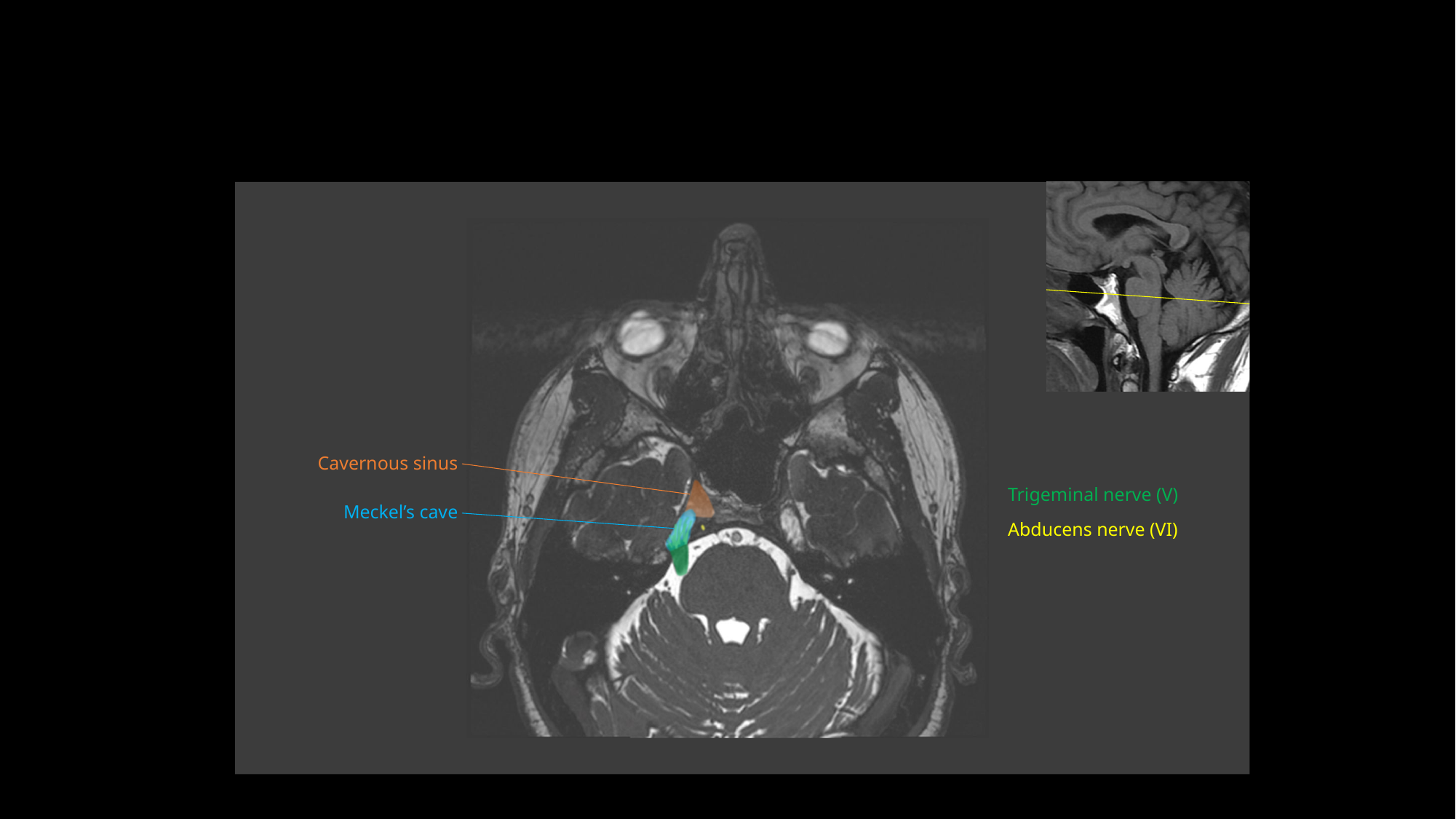

Cavernous sinus
Trigeminal nerve (V)
Meckel’s cave
Abducens nerve (VI)

## Slide 115
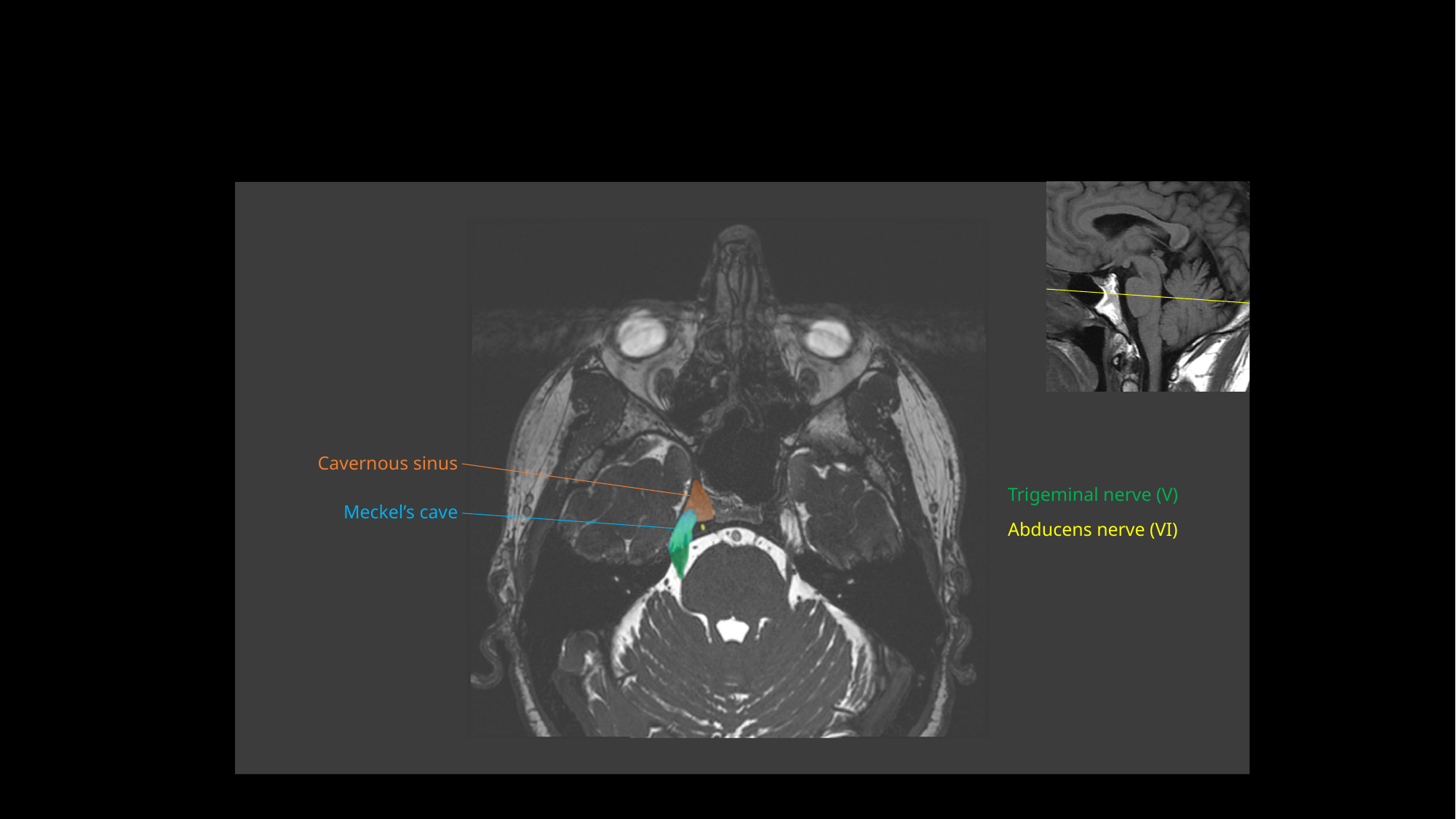

Cavernous sinus
Trigeminal nerve (V)
Meckel’s cave
Abducens nerve (VI)

## Slide 116
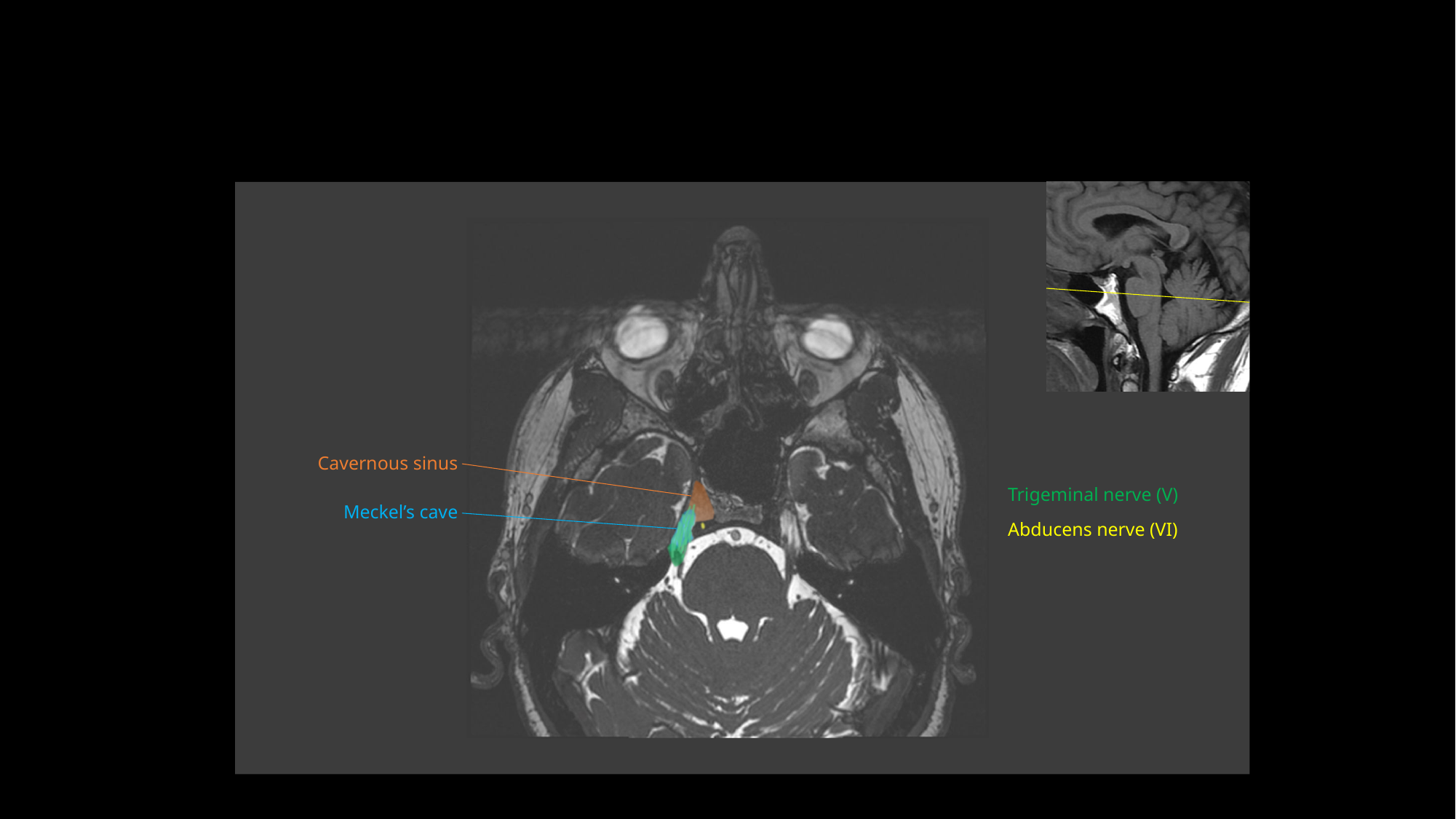

Cavernous sinus
Trigeminal nerve (V)
Meckel’s cave
Abducens nerve (VI)

## Slide 117
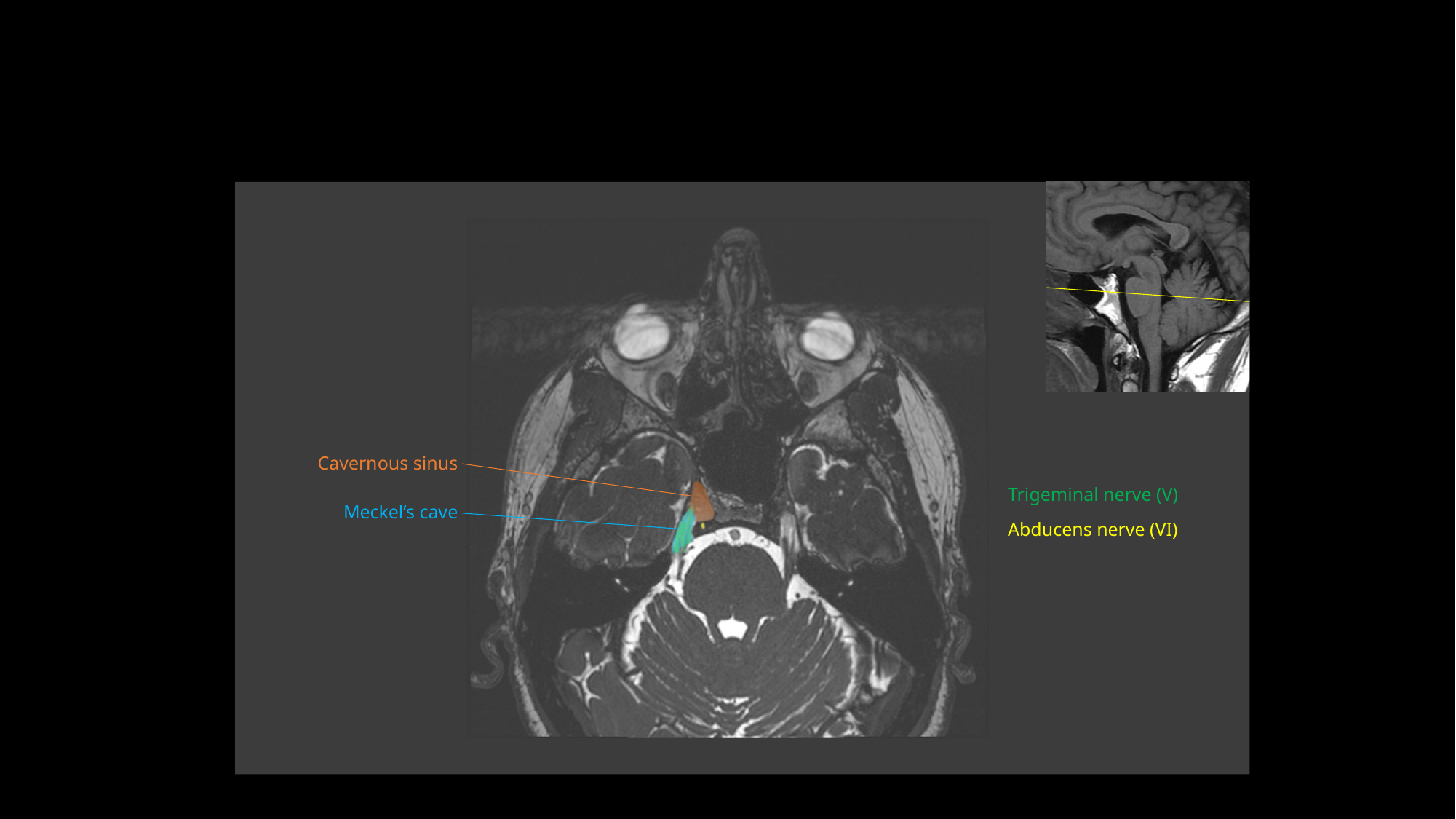

Cavernous sinus
Trigeminal nerve (V)
Meckel’s cave
Abducens nerve (VI)

## Slide 118
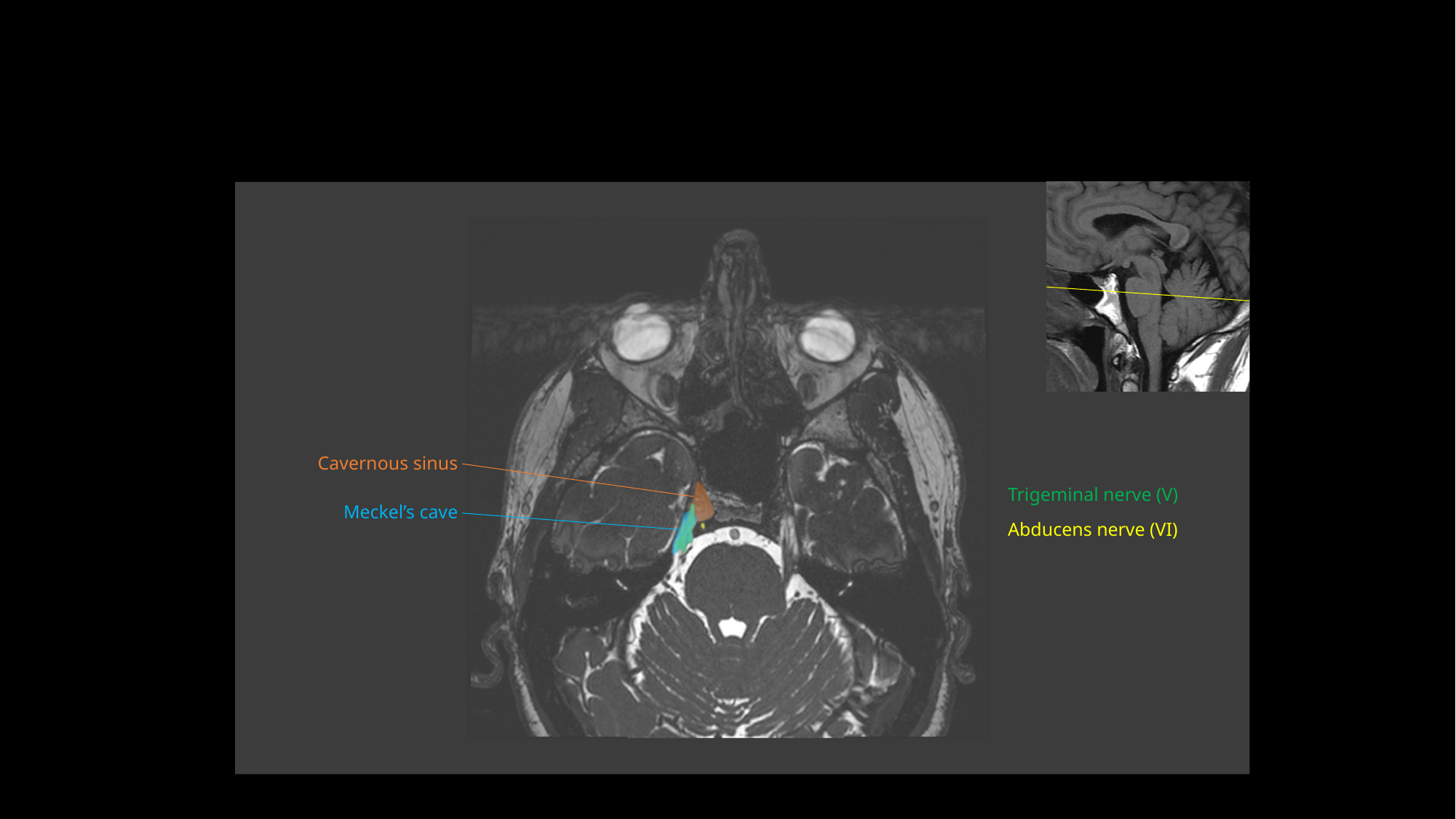

Cavernous sinus
Trigeminal nerve (V)
Meckel’s cave
Abducens nerve (VI)

## Slide 119
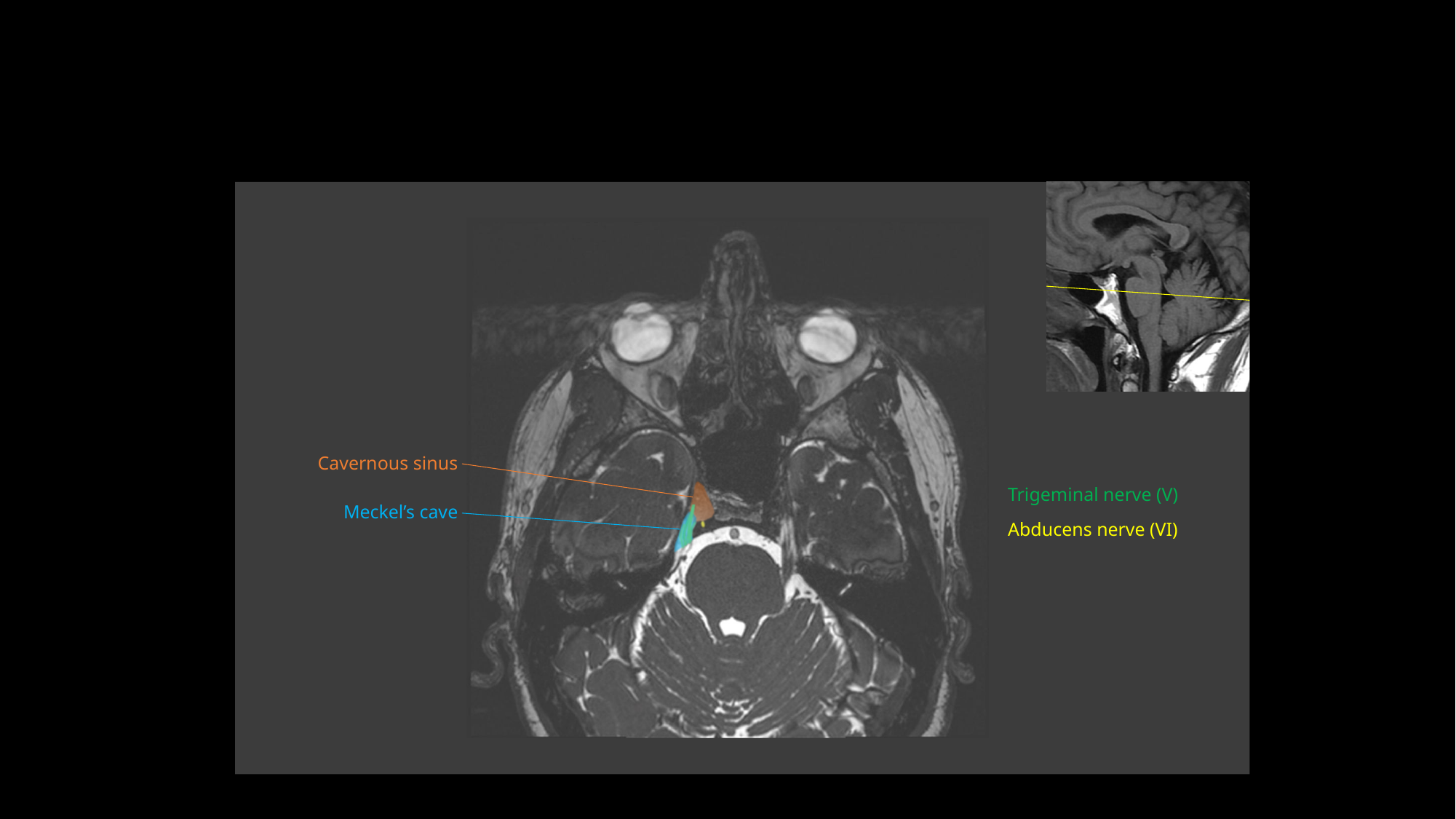

Cavernous sinus
Trigeminal nerve (V)
Meckel’s cave
Abducens nerve (VI)

## Slide 120
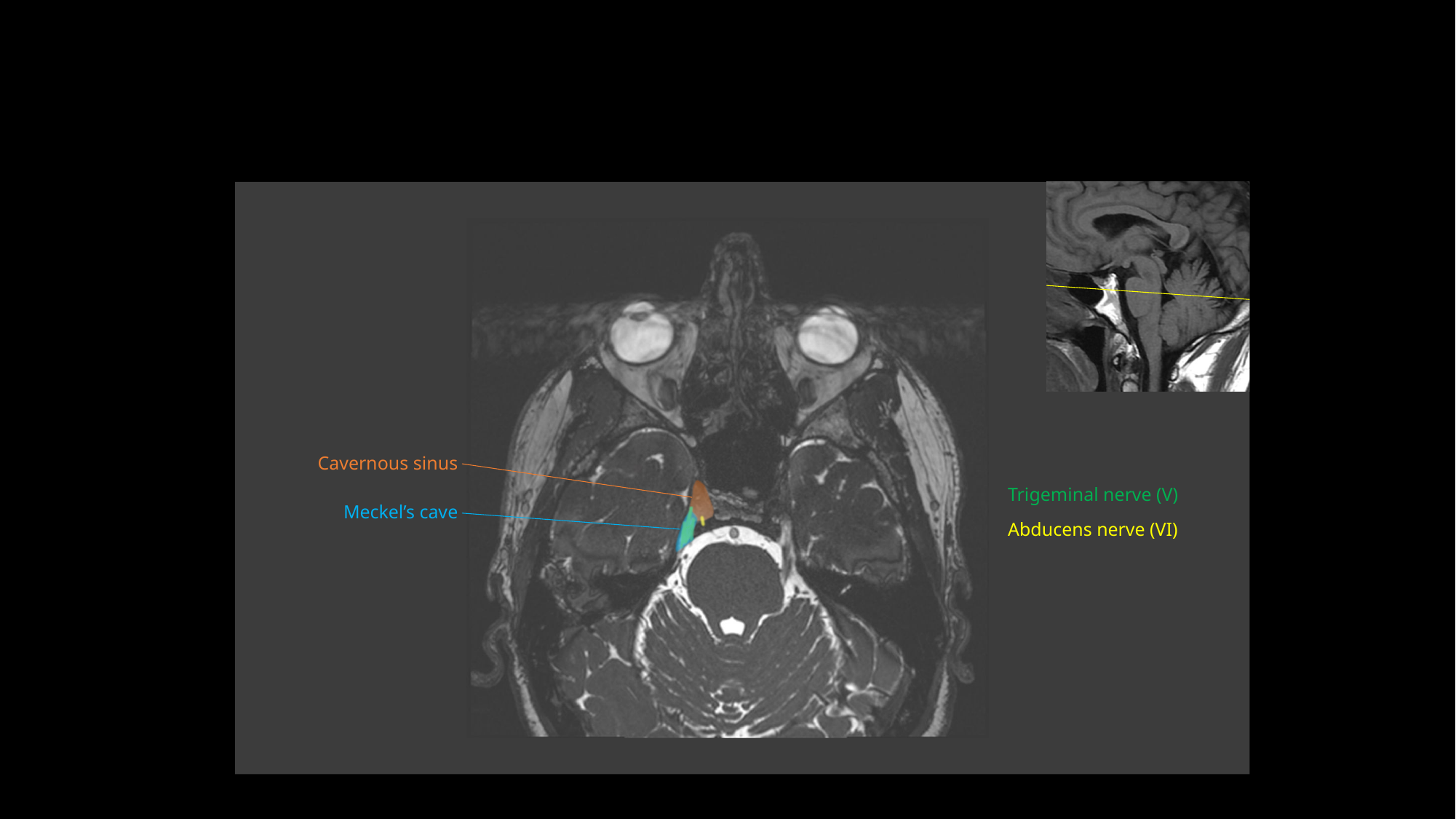

Cavernous sinus
Trigeminal nerve (V)
Meckel’s cave
Abducens nerve (VI)

## Slide 121
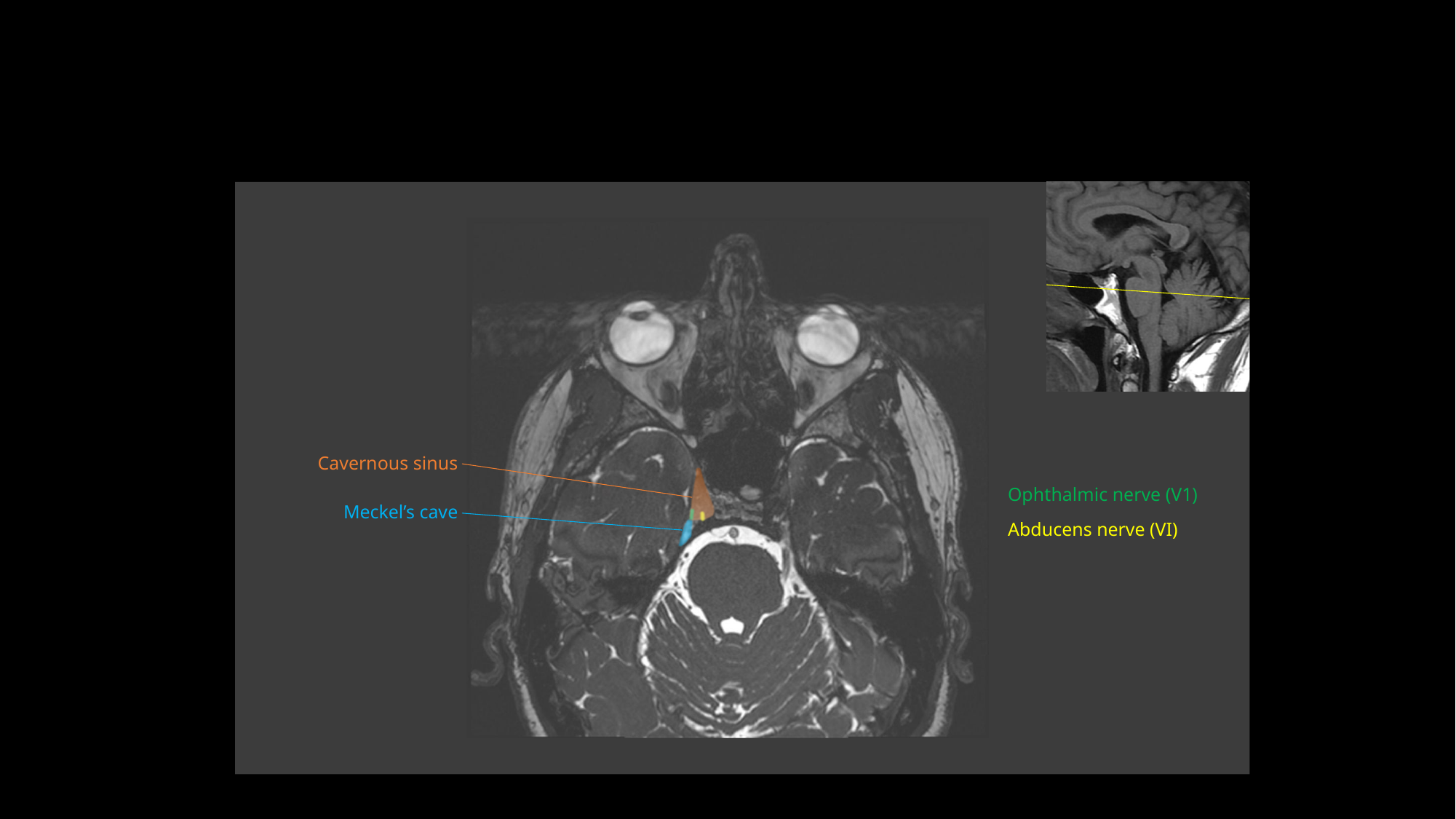

Cavernous sinus
Ophthalmic nerve (V1)
Meckel’s cave
Abducens nerve (VI)

## Slide 122
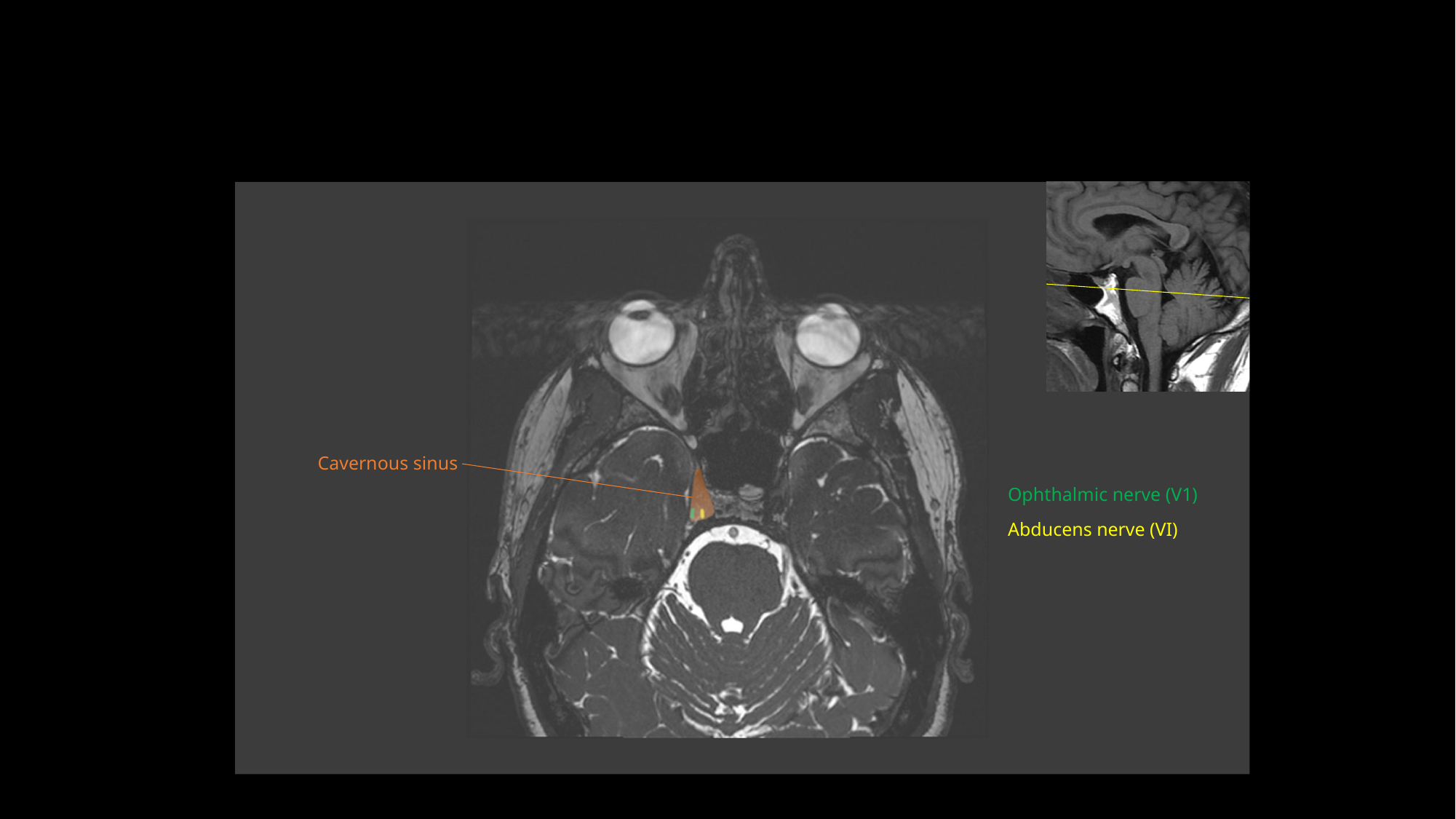

Cavernous sinus
Ophthalmic nerve (V1)
Abducens nerve (VI)

## Slide 123
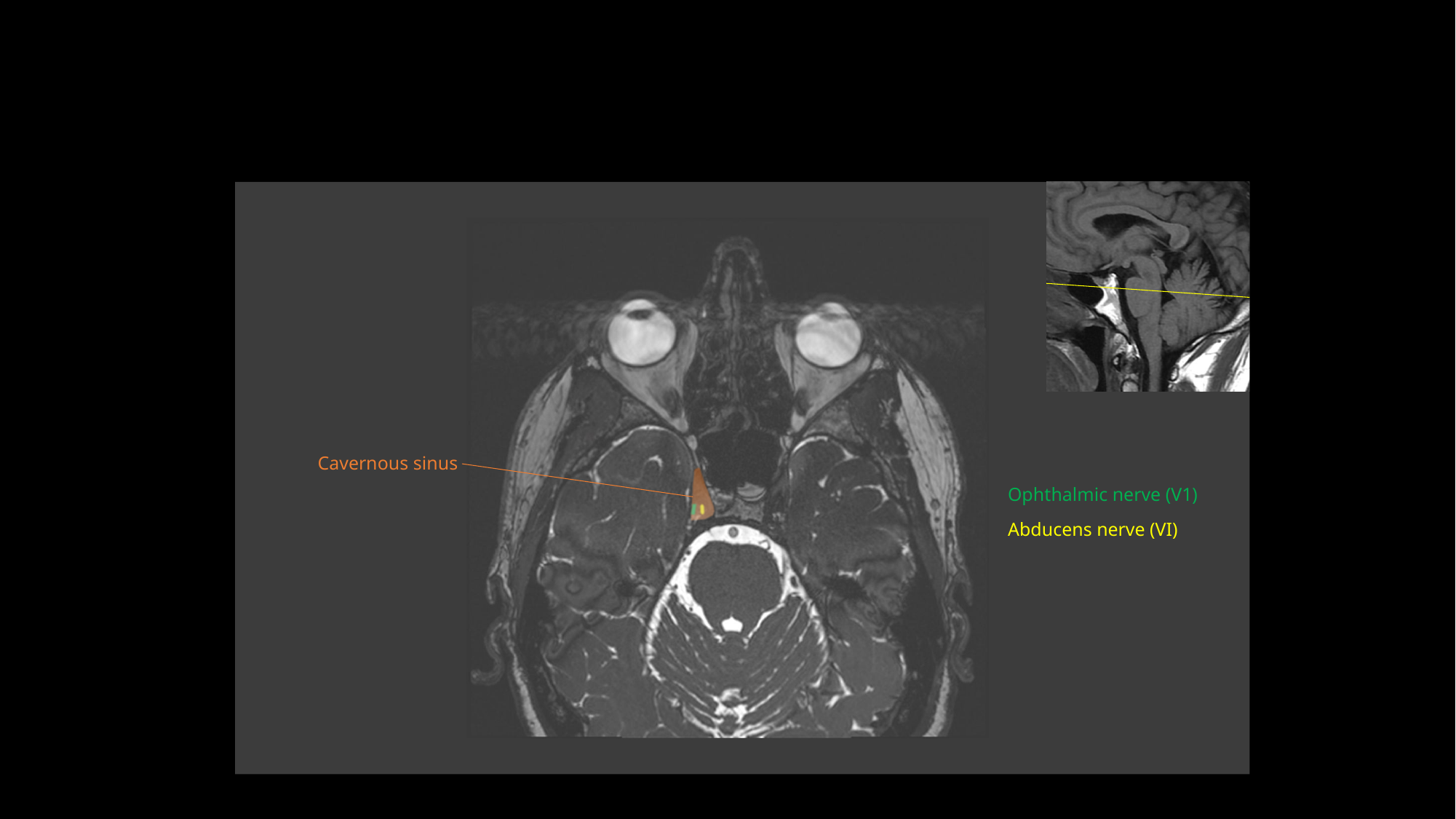

Cavernous sinus
Ophthalmic nerve (V1)
Abducens nerve (VI)

## Slide 124
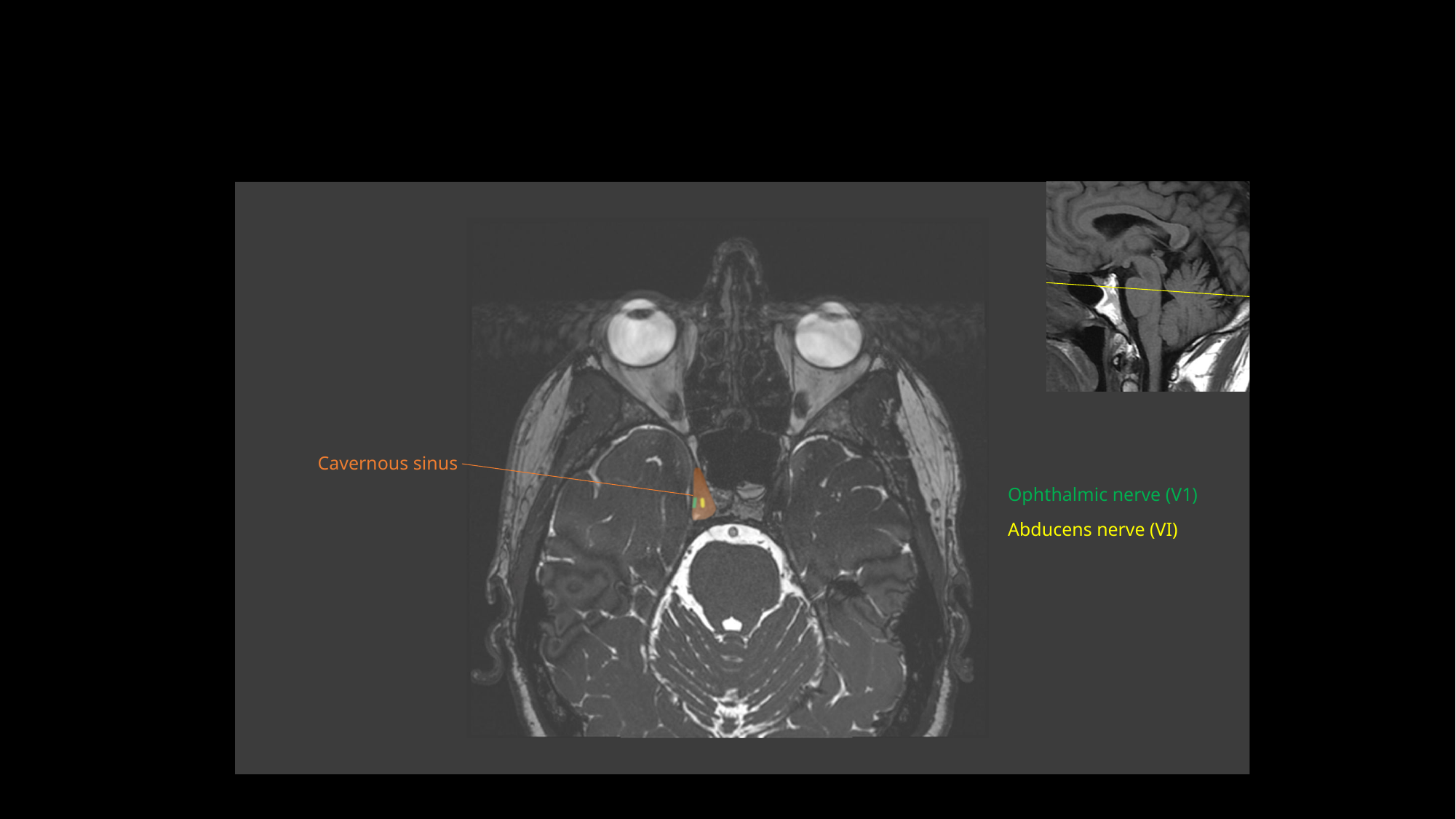

Cavernous sinus
Ophthalmic nerve (V1)
Abducens nerve (VI)

## Slide 125
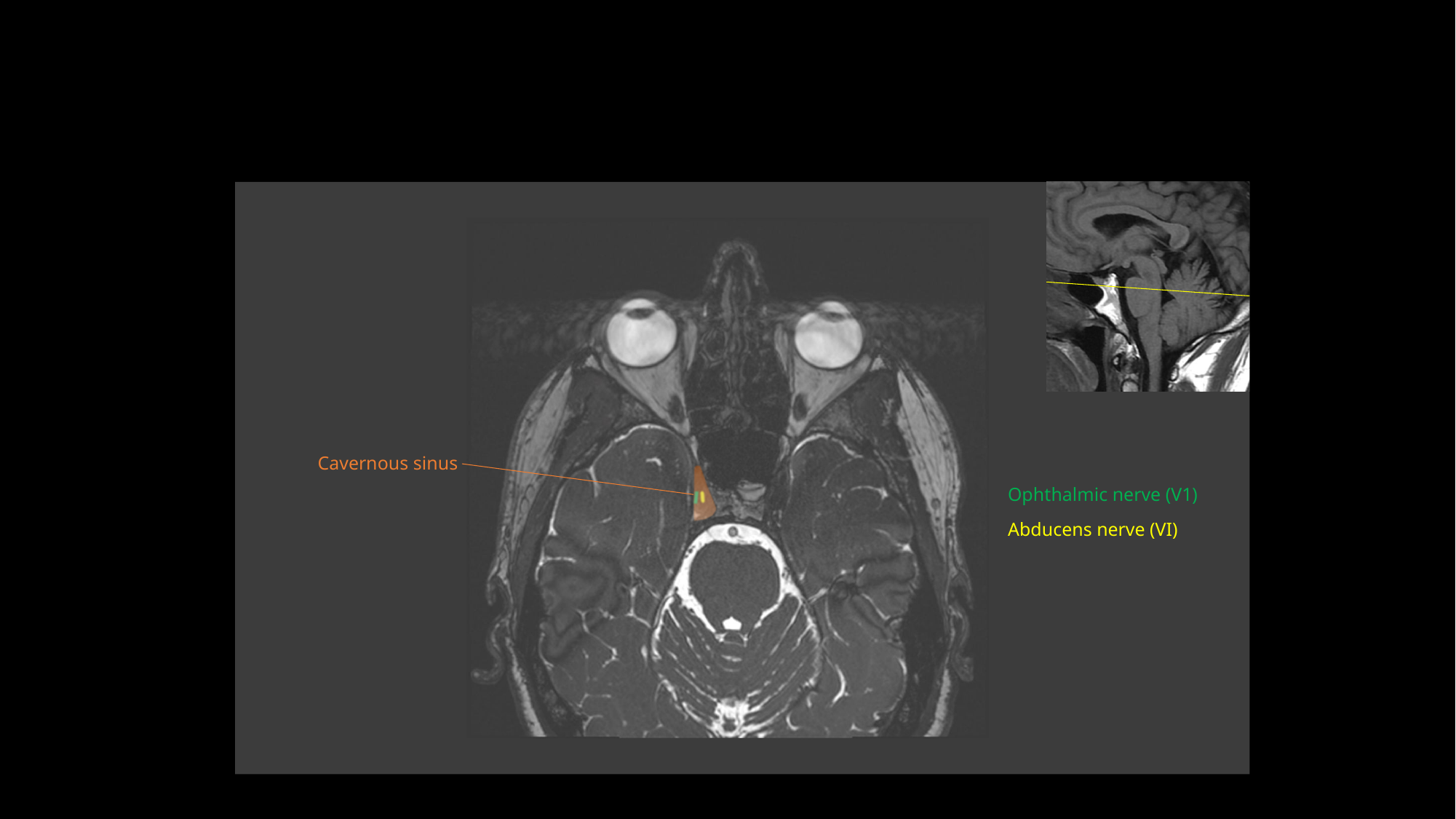

Cavernous sinus
Ophthalmic nerve (V1)
Abducens nerve (VI)

## Slide 126
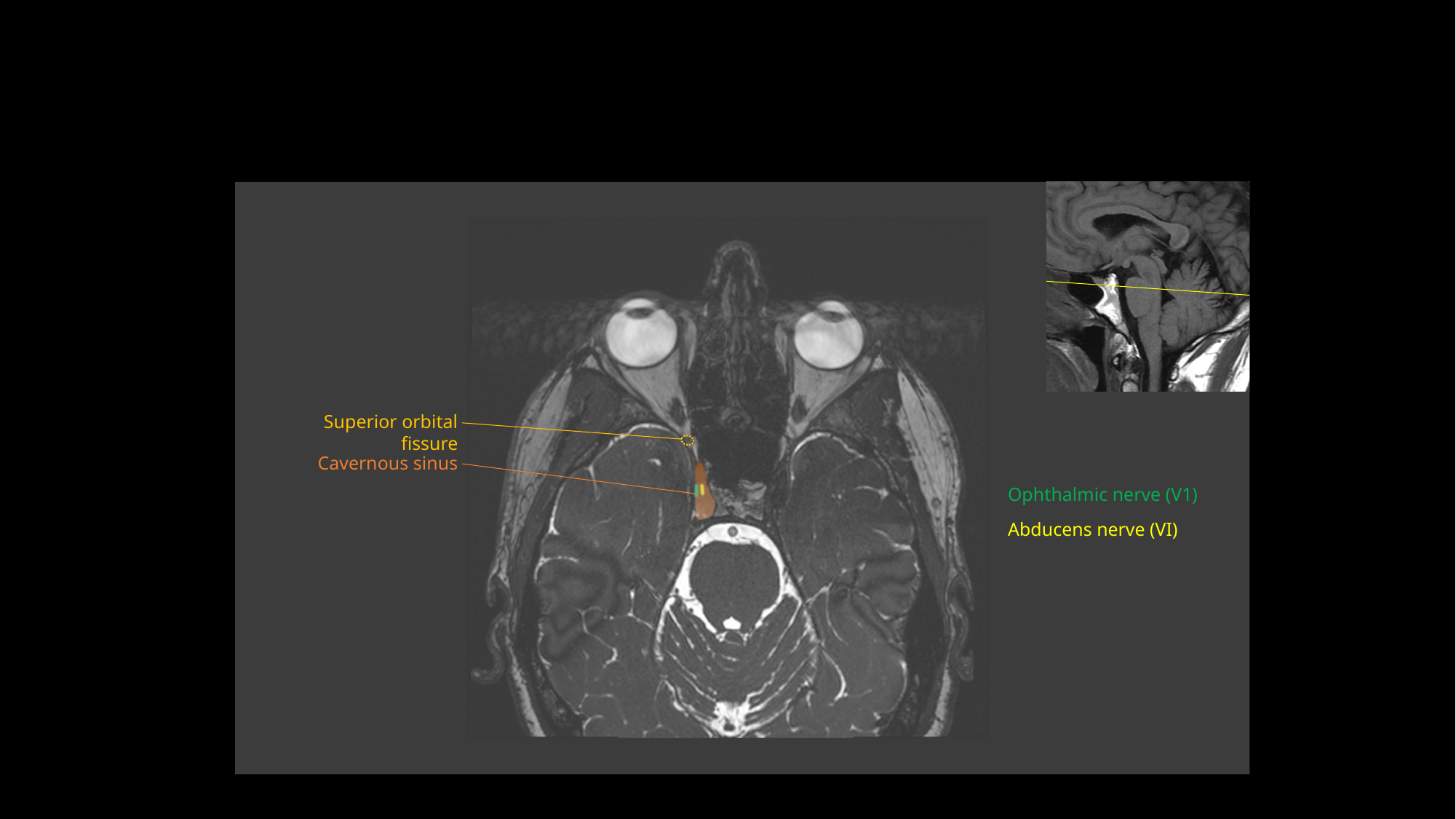

Superior orbital fissure
Cavernous sinus
Ophthalmic nerve (V1)
Abducens nerve (VI)

## Slide 127
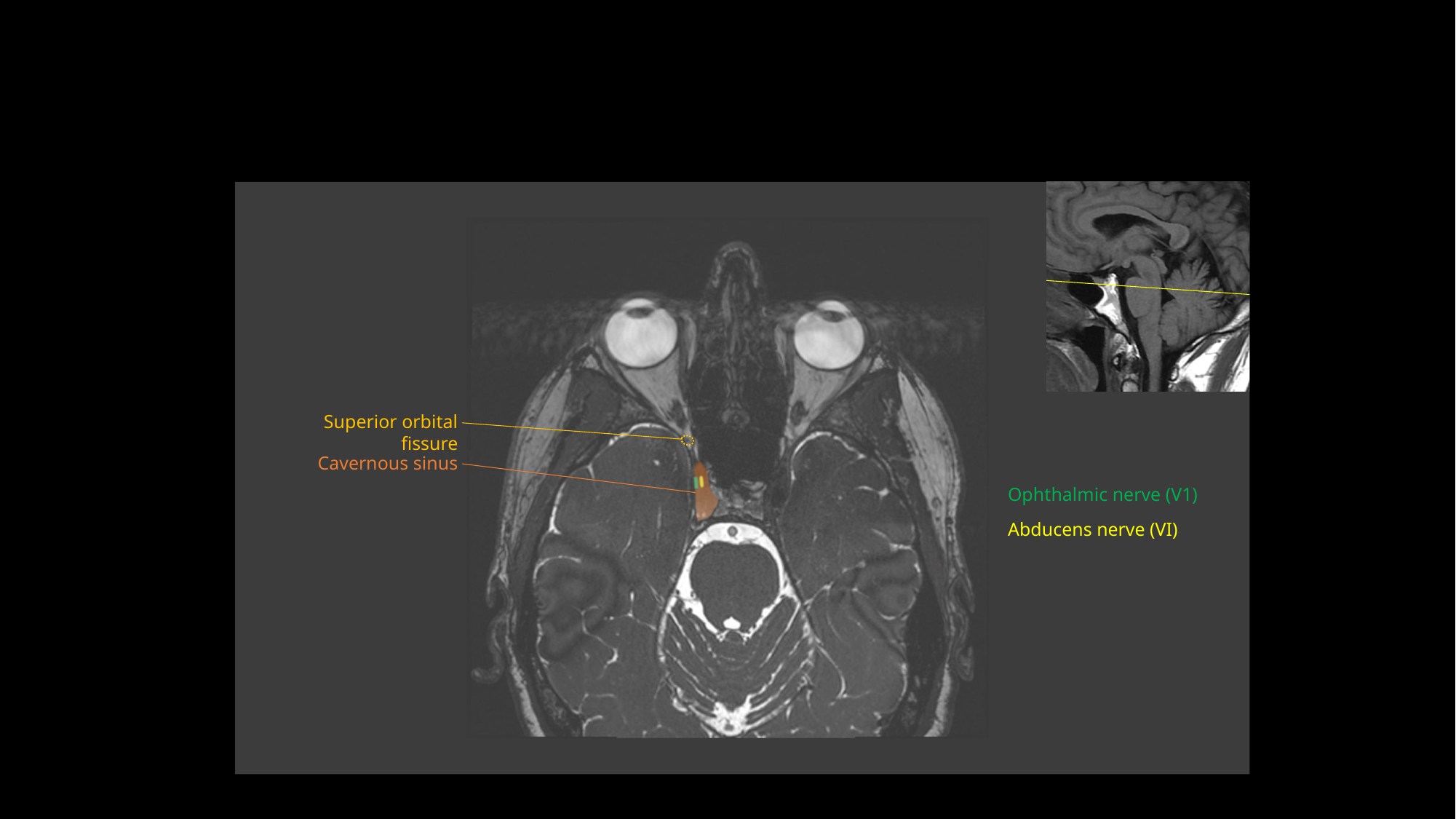

Superior orbital fissure
Cavernous sinus
Ophthalmic nerve (V1)
Abducens nerve (VI)

## Slide 128
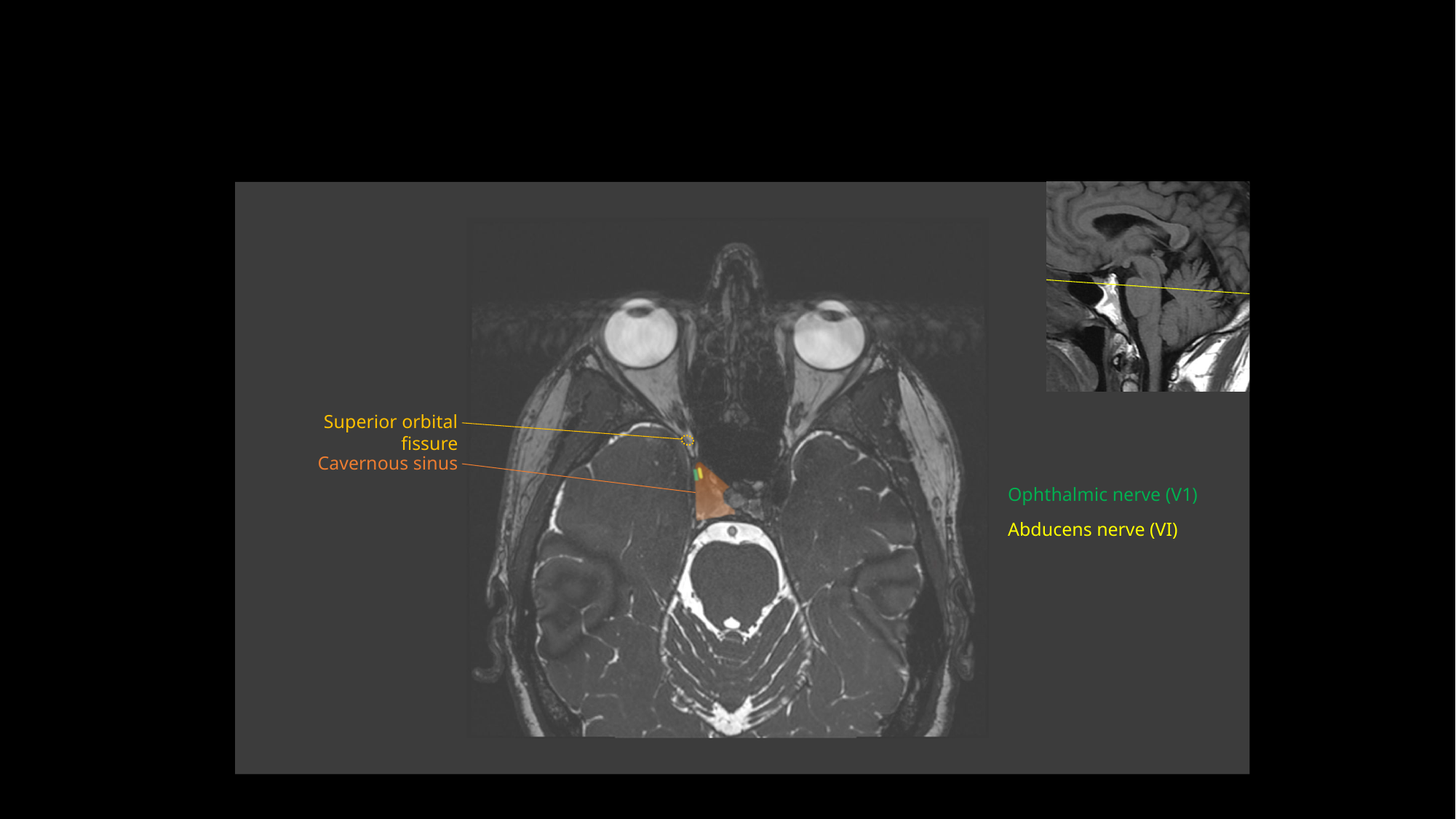

Superior orbital fissure
Cavernous sinus
Ophthalmic nerve (V1)
Abducens nerve (VI)

## Slide 129
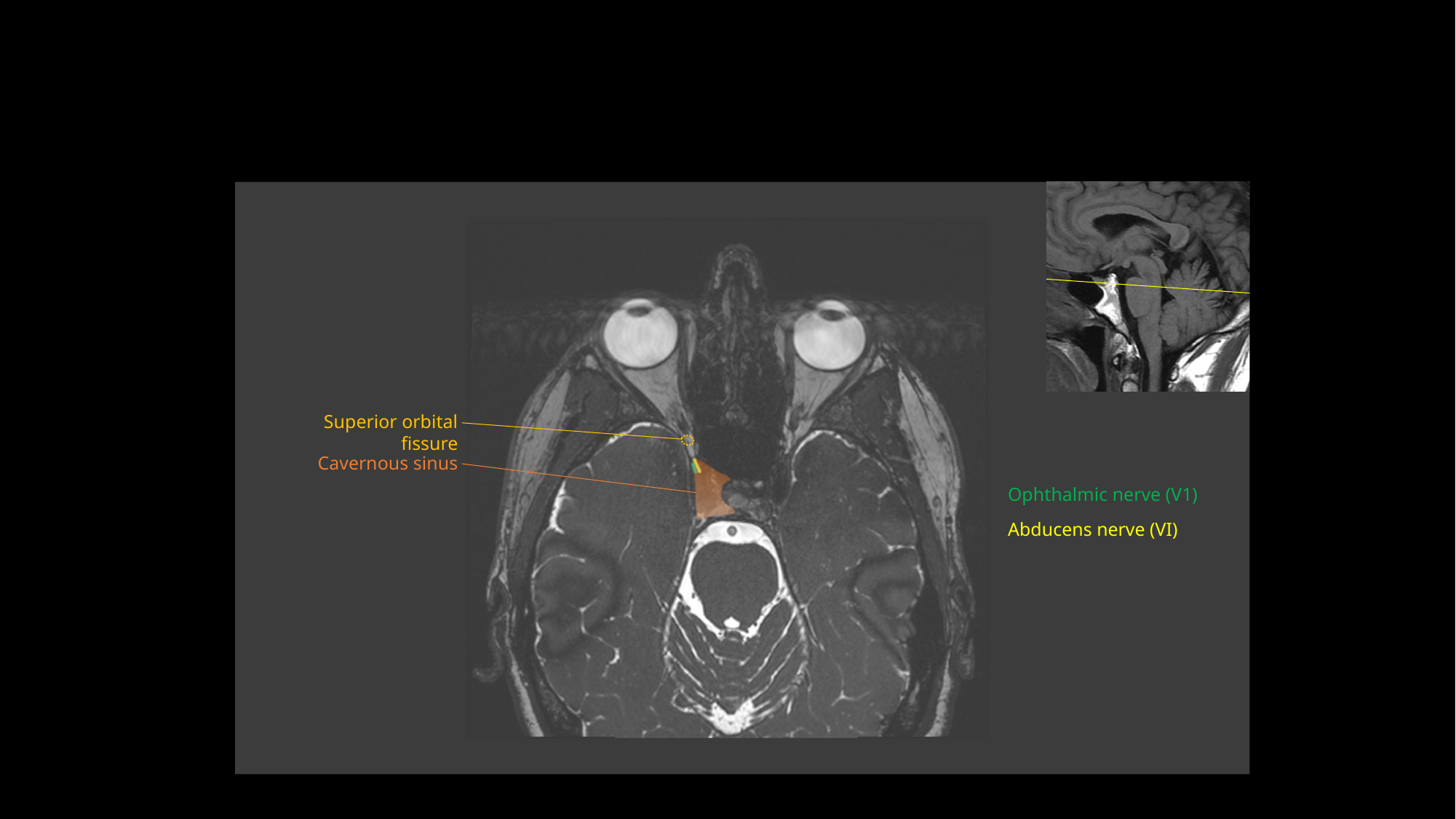

Superior orbital fissure
Cavernous sinus
Ophthalmic nerve (V1)
Abducens nerve (VI)

## Slide 130
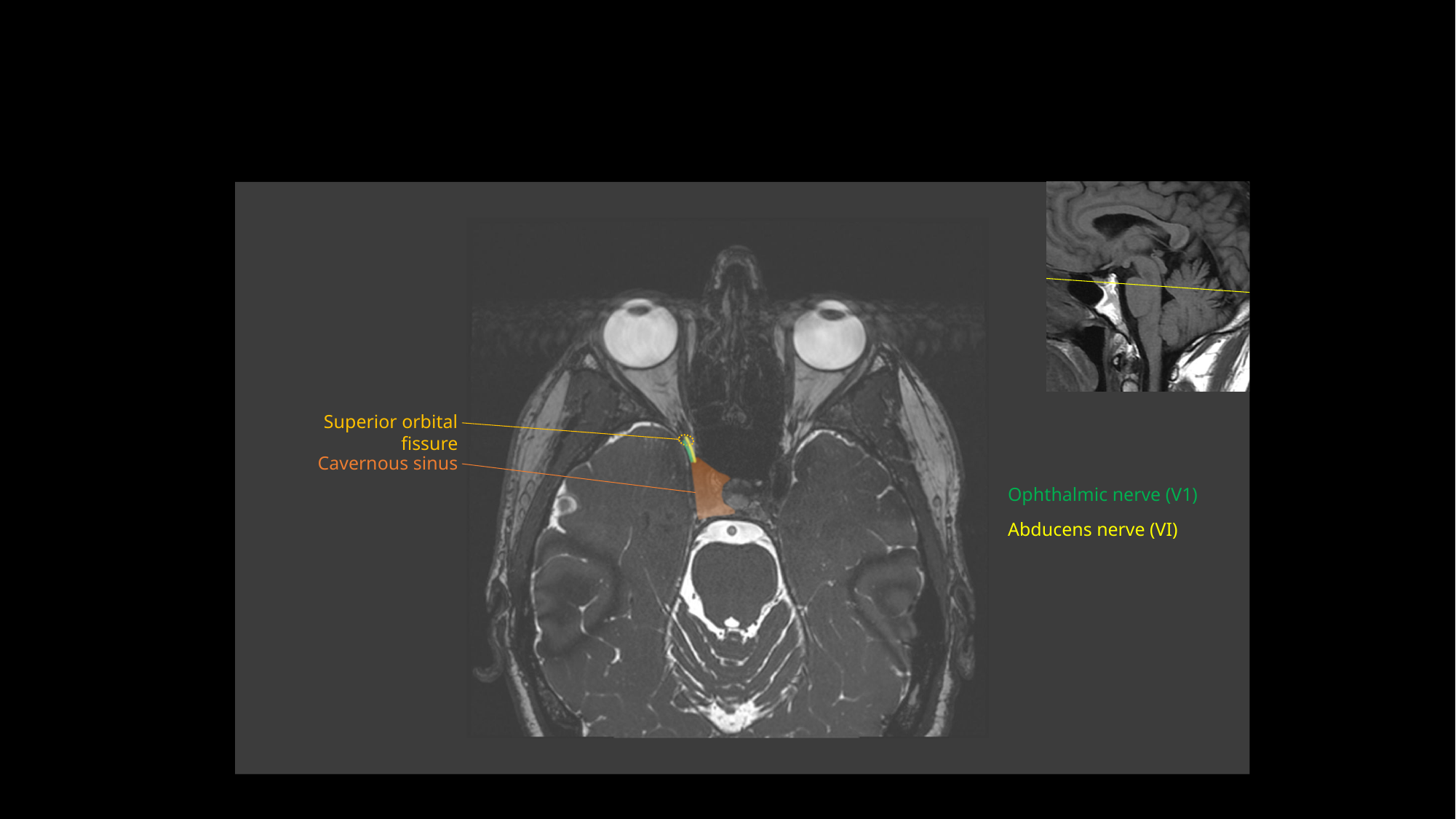

Superior orbital fissure
Cavernous sinus
Ophthalmic nerve (V1)
Abducens nerve (VI)

## Slide 131
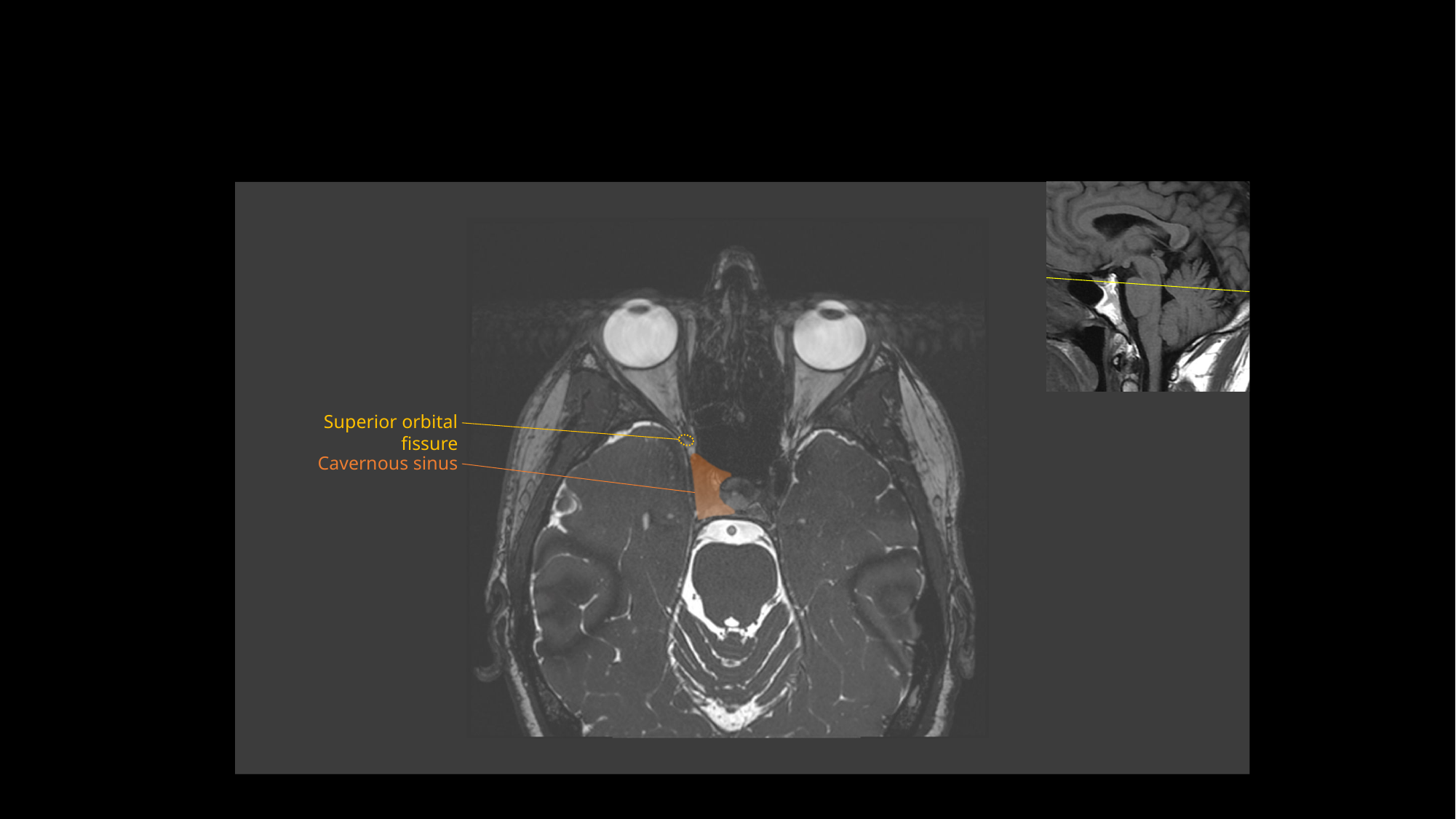

Superior orbital fissure
Cavernous sinus

## Slide 132
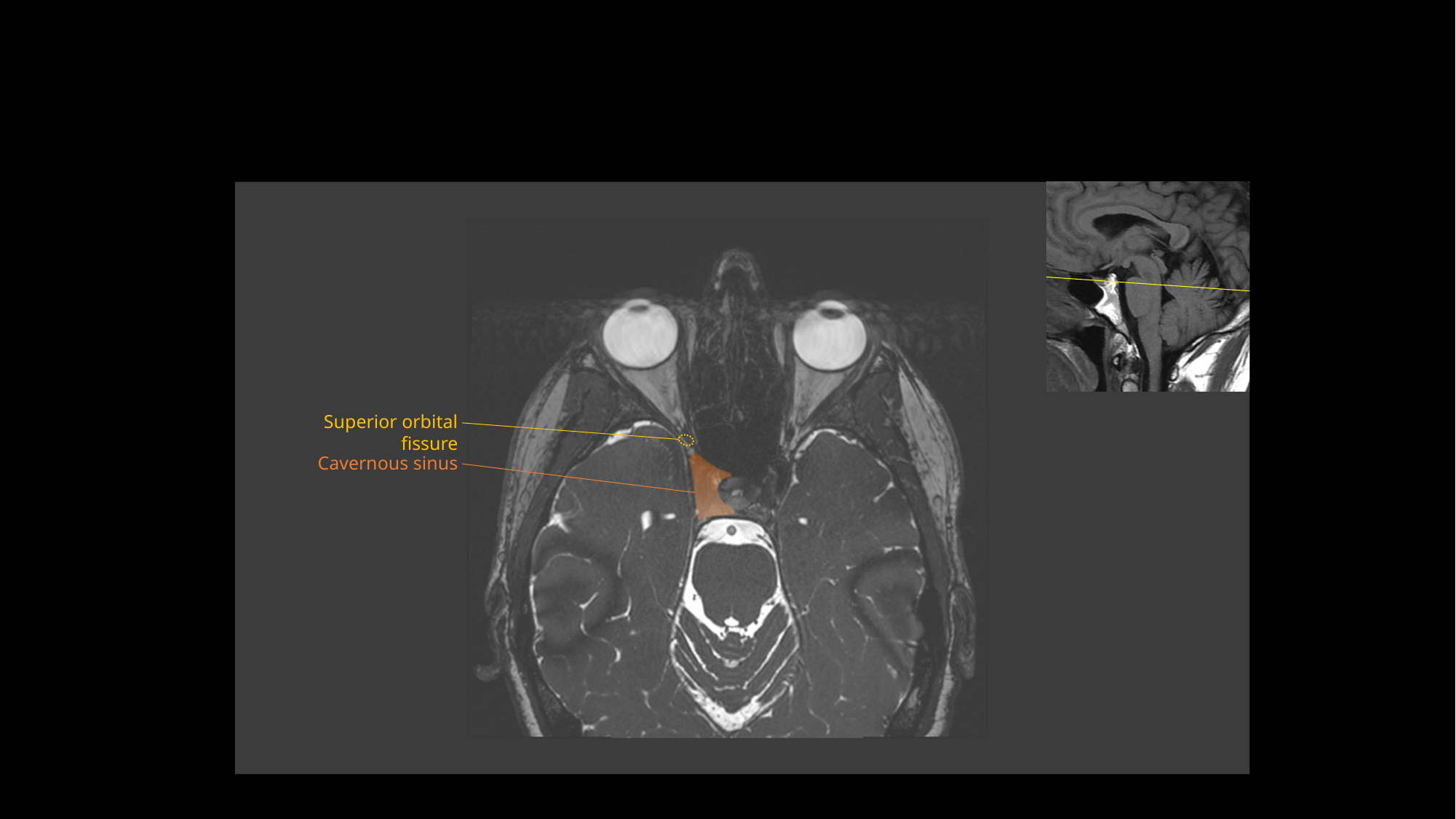

Superior orbital fissure
Cavernous sinus

## Slide 133
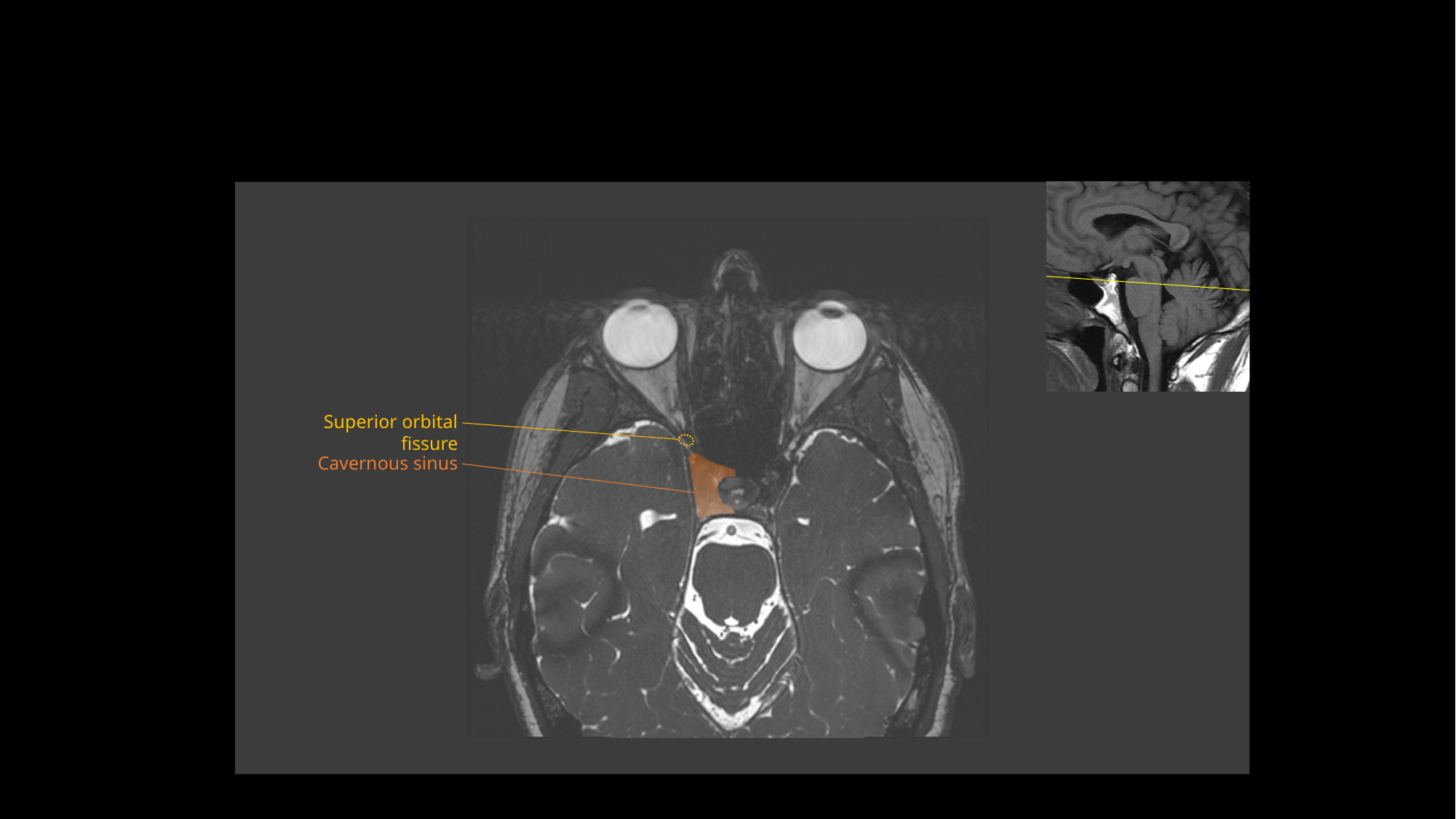

Superior orbital fissure
Cavernous sinus

## Slide 134
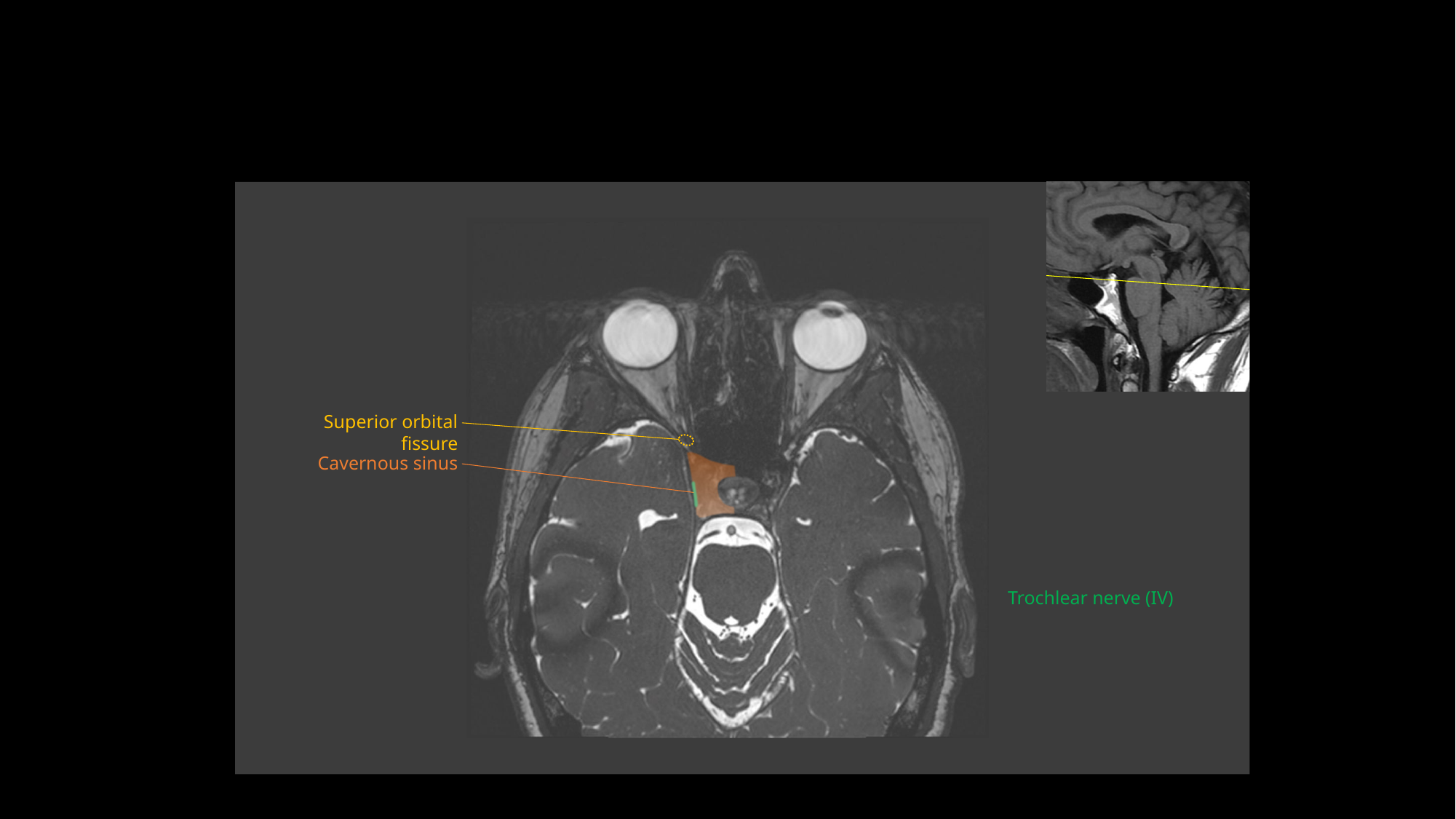

Superior orbital fissure
Cavernous sinus
Trochlear nerve (IV)

## Slide 135
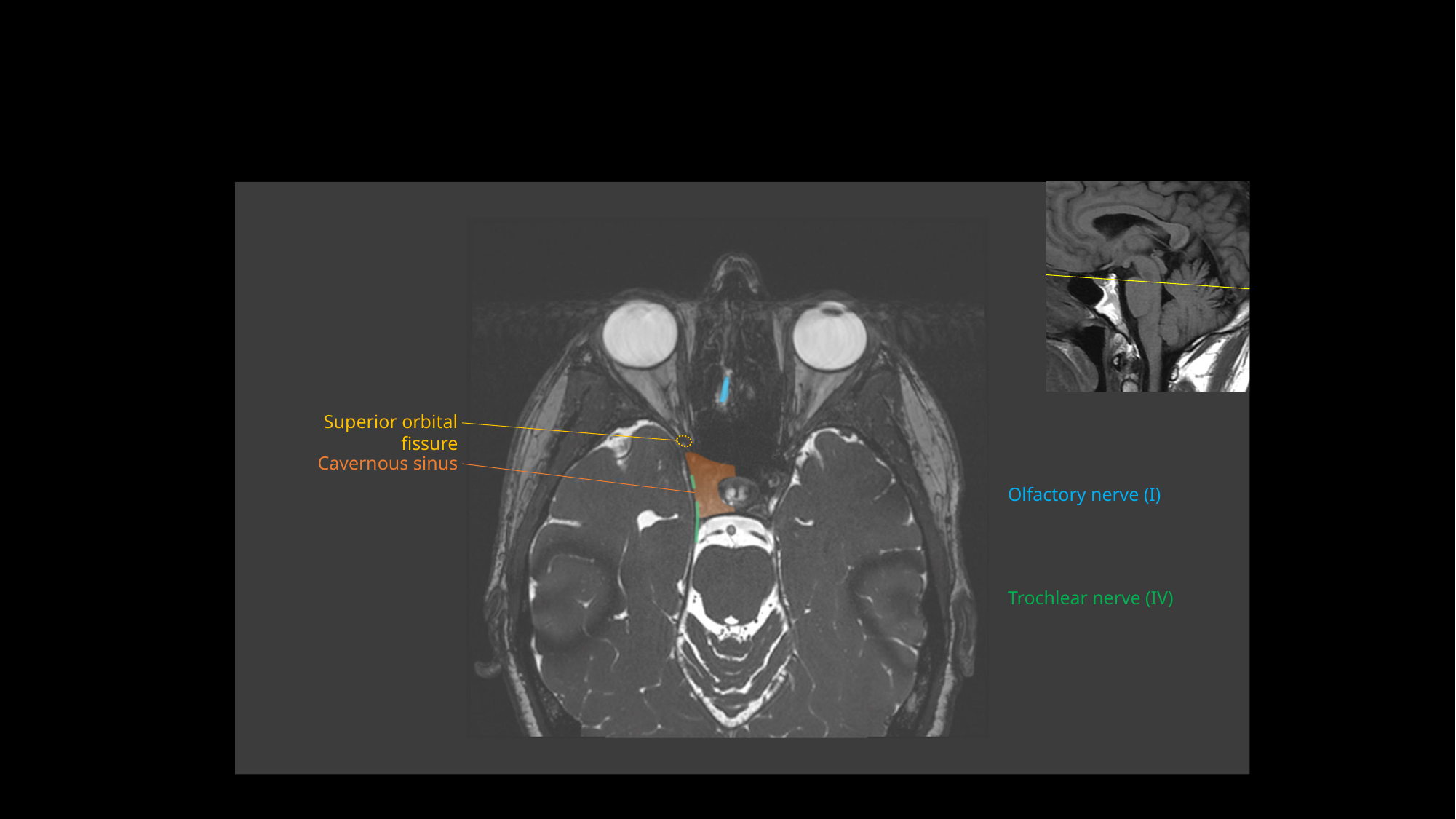

Superior orbital fissure
Cavernous sinus
Olfactory nerve (I)
Trochlear nerve (IV)

## Slide 136
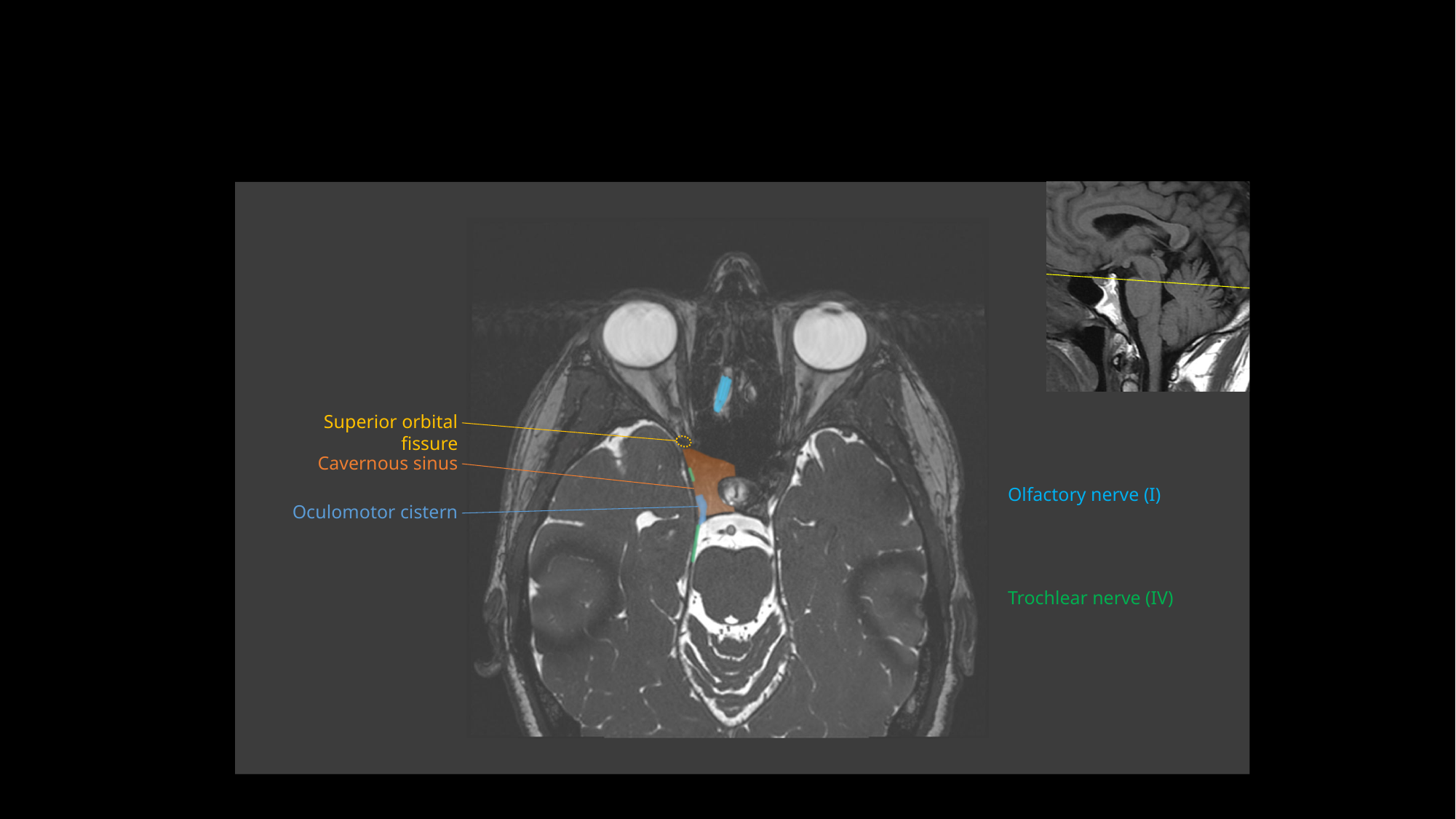

Superior orbital fissure
Cavernous sinus
Olfactory nerve (I)
Oculomotor cistern
Trochlear nerve (IV)

## Slide 137
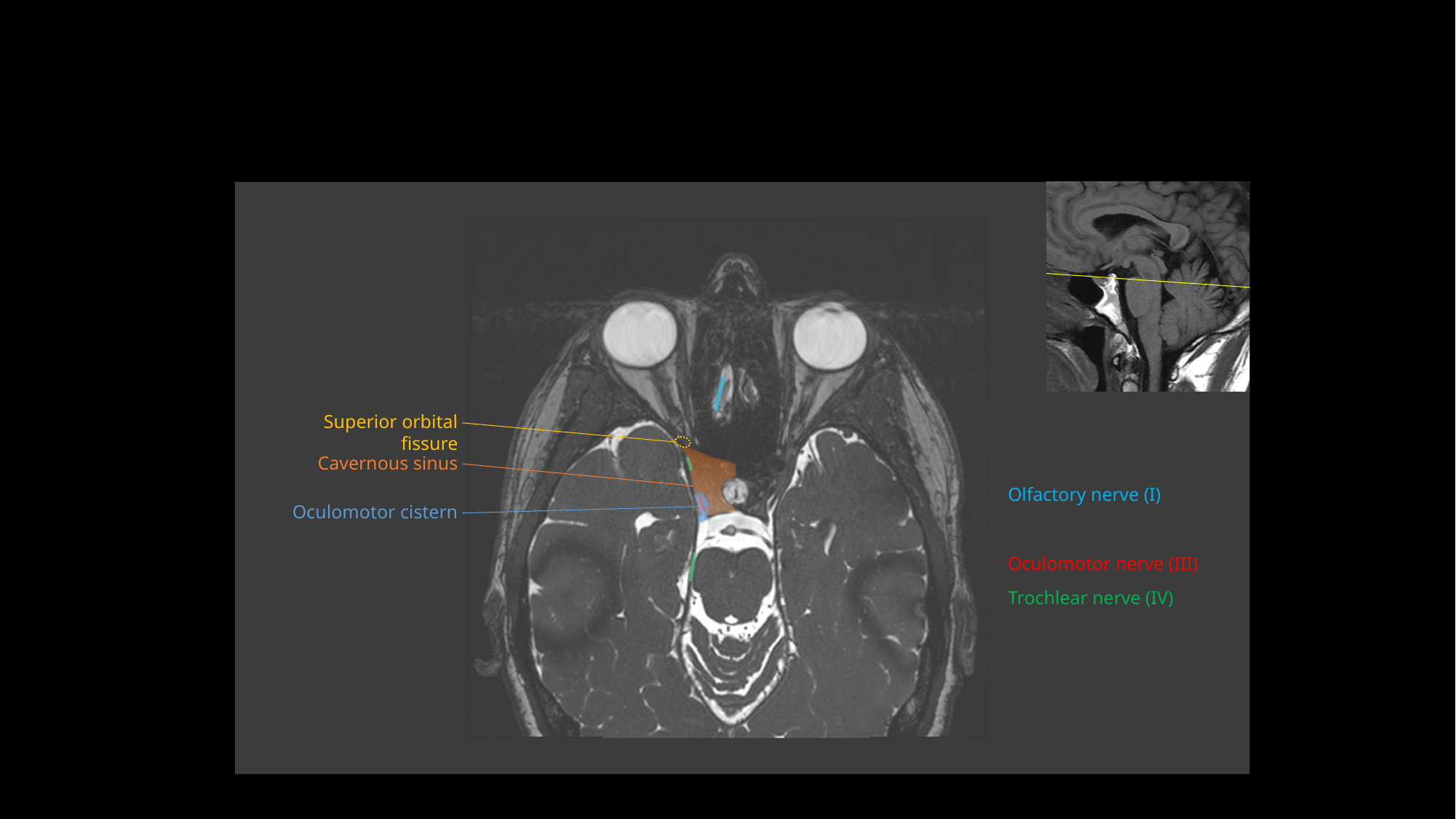

Superior orbital fissure
Cavernous sinus
Olfactory nerve (I)
Oculomotor cistern
Oculomotor nerve (III)
Trochlear nerve (IV)

## Slide 138
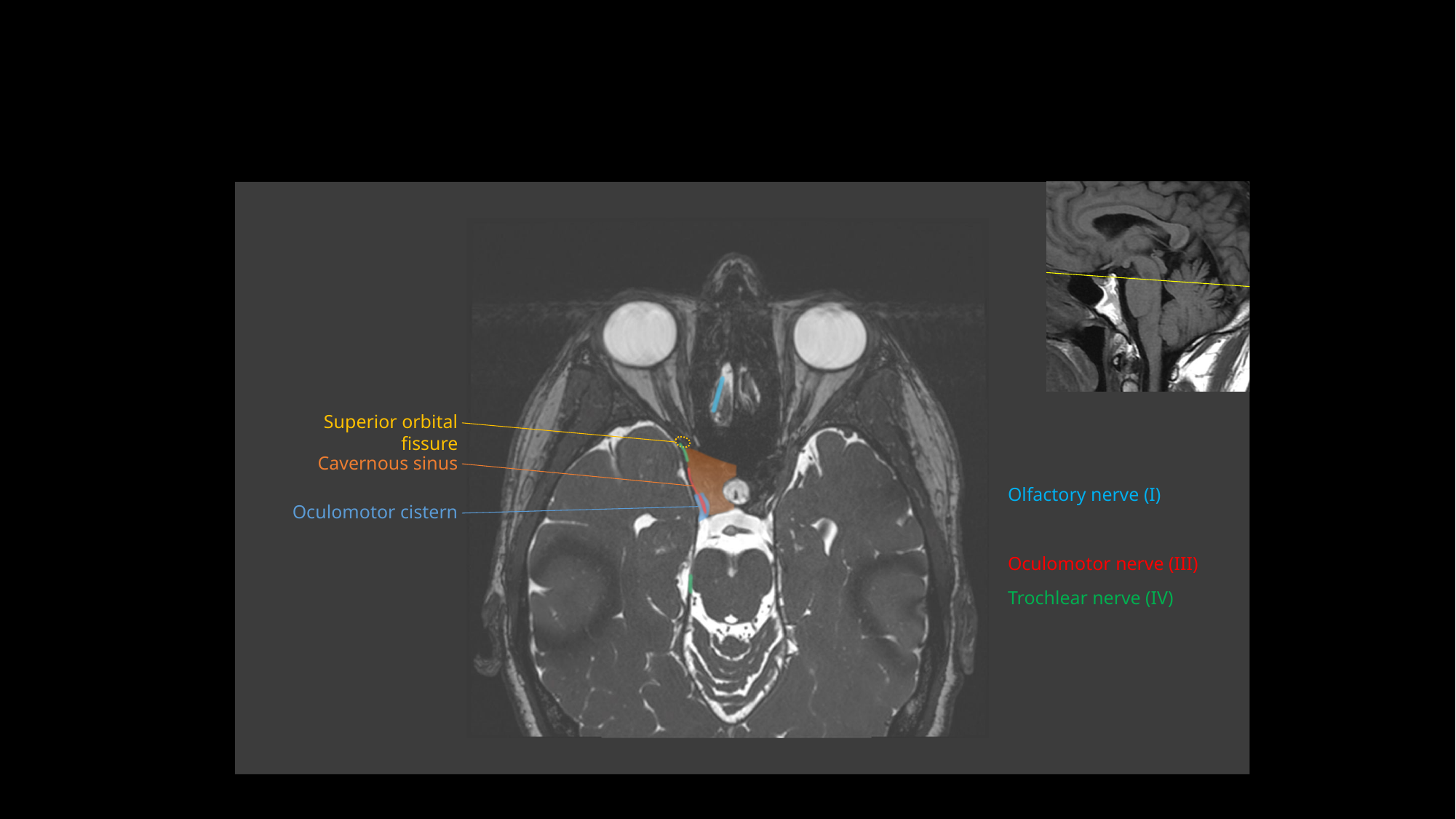

Superior orbital fissure
Cavernous sinus
Olfactory nerve (I)
Oculomotor cistern
Oculomotor nerve (III)
Trochlear nerve (IV)

## Slide 139
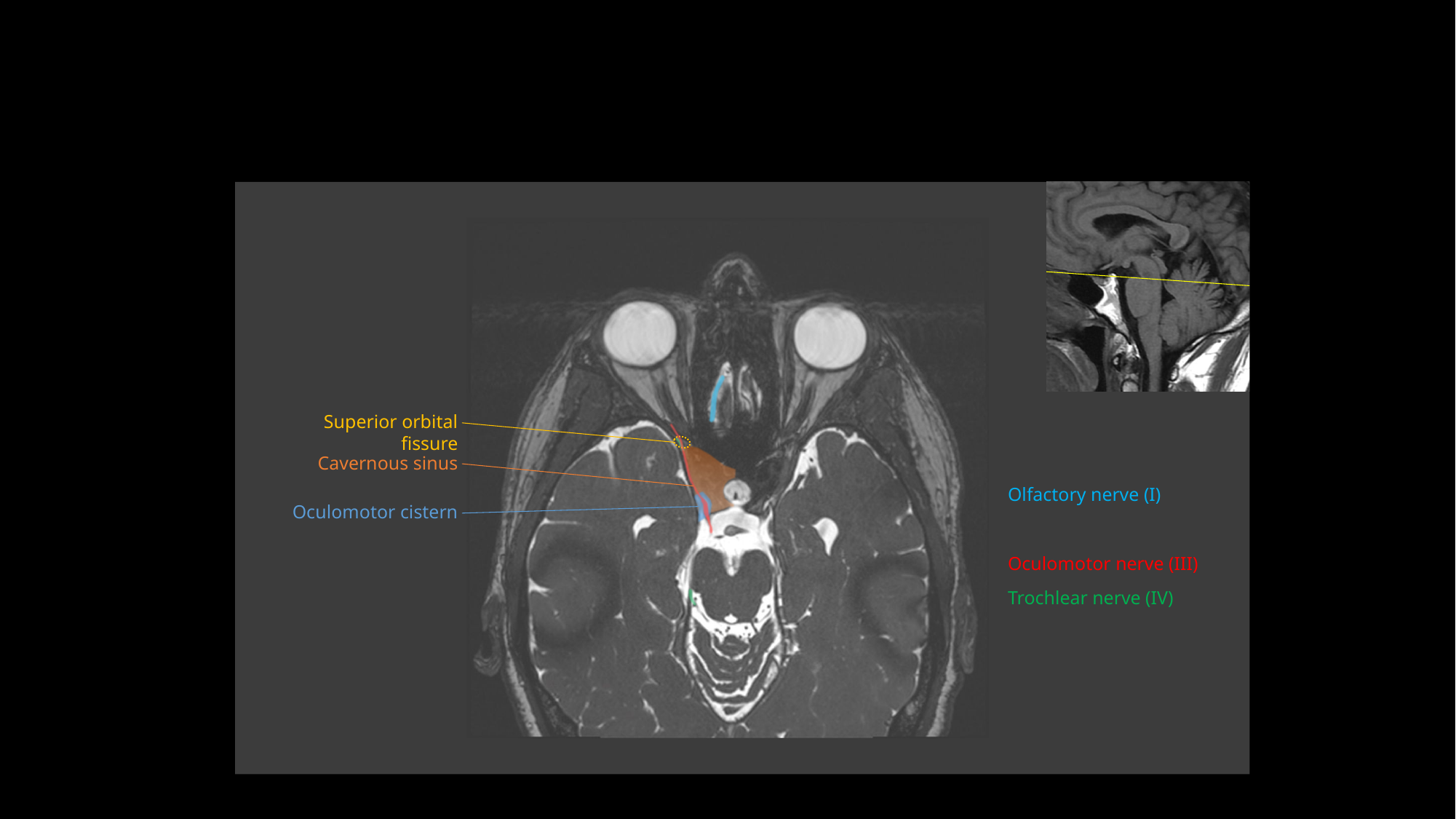

Superior orbital fissure
Cavernous sinus
Olfactory nerve (I)
Oculomotor cistern
Oculomotor nerve (III)
Trochlear nerve (IV)

## Slide 140
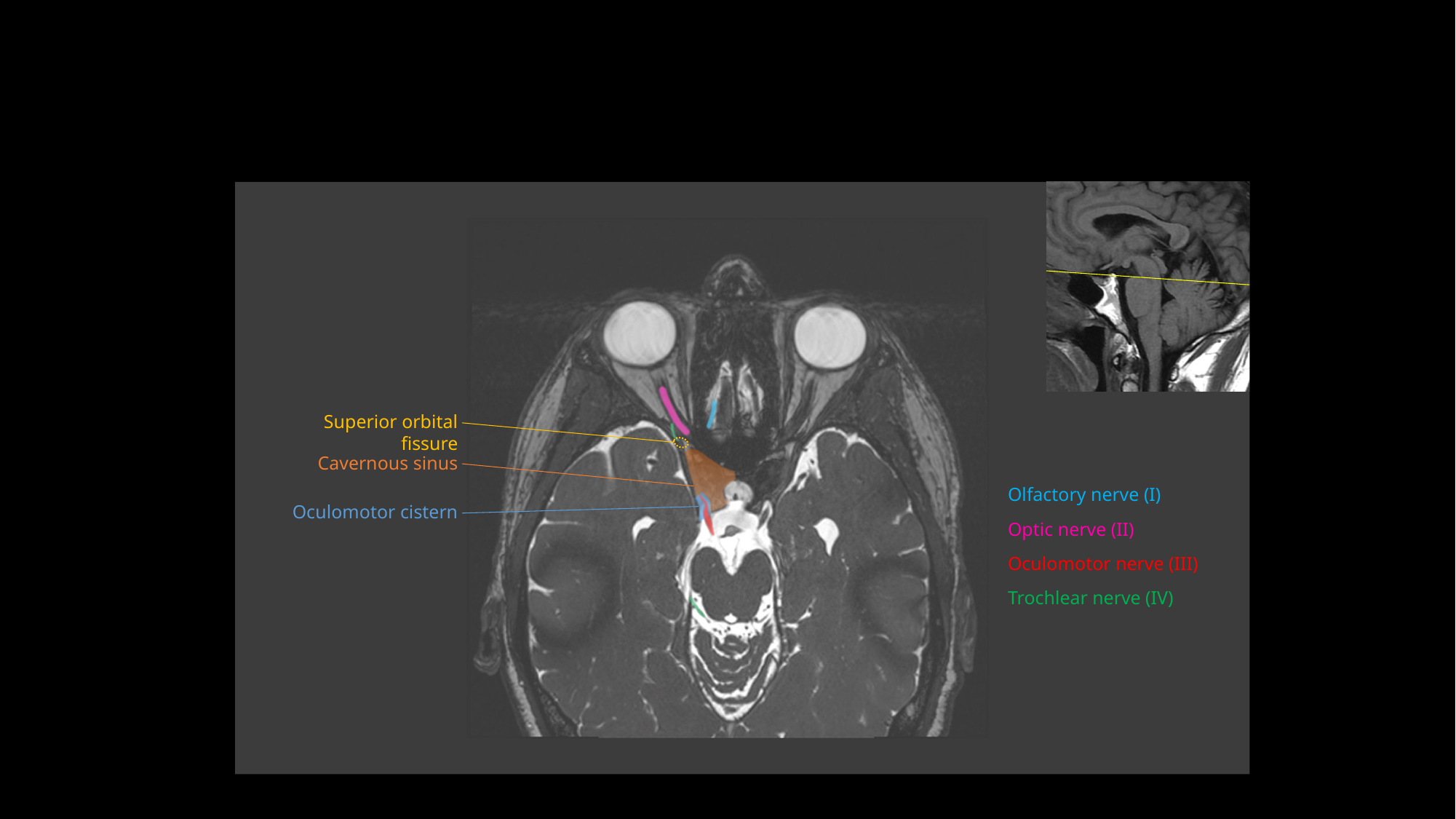

Superior orbital fissure
Cavernous sinus
Olfactory nerve (I)
Oculomotor cistern
Optic nerve (II)
Oculomotor nerve (III)
Trochlear nerve (IV)

## Slide 141
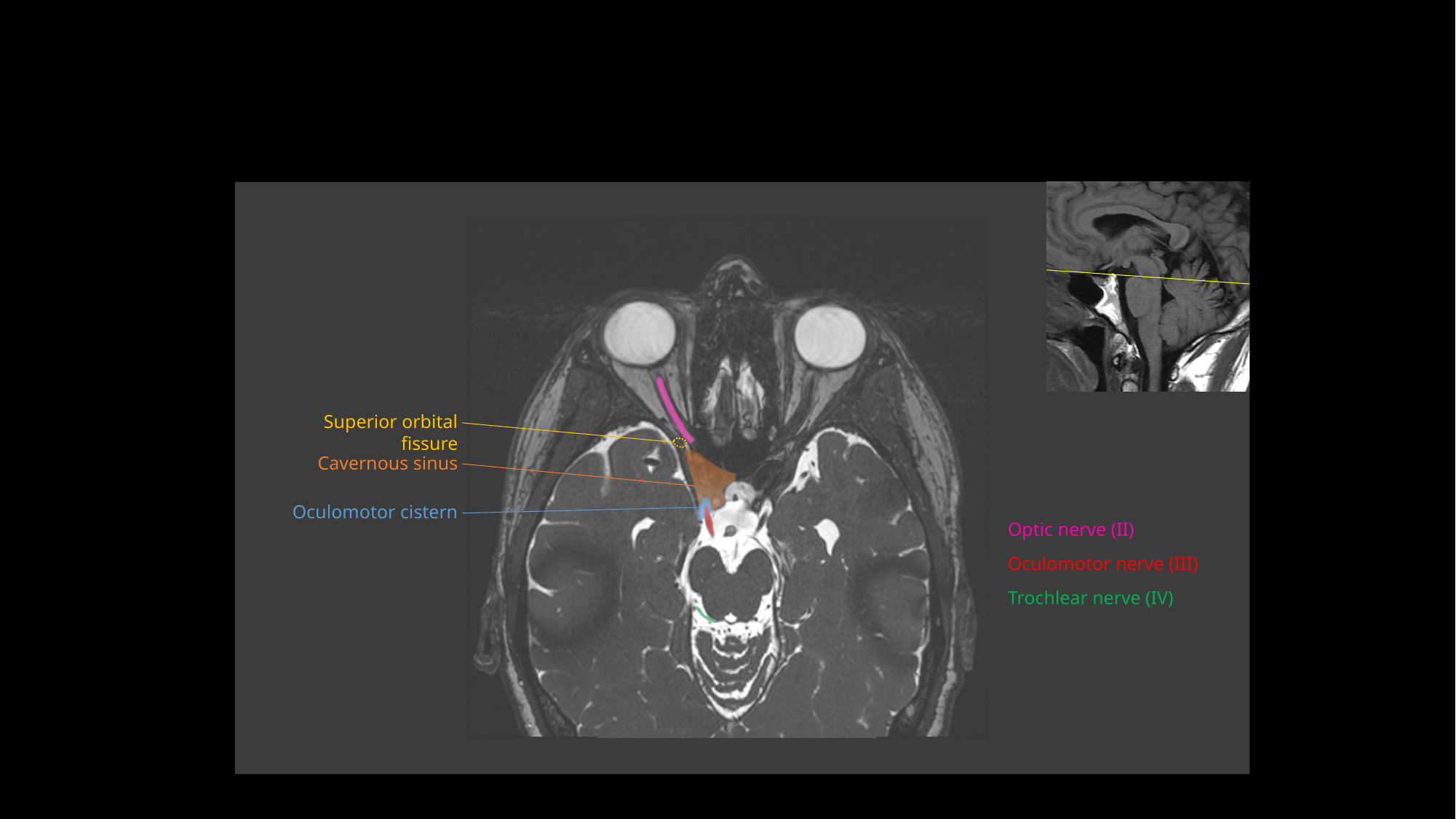

Superior orbital fissure
Cavernous sinus
Oculomotor cistern
Optic nerve (II)
Oculomotor nerve (III)
Trochlear nerve (IV)

## Slide 142
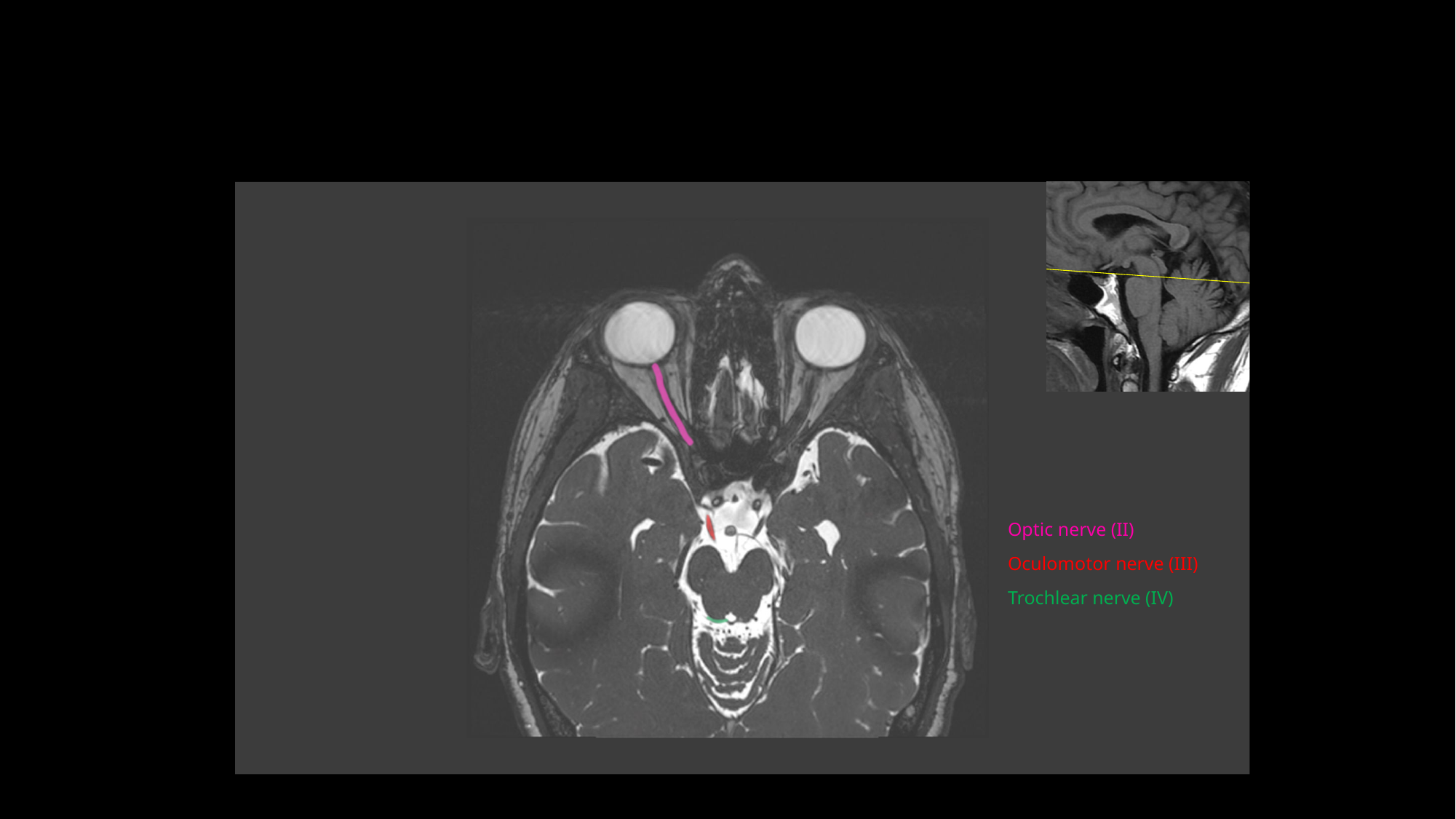

Optic nerve (II)
Oculomotor nerve (III)
Trochlear nerve (IV)

## Slide 143
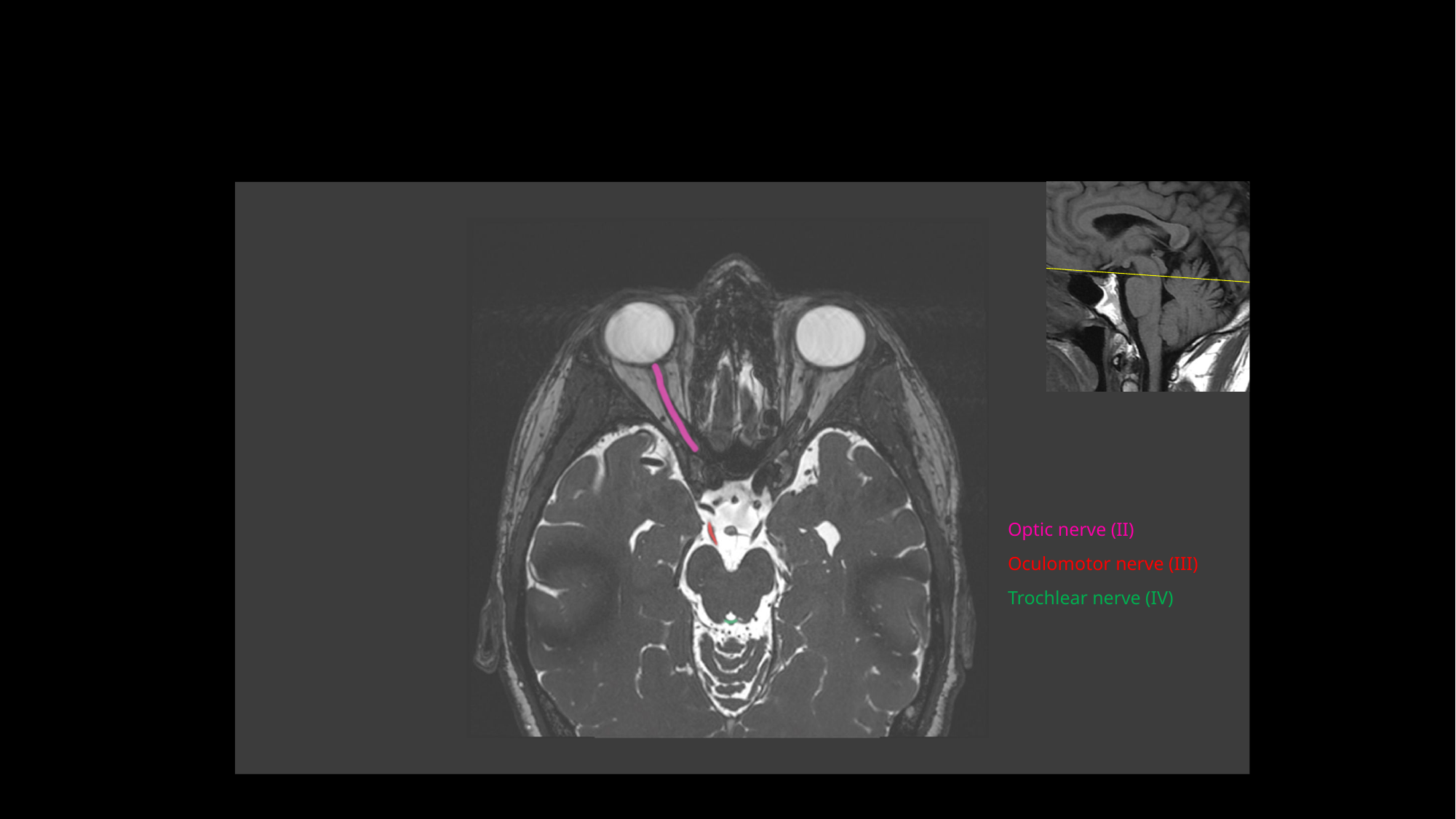

Optic nerve (II)
Oculomotor nerve (III)
Trochlear nerve (IV)

## Slide 144
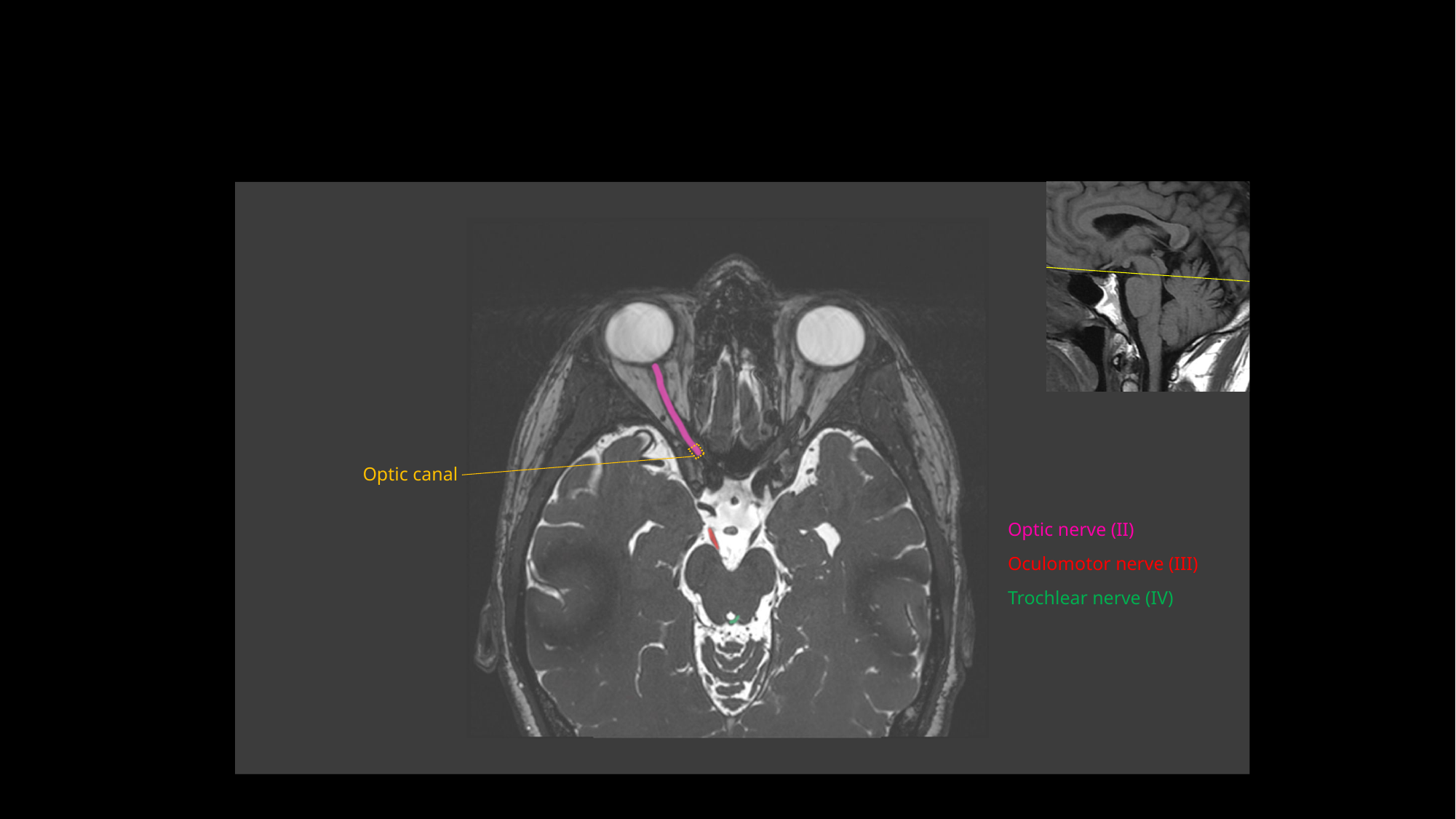

Optic canal
Optic nerve (II)
Oculomotor nerve (III)
Trochlear nerve (IV)

## Slide 145
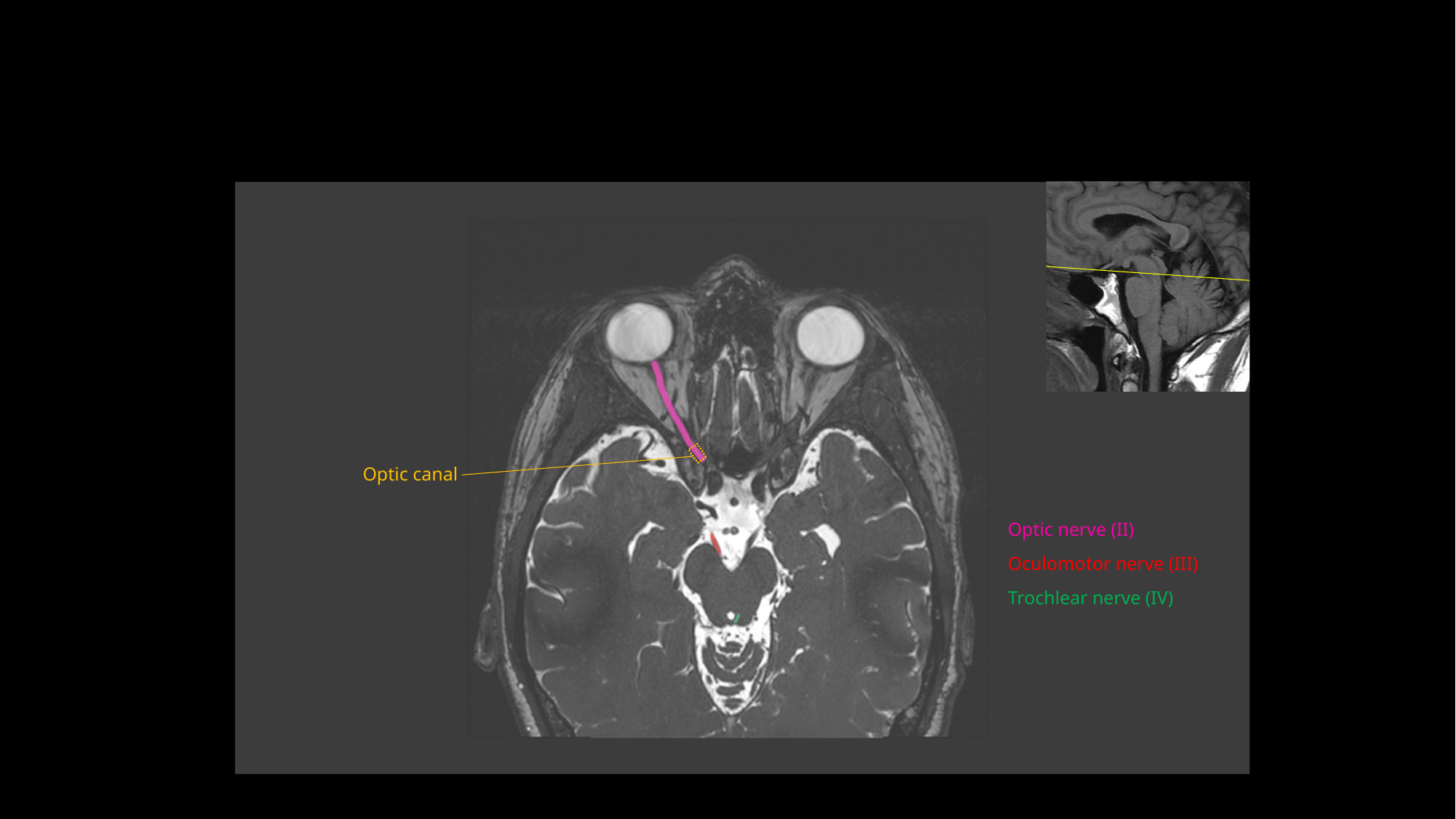

Optic canal
Optic nerve (II)
Oculomotor nerve (III)
Trochlear nerve (IV)

## Slide 146
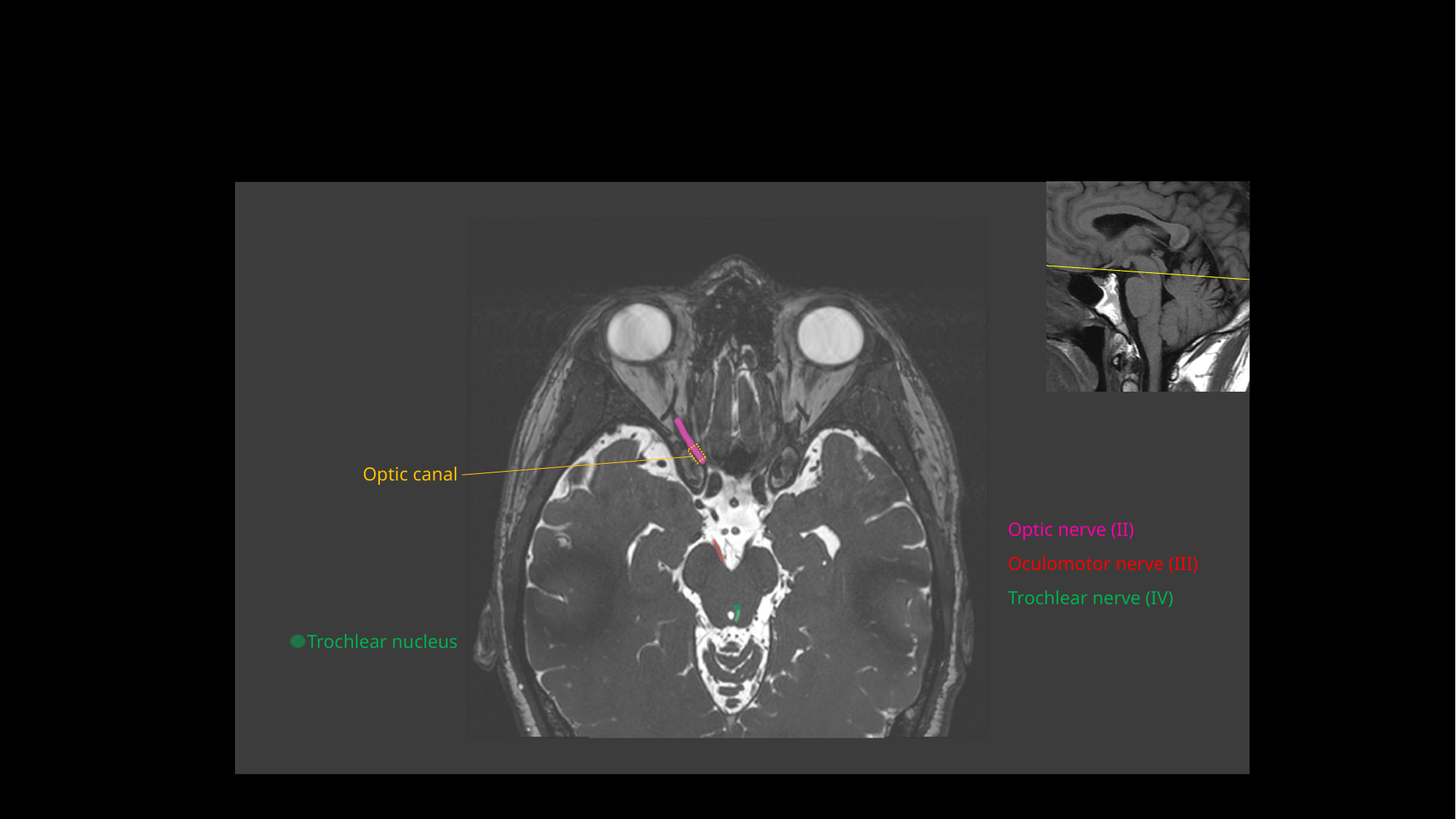

Optic canal
Optic nerve (II)
Oculomotor nerve (III)
Trochlear nerve (IV)
Trochlear nucleus

## Slide 147
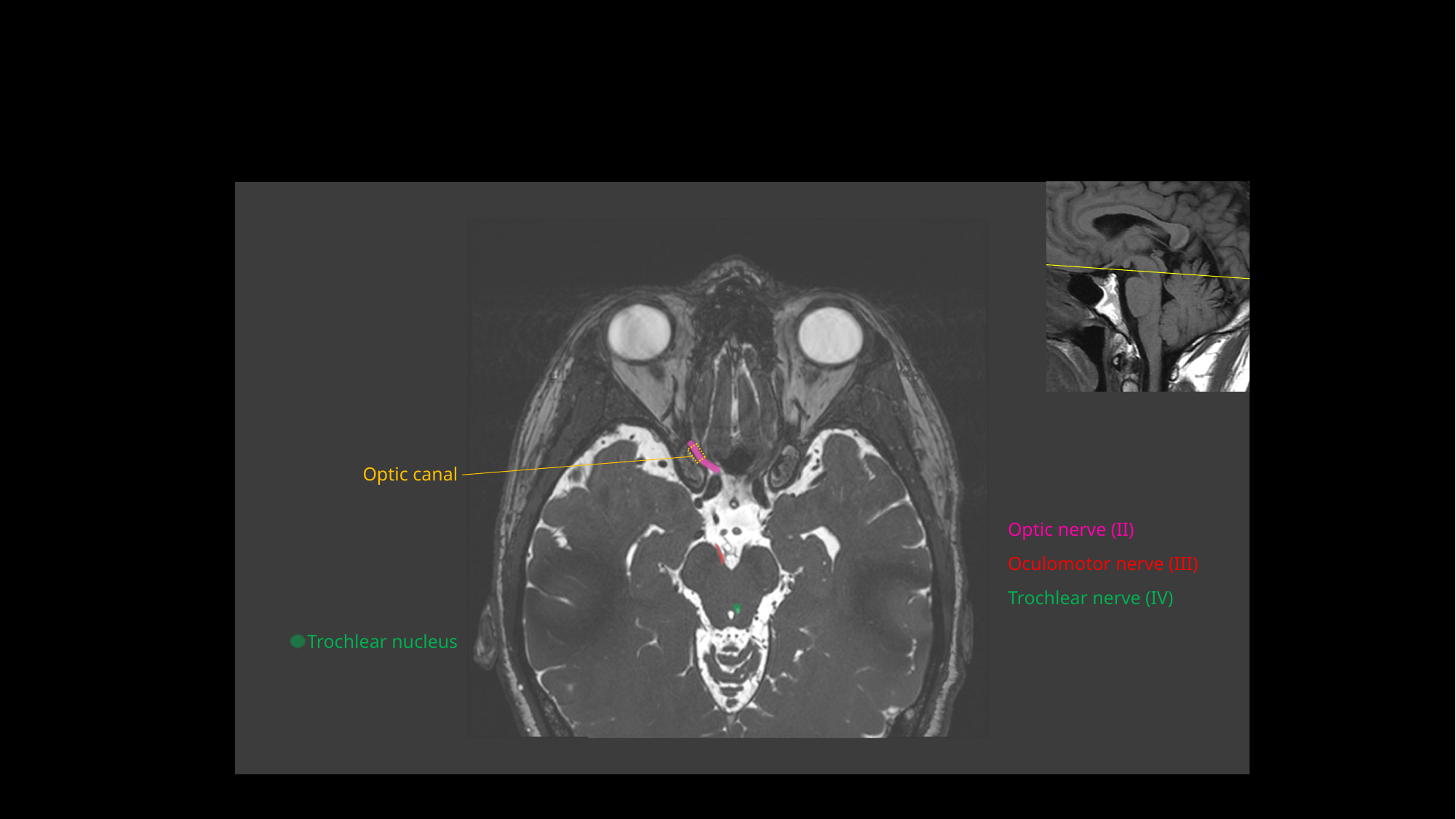

Optic canal
Optic nerve (II)
Oculomotor nerve (III)
Trochlear nerve (IV)
Trochlear nucleus

## Slide 148
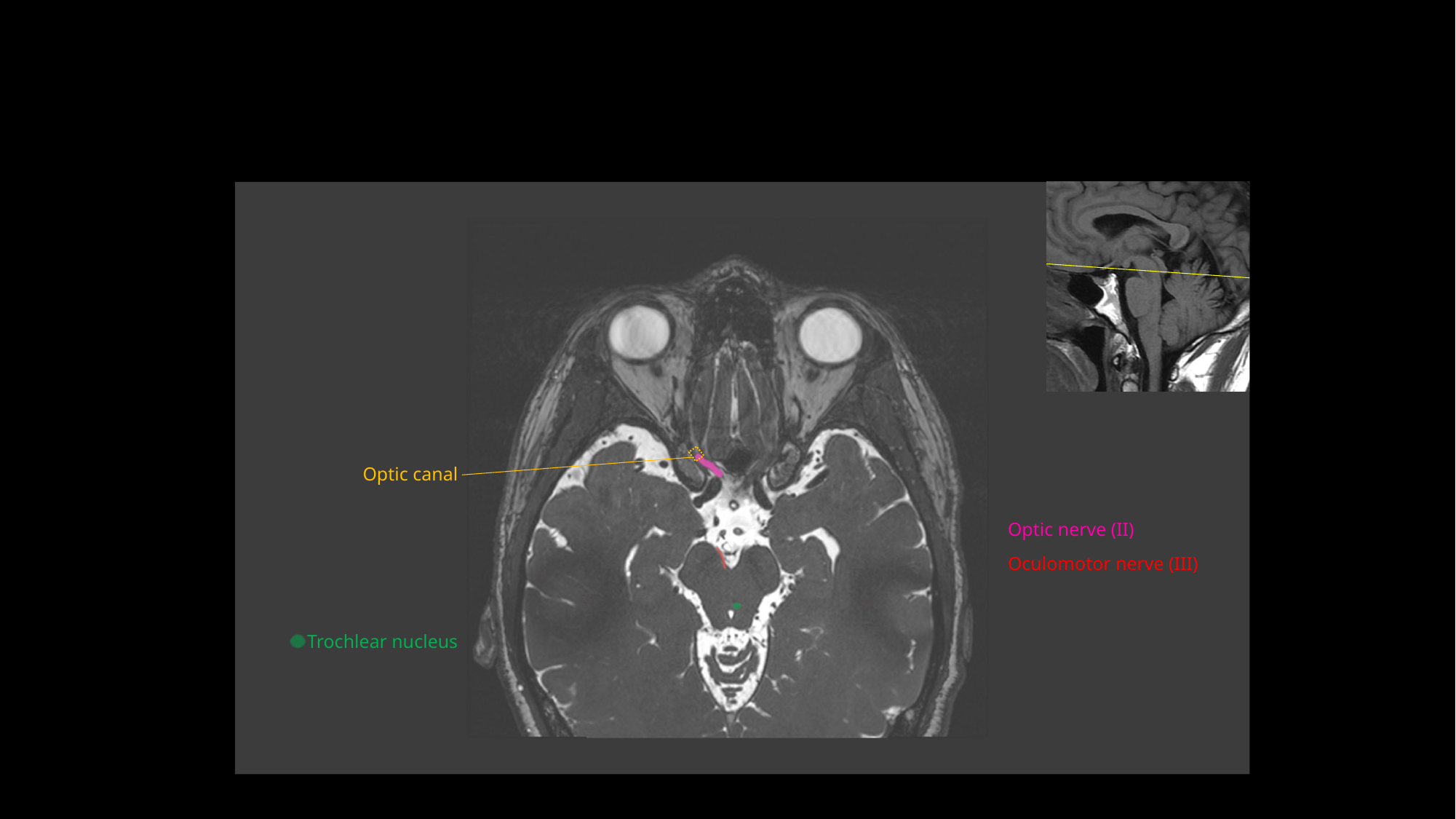

Optic canal
Optic nerve (II)
Oculomotor nerve (III)
Trochlear nucleus

## Slide 149
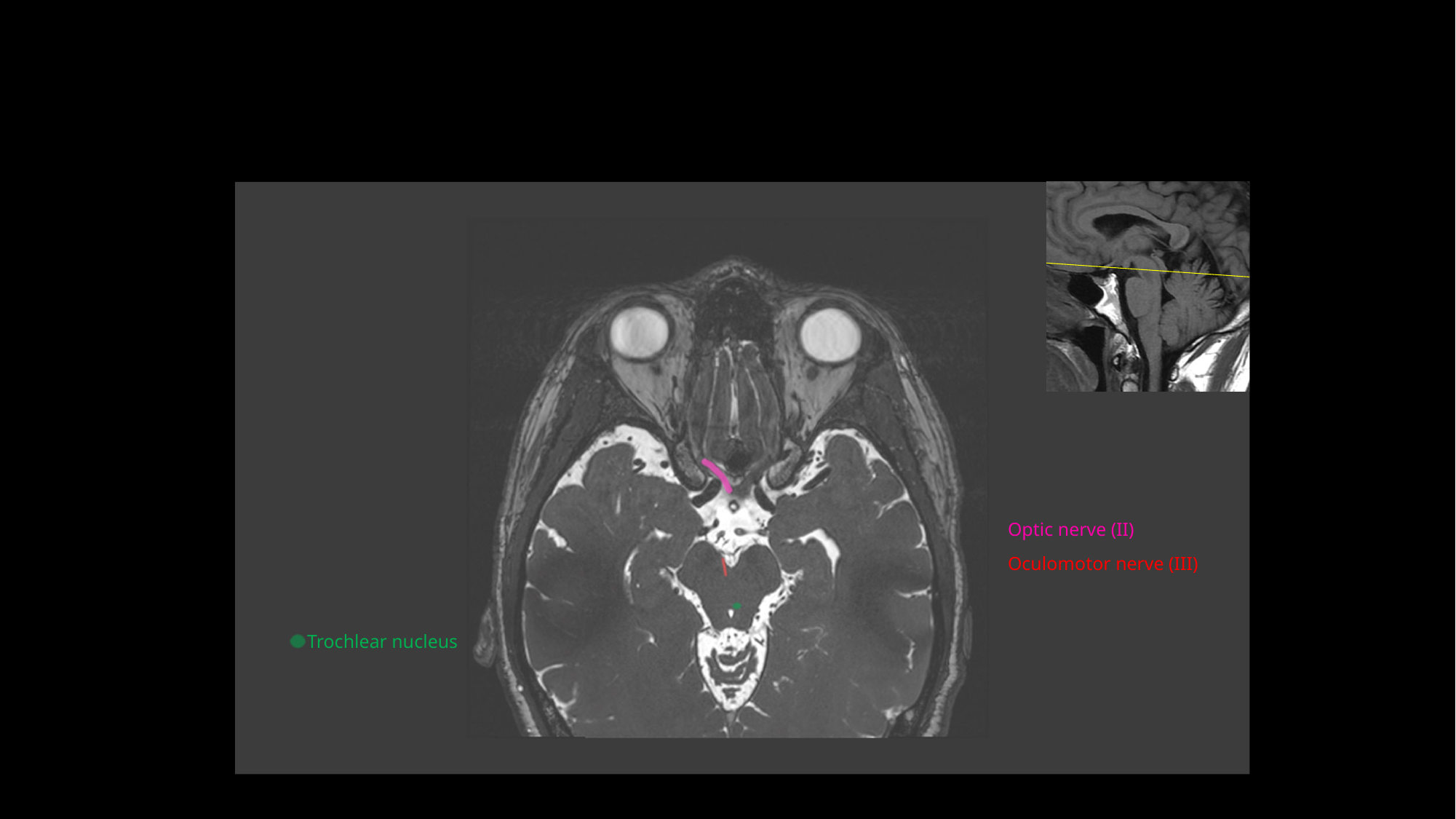

Optic nerve (II)
Oculomotor nerve (III)
Trochlear nucleus

## Slide 150
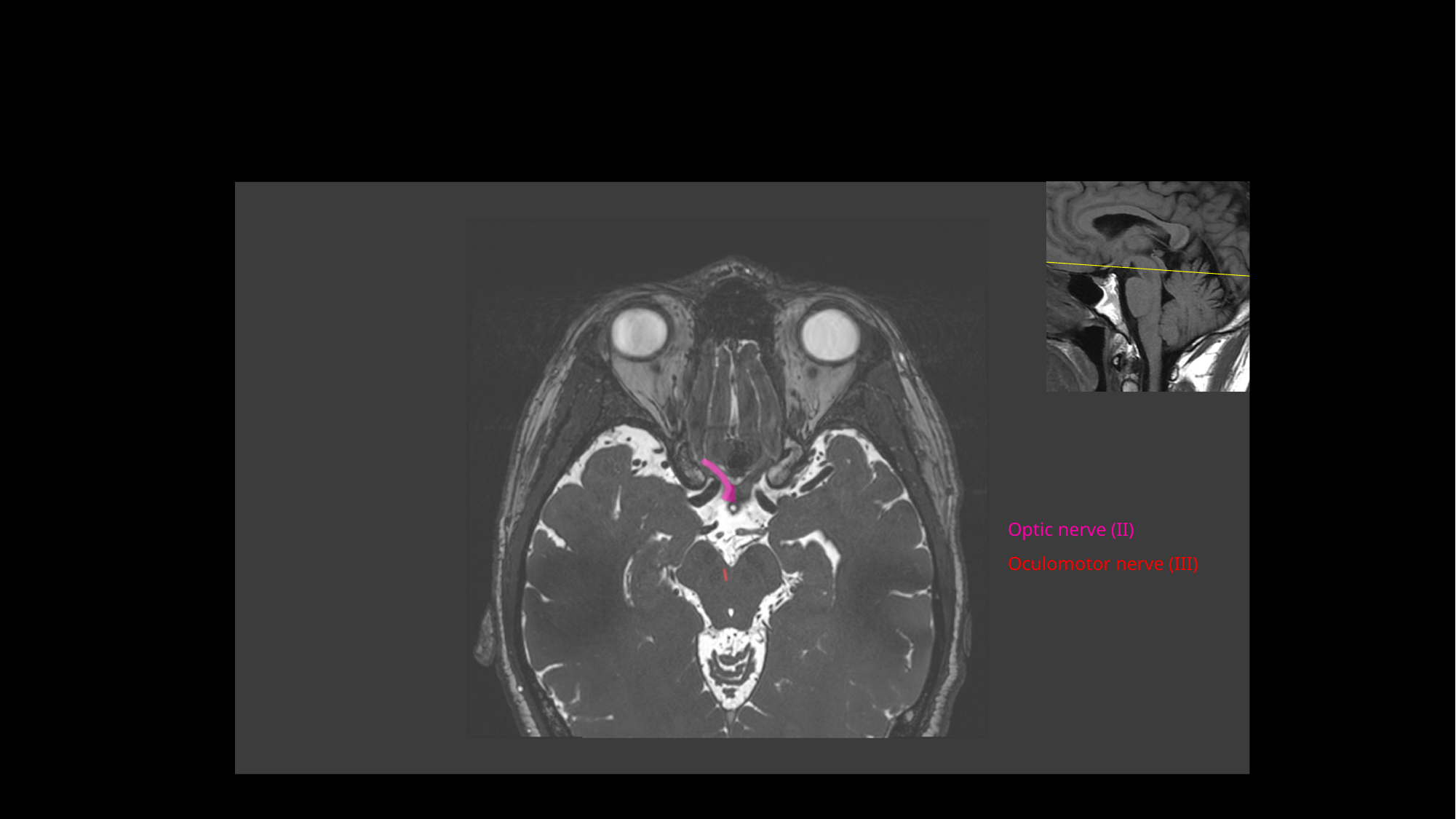

Optic nerve (II)
Oculomotor nerve (III)

## Slide 151
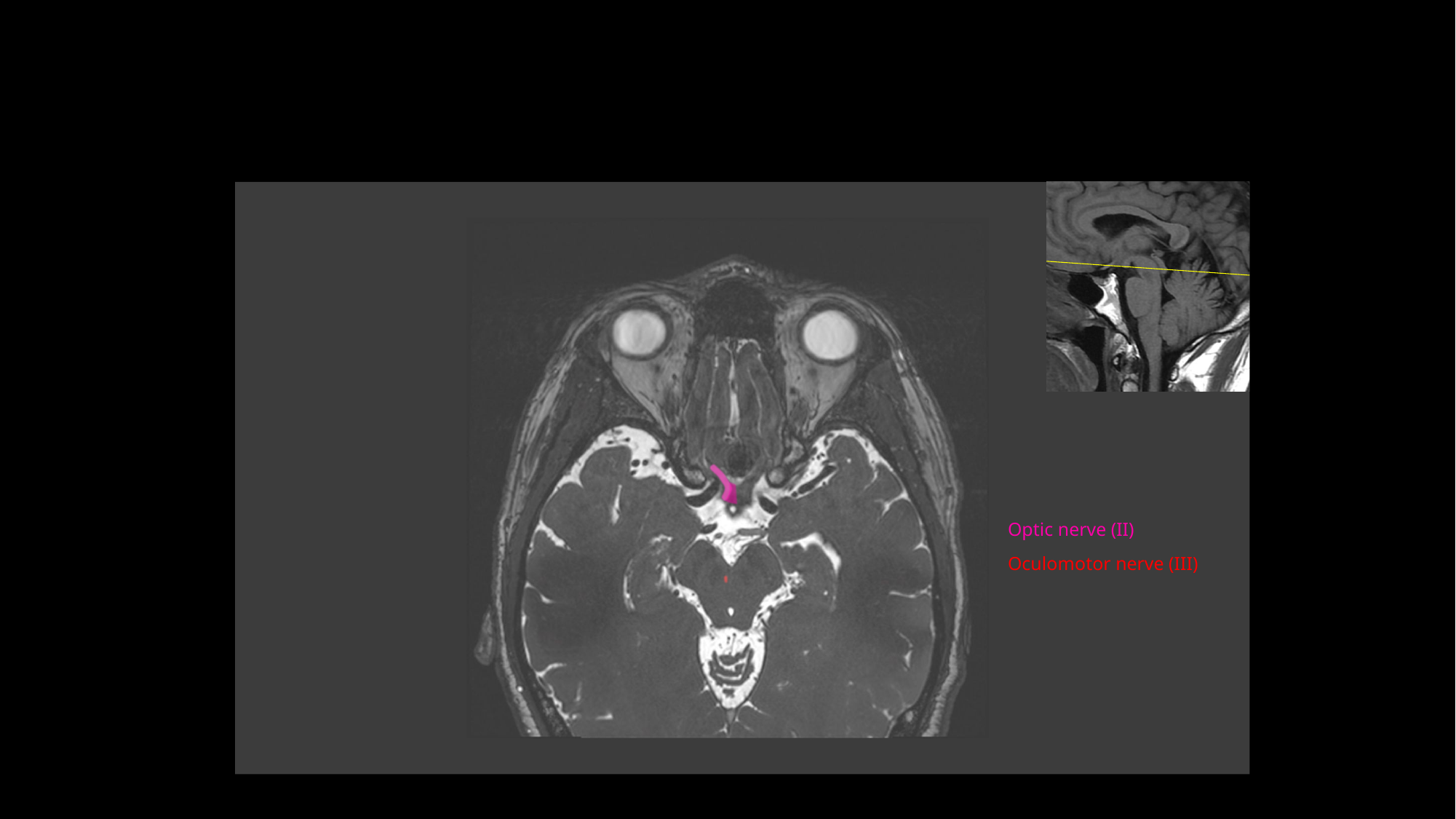

Optic nerve (II)
Oculomotor nerve (III)

## Slide 152
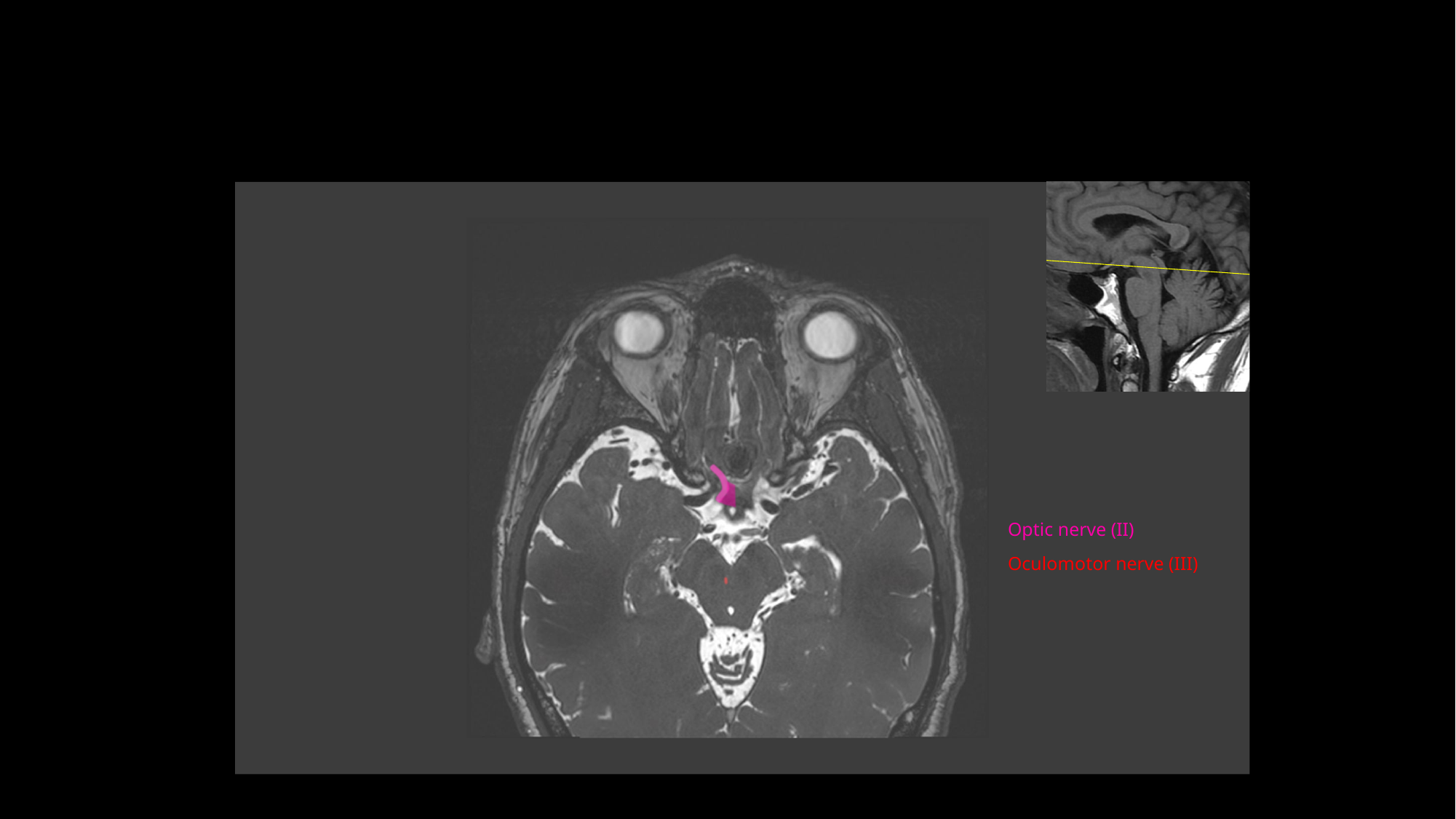

Optic nerve (II)
Oculomotor nerve (III)

## Slide 153
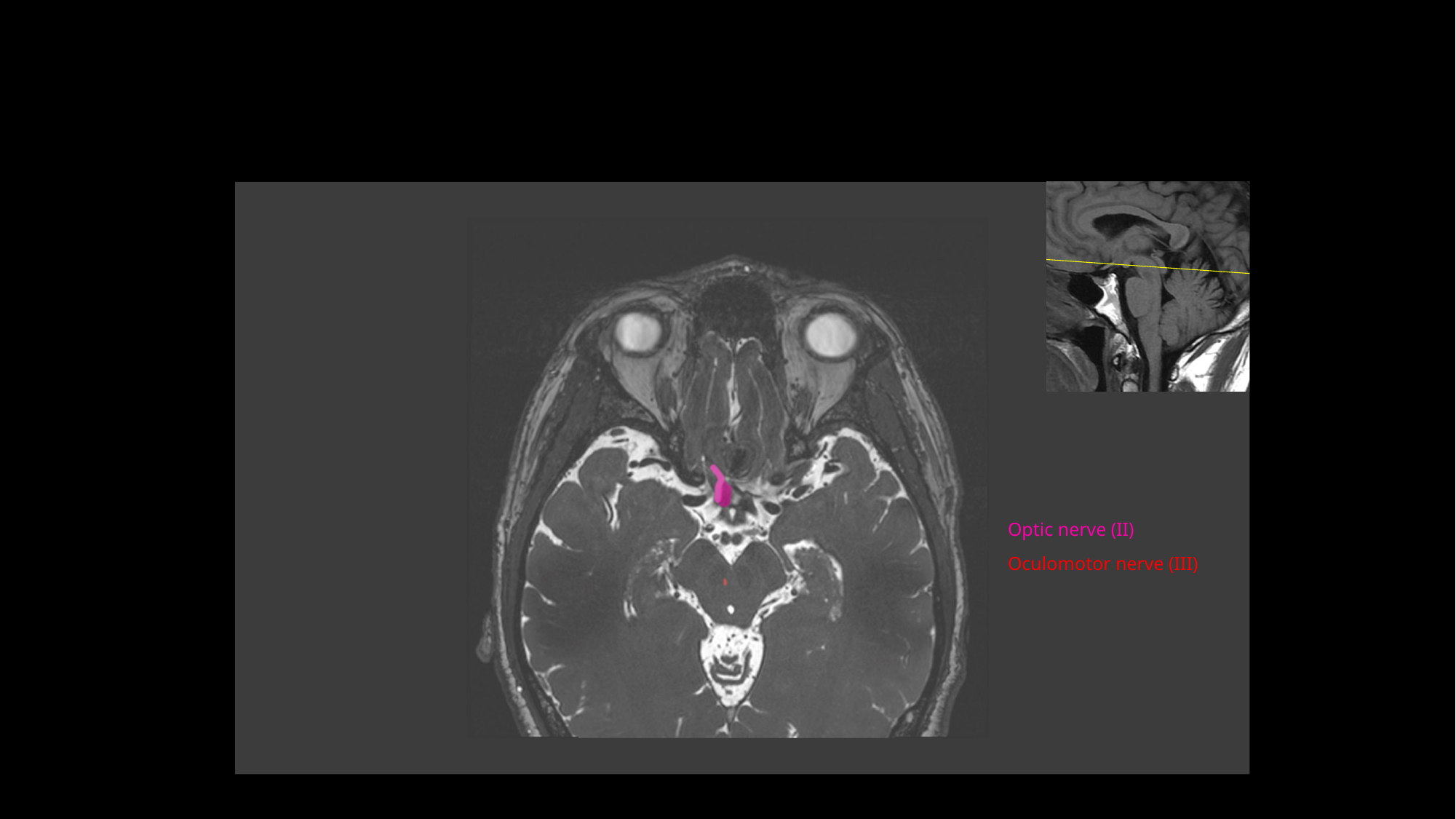

Optic nerve (II)
Oculomotor nerve (III)

## Slide 154
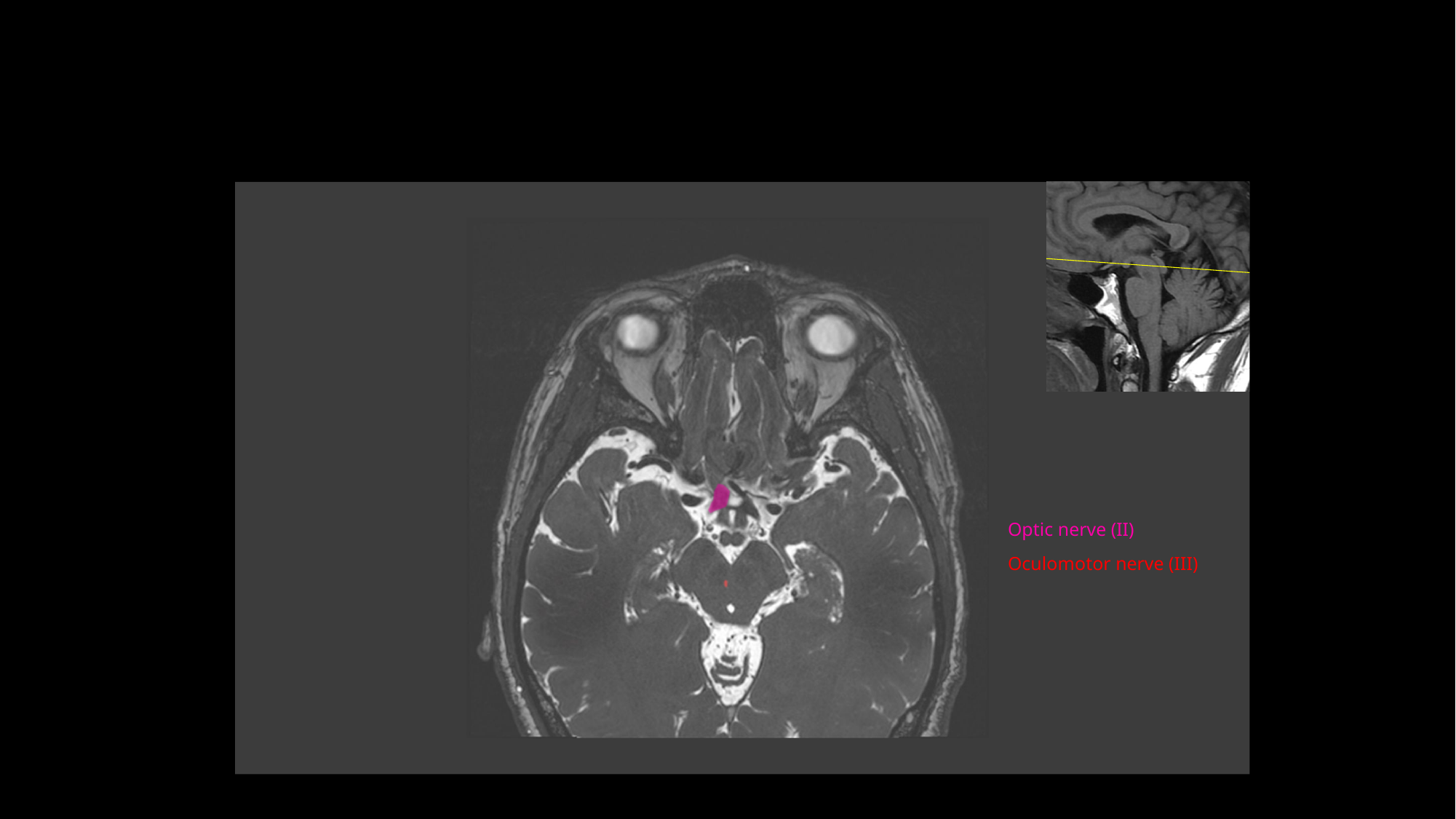

Optic nerve (II)
Oculomotor nerve (III)

## Slide 155
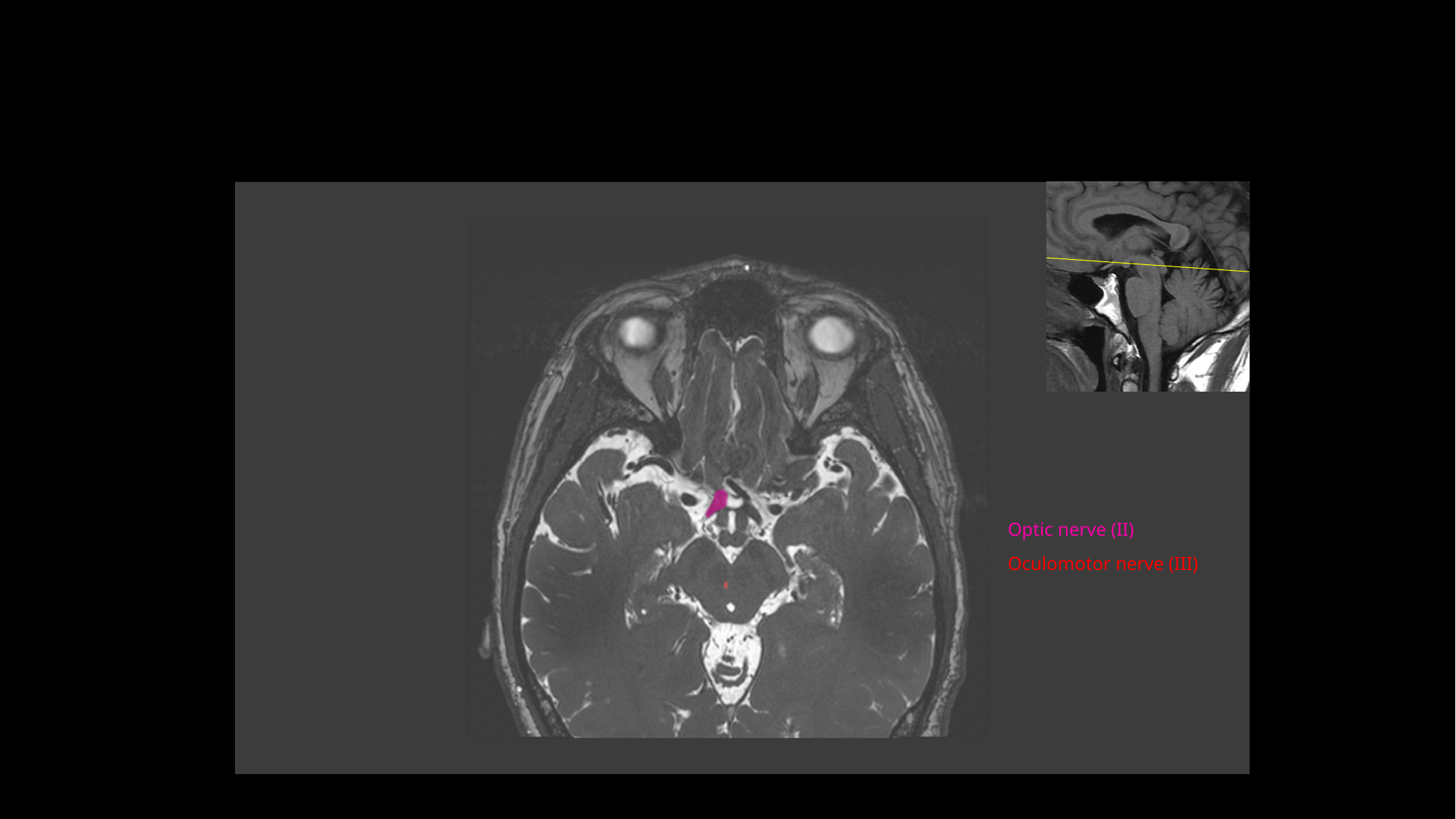

Optic nerve (II)
Oculomotor nerve (III)

## Slide 156
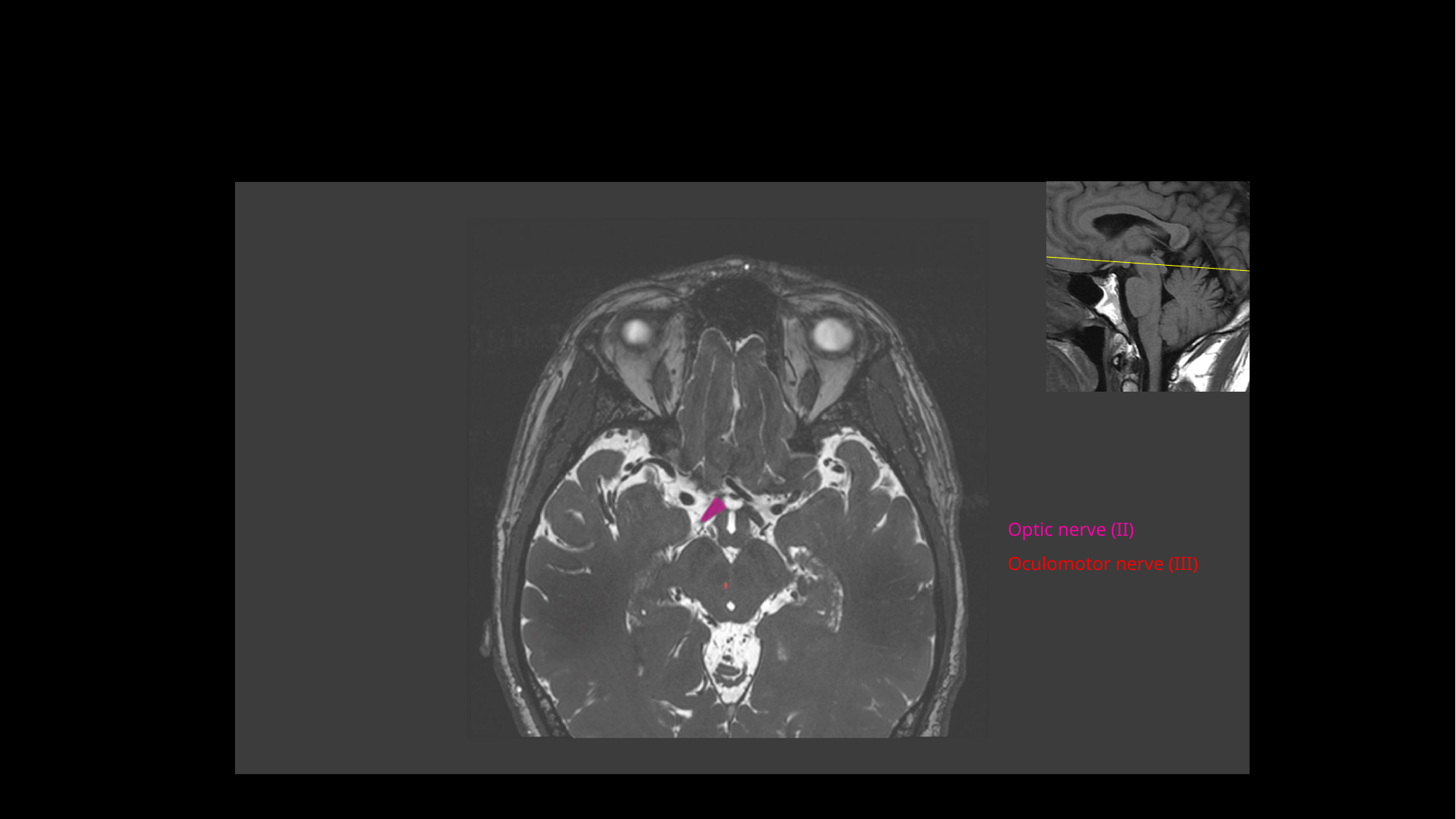

Optic nerve (II)
Oculomotor nerve (III)

## Slide 157
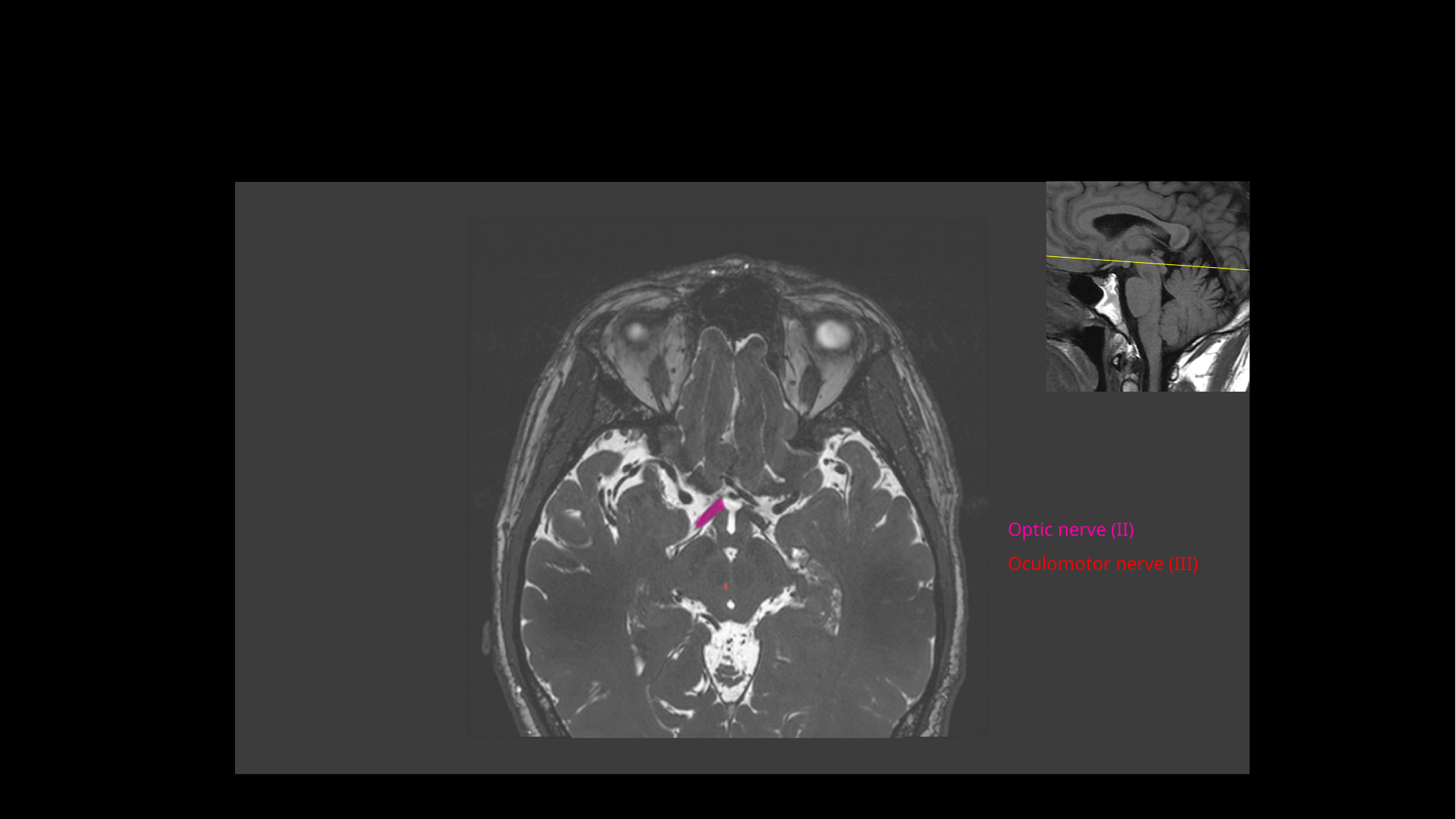

Optic nerve (II)
Oculomotor nerve (III)

## Slide 158
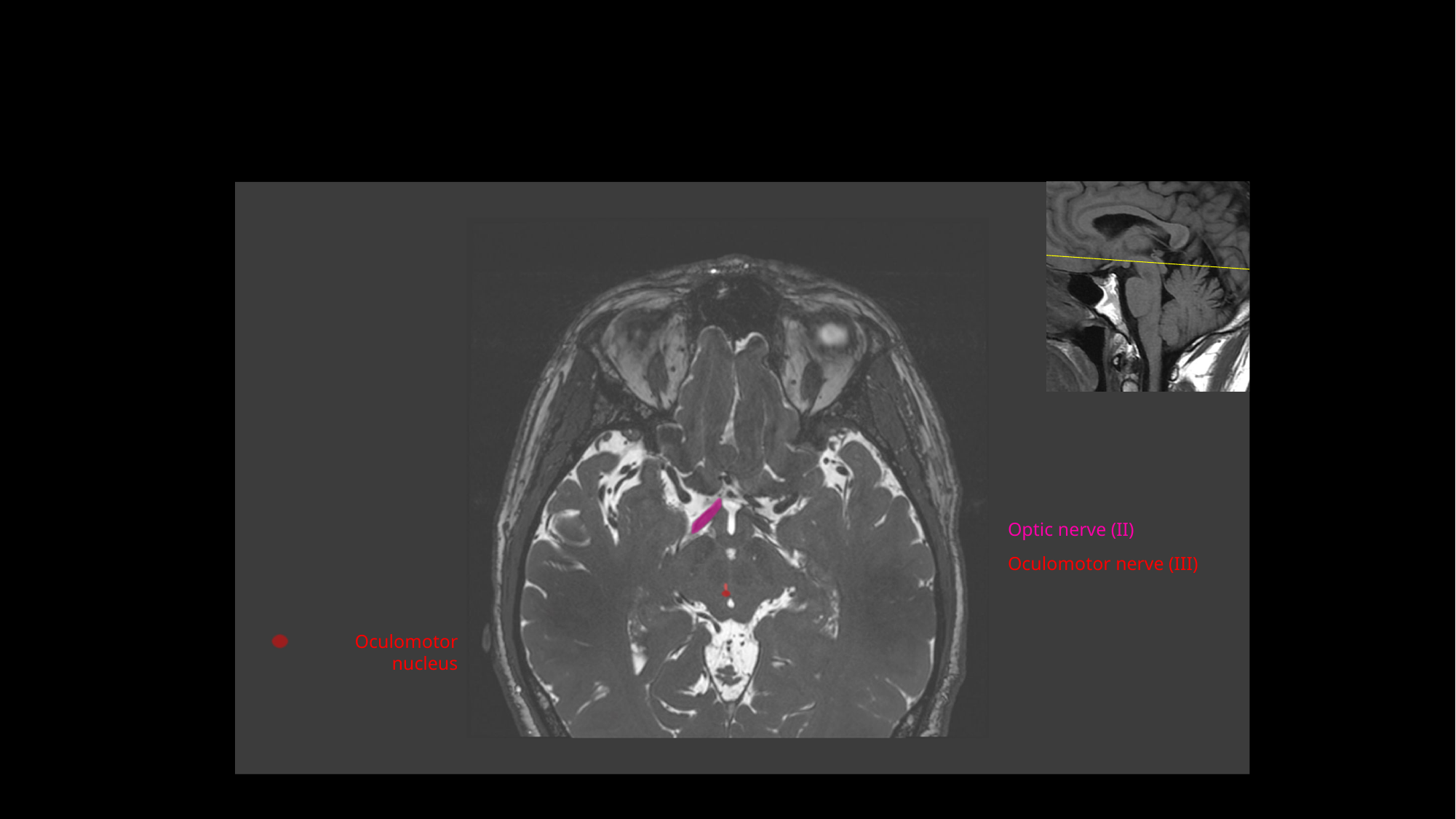

Optic nerve (II)
Oculomotor nerve (III)
Oculomotor nucleus

## Slide 159
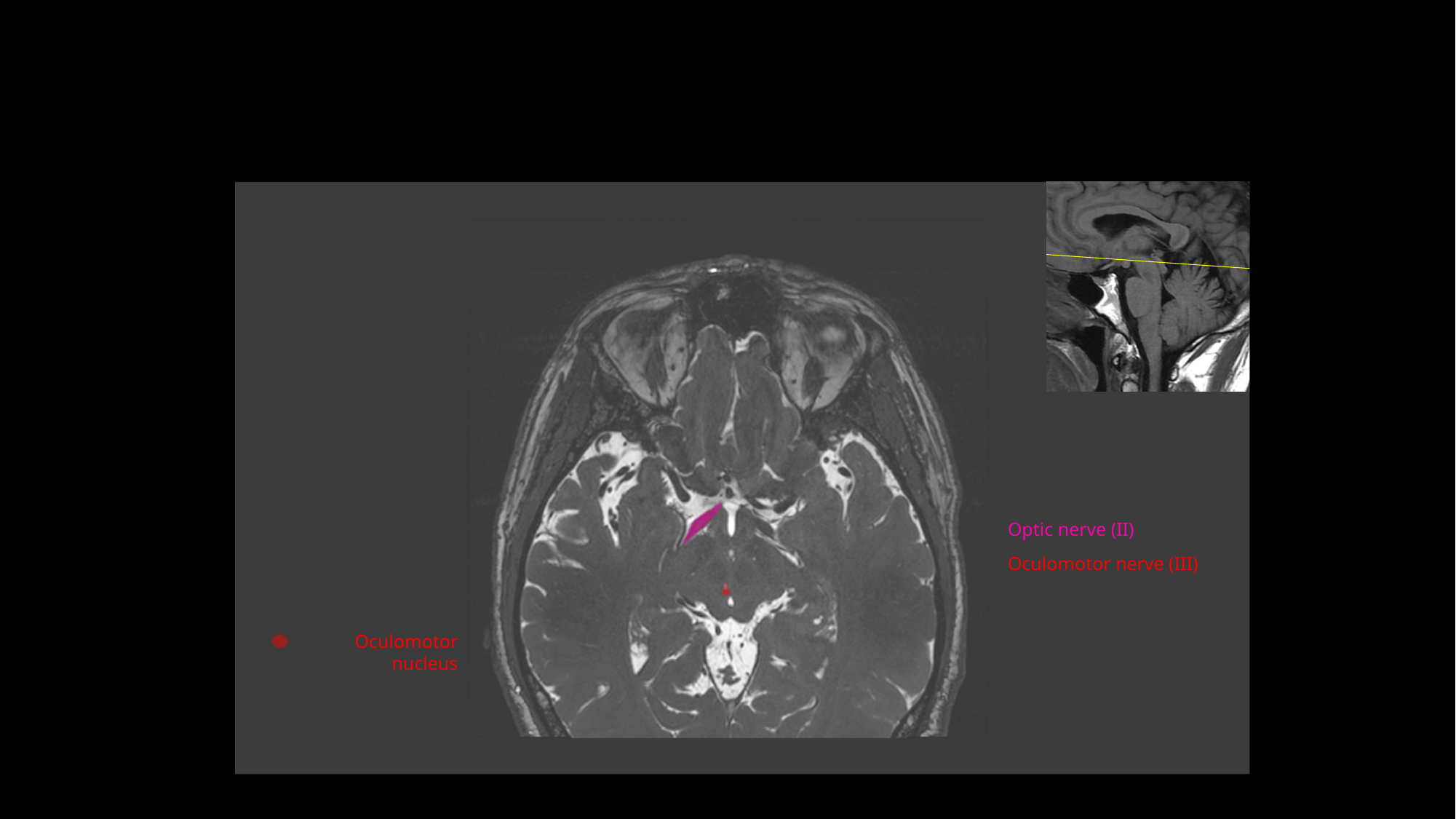

Optic nerve (II)
Oculomotor nerve (III)
Oculomotor nucleus

## Slide 160
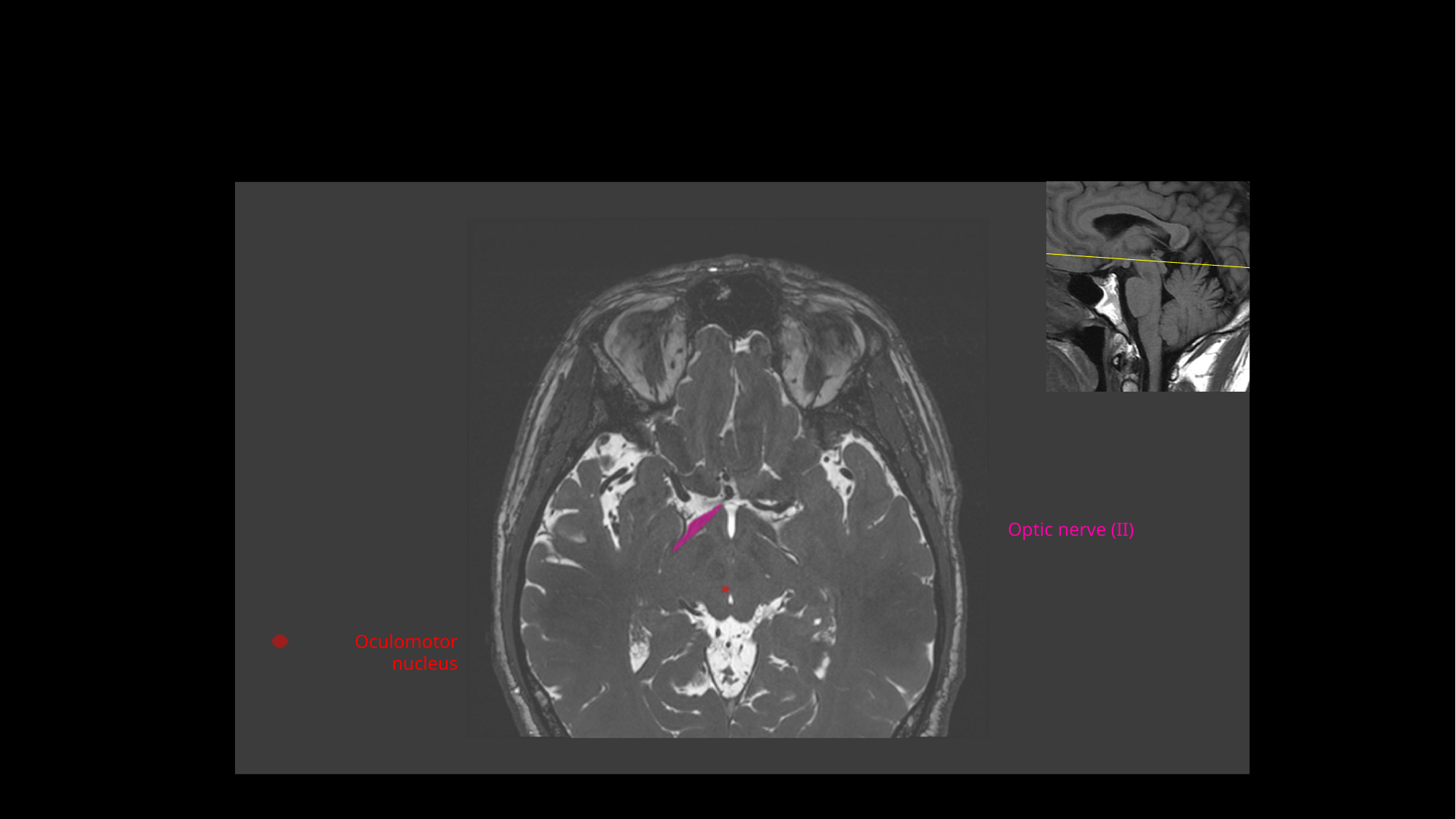

Optic nerve (II)
Oculomotor nucleus

## Slide 161
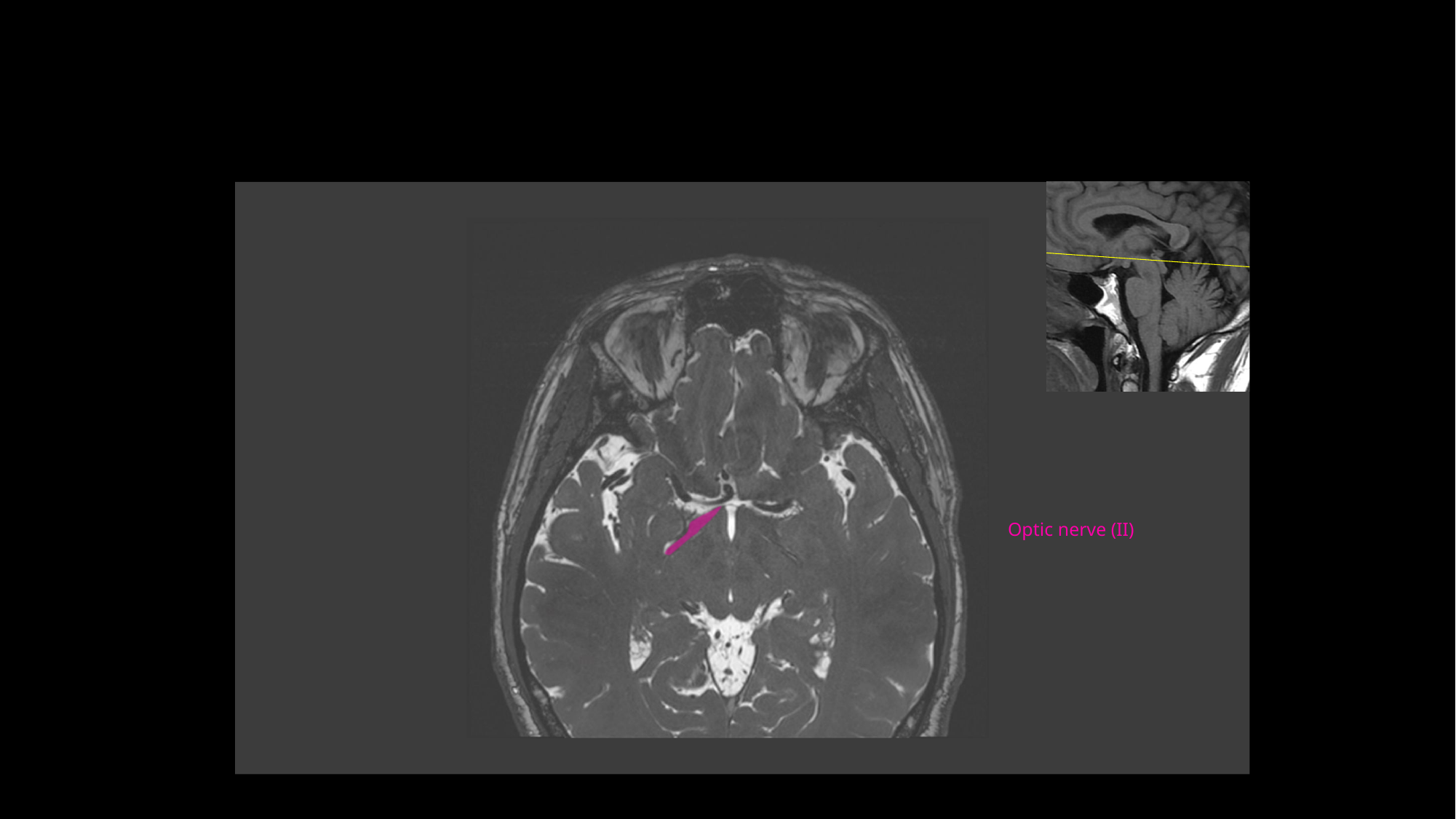

Optic nerve (II)

## Slide 162
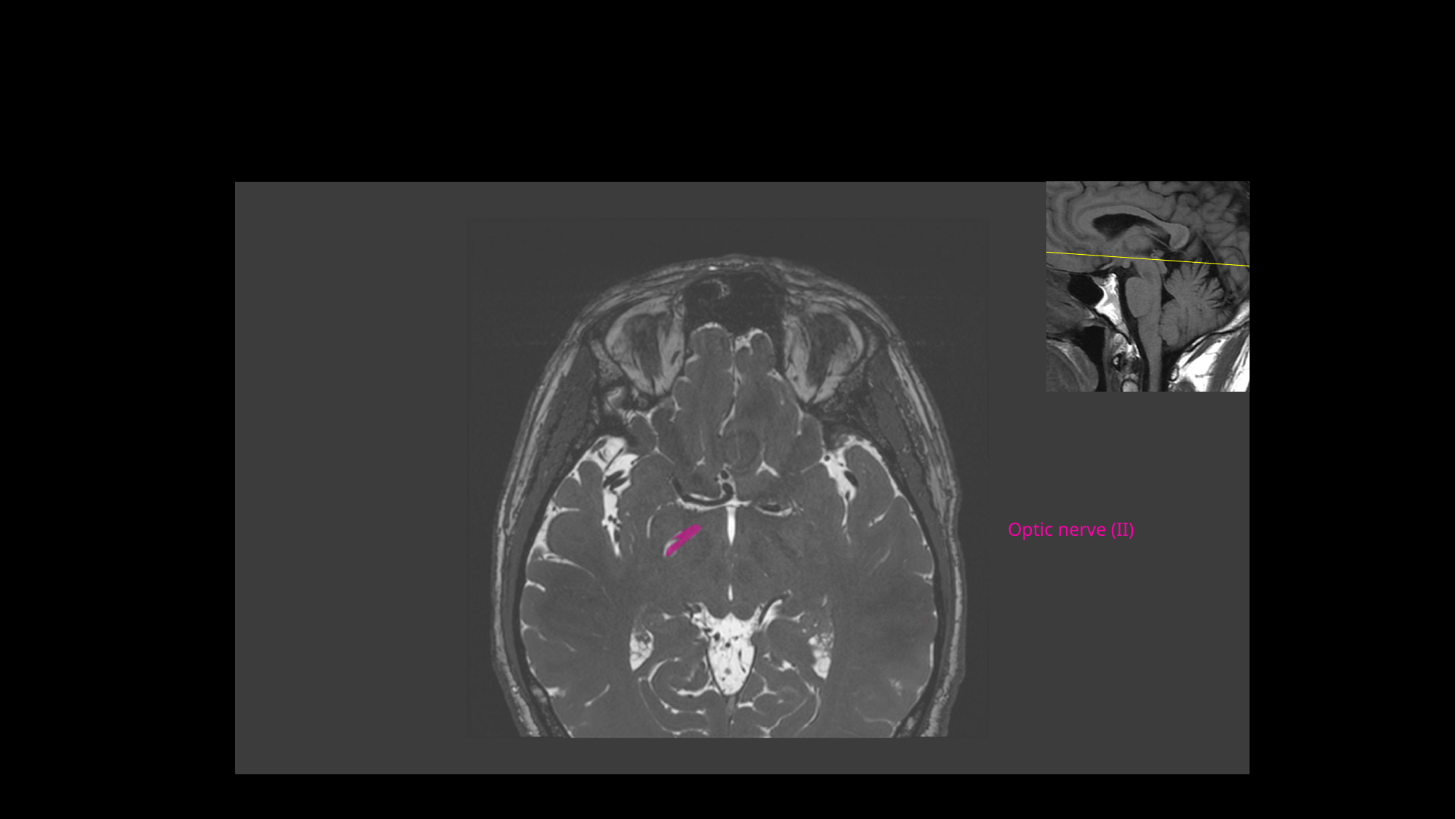

Optic nerve (II)

## Slide 163
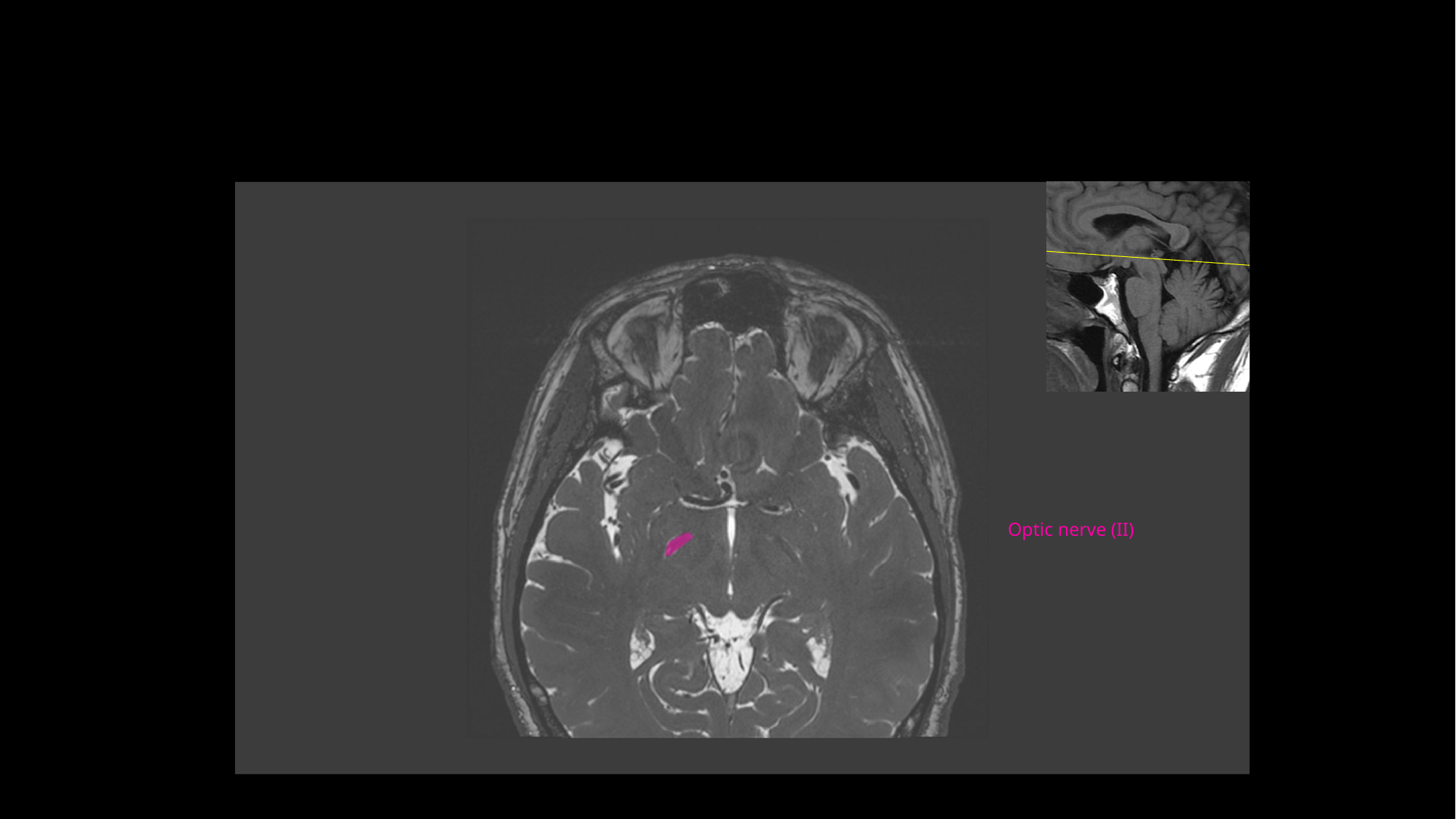

Optic nerve (II)

## Slide 164
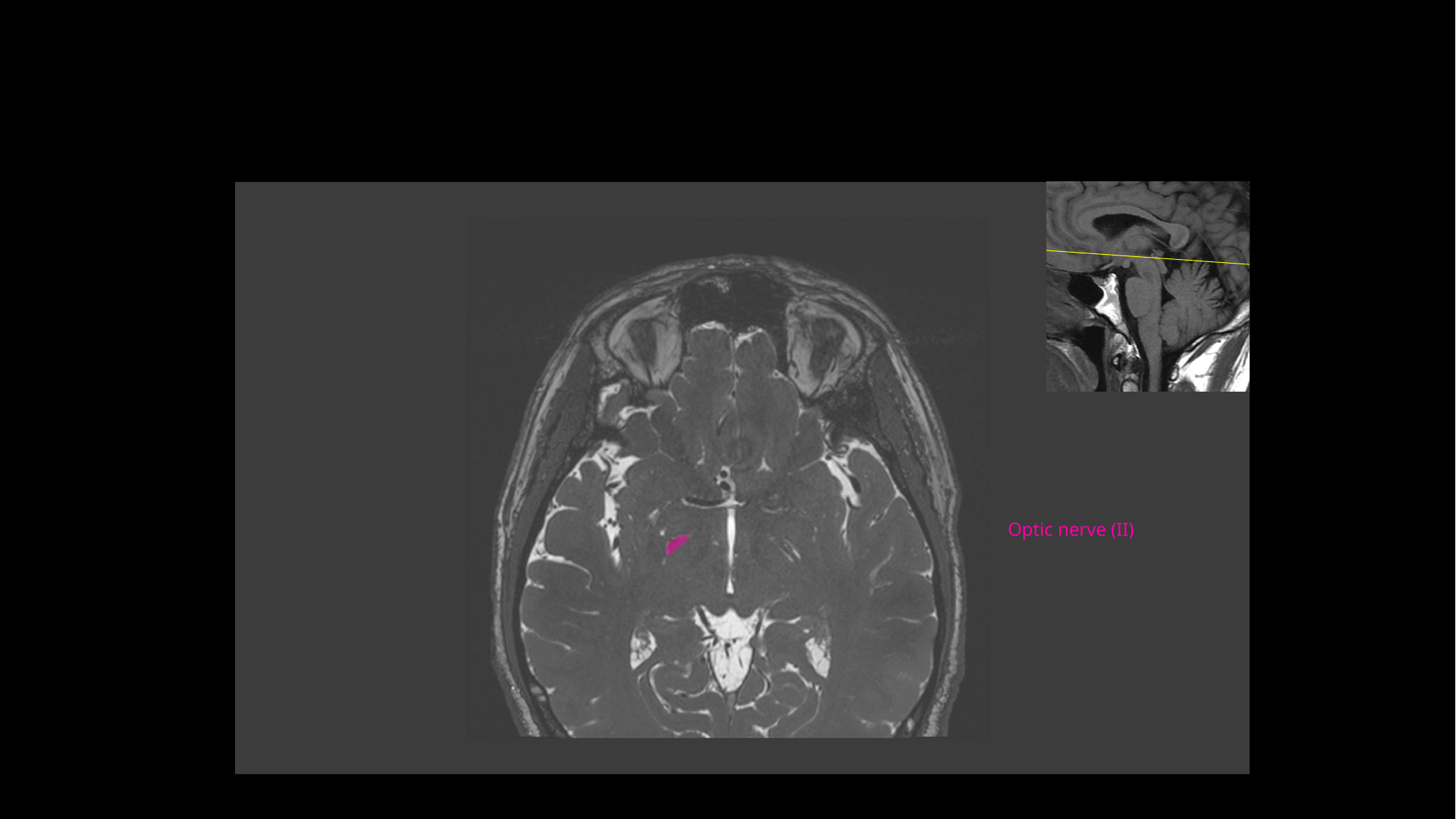

Optic nerve (II)

## Slide 165
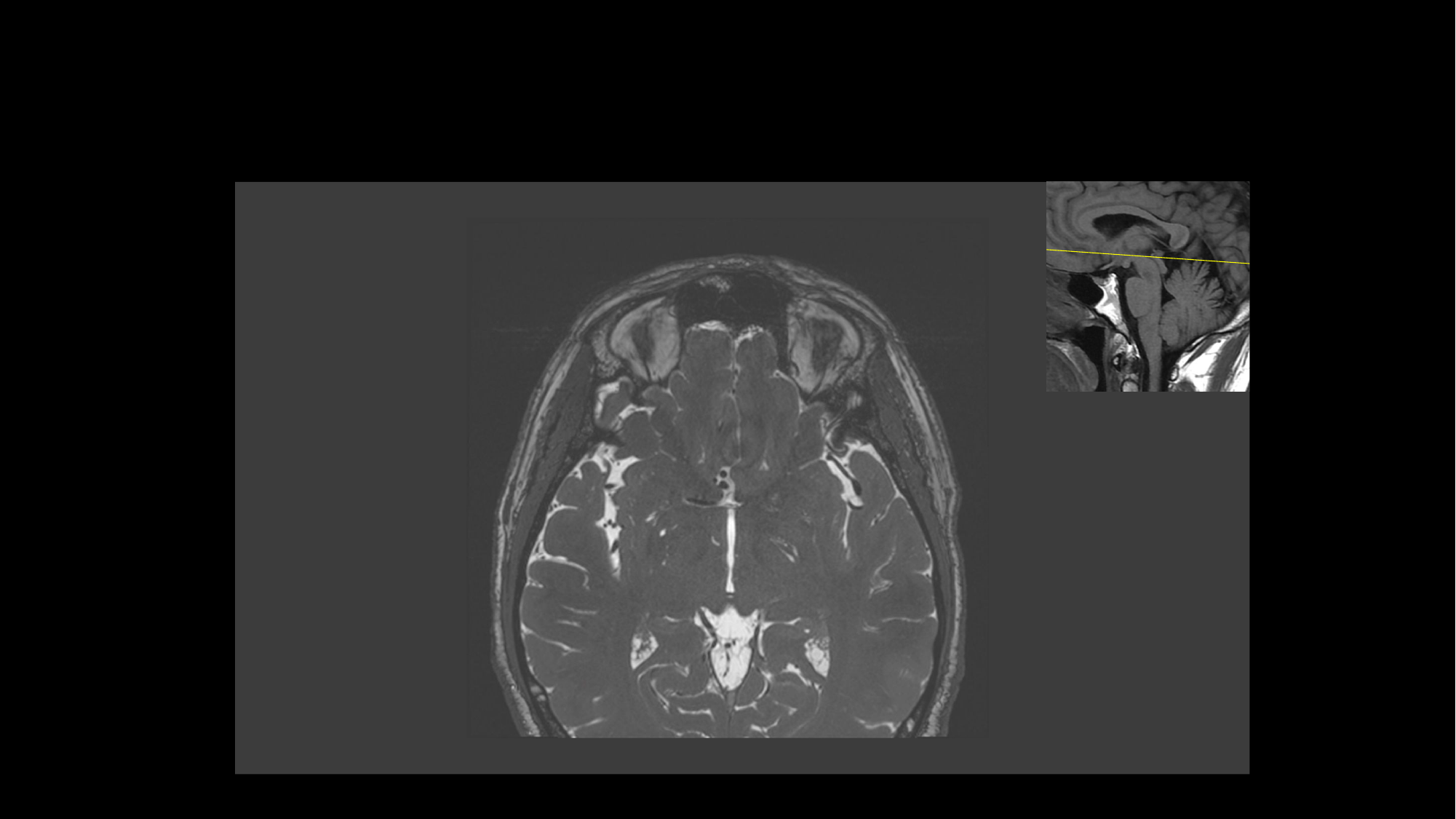

## Slide 166
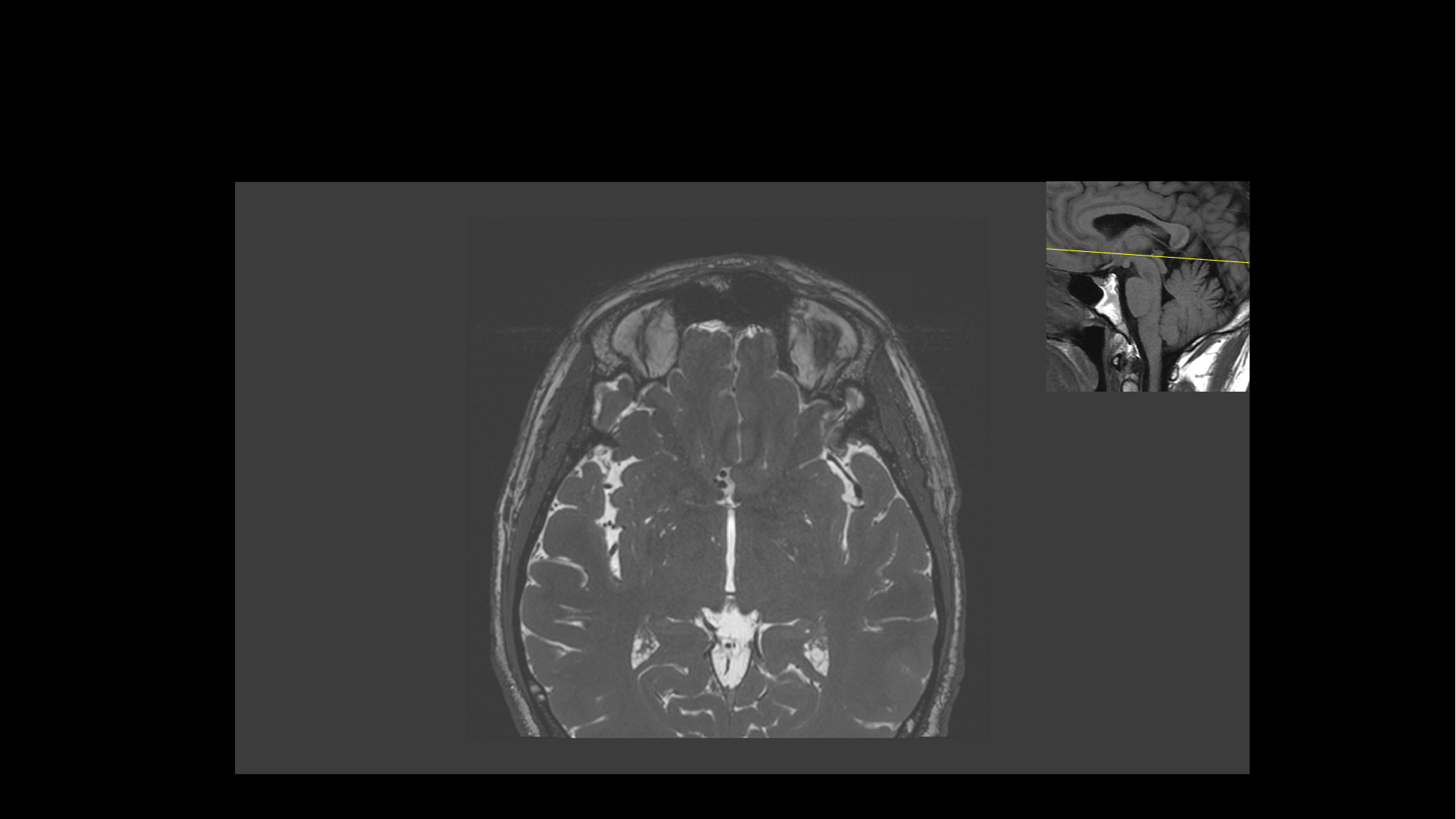

## Slide 167
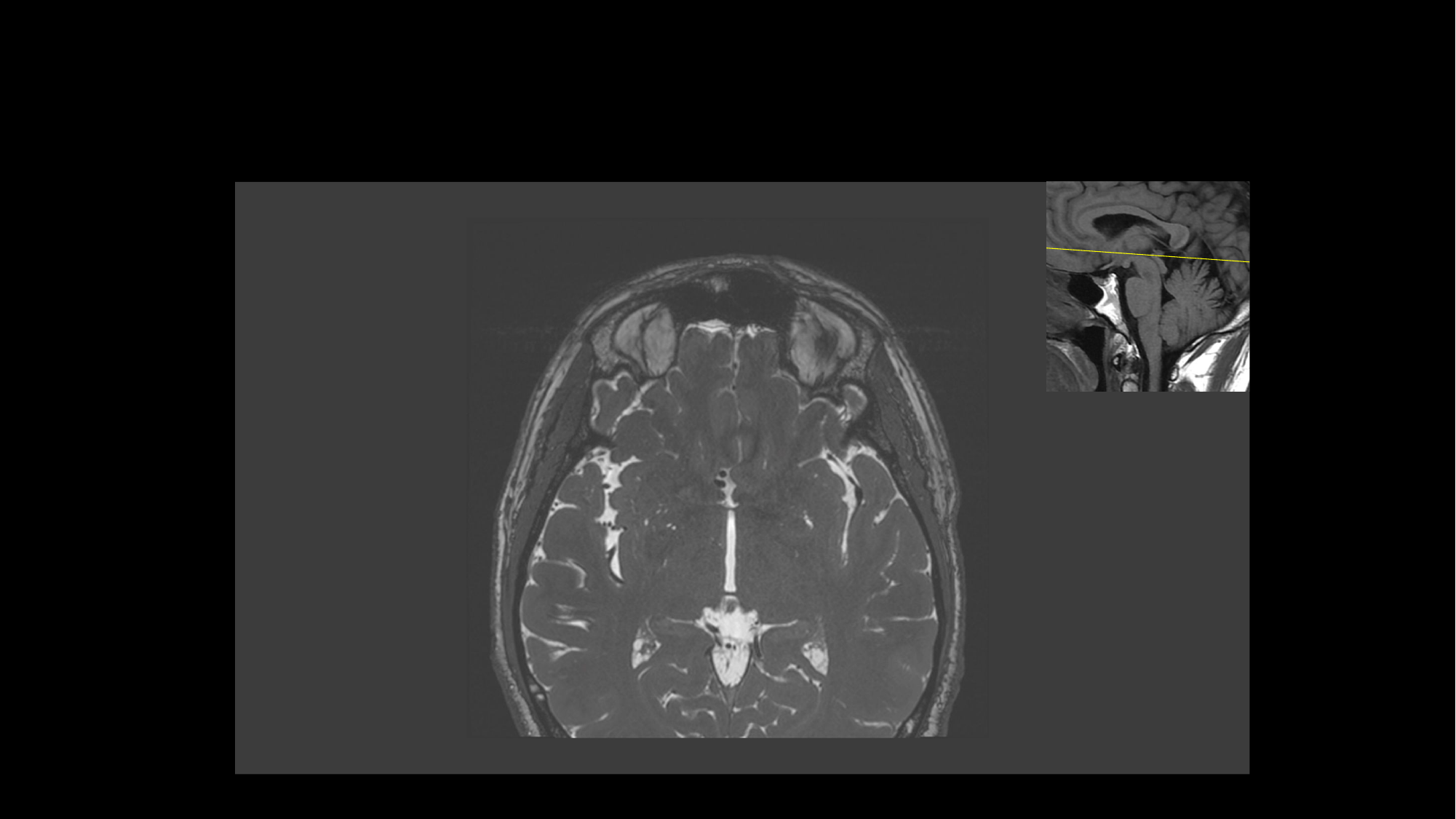

## Slide 168
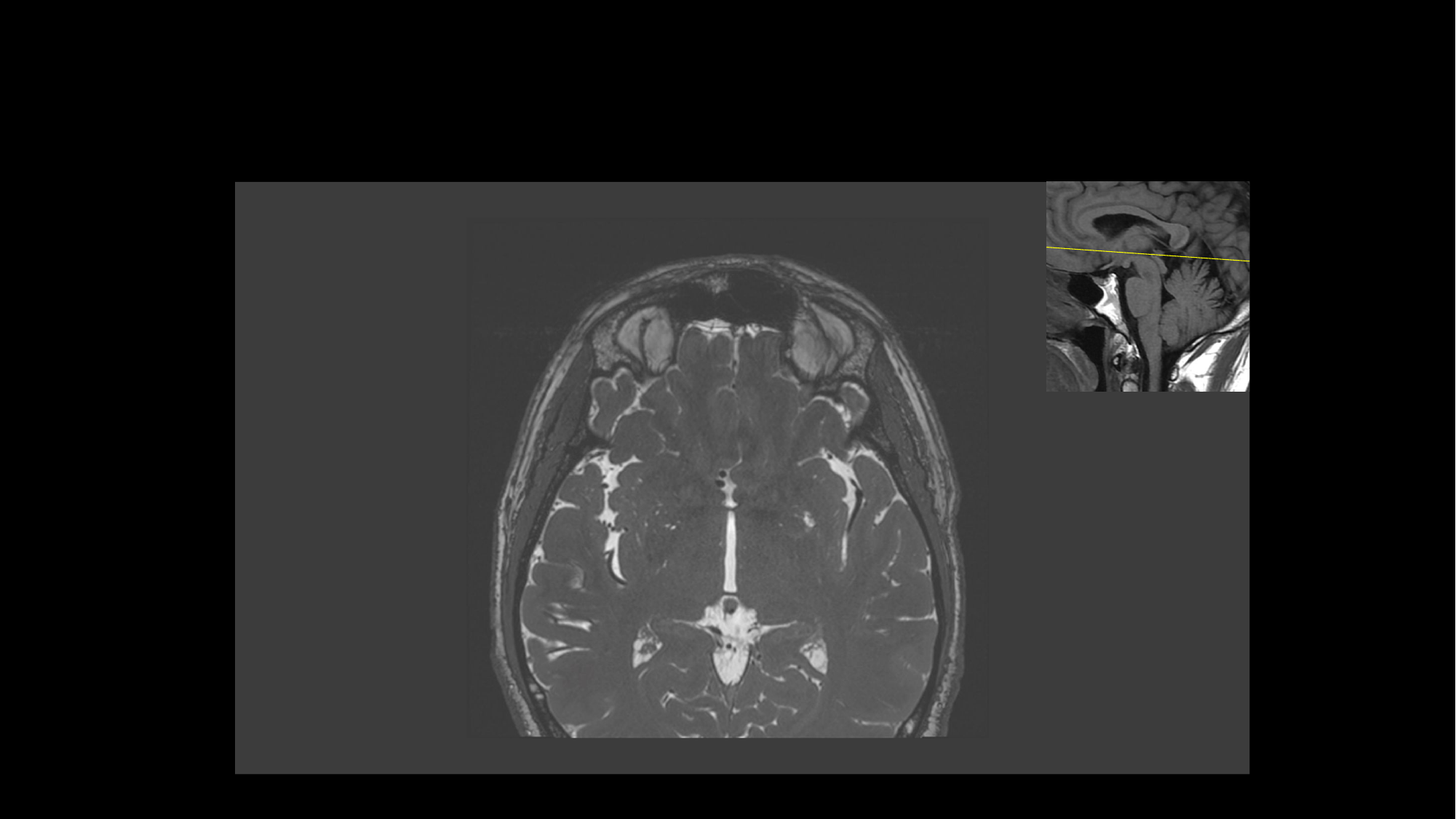

## Slide 169
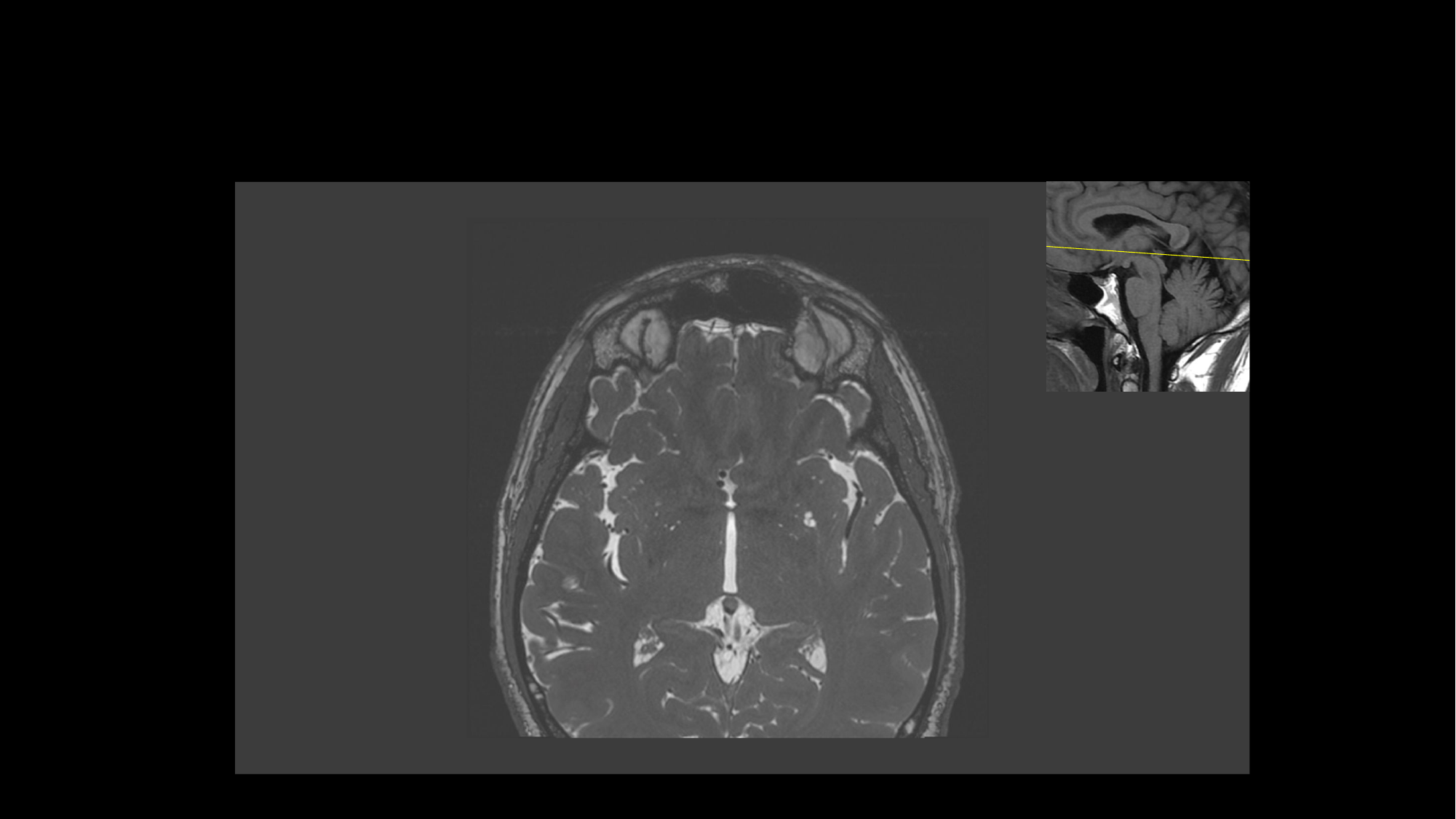

## Slide 170
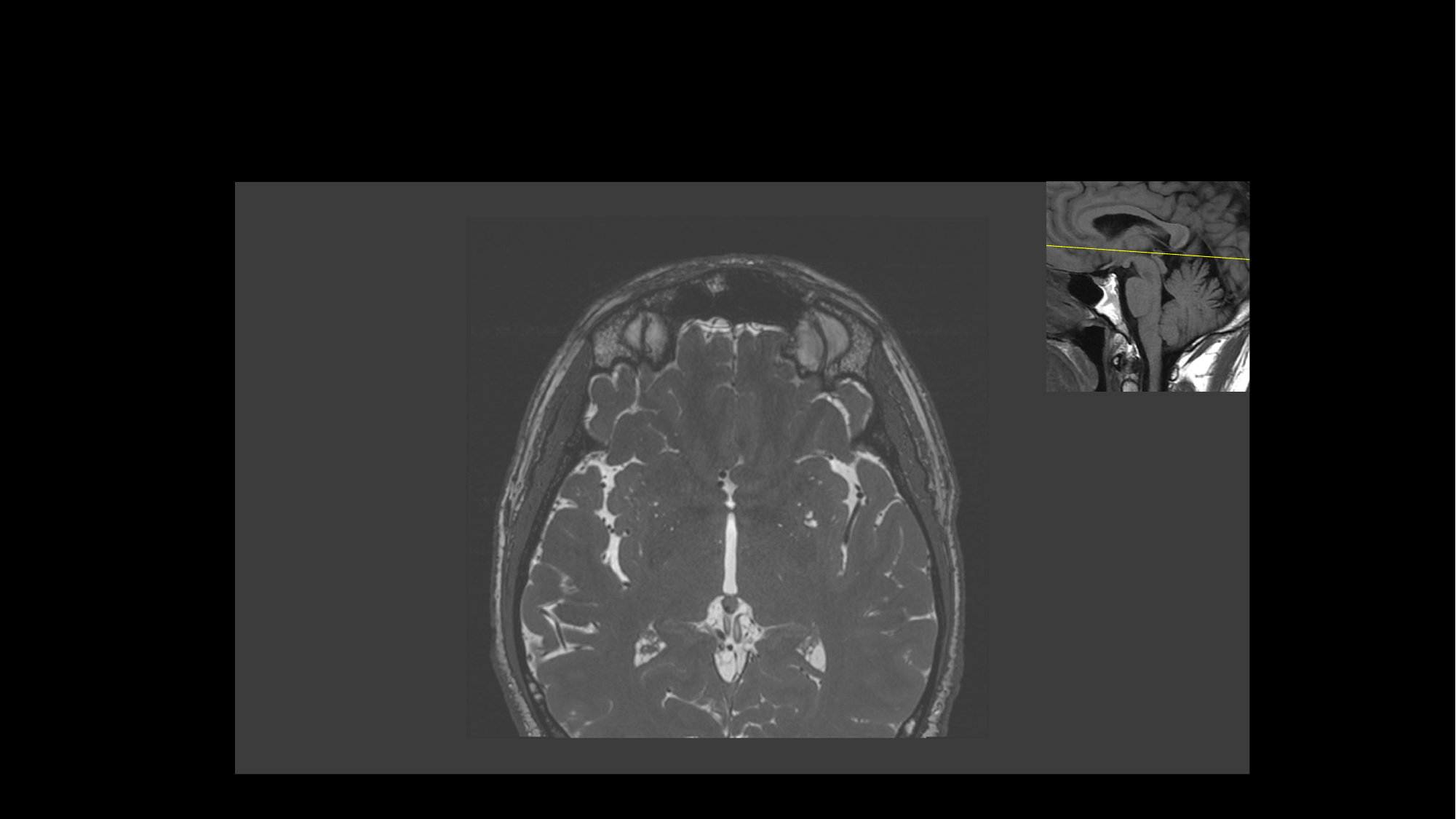

## Slide 171
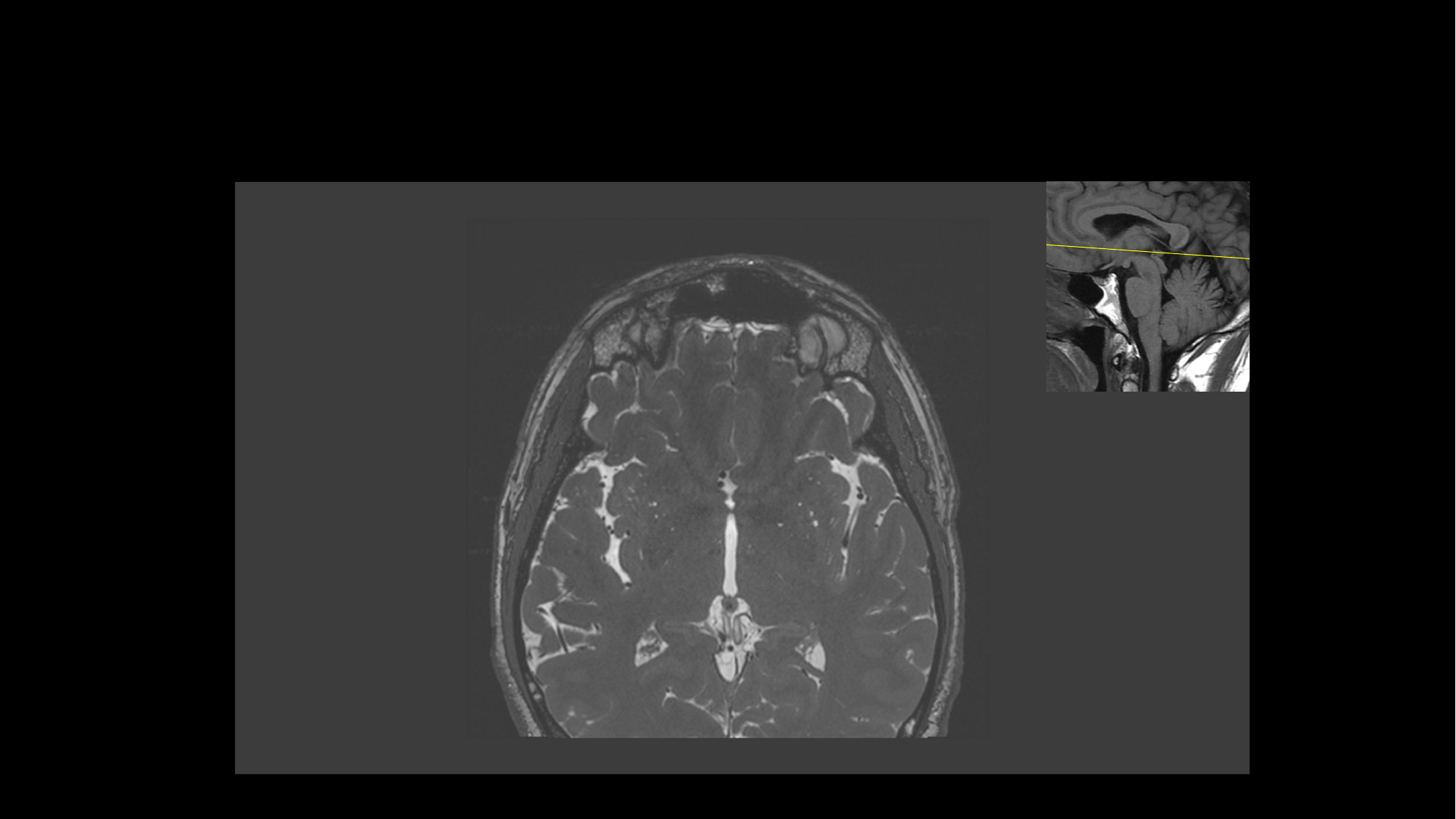

## Slide 172
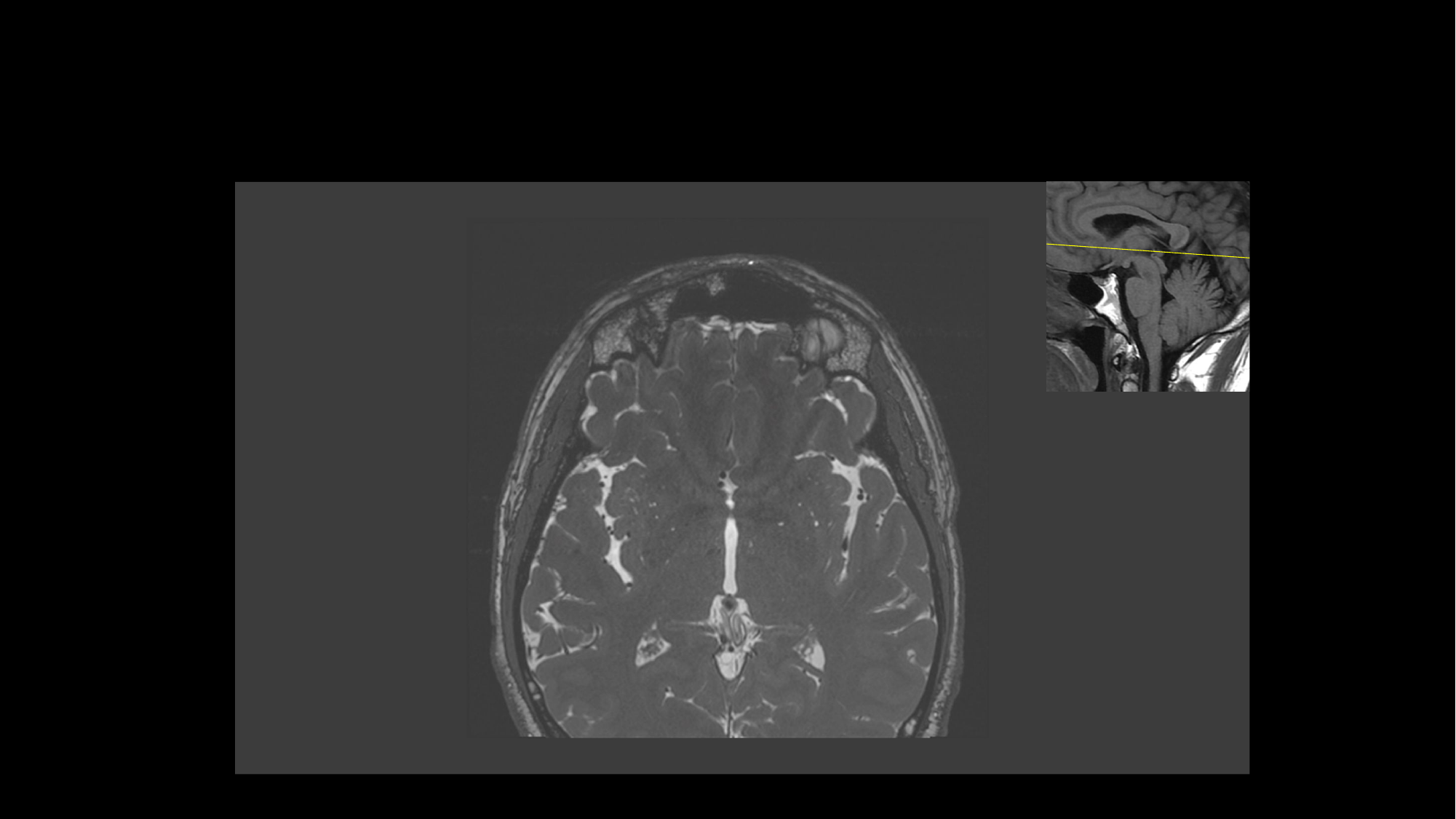

## Slide 173
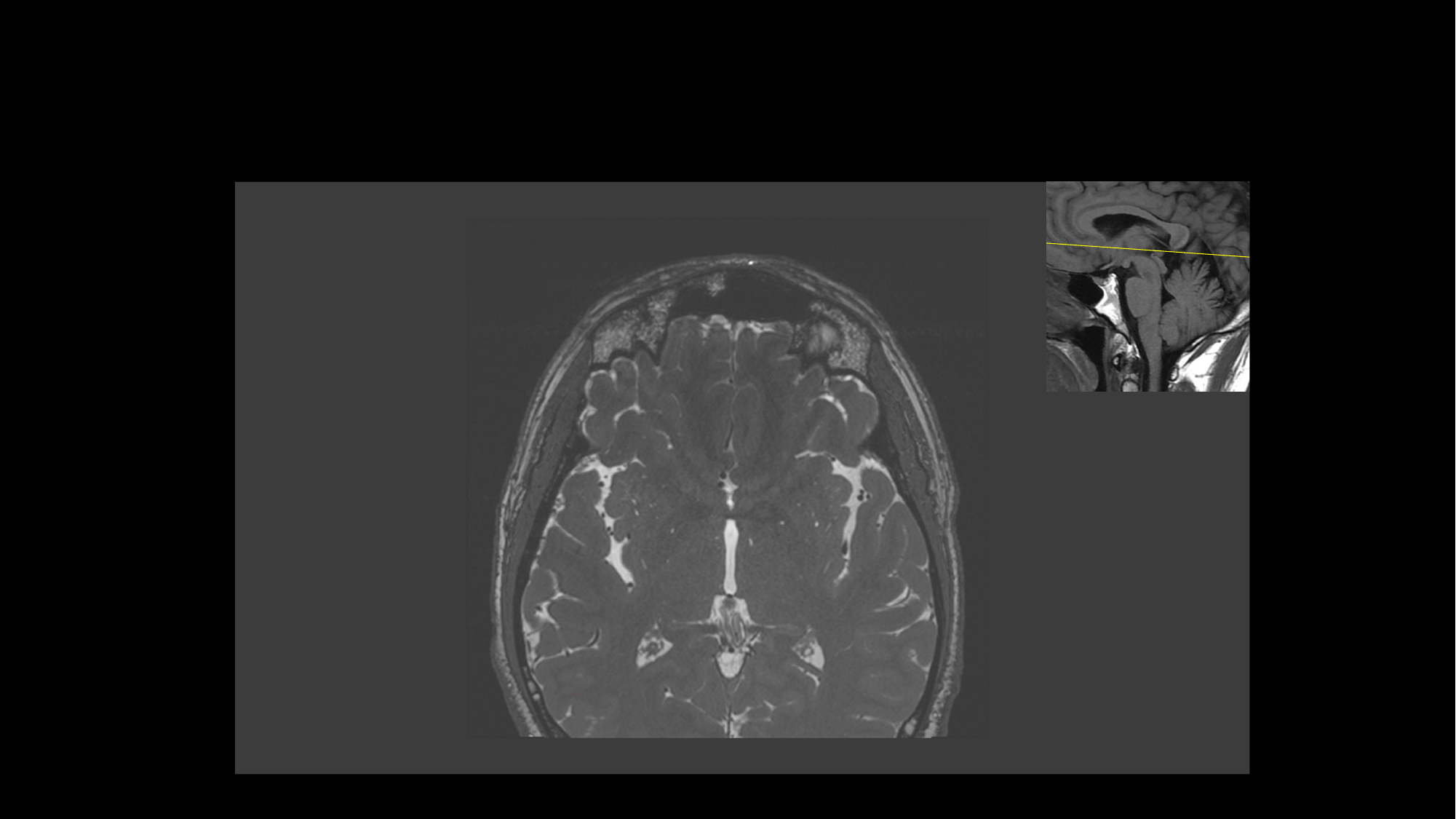

## Slide 174
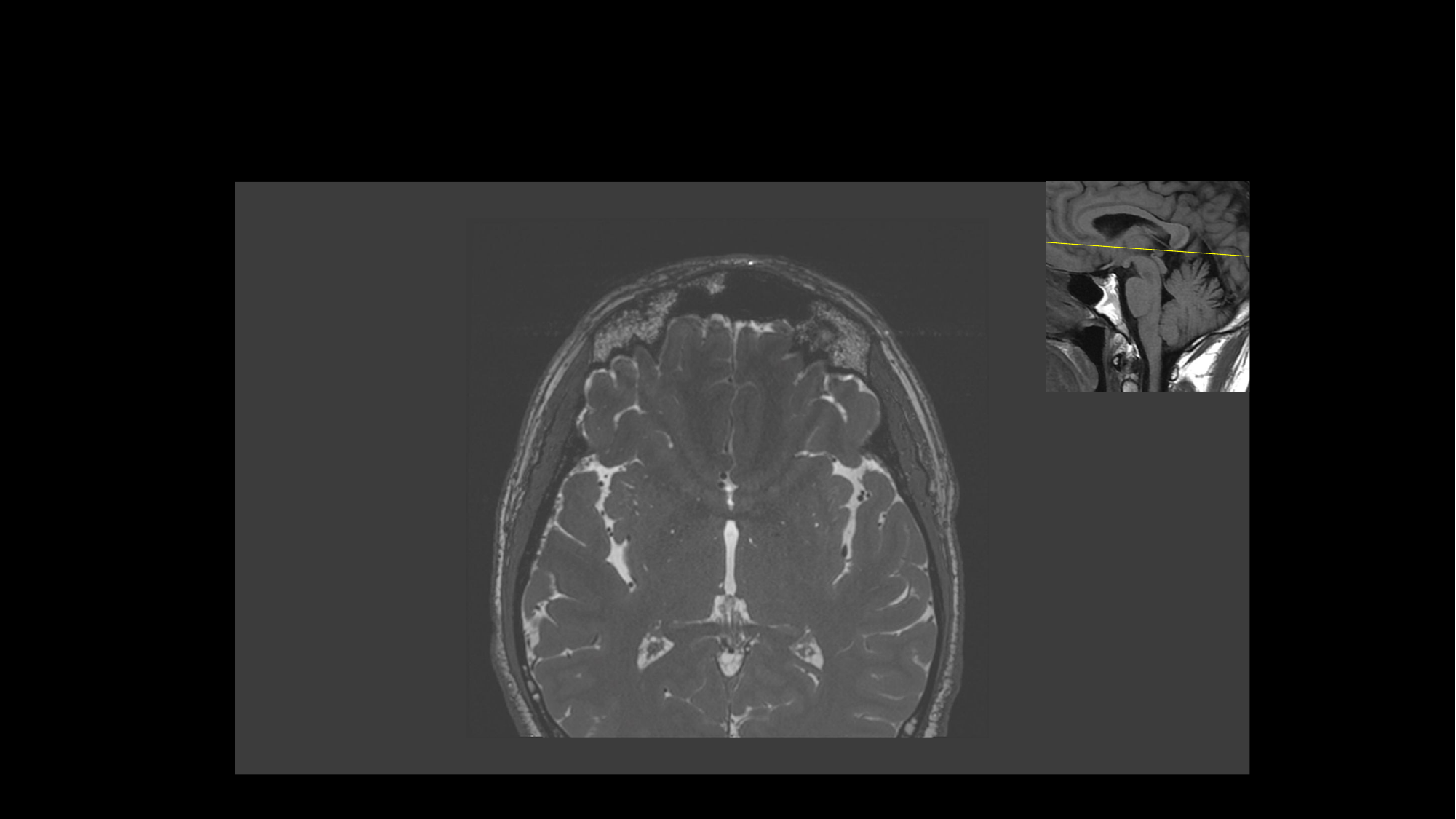

## Slide 175
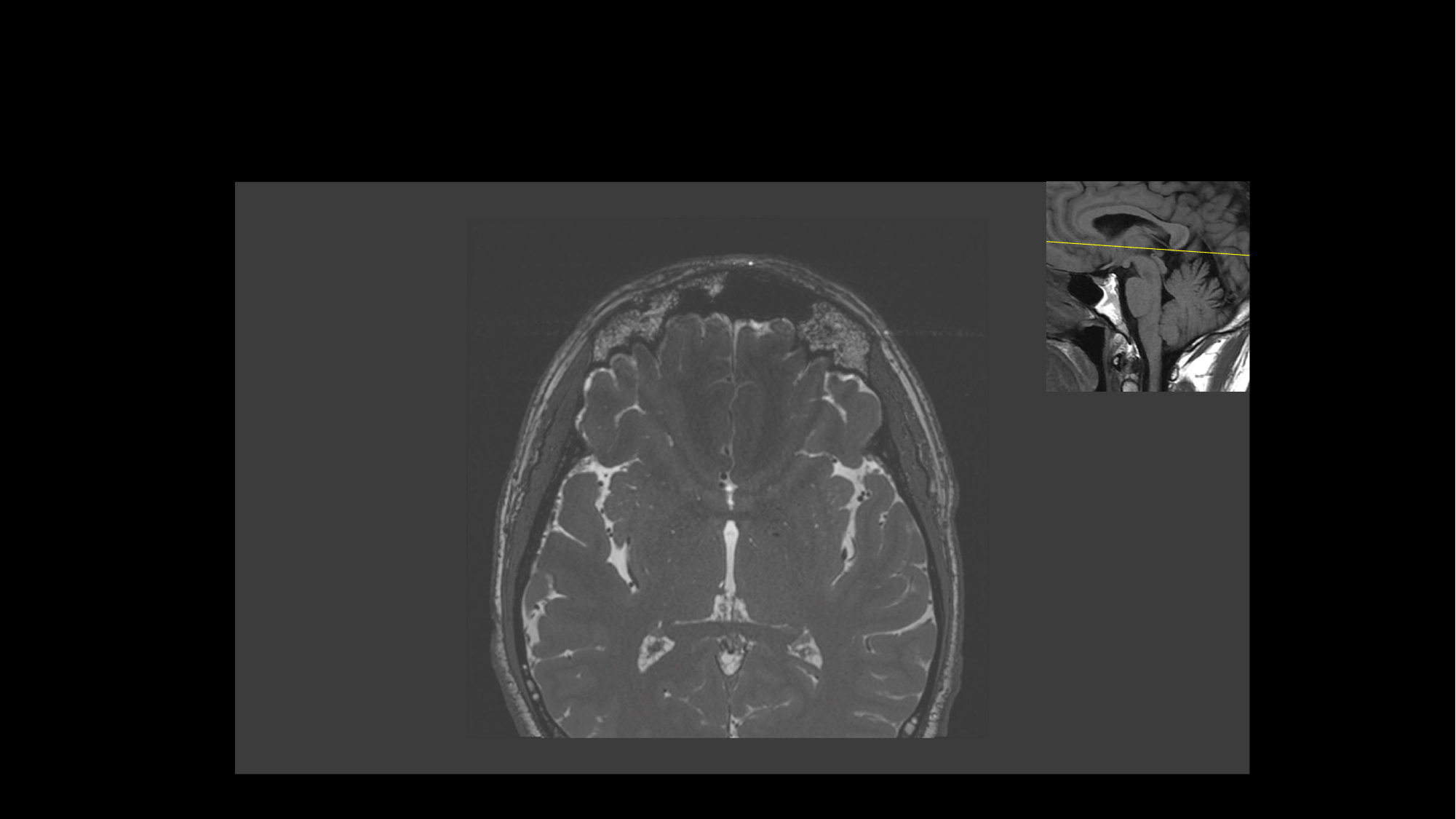

## Slide 176
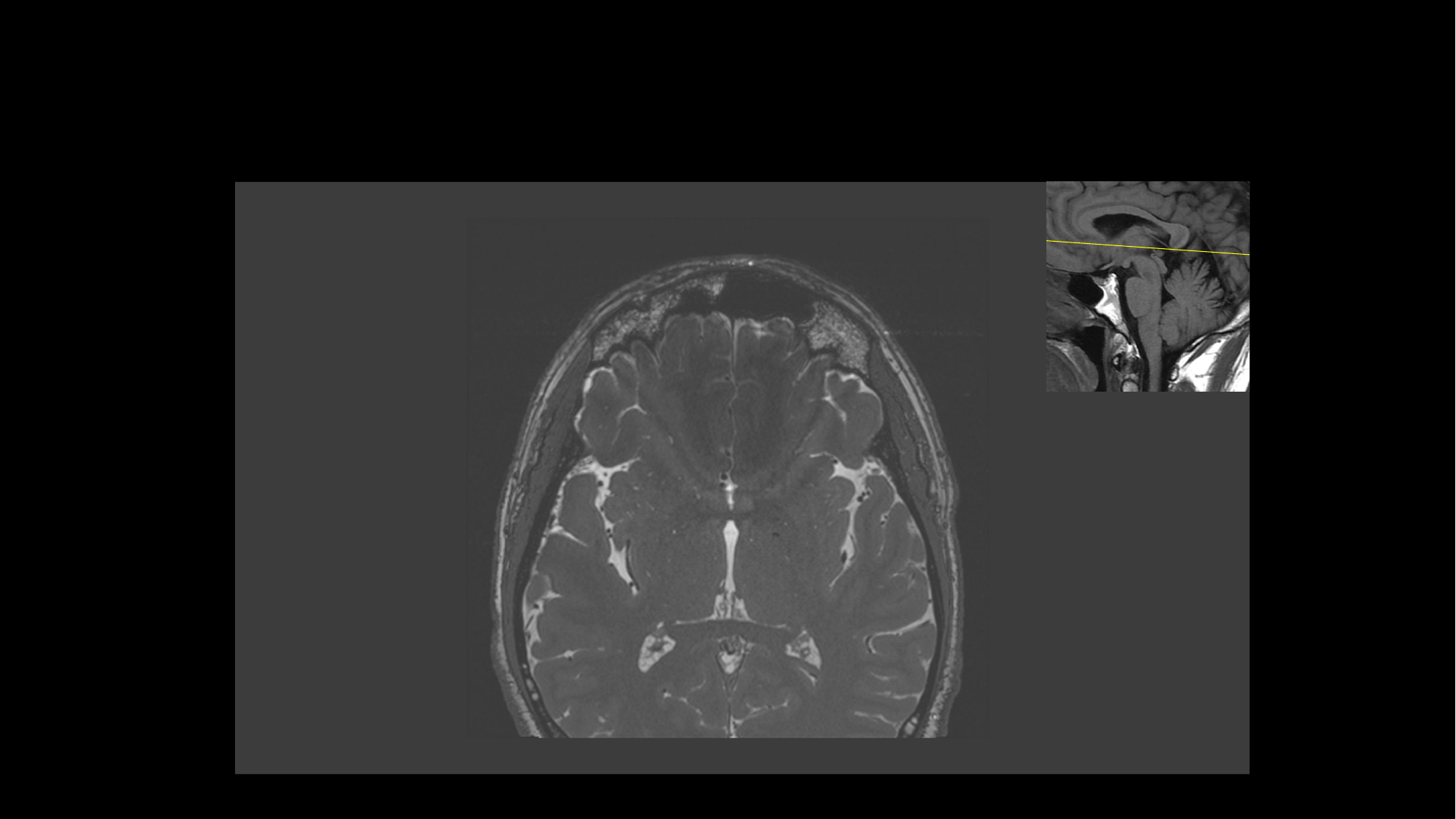

## Slide 177
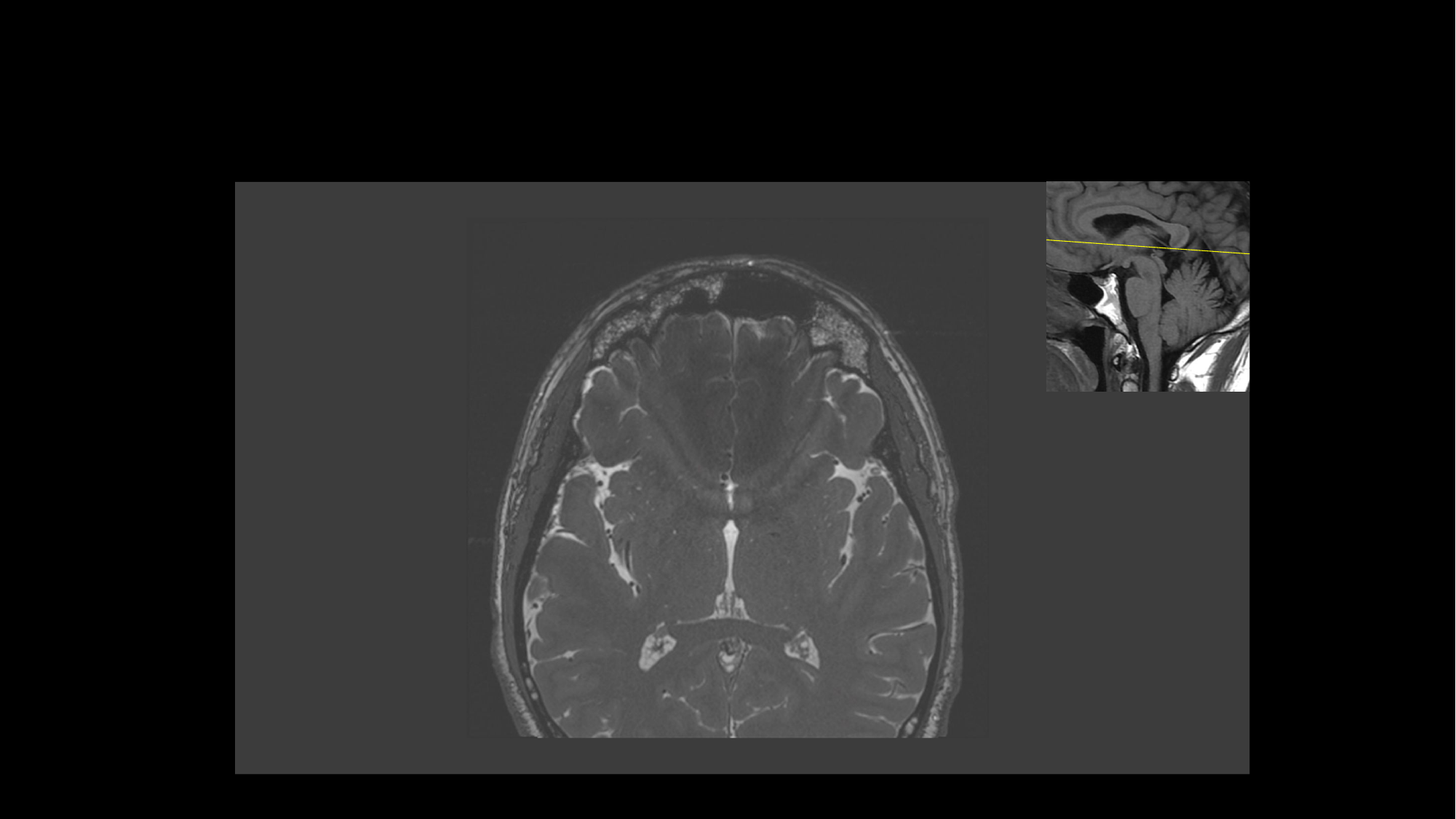

## Slide 178
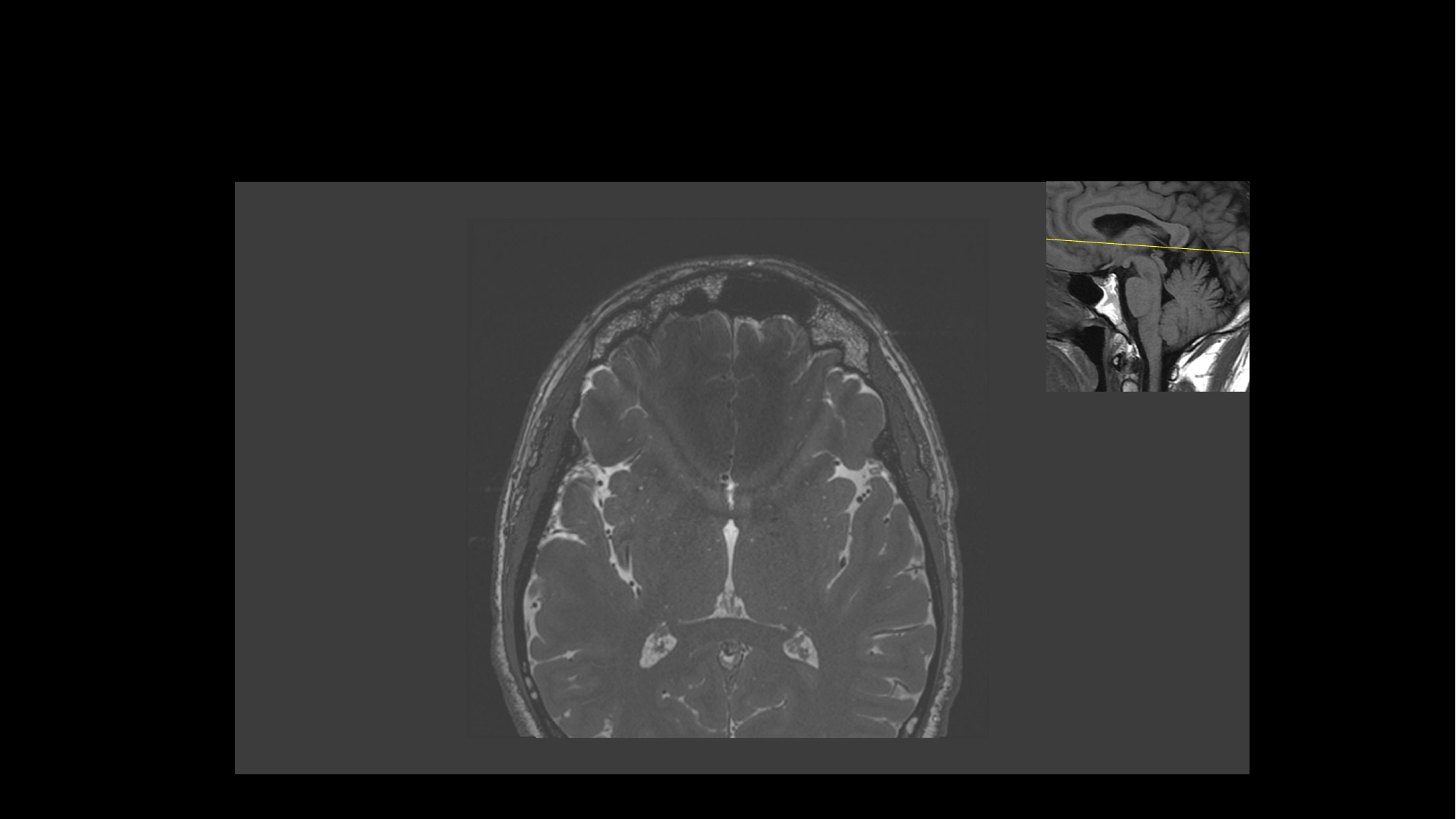

## Slide 179
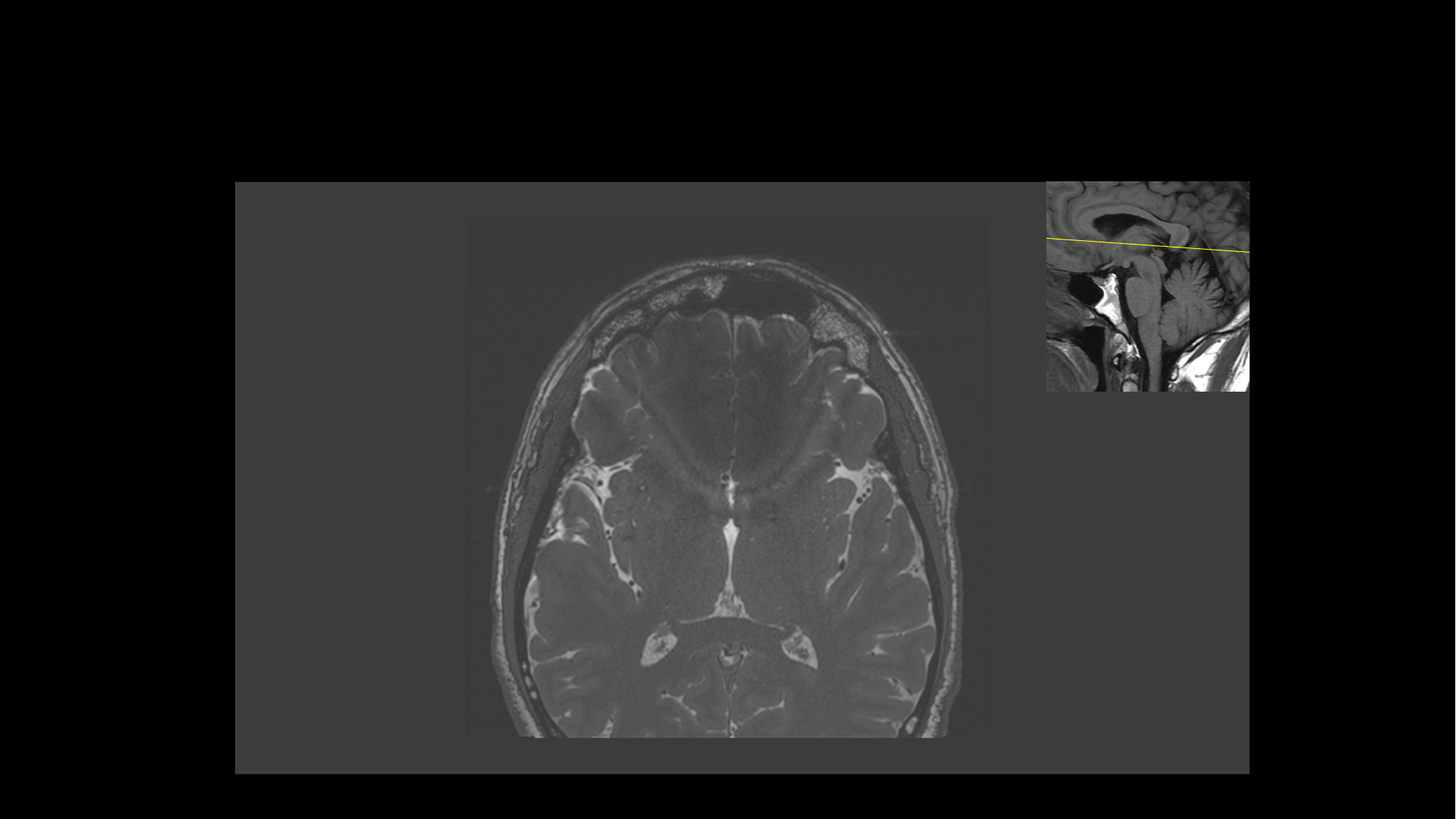

## Slide 180
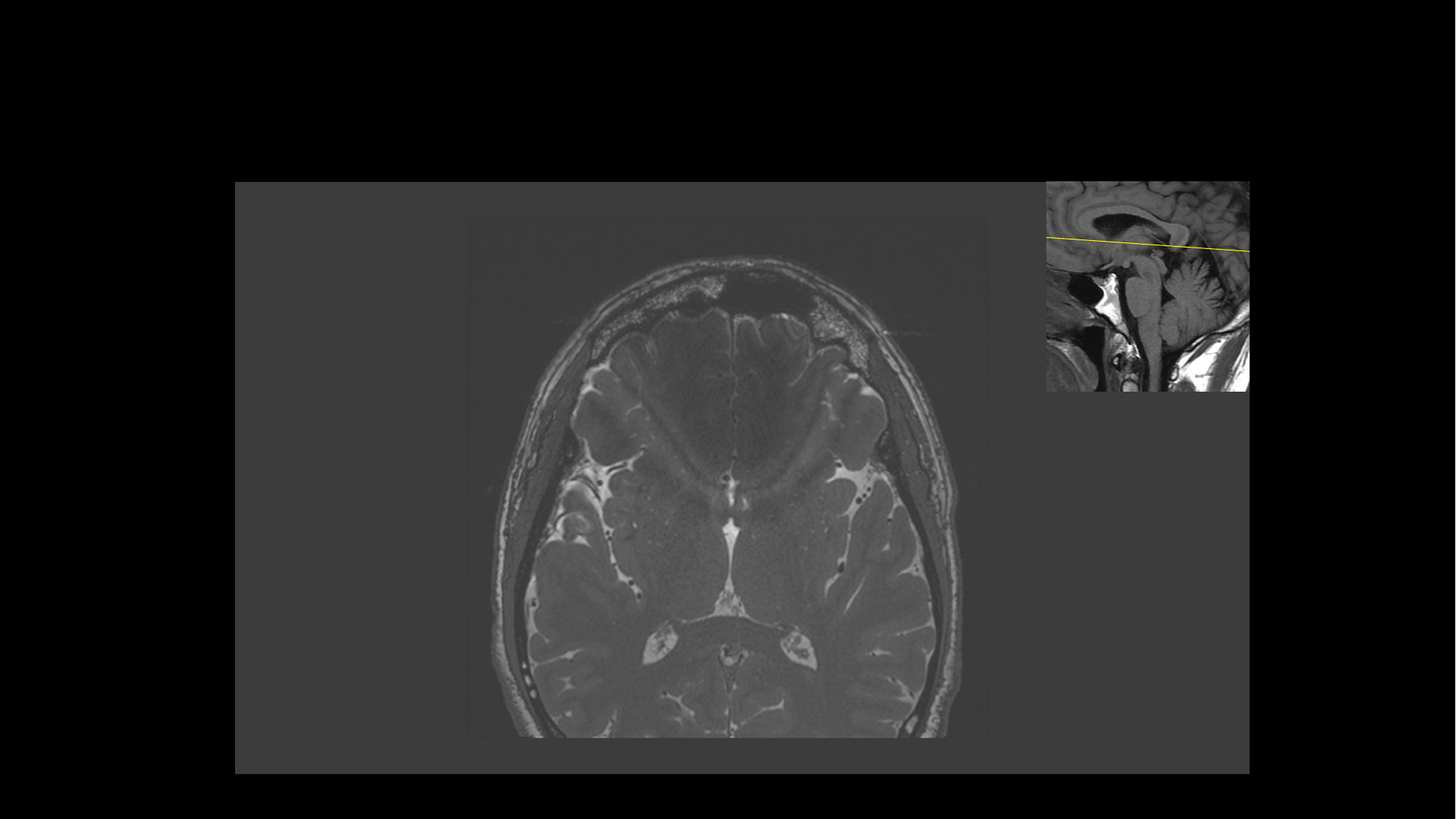

## Slide 181
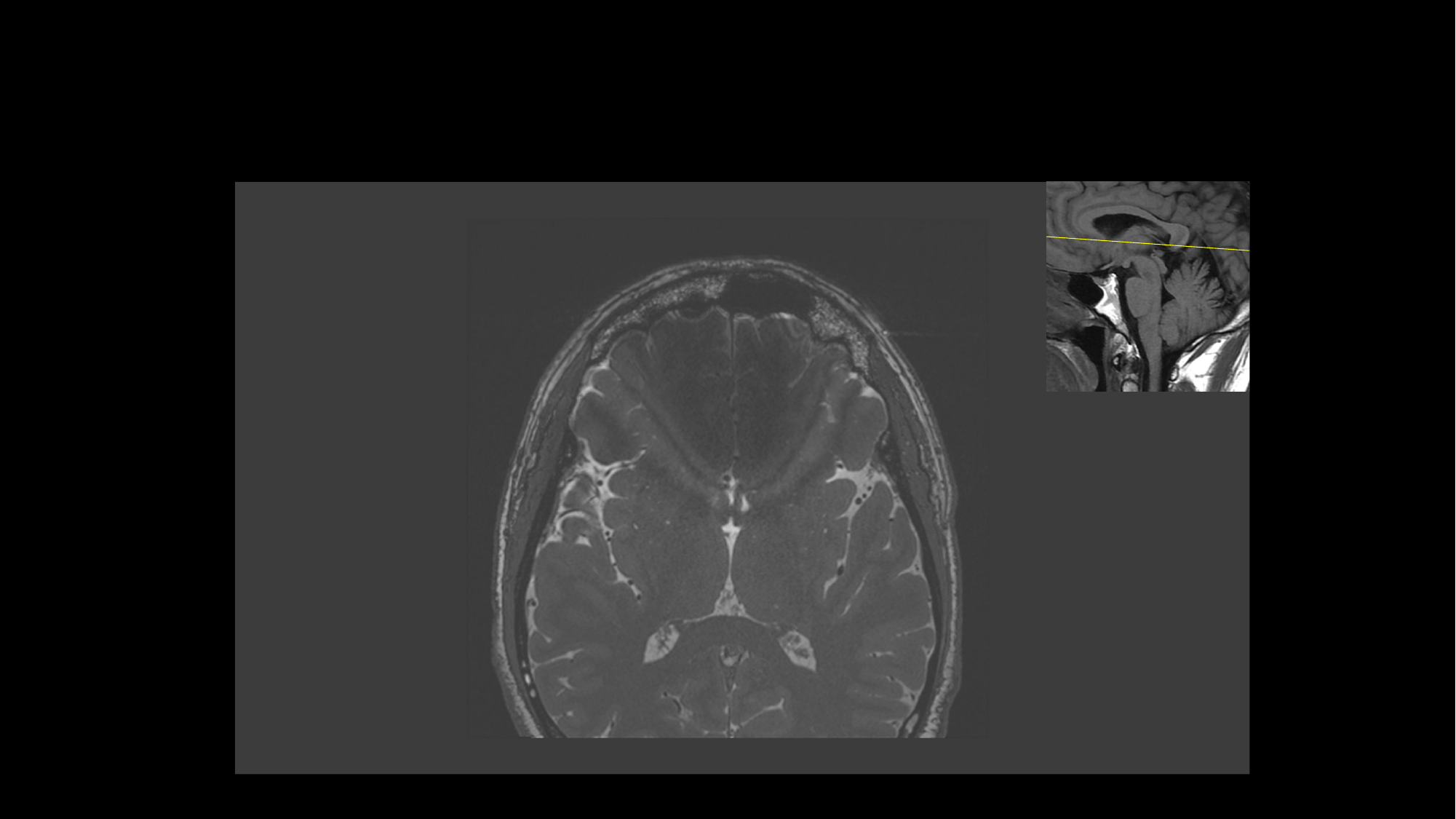

## Slide 182
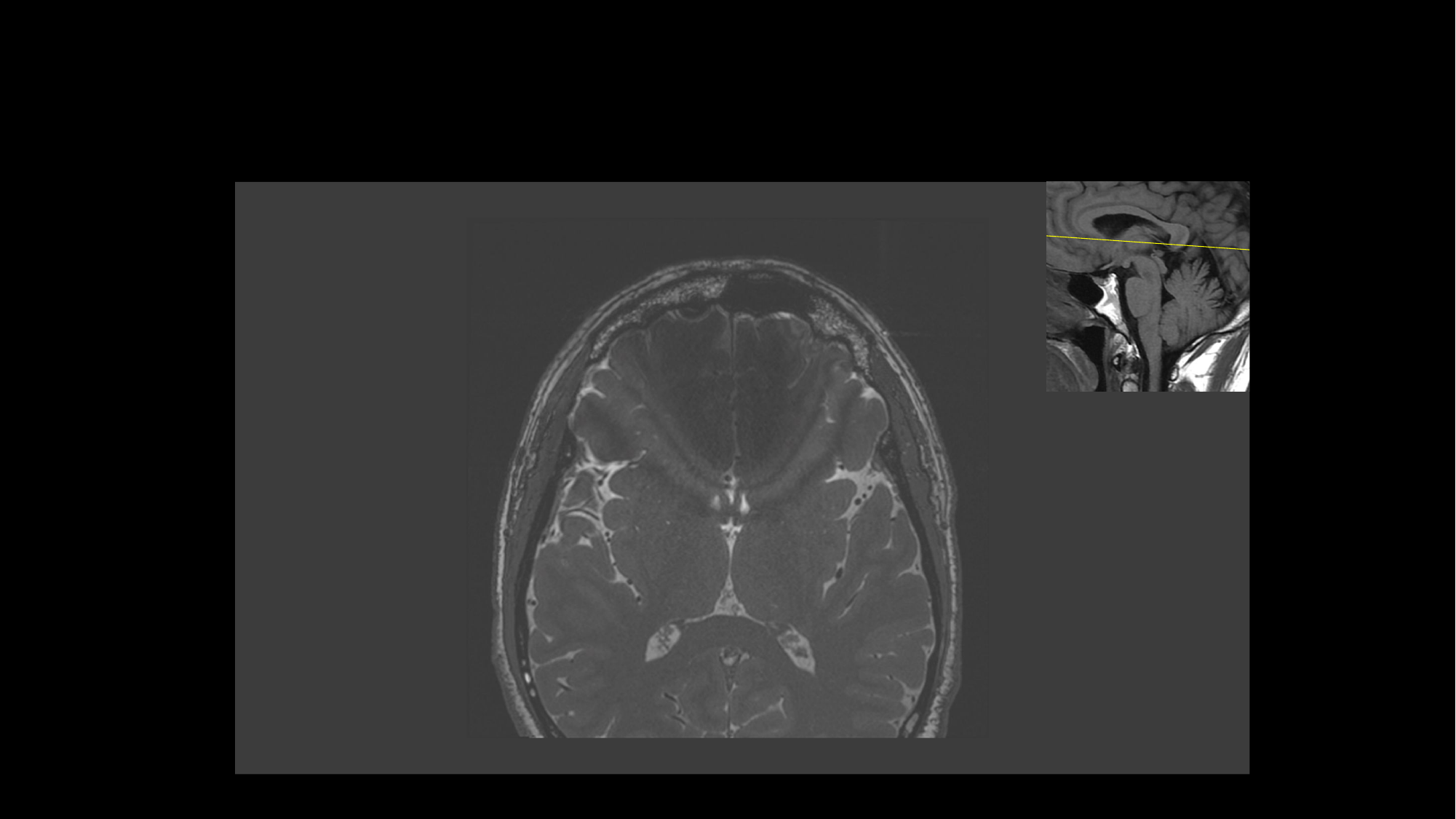

## Slide 183
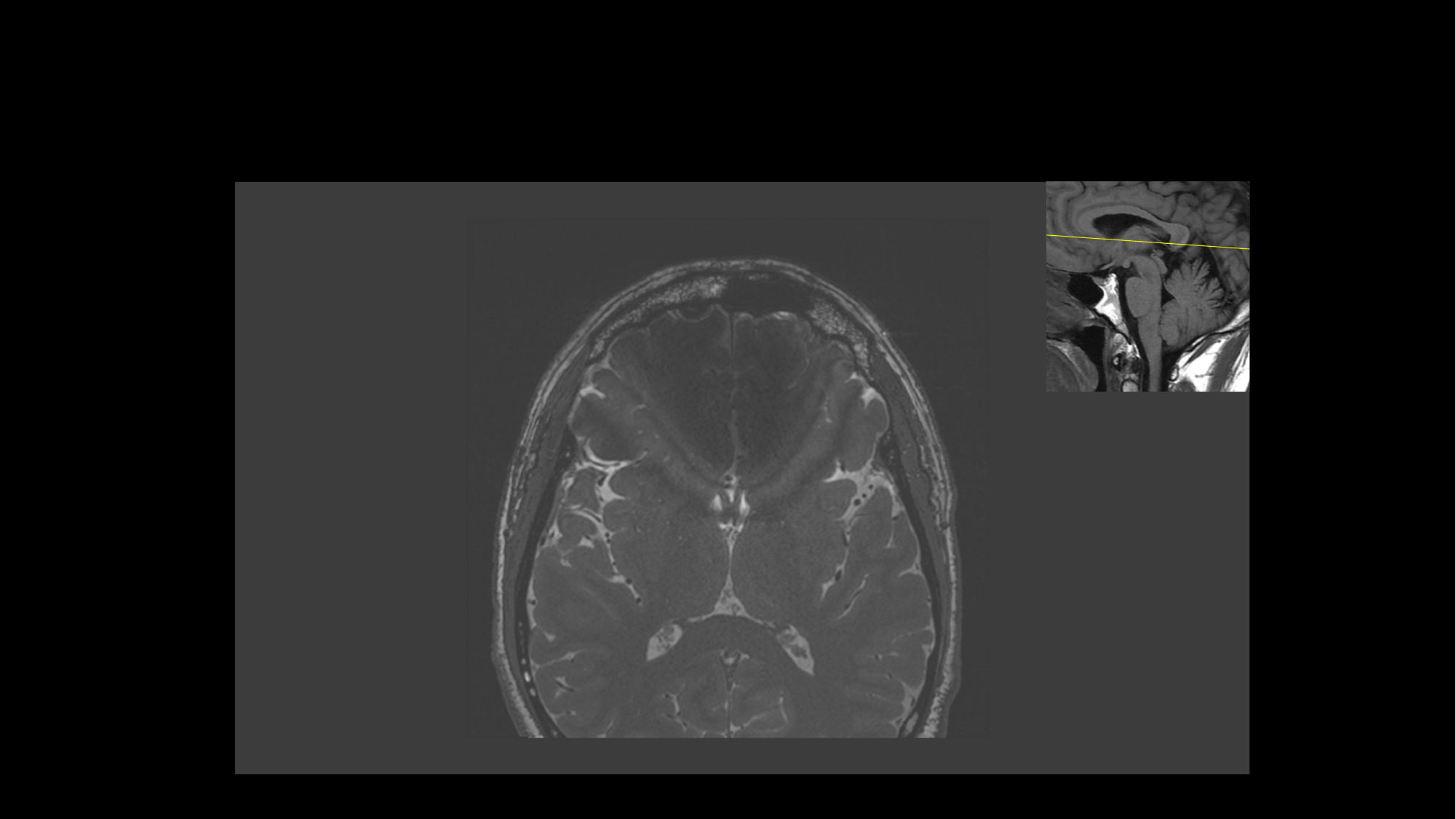

## Slide 184
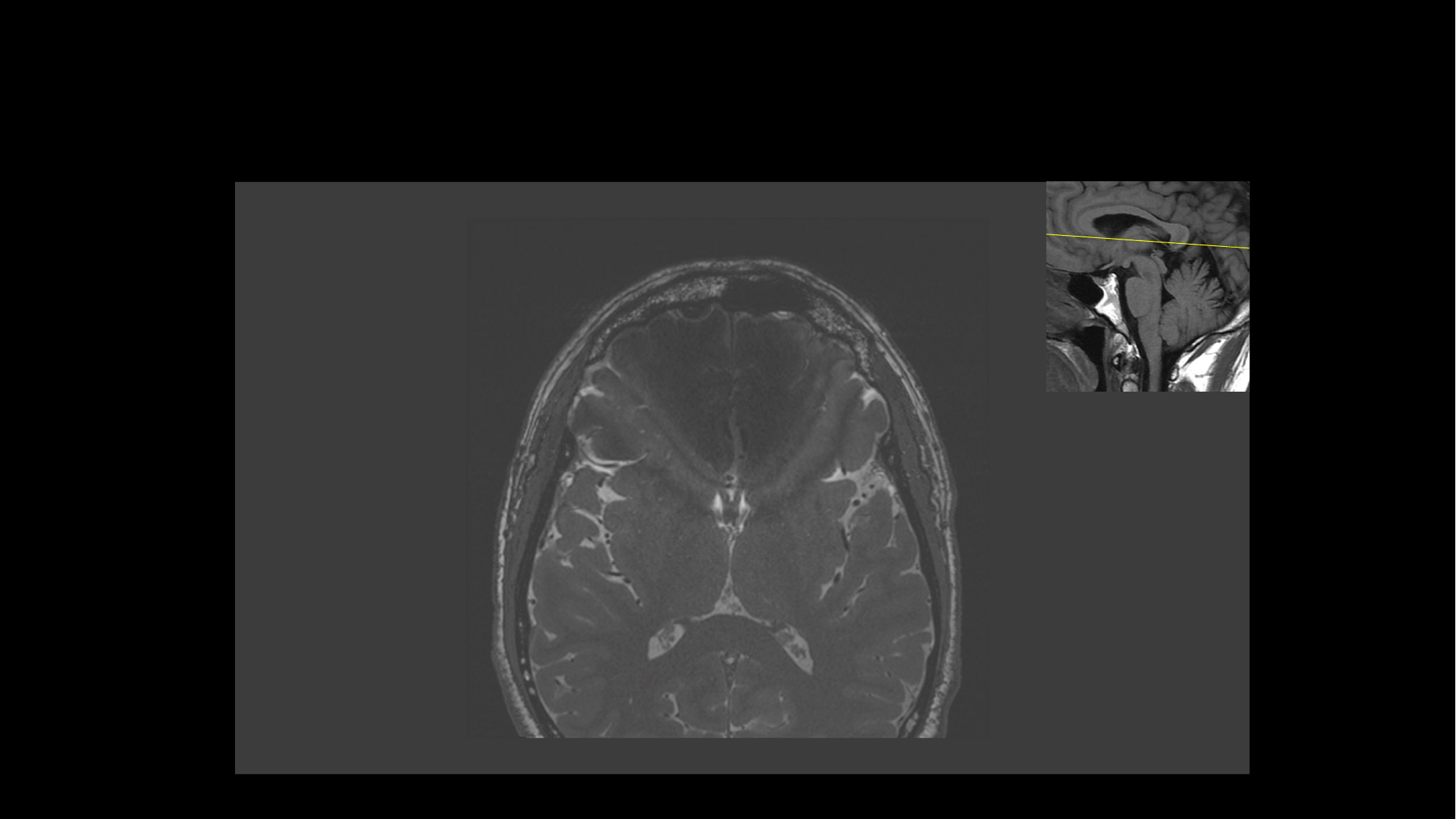

## Slide 185
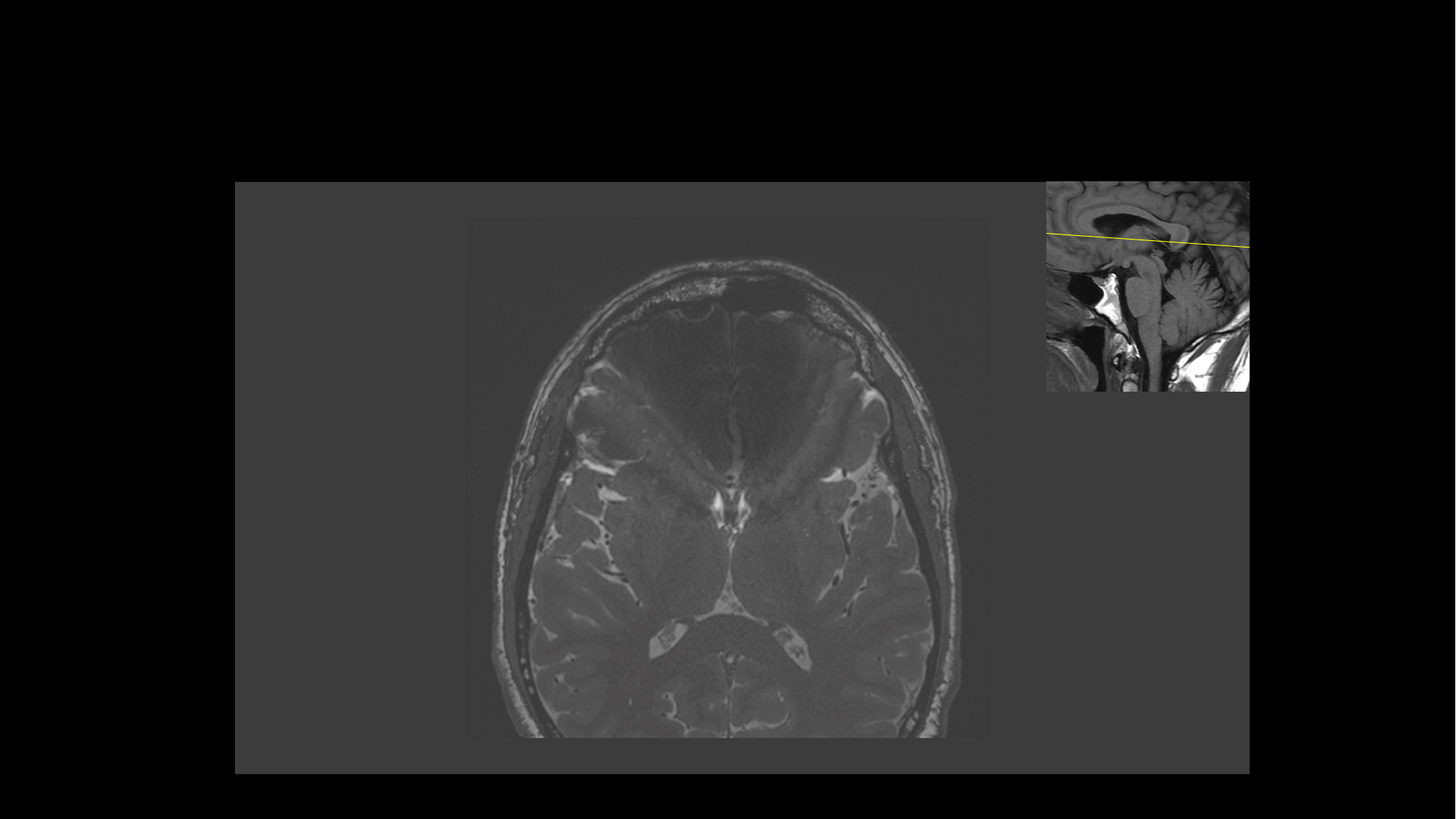

## Slide 186
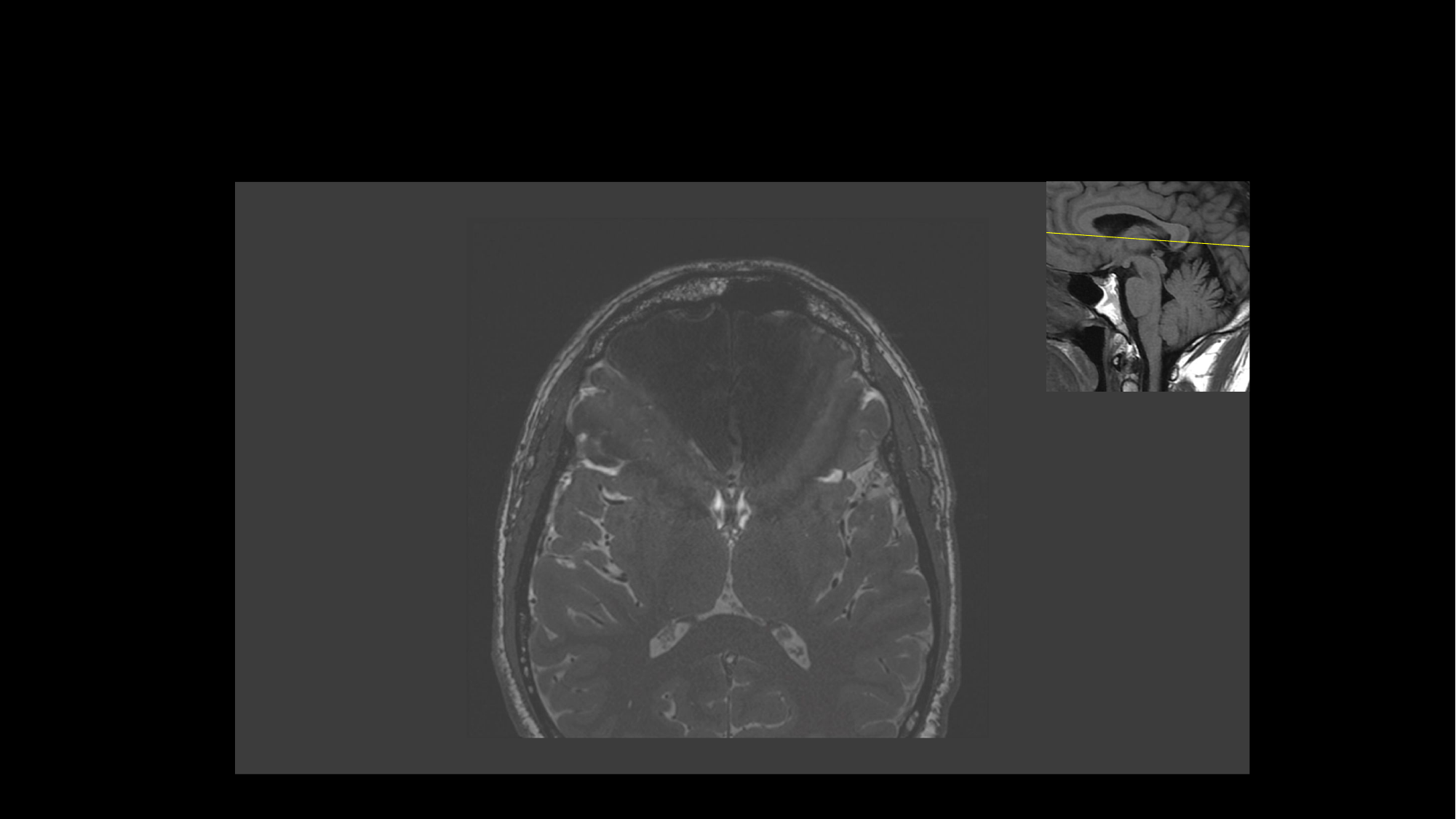

## Slide 187
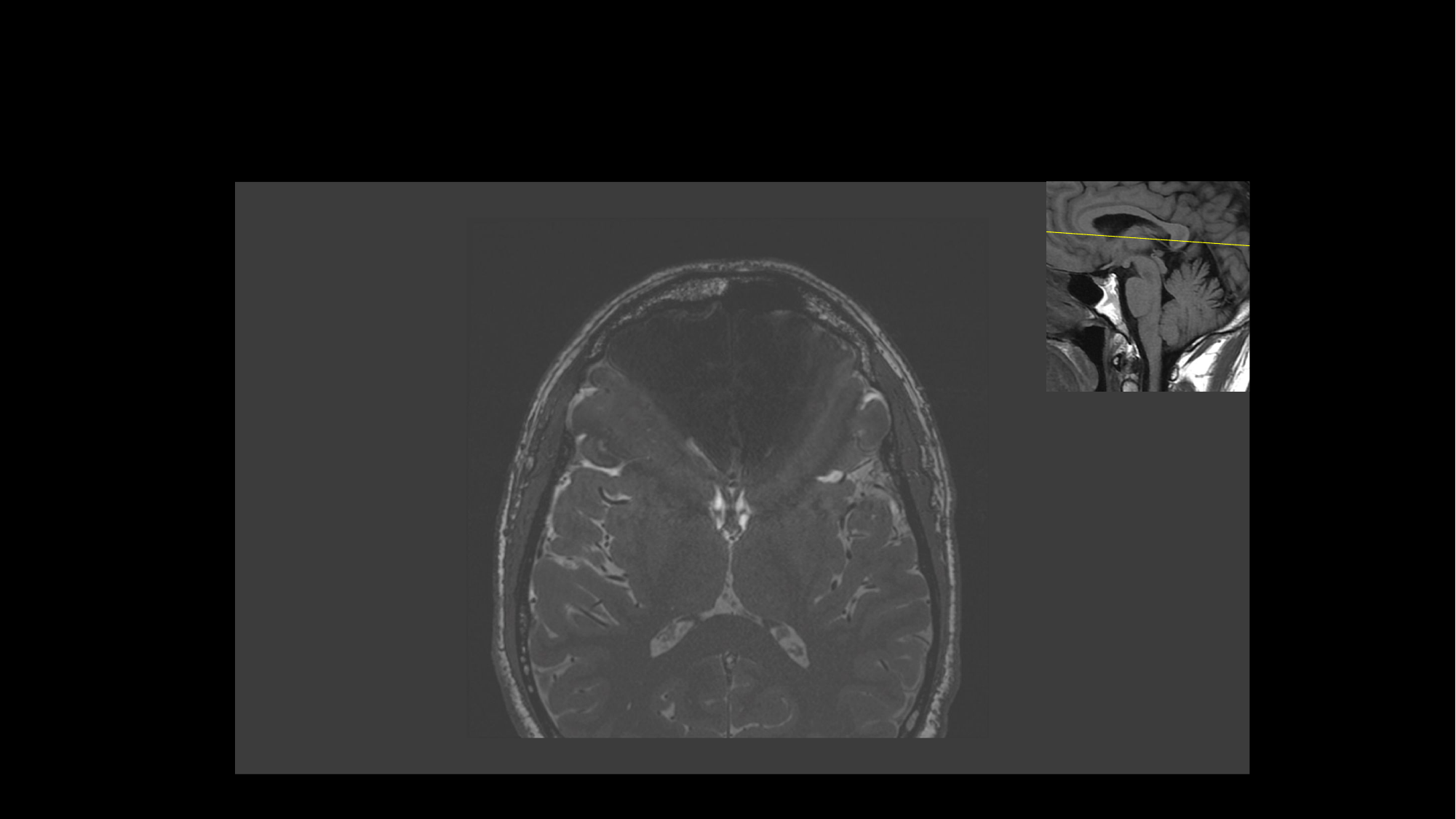

## Slide 188
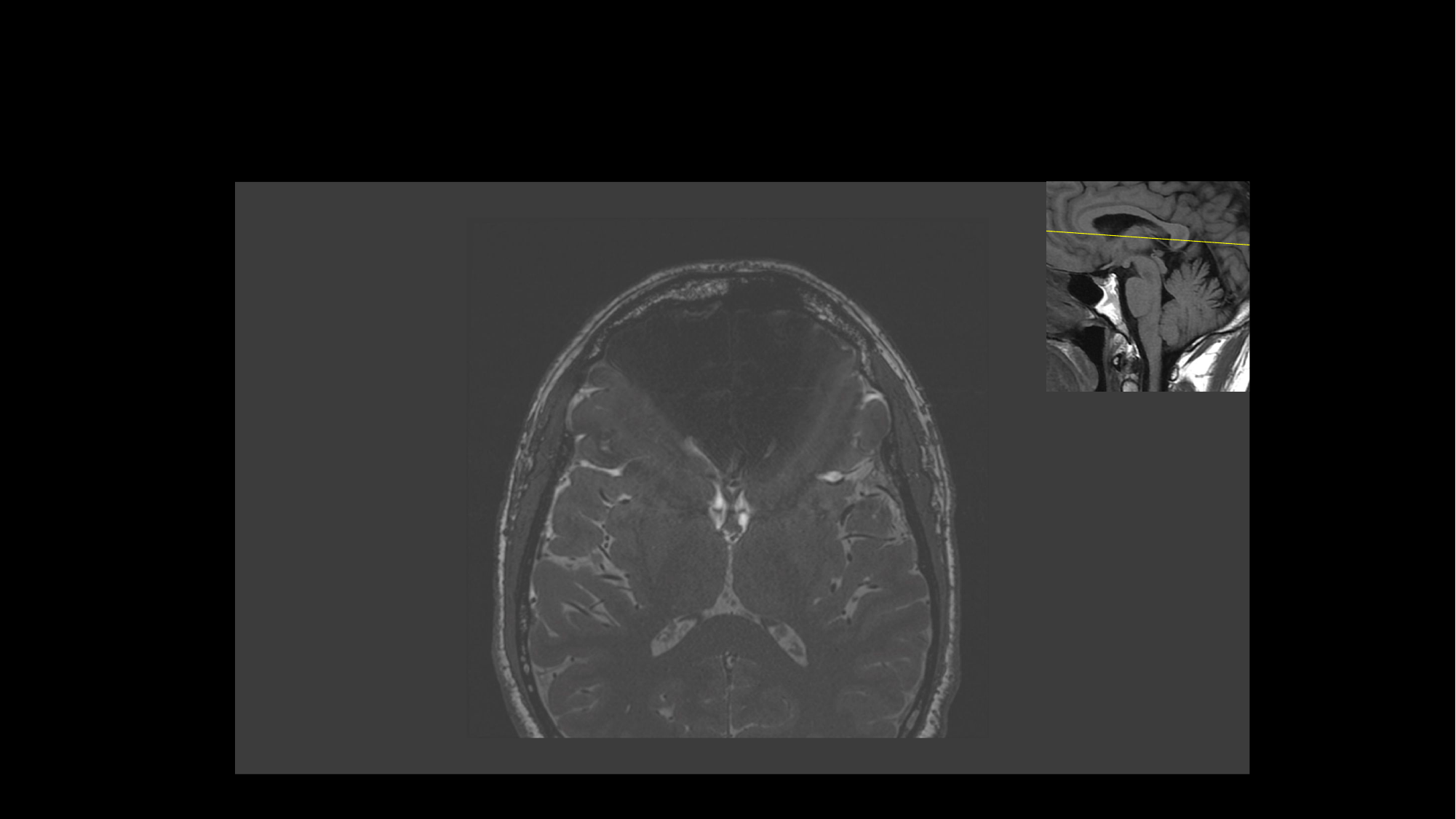

## Slide 189
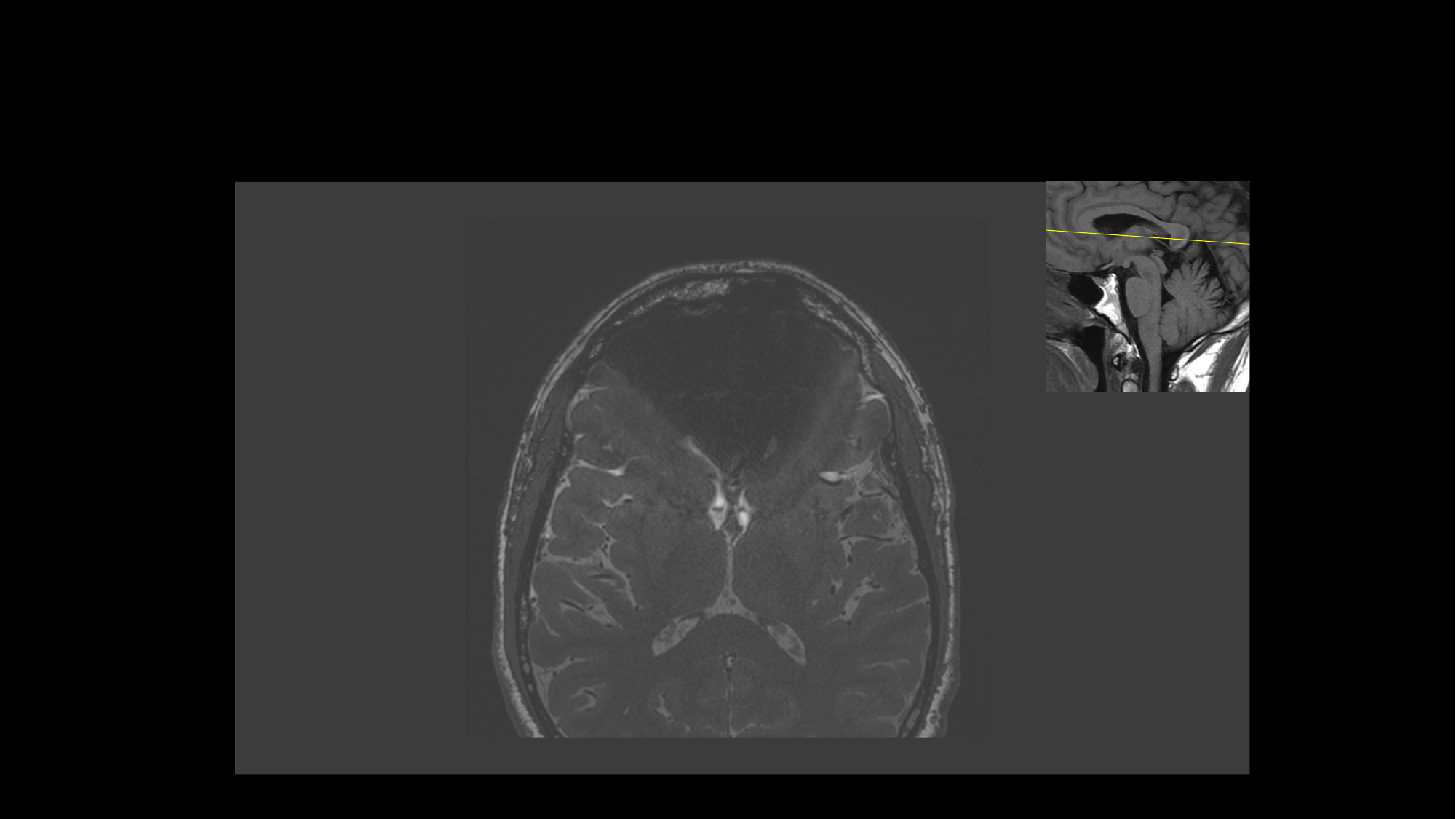

## Slide 190
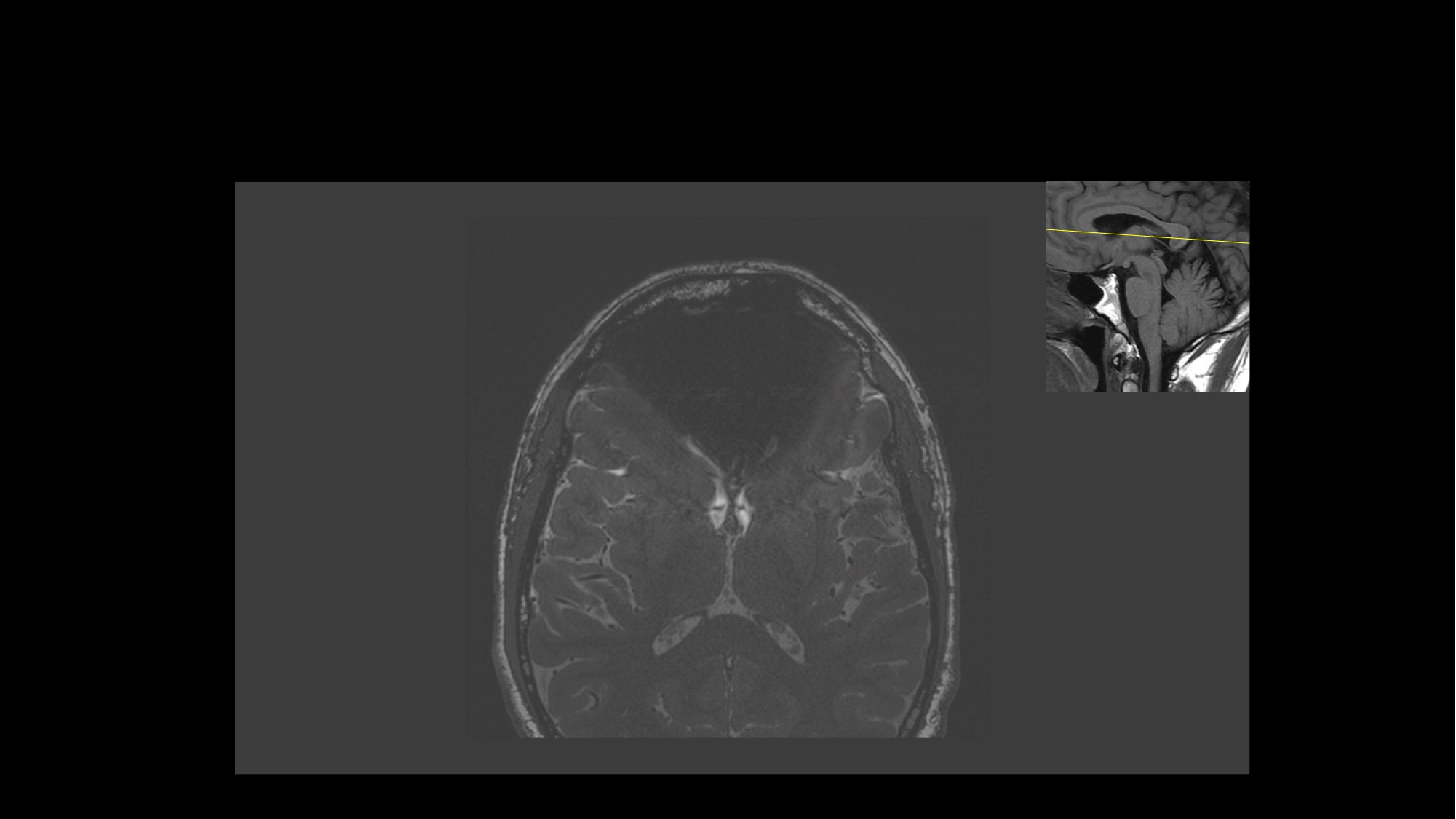

## Slide 191
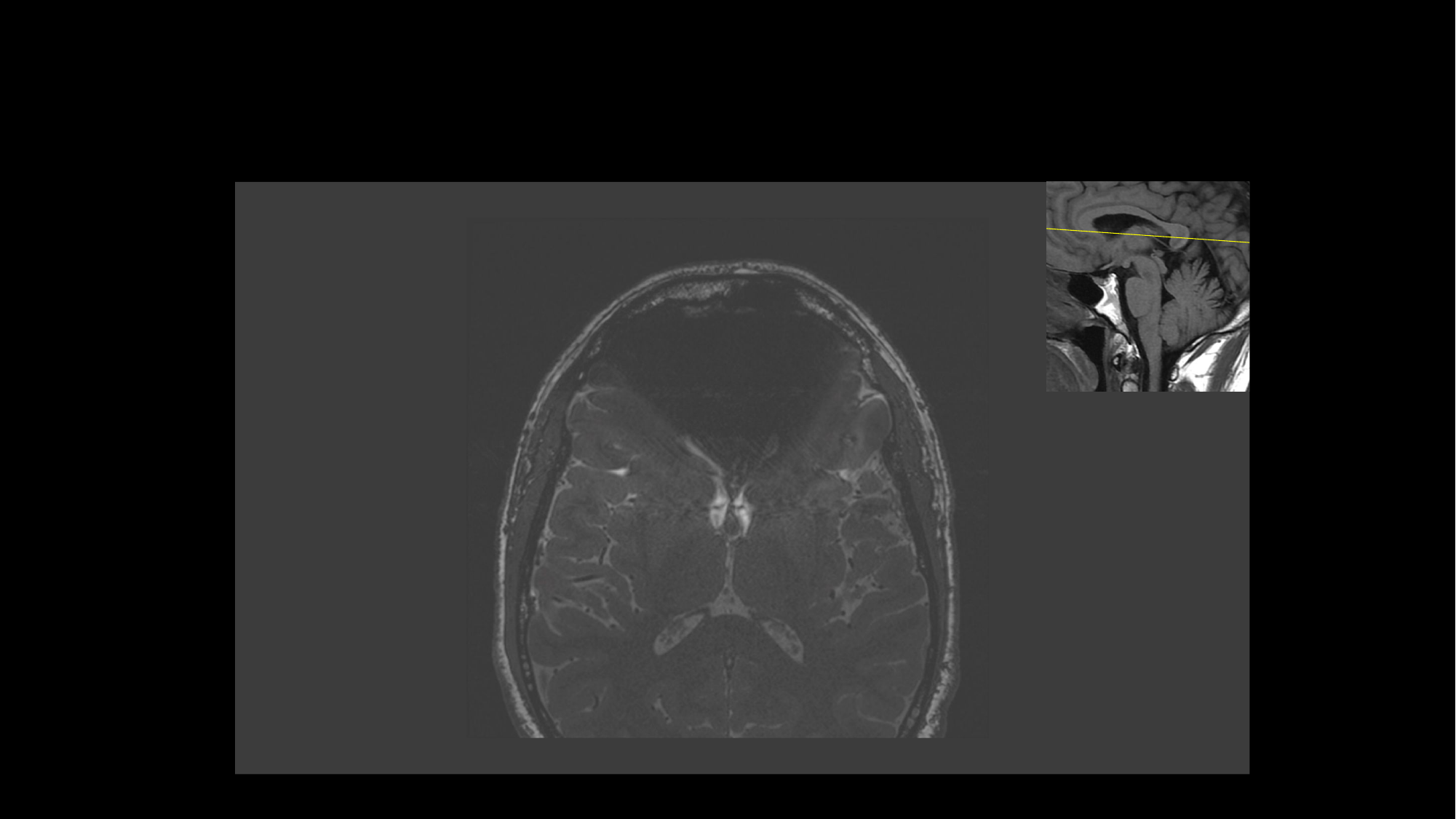

## Slide 192
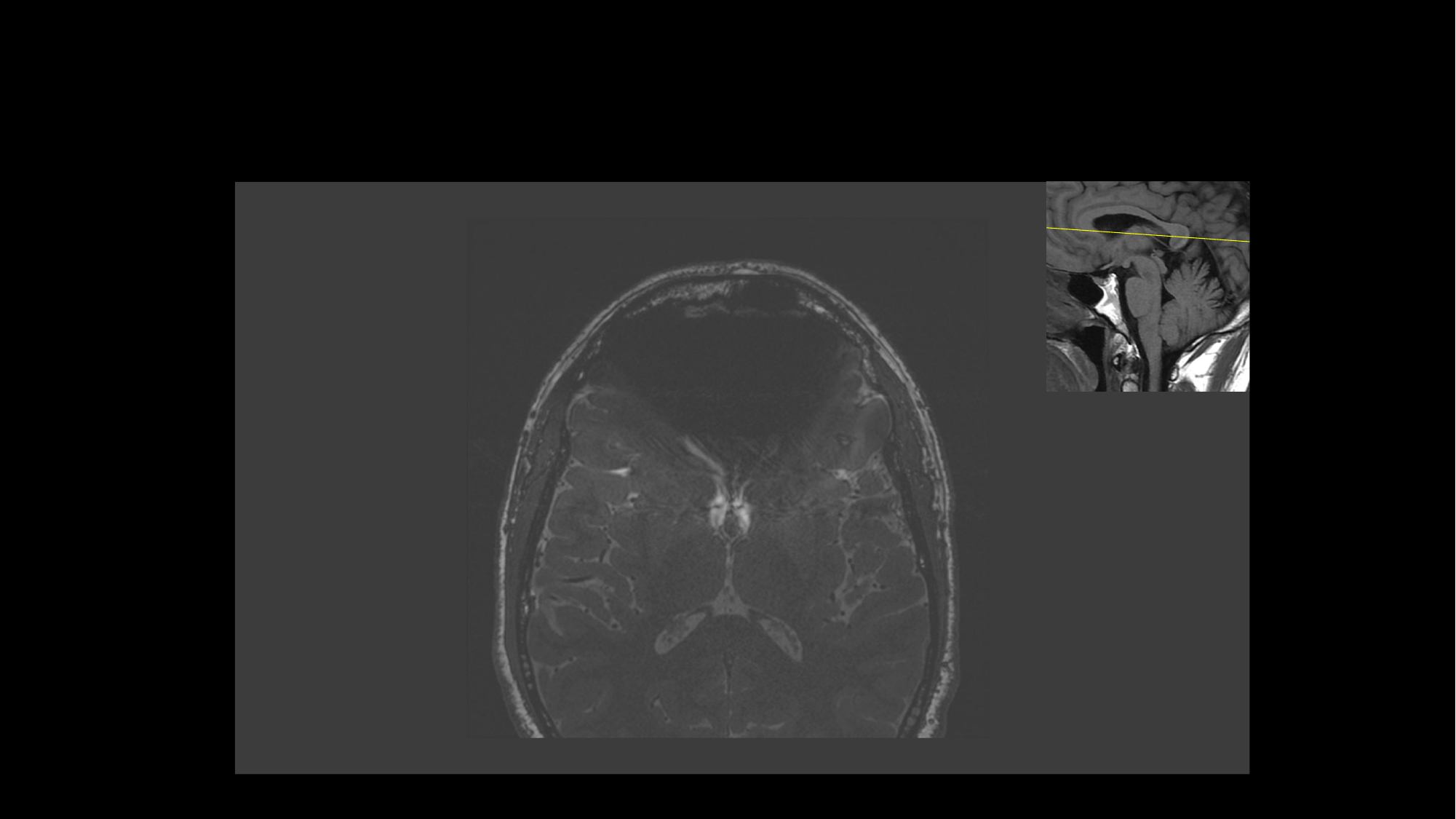

## Slide 193
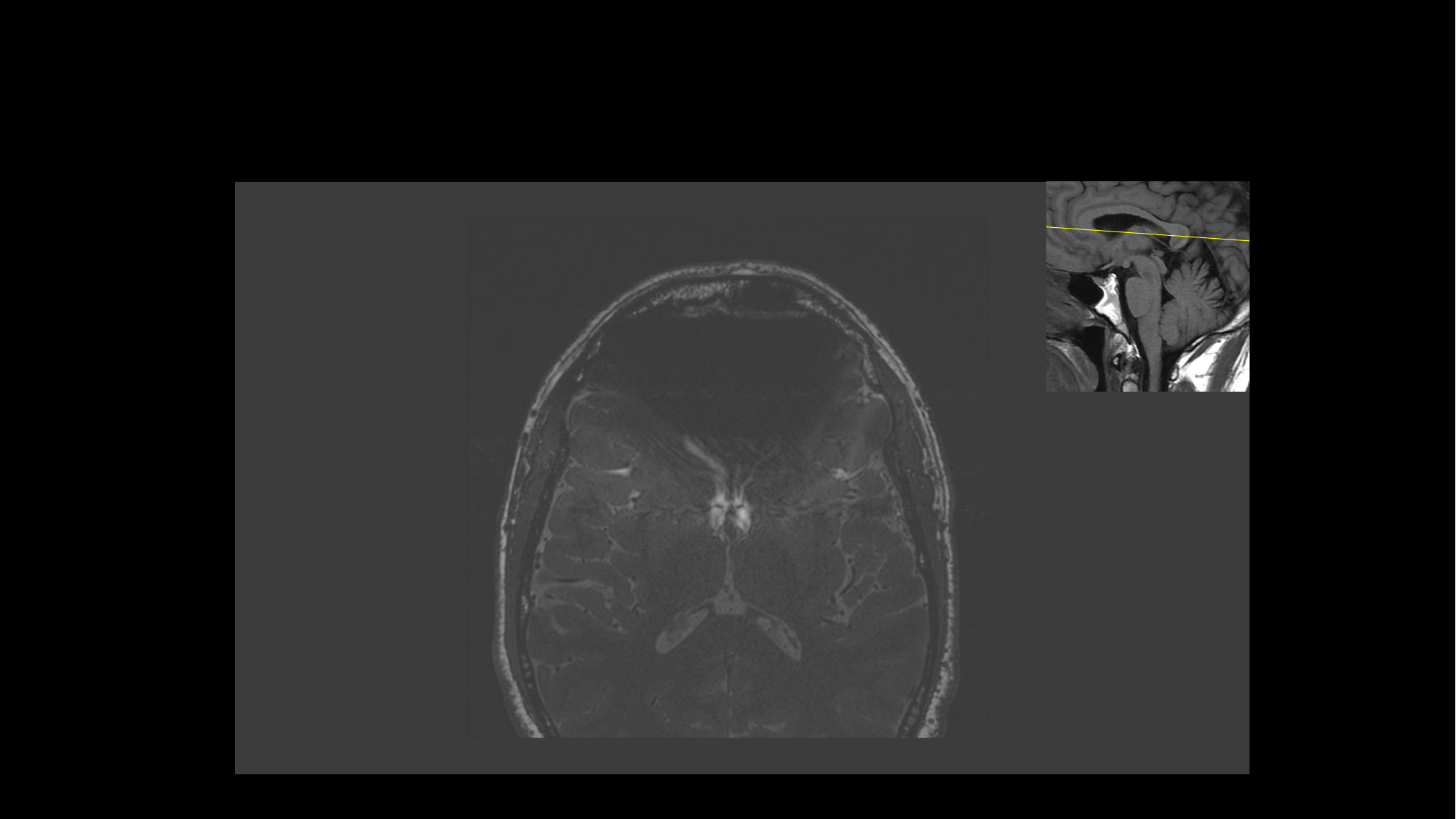

Supplement: Supplementary file 1 — Self-guided Anatomy Review.pptxCranial Nerve Video.mp4Cranial Nerve Lecture.pptxNeuroanatomy Lab.pptxNormal MRI and CT Scans - CT Bone Axials.pptxNormal MRI and CT Scans - T1 Sagittal.pptxNormal MRI and CT Scans - T2 Axial.pptxNormal MRI and CT Scans - T2 SPACE Axial.pptxPre- and Posttest.pptxSatisfaction Survey.docxAppendix Guide.docx [file mep_2374-8265.11261-s001.zip › A. Self-guided Anatomy Review.pptx]
