# Supplementary material for: Cranial Nerve Anatomy Using a Modular and Multimodal Radiologic Approach
Source: MedEdPORTAL. 2022 Jun 10;18:11261. doi: 10.15766/mep_2374-8265.11261 (PMC9184306; doi:10.15766/mep_2374-8265.11261)
Supplement: Supplementary file 1 — Self-guided Anatomy Review.pptxCranial Nerve Video.mp4Cranial Nerve Lecture.pptxNeuroanatomy Lab.pptxNormal MRI and CT Scans - CT Bone Axials.pptxNormal MRI and CT Scans - T1 Sagittal.pptxNormal MRI and CT Scans - T2 Axial.pptxNormal MRI and CT Scans - T2 SPACE Axial.pptxPre- and Posttest.pptxSatisfaction Survey.docxAppendix Guide.docx [file mep_2374-8265.11261-s001.zip › C. Cranial Nerve Lecture.pptx]

## Slide 1
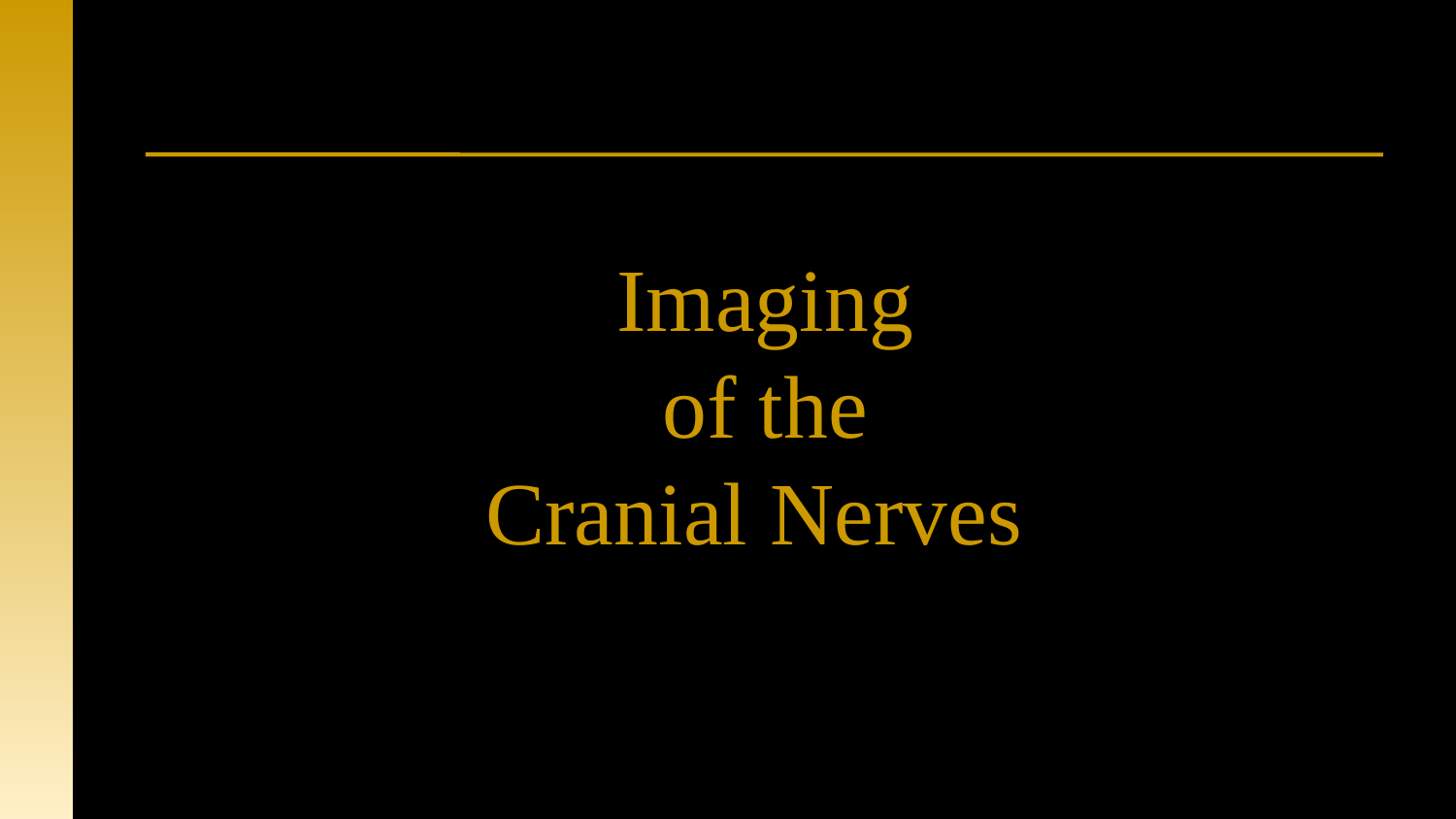

# Imaging of the Cranial Nerves

## Slide 2
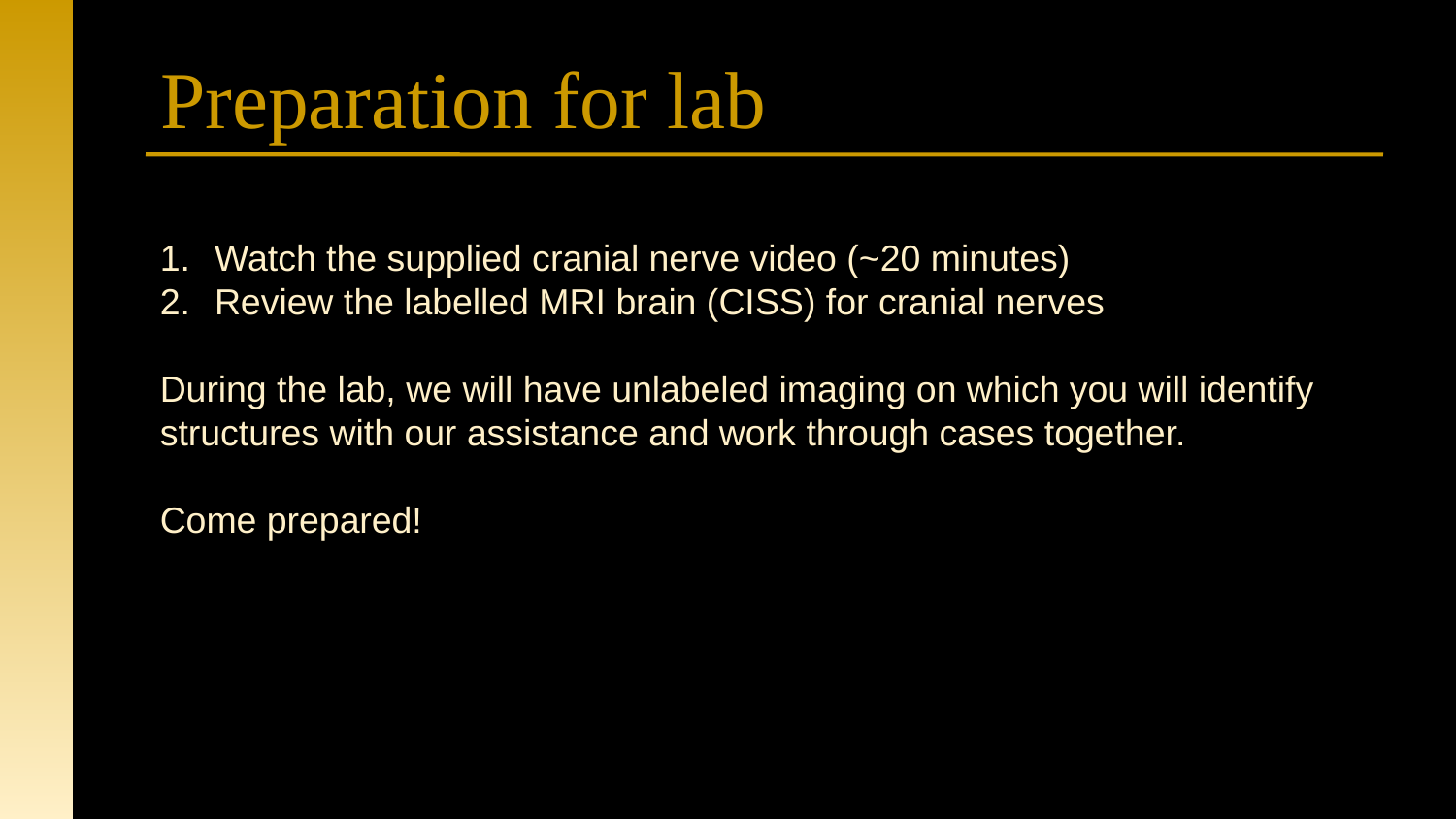

# Preparation for lab
Watch the supplied cranial nerve video (~20 minutes)
Review the labelled MRI brain (CISS) for cranial nerves
During the lab, we will have unlabeled imaging on which you will identify structures with our assistance and work through cases together.
Come prepared!

## Slide 3
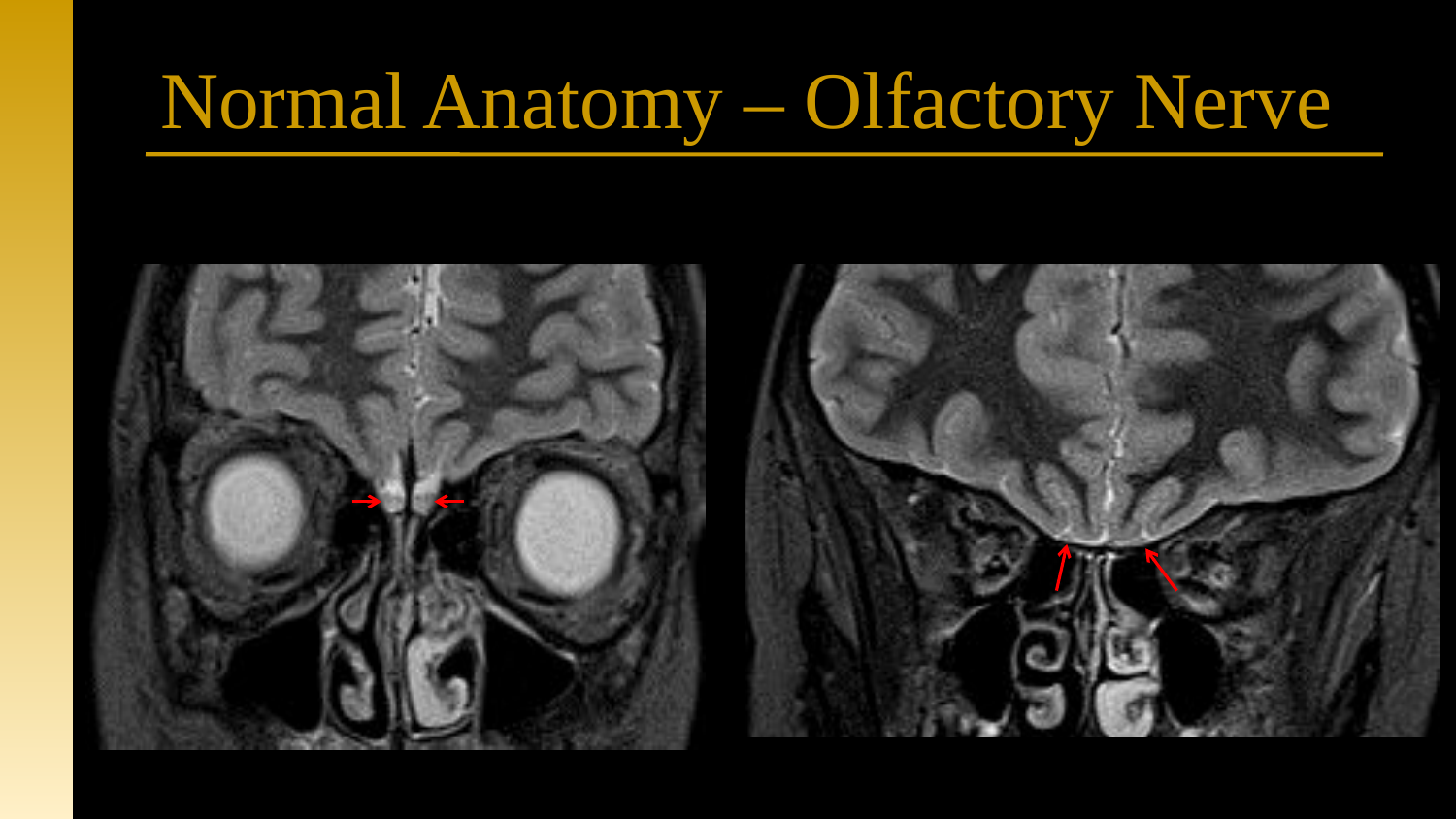

# Normal Anatomy – Olfactory Nerve

## Slide 4
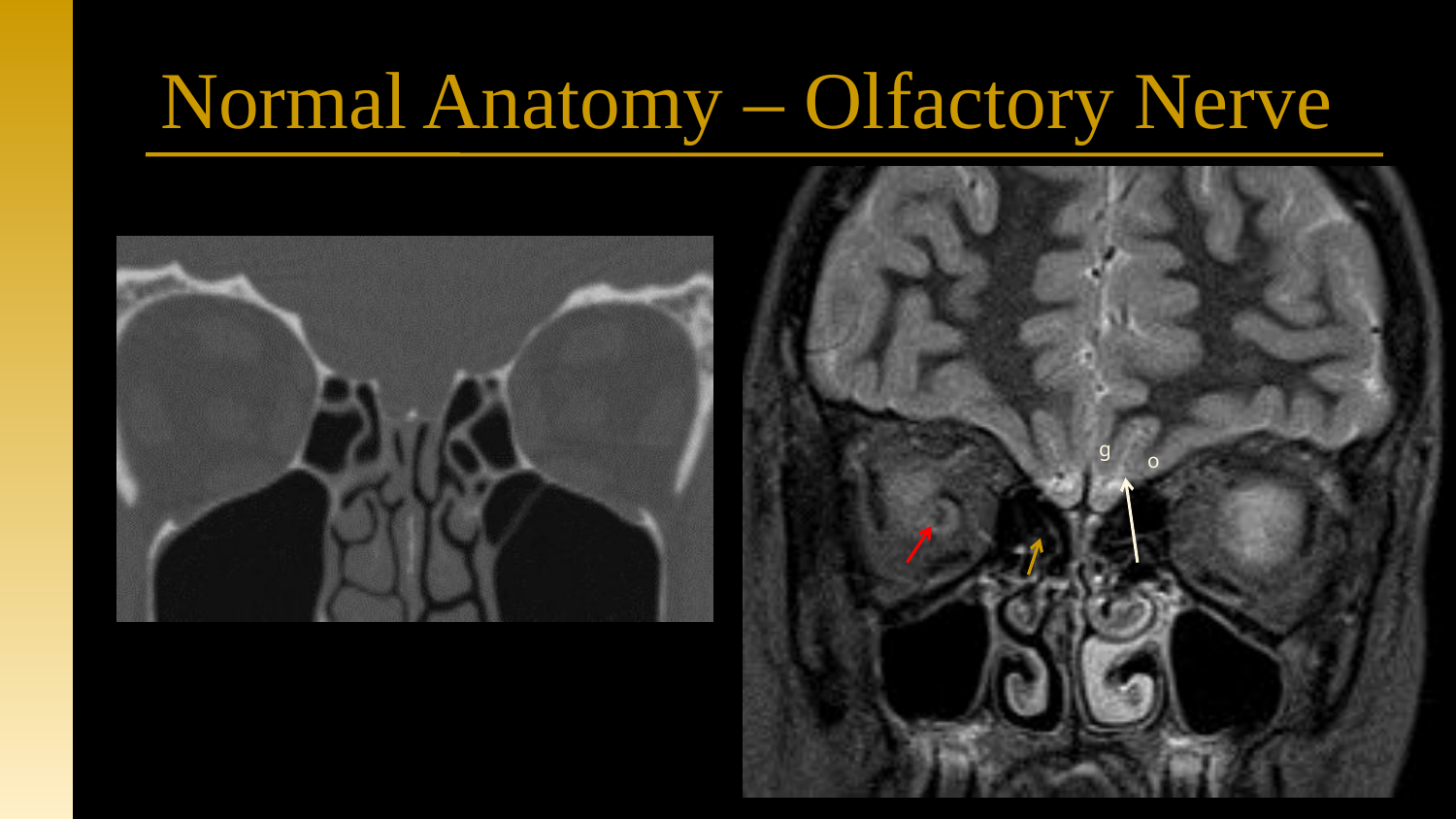

# Normal Anatomy – Olfactory Nerve
g
o

## Slide 5
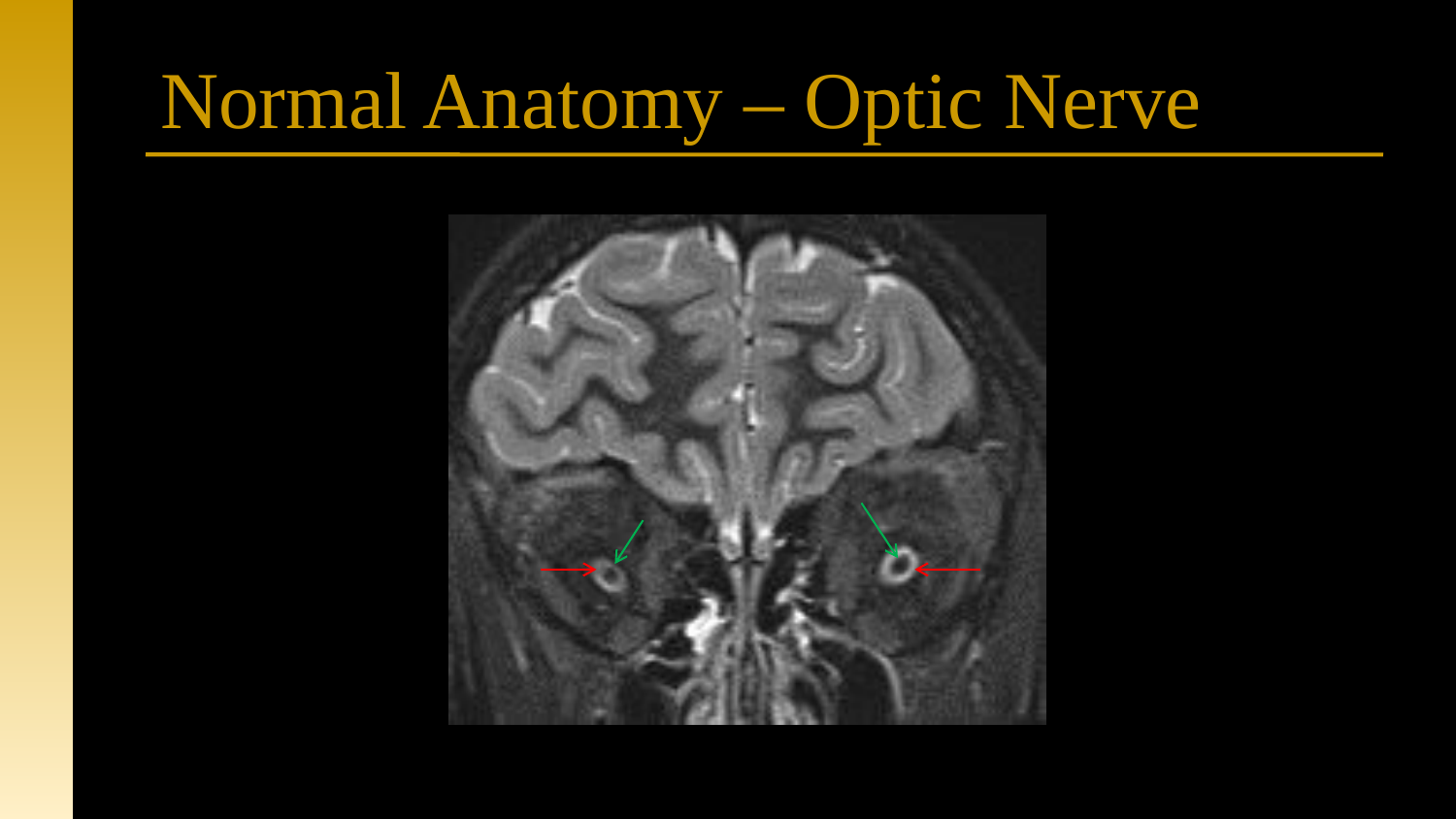

# Normal Anatomy – Optic Nerve

## Slide 6
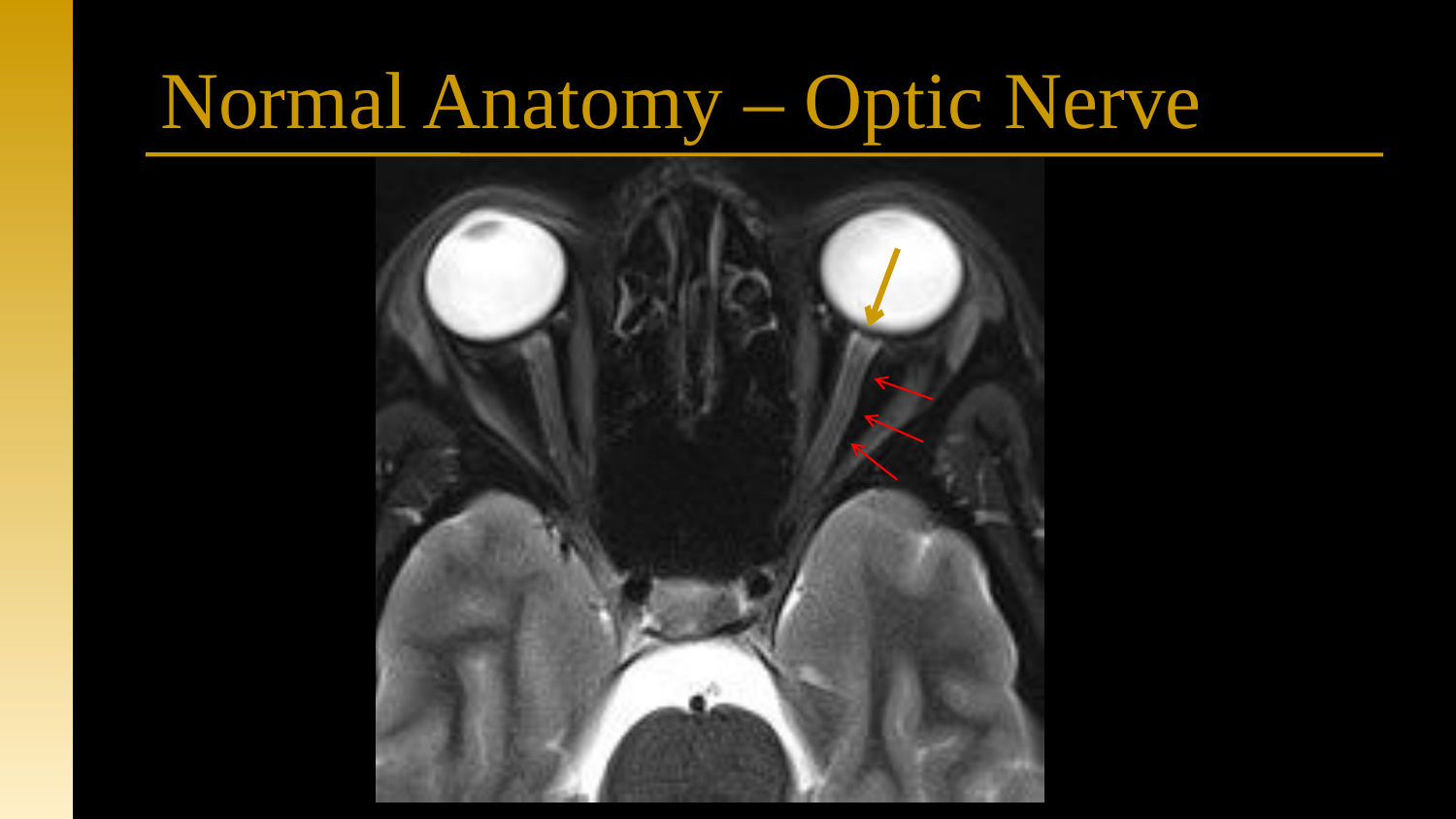

# Normal Anatomy – Optic Nerve

## Slide 7
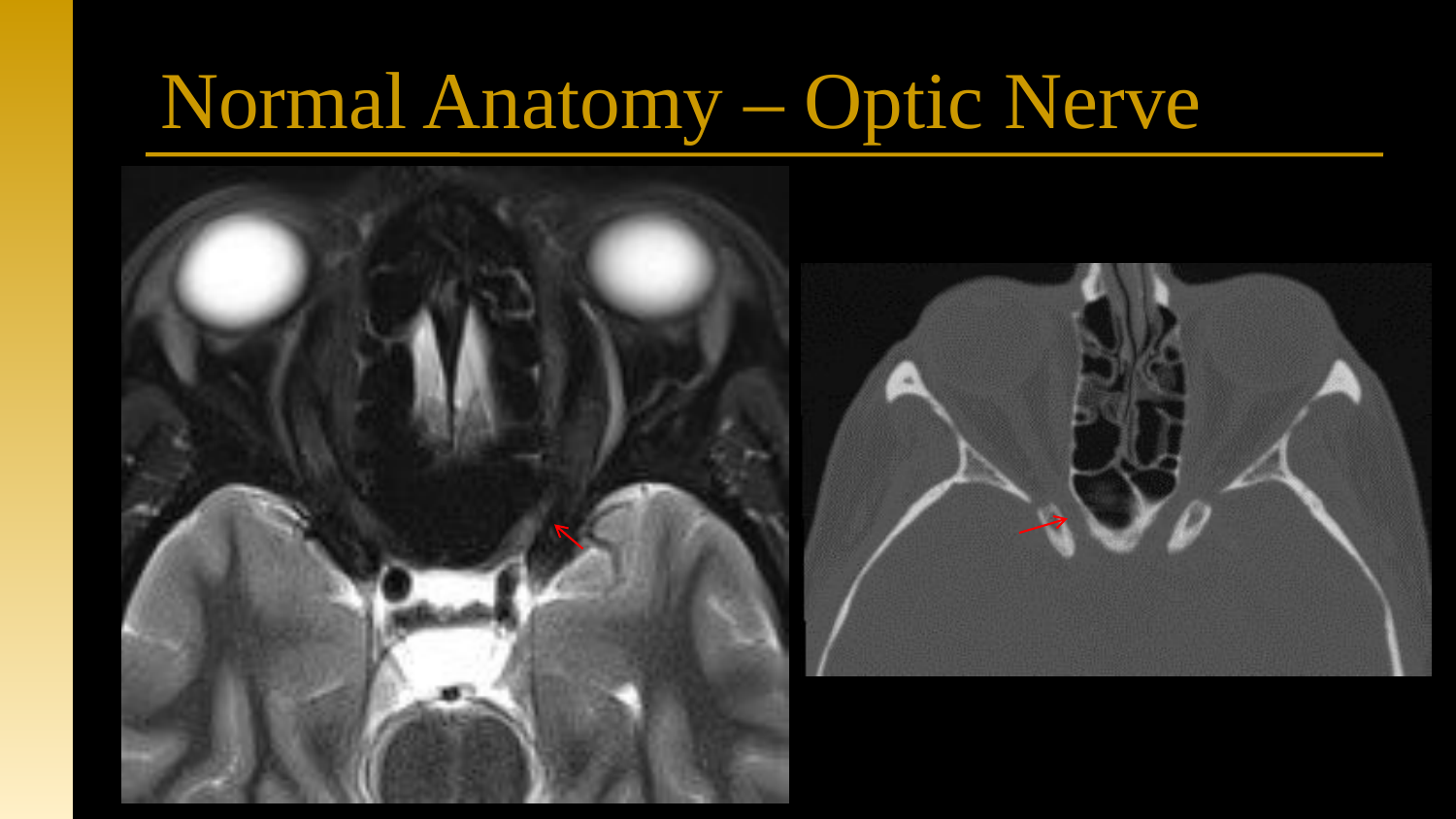

# Normal Anatomy – Optic Nerve

## Slide 8
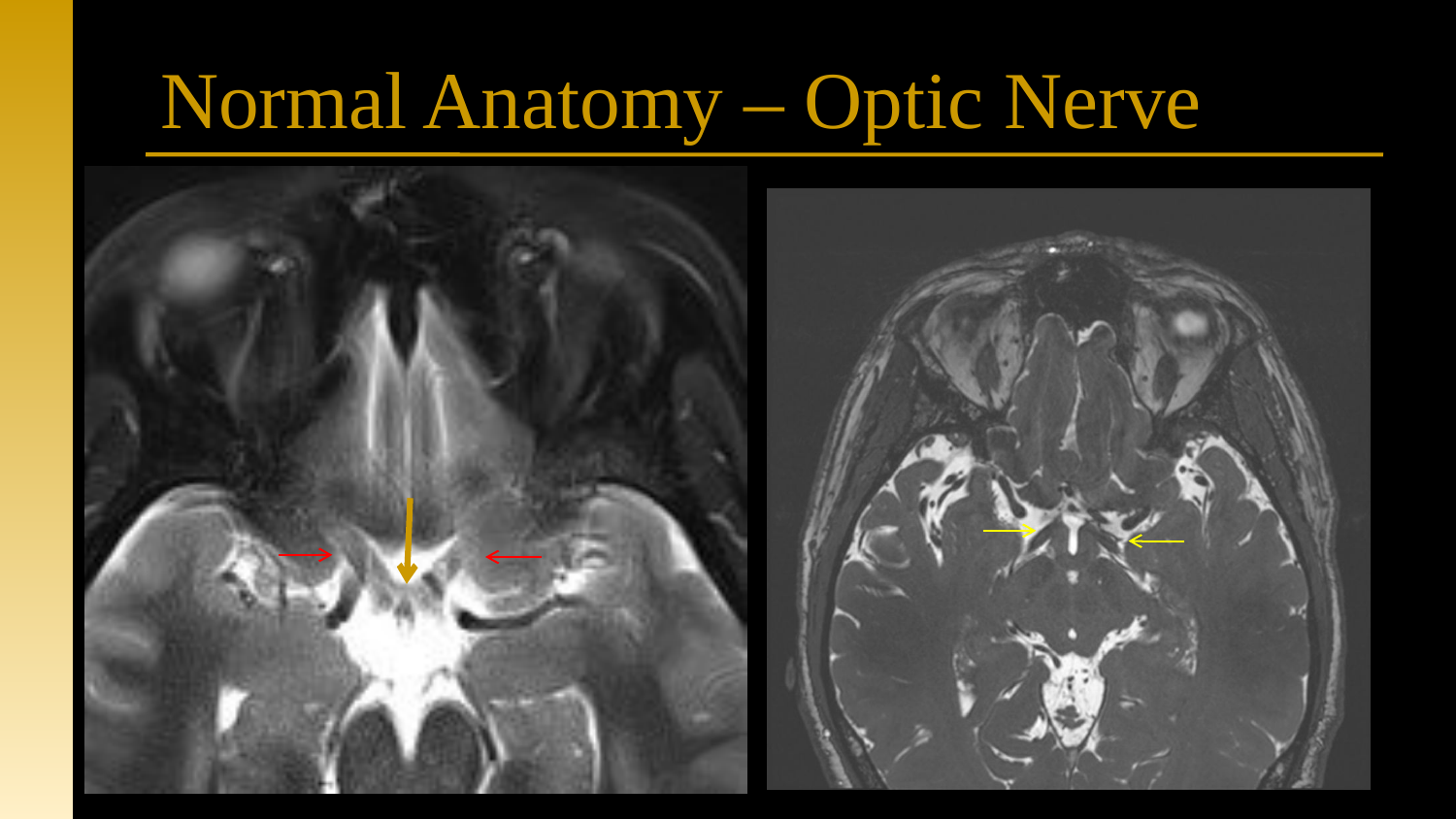

# Normal Anatomy – Optic Nerve
Image of optic tract?

## Slide 9
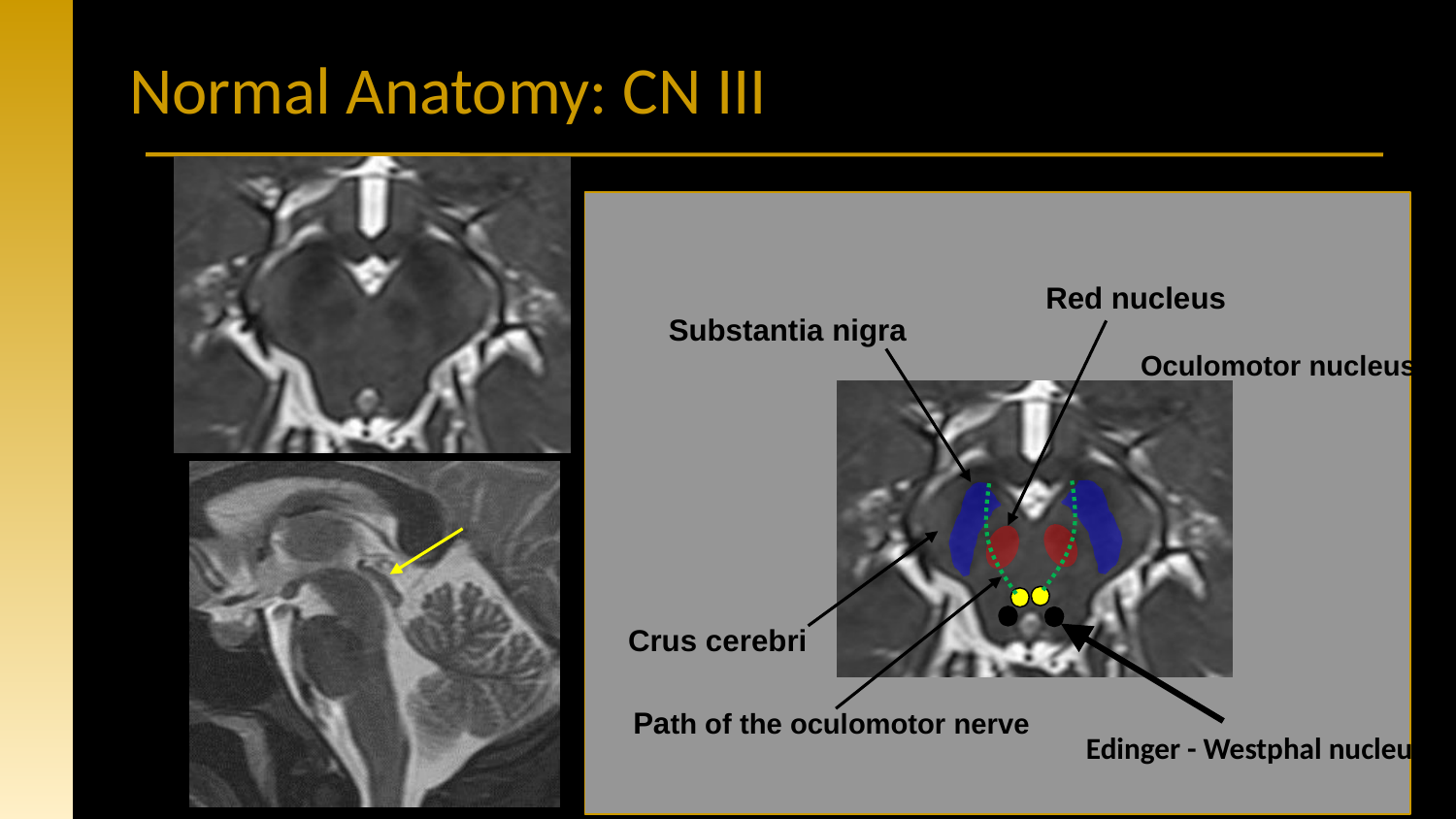

Normal Anatomy: CN III
Red nucleus
Substantia nigra
Oculomotor nucleus
Crus cerebri
Path of the oculomotor nerve
Edinger - Westphal nucleus

## Slide 10
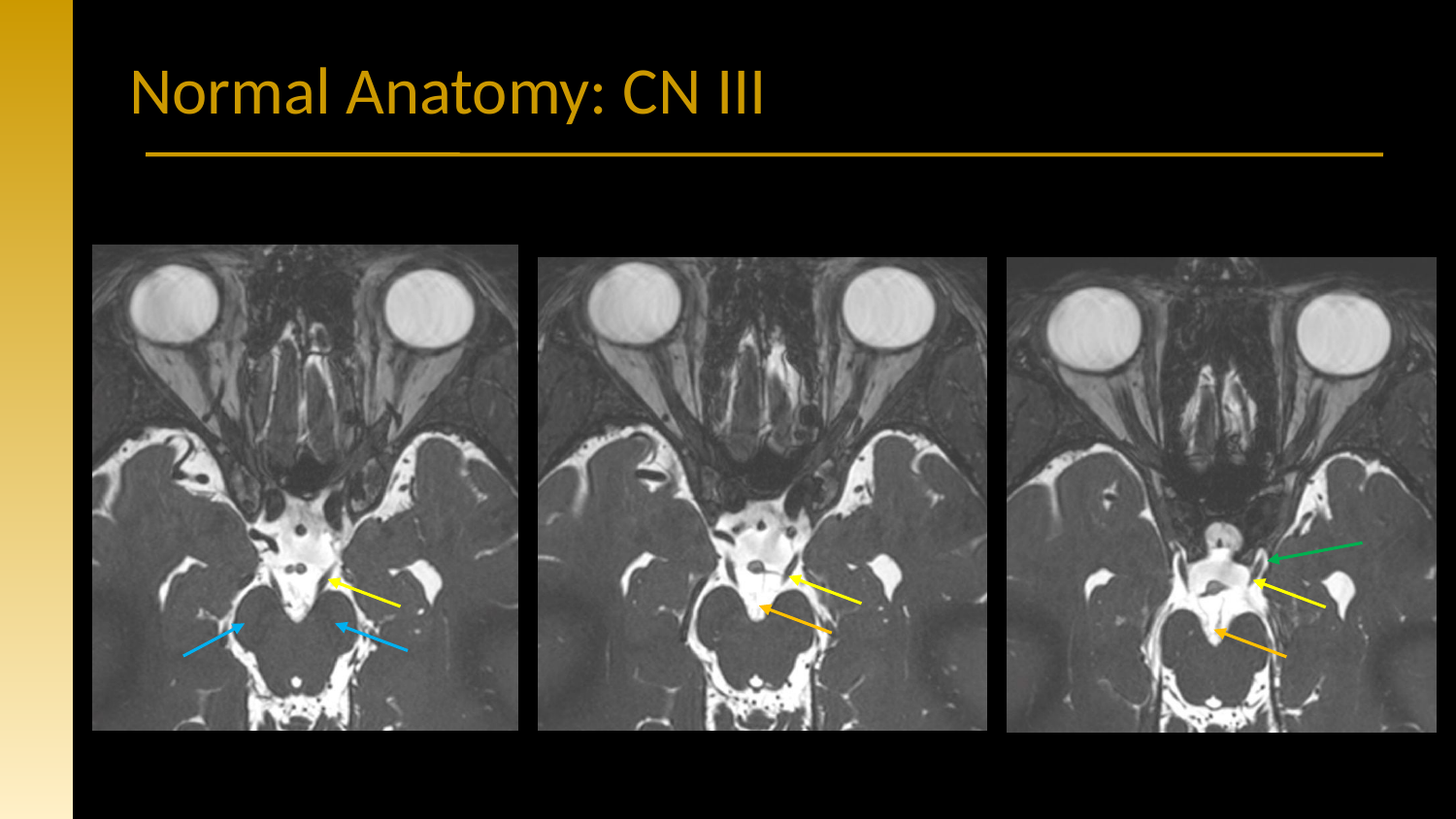

Normal Anatomy: CN III

## Slide 11
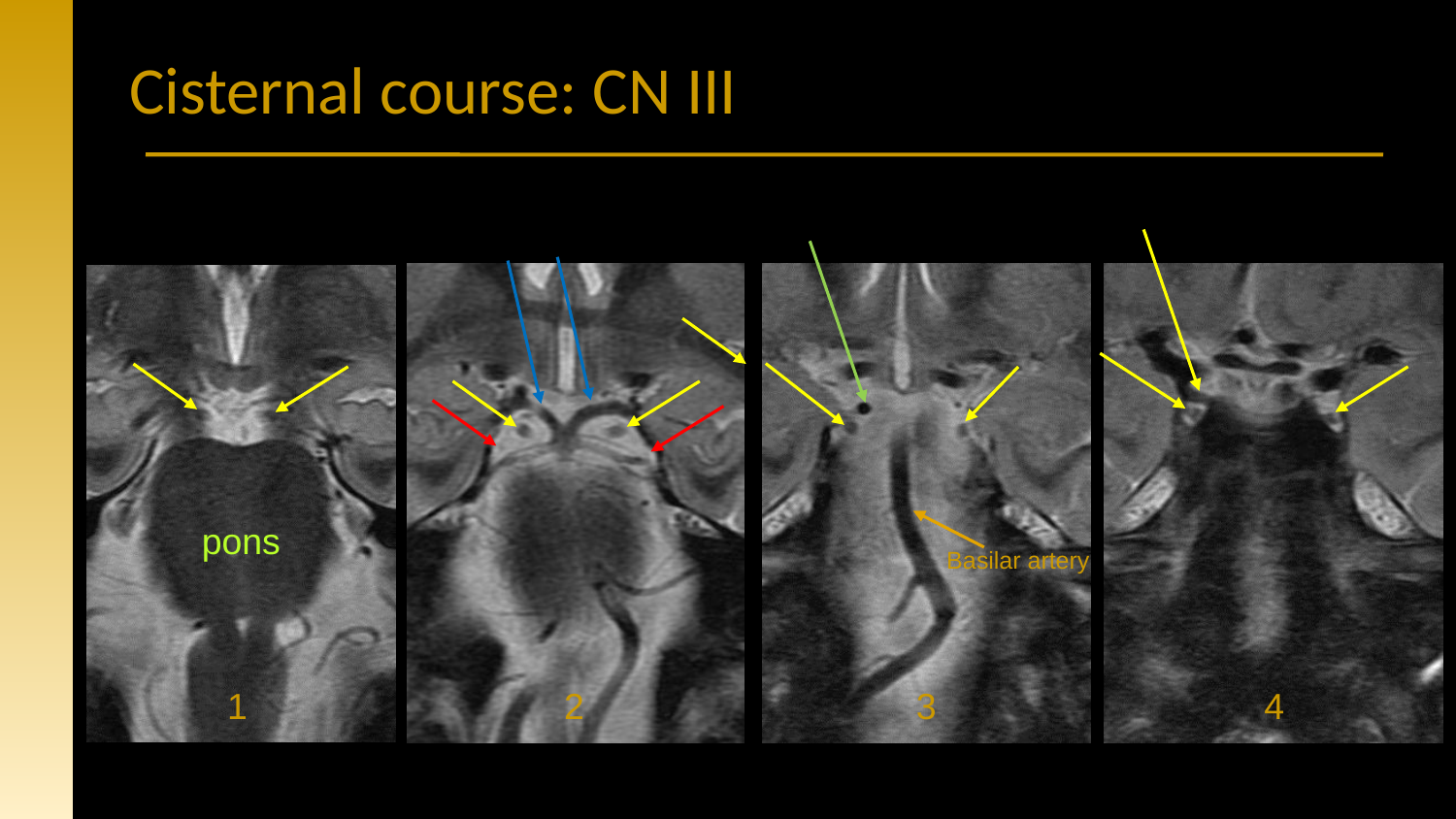

Cisternal course: CN III
pons
Basilar artery
1
2
3
4

## Slide 12
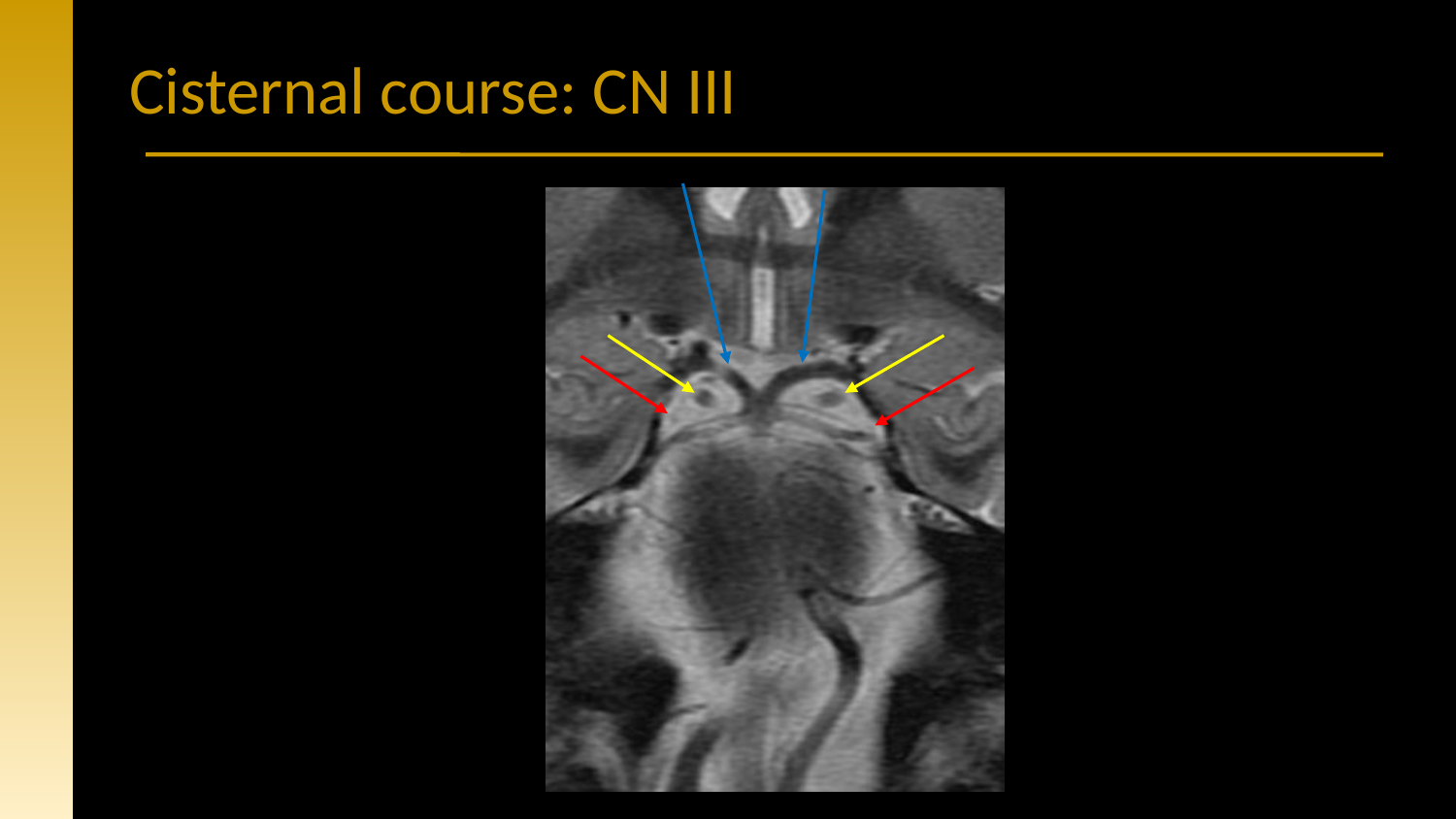

Cisternal course: CN III

## Slide 13
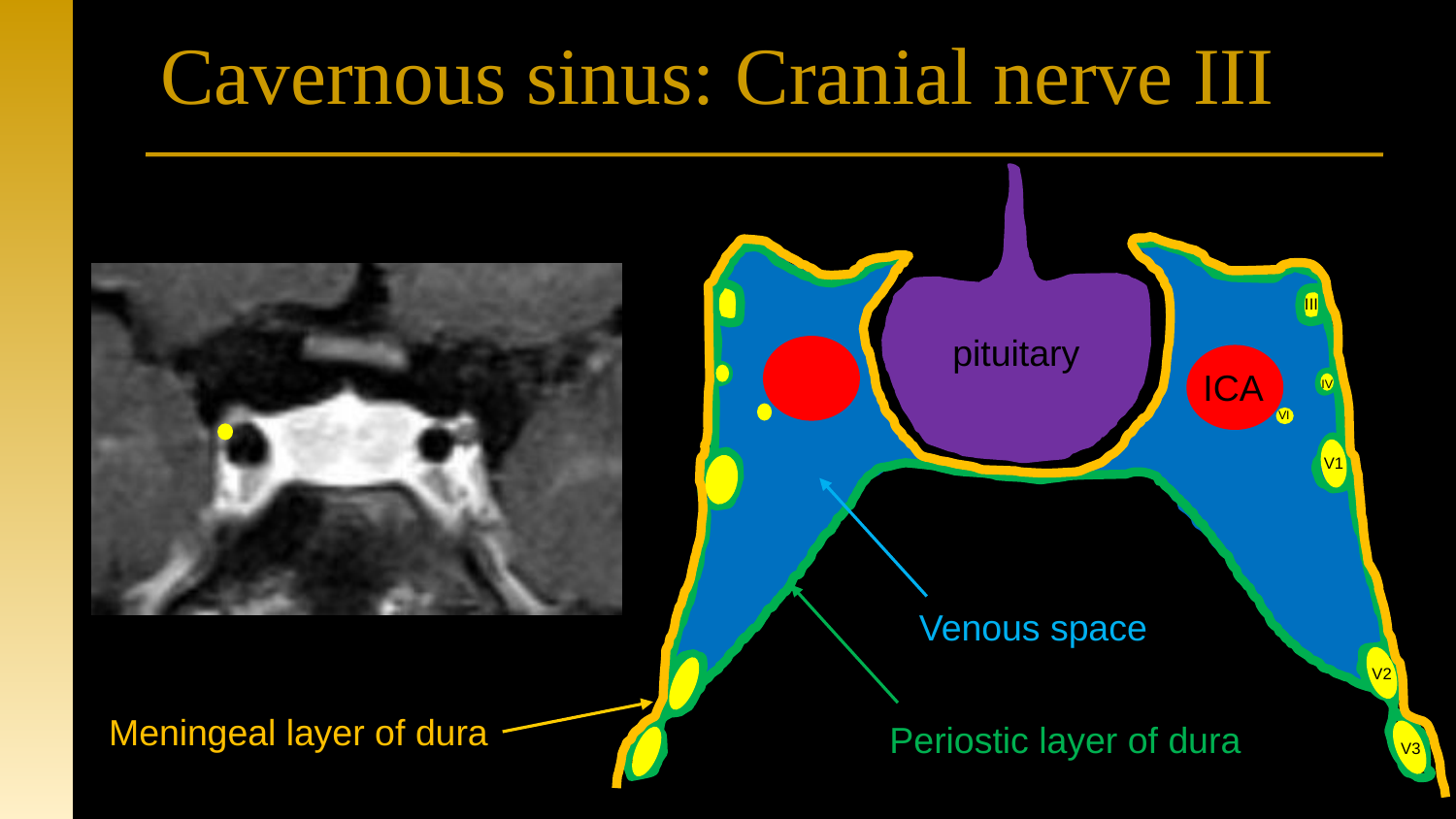

# Cavernous sinus: Cranial nerve III
III
pituitary
ICA
IV
VI
V1
Venous space
V2
Meningeal layer of dura
Periostic layer of dura
V3

## Slide 14
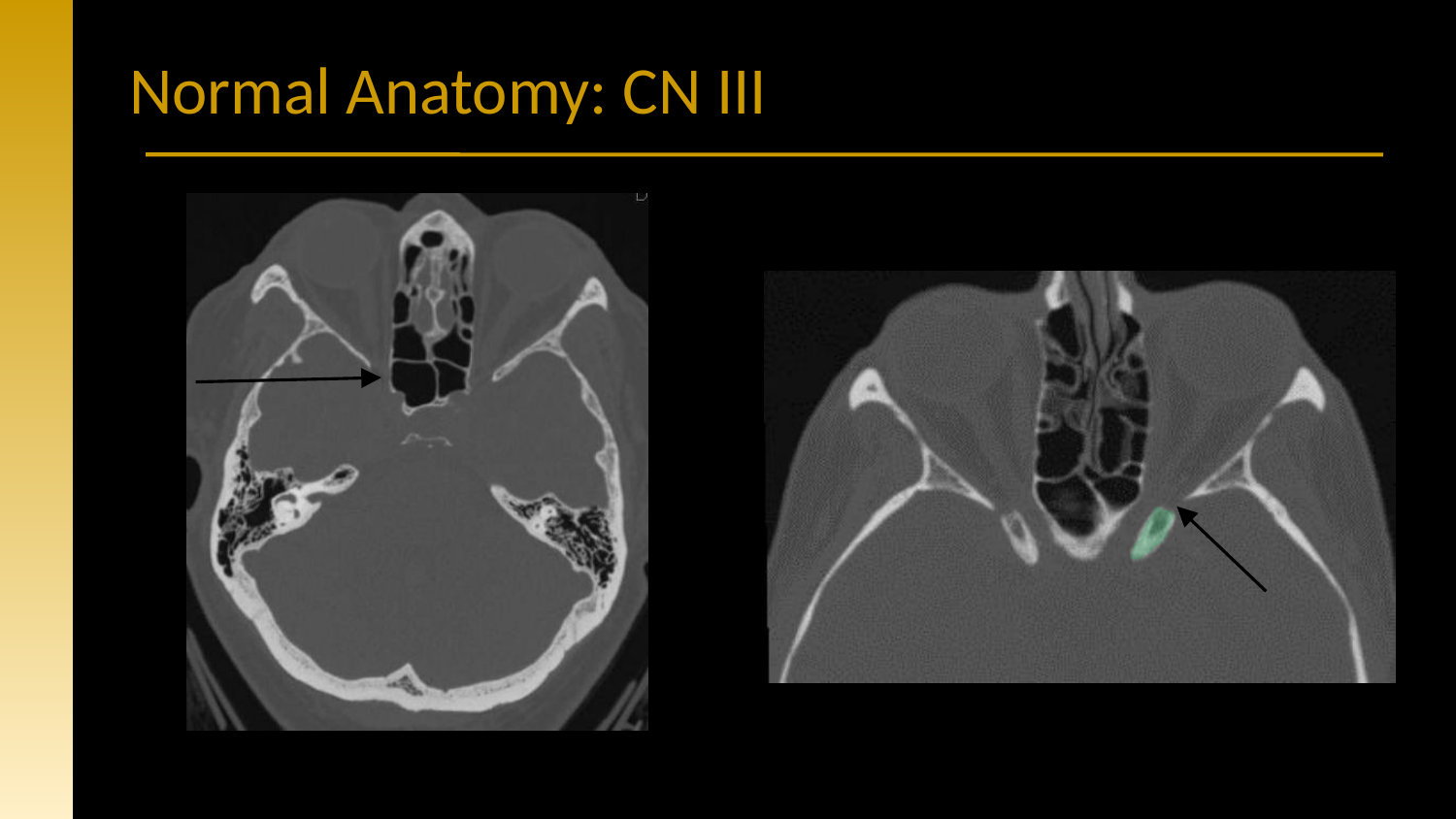

Normal Anatomy: CN III

## Slide 15
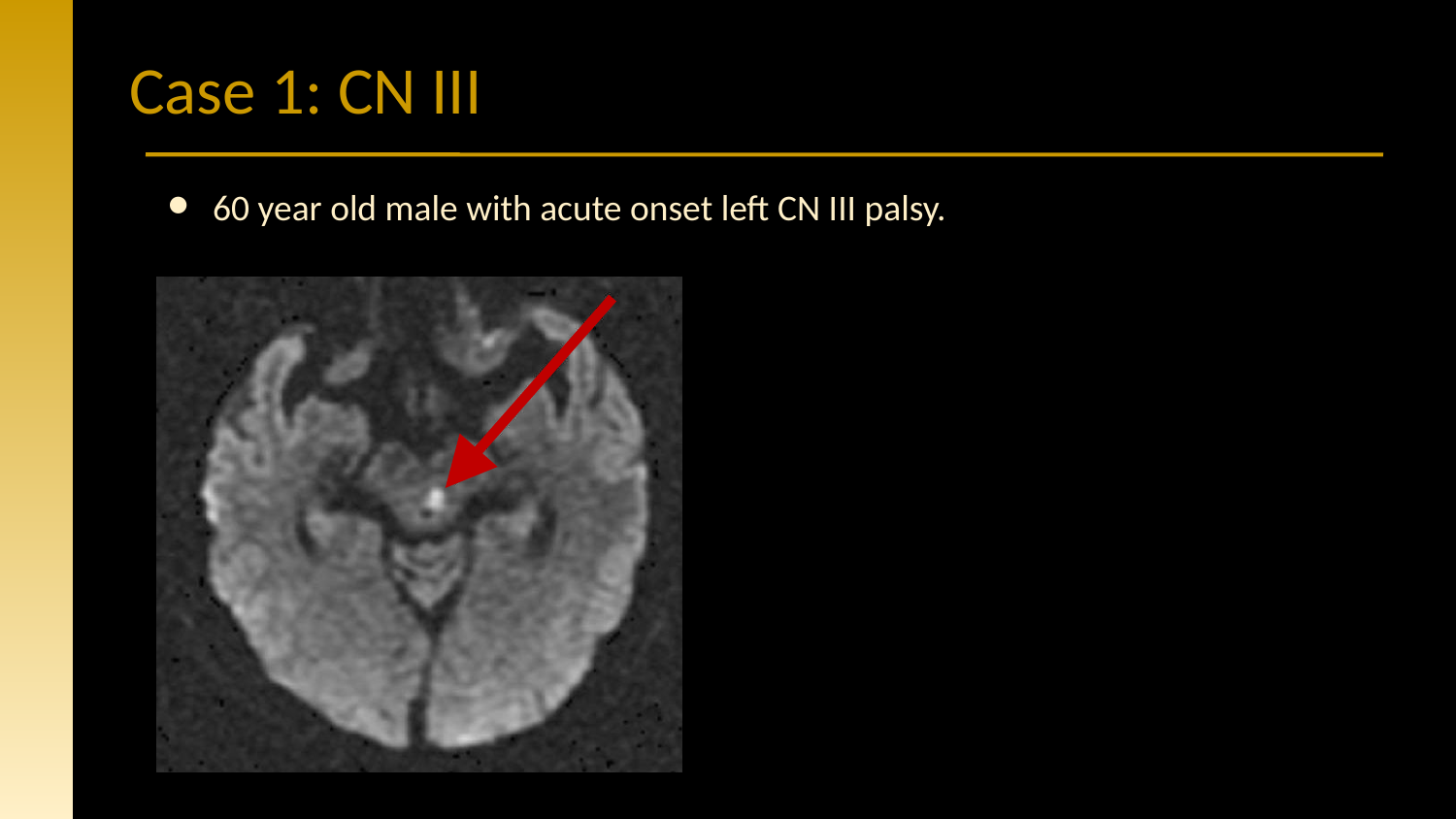

Case 1: CN III
# 60 year old male with acute onset left CN III palsy.

## Slide 16
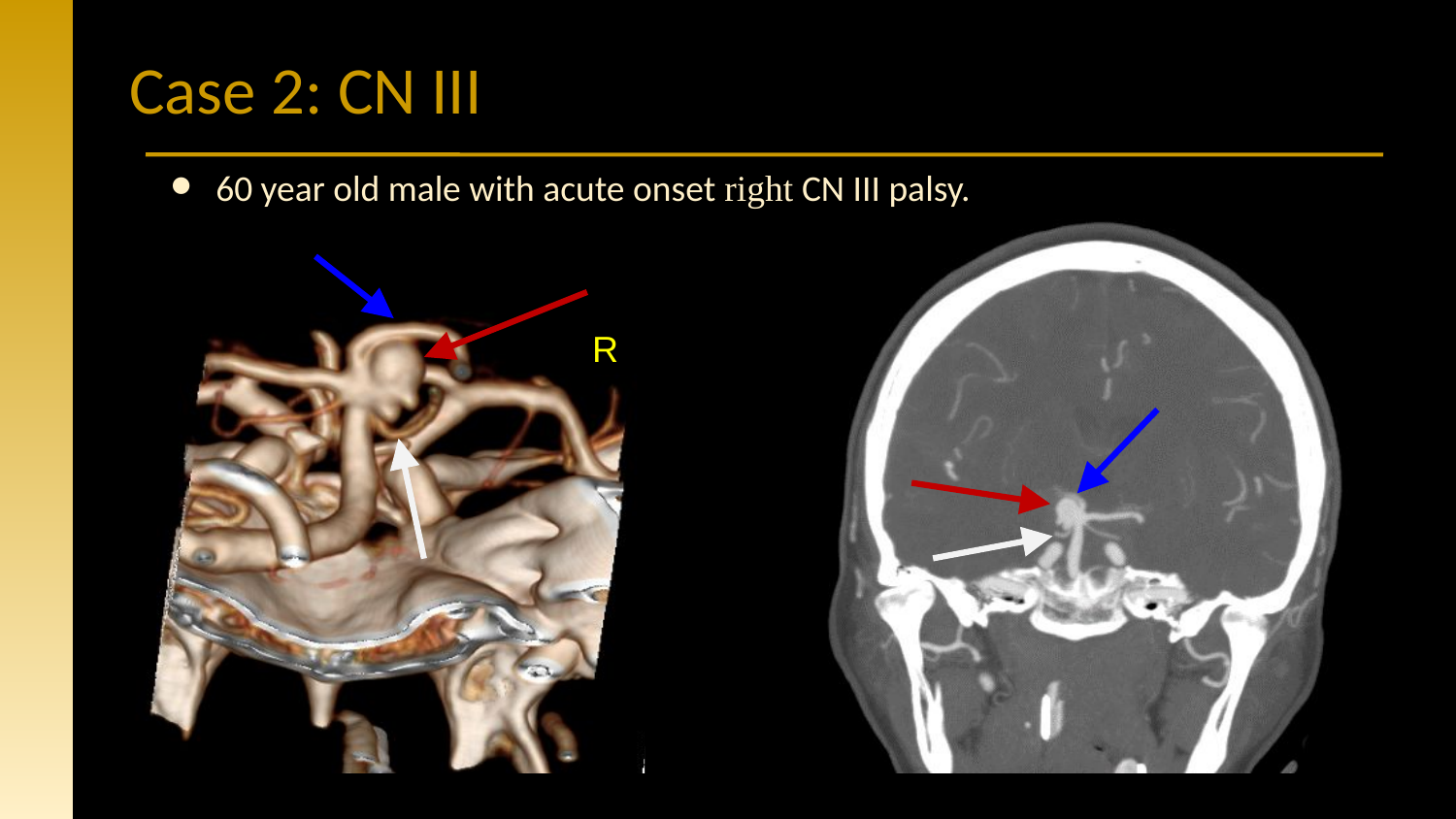

Case 2: CN III
# 60 year old male with acute onset right CN III palsy.
R

## Slide 17
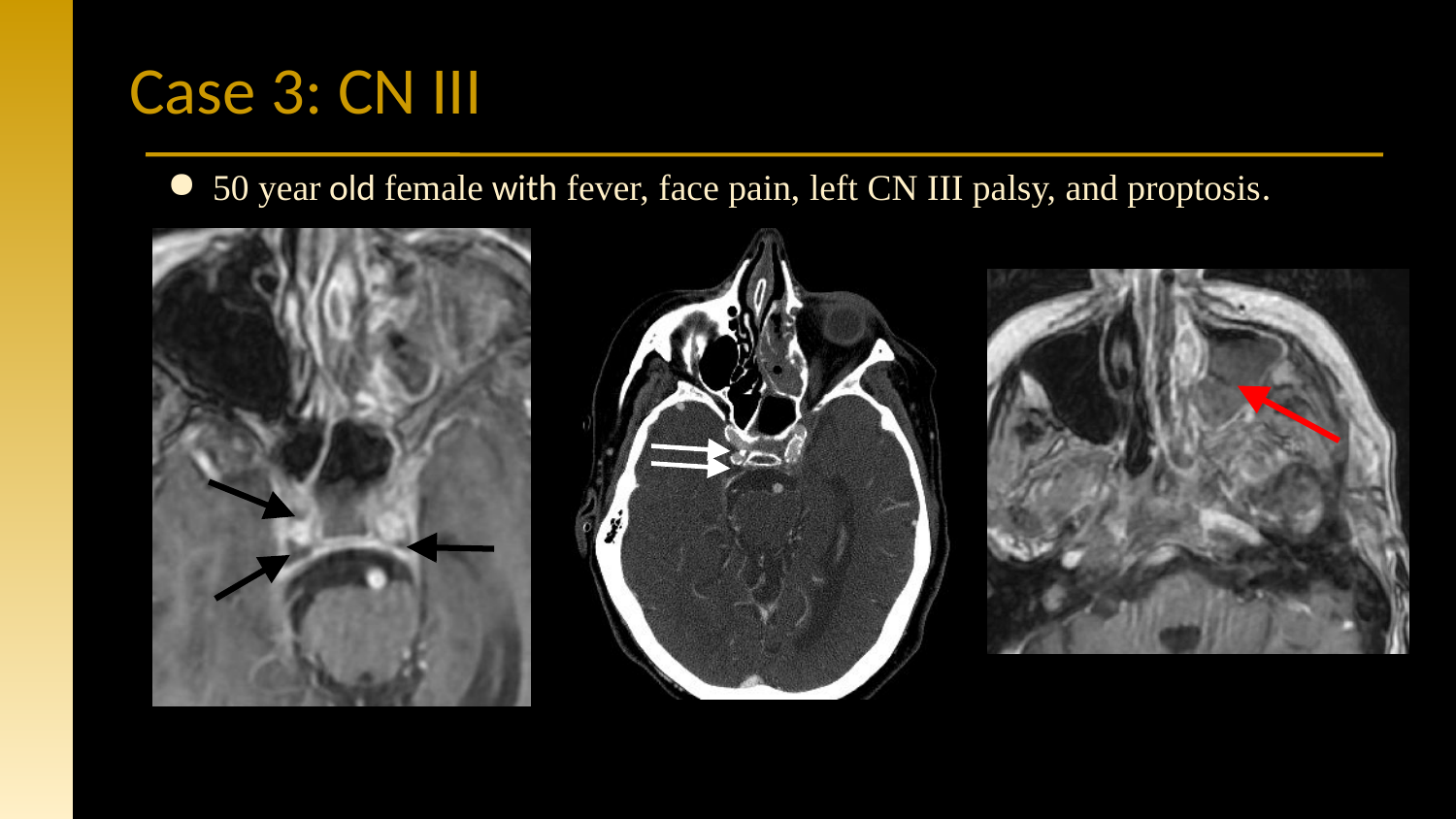

Case 3: CN III
# 50 year old female with fever, face pain, left CN III palsy, and proptosis.

## Slide 18
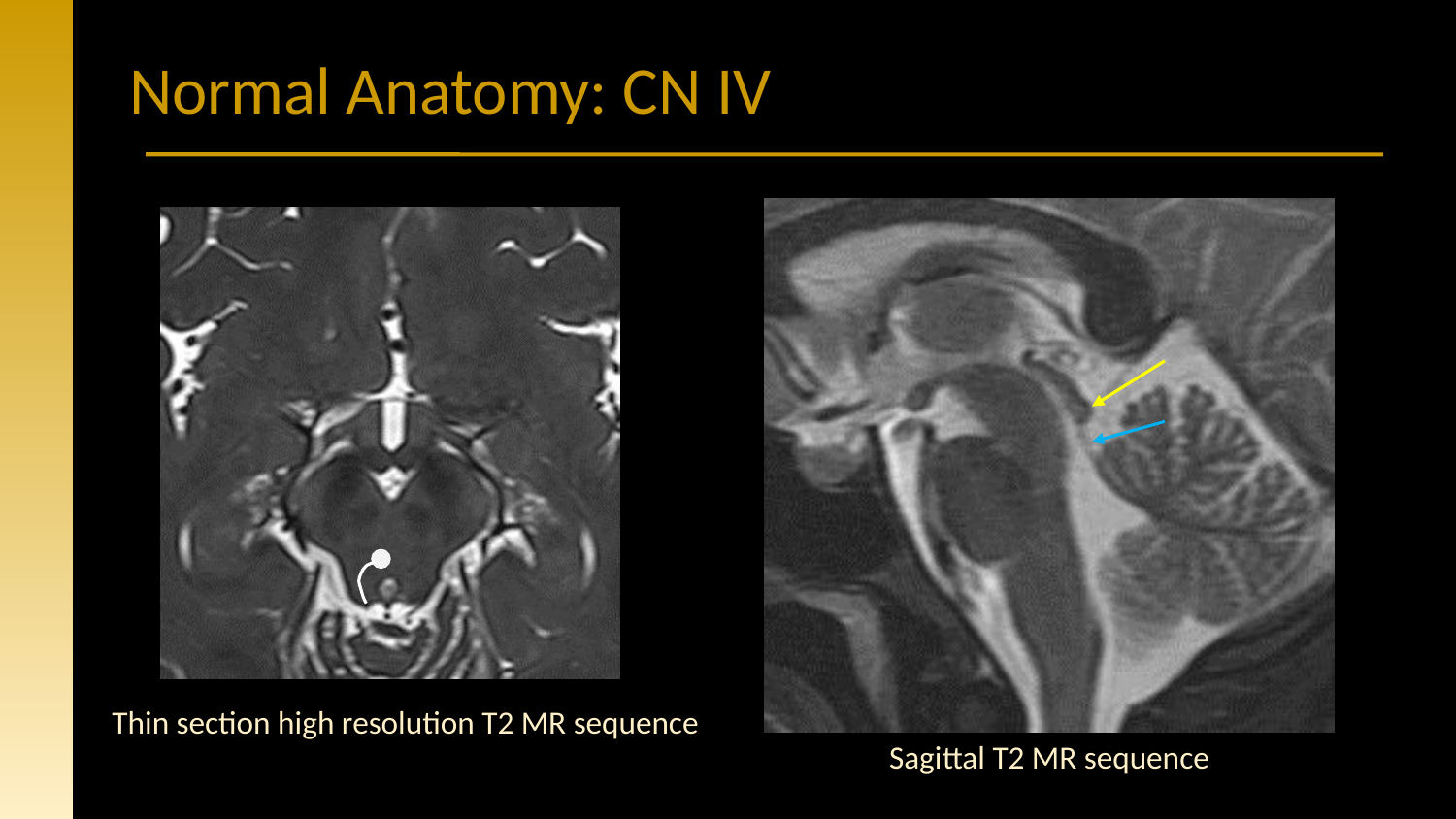

Normal Anatomy: CN IV
Thin section high resolution T2 MR sequence
Sagittal T2 MR sequence

## Slide 19
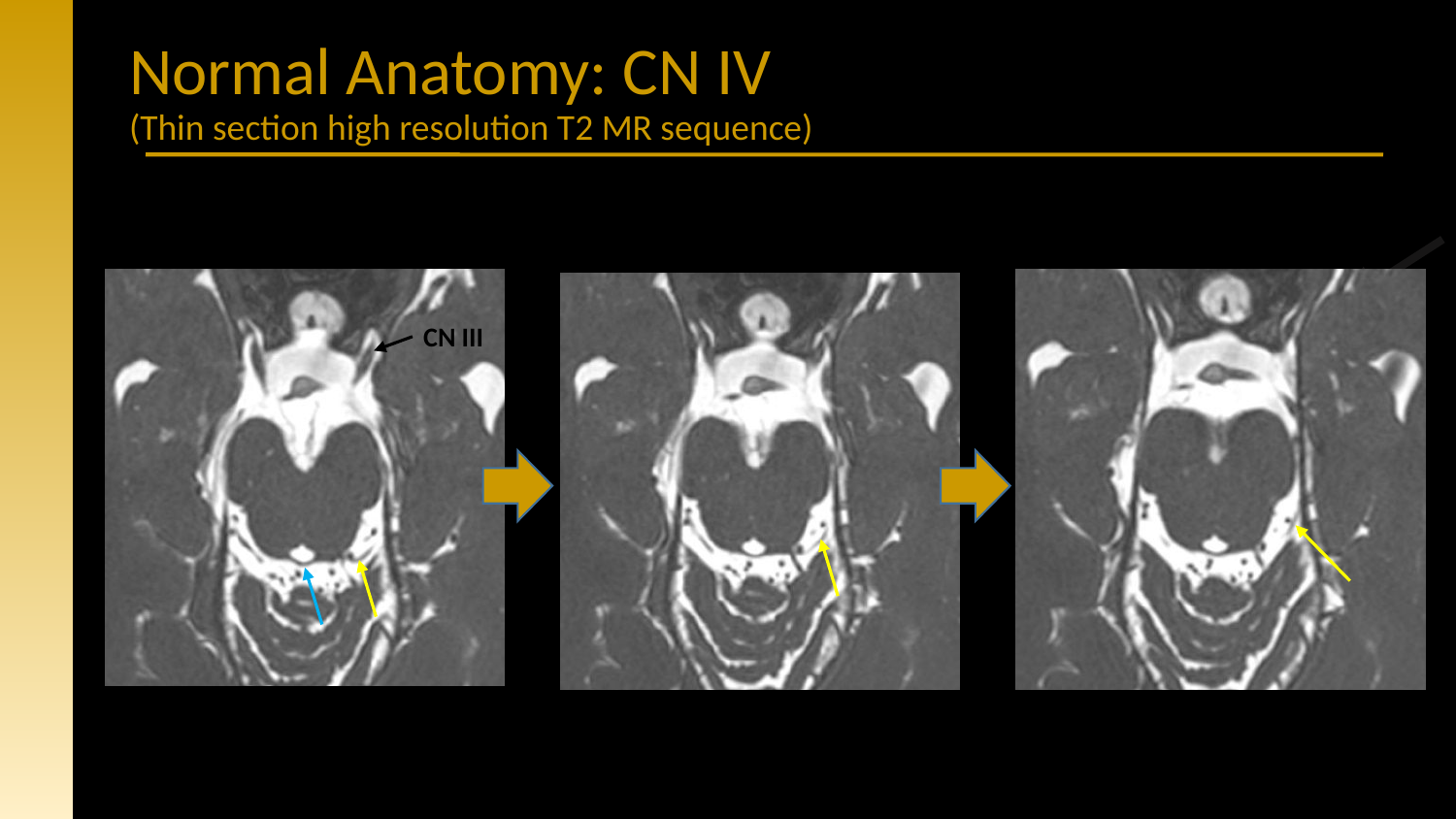

Normal Anatomy: CN IV
(Thin section high resolution T2 MR sequence)
CN III
.

## Slide 20
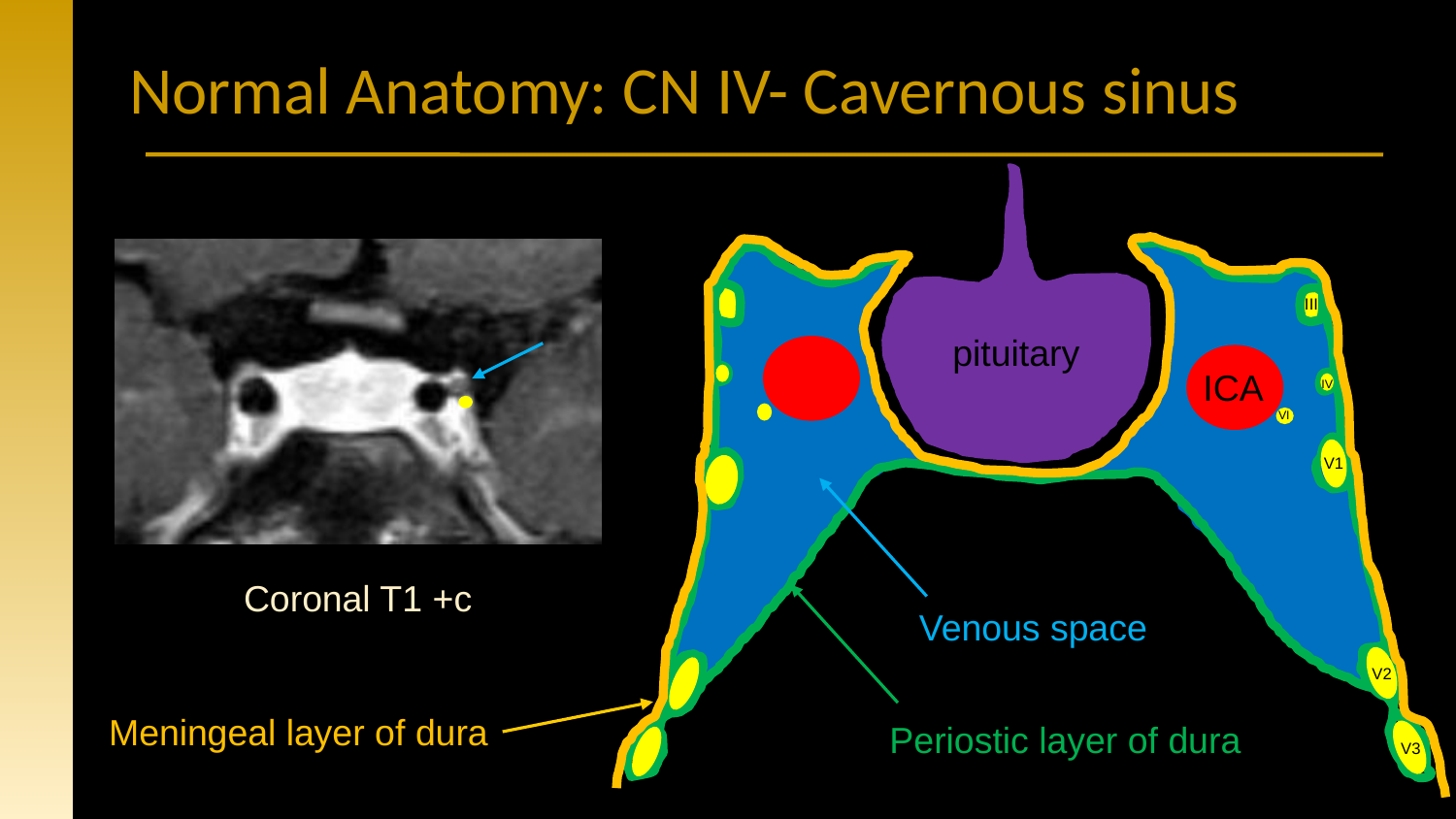

Normal Anatomy: CN IV- Cavernous sinus
Coronal T1 +c
III
pituitary
ICA
IV
VI
V1
Venous space
V2
Meningeal layer of dura
Periostic layer of dura
V3

## Slide 21
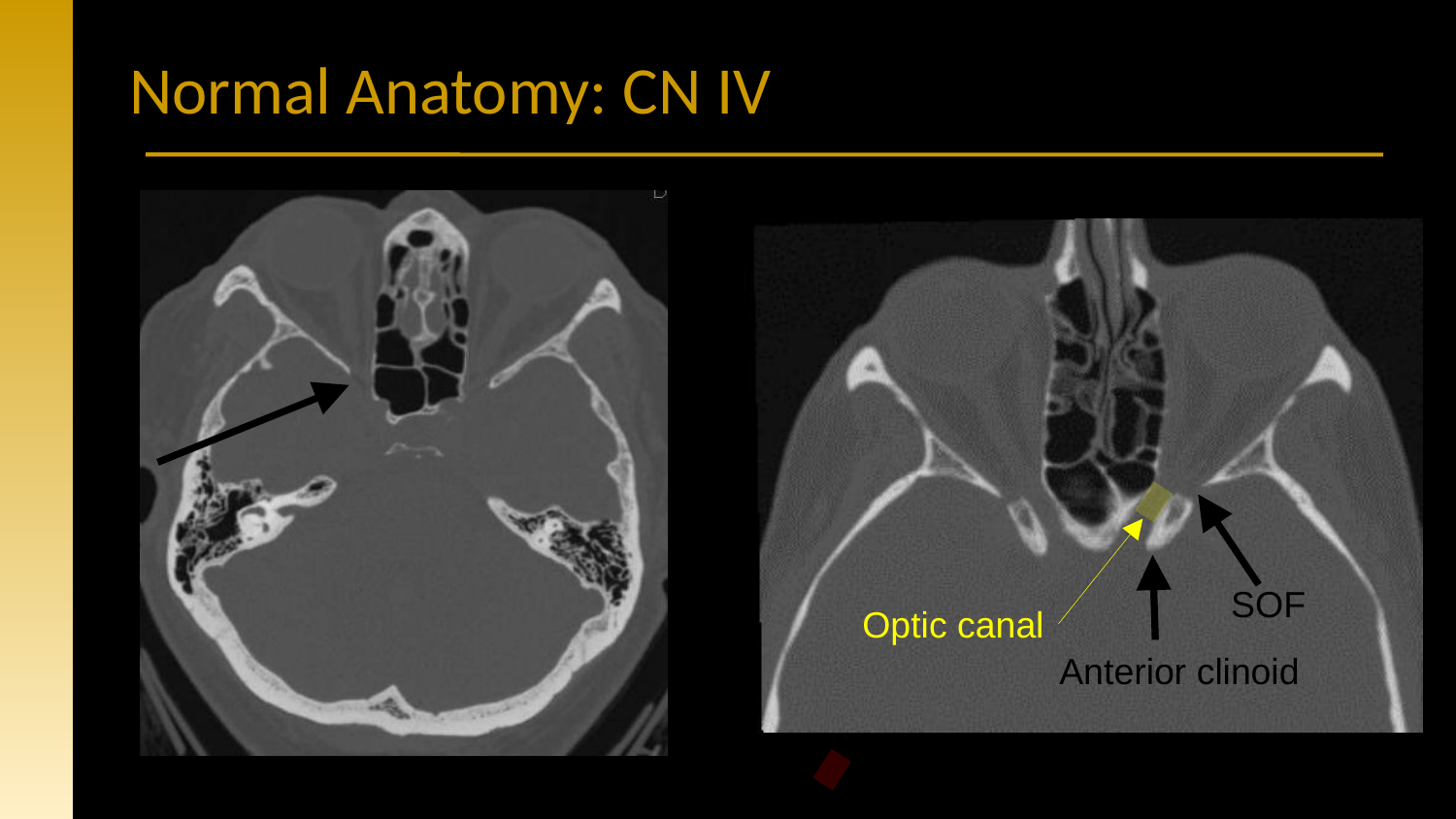

Normal Anatomy: CN IV
SOF
Optic canal
Anterior clinoid

## Slide 22
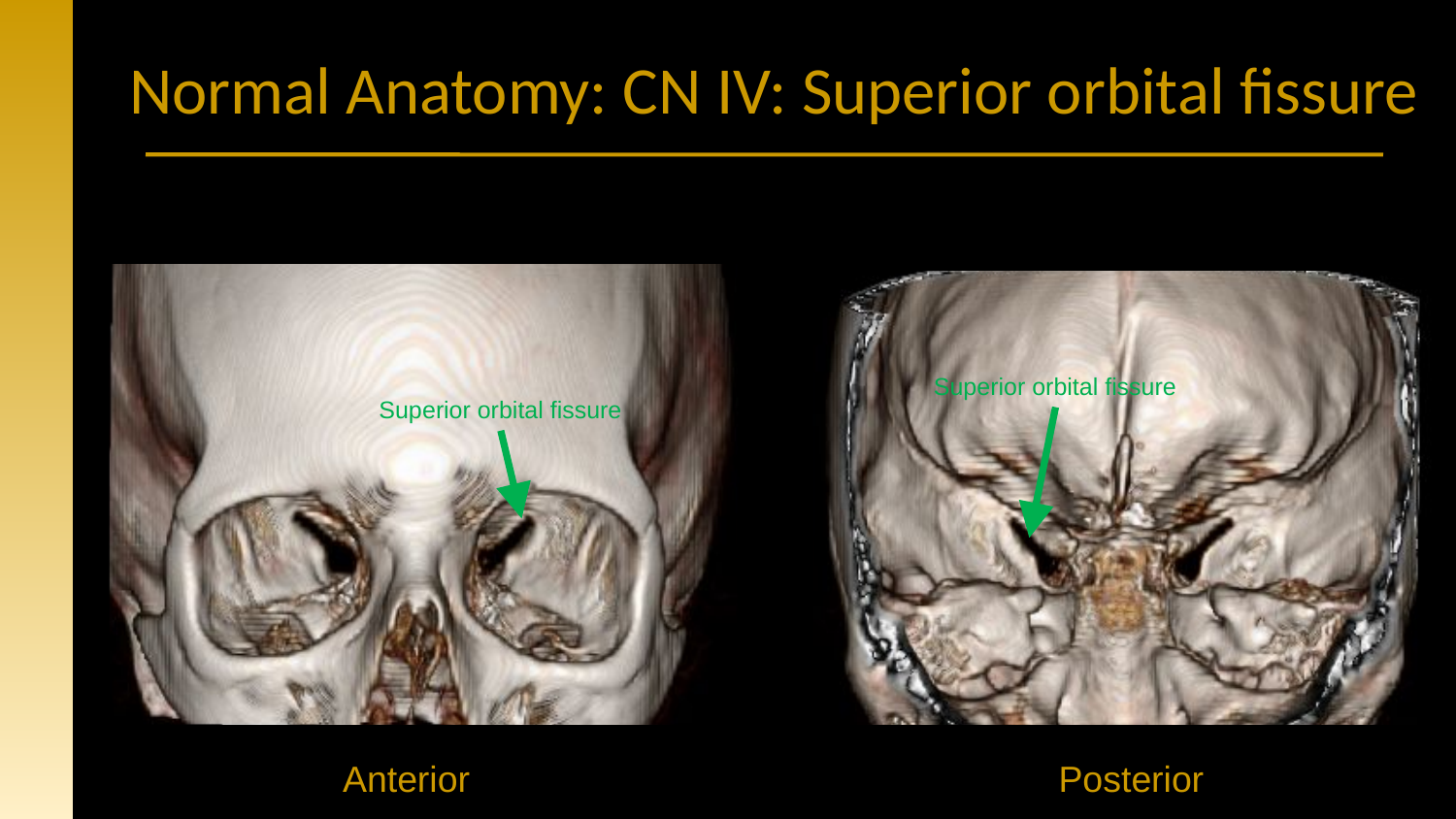

Normal Anatomy: CN IV: Superior orbital fissure
Superior orbital fissure
Superior orbital fissure
Anterior
Posterior

## Slide 23
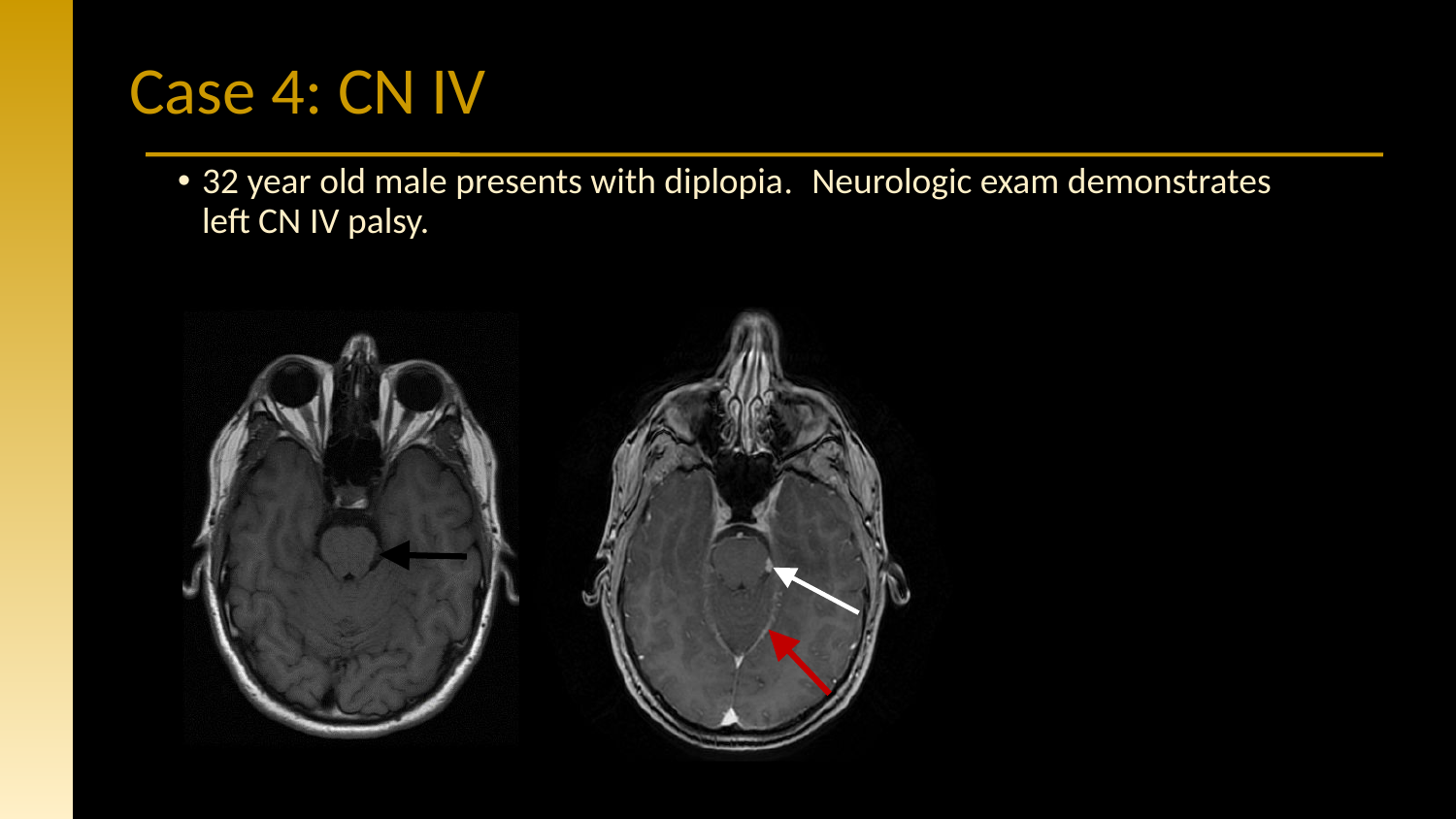

Case 4: CN IV
32 year old male presents with diplopia. Neurologic exam demonstrates left CN IV palsy.

## Slide 24
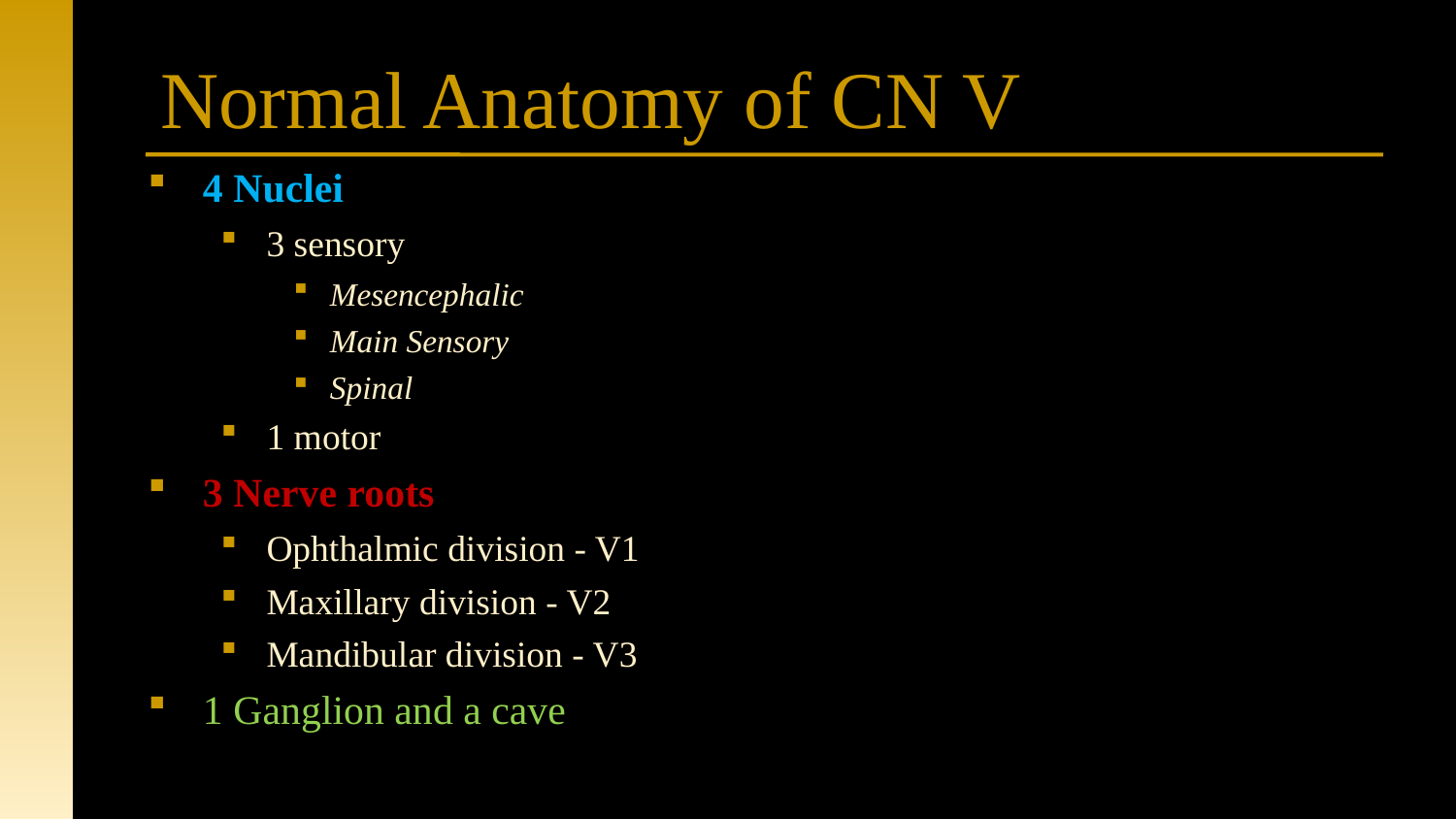

# Normal Anatomy of CN V
4 Nuclei
3 sensory
Mesencephalic
Main Sensory
Spinal
1 motor
3 Nerve roots
Ophthalmic division - V1
Maxillary division - V2
Mandibular division - V3
1 Ganglion and a cave

## Slide 25
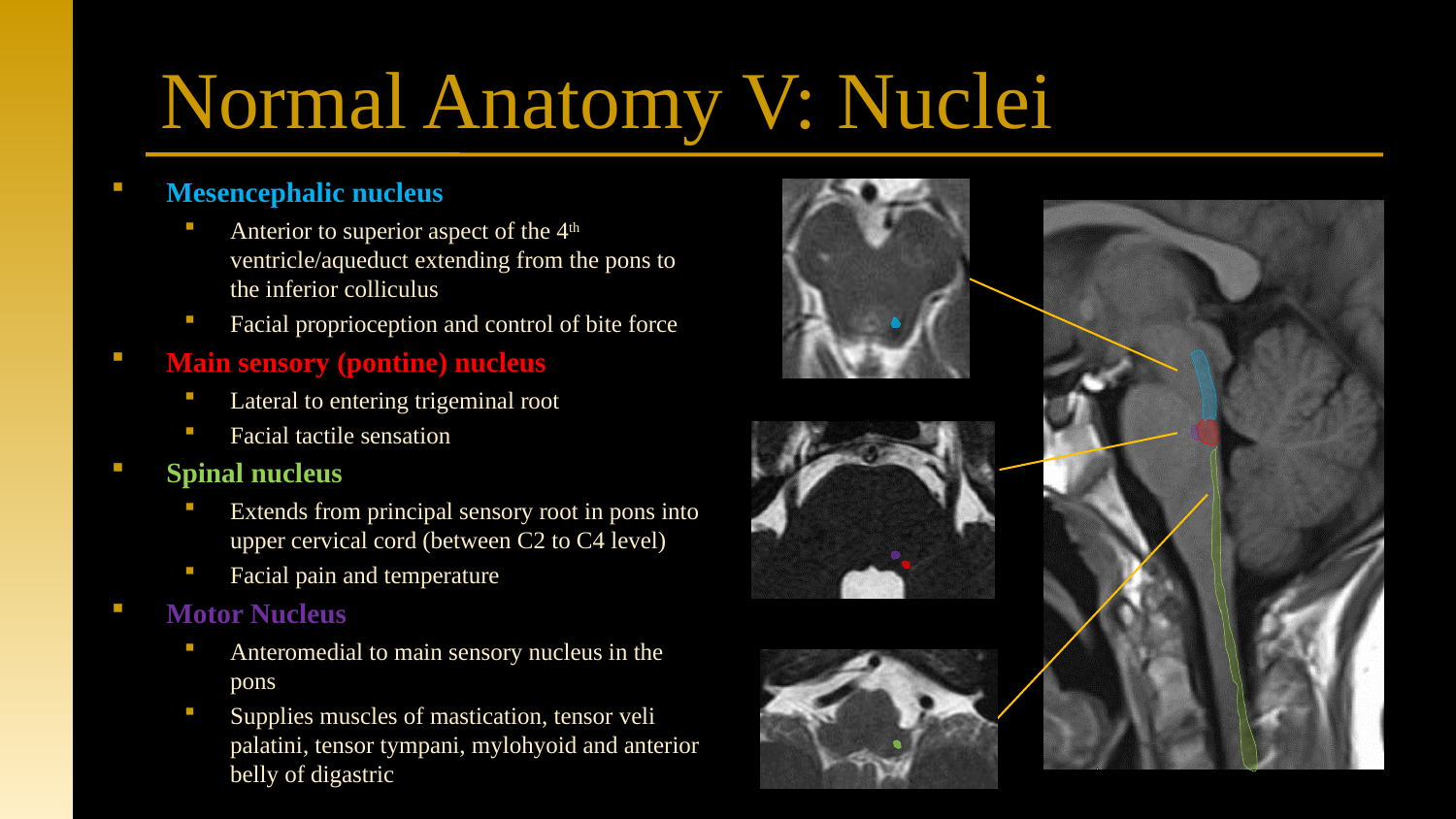

# Normal Anatomy V: Nuclei
Mesencephalic nucleus
Anterior to superior aspect of the 4th ventricle/aqueduct extending from the pons to the inferior colliculus
Facial proprioception and control of bite force
Main sensory (pontine) nucleus
Lateral to entering trigeminal root
Facial tactile sensation
Spinal nucleus
Extends from principal sensory root in pons into upper cervical cord (between C2 to C4 level)
Facial pain and temperature
Motor Nucleus
Anteromedial to main sensory nucleus in the pons
Supplies muscles of mastication, tensor veli palatini, tensor tympani, mylohyoid and anterior belly of digastric

## Slide 26
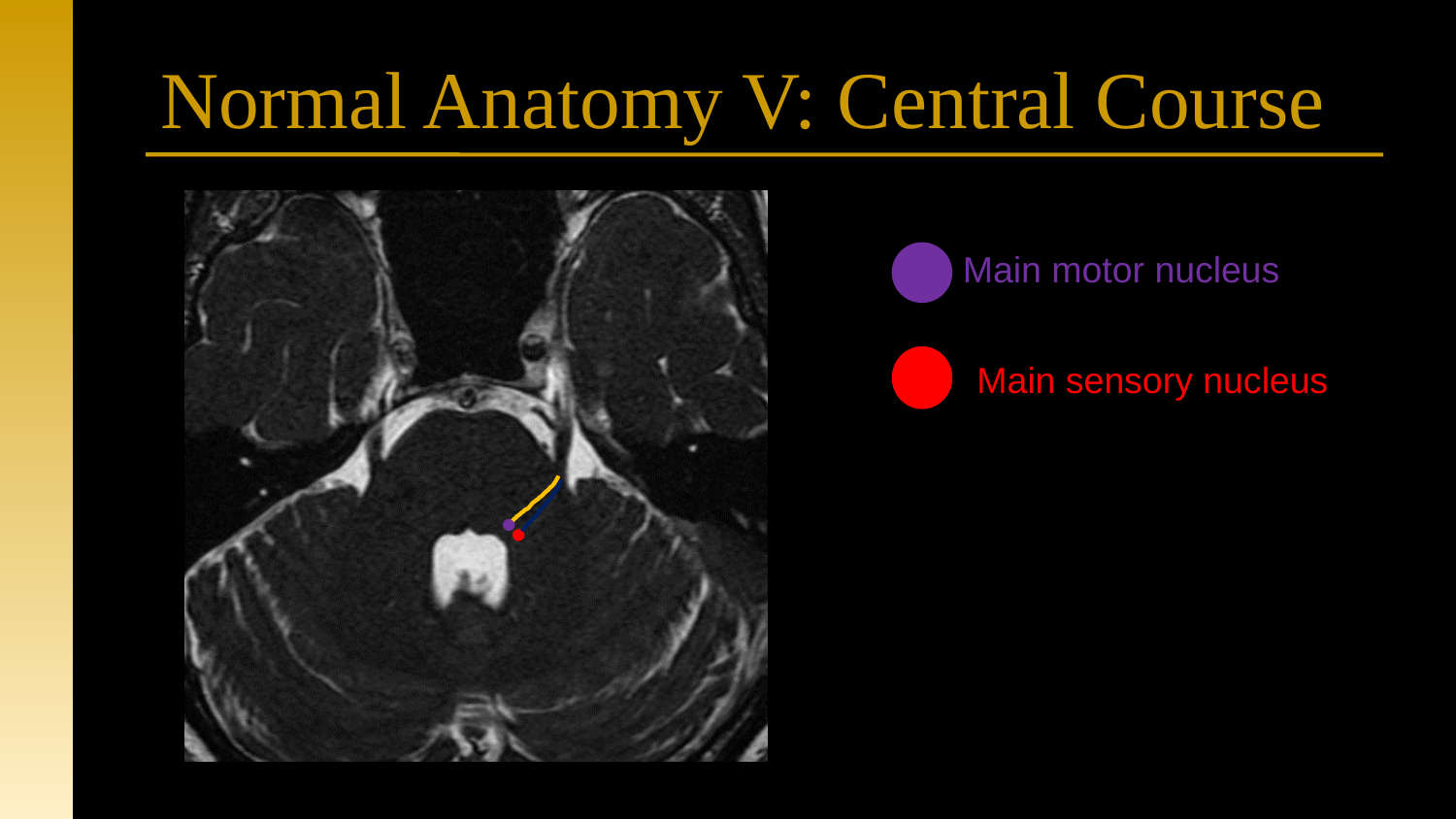

# Normal Anatomy V: Central Course
Main motor nucleus
Main sensory nucleus

## Slide 27
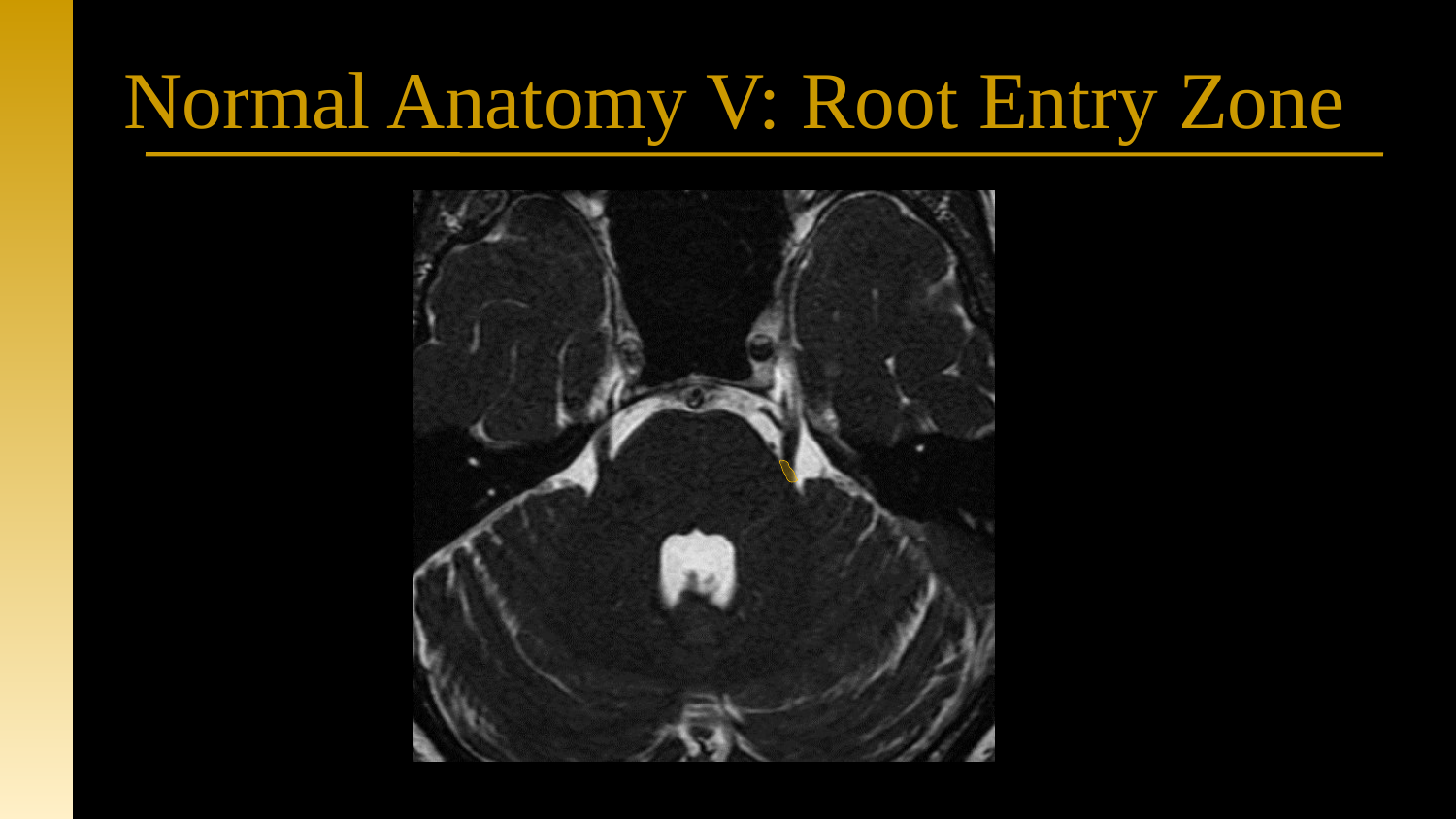

# Normal Anatomy V: Root Entry Zone

## Slide 28
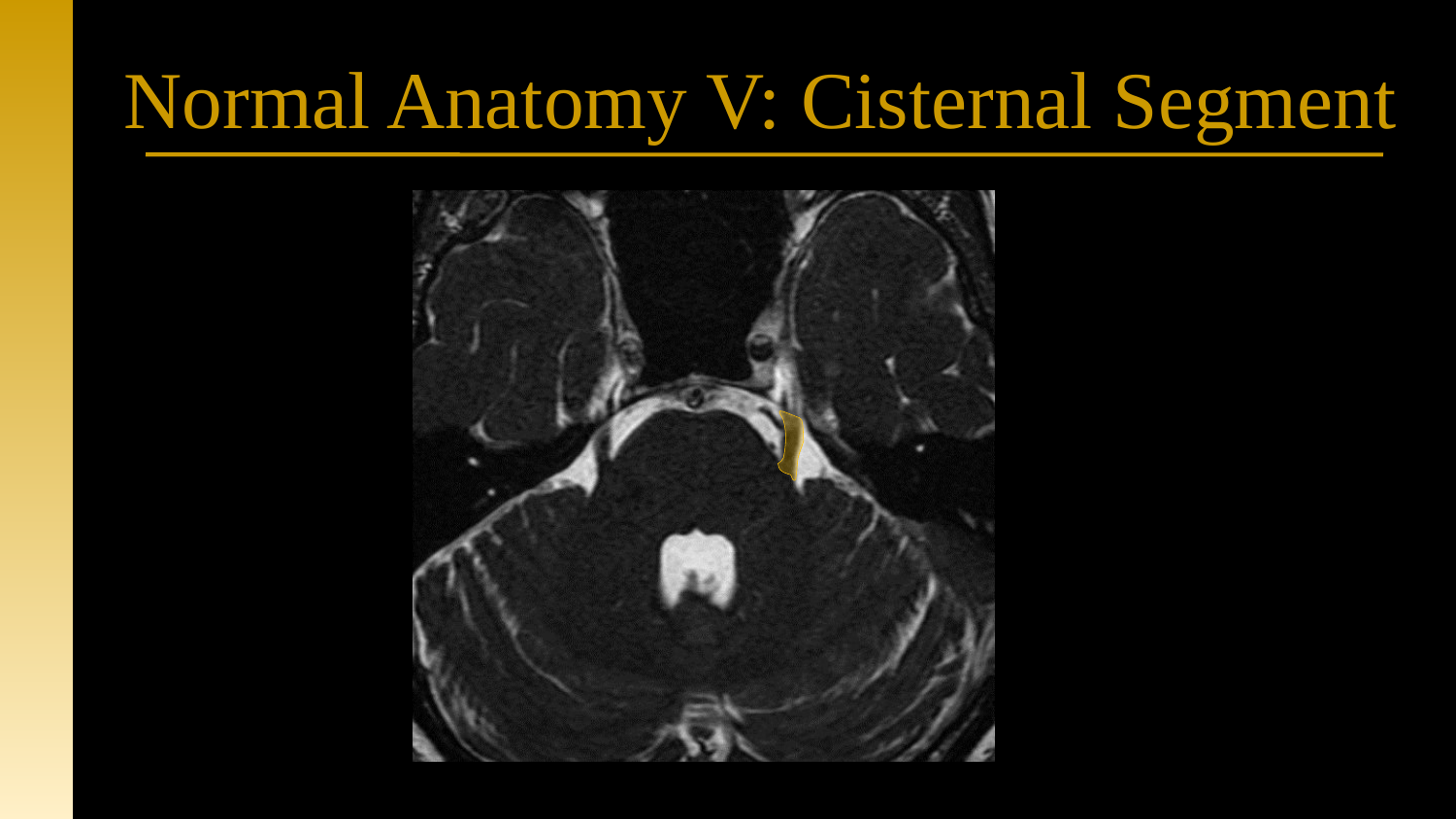

# Normal Anatomy V: Cisternal Segment

## Slide 29
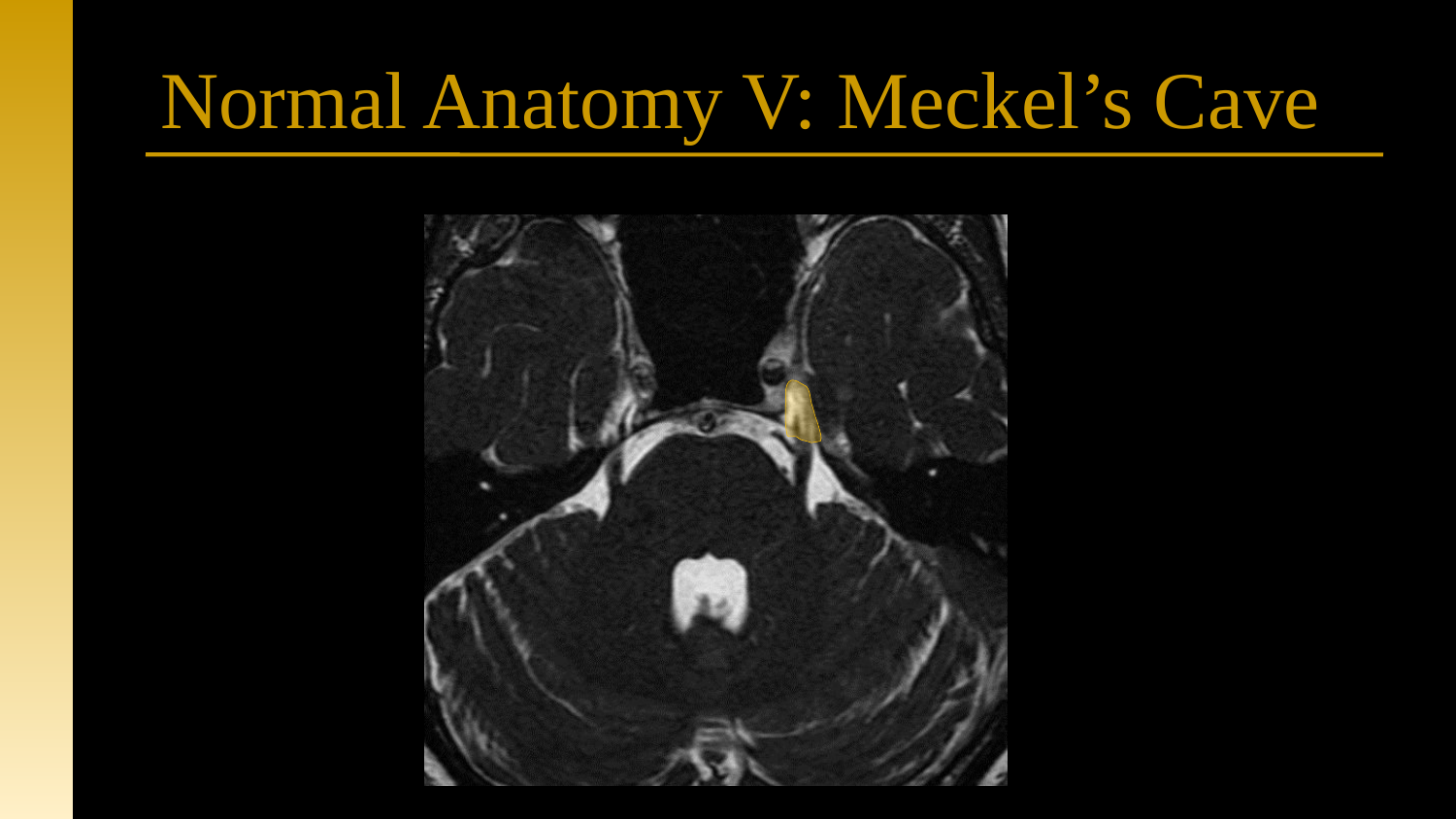

# Normal Anatomy V: Meckel’s Cave

## Slide 30
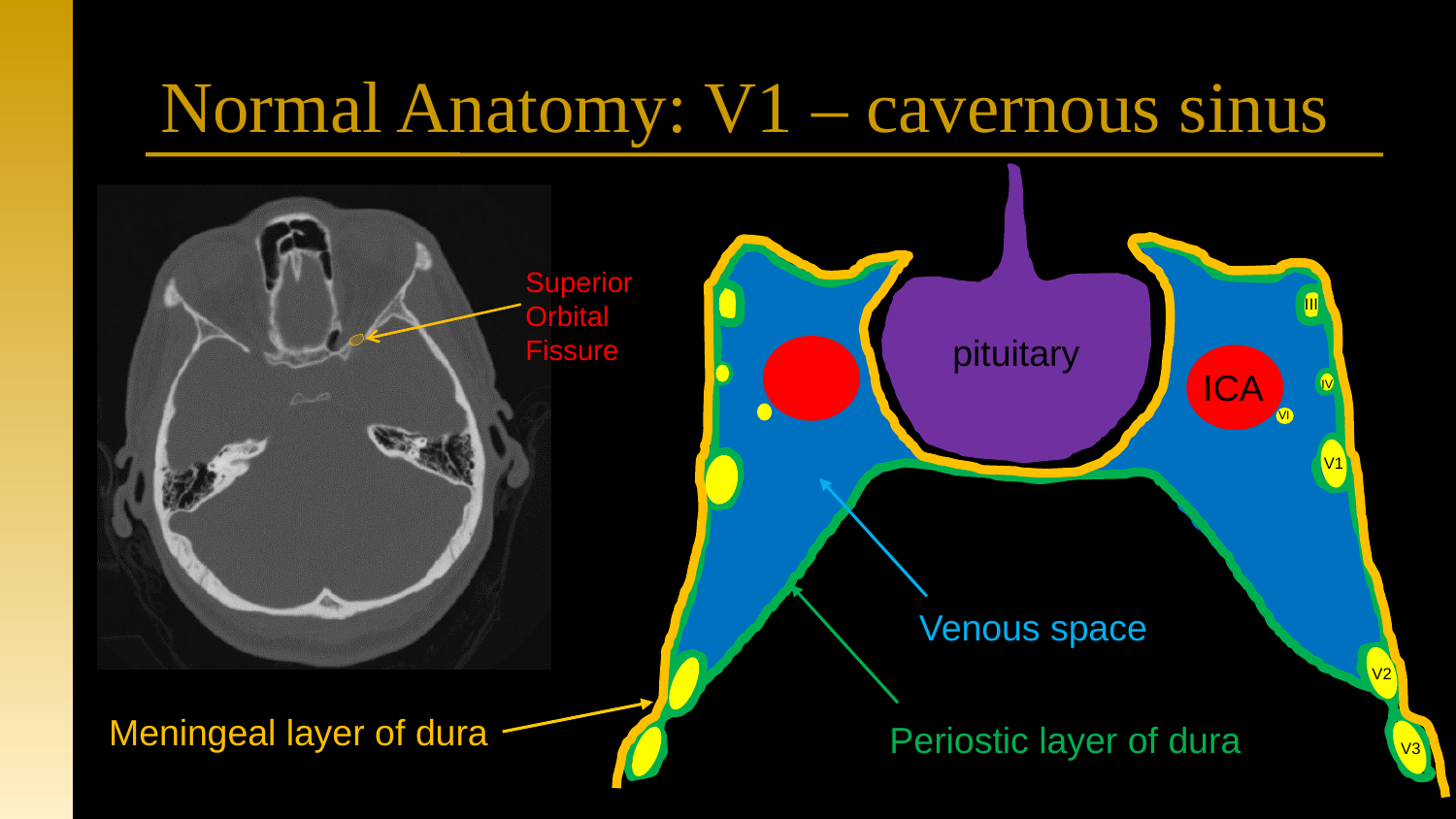

# Normal Anatomy: V1 – cavernous sinus
Superior
Orbital
Fissure
III
pituitary
ICA
IV
VI
V1
Venous space
V2
Meningeal layer of dura
Periostic layer of dura
V3

## Slide 31
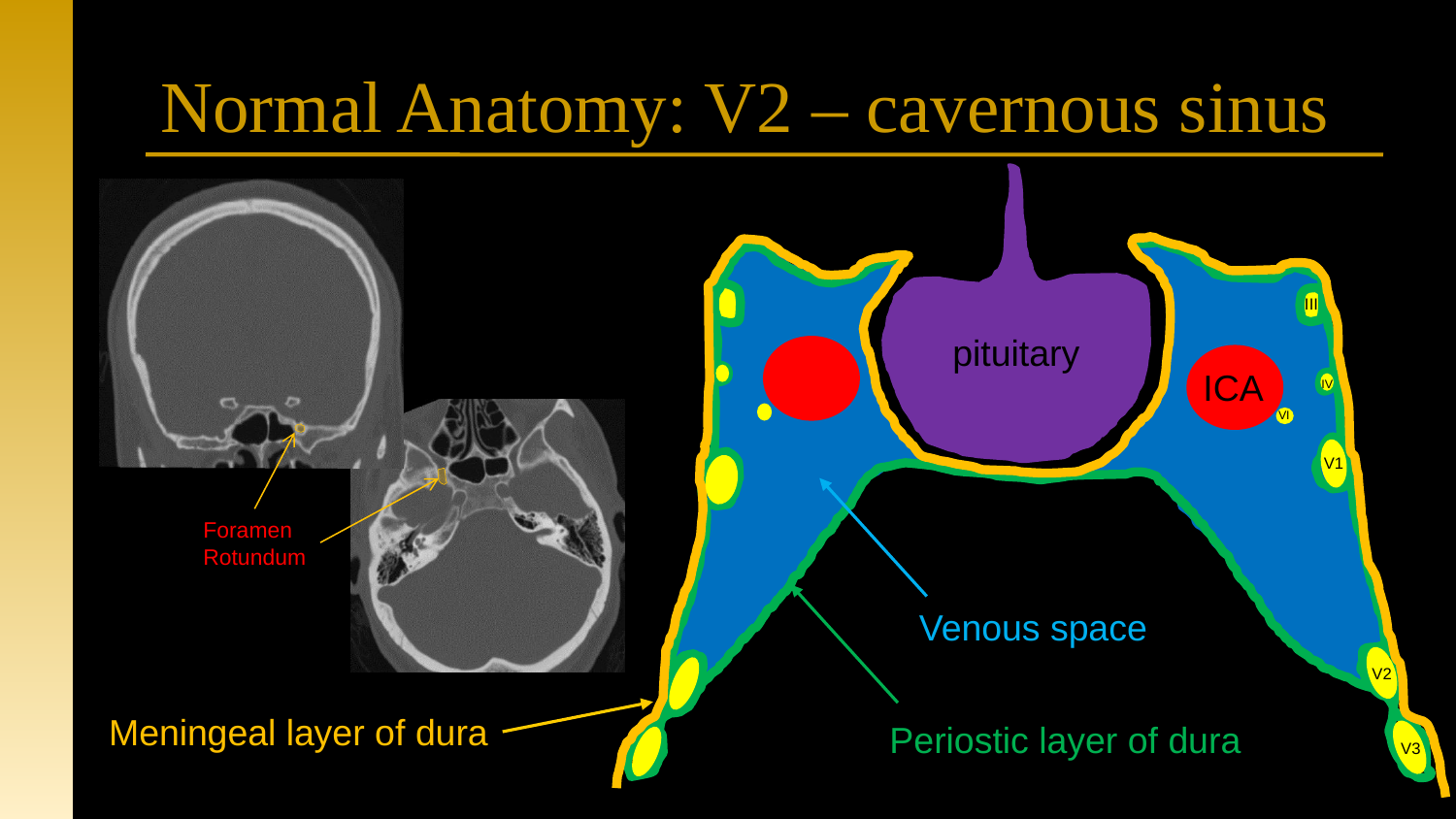

# Normal Anatomy: V2 – cavernous sinus
Foramen
Rotundum
III
pituitary
ICA
IV
VI
V1
Venous space
V2
Meningeal layer of dura
Periostic layer of dura
V3

## Slide 32
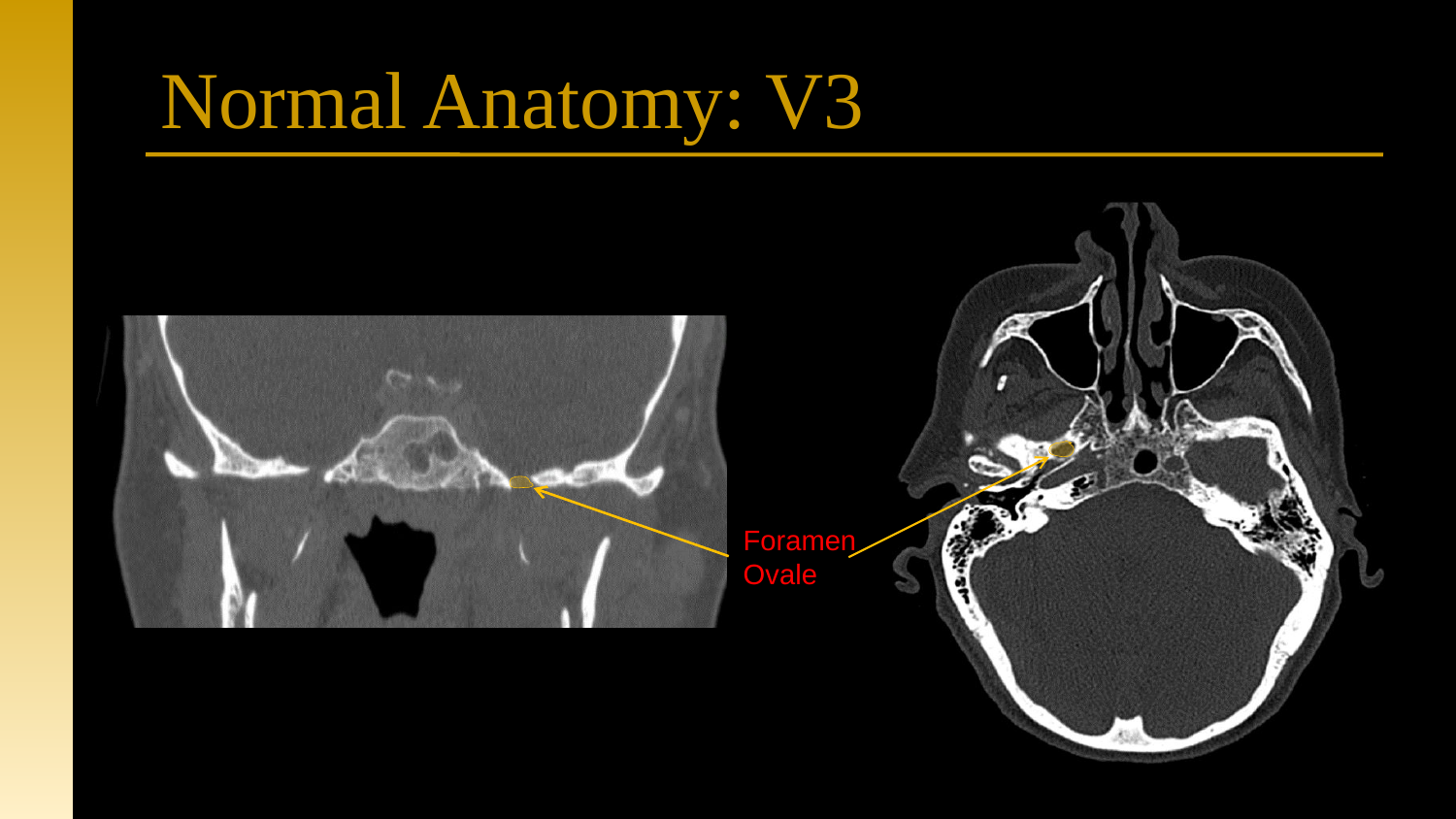

# Normal Anatomy: V3
Foramen
Ovale

## Slide 33
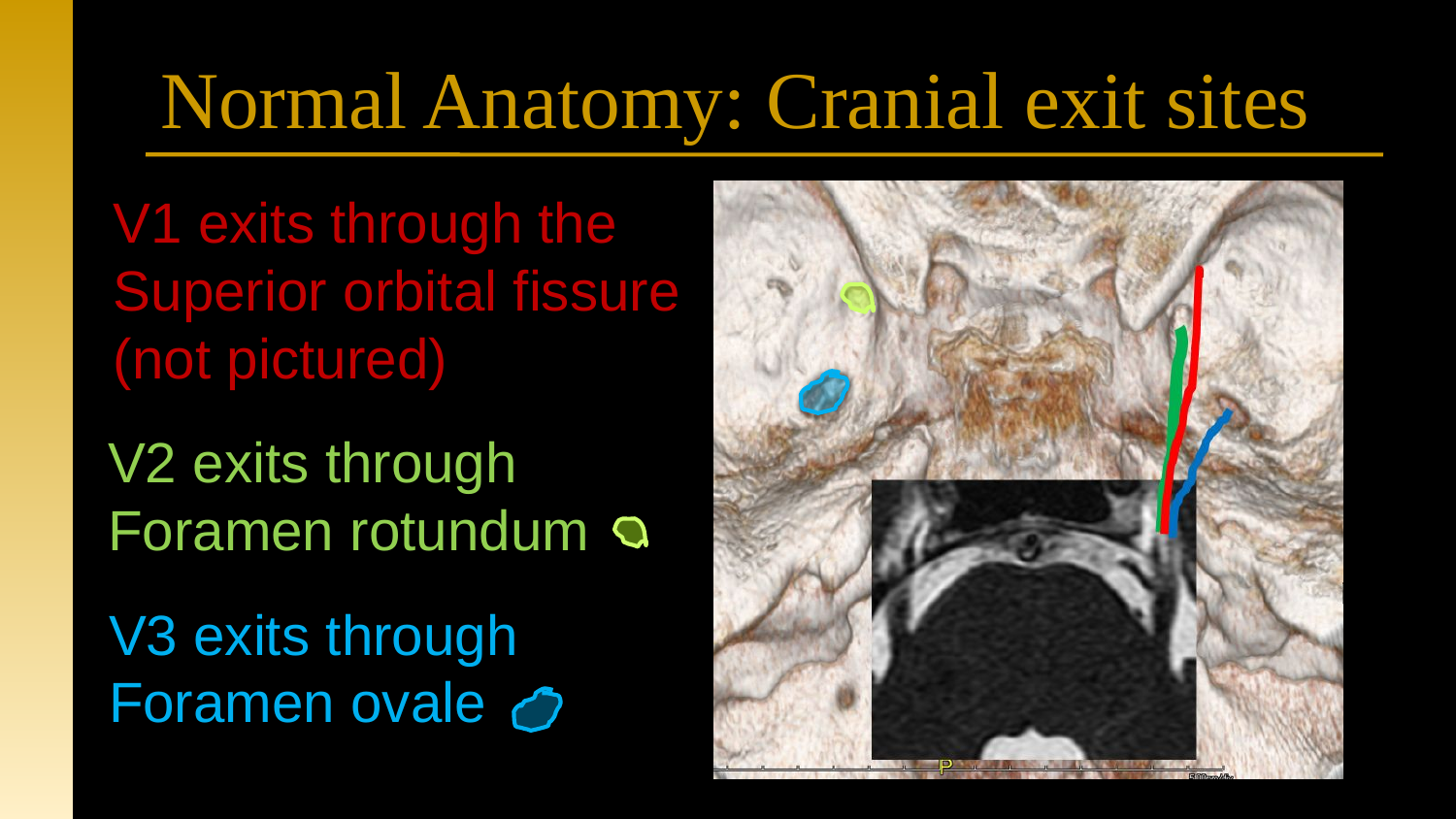

# Normal Anatomy: Cranial exit sites
V1 exits through the
Superior orbital fissure
(not pictured)
V2 exits through
Foramen rotundum
V3 exits through
Foramen ovale

## Slide 34
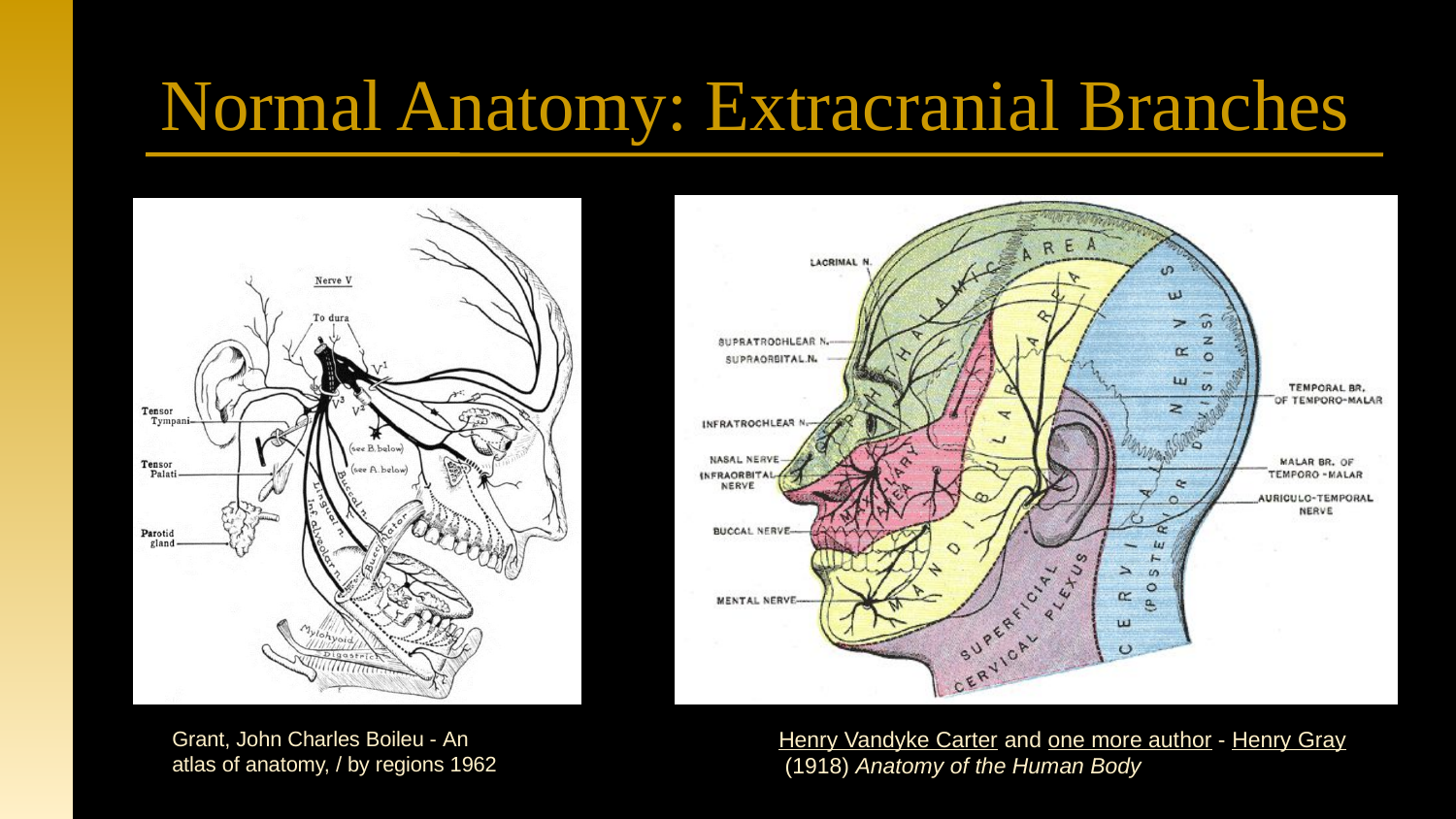

# Normal Anatomy: Extracranial Branches
Grant, John Charles Boileu - An atlas of anatomy, / by regions 1962
Henry Vandyke Carter and one more author - Henry Gray (1918) Anatomy of the Human Body

## Slide 35
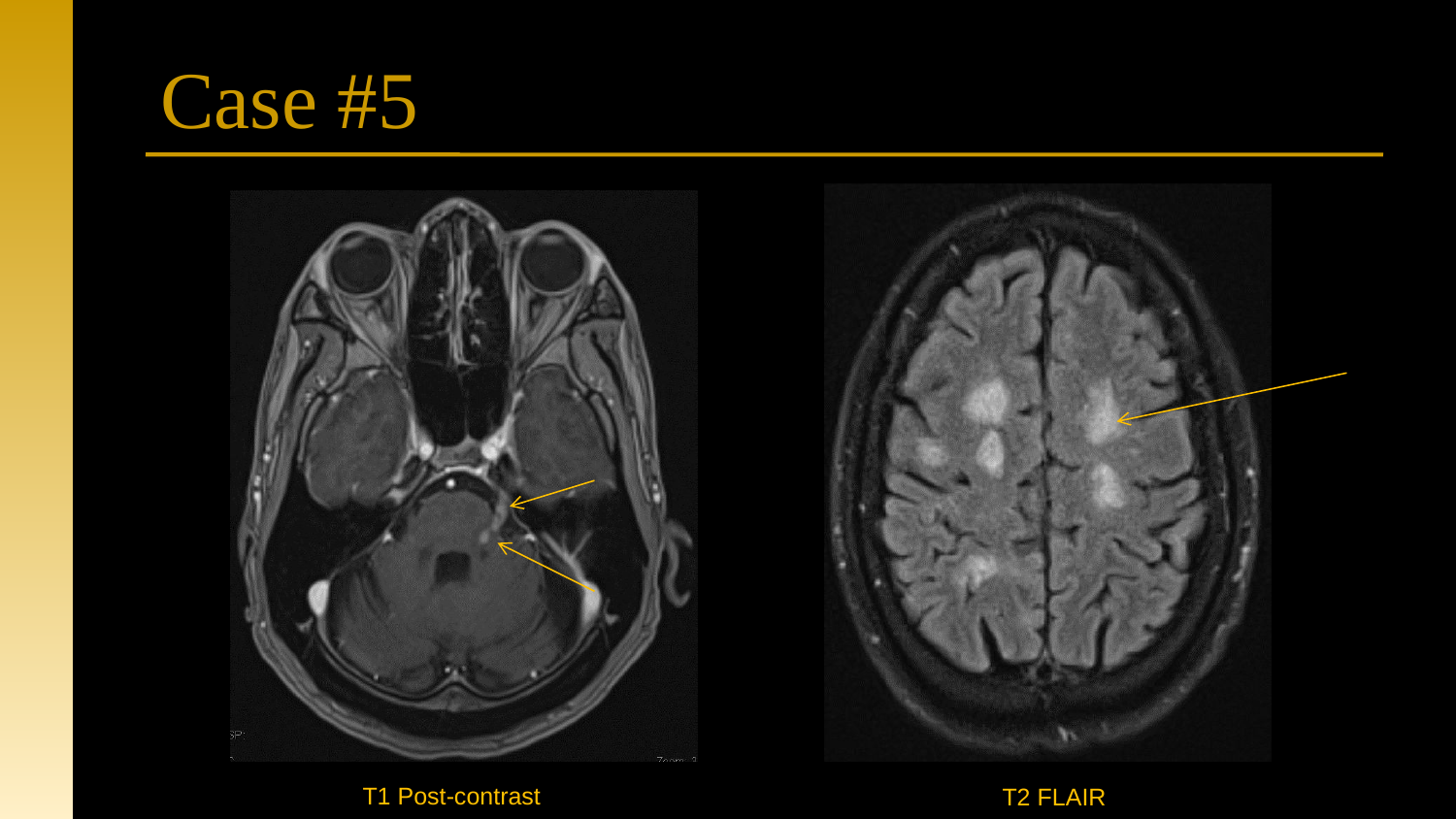

# Case #5
T1 Post-contrast
T2 FLAIR

## Slide 36
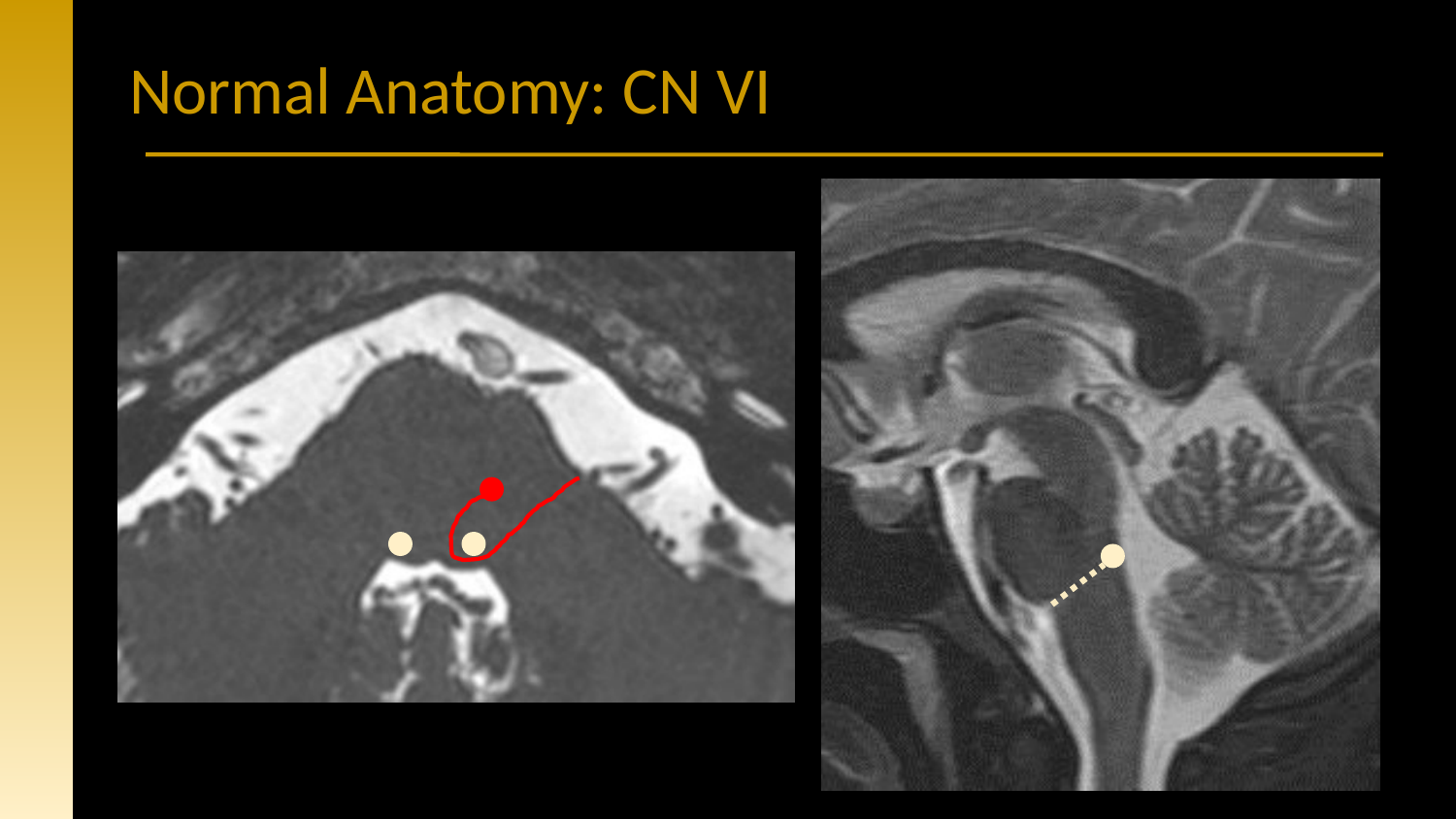

Normal Anatomy: CN VI

## Slide 37
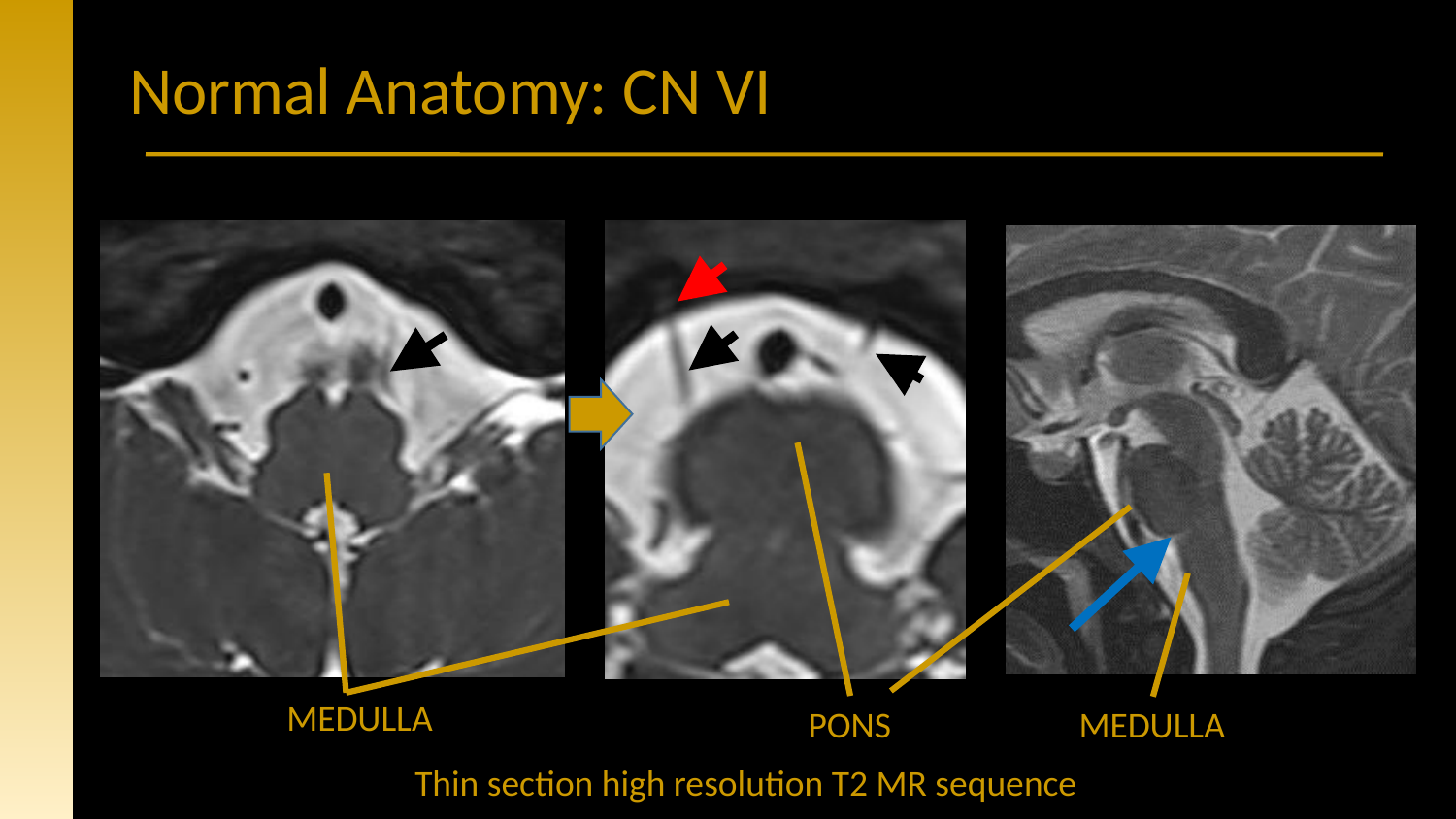

Normal Anatomy: CN VI
MEDULLA
PONS
MEDULLA
Thin section high resolution T2 MR sequence

## Slide 38
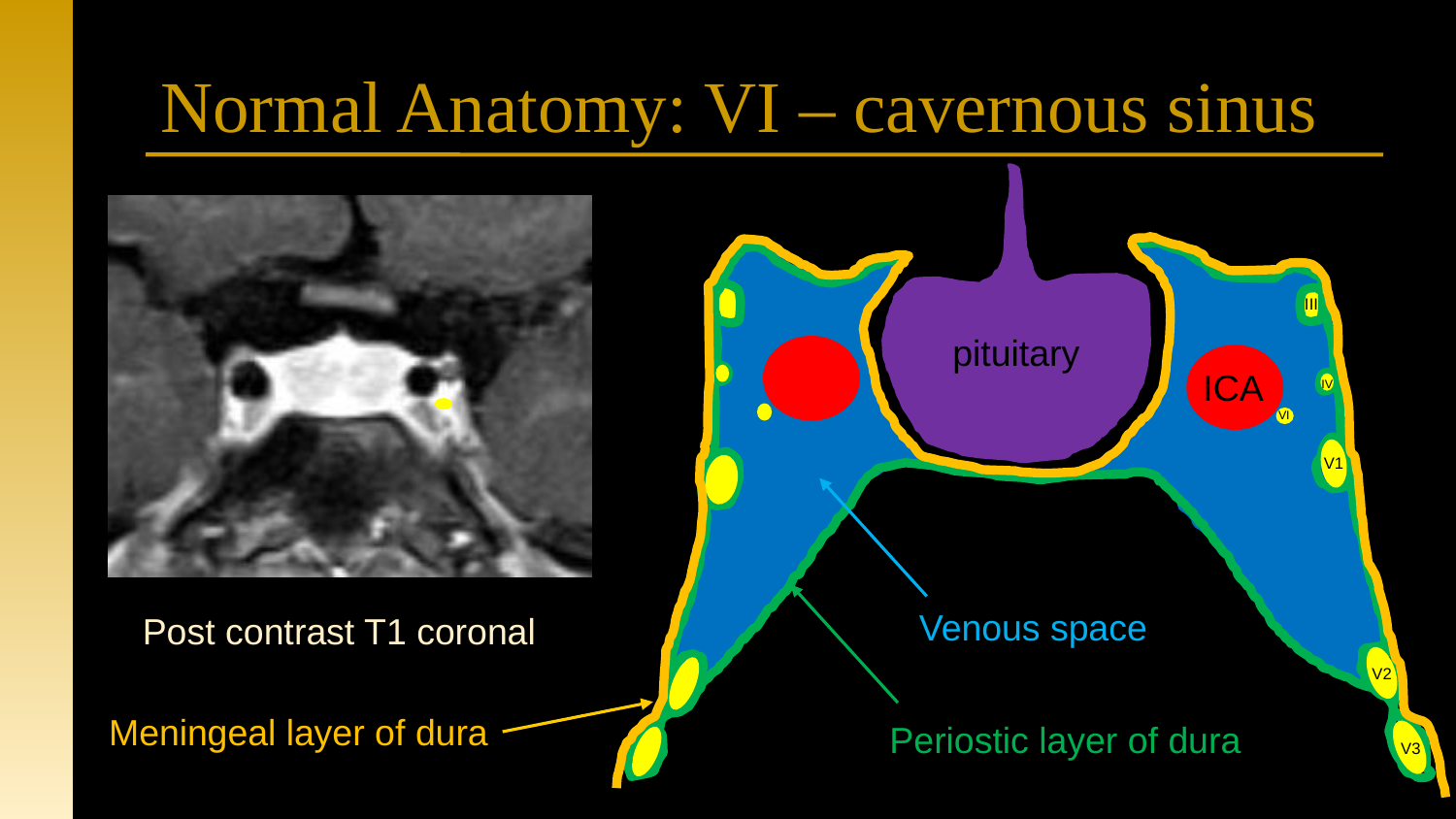

# Normal Anatomy: VI – cavernous sinus
Post contrast T1 coronal
III
pituitary
ICA
IV
VI
V1
Venous space
V2
Meningeal layer of dura
Periostic layer of dura
V3

## Slide 39
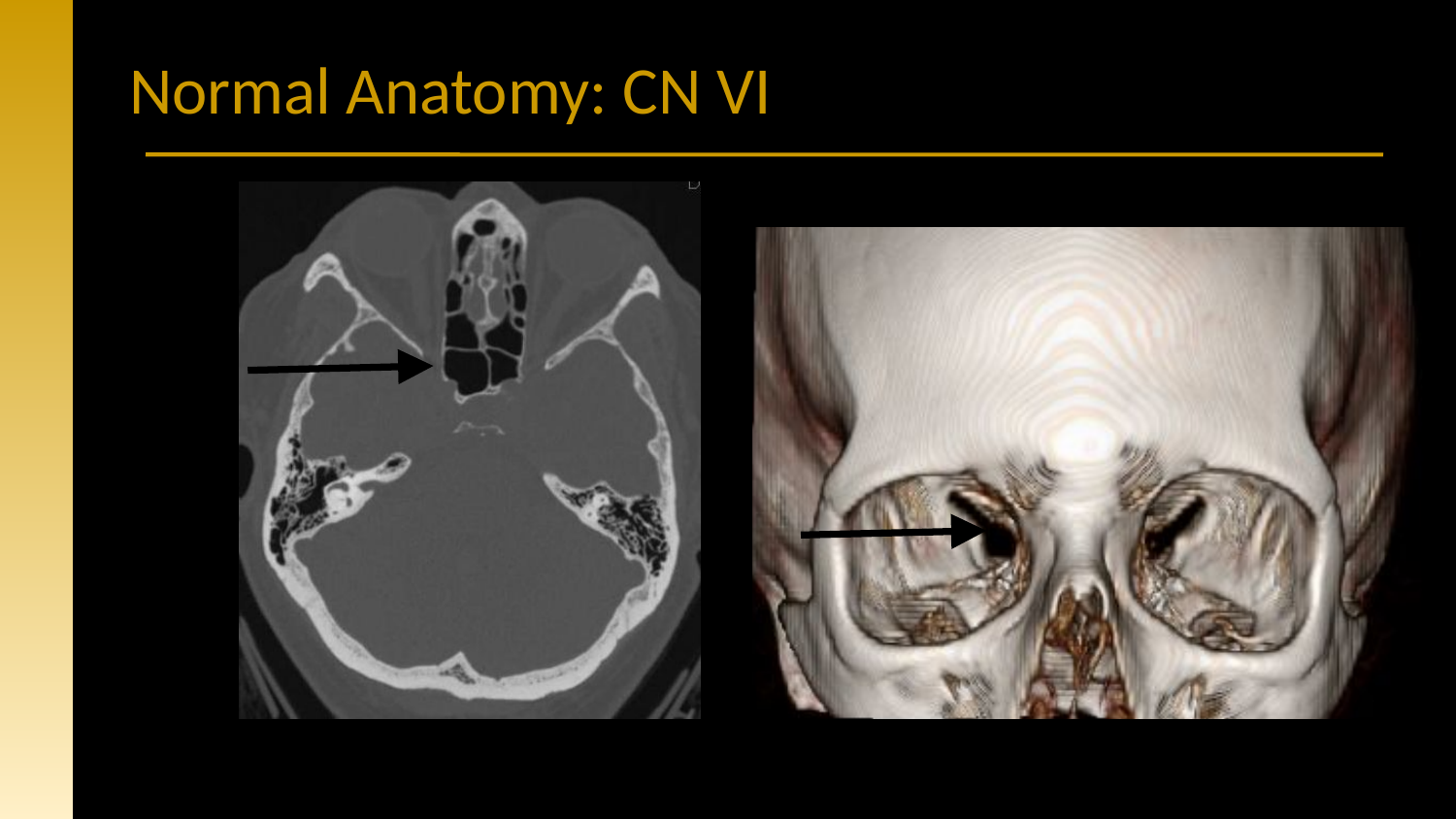

Normal Anatomy: CN VI

## Slide 40
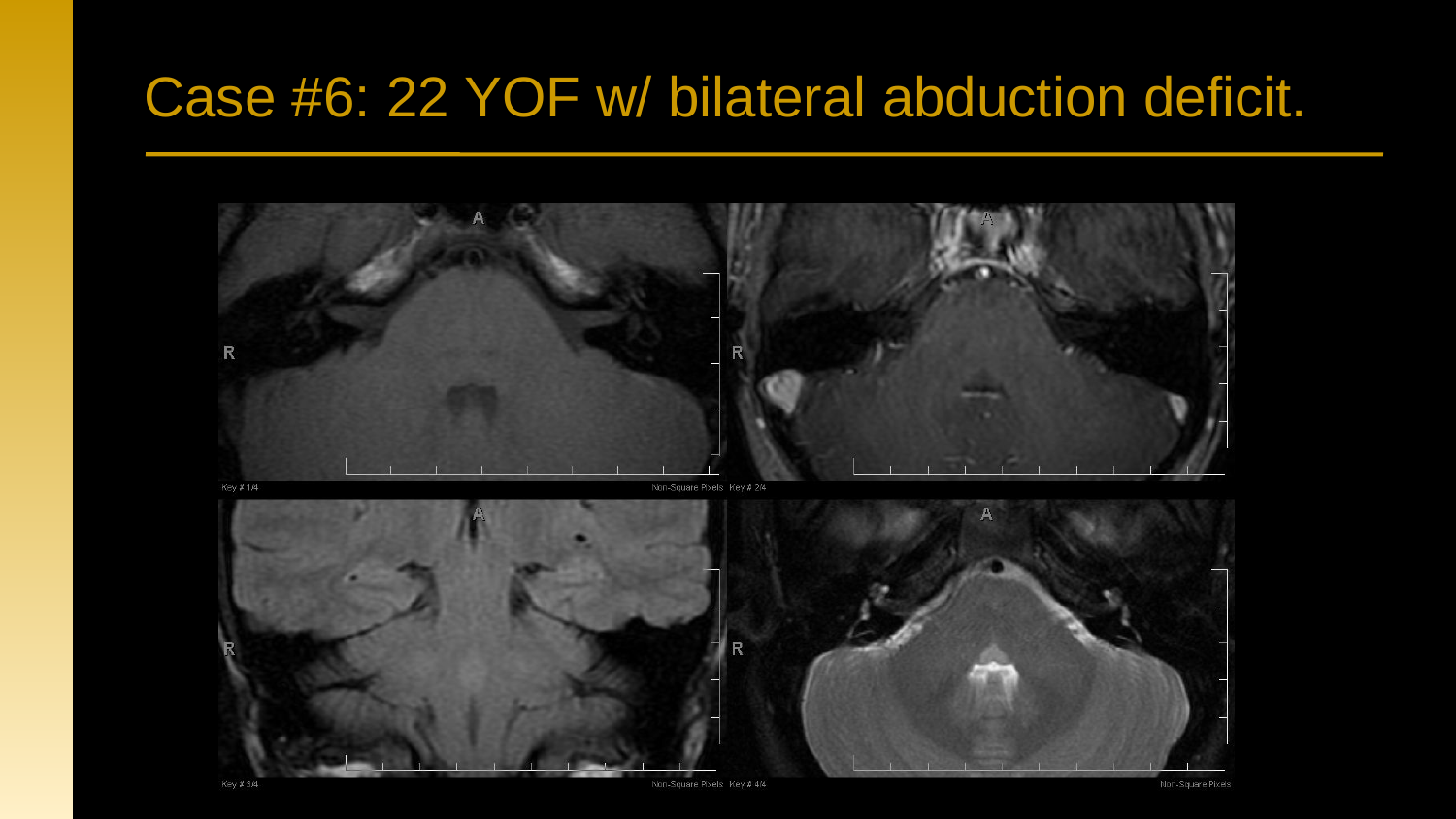

Case #6: 22 YOF w/ bilateral abduction deficit.

## Slide 41
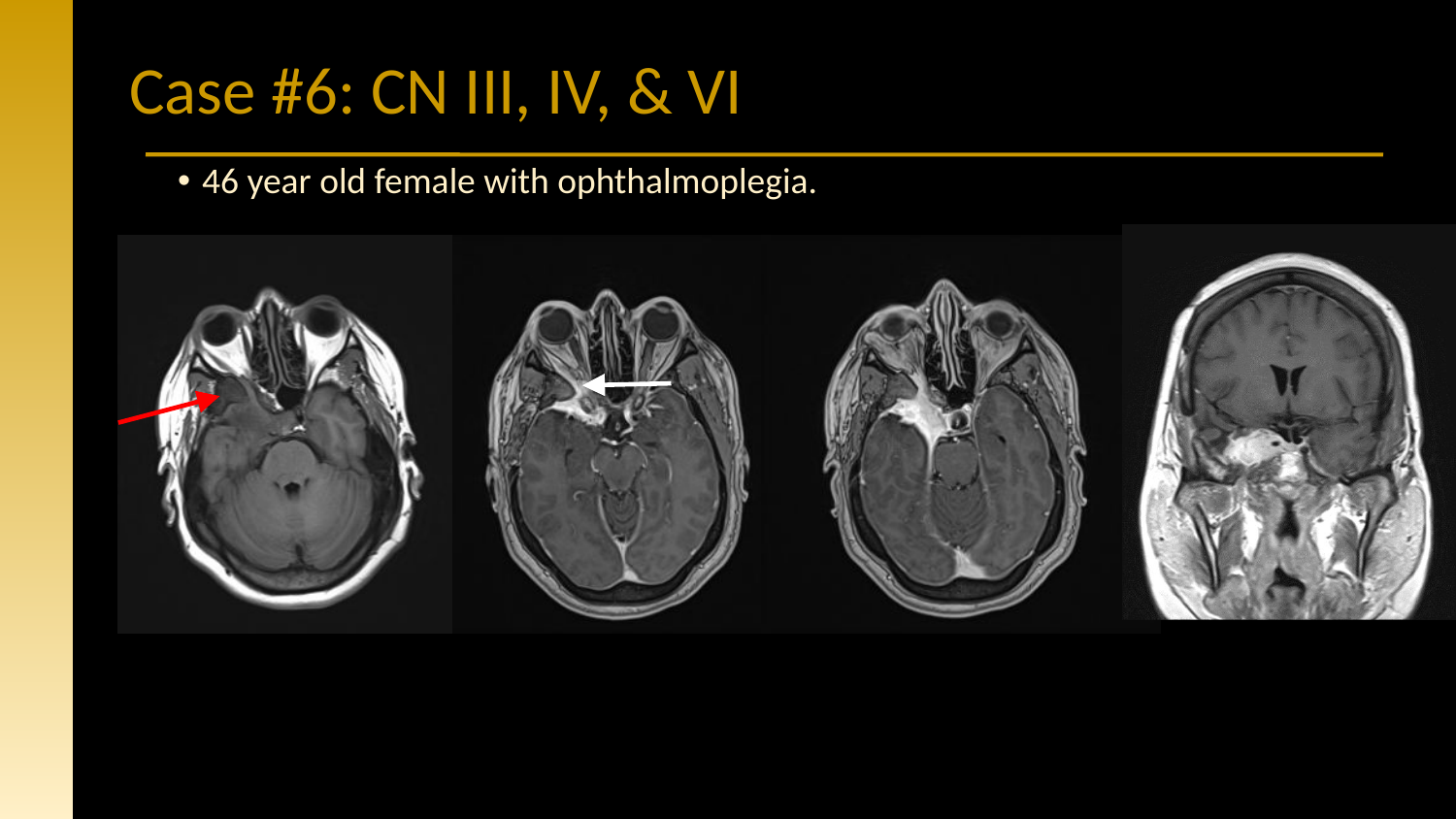

Case #6: CN III, IV, & VI
46 year old female with ophthalmoplegia.

## Slide 42
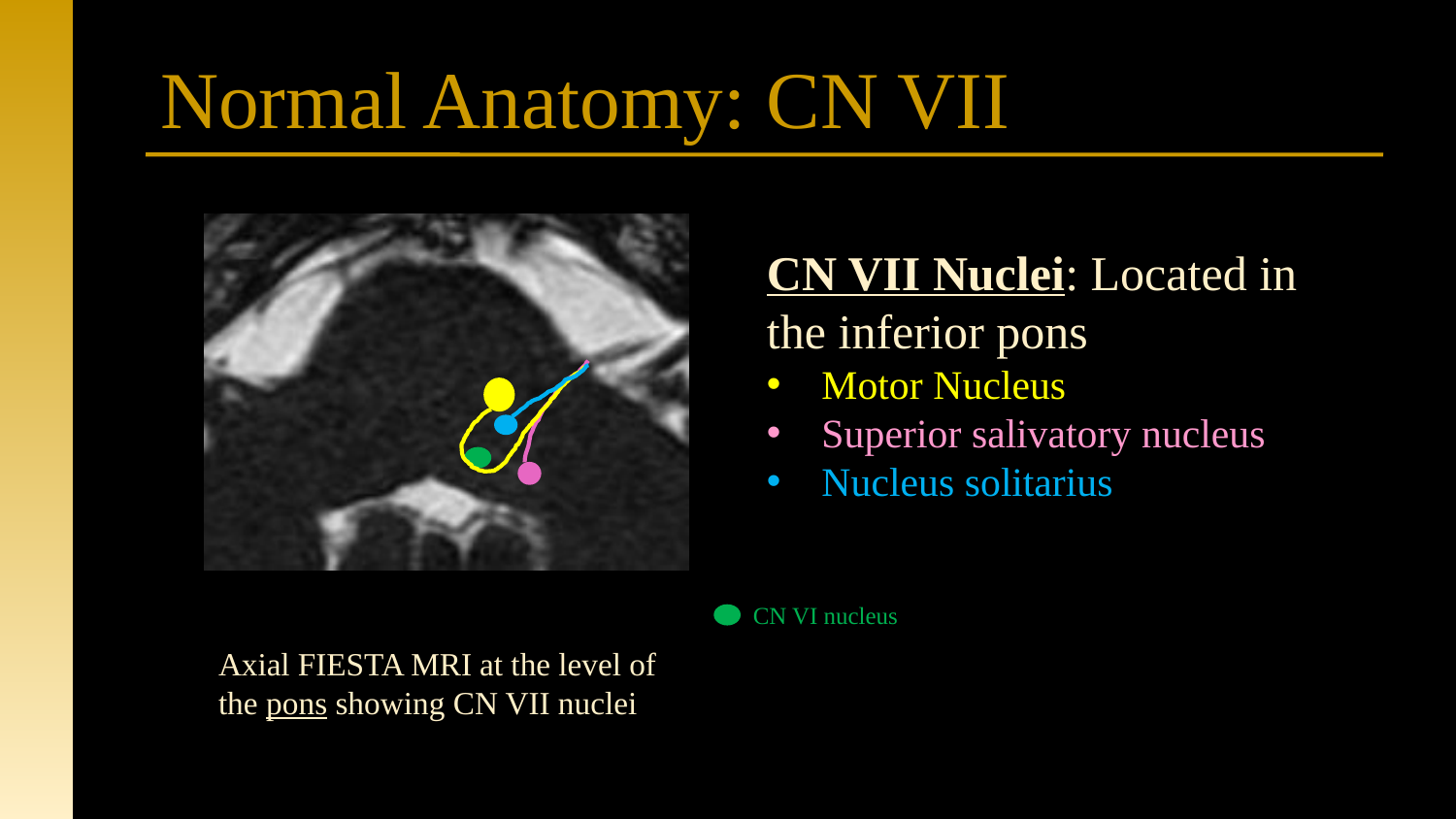

# Normal Anatomy: CN VII
CN VII Nuclei: Located in the inferior pons
Motor Nucleus
Superior salivatory nucleus
Nucleus solitarius
CN VI nucleus
Axial FIESTA MRI at the level of the pons showing CN VII nuclei

## Slide 43
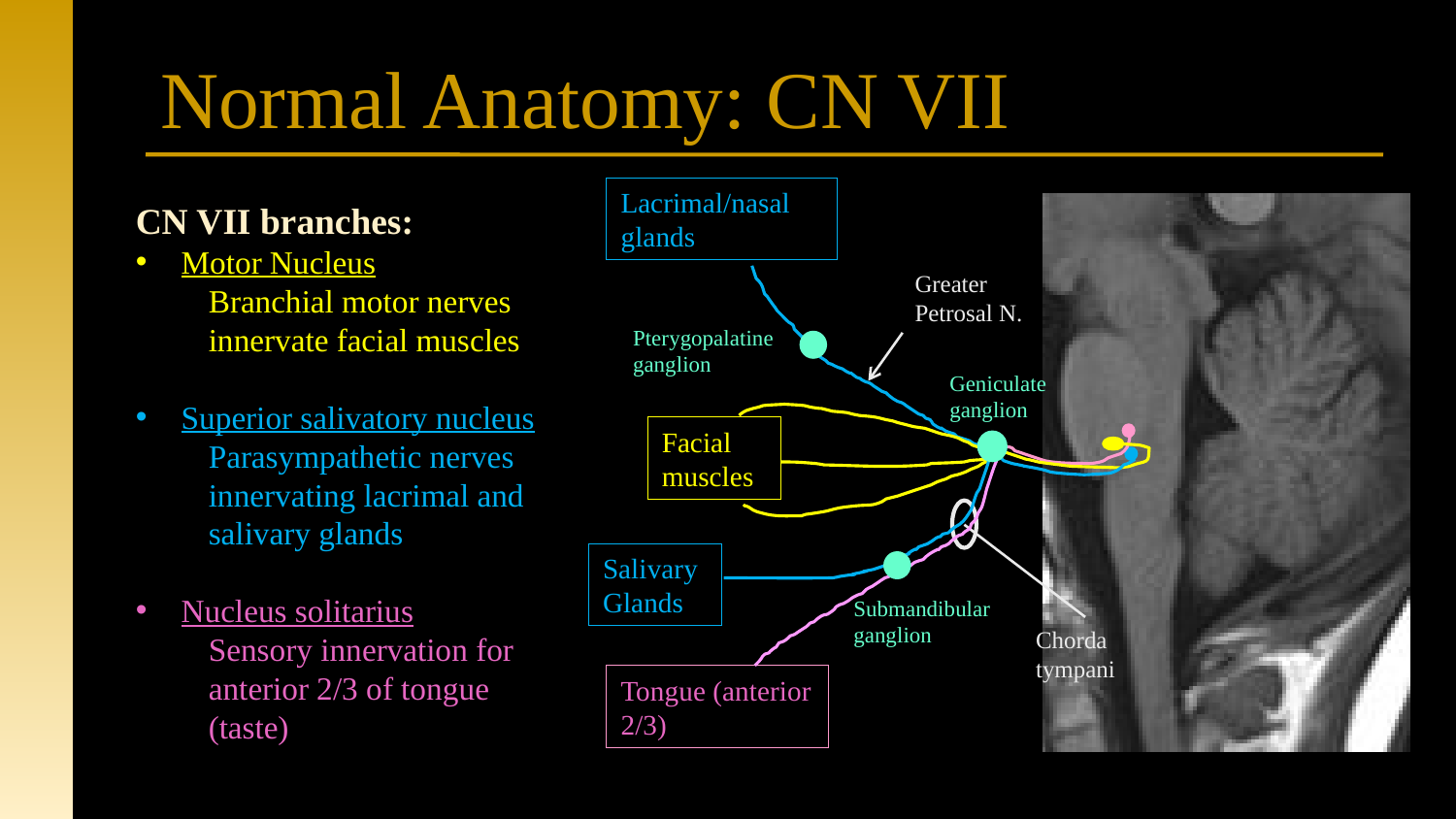

# Normal Anatomy: CN VII
Lacrimal/nasal glands
CN VII branches:
Motor Nucleus
Branchial motor nerves innervate facial muscles
Superior salivatory nucleus
Parasympathetic nerves innervating lacrimal and salivary glands
Nucleus solitarius
Sensory innervation for anterior 2/3 of tongue (taste)
Greater Petrosal N.
Pterygopalatine ganglion
Geniculate ganglion
Facial muscles
Salivary Glands
Submandibular ganglion
Chorda tympani
Tongue (anterior 2/3)

## Slide 44
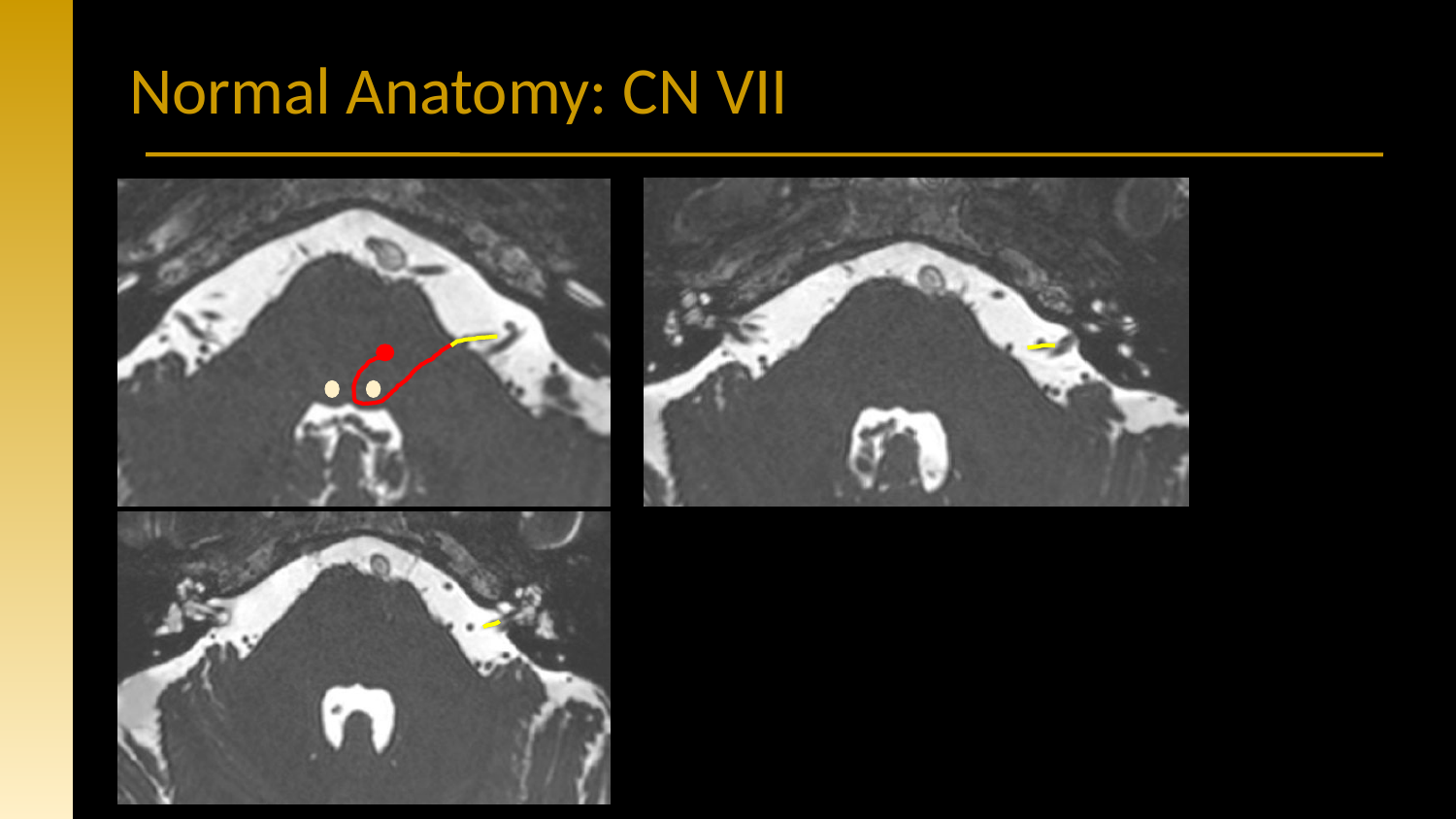

Normal Anatomy: CN VII

## Slide 45
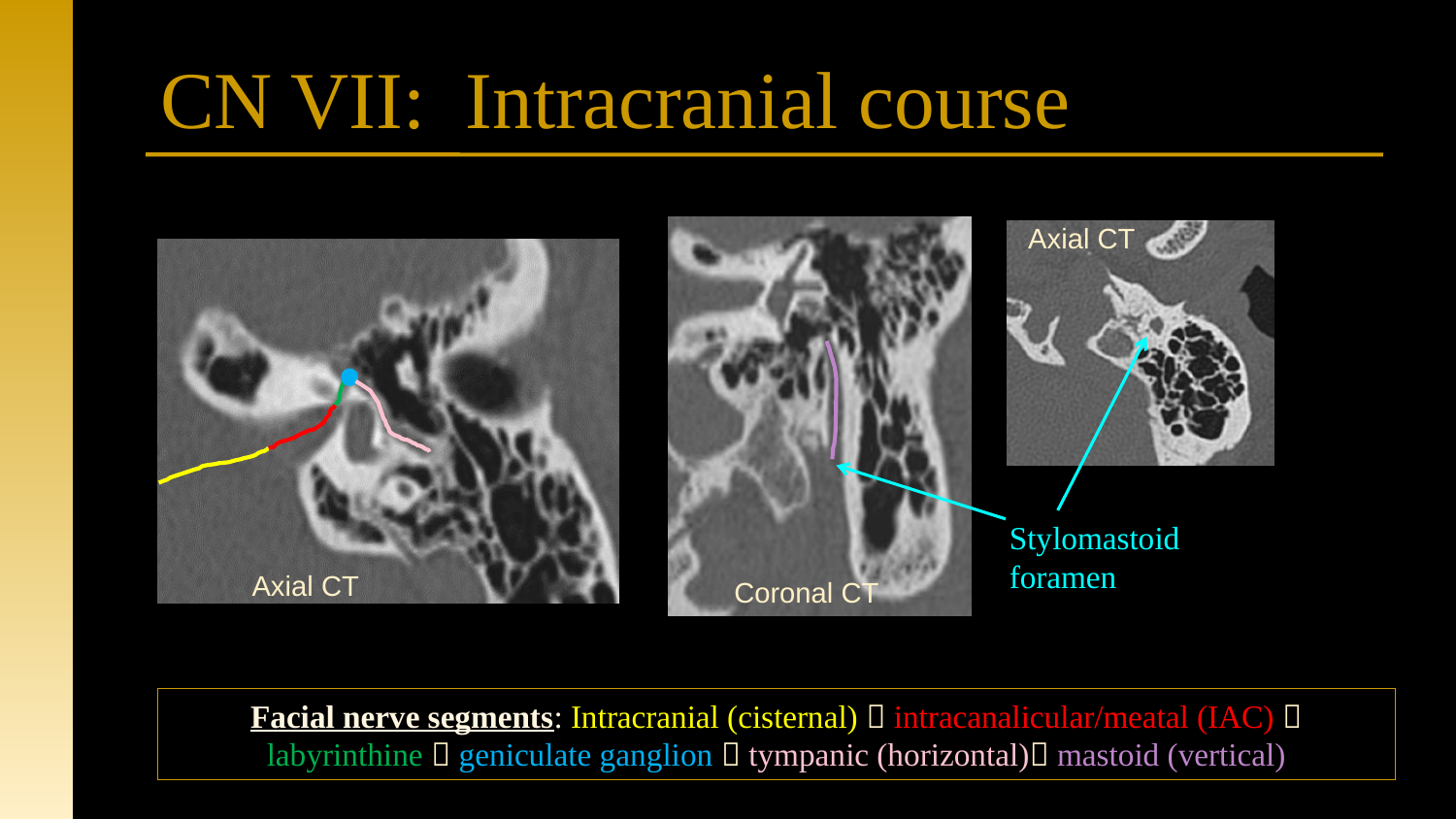

# CN VII: Intracranial course
Axial CT
Stylomastoid foramen
Coronal CT
Axial CT
Facial nerve segments: Intracranial (cisternal)  intracanalicular/meatal (IAC)  labyrinthine  geniculate ganglion  tympanic (horizontal) mastoid (vertical)

## Slide 46
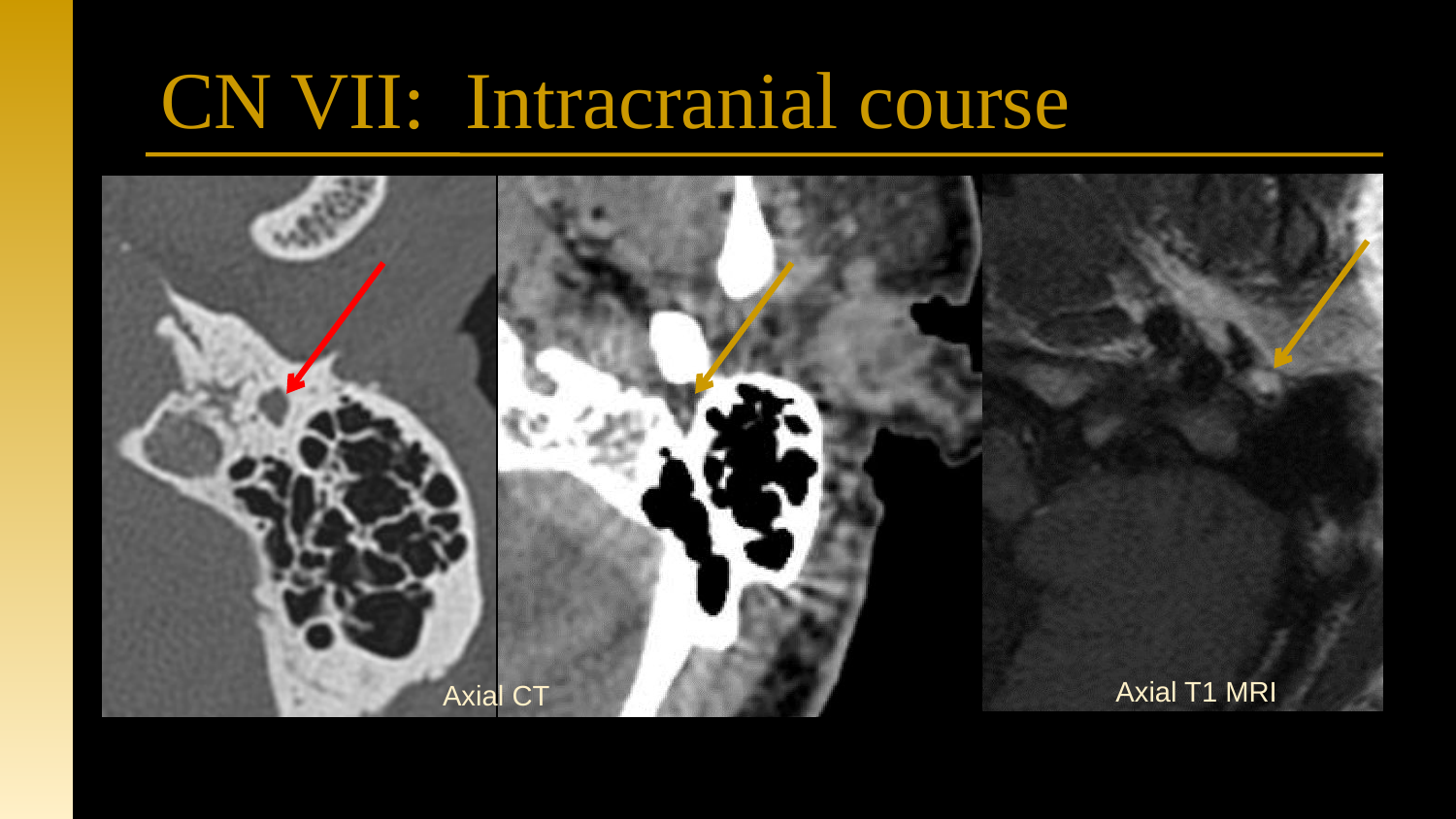

# CN VII: Intracranial course
Axial T1 MRI
Axial CT

## Slide 47
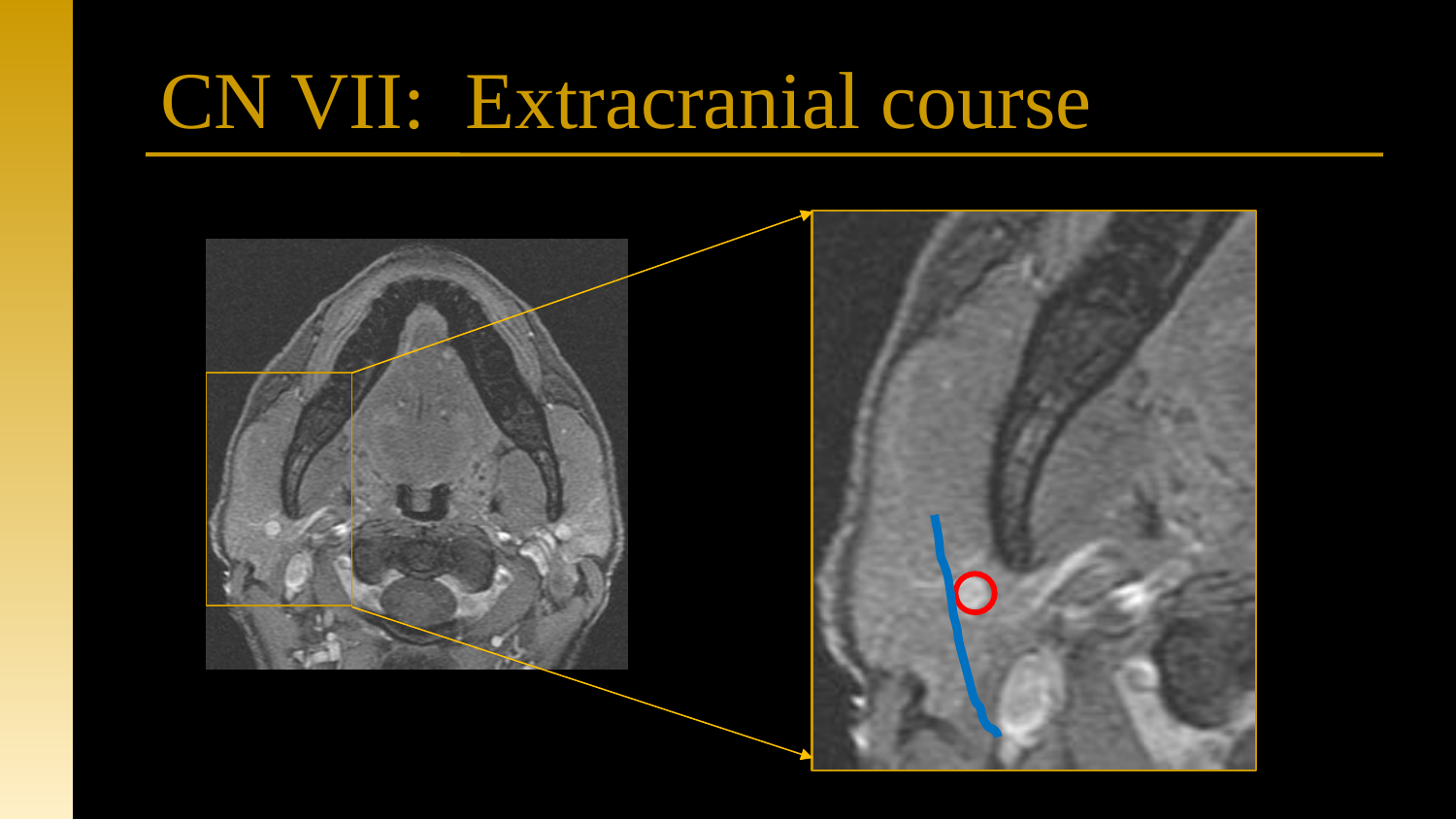

# CN VII: Extracranial course

## Slide 48
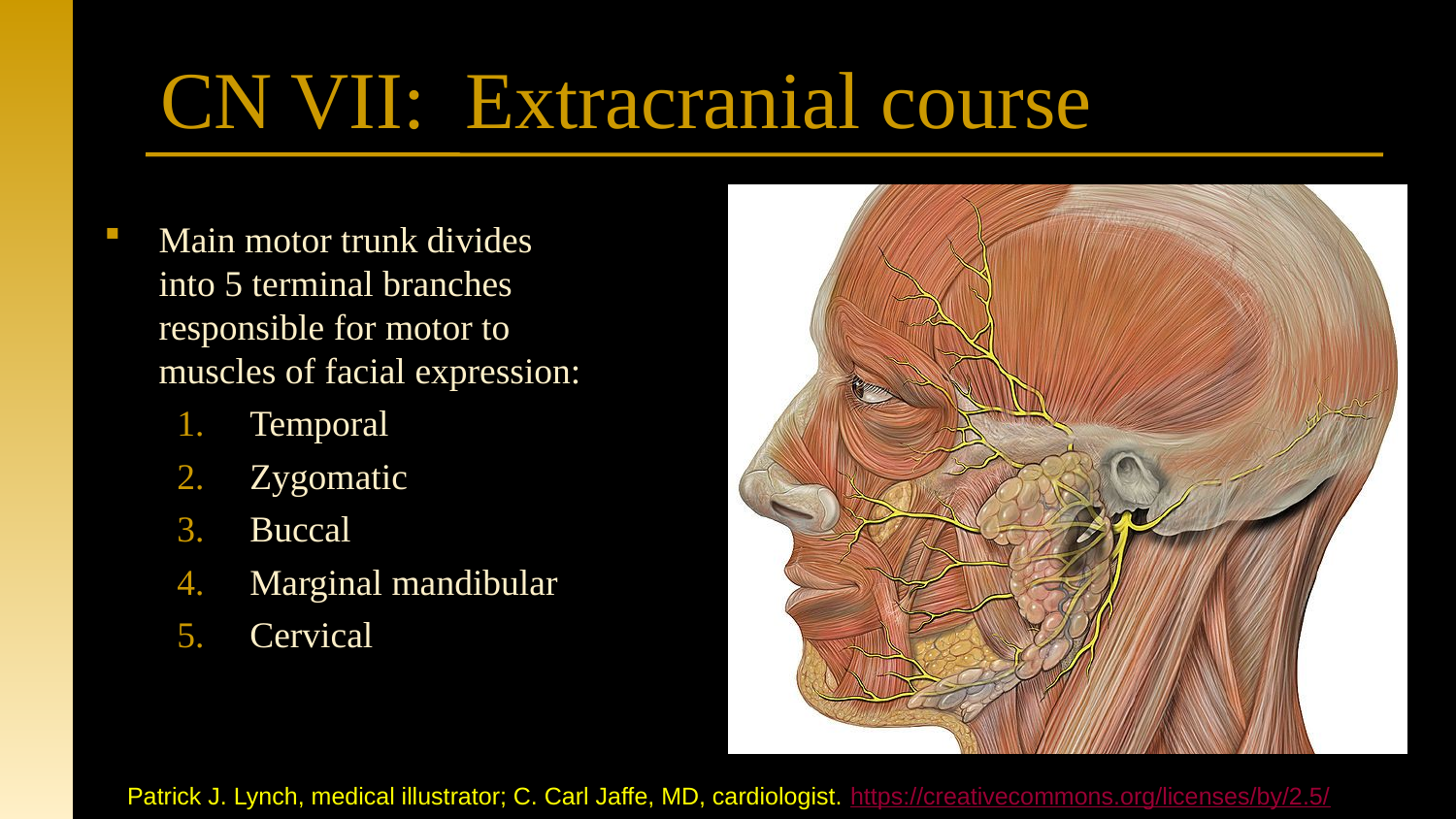

# CN VII: Extracranial course
Main motor trunk divides into 5 terminal branches responsible for motor to muscles of facial expression:
Temporal
Zygomatic
Buccal
Marginal mandibular
Cervical
Patrick J. Lynch, medical illustrator; C. Carl Jaffe, MD, cardiologist. https://creativecommons.org/licenses/by/2.5/

## Slide 49
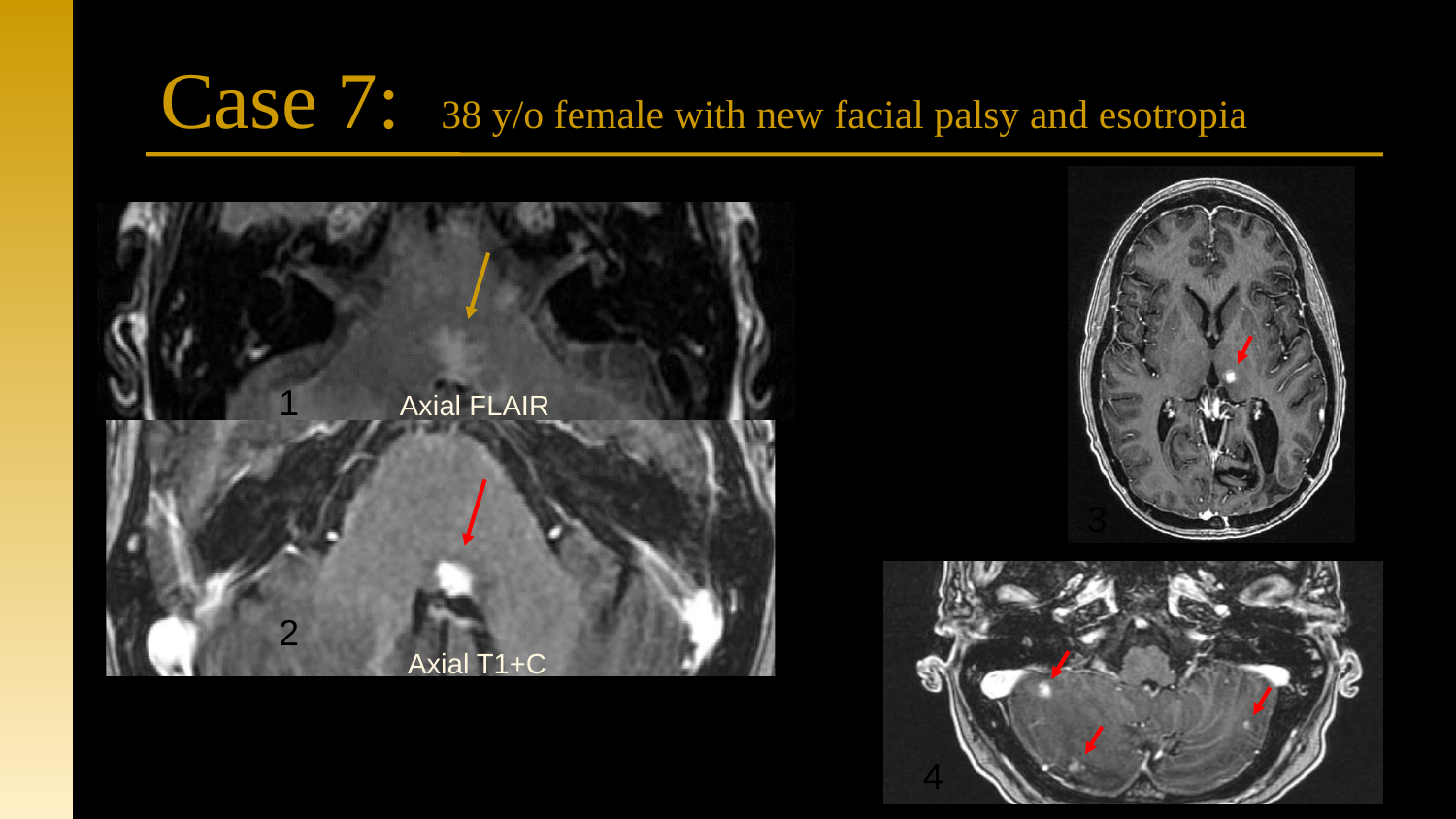

# Case 7: 38 y/o female with new facial palsy and esotropia
Axial FLAIR
Axial T1+C
1
CN_06
CN_06
3
2
4

## Slide 50
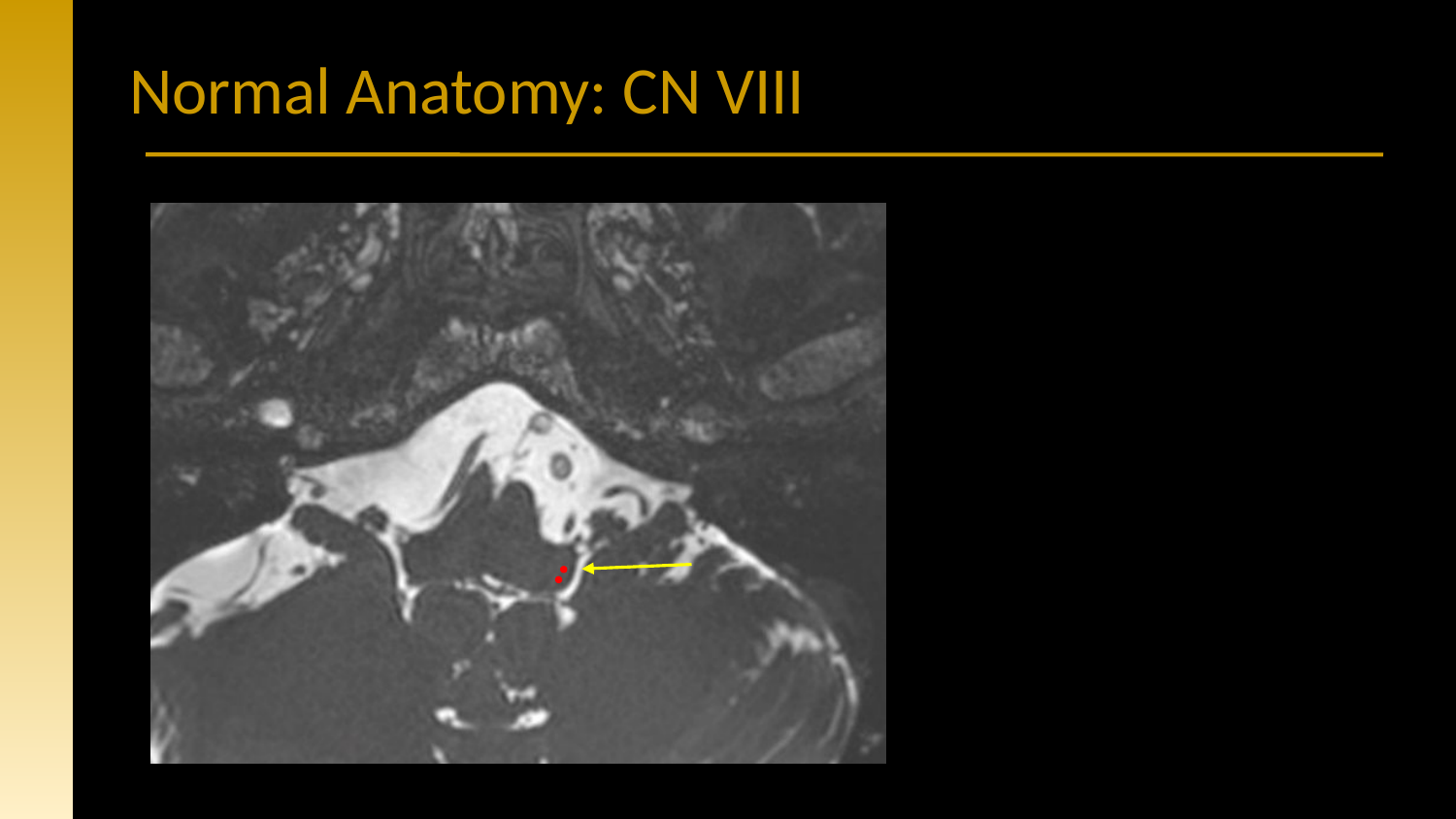

Normal Anatomy: CN VIII

## Slide 51
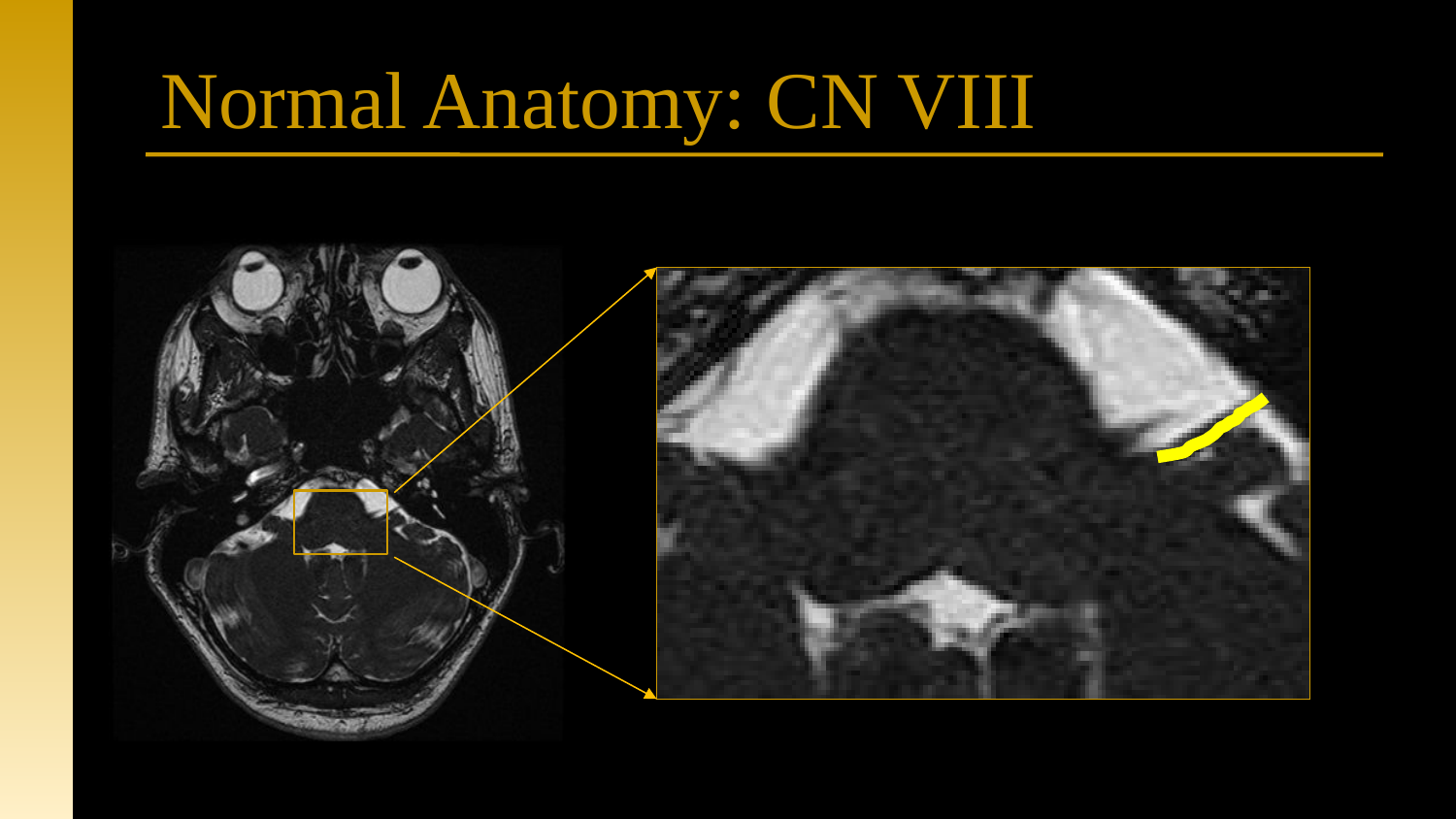

# Normal Anatomy: CN VIII

## Slide 52
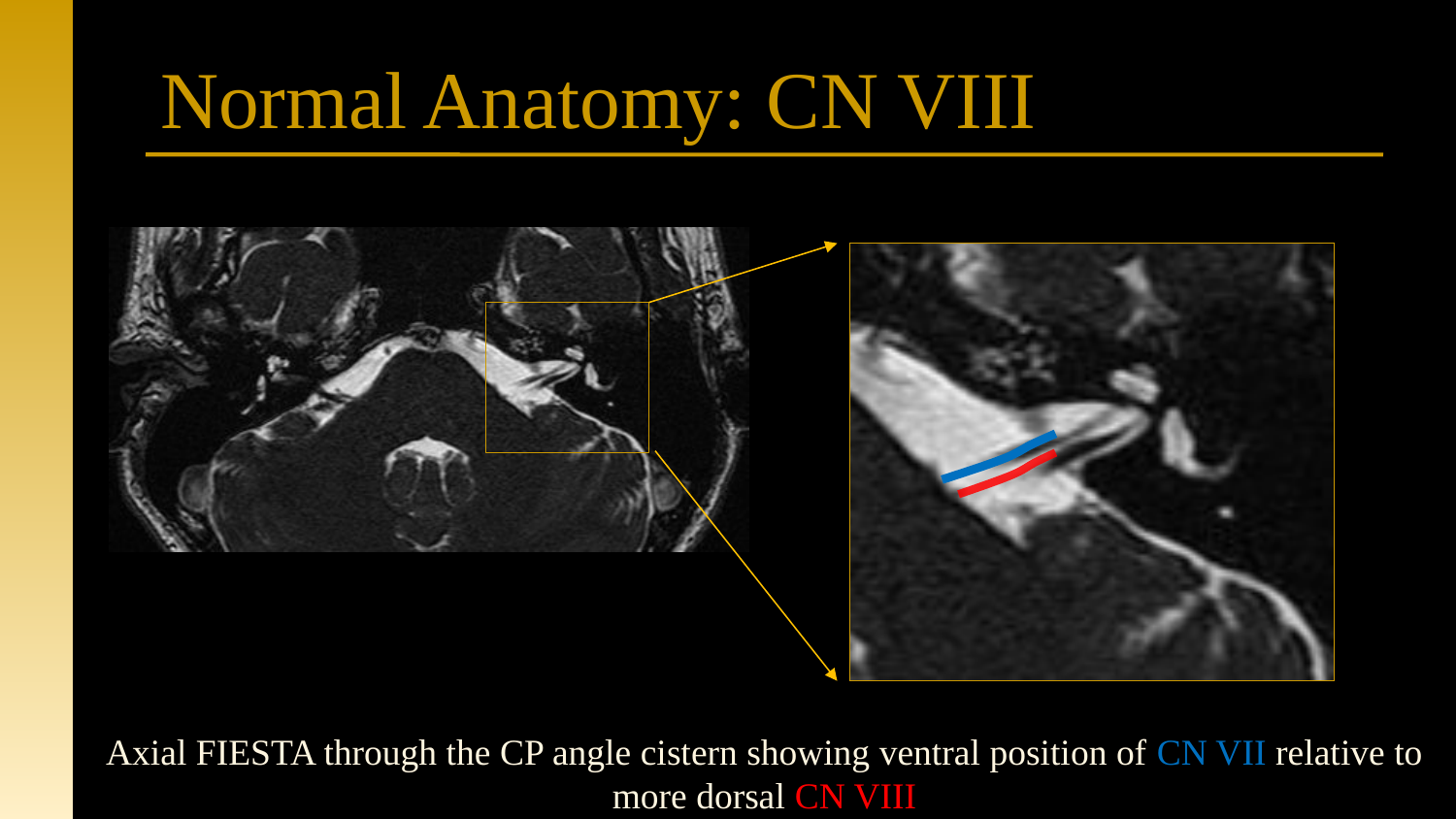

# Normal Anatomy: CN VIII
Axial FIESTA through the CP angle cistern showing ventral position of CN VII relative to more dorsal CN VIII

## Slide 53
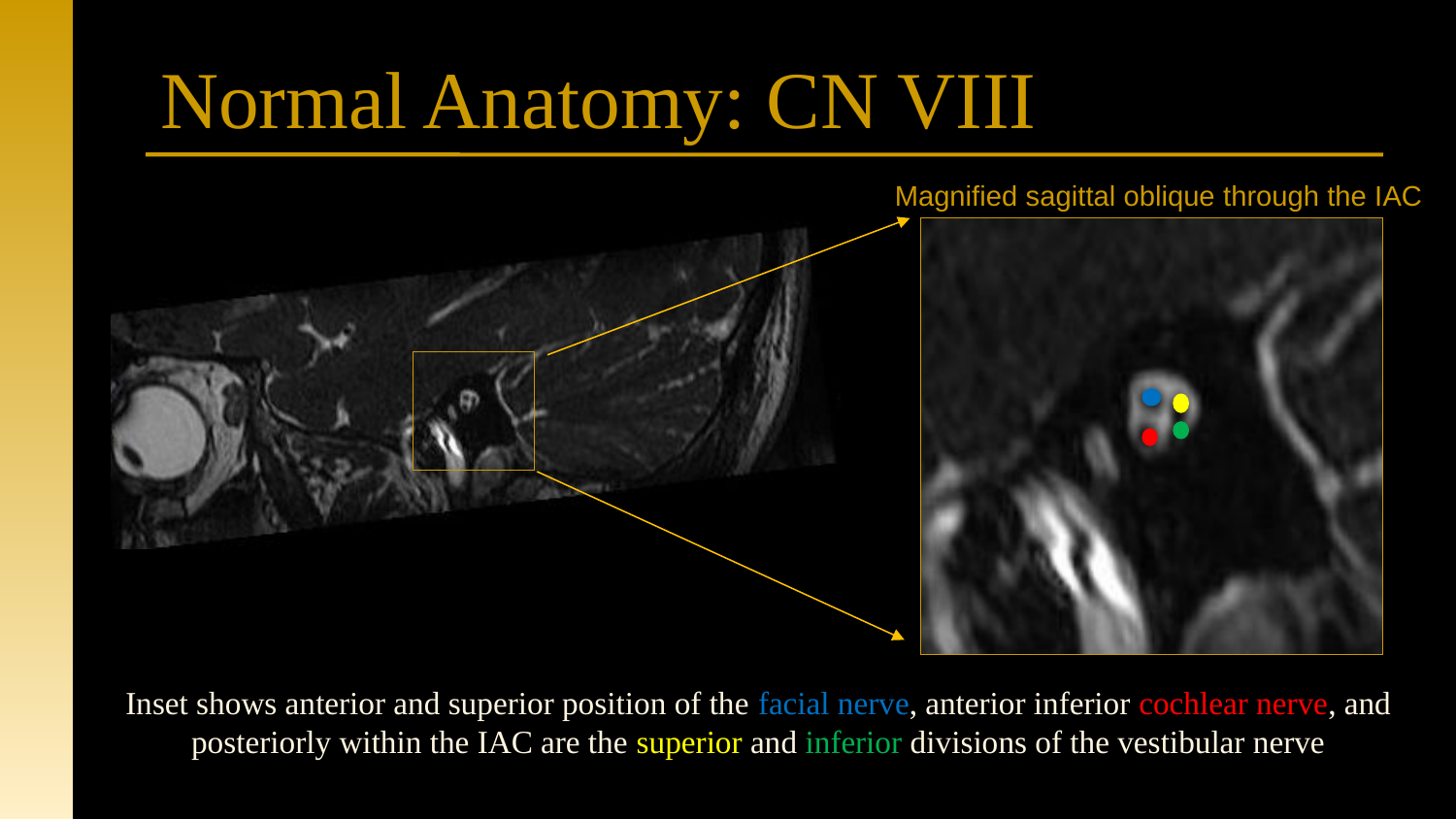

# Normal Anatomy: CN VIII
Magnified sagittal oblique through the IAC
Inset shows anterior and superior position of the facial nerve, anterior inferior cochlear nerve, and posteriorly within the IAC are the superior and inferior divisions of the vestibular nerve

## Slide 54
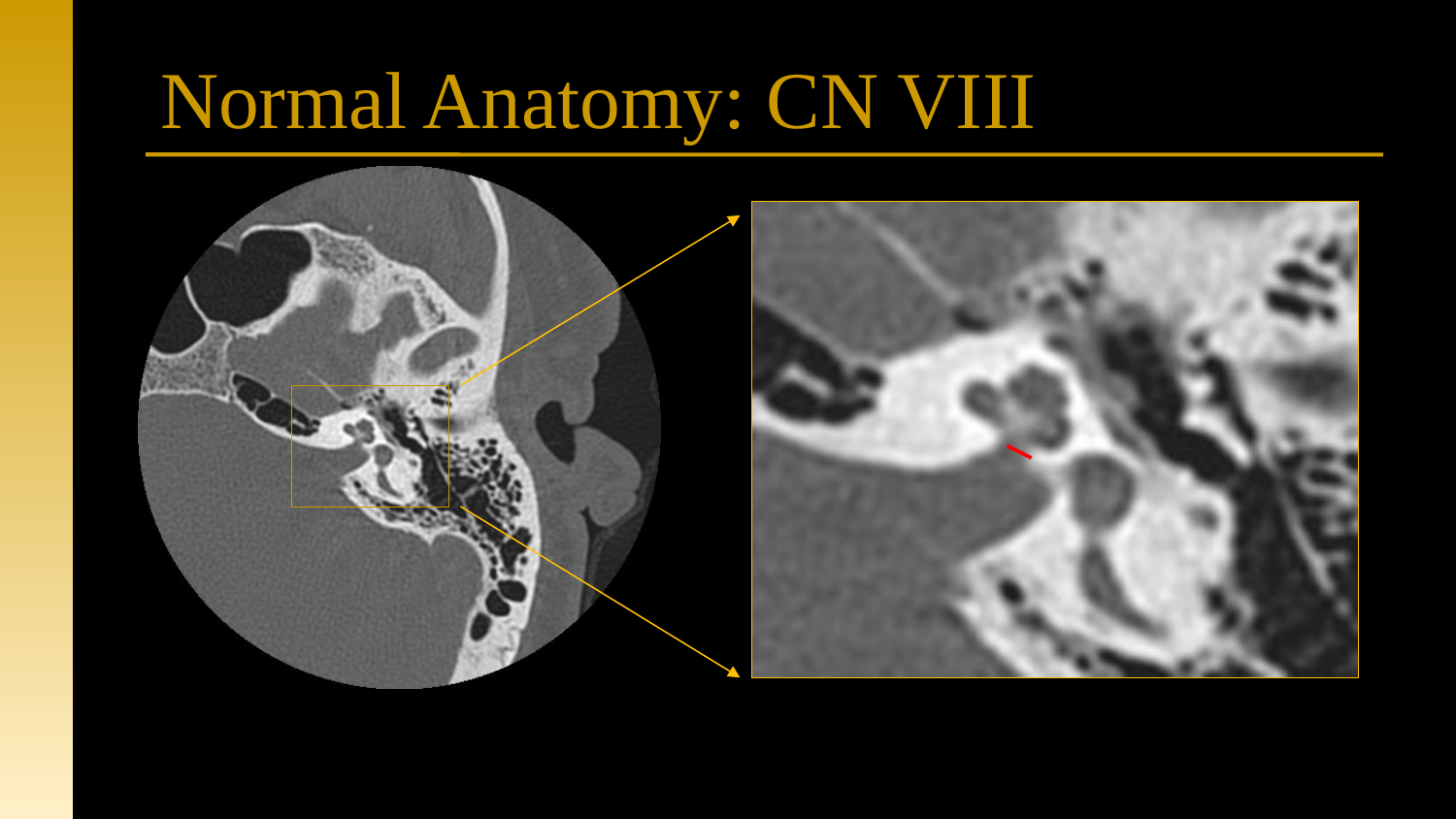

# Normal Anatomy: CN VIII

## Slide 55
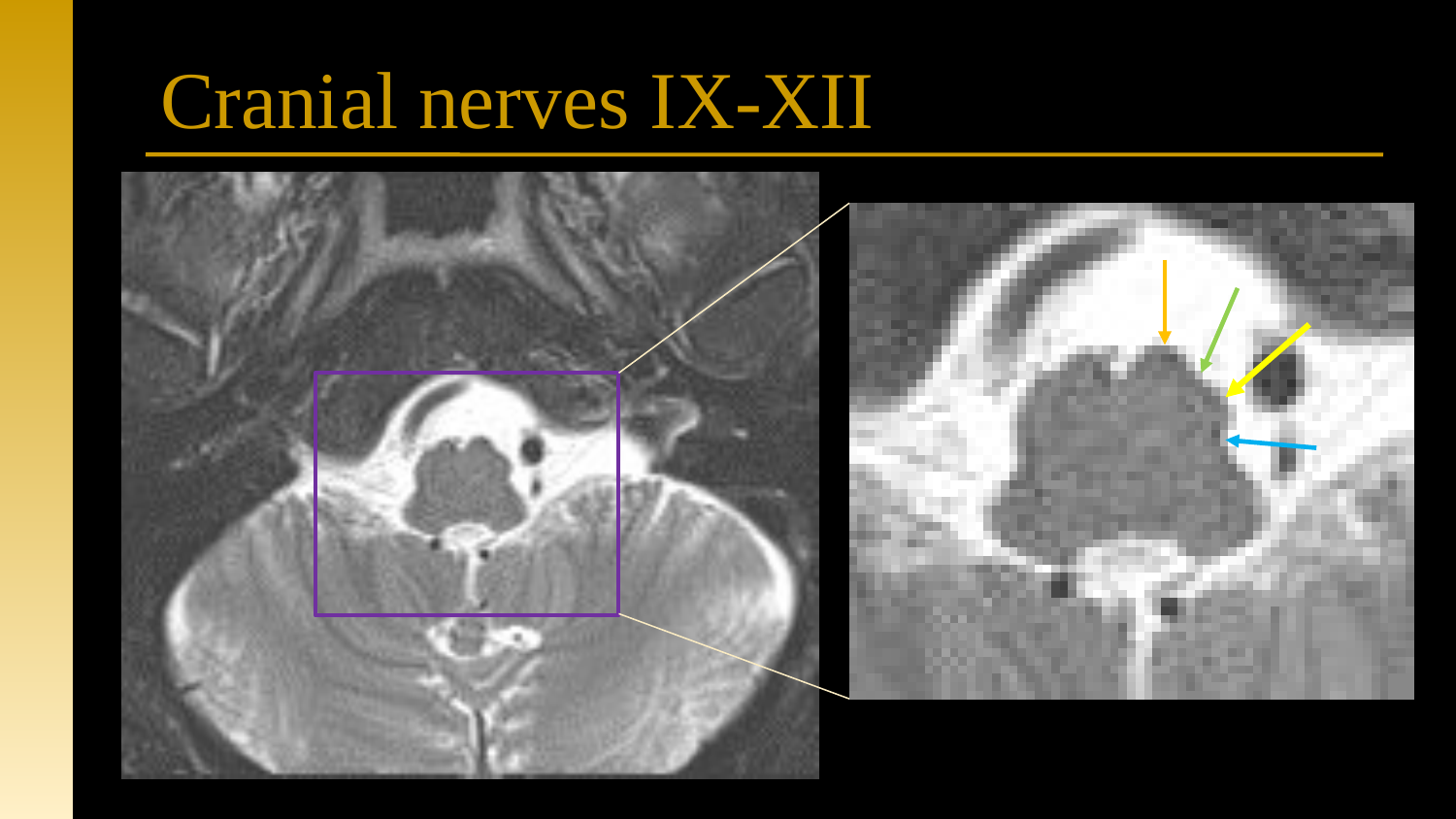

# Cranial nerves IX-XII

## Slide 56
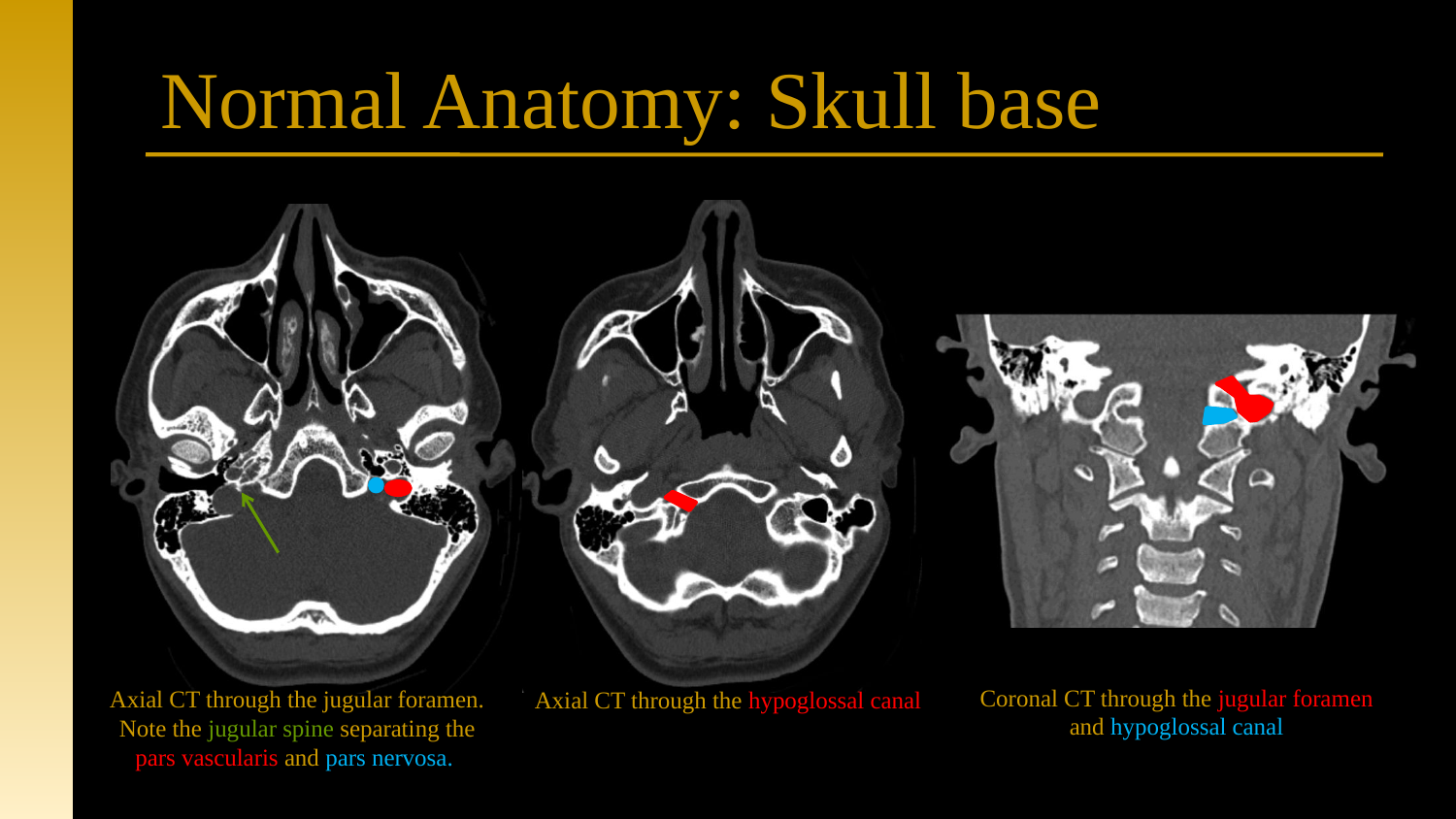

# Normal Anatomy: Skull base
Axial CT through the hypoglossal canal
Axial CT through the jugular foramen. Note the jugular spine separating the
pars vascularis and pars nervosa.
Coronal CT through the jugular foramen and hypoglossal canal

## Slide 57
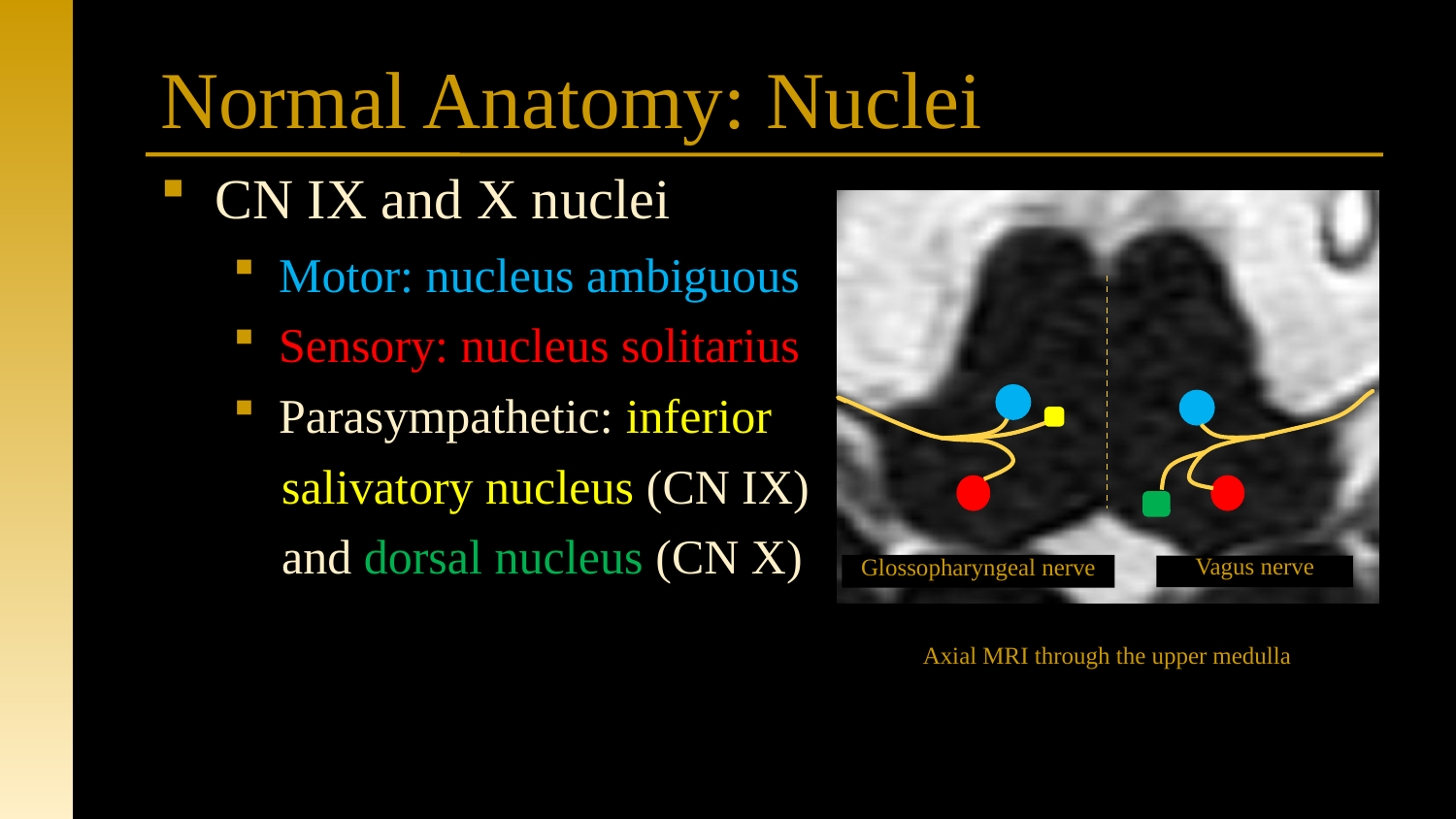

# Normal Anatomy: Nuclei
CN IX and X nuclei
Motor: nucleus ambiguous
Sensory: nucleus solitarius
Parasympathetic: inferior
 salivatory nucleus (CN IX)
 and dorsal nucleus (CN X)
Glossopharyngeal nerve
Vagus nerve
Axial MRI through the upper medulla

## Slide 58
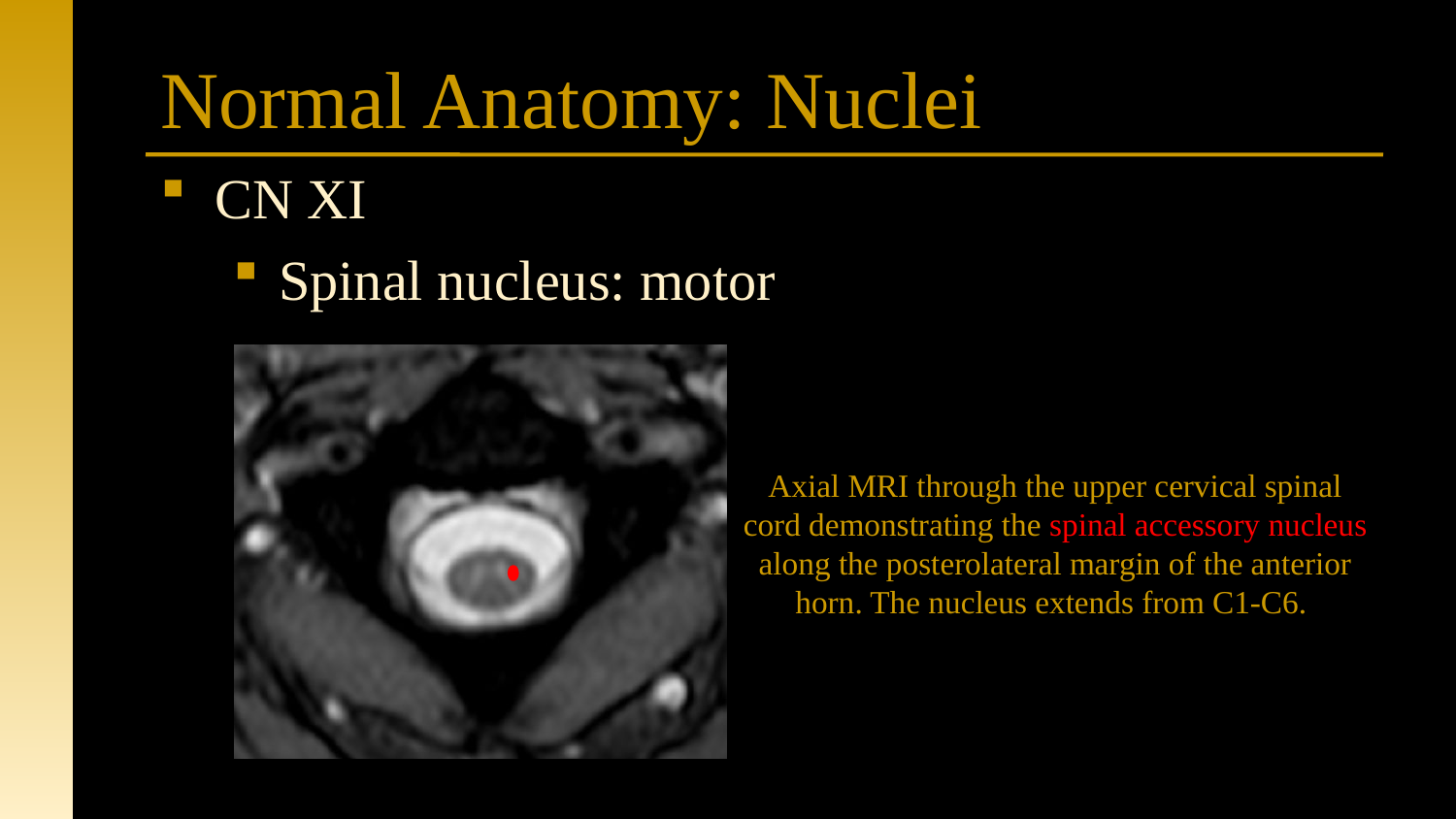

# Normal Anatomy: Nuclei
CN XI
Spinal nucleus: motor
Axial MRI through the upper cervical spinal cord demonstrating the spinal accessory nucleus along the posterolateral margin of the anterior horn. The nucleus extends from C1-C6.

## Slide 59
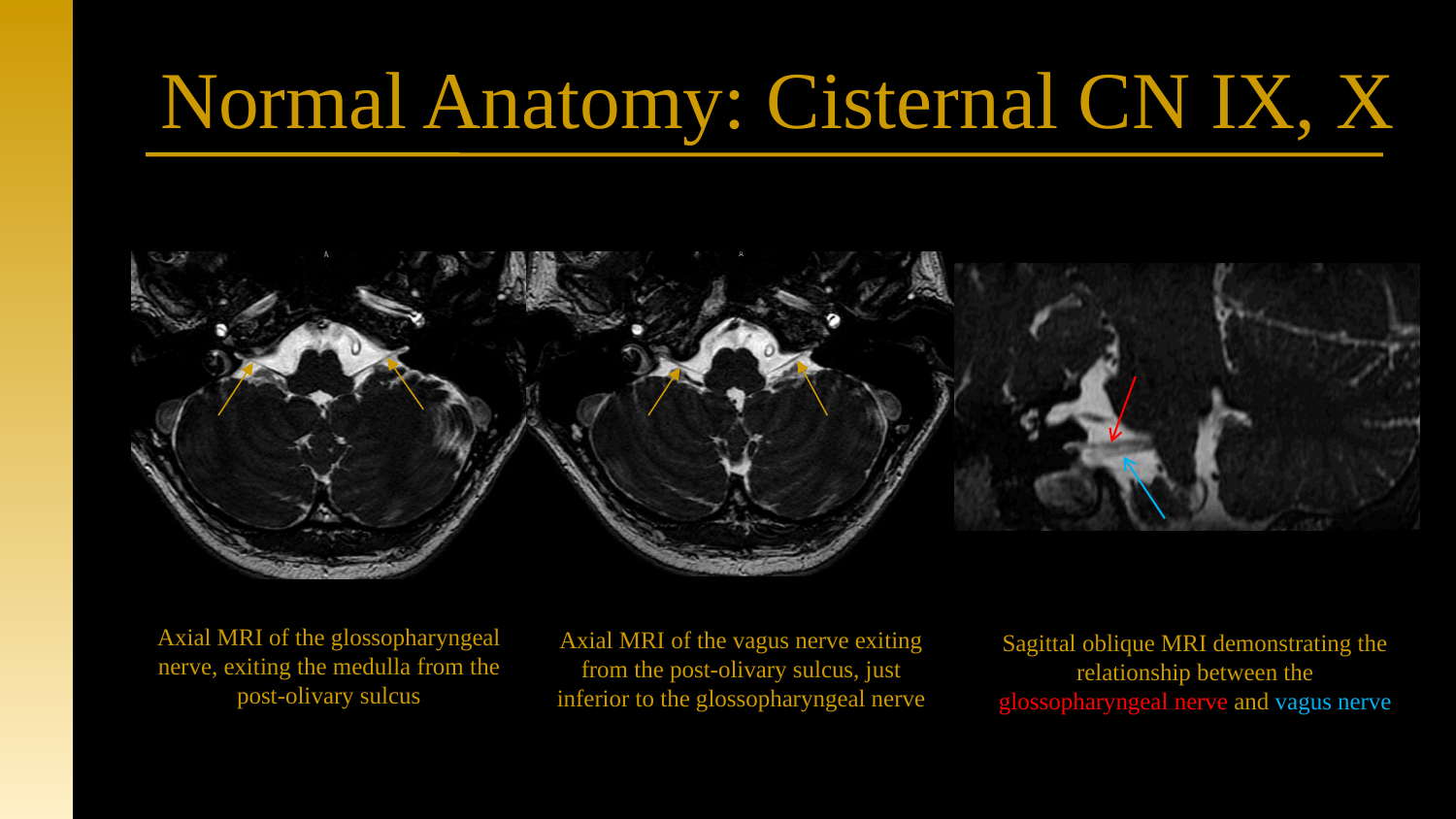

# Normal Anatomy: Cisternal CN IX, X
Sagittal oblique MRI demonstrating the relationship between the glossopharyngeal nerve and vagus nerve
Axial MRI of the vagus nerve exiting from the post-olivary sulcus, just inferior to the glossopharyngeal nerve
Axial MRI of the glossopharyngeal nerve, exiting the medulla from the post-olivary sulcus

## Slide 60
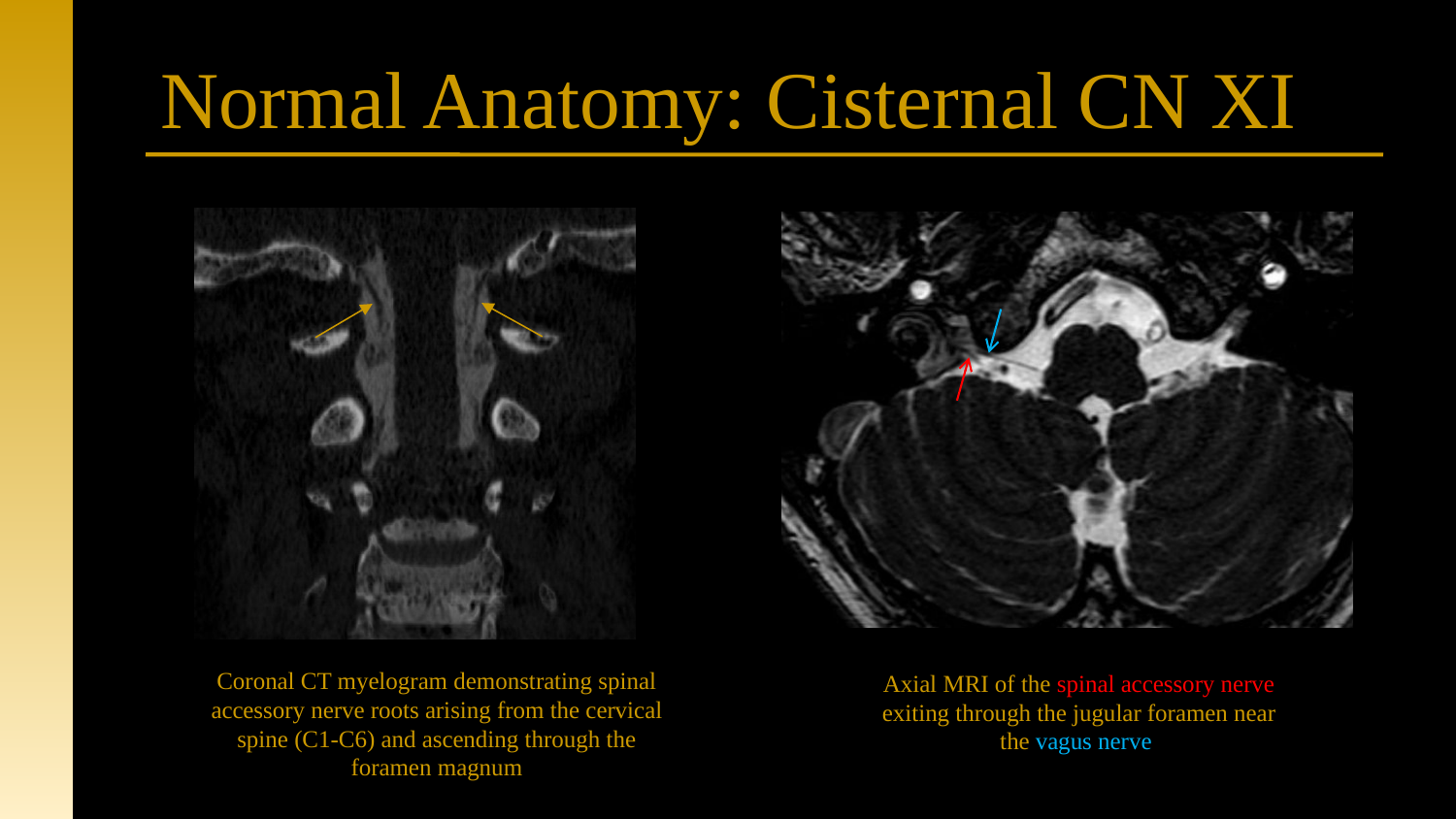

# Normal Anatomy: Cisternal CN XI
Axial MRI of the spinal accessory nerve exiting through the jugular foramen near the vagus nerve
Coronal CT myelogram demonstrating spinal accessory nerve roots arising from the cervical spine (C1-C6) and ascending through the foramen magnum

## Slide 61
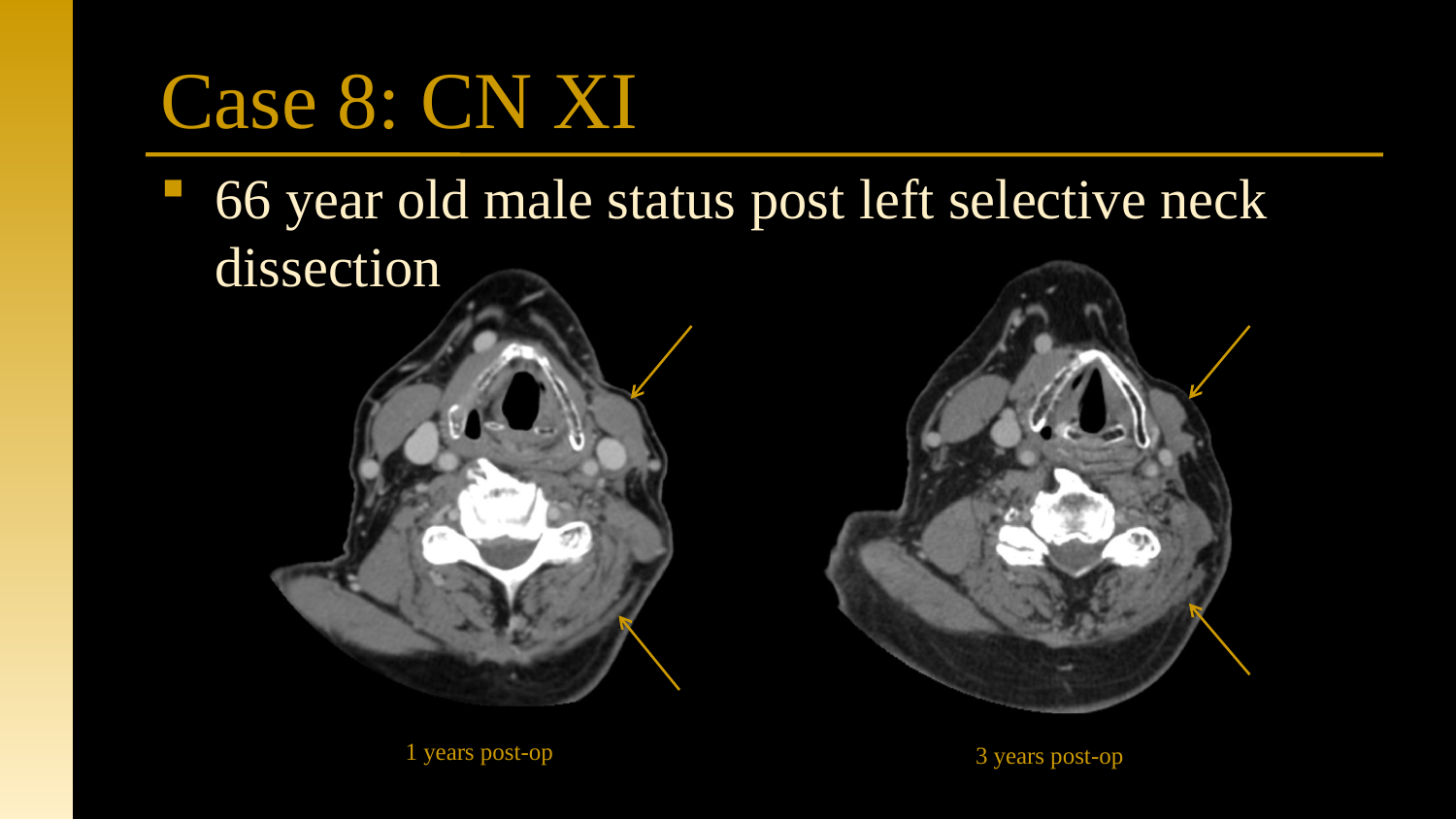

# Case 8: CN XI
66 year old male status post left selective neck dissection
CN_06
1 years post-op
3 years post-op

## Slide 62
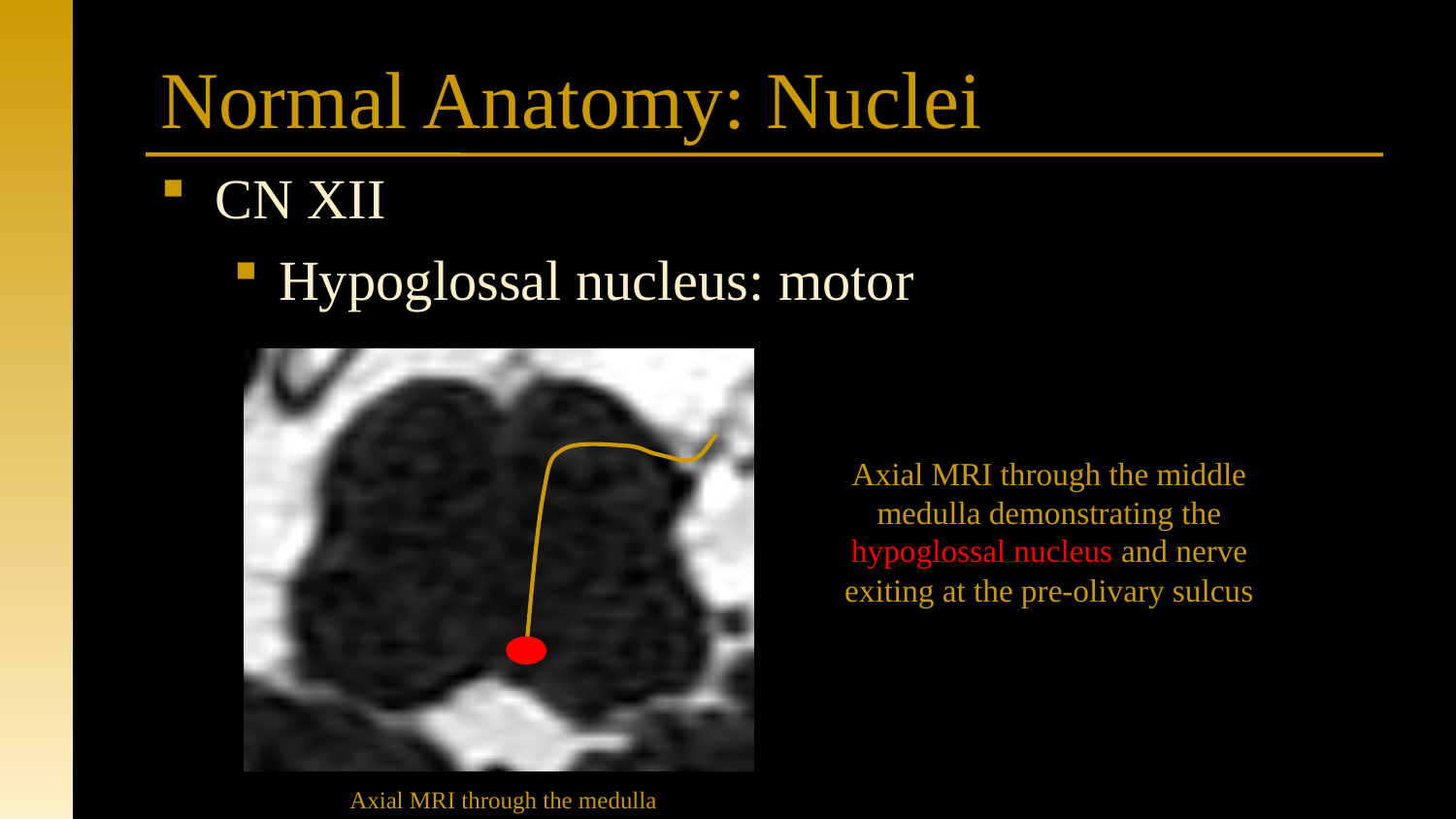

# Normal Anatomy: Nuclei
CN XII
Hypoglossal nucleus: motor
Axial MRI through the middle medulla demonstrating the hypoglossal nucleus and nerve exiting at the pre-olivary sulcus
Axial MRI through the medulla

## Slide 63
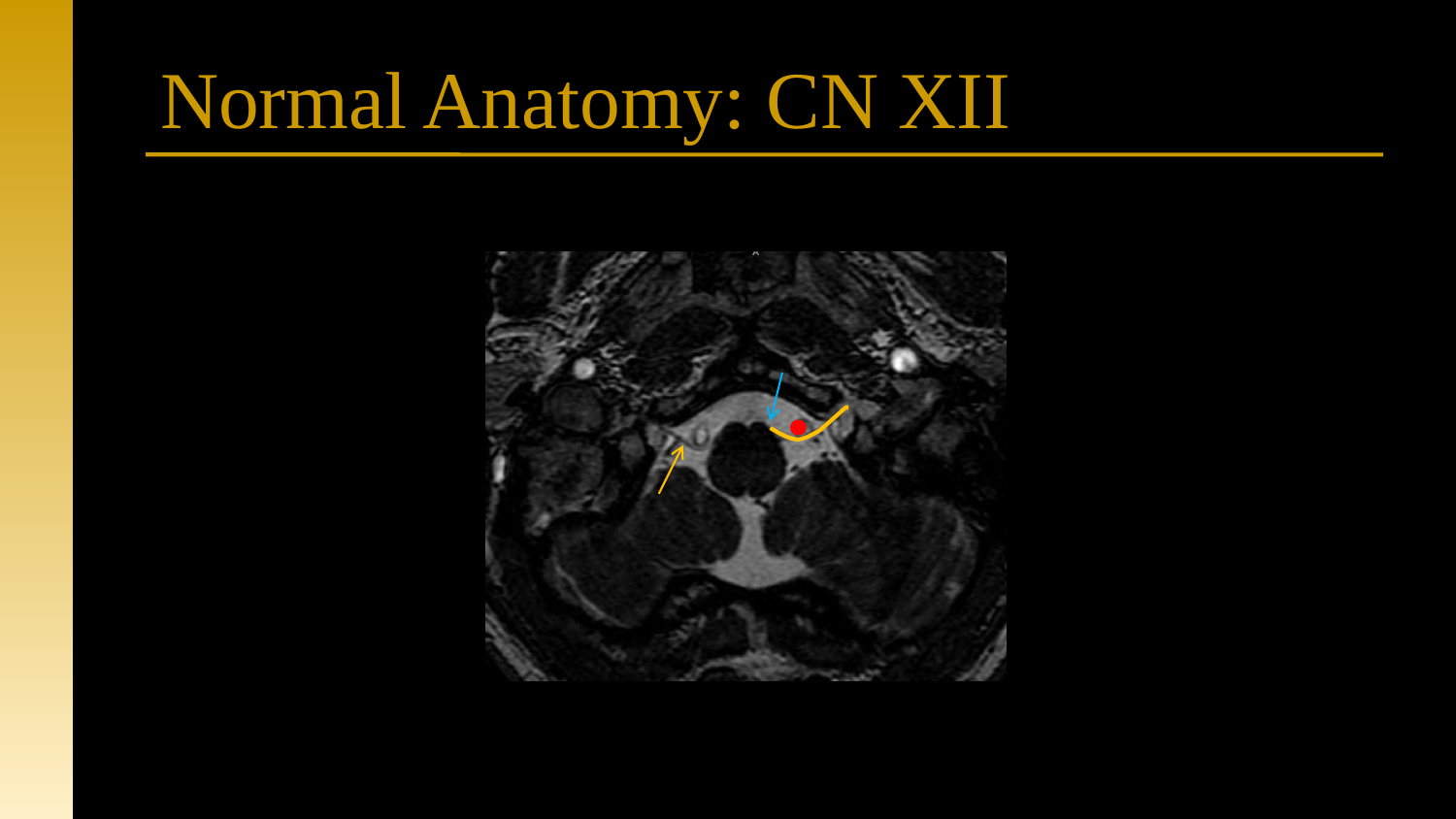

# Normal Anatomy: CN XII

## Slide 64
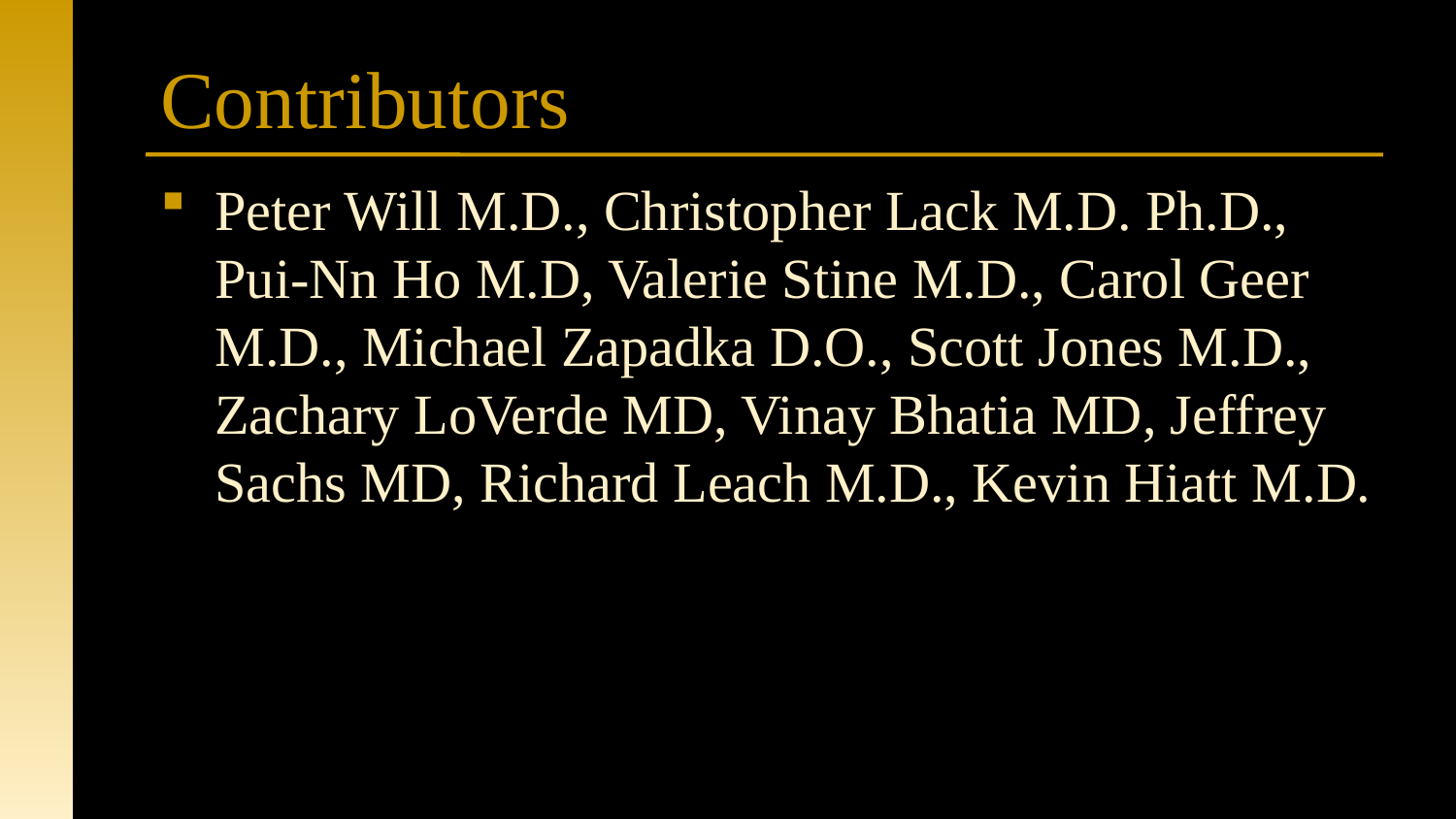

# Contributors
Peter Will M.D., Christopher Lack M.D. Ph.D., Pui-Nn Ho M.D, Valerie Stine M.D., Carol Geer M.D., Michael Zapadka D.O., Scott Jones M.D., Zachary LoVerde MD, Vinay Bhatia MD, Jeffrey Sachs MD, Richard Leach M.D., Kevin Hiatt M.D.
