# Supplementary material for: Cranial Nerve Anatomy Using a Modular and Multimodal Radiologic Approach
Source: MedEdPORTAL. 2022 Jun 10;18:11261. doi: 10.15766/mep_2374-8265.11261 (PMC9184306; doi:10.15766/mep_2374-8265.11261)
Supplement: Supplementary file 1 — Self-guided Anatomy Review.pptxCranial Nerve Video.mp4Cranial Nerve Lecture.pptxNeuroanatomy Lab.pptxNormal MRI and CT Scans - CT Bone Axials.pptxNormal MRI and CT Scans - T1 Sagittal.pptxNormal MRI and CT Scans - T2 Axial.pptxNormal MRI and CT Scans - T2 SPACE Axial.pptxPre- and Posttest.pptxSatisfaction Survey.docxAppendix Guide.docx [file mep_2374-8265.11261-s001.zip › D. Neuroanatomy Lab.pptx]

## Slide 1
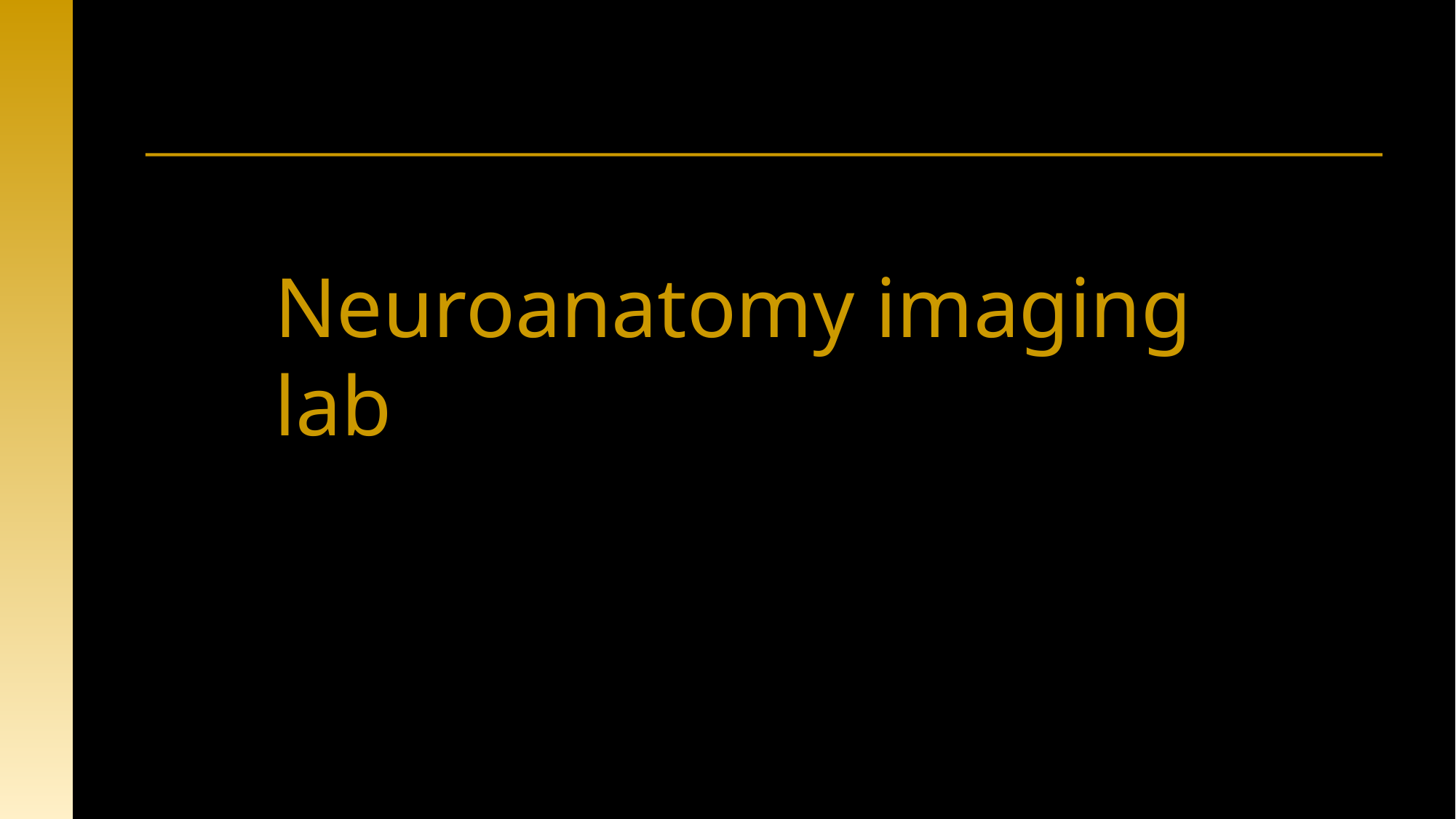

# Neuroanatomy imaging lab

## Slide 2
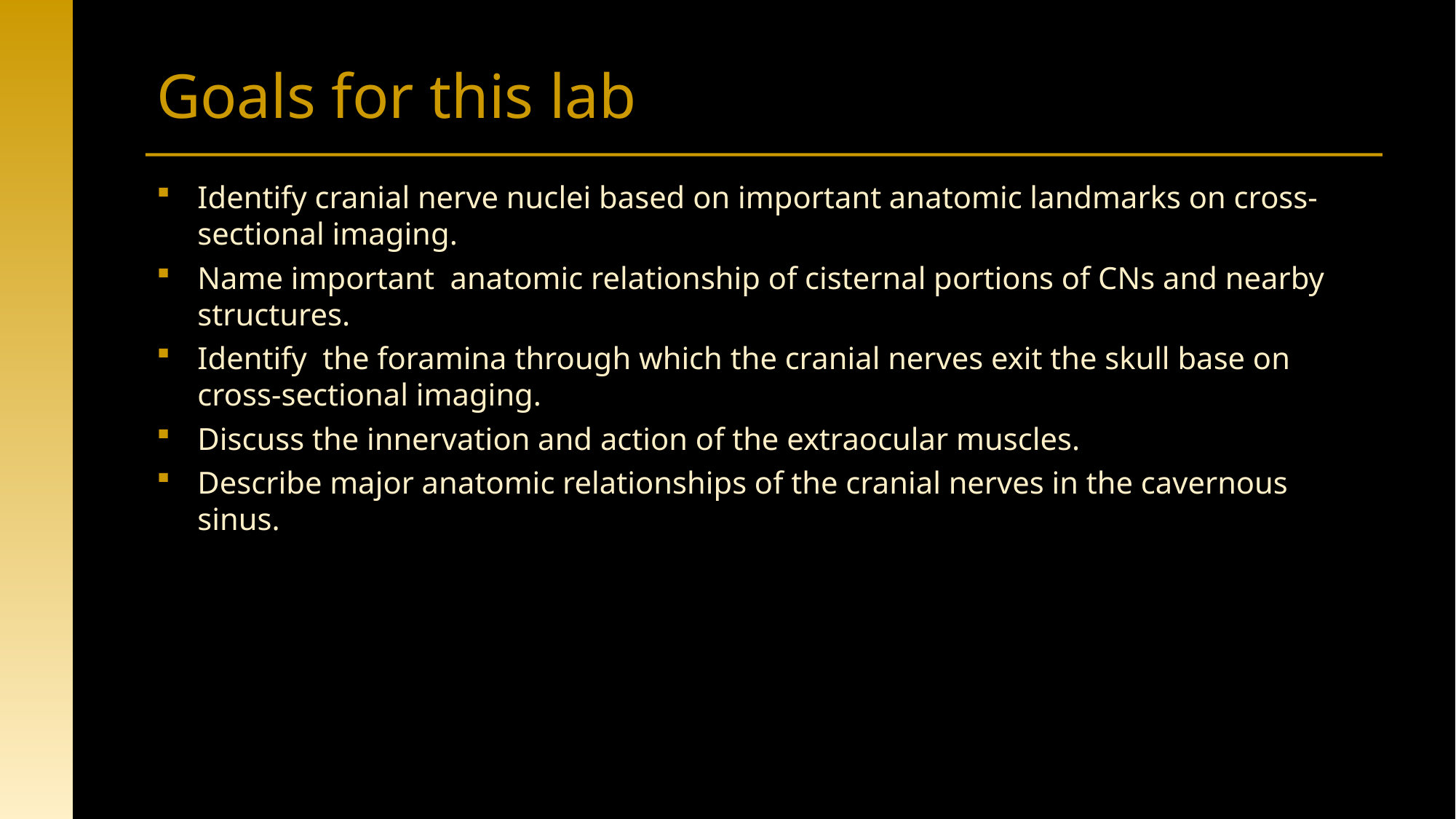

# Goals for this lab
Identify cranial nerve nuclei based on important anatomic landmarks on cross-sectional imaging.
Name important  anatomic relationship of cisternal portions of CNs and nearby structures.
Identify  the foramina through which the cranial nerves exit the skull base on cross-sectional imaging.
Discuss the innervation and action of the extraocular muscles. ​
Describe major anatomic relationships of the cranial nerves in the cavernous sinus.

## Slide 3
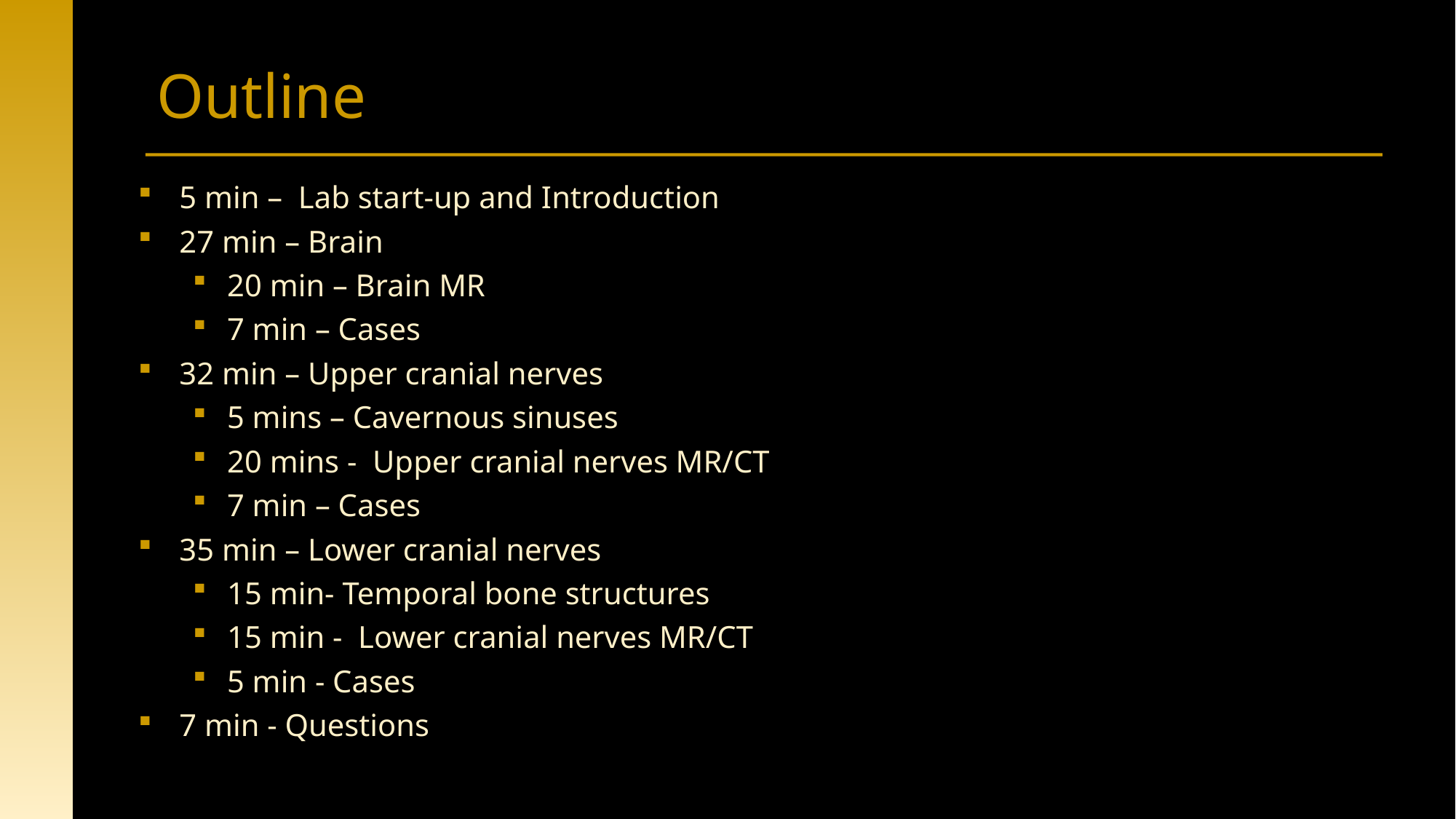

Outline
5 min – Lab start-up and Introduction
27 min – Brain
20 min – Brain MR
7 min – Cases
32 min – Upper cranial nerves
5 mins – Cavernous sinuses
20 mins - Upper cranial nerves MR/CT
7 min – Cases
35 min – Lower cranial nerves
15 min- Temporal bone structures
15 min - Lower cranial nerves MR/CT
5 min - Cases
7 min - Questions

## Slide 4
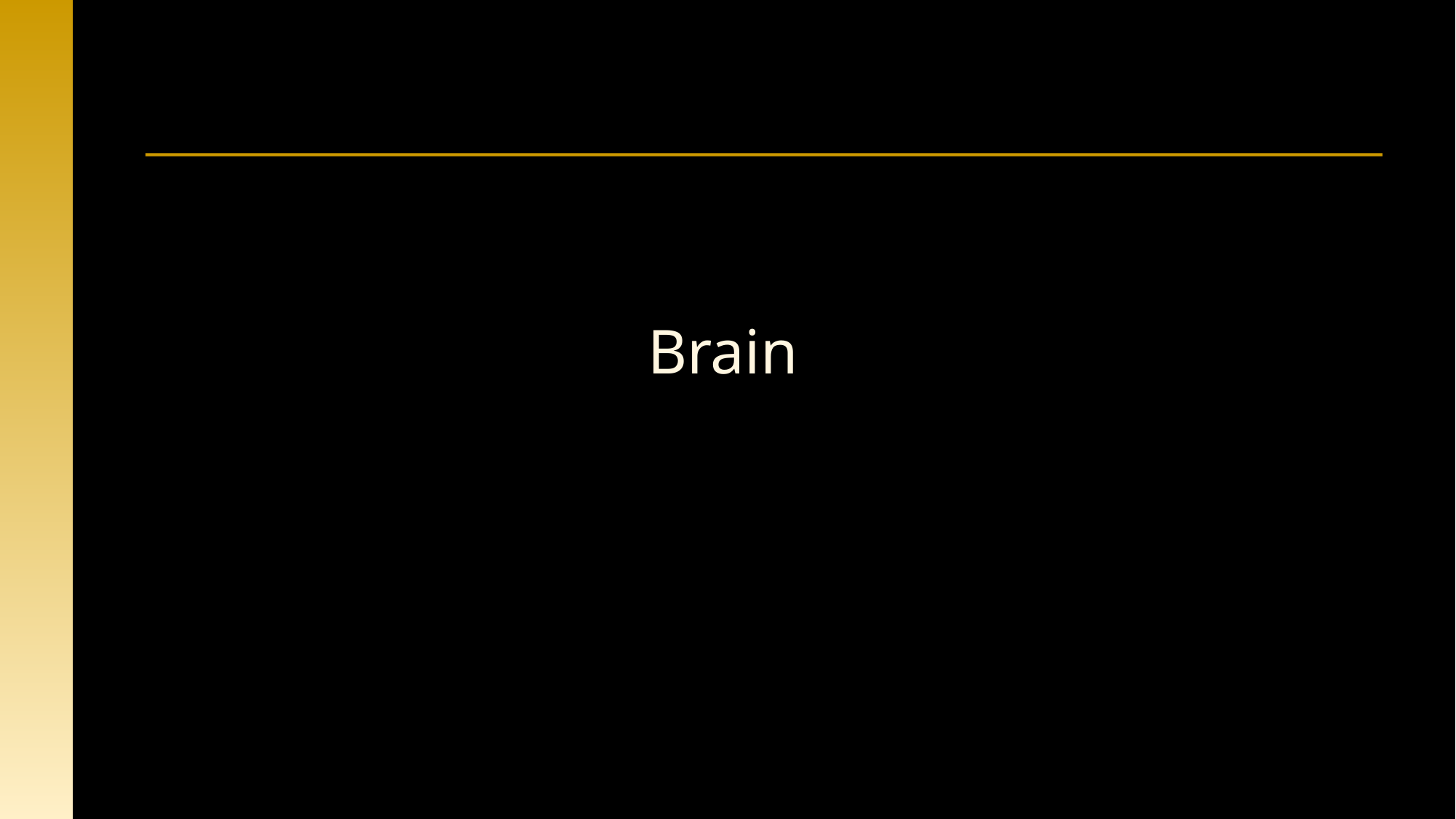

Brain

## Slide 5
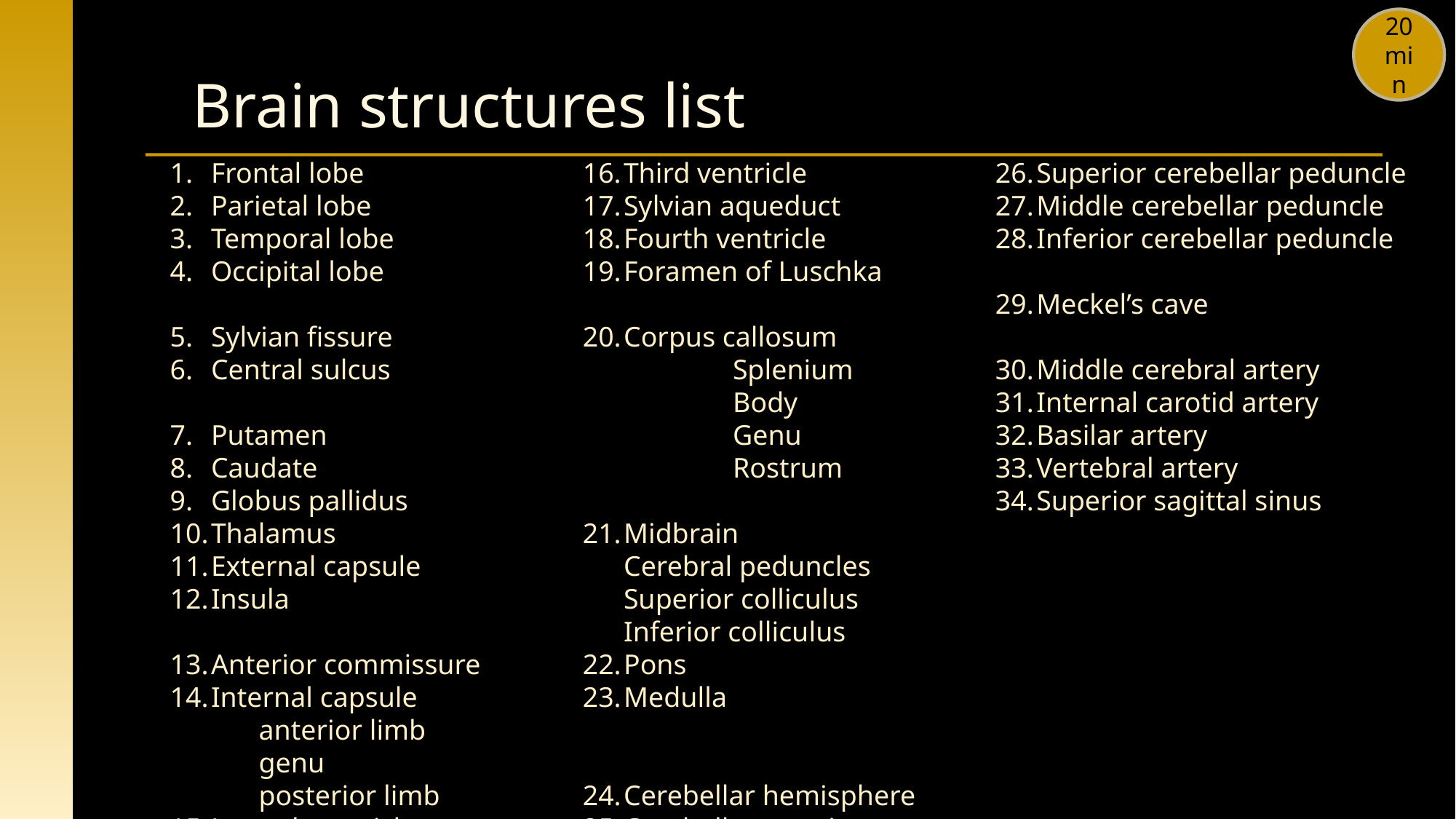

20 min
Brain structures list
Frontal lobe
Parietal lobe
Temporal lobe
Occipital lobe
Sylvian fissure
Central sulcus
Putamen
Caudate
Globus pallidus
Thalamus
External capsule
Insula
Anterior commissure
Internal capsule
anterior limb
genu
posterior limb
Lateral ventricles
Third ventricle
Sylvian aqueduct
Fourth ventricle
Foramen of Luschka
Corpus callosum
	Splenium
	Body
	Genu
	Rostrum
Midbrain
Cerebral peduncles
Superior colliculus
Inferior colliculus
Pons
Medulla
Cerebellar hemisphere
Cerebellar vermis
Superior cerebellar peduncle
Middle cerebellar peduncle
Inferior cerebellar peduncle
Meckel’s cave
Middle cerebral artery
Internal carotid artery
Basilar artery
Vertebral artery
Superior sagittal sinus

## Slide 6
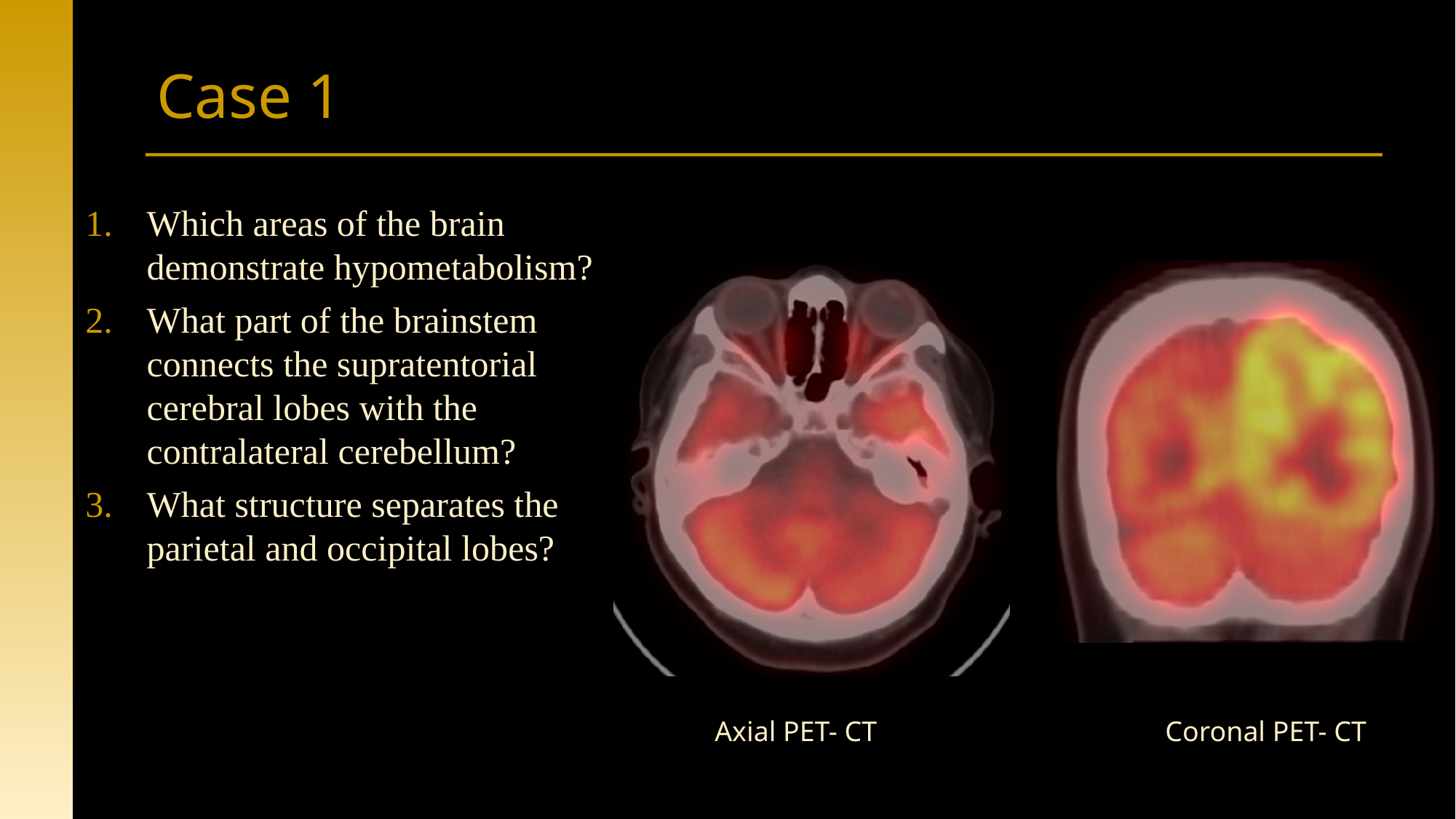

# Case 1
Which areas of the brain demonstrate hypometabolism?
What part of the brainstem connects the supratentorial cerebral lobes with the contralateral cerebellum?
What structure separates the parietal and occipital lobes?
Axial PET- CT
Coronal PET- CT

## Slide 7
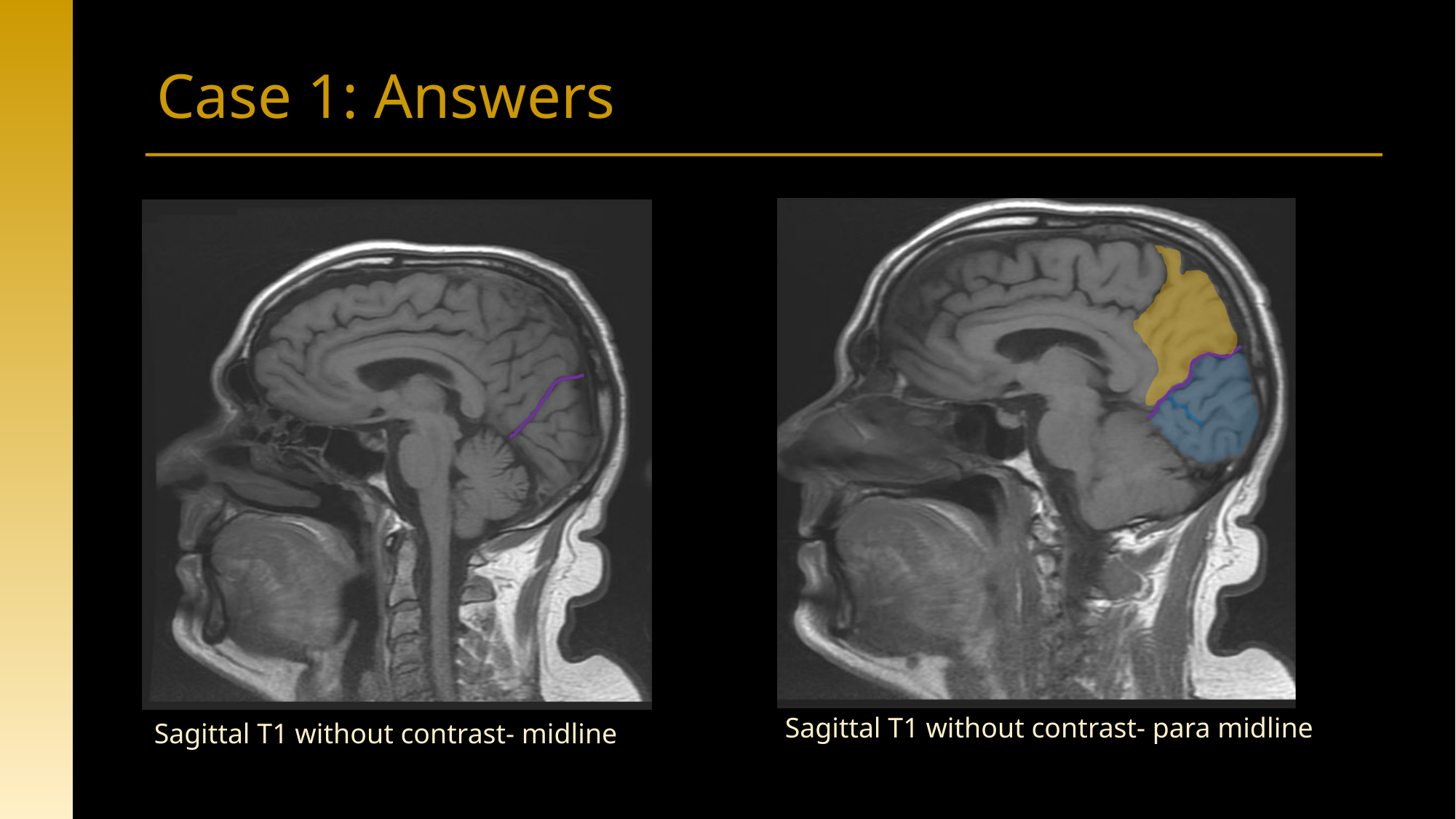

# Case 1: Answers
Sagittal T1 without contrast- para midline
Sagittal T1 without contrast- midline

## Slide 8
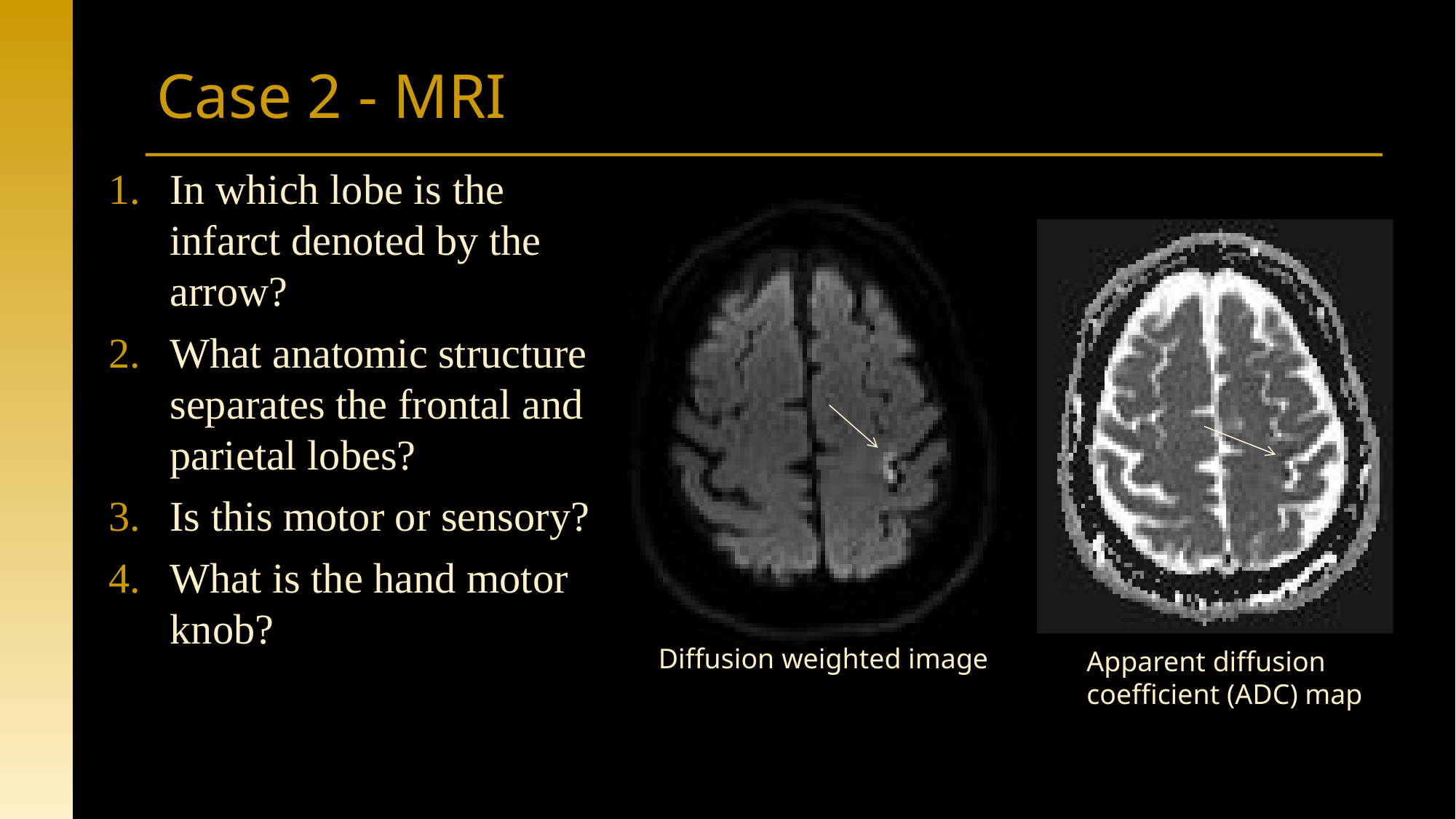

# Case 2 - MRI
In which lobe is the infarct denoted by the arrow?
What anatomic structure separates the frontal and parietal lobes?
Is this motor or sensory?
What is the hand motor knob?
Diffusion weighted image
Apparent diffusion
coefficient (ADC) map

## Slide 9
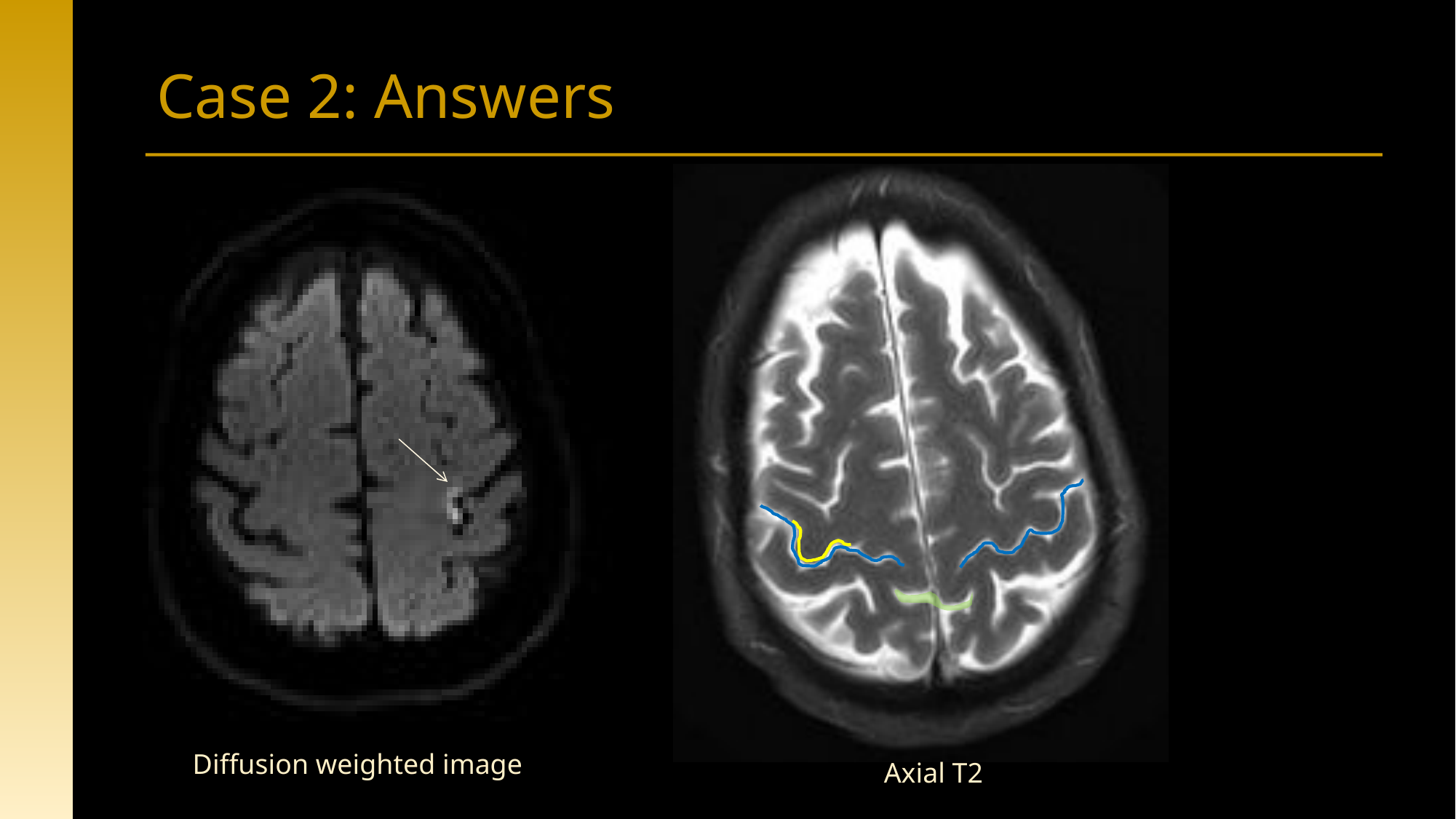

# Case 2: Answers
Diffusion weighted image
Axial T2

## Slide 10
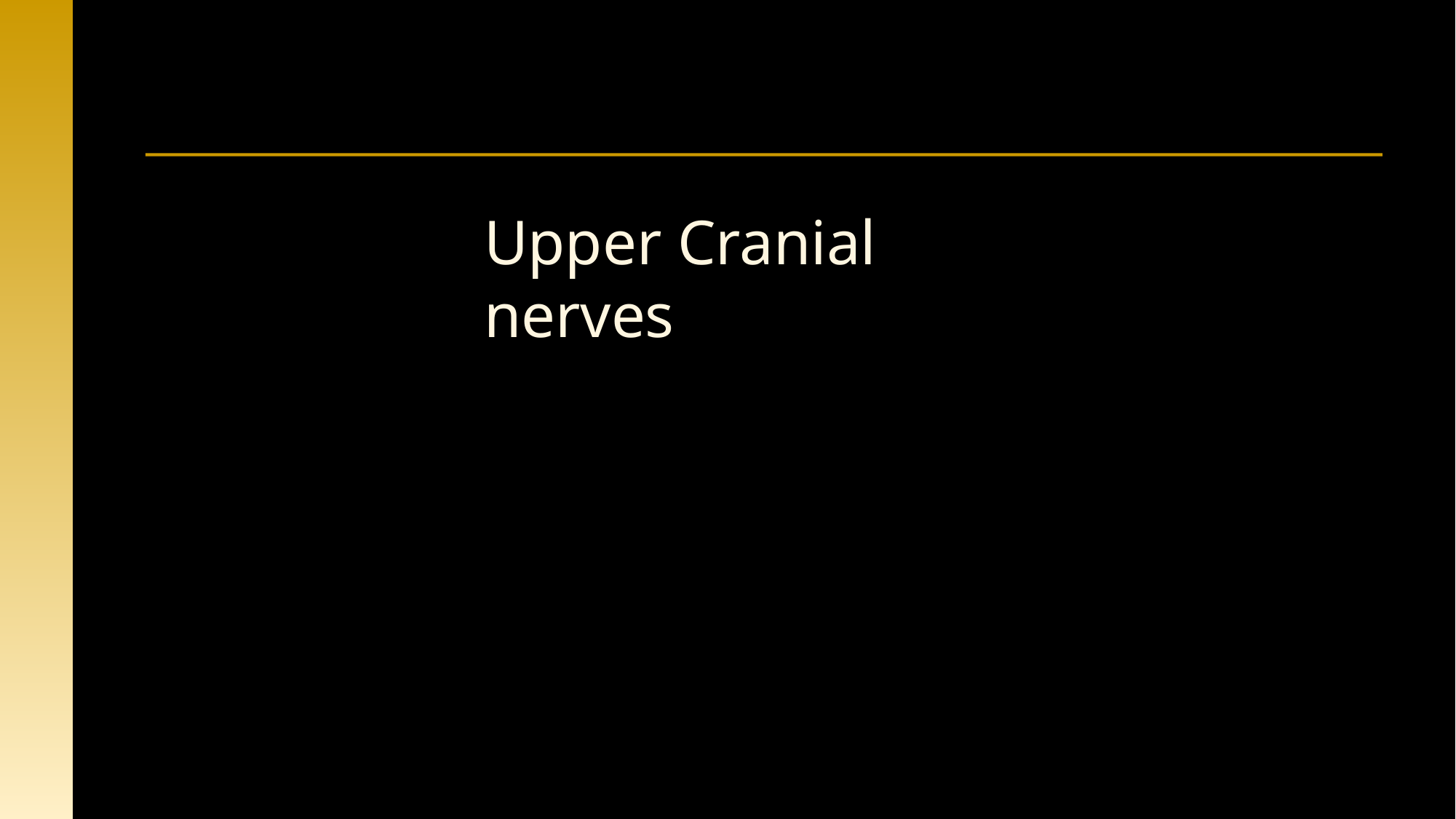

Upper Cranial nerves

## Slide 11
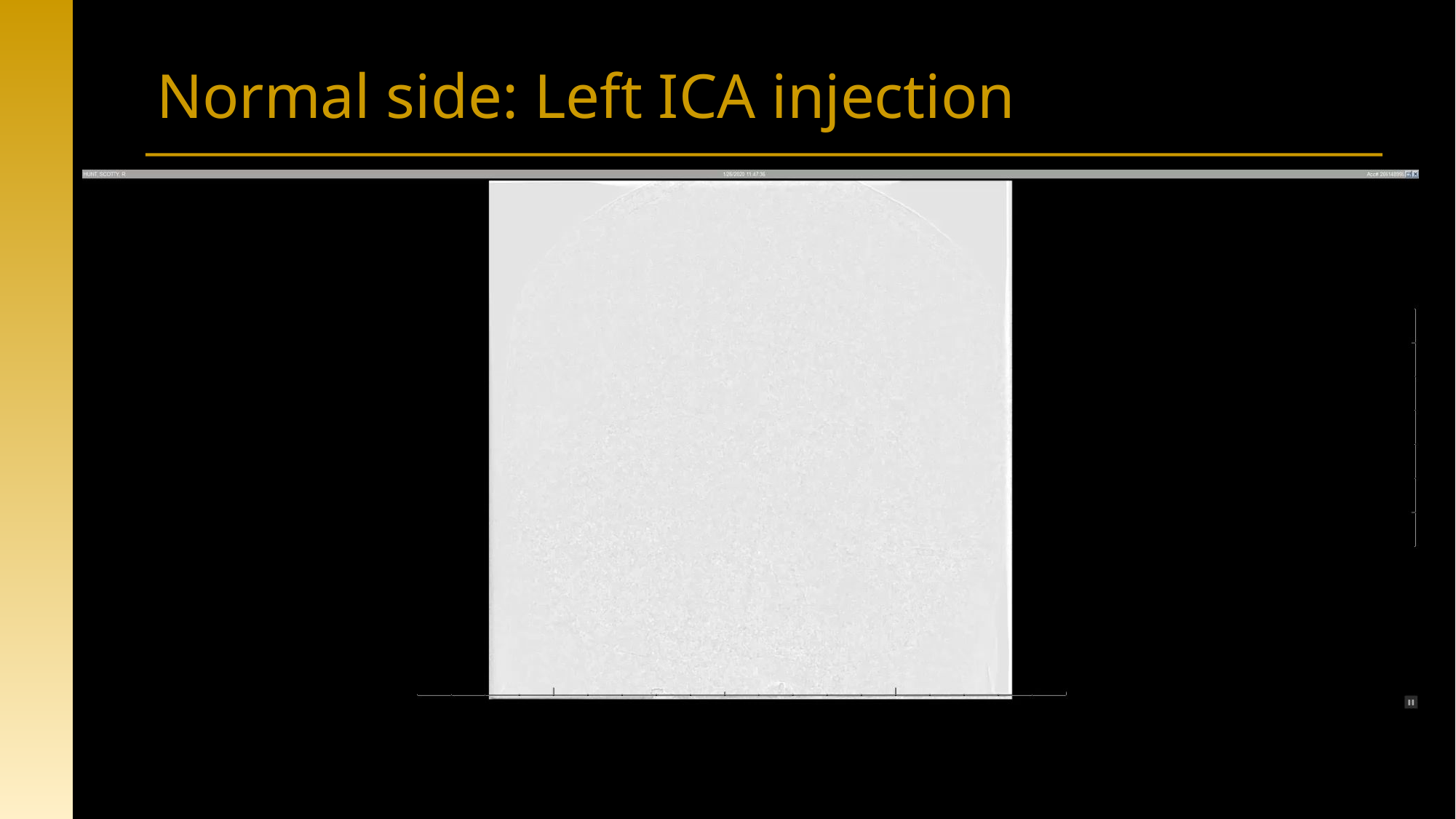

# Normal side: Left ICA injection

## Slide 12
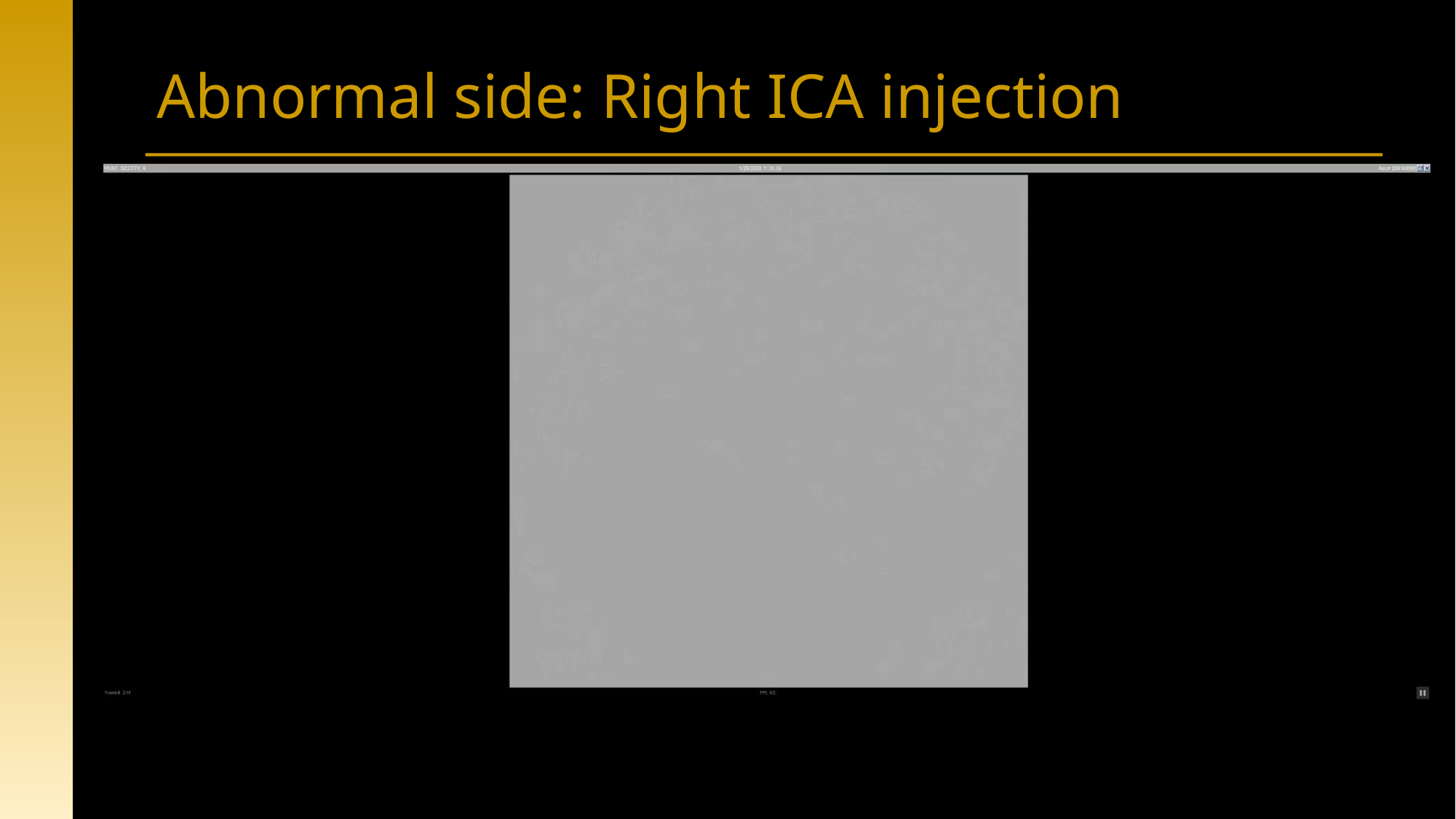

# Abnormal side: Right ICA injection

## Slide 13
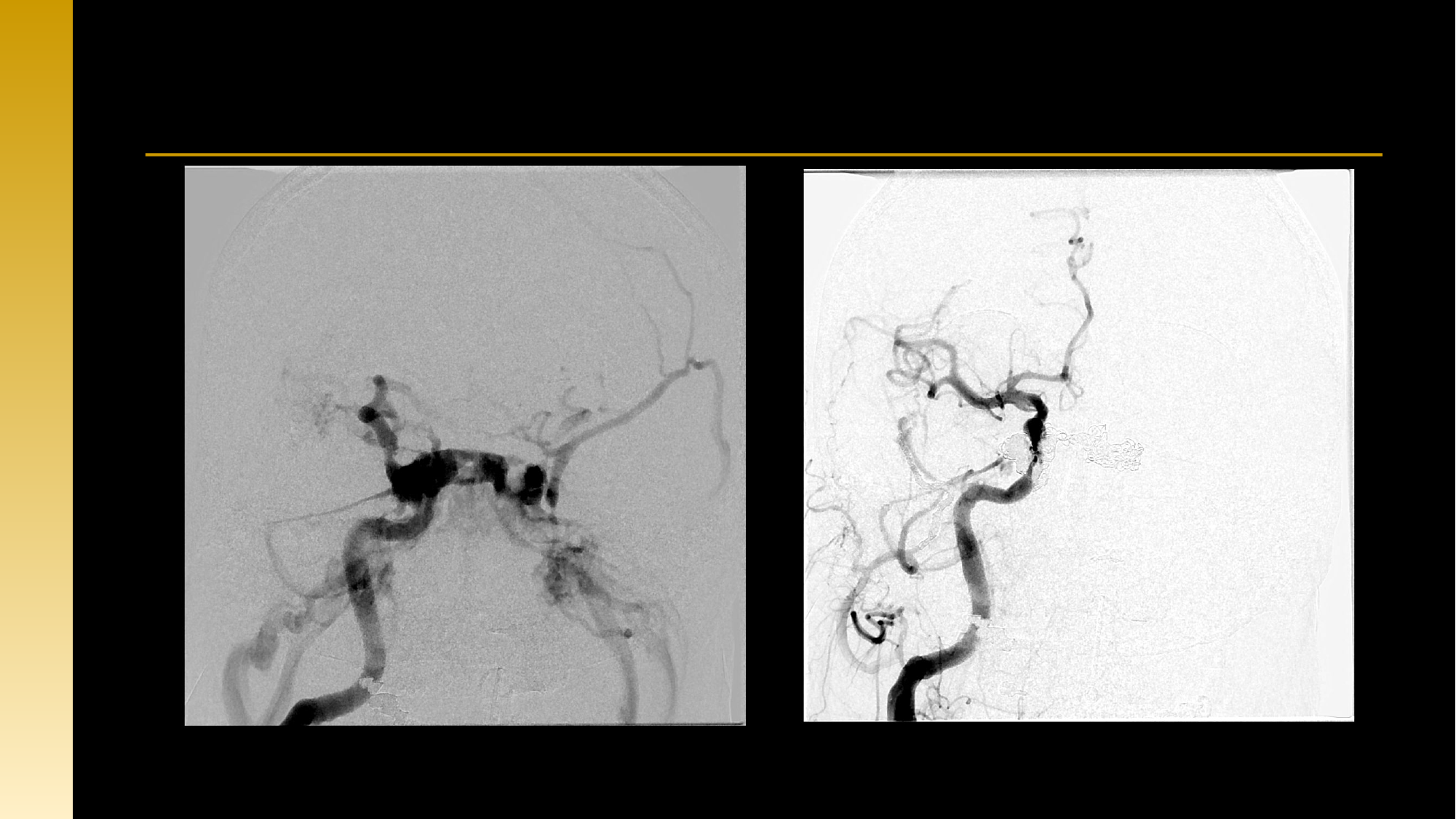

## Slide 14
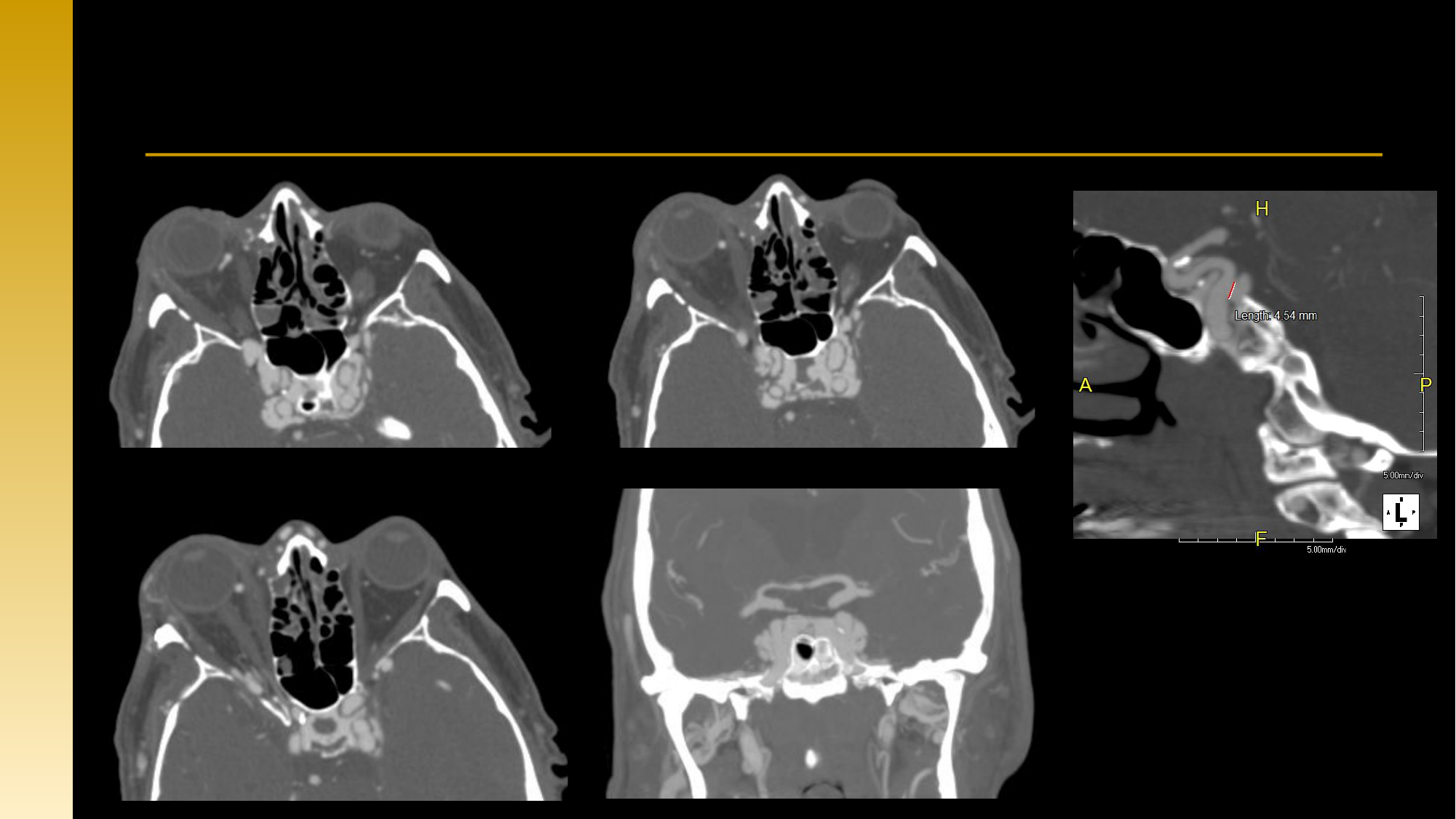

## Slide 15
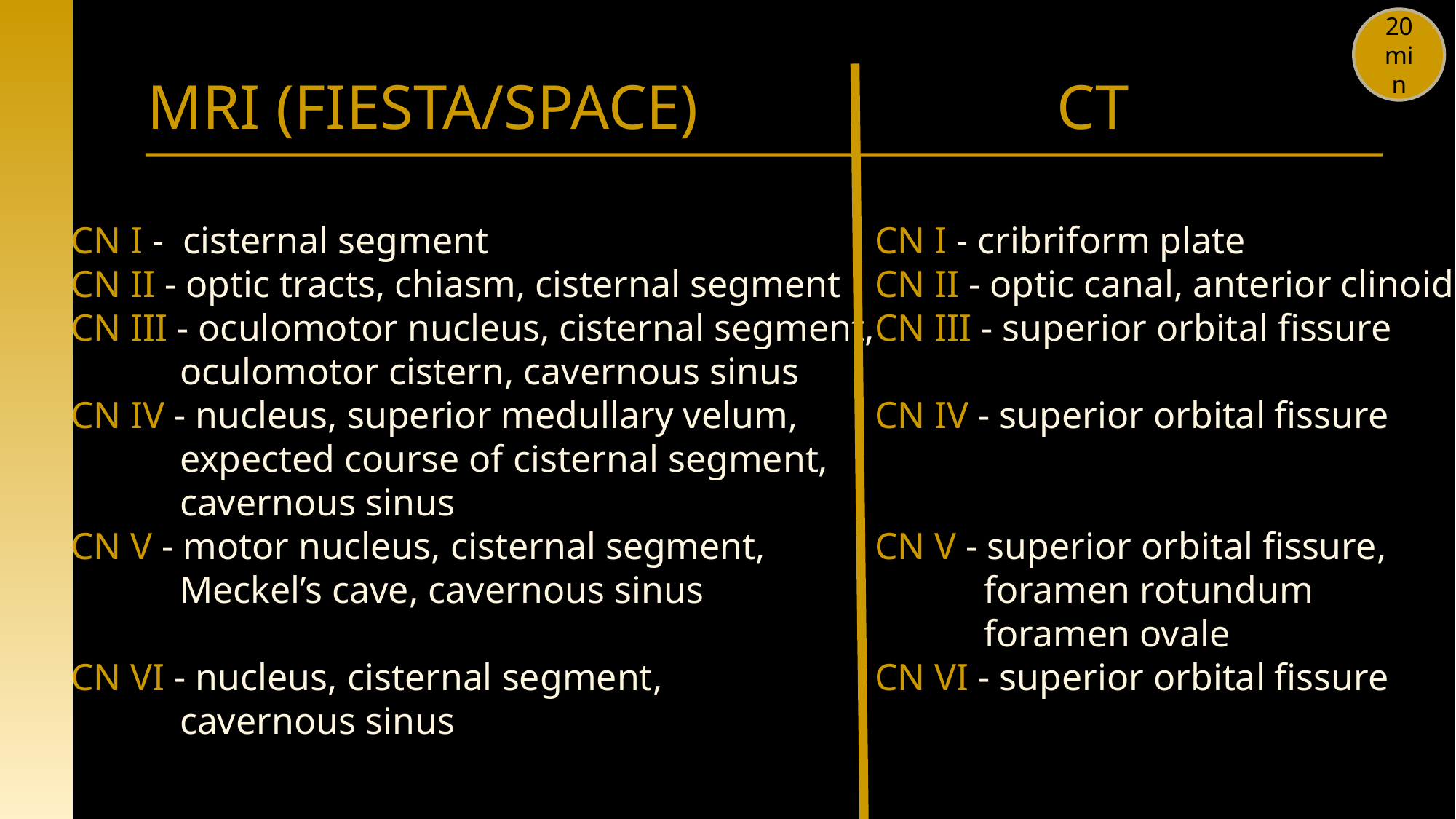

20 min
MRI (FIESTA/SPACE)
CT
CN I - cisternal segment
CN II - optic tracts, chiasm, cisternal segment
CN III - oculomotor nucleus, cisternal segment,
	oculomotor cistern, cavernous sinus
CN IV - nucleus, superior medullary velum,
	expected course of cisternal segment,
	cavernous sinus
CN V - motor nucleus, cisternal segment,
	Meckel’s cave, cavernous sinus
CN VI - nucleus, cisternal segment,
	cavernous sinus
CN I - cribriform plate
CN II - optic canal, anterior clinoid
CN III - superior orbital fissure
CN IV - superior orbital fissure
CN V - superior orbital fissure,
	foramen rotundum
	foramen ovale
CN VI - superior orbital fissure

## Slide 16
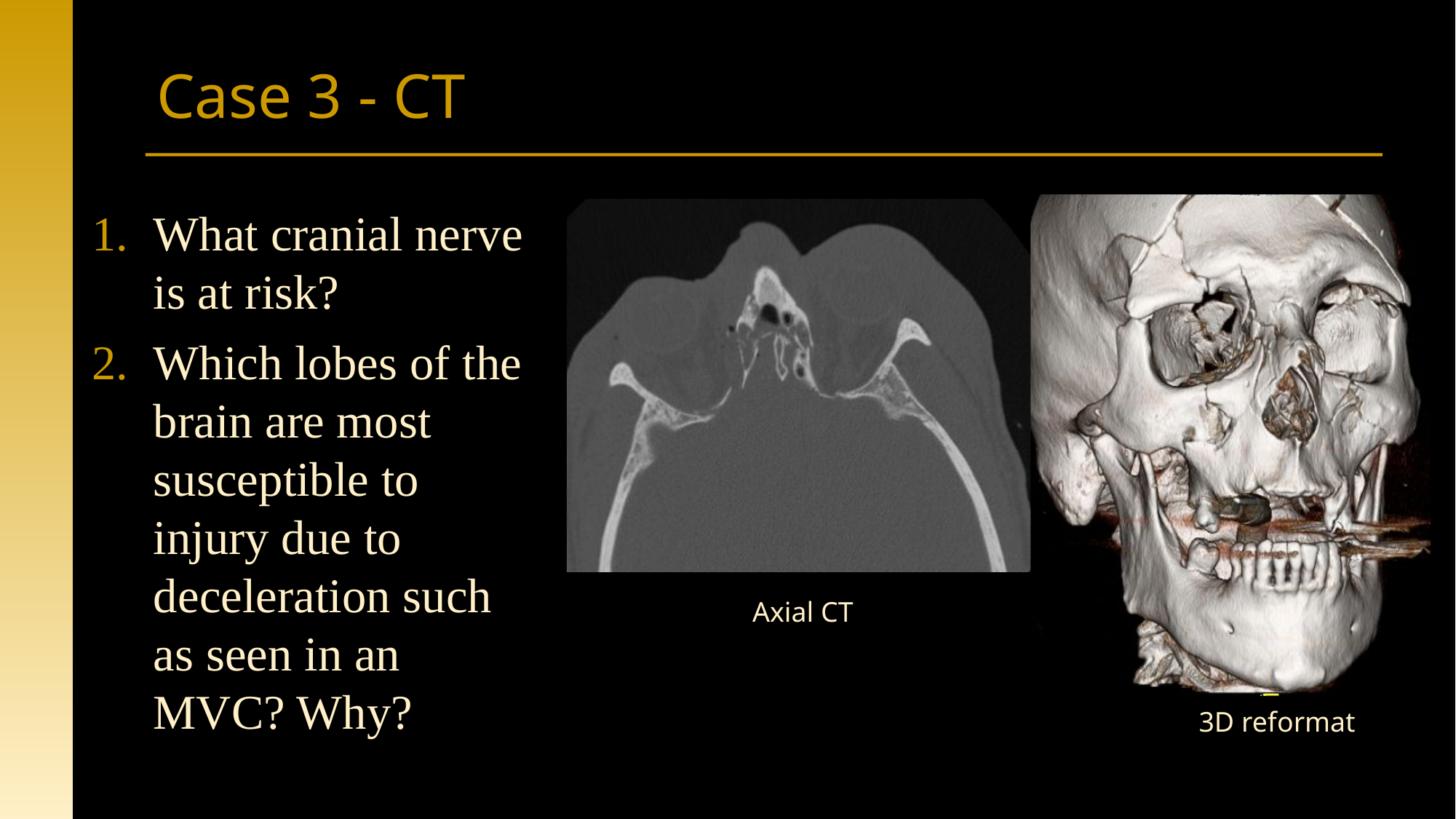

# Case 3 - CT
What cranial nerve is at risk?
Which lobes of the brain are most susceptible to injury due to deceleration such as seen in an MVC? Why?
Axial CT
3D reformat

## Slide 17
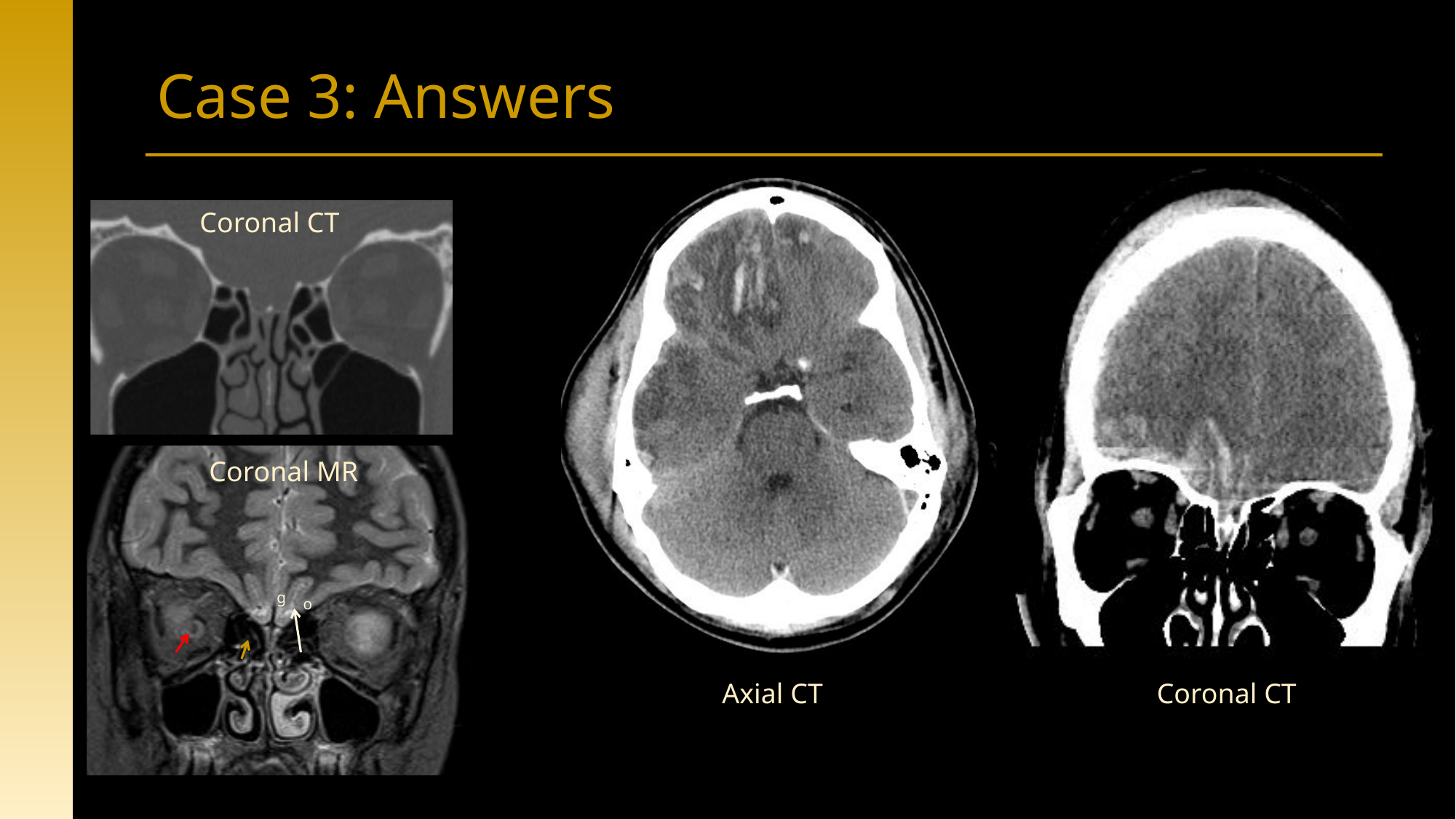

# Case 3: Answers
Coronal CT
g
o
Coronal MR
Axial CT
Coronal CT

## Slide 18
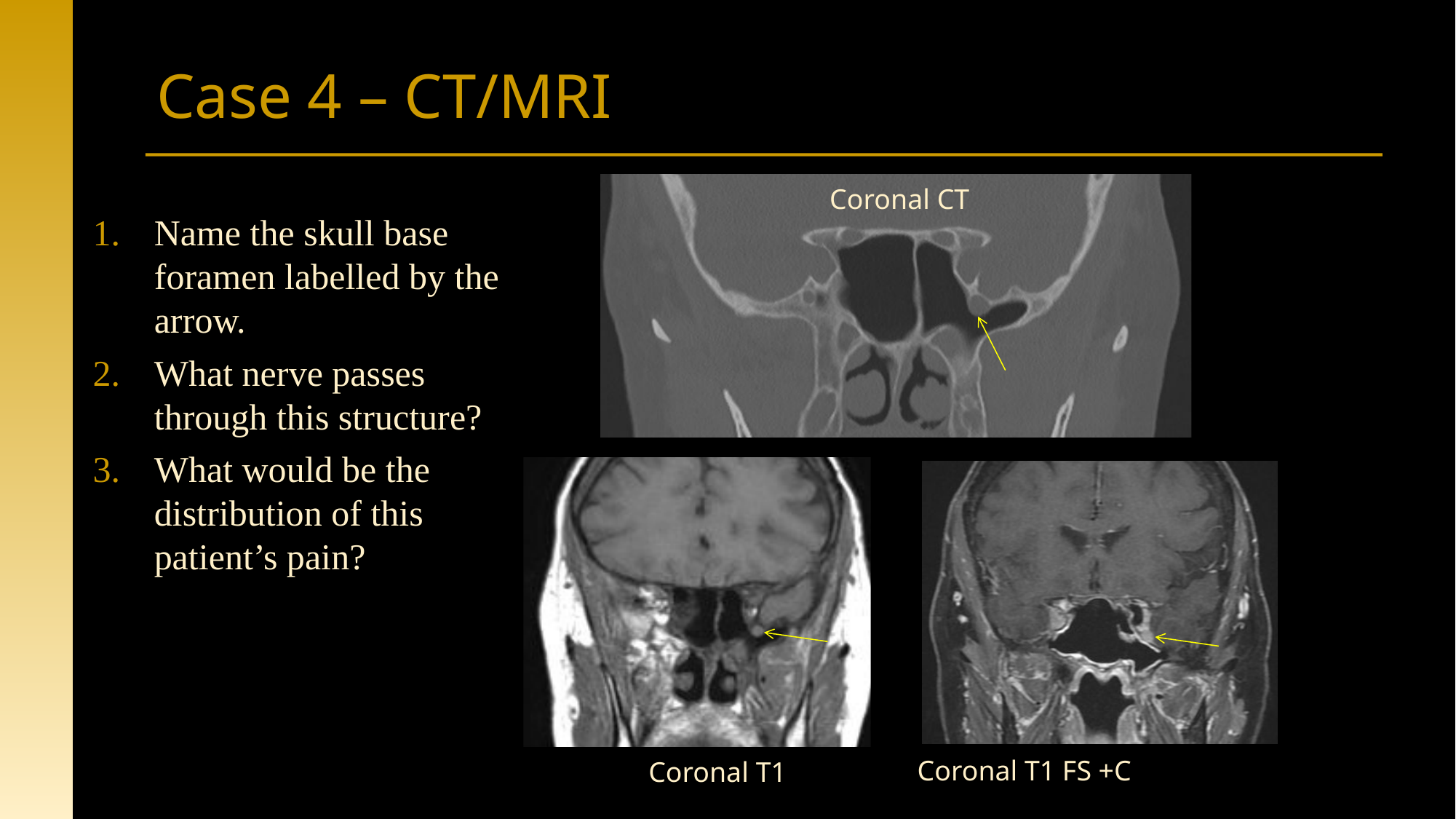

# Case 4 – CT/MRI
Coronal CT
Name the skull base foramen labelled by the arrow.
What nerve passes through this structure?
What would be the distribution of this patient’s pain?
Coronal T1 FS +C
Coronal T1

## Slide 19
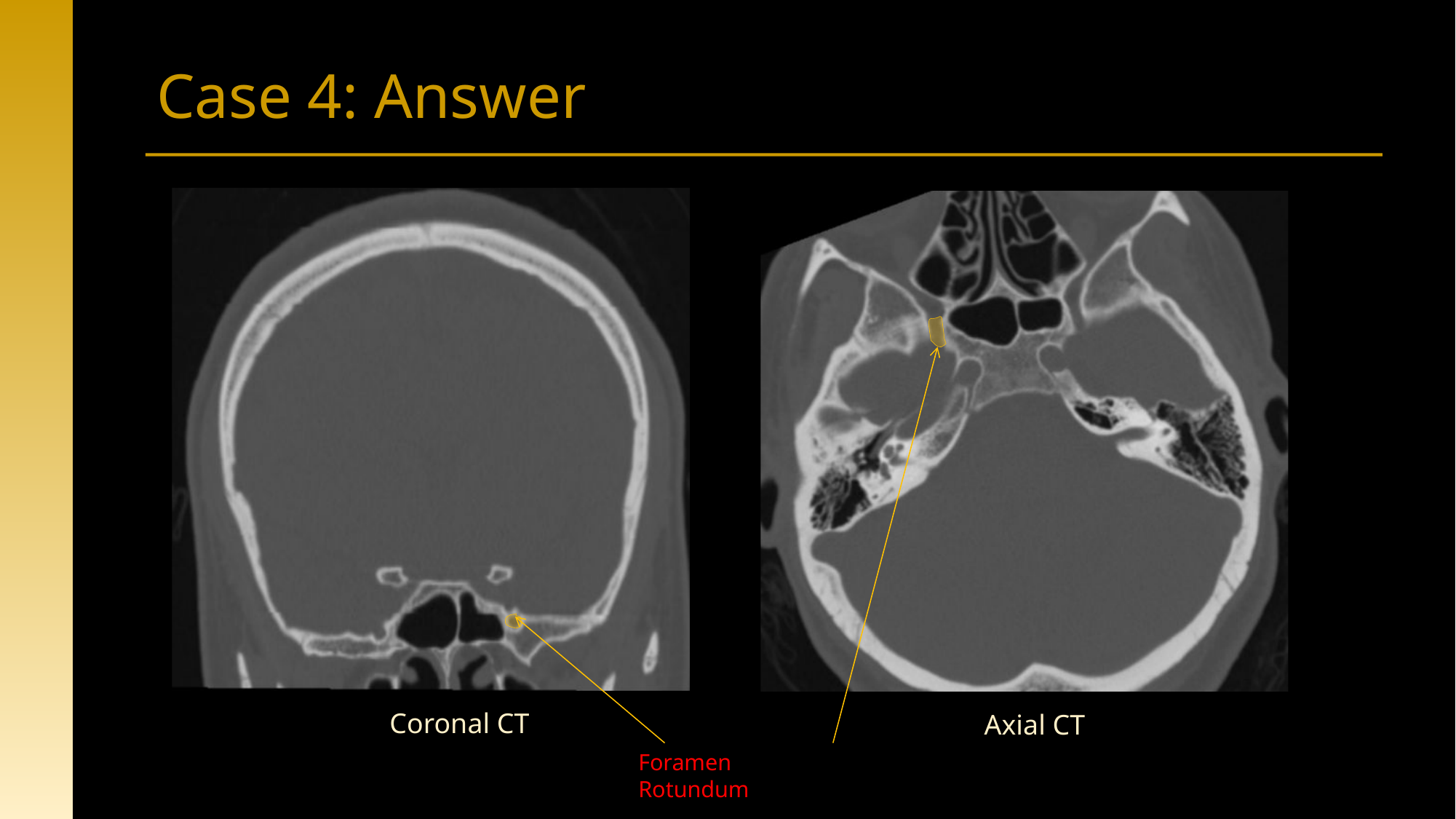

# Case 4: Answer
Coronal CT
Axial CT
Foramen Rotundum

## Slide 20
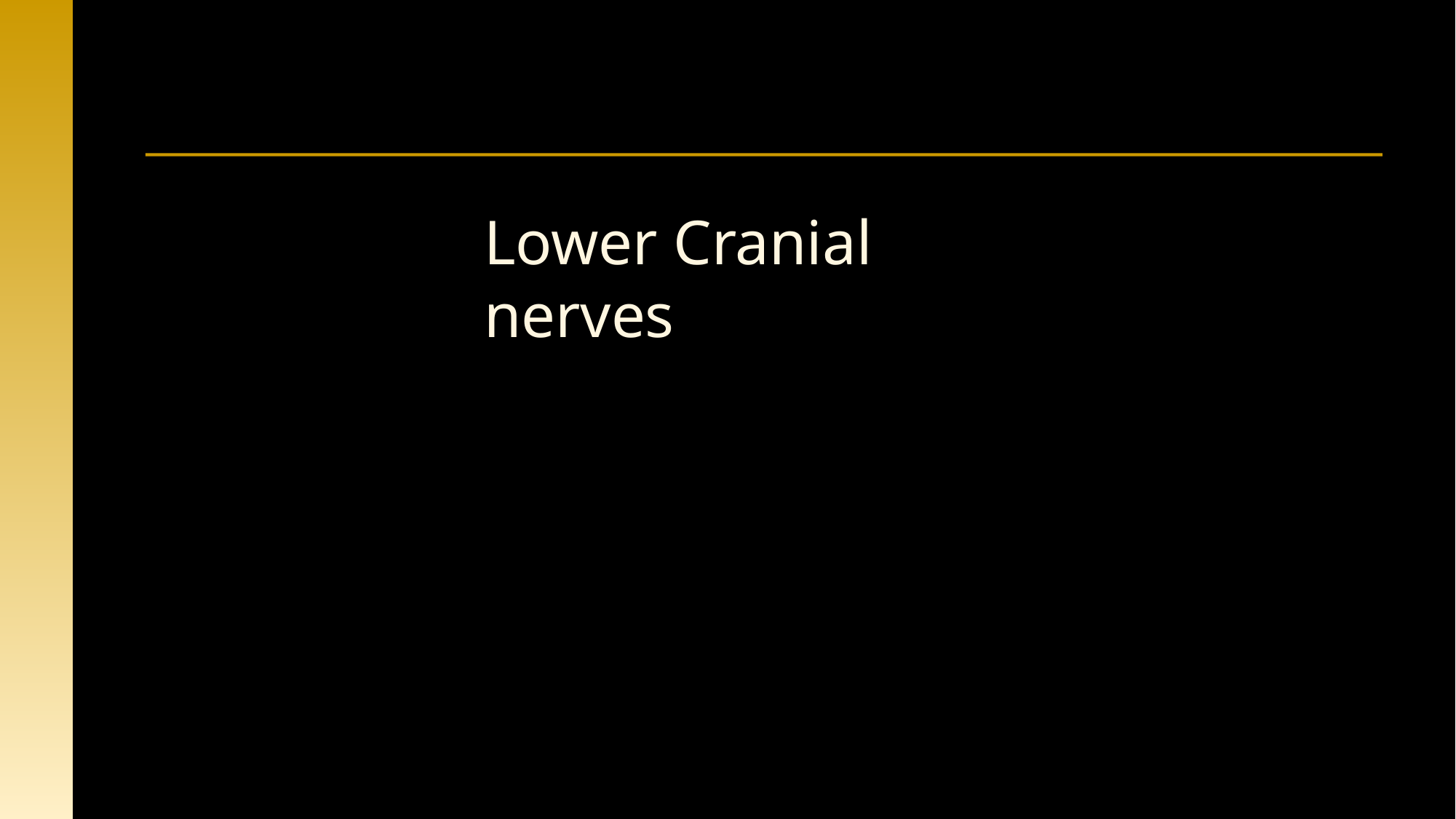

Lower Cranial nerves

## Slide 21
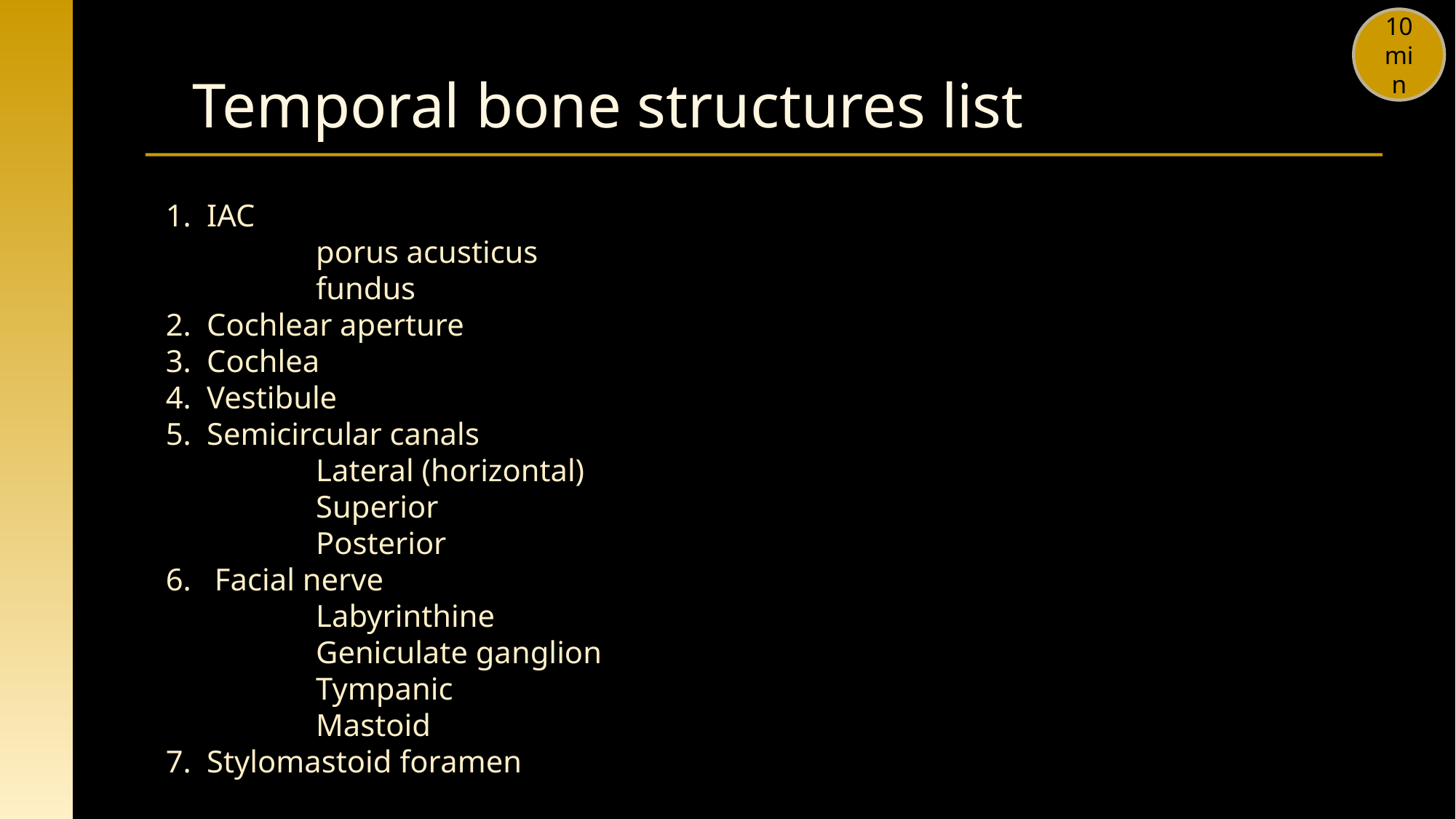

10 min
Temporal bone structures list
IAC
	porus acusticus
	fundus
Cochlear aperture
Cochlea
Vestibule
Semicircular canals
	Lateral (horizontal)
	Superior
	Posterior
 Facial nerve
	Labyrinthine
	Geniculate ganglion
	Tympanic
	Mastoid
Stylomastoid foramen

## Slide 22
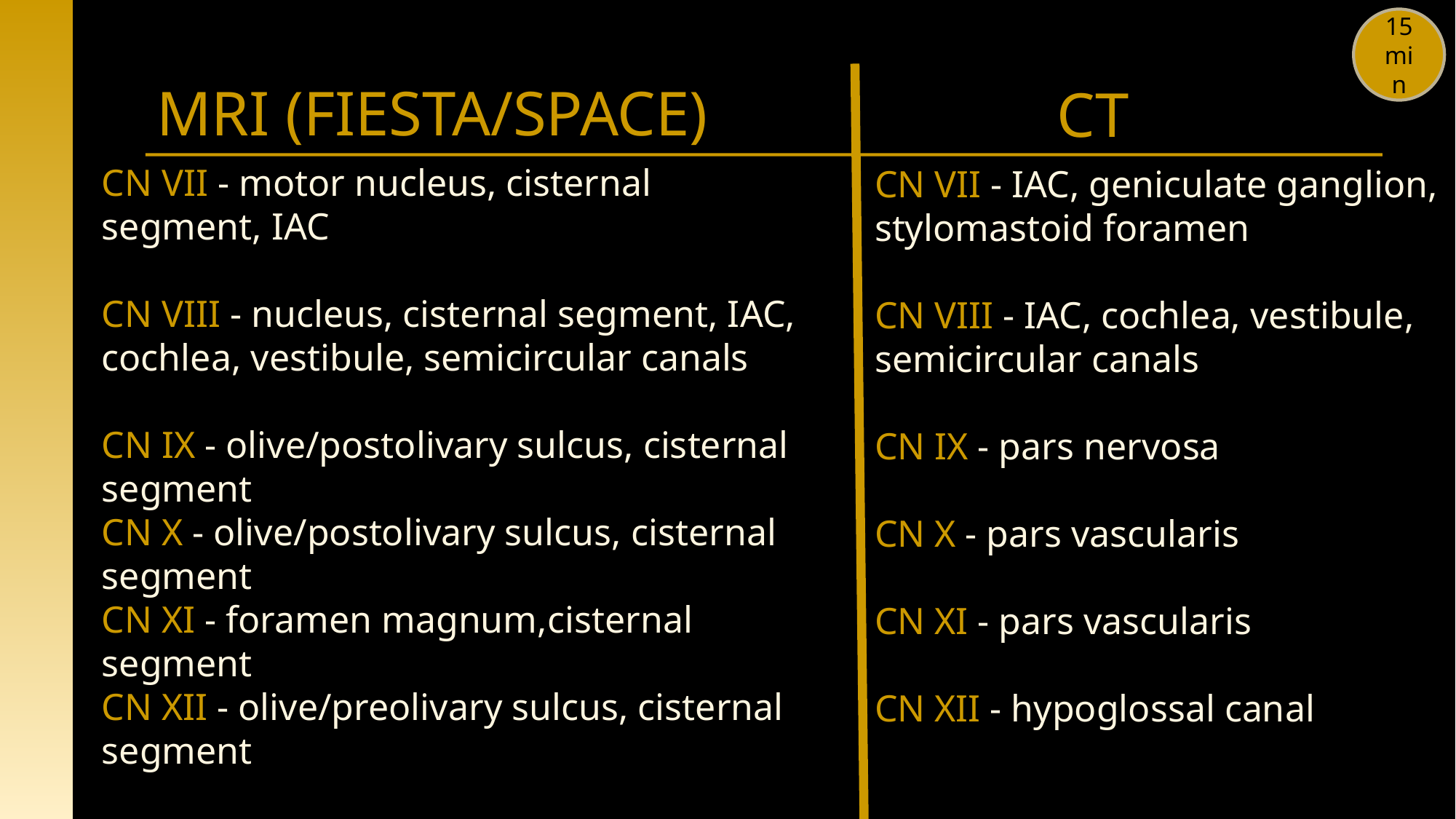

15 min
MRI (FIESTA/SPACE)
CT
CN VII - motor nucleus, cisternal segment, IAC
CN VIII - nucleus, cisternal segment, IAC, cochlea, vestibule, semicircular canals
CN IX - olive/postolivary sulcus, cisternal segment
CN X - olive/postolivary sulcus, cisternal segment
CN XI - foramen magnum,cisternal segment
CN XII - olive/preolivary sulcus, cisternal segment
CN VII - IAC, geniculate ganglion, stylomastoid foramen
CN VIII - IAC, cochlea, vestibule, semicircular canals
CN IX - pars nervosa
CN X - pars vascularis
CN XI - pars vascularis
CN XII - hypoglossal canal

## Slide 23
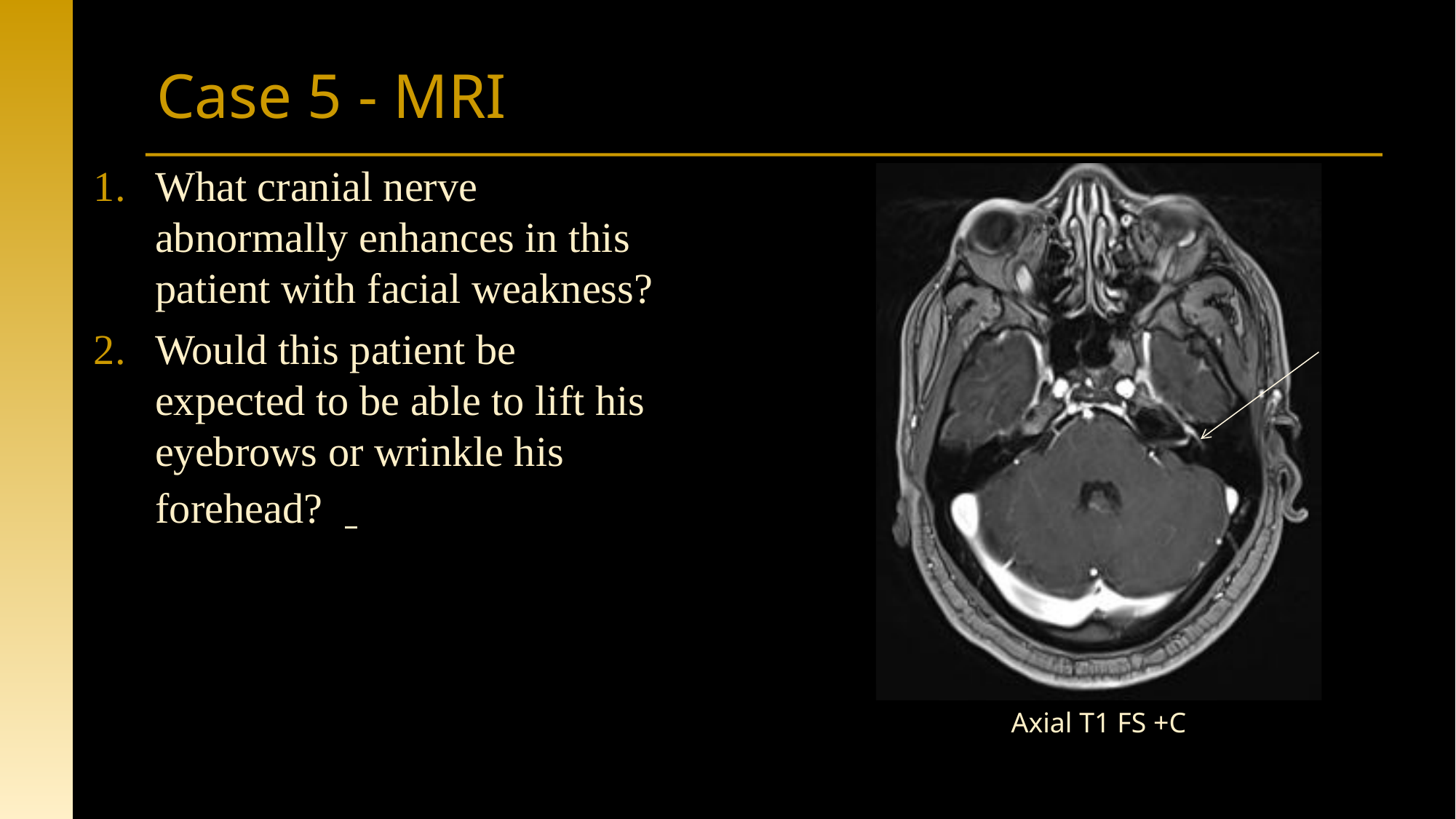

# Case 5 - MRI
What cranial nerve abnormally enhances in this patient with facial weakness?
Would this patient be expected to be able to lift his eyebrows or wrinkle his forehead?
Axial T1 FS +C

## Slide 24
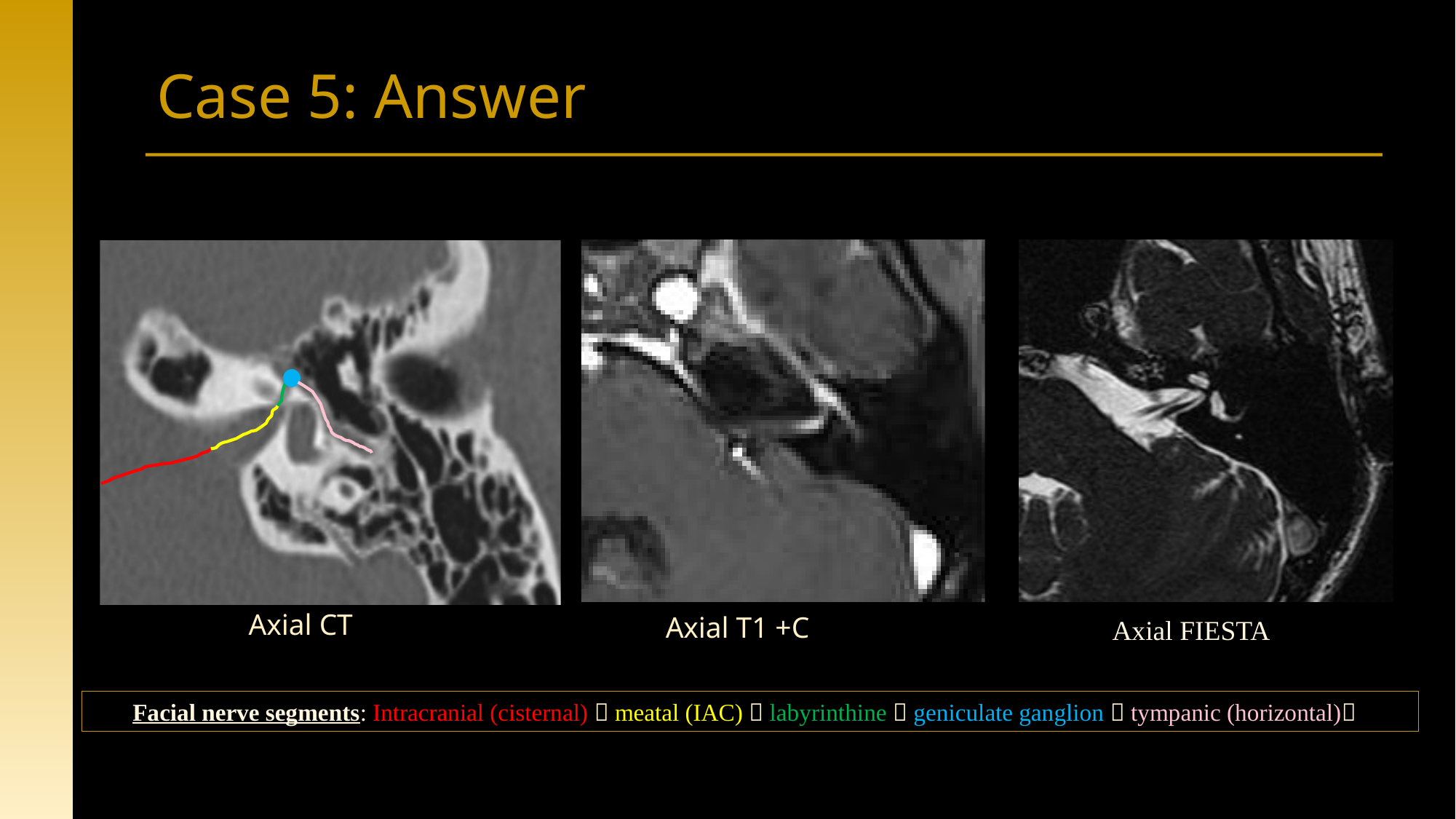

# Case 5: Answer
Axial CT
Axial T1 +C
Axial FIESTA
Facial nerve segments: Intracranial (cisternal)  meatal (IAC)  labyrinthine  geniculate ganglion  tympanic (horizontal)

## Slide 25
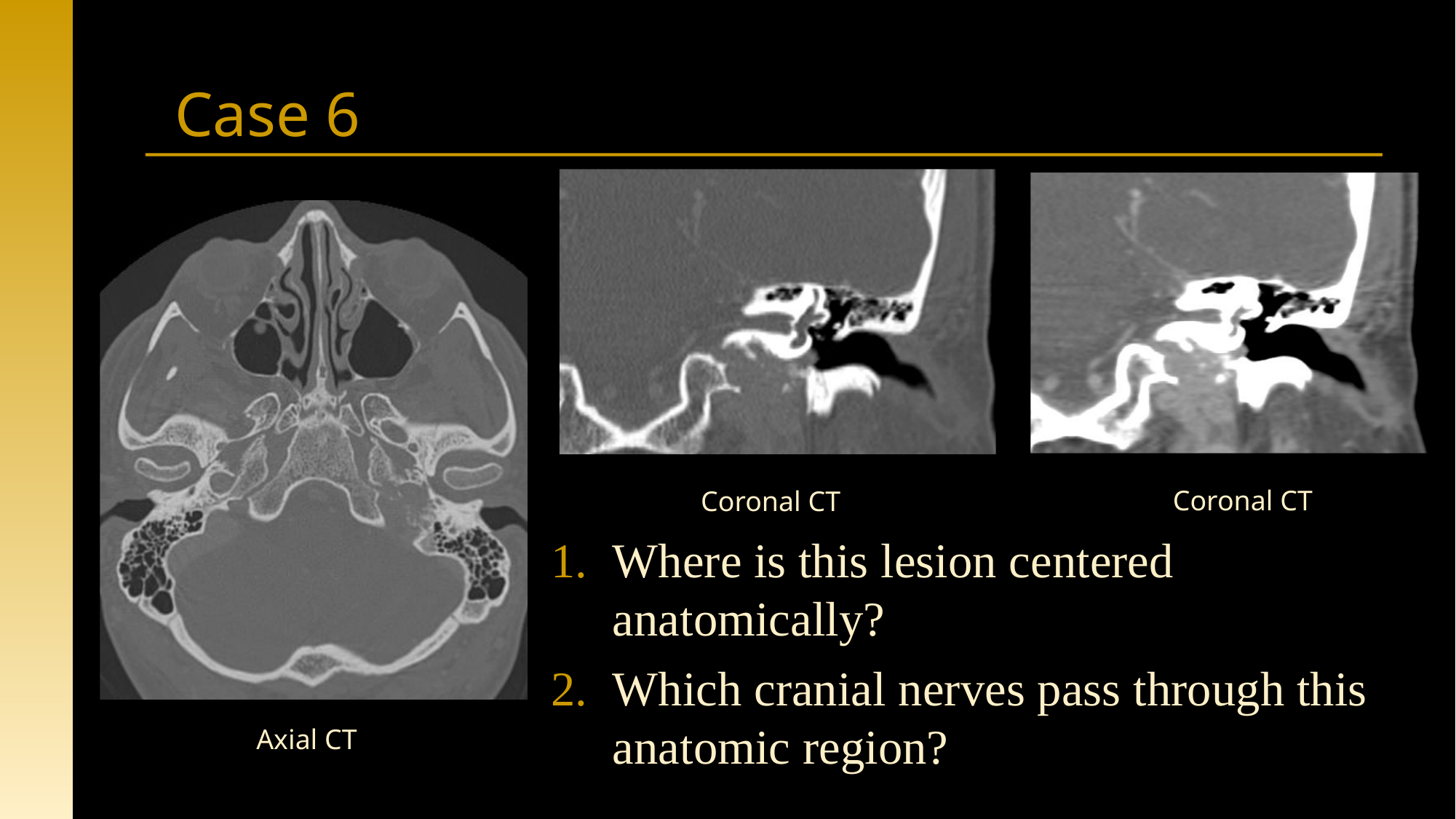

Case 6
Coronal CT
Coronal CT
Where is this lesion centered anatomically?
Which cranial nerves pass through this anatomic region?
Axial CT

## Slide 26
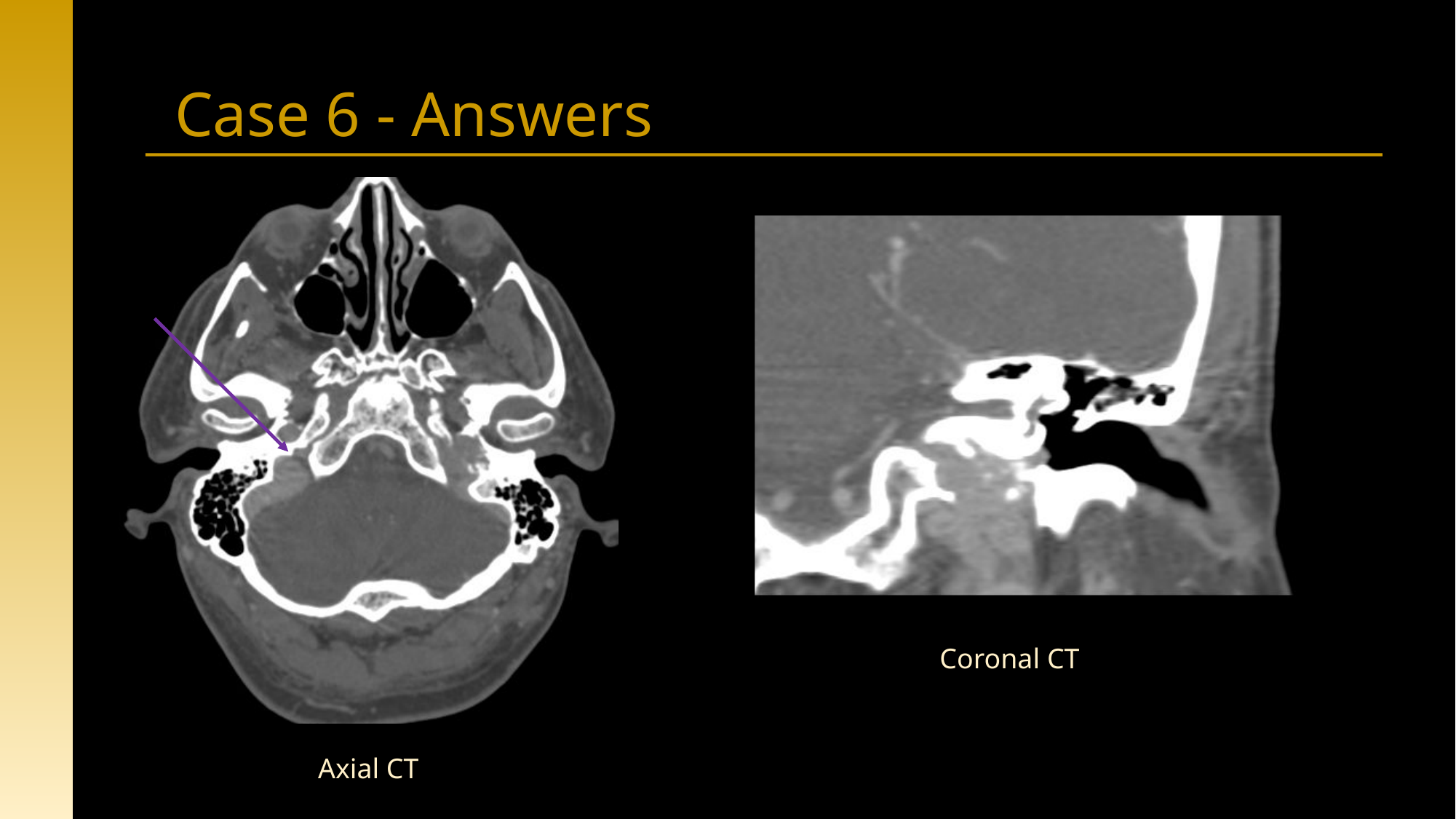

Case 6 - Answers
Coronal CT
Axial CT

## Slide 27
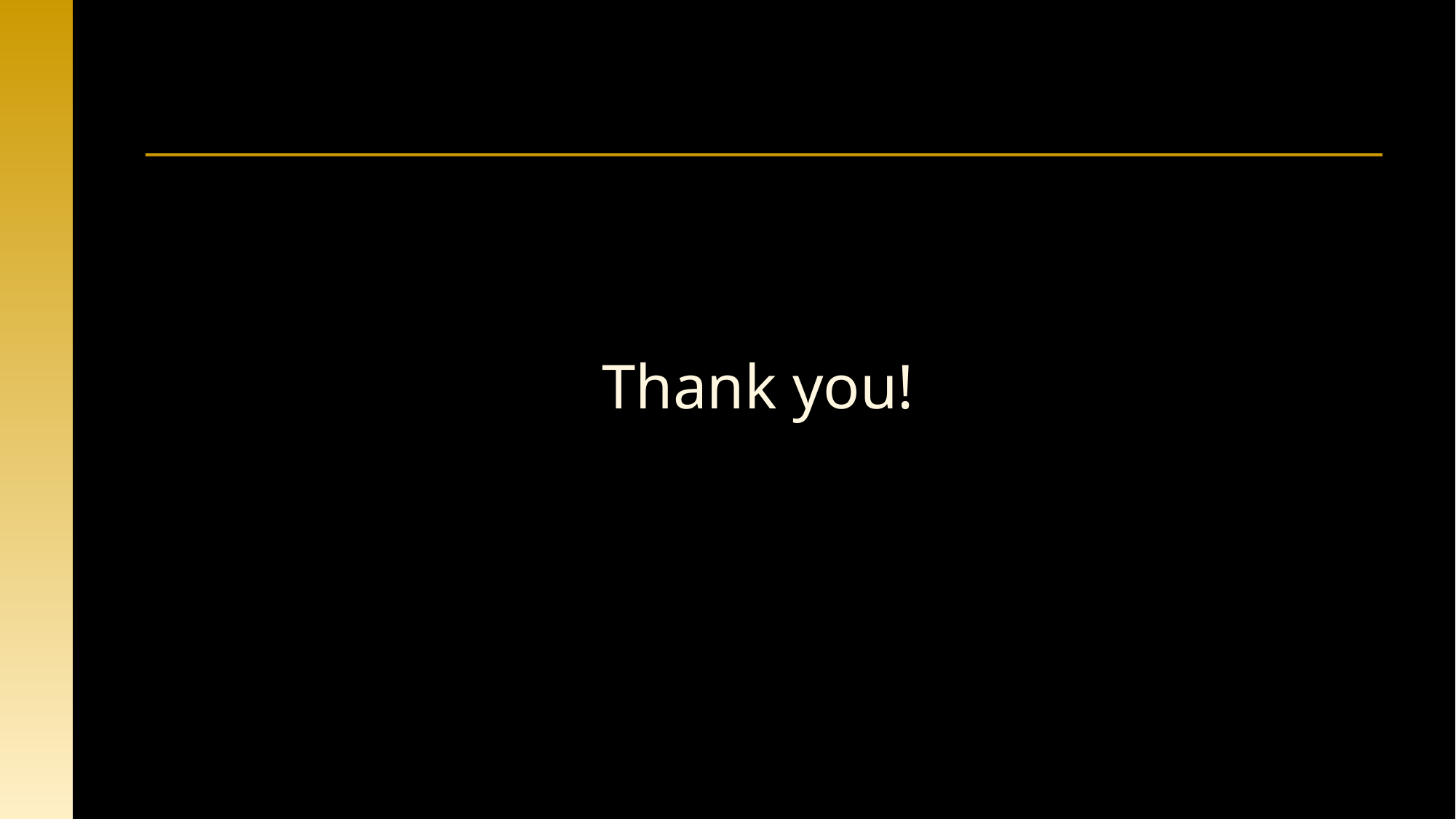

Thank you!
