# Supplementary material for: Cranial Nerve Anatomy Using a Modular and Multimodal Radiologic Approach
Source: MedEdPORTAL. 2022 Jun 10;18:11261. doi: 10.15766/mep_2374-8265.11261 (PMC9184306; doi:10.15766/mep_2374-8265.11261)
Supplement: Supplementary file 1 — Self-guided Anatomy Review.pptxCranial Nerve Video.mp4Cranial Nerve Lecture.pptxNeuroanatomy Lab.pptxNormal MRI and CT Scans - CT Bone Axials.pptxNormal MRI and CT Scans - T1 Sagittal.pptxNormal MRI and CT Scans - T2 Axial.pptxNormal MRI and CT Scans - T2 SPACE Axial.pptxPre- and Posttest.pptxSatisfaction Survey.docxAppendix Guide.docx [file mep_2374-8265.11261-s001.zip › J. Satisfaction Survey.docx]

Questions for students participating in Cranial Nerve Lecture and Lab

I am more confident in my knowledge of the anatomy of the cranial nerves after completing the radiology cranial nerve lab.

Strongly agree

Agree

Neither agree nor disagree

Disagree

Strongly disagree

I would recommend the radiology cranial nerve lecture, prelab and lab to other medical students.

Strongly agree

Agree

Neither agree nor disagree

Disagree

Strongly disagree

The structure of the radiology cranial nerve lecture, prelab and lab was interesting and engaging.

Strongly agree

Agree

Neither agree nor disagree

Disagree

Strongly disagree

How would you rate the quality of the materials utilized for the radiology cranial nerve lecture, prelab and lab?

Excellent

Good

Fair

Poor

Very poor
