# Supplementary material for: Cranial Nerve Anatomy Using a Modular and Multimodal Radiologic Approach
Source: MedEdPORTAL. 2022 Jun 10;18:11261. doi: 10.15766/mep_2374-8265.11261 (PMC9184306; doi:10.15766/mep_2374-8265.11261)
Supplement: Supplementary file 1 — Self-guided Anatomy Review.pptxCranial Nerve Video.mp4Cranial Nerve Lecture.pptxNeuroanatomy Lab.pptxNormal MRI and CT Scans - CT Bone Axials.pptxNormal MRI and CT Scans - T1 Sagittal.pptxNormal MRI and CT Scans - T2 Axial.pptxNormal MRI and CT Scans - T2 SPACE Axial.pptxPre- and Posttest.pptxSatisfaction Survey.docxAppendix Guide.docx [file mep_2374-8265.11261-s001.zip › I. Pre- and Posttest.pptx]

## Slide 1
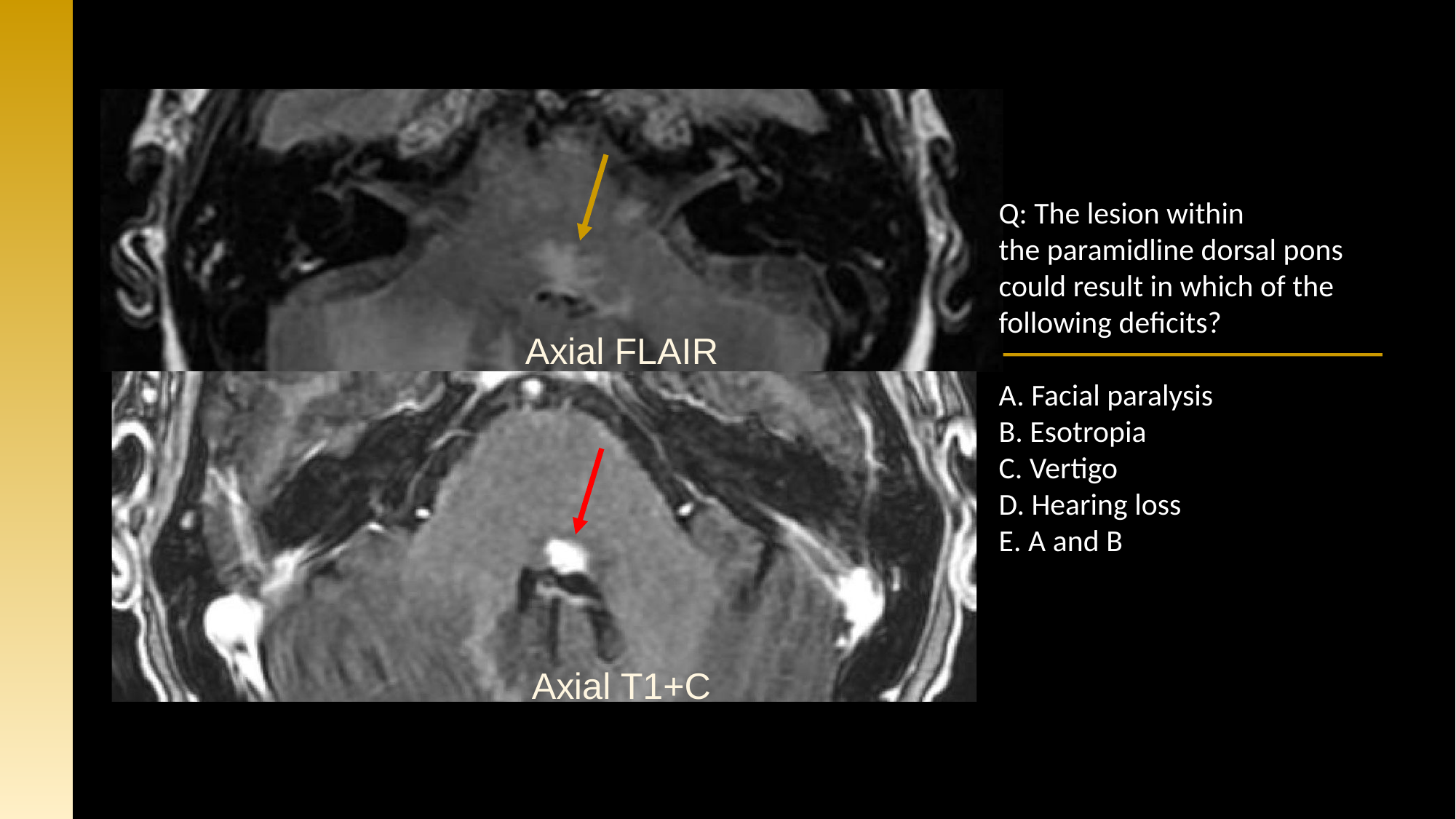

Q: The lesion within the paramidline dorsal pons could result in which of the following deficits?​
​
A. Facial paralysis
B. Esotropia
C. Vertigo
D. Hearing loss
E. A and B

## Slide 2
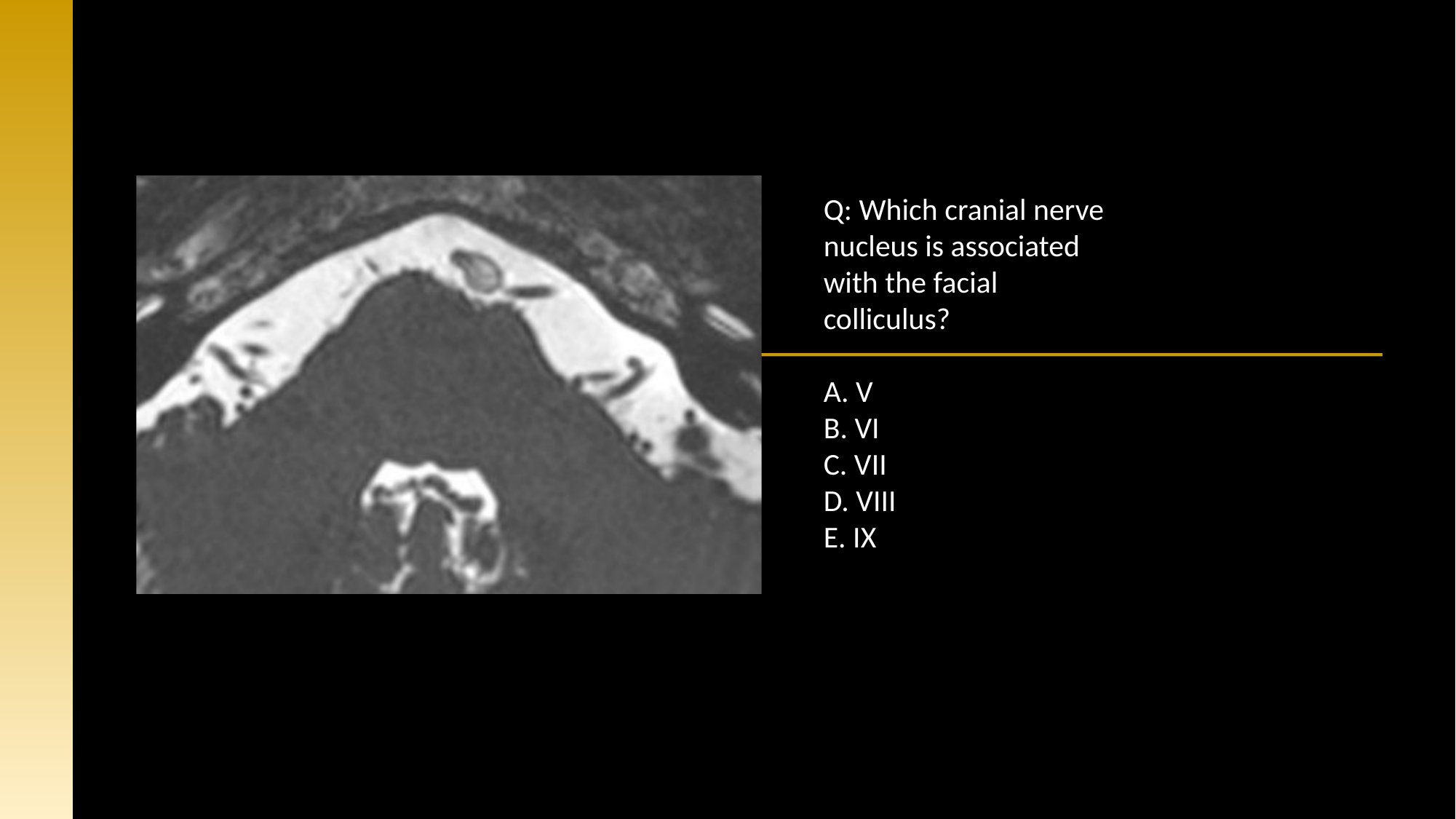

Q: Which cranial nerve nucleus is associated with the facial colliculus?​
A. V
B. VI
C. VII
D. VIII
E. IX

## Slide 3
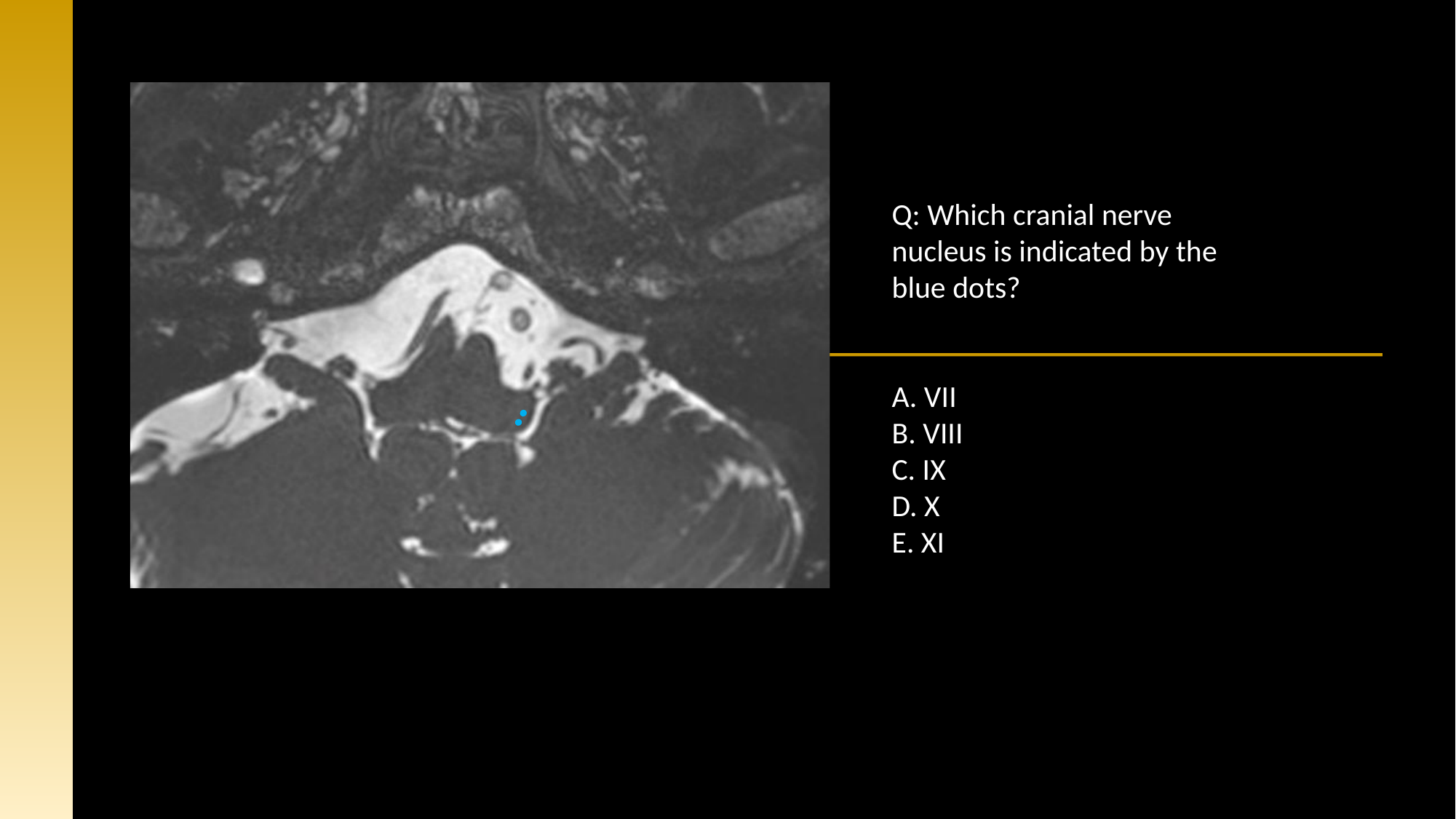

Q: Which cranial nerve nucleus is indicated by the blue dots?​
A. VII
B. VIII
C. IX
D. X
E. XI

## Slide 4
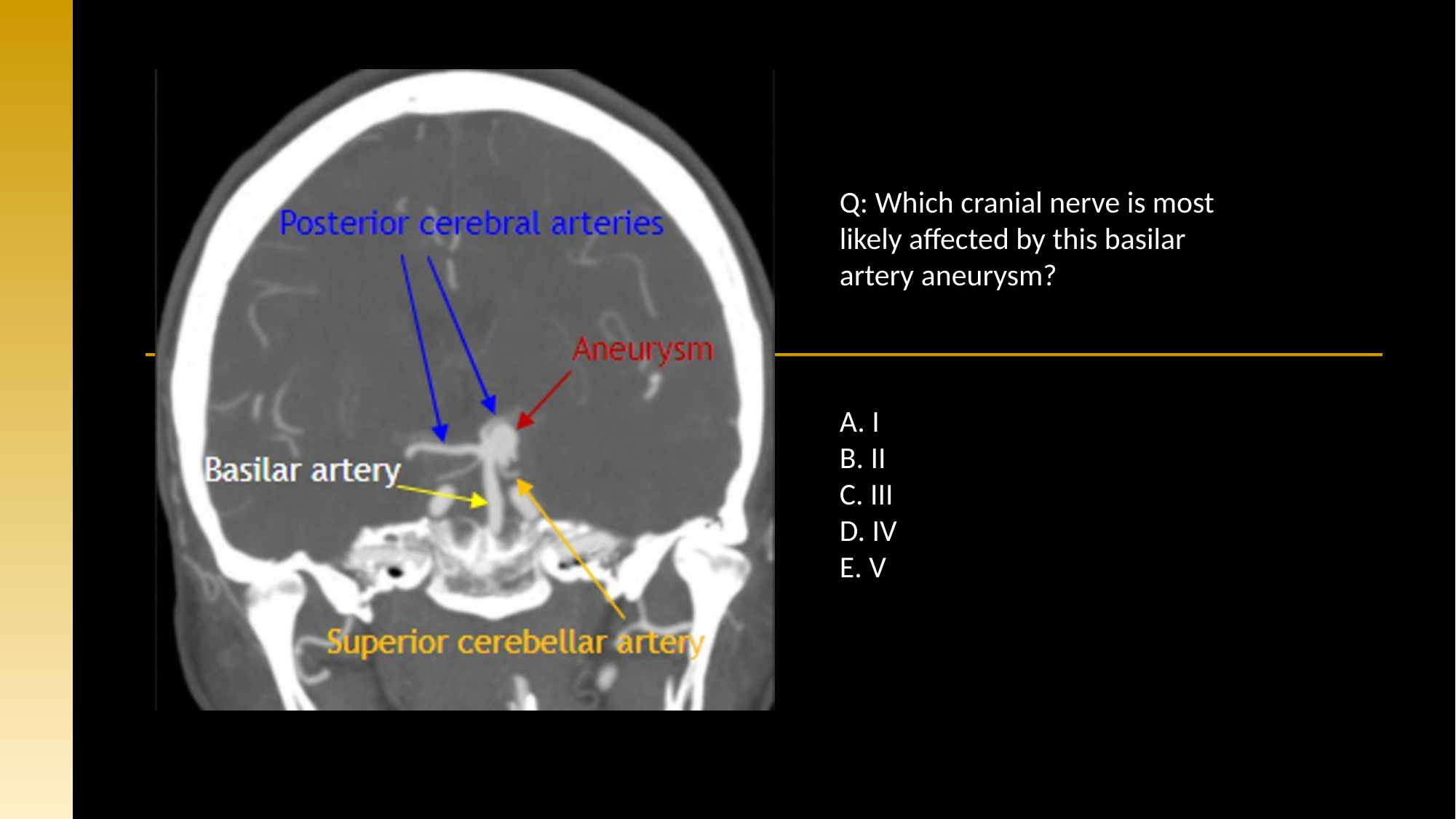

Q: Which cranial nerve is most likely affected by this basilar artery aneurysm?​
A. I
B. II
C. III
D. IV
E. V

## Slide 5
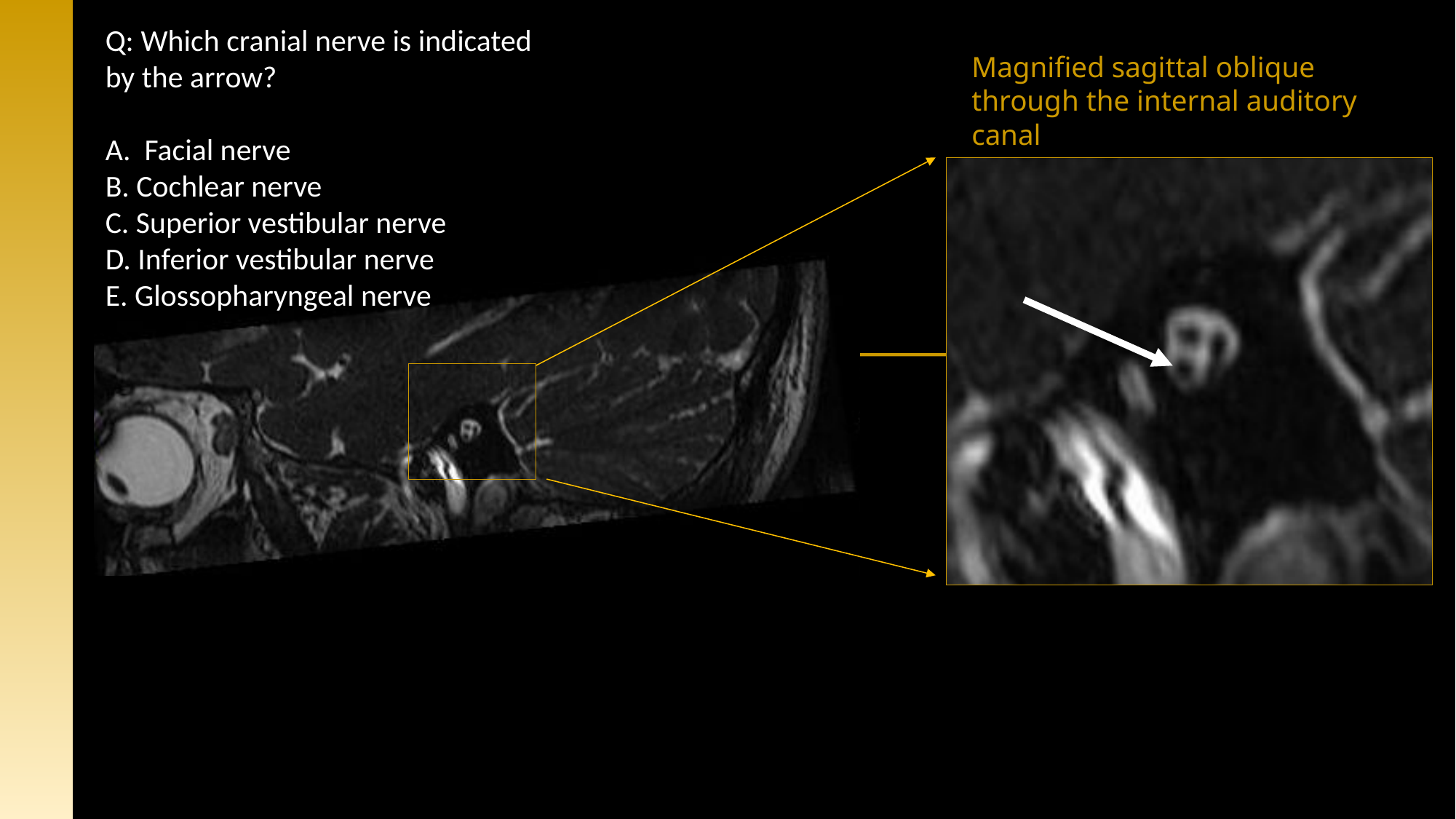

Q: Which cranial nerve is indicated by the arrow?​
A.  Facial nerve
B. Cochlear nerve
C. Superior vestibular nerve
D. Inferior vestibular nerve
E. Glossopharyngeal nerve
Magnified sagittal oblique through the internal auditory canal

## Slide 6
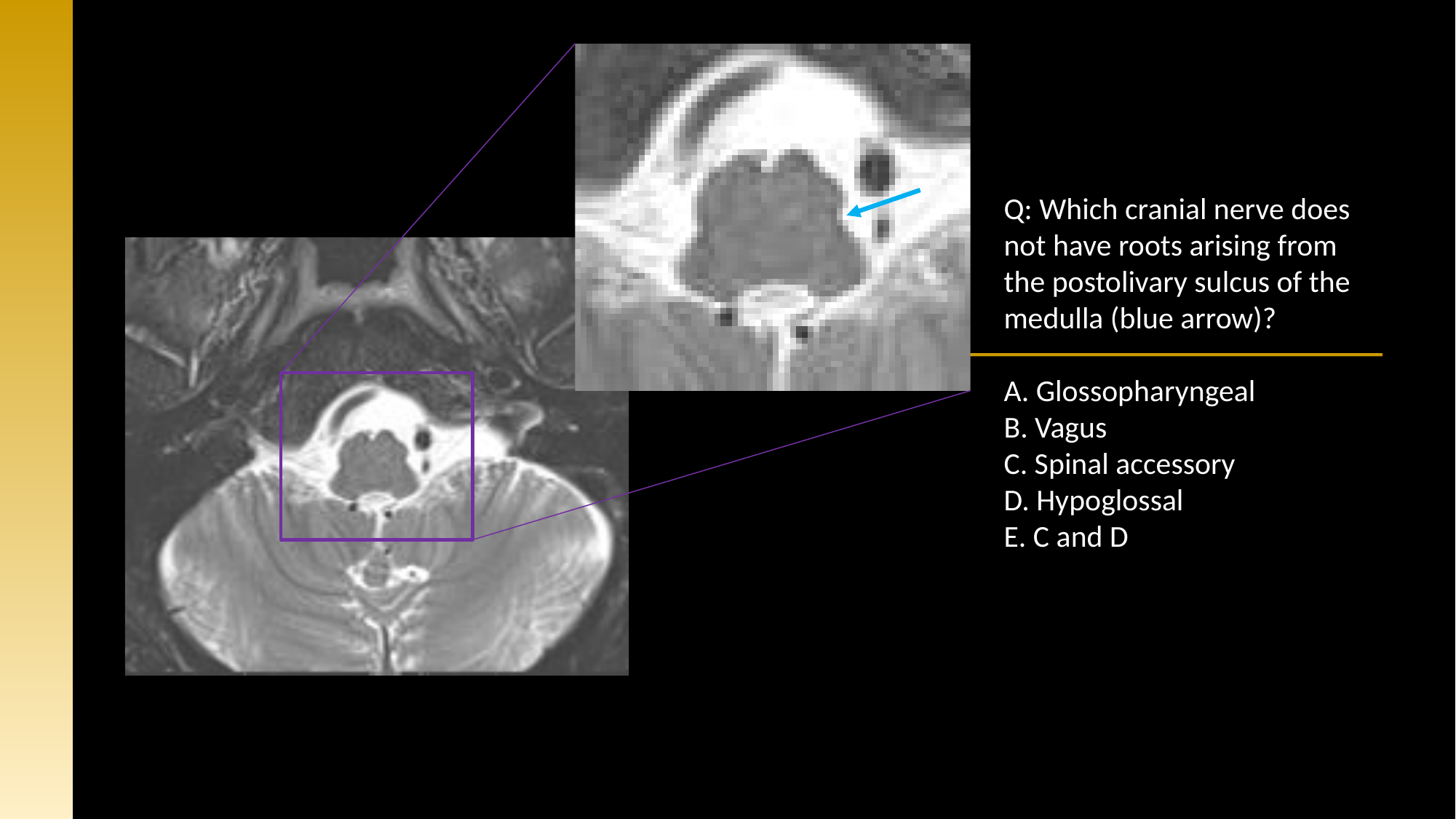

Q: Which cranial nerve does not have roots arising from the postolivary sulcus of the medulla (blue arrow)? ​
A. Glossopharyngeal
B. Vagus
C. Spinal accessory
D. Hypoglossal
E. C and D

## Slide 7
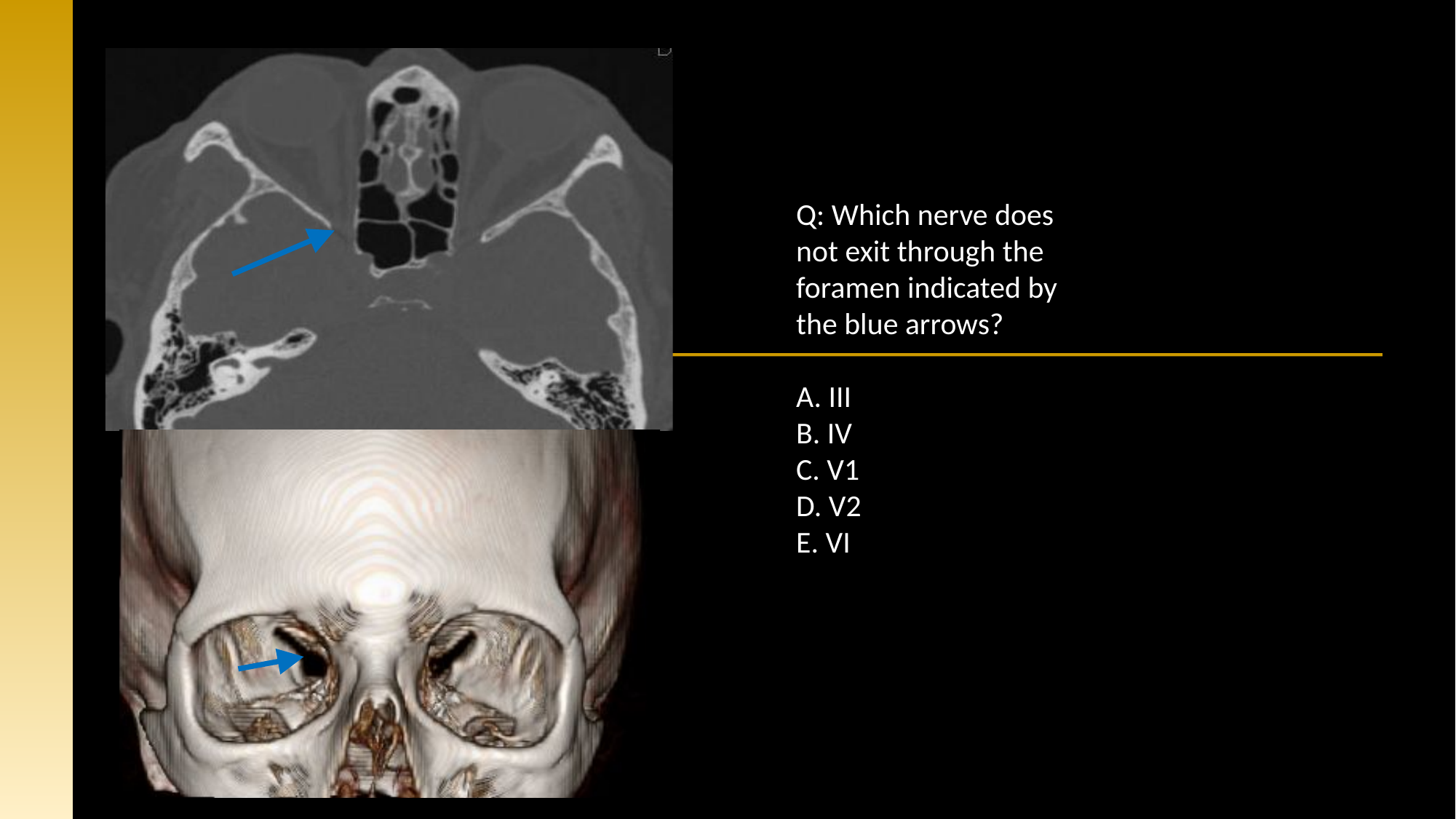

Q: Which nerve does not exit through the foramen indicated by the blue arrows?​
A. III
B. IV
C. V1
D. V2
E. VI

## Slide 8
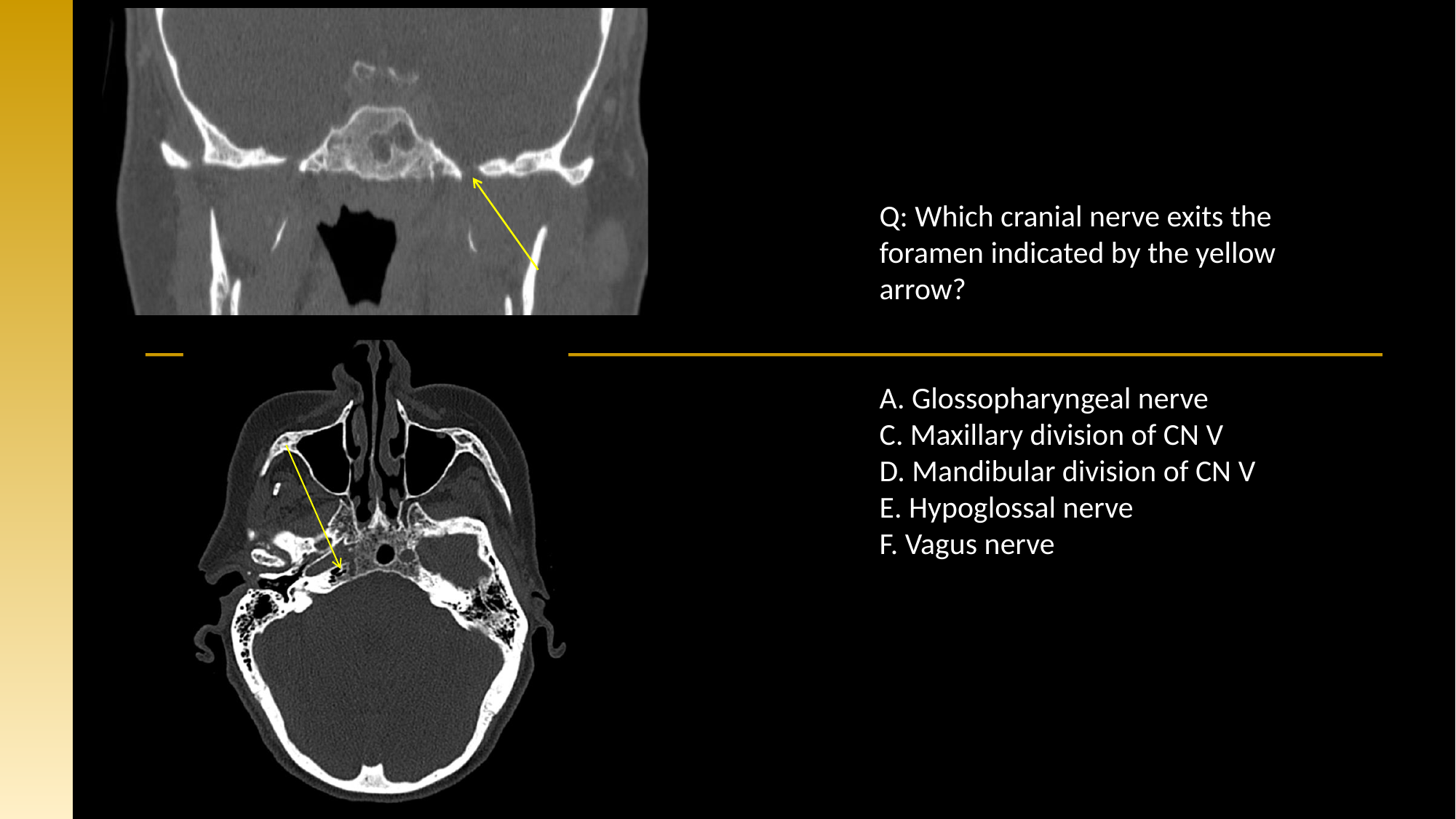

Q: Which cranial nerve exits the foramen indicated by the yellow arrow?​
A. Glossopharyngeal nerve
C. Maxillary division of CN V
D. Mandibular division of CN V
E. Hypoglossal nerve
F. Vagus nerve

## Slide 9
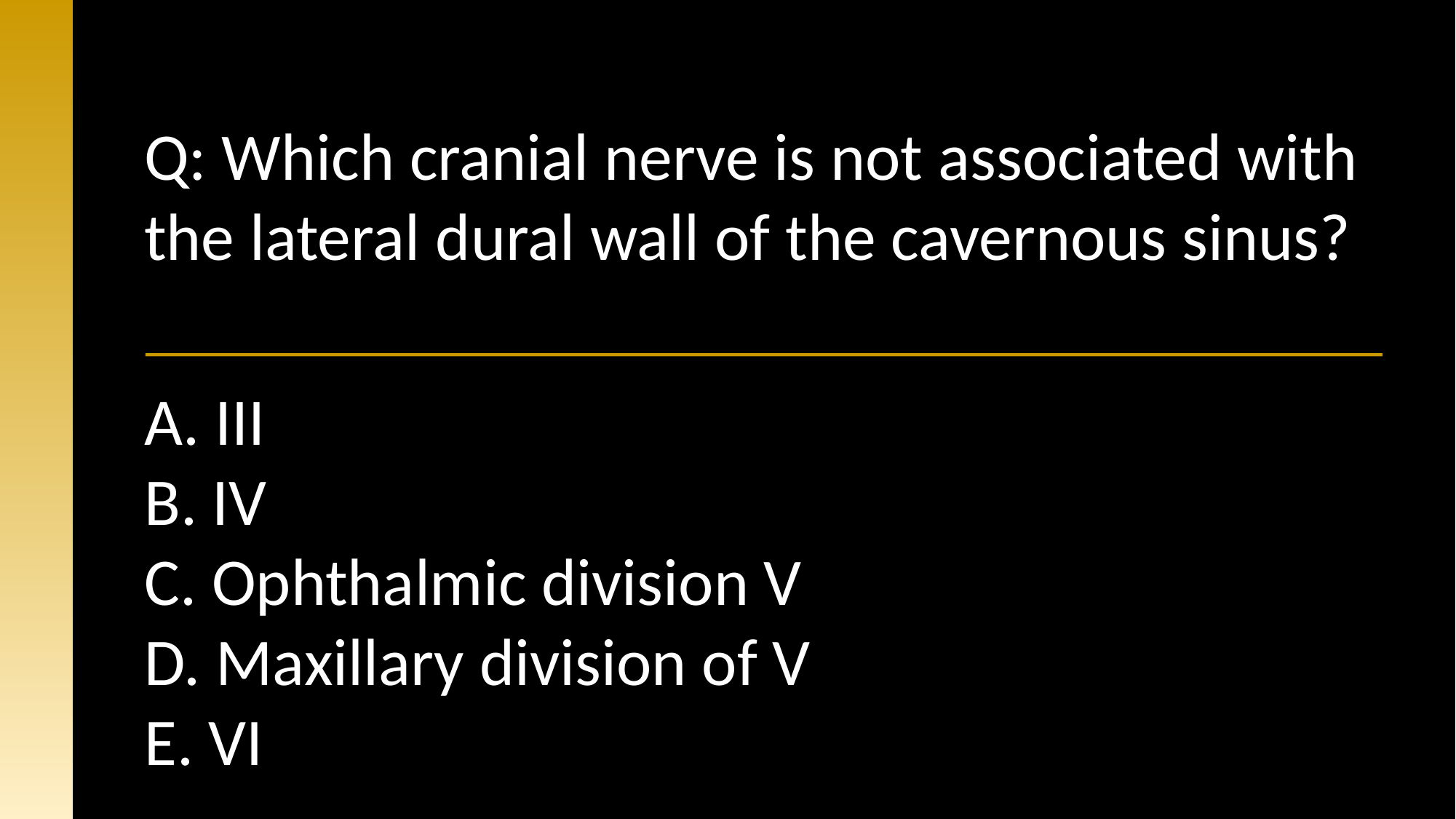

Q: Which cranial nerve is not associated with the lateral dural wall of the cavernous sinus?
A. III
B. IV
C. Ophthalmic division V
D. Maxillary division of V
E. VI
Click to add text

## Slide 10
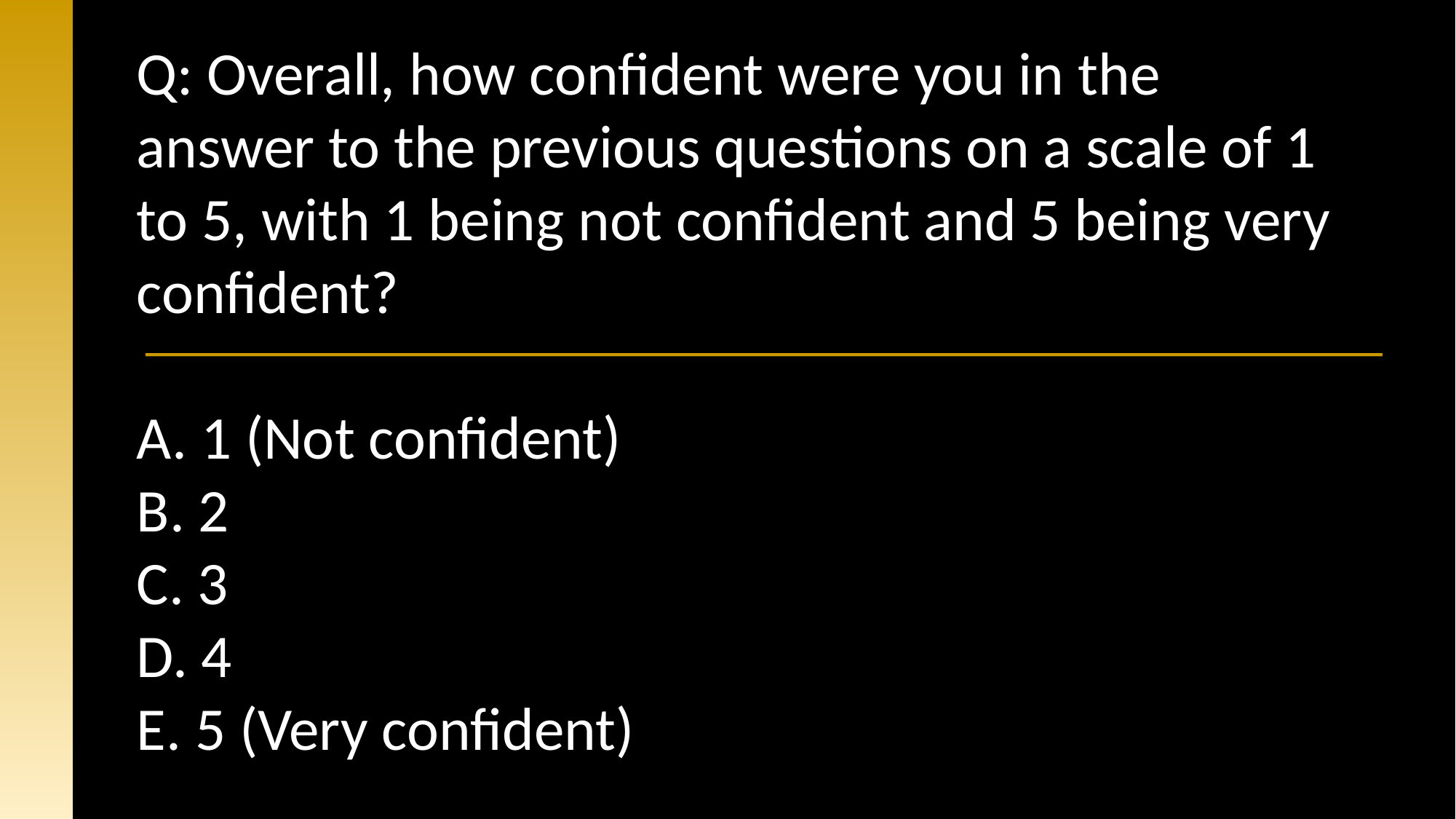

Q: Overall, how confident were you in the answer to the previous questions on a scale of 1 to 5, with 1 being not confident and 5 being very confident?​
A. 1 (Not confident)
B. 2
C. 3
D. 4
E. 5 (Very confident)
