# Supplementary material for: Cranial Nerve Anatomy Using a Modular and Multimodal Radiologic Approach
Source: MedEdPORTAL. 2022 Jun 10;18:11261. doi: 10.15766/mep_2374-8265.11261 (PMC9184306; doi:10.15766/mep_2374-8265.11261)
Supplement: Supplementary file 1 — Self-guided Anatomy Review.pptxCranial Nerve Video.mp4Cranial Nerve Lecture.pptxNeuroanatomy Lab.pptxNormal MRI and CT Scans - CT Bone Axials.pptxNormal MRI and CT Scans - T1 Sagittal.pptxNormal MRI and CT Scans - T2 Axial.pptxNormal MRI and CT Scans - T2 SPACE Axial.pptxPre- and Posttest.pptxSatisfaction Survey.docxAppendix Guide.docx [file mep_2374-8265.11261-s001.zip › K. Appendix Guide.docx]

**Guide to Using the Appendix Resources**

Appendix A - Self-Guided Anatomy Review

**Description:** This is a normal axial MRI with labeled visible cranial nerve segments (predominantly the cisternal segments) and relevant adjacent anatomic structures, such as the skull base foramina through which the nerves travel. It was formatted to allow students to scroll through images either using a mouse or the keyboard. Note that only right or left side of the scan is labeled, depending on structure, to allow visualization of the anatomy without labels on the opposite side.

**Setting:** Designed as a self-guided pre-learning prior to the cranial nerve lecture.

**Time:** 10 minutes

Appendix B - Cranial Nerve Video

**Description:** A video on cranial nerve anatomy narrated by a neuroradiology faculty member.

**Setting:** Designed as a pre-learning prior to the cranial nerve lecture. Suggest pausing the video as needed and using Appendix A to identify the structures.

**Time:** 20 minutes

Appendix C - Cranial Nerve Lecture Slide Deck

**Description:** This is an in-person cranial nerve lecture created as a PowerPoint presentation delivered to the students a few days prior to the neuroanatomy lab. The lecture itself can be led by a radiologist. The lecture covers normal anatomy of cranial nerves I-XII using imaging (CT, MRI) and pathologic cases to highlight the anatomy and importance of understanding the anatomy. During this lecture, basic concepts of the anatomic divisions of the cranial nerves including nuclei, central course, cisternal course, skull base exit sites and peripheral courses are introduced. These skills are then built upon during the neuroanatomy lab section of the lesson.

**Setting:** The students can attend this lecture in-person, watch it live remotely, or watch it at a later time-point if recorded.

**Time:** 50 minutes

Appendix D - Neuroanatomy Labs and Answers

**Description:** This is a PowerPoint presentation with lab instructions and six clinical cases with imaging. It was divided into three sections – brain, upper cranial nerves, and lower cranial nerves. Normal scans including CT Head, MRI Brain and Cranial Nerves containing high resolution T2 weighted imaging (Appendices E-H) were uploaded to the course page and accessible to students to download.

*Please note information and answers to the questions in the comment section of PowerPoint on each slide.

**Setting:** This is designed as an in-person, interactive lab given after students have attended or viewed the cranial nerve lecture (Appendix C). Students work in breakout groups consisting of 3-4 students. At the beginning of each section, students are given a list of anatomical structures to identify on MRI and CT imaging. During this time, radiology faculty members circulate the room to assist the students in identifying the structures. After completion of each section, students work through clinical cases with images.

**Time:** 1 hour 50 minutes

Appendices E-H - Normal MRI and CT Scans

**Description**: These appendices include MRI and CT head scans to be used during the neuroanatomy lab when identifying anatomical structures. The images have been converted to be displayed in a PowerPoint presentation.

**Setting**: Used during the in-person, interactive neuroanatomy lab.

**Time**: 1 hour 50 minutes

Appendix I – Pre- and Posttest

**Description**: Nine multiple-choice topic-based questions designed around the educational objectives. Following the content-related questions, students are asked to rate their confidence in their answers on a scale of 1-5.

*Please note answers to the questions in the comment section of PowerPoint on each slide.

**Setting**: Pretest to be completed prior to the cranial nerve lecture. Posttest to be completed immediately following the neuroanatomy lab.

**Time**: 15 minutes

Appendix J - Satisfaction Survey Document

**Description**: This is an anonymous survey given to students who completed the neuroanatomy lab. Students are asked to rate their confidence level with knowledge obtained and satisfaction with the lab.

**Setting**: Completed electronically following the neuroanatomy lab.

**Time**: 5 minutes

Appendix K – Appendix Guide

This document
